# d-Glucuronate and d-Glucuronate Glycal Acceptors for the Scalable Synthesis of d-GlcN-α-1,4-d-GlcA Disaccharides and Modular Assembly of Heparan Sulfate

**DOI:** 10.1021/acs.joc.3c01108

**Published:** 2023-07-17

**Authors:** Imlirenla Pongener, Gavin J. Miller

**Affiliations:** School of Chemical and Physical Sciences & Centre for Glycoscience, Keele University, Keele, Staffordshire ST5 5BG, U.K.

## Abstract

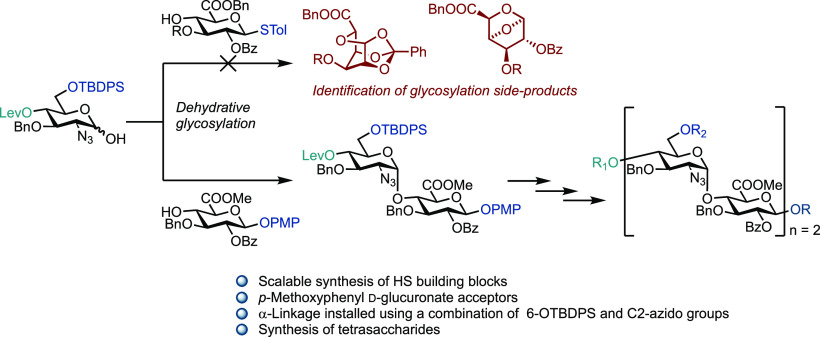

Reported herein is a scalable chemical synthesis of disaccharide building blocks for heparan sulfate (HS) oligosaccharide assembly. The use of d-glucuronate-based acceptors for dehydrative glycosylation with d-glucosamine partners is explored, enabling diastereoselective synthesis of appropriately protected HS disaccharide building blocks (d-GlcN-α-1,4-d-GlcA) on a multigram scale. Isolation and characterization of key donor (1,2 glycal)- and acceptor (ortho-ester, anhydro)-derived side products ensure methodology improvements to reduce their formation; protecting the d-glucuronate acceptor at the anomeric position with a *para*-methoxyphenyl unit proves optimal. We also introduce glycal uronate acceptors, showing them to be comparative in reactivity to their pyranuronate counterparts. Taken together, this gram-scale access offers the capability to explore the iterative assembly of defined HS sequences containing the d-GlcN-α-1,4-d-GlcA repeat, highlighted by completing this for two tetrasaccharide syntheses.

## Introduction

Heparan sulfate (HS) is a linear, highly sulfated glycosaminoglycan (GAG) present on most animal cell surfaces and in the surrounding extracellular matrix. This ubiquitous polysaccharide mediates mammalian cell functions (for example, cell proliferation, differentiation, and angiogenesis), alongside pathological conditions including cancer, Alzheimer’s disease, and viral infections, such as SARS-CoV-2, HIV, and HSV.^1−8^ Accordingly, there is a longstanding requirement to efficiently synthesize defined HS fragments,^3,9−18^ to provide materials for the study of its structure-to-function relationships, and to explore new therapeutic avenues.^19−23^

**Figure 1:**
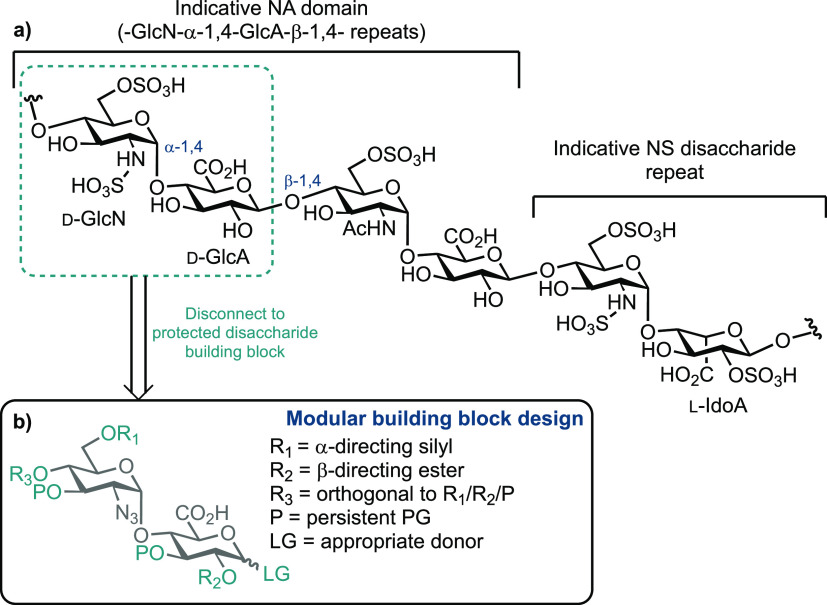
(a) Overview of chemical structure of HS, highlighting common core disaccharide and NA (blue dotted box) and NS domains. (b) Design concept for disaccharide building blocks to effect modular HS assembly using d-GlcN-α-1,4-d-GlcA donors.

The chemical structure of HS is complex. Broadly, it consists of repeating disaccharide units composed of glucosamine (d-GlcN) and a uronic acid (Figure 1a). Beyond this, the amino sugar can be N-sulfated (d-GlcNS) or N-acetylated (d-GlcNAc); the uronic acid d-GlcA can be epimerized to l-IdoA and saccharide units are variably sulphated, most commonly at *O*-6 of d-GlcN and *O*-2 of l-IdoA. At a macrostructural level, HS polysaccharides display distinct regions, termed NA and NS domains, broadly conferring lower (NA) or higher (NS) levels of backbone O/N-sulfation and l-IdoA content. Mixed NA/NS domains also exist and thus NA domains are key in enabling HS to regulate the extracellular matrix (ECM). This is typified by heparanase, an endo-β-glucuronidase that cleaves d-GlcA-β-1,4-d-GlcN linkages within all domain types and releases HS fragments as part of the degradation of HS and remodeling within the ECM. Synthetic sequences that mimic NA domains are thus of high value as biochemical tools, alongside providing substrates to complete enzymatic modifications, such as uronate C5 epimerization or O/N-sulfation, to access related NS domain sequences.^16,21^

Within such a context, we sought to develop a robust and scalable synthesis of d-GlcN-α-1,4-d-GlcA building blocks and evaluate their capability for modular oligosaccharide synthesis. This design concept is highlighted in Figure 1b. An impressive number of HS building blocks and oligosaccharide syntheses have been completed, and these have been reviewed recently.^14,15^ Briefly, a common strategy is to effect iterative [2 + 2] oligosaccharide assembly, building from the reducing end upward, although examples have been reported using other iteration blocks, such as [4 + 4].^24^ Despite this, there exists no universal method to assemble HS sequences, in part due to the complexity of any oligosaccharide design being dependent on the final sequence identity but also due to the range of options/preference for synthesis, such as pre- or post-glycosylation oxidation of d-GlcA/d-Glc or l-IdoA/l-Ido components. In this respect, disaccharide building block components with a d-GlcA-reducing (donor) end are of interest, and their stereoselective construction has received significantly less attention than l-IdoA-containing counterparts.^10,25−28^ The synthetically challenging d-GlcN α-1,4 linkage is commonly (but not ubiquitously) installed within a given disaccharide building block first, circumventing complex diastereomeric separation issues during later oligosaccharide assembly. Given this and our desire to explore d-GlcA disaccharide donors for modular HS synthesis, we targeted gram-scale access to building blocks of the type shown in Figure 1b.

## Results and Discussion

### Monosaccharide Building Blocks

**Figure 2:**
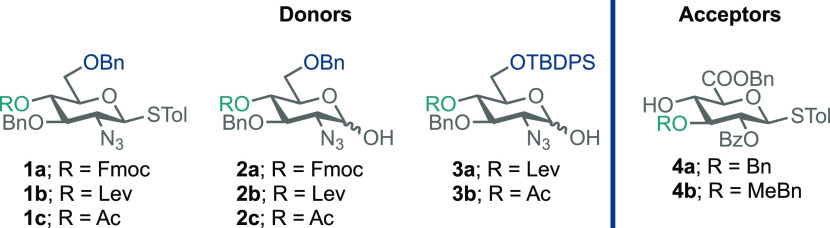
Panel of donor and acceptor components to explore d-GlcN-α-1,4-d-GlcA disaccharide synthesis.

To establish robust glycosylation and access d-GlcN-α-1,4-d-GlcA disaccharides, we wished to explore protecting-group influence (within constituent monosaccharides) upon the glycosylation outcome (Figure 2). Accordingly, we prepared glucosamine donors, **1a–c**, **2a–c**, and **3a,b**. d-GlcN was masked with a non-participating azide at C-2, reasoning that this would promote α-selectivity during glycosylation, and *O*-6 with a bulky silyl or carbon ether.^29^ Finally, *O*-4 was masked with temporary protecting groups (Fmoc, Lev, or Ac) to later reveal acceptor capability for glycosidation.

In addition, glucuronate acceptors **4a** and **4b** were designed with an ester at C-2 to introduce a 1,2-*trans* linkage upon later iterative glycosylation. The 3-*O* position was protected with either Bn or MeBn to facilitate a comparative evaluation of acceptor reactivity. Details of the synthesis of **1**–**4** can be found in the Supporting Information.

### Targeting Thioglycoside Donor Disaccharides

**Scheme 1:**
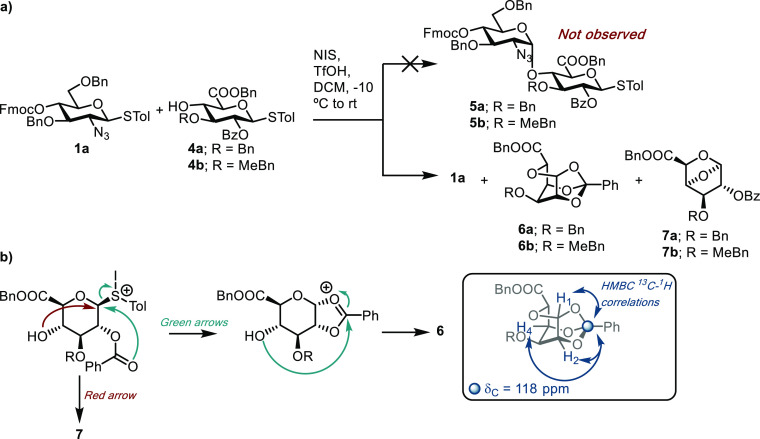
(a) Initial Orthogonal Glycosylation Attempts Using Thioglycoside Donor and Acceptor Components with an NIS/TfOH Promoter System (b) Plausible Mechanism for the Formation of Acceptor Side-Products 6 and 7 alongside Observed HMBC NMR Correlations for 6

We first trialed thioglycoside donor **1a** and uronate acceptors **4a** and **4b** using NIS/TfOH activation (Scheme 1), reasoning that preferential activation of **1a** might be possible in the presence of disarmed thioglycoside **4a** or **4b**.^29−31^ This would then enable rapid access to manipulable thioglycoside disaccharide donors. Unfortunately, TLC and ^1^H NMR analysis of these reactions revealed no indication that **5a** or **5b** had formed. Increasing the amount of TfOH (to stoichiometric relative to the donor) or pre-activation of the donor made no change to the reaction outcome. From these reactions we isolated unreacted **1a**, indicating aglycone transfer had likely occurred,^32^ alongside two intriguing side-products, tentatively assigned here as tricyclicortho-ester **6** and 1,4-anhydro derivative **7**, and both derived from the acceptor; no **4a** or **4b** was recovered from reaction mixtures.

^13^C NMR for **6a** displayed only one carbonyl carbon at δ_C_ = 168.6 ppm, which had cross-peaks to benzylic protons (δ_H_ = 5.04–5.22 ppm), identifying this as the uronate carbonyl. Furthermore, a new quaternary carbon at δ_C_ = 118 ppm displayed heteronuclear multiple bond correlation (HMBC) cross-peaks to H-1, H-2 and H-4; related tricyclic ortho esters have been reported in the glucopyranose series.^33−35^ For **7**, H-1 exhibited a new HMBC cross-peak to C-4.^36^ The ^3^*J* coupling constants for both **6** and **7** suggested a deviation from the ^4^*C*_1_ conformation (for **6**: ^3^*J*_H1–H2_ = 4.9 Hz, ^3^*J*_H2–H3_ = 2.1 Hz, ^3^*J*_H3–H4_ = 4.8 Hz; for **7**: ^3^*J*_H1–H2_ ≈ 1.0 Hz, ^3^*J*_H2–H3_ = 2.0 Hz, ^3^*J*_H3–H4_ = 4.8 Hz).^33,37^ Electrospray ionization-high-resolution mass spectrometry (ESI-HRMS) data further supported these assignments, and indicative mechanisms for the formation of **6** and **7** are shown in Scheme 1b.

Considering these unsuccessful attempts at orthogonal thioglycoside activation, we turned to work reported by Marel and co-workers.^10^ Their protocol saw pre-activation of related hemiacetal donors under conditions established by Gin (Ph_2_SO/Tf_2_O)^38^ to glycosidate GlcA and IdoA acceptors. Accordingly, glycosylations were performed between donors **2a–c** and **3a,b** and uronate acceptors **4a** and **4b** (Table 1). Glycosylation using 4-*O*-Fmoc-protected donor **2a** with acceptor **4a** gave complex mixtures by ^1^H NMR and TLC analysis, and the desired product **5a** was only isolated in a low yield of 14% (Table 1, entry 1). Switching the temporary 4-*O*-protecting group to Lev (**2b**) or Ac (**2c**) improved the glycosylation outcome, and moderate yields were obtained (41% for **8a** and 52% for **9a**) (Table 1, entries 2 and 3). However, the glycosylation for **2c** was not α-selective and delivered **9a** as a 9/1 α/β mixture (Table 1, entry 3). Using acceptor **4b** (with 3-*O*-methyl benzyl in place of benzyl), glycosylation results were less favorable; a very low yield was observed for **8b** (11%, Table 1 Entry 4), alongside ortho-ester **6b** which was isolated as the major product (36% yield). A similar loss of glycosylation stereoselectivity was noted when using donor **2c** (Table 1, Entry 5).

**Table 1:**
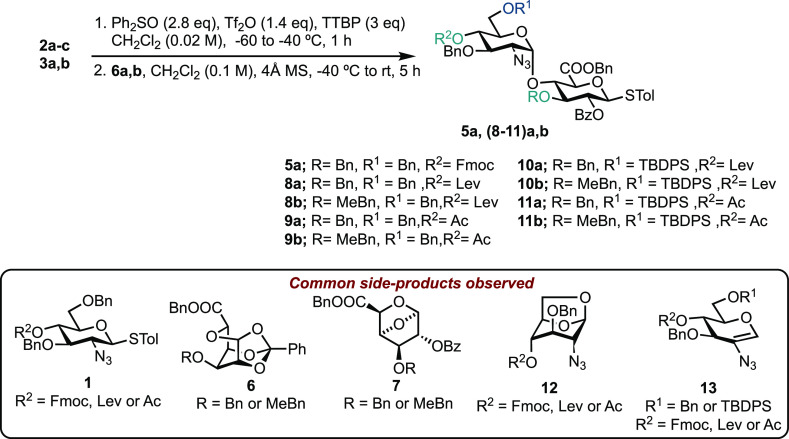
Glycosylation of Glucoazide Hemiacetals Using a Ph_2_SO/Tf_2_O Promoter System

entry	donor	acceptor	product	yield %	α/β^a^	side-products observed
1	**2a**	**4a**	**5a**	2		1, 6, 7, 12, 13
2	**2b**	**4a**	**8a**	41		1, 6, 7, 12, 13
3	**2c**	**4a**	**9a**	52	89:11	1, 6, 7, 12, 13
4	**2b**	**4b**	**8b**	11		1, 6, 7, 12, 13
5	**2c**	**4b**	**9b**	45	83:17	1, 6, 7, 12, 13
6	**3a**	**4a**	**10a**	45	α-only	1, 6, 7, 13
7	**3a**	**4b**	**10b**	38	α-only	1, 6, 7, 13
8	**3b**	**4a**	**11a**	46	α-only	1, 6, 7, 13
9	**3b**	**4b**	**11b**	48	α-only	1, 6, 7, 13

a α/β ratios were determined by ^1^H NMR of the crude reaction mixture; for entries 1, 2, and 4, α/β ratios could not be determined.

Hemiacetals **3a,b**, bearing a bulky silyl ether at C6, were selected next, with a view to increase (1) donor reactivity and (2) α-selectivity.^29^ Glycosylation using these donors with acceptors **4a** or **4b** showed excellent α-selectivity (Table 1, entries 6–9). Unfortunately, this partnered with consistent low isolated yields for these reactions, alongside significant side-product formation. In general, for the systems evaluated in Table 1, it was observed that thioglycoside glucuronate acceptors **4a,b** were not fully consumed during the reaction, and a range of side-products from both the donor (1,6-anhydro system **12**, glycal **13**, and aglycone transfer product **1**) and acceptor (ortho-ester **6** and 1,4-anhydro derivative **7**) were observed. These are highlighted and contained within Table 1 (notably any formation of **12** was removed when using donors of type **3**).

From these results, we settled on **3a/4a** as an optimal donor/acceptor system given the excellent α-selectivity observed, albeit only forming **10a** in a moderate yield. Accordingly, we attempted a gram-scale synthesis of disaccharide **10a**. Disappointingly, this reaction was unsuccessful, despite several attempts. ^1^H NMR of crude reaction mixtures showed very little product formation. Glycal **13a** and ortho-ester **6a** were the predominant side-products from the donor and acceptor, respectively. A plausible explanation for this outcome is that under these glycosylation conditions the glucuronate acceptor thioglycoside was activated, leading to the formation of **6a** and with no acceptor remaining, the activated donor underwent elimination to give **13a**. As a final attempt to access **10a**, we synthesized an imidate donor from **2b** but only observed aglycone transfer upon attempted glycosylation.

### Modifying the d-Glucuronate Acceptor Reducing End

Having evaluated several modifications to the glucosamine donor component (in terms of anomeric leaving group and ring-protecting groups), we contended that the main issue in our glycosylation reactions presented from the anomeric group in the acceptor. To explore overcoming this, the anomeric group was changed to *p*-methoxyphenol and d-GlcA acceptor **14** was synthesized accordingly from commercial peracetylated glucose (see Supporting Information). Glycosylations were then attempted between hemiacetal **3a** and d-GlcA acceptor **14** using the same Ph_2_SO/Tf_2_O promoter system as previously (Table 2).

**Table 2:**
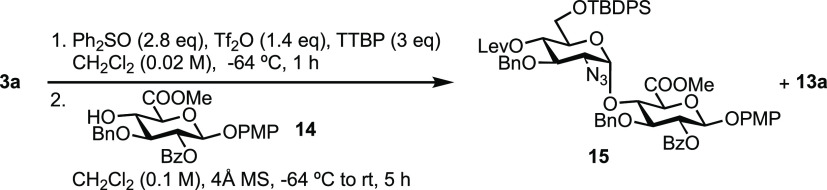
Optimization and Scale-Up Glycosylations Using Ph_2_SO/Tf_2_O Promoter System

entry	donor scale (g)	**14** (eq)	isolated yield (%)	**15**/**13a** ratio^a^
1	0.10	0.8	30	50:50
2	0.10	1.5	39	73:27
3	0.10	2.0	45	78:22
4	0.98	2.0	55	84:16
5^b^	2.00	2.0	67	93:7
6^b^	3.00	2.0	67	96:4
7^b^	3.50	2.0	73	95:5

a Ratio determined by ^1^H NMR analysis of reaction mixture.

b Step 1: CH_2_Cl_2_ (0.04 M), step 2: CH_2_Cl_2_ (0.2 M), reaction was kept at −60 °C for 1.5 h and then at −45 °C for about 5 h and then slowly warmed up to −10 °C (total time 8 h, instead of 5 h, after addition of acceptor).

Expectedly, glycosylations using **14** led to no formation of the previously problematic aglycone side-product **6a**. However, glycal side-product **13a**, which forms from the donor, was still evident in the crude reaction. Increasing the equivalents of **14** led to (1) suppression of **13a** forming and (2) a small increase in the yield of the desired disaccharide **15** (Table 2, entries 1–3). Furthermore, the yield was improved to 67% upon increasing to gram-scale synthesis and by maintaining the reaction at −45 °C for a longer period of time (Table 2, entry 5). The combination of low temperature and longer reaction time was likely crucial to prevent decomposition (to **13a**) of an active triflate intermediate. The excess acceptor employed could be recovered after the reaction using purification by column chromatography and subsequent washing with Et_2_O to remove excess Ph_2_SO. Overall, this delivered a robust and scalable entry (up to 3.5 g of donor, Table 2 entries 6 and 7) to our desired d-GlcN-α-1,4-d-GlcA building block.

### Manipulation of d-GlcN-α-1,4-d-GlcA Donor Disaccharides

**Scheme 2:**
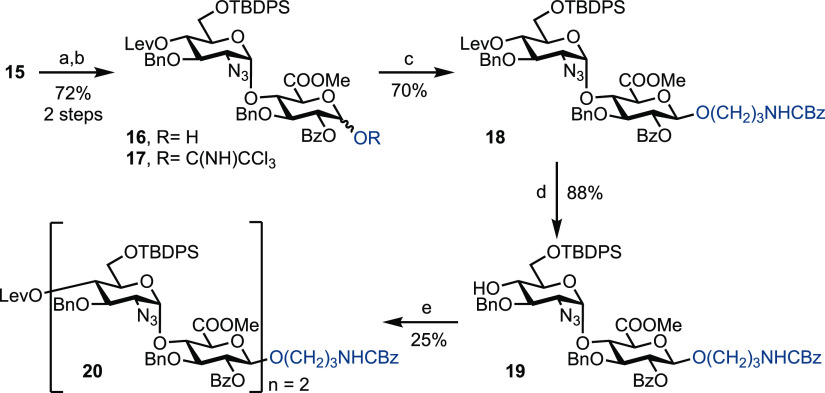
Transformations from Disaccharide 15 to Deliver a Protected NA Domain Tetrasaccharide, Equipped with Non-reducing End Handle Reagents: (a) CAN, Tol/MeCN/H_2_O (1:1.5:1), 76%; (b) CCl_3_CN, K_2_CO_3_, CH_2_Cl_2_, 95%; (c) HO(CH_2_)_3_NHCBz, TMSOTf, CH_2_Cl_2_, −15 to 0 °C, 70%; (d) N_2_H_4_.AcOH, CH_2_Cl_2_/MeOH, 88%; and (e) 17, TMSOTf, CH_2_Cl_2_, −15 to 0 °C, 25%.

With gram-scale access to disaccharide **15**, we next sought to demonstrate its utility for iterative glycosylation. Accordingly, the reducing end PMP group was oxidatively removed using CAN, affording **16** in a 76% yield (Scheme 2). Solvent choice proved important here, with a toluene/MeCN/H_2_O system (1:1.5:1) proving most successful. Attempts using CAN and MeCN/H_2_O (7:1) only afforded **16** in a 69% yield after two cycles of reactions and with unreacted starting material still present. Hemiacetal **16** was then converted to its corresponding imidate donor **17** and glycosylated with an *N*-benzyloxycarbonyl amino propanol linker to deliver **18** in a 67% yield, over the two steps.

Removal of the temporary protecting group at the non-reducing end C4 position within **18** was accomplished in an 88% yield using N_2_H_4_^.^AcOH to unmask a new acceptor, **19**. The same disaccharide imidate donor **17** was then used to glycosylate acceptor **19** with catalytic trimethylsilyl trifluoromethanesulfonate (TMSOTf) and deliver tetrasaccharide **20** but only in a poor yield (25%). From this reaction, we recovered mostly unreacted **19** (70%) alongside donor-derived side-products (an *N*-linked amide disaccharide and C1–C2 glycal). Taken together, we observed a low reactivity in the glycosidating acceptor **19**, possibly due to a steric effect to C4 caused by the bulky tert-butyldiphenylsilyl (TBDPS) group at C6 of the non-reducing end sugar.

**Scheme 3:**
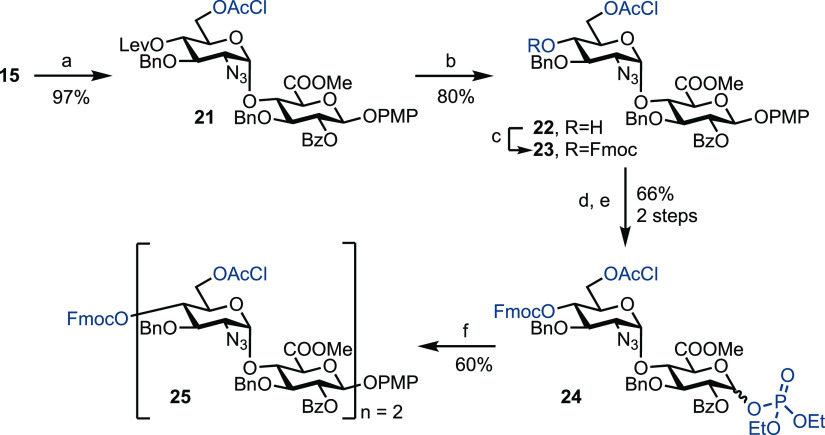
Exploring Phosphate Donors for Iterative HS Tetrasaccharide Synthesis Reagents: (a) (i) HF pyridine, pyridine, 0 °C to rt; (ii) ClCH_2_COCl, pyridine, DMAP, CH_2_Cl_2_, 97%, 2 steps; (b) N_2_H_4_.AcOH, CH_2_Cl_2_/MeOH, 80%; (c) FmocCl, pyridine, CH_2_Cl_2_, 89%; (d) CAN, Tol/MeCN/H_2_O (1:1.5:1), 0 °C, 78%; (e) ClPO(OEt)_2_, K_2_CO_3_, Cs_2_CO_3_, CH_2_Cl_2_, 84%; and (f) **22**, TMSOTf, CH_2_Cl_2_, −45 °C to rt, 60%.

In an attempt to circumvent this observed low yield upon iterative glycosylation and to highlight a versatility for protecting-group manipulation within the disaccharide building block **15**, 6-OTBDPS was removed using HF^.^pyridine and replaced with chloroacetyl to give disaccharide **21** in a 97% yield (Scheme 3). It was reasoned that by replacing the bulky TBDPS with chloracetyl, this may avoid steric hindrance at the acceptor 4-OH during glycosylation. Furthermore, disaccharide glycosyl donors bearing 4-*O*-Lev/6-*O*-AcCl and derived from building block **21** were found to be very unreactive (results not shown), and hence it was decided to replace the 4-*O*-Lev with 4-*O*-Fmoc. Accordingly, the 4-*O*-Lev group was removed in the presence of 6-*O*-chloroacetyl in an 80% yield (7–14% of the 4,6-diol was also isolated) and the resultant free 4-OH protected with Fmoc to give disaccharide **23** in an 89% yield. It was necessary to use an excess of FmocCl (10 equivalents) to drive this reaction to completion. Given the *N*-linked amide side-product observed when using imidate donor **17**, we decided to trial a phosphate-leaving group. The anomeric p-methoxyphenyl (PMP) group was thus cleaved using the conditions established previously, and the resultant hemiacetal converted to phosphate donor **24** using diethyl chlorophosphate and a combination of K_2_CO_3_ and Cs_2_CO_3_ in a 66% yield over the two steps, noting that the Fmoc group was stable under these conditions. Finally, glycosylation using donor **24** and stoichiometric TMSOTf successfully furnished tetrasaccharide **25** in an improved yield of 60% and demonstrates the first example of a phosphate-based disaccharide donor for iterative HS synthesis.

### Exploring Disaccharide Synthesis Using a d-Glucuronate Glycal Acceptor

Given the issues encountered using thioglycoside d-GlcA donors and the subsequent additional steps needed to manipulate a PMP anomeric group, we decided to also explore employing glycal uronate acceptors. It was envisioned that using a glycal acceptor could deliver (1) a more reactive acceptor, owing to the absence of two hydroxyl substituents at C1/C2, (2) a shorter synthesis route (5 steps for **29***vs* 8 steps for **14**), and (3) eliminate protecting-group regioselectivity requirements (*e.g.*, *O*-3 benzylation). The use of glycals as precursors to l-iduronic and d-glucuronic acid components has been reported previously.^39−43^ However, the use of uronic acid glycals as glycosyl acceptors has not been explored, and we envisioned that disaccharide reducing end donor capability could later be effected through manipulation of the glycal at the disaccharide level; for example, Seeberger and co-workers have reported an efficient synthesis of glycosyl phosphates from 1,2-glycals in a one-pot, three-step route.^44^

**Scheme 4:**
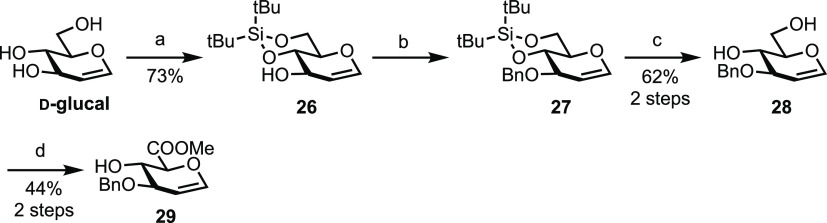
Synthesis of d-Glucuronate Acceptor 29 Reagents: (a) (^*t*^Bu)_2_Si(OTf)_2_, pyridine, DMF, −40 °C to rt, 73%; (b) NaH, BnBr, DMF; (c) TBAF, THF, 62% over 2 steps; and (d) (1) TEMPO, PIDA, CH_2_Cl_2_/H_2_O (2:1), 0 °C – rt, (2) MeI, K_2_CO_3_, DMF, rt, 44% over 2 steps.

Glucuronic acid glycal **29** was thus synthesized in five steps from commercial d-glucal (Scheme 4). Regioselective silylene protection of the 4- and 6-OH groups in d-glucal was first completed to give alcohol **26** in a 73% yield. This was followed by benzylation of the remaining hydroxyl group to furnish **27** and, following silylene deprotection using TBAF, diol **28** was isolated in a 62% yield over the two steps from **26**. Finally, a TEMPO-mediated oxidation and esterification of the resultant carboxylic acid delivered the desired acceptor **29** in a 44% yield over two steps. Notable during this synthesis development was that the C6 oxidation and esterification reactions for glycal diol **28** were slower compared to that observed for glycosyl diol **34** (15 h *vs* 2 h). In addition to this, diminished yields were also noted (44% for **29***vs* 72% for **14**, over two steps). Overall, glucuronate glycal acceptor **29** was synthesized in a 20% yield over five steps, whereas glucuronate acceptor **14** was synthesized in a 27% yield over eight steps.

With glucal acceptor **29** in hand, glycosylations were performed using the previously established dehydrative conditions (Table 3). As observed when using glucuronate acceptor **14**, increasing the equivalents of acceptor from 1.0 to 1.5 led to a reduction of the donor derived glycal side-product **13a** and a slight increase in the yield of **29** (Table 3, entries 1–2). Adopting the optimized conditions used for **14**, glycosylation was attempted on a gram-scale using two equivalents of **29** (Table 3, entry 3). Pleasingly, the formation of **13a** was reduced, and the glycosylation yield was improved (60% *vs* 41%).

**Table 3:**
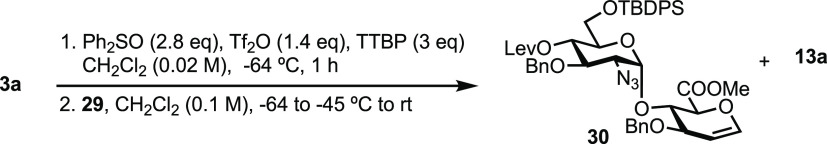
Optimization and Scale-Up Glycosylation Utilizing a Glycal Acceptor 29

entry	donor scale (g)	**29** (eq)	yield (%)	**30**/**13a** ratio^a^
1	0.10	1.0	30	61:39
2	0.10	1.5	41	82:18
3^b^	1.2	2.0	60	92:8

a Ratio determined by ^1^H NMR spectroscopic analysis of reaction mixture.

b Step 1: CH_2_Cl_2_ (0.05 M), Step 2: CH_2_Cl_2_ (0.3 M).

As a result of the successful implementation of glycal acceptor **29**, we next compared its capabilities with pyranuronate **14** (Table 4). At different donor scales and acceptor equivalents, there appeared to be negligible difference in the glycosylation yield (Table 4, entries 1 *vs* 2). However, the elimination side-reaction leading to glycal **13a** was reduced slightly when using the glycal acceptor **29** (Table, entries 3 *vs* 4). The difference observed in the rate of reaction to produce **13a** may indicate that acceptor **29** is more reactive than **14**. Finally, when completing these reactions on the gram scale, the glycosylation outcome performed well with both acceptors and with minimal side-product formation (Table 4, entries 5 *vs* 6).

**Table 4:**
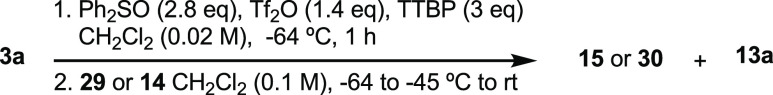
Comparison between Acceptors: Glucuronate 14 and Glucuronate Glycal 29

entry	donor scale (g)	ROH	ROH (eq)	yield (%)	**15** or **30**/**13a**ratio^a^
1	0.10	GlcA **14**	0.8	30	50:50
2	0.10	GlcA glycal **29**	1.0	30	61:39
3	0.10	GlcA **14**	1.5	39	73:27
4	0.10	GlcA glycal **29**	1.5	41	82:18
5	1.20	GlcA glycal **29**	2.0	60	92:8
6	2.00	GlcA **14**	2.0	67	93:7

a Ratio determined by ^1^H NMR spectroscopic analysis of reaction mixture.

Given their apparent similarity in completing dehydrative glycosylation on the gram scale, a competition reaction between acceptors **14** and **29** was performed to determine their relative reactivities (see Supporting Information). Following the established procedure, pre-activated hemiacetal **3** was treated with one equivalent each of **29** and **14**. Subsequent ^1^H NMR of the crude reaction mixture revealed a 50:50 mixture of disaccharides **30** and **15**. Additionally, unreacted **29** and **14** were observed: 64% remaining for **29** (based on **30** formed) and 78% remaining for **14** (based on **15** formed). From these observations, acceptor **14** is comparatively less reactive than glycal **29**. As such, the availability of acceptor **29** offers a scalable new option for the assembly of related HS disaccharide building blocks.

Finally, to set these results into a wider context of similar HS building blocks and synthetic methodologies thereto, we compare our results using donor **3a** and uronate acceptor **14** against alternative syntheses that delivered d-GlcN-α-1,4-d-GlcA materials with complete α-selectivity (Table 5). Our approach compares favorably with the closest related example (Table 5, entry 2),^10^ with both methods incorporating protecting-group orthogonality to the non-reducing sugar, but this system has the capability to differentiate O2 in d-GlcA and easily release a free reducing end. Examples utilizing locked acceptor components (but noting imidate or thioglycoside donors) can deliver suitably protected disaccharide systems (Table 5, entries 3–4)^25,45^ and link to the successes observed in using such approaches to afford l-IdoA-containing HS disaccharides.^28,46^ The additional requirement for these systems is to re-protect at disaccharide level upon release of the locking group. Lastly, our dehydrative glycosylation approach compares well to methods harnessing imidate donors (Table 5 entry 5).^47^

**Table 5:**
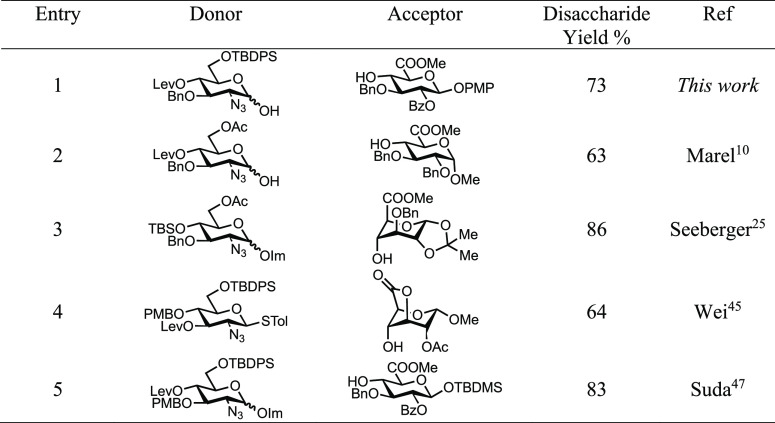
Summary of Access to d-GlcN-α-1,4-d-GlcA Disaccharides with Complete α-Selectivity

General patterns that arise from considering these results indicate that variation in the protecting group within the donor presents some flexibility. At C4, TBS, Lev, and PMB are effective, C3 benzyl is also tolerated, while C3-Lev has been shown to be problematic.^45^ Excellent glycosylation diastereoselectivity has been achieved with both large (TBDPS) and small (Ac) groups at d-GlcN C6, noting improved orthogonality for TBDPS regarding later transformations in oligosaccharide synthesis. Within acceptor components, C3–C5 and C1–C2 locking groups are effective, and there appears to be tolerance for the protecting group at C2 being an ether or an ester, while C3 prefers an ether (noting also that the work herein also demonstrates capability for methyl benzyl).

Taking these observations and patterns into account, the gram-scale dehydrative glycosylation delivering disaccharides of type **15** developed here offers an important addition to the urinate-level building block arsenal now available for the wider synthesis of complex HS targets, using both solution and solid phase synthesis.

## Conclusions

We have developed a robust methodology to synthesize d-GlcN-α-1,4-d-GlcA HS building blocks on a multigram scale. Using a d-GlcN hemiacetal donor and a hitherto unexplored D-GlcA acceptor, we identify several important side-products resulting from attempted glycosylation reactions and utilize this to refine the building block design and synthetic glycosylation methodology. Protecting the d-GlcA acceptor at the anomeric position with a *para*-methoxyphenyl unit proves optimal and greatly improves the glycosylation yield, alongside increasing the equivalents of acceptor. The required α-glycosidic disaccharide linkage was installed using a combination of a 6-OTBDPS and C2-azido protected d-GlcN donor. The TBDPS group increased the donor reactivity and additionally prevented the formation of 1,6-anhydro sugars which was observed when 6-OBn was used.

We also showcase the versatility and modularity for these building blocks, manipulating to both phosphate and imidate donors, and synthesizing two protected HS tetrasaccharides. Finally, we demonstrate the first examples of a glycal uronate as a glycosyl acceptor for HS disaccharide synthesis, confirming it to have a reactivity profile similar to the more common pyranuronate acceptor. Access to this capability and these materials will enable further exploring of their use in the synthesis of defined HS sequences, especially those that constitute NA domains within the ultimate glycosaminoglycan sequence.

## Data Availability

The data underlying this study are available in the published article and its Supporting Information.

## Supporting Information Available

Experimental procedures for all compounds (PDF)
Relevant 1D and 2D NMR spectra for all compounds (PDF)
