# Supplementary material for: d-Glucuronate and d-Glucuronate Glycal Acceptors for the Scalable Synthesis of d-GlcN-α-1,4-d-GlcA Disaccharides and Modular Assembly of Heparan Sulfate
Source: J Org Chem. 2023 Jul 17;88(15):11130–9. doi: 10.1021/acs.joc.3c01108 (PMC10407932; doi:10.1021/acs.joc.3c01108)
Supplement: Supplementary file 1 — jo3c01108_si_001.pdf [file jo3c01108_si_001.pdf]

D-Glucuronate and D-glucuronate glycal acceptors for the scalable synthesis of D-GlcN-α-1,4-D-GlcA disaccharides and modular assembly of heparan sulfate

Imlirenla Pongener and Gavin J. Miller*

School of Chemical and Physical Sciences & Centre for Glycoscience, Keele University, Keele, Staffordshire, ST5 5BG, United Kingdom

*Email: g.j.miller@keele.ac.uk

Supporting Information 1

Experimental

Table of Contents

General Experimental S2

Synthesis of Glucuronic Acceptors S3

Synthesis of Glucuronic Glycal Acceptor S15

Synthesis of Glucosamine Donors S17

Glycosylations S28

Donor side products S40

Acceptor side products S41

Transformations of Disaccharide Building Block with GlcA Reducing End S42

References S56

General Experimental

The reagents and solvents used in the following experiments were bought commercially and used without further purification. Dry solvents were obtained using equipment based on Grubb's design[1] and stored under N_2 in Young's flask over 4 Å molecular sieves. Anhydrous DMF and Pyridine were purchased from Acros. For air-sensitive reactions, solvents were added *via* syringe through rubber septa. For reactions that required heating, DrySyn heating blocks were used as the heat source. Reactions were monitored by thin layer chromatography using Merck silica-coated 60F254 aluminium plates and the eluents are outlined in the respective experiments; spots were detected under 254 nm UV light and 10% H_2SO_4/EtOH staining followed by heat. Flash column chromatography was performed using silica gel [Davisil, 400–230 mesh (63–40 μm)]. ^{1}H NMR, ^{13}C{^{1}H} NMR and 2D NMR were carried out at 400 MHz on a Bruker AVIII400 spectrometer using deuterated chloroform ($CDCl_3$). Chemical shifts are reported in parts per million (ppm), coupling constants (*J*) are reported in Hertz (Hz) and multiplicities are abbreviated as; s (singlet), d (doublet), t (triplet) or m (multiplet) or combinations thereof. Chemical shifts were referenced to the residual proton of TMS for ^{1}H NMR spectra and to the ^{13}C chemical shift of deuterated chloroform ($CDCl_3$) for ^{13}C{^{1}H} NMR spectra. For compounds not reported in literature, NMR assignments have been made using COSY, HSQC and HMBC. HRMS were recorded on a ThermoScientific LTQ Orbitrap XL at the ESPRC National Mass Spectrometry Facility at Swansea University. Optical rotations were recorded on a Bellingham + Stanley ADP430 (specific rotation, tube length: 50 mm, concentrations in g per 100 mL).

1,2,3,4,6-Penta-*O*-acetyl-β-D-glucopyranose was purchased from Biosynth and *p*-tolyl 2-azido-3-*O*-benzyl-2-deoxy-1-thio-β-D-glucopyranoside **S11** was purchased from Heparin Building Blocks.

Synthesis of Glucuronic Acceptors

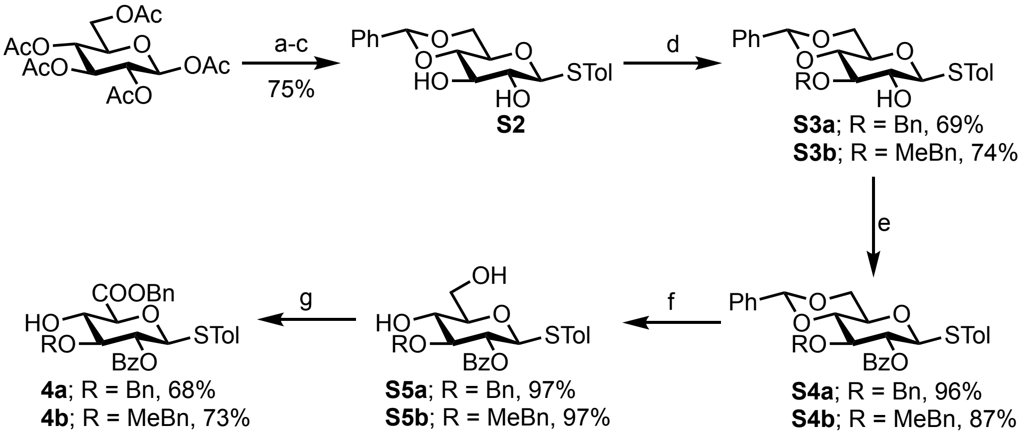

Scheme S1. Synthesis of STol GlcA acceptors 4a and 4b. Reagents: (a) TolSH, $BF_3.Et_2O$, CH_2Cl_2, 0 °C - rt (b) Na_2CO_3, MeOH (c) $PhCH(OMe)_2$, CSA, MeCN, 60 °C (d) (1) Bu_2SO, toluene, reflux (2) BnBr or MeBnBr, CsF, DMF, 118 °C (e) BzCl, pyridine, DMAP, CH_2Cl_2, rt (f) $TsOH.H_2O$, MeOH, reflux (g) (1) TEMPO, PIDA, CH_2Cl_2/H_2O (2:1), 0 °C - rt (2) BnBr, K_2CO_3, DMF, rt.

p-Tolyl 4,6-*O*-benzylidene-1-thio-β-D-glucopyranoside S2

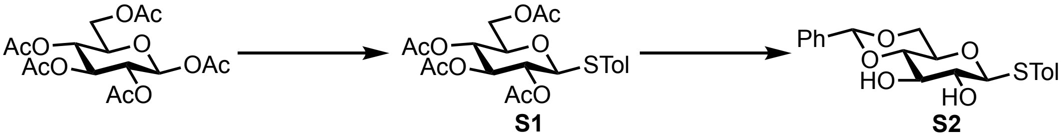

Under a N_2 atmosphere, a solution of pentaacetate glucose (20 g, 51 mmol) and toluenethiol (15.9 g, 128 mmol) in anhydrous CH_2Cl_2 (170 mL), was stirred at 0 °C for 30 min. $BF_3.Et_2O$ (19 mL, 0.16 mol) was then slowly added and the reaction was stirred at room temperature. TLC analysis after 4 h showed complete consumption of staring material (9:1; , R_f = 0.6). The reaction was quenched with saturated $NaHCO_3$ (400 mL). The product was extracted with CH_2Cl_2 (2 x 100 mL). The combined organic layers were washed with H_2O (200 mL), dried over anhydrous $MgSO_4$, filtered and concentrated *in vacuo* to give a white solid. The crude material **S1** was used in the next step without further purification. **^{1}H NMR** (400 MHz, $CDCl_3$): δ 7.39 (d, *J* = 8.2 Hz, 2H, Ph), 7.12 (d, *J* = 7.9 Hz, 2H, Ph), 5.21 (t, *J* = 9.4 Hz, 1H, H-3), 5.02 (t, *J* = 9.8 Hz, 1H, H-4), 4.93 (dd, *J* = 10.0, 9.2 Hz, 1H, H-2), 4.63 (d, *J* = 10.0 Hz, 1H, H-1), 4.22 (td, *J* = 12.3, 4.8 Hz, 1H, H-6a), 4.17 (dd, *J* = 12.3, 2.7 Hz, 1H, H-6b), 3.70 (ddd, *J* = 10.1, 4.8, 2.7 Hz, 1H, H-5), 2.35 (s, 3H, CH_3), 2.09 (s, 3H, CH_3), 2.08 (s, 3H, CH_3), 2.01 (s, 3H, CH_3), 1.99 (s, 3H, CH_3). NMR data were consistent with literature data.[2]

A solution of crude **S1** in bench MeOH (180 mL) was treated with Na_2CO_3 (1.6 g, 15 mmol) and stirred at room temperature for 13 h. The reaction mixture was neutralised with resin IR-120, filtered and concentrated *in vacuo* to give an off-white solid. The solid was washed with Et_2O to give a white solid. A solution of the solid in anhydrous MeCN (100 mL) was treated with CSA (2.37 g, 10.2 mmol), followed by benzaldehyde dimethyl acetal (12 mL, 77 mmol) and stirred at 60 °C for 18 h. The reaction mixture was concentrated *in vacuo*, re-dissolved in CH_2Cl_2 (500 mL), washed with saturated $NaHCO_3$ (500 mL), dried over anhydrous $MgSO_4$, filtered and concentrated *in vacuo* to give a pale yellow solid which was washed with Et_2O to give the title compound **S2** as a white solid (14.4 g, 75% yield over 3 steps). R_f = 0.23, 9:1; CH_2Cl_2/EtOAc. **^{1}H NMR** (400 MHz, $CDCl_3$): δ 7.50 – 7.40 (m, 4H, Ph), 7.36 (dd, *J* = 5.1, 2.0 Hz, 3H. Ph), 7.15 (d, *J* = 7.9 Hz, 2H, Ph), 5.52 (s, 1H, PhC*H*), 4.56 (d, *J* = 9.7 Hz, 1H, H-1), 4.37 (dd, *J* = 10.5, 4.5 Hz, 1H, H-6a), 3.82 (ddd, *J* = 8.8, 6.1, 2.1 Hz, 1H, H-3), 3.80 – 3.72 (m, 1H, H-6b), 3.53 – 3.45 (m, 2H, H-5, H-4), 3.42 (ddd, *J* = 9.7, 8.5, 2.3 Hz, 1H, H-2), 2.87 (d, *J* = 2.3 Hz, 1H, OH), 2.71 (d, *J* = 2.4 Hz, 1H, OH), 2.36 (s, 3H, CH_3). **^{13}C{^{1}H} NMR** (101 MHz, $CDCl_3$): δ 139.1 (C), 137.0 (C), 133.9 (CH), 130.1 (CH), 129.5 (CH), 128.5 (CH), 127.3 (C), 126.4 (CH), 102.1 (Ph*C*H), 88.9 (C-1), 80.4 (C-4), 74.7 (C-3), 72.6 (C-2), 70.7 (C-5), 68.7 (C-6), 21.3 (CH_3). NMR data were consistent with literature data.[3] **ESI-HRMS** for $C_{20}H_{21}O_5S$ $(M-H)^-$ calculated: 373.1115; found: 373.1116.

***p*-Tolyl 3-*O*-benzyl-4,6-*O*-benzylidene-1-thio-β-D-glucopyranoside S3a**

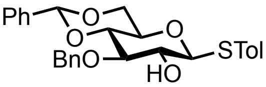

Under a N_2 atmosphere, a suspension of S**2** (4.82 g, 12.9 mmol) and Bu_2SnO (4.18 g, 16.8 mmol) in anhydrous toluene (130 mL) was heated to reflux using a Dean-Stark apparatus. The solution was stirred at reflux for 15 h. The reaction was then cooled down and concentrated *in vacuo* to give a pale yellow syrup. Under a N_2 atmosphere, the syrup was re-dissolved in anhydrous DMF, treated with CsF (5.88 g, 38.7 mmol) and BnBr (3.8 mL, 32 mmol) and was stirred at 118 °C. TLC analysis (CH_2Cl_2; R_f = 0.38) after 7 h showed complete consumption of starting material. The reaction mixture was cooled down to room temperature, filtered through a silica plug and washed with CH_2Cl_2. The filtrate was concentrated *in vacuo* to give a brownish slurry. After purification by column chromatography (CH_2Cl_2), the title compound **S3a** was obtained as a white solid (4.05 g, 69% yield). **^{1}H NMR** (400 MHz, $CDCl_3$): δ 7.53 – 7.29 (m,

12H, Ph), 7.13 (d, J = 7.9 Hz, 2H, Ph), 5.56 (s, 1H, PhCH), 4.94 (d, J = 11.5 Hz, 1H, CHHPh), 4.79 (d, J = 11.5 Hz, 1H, CHHPh), 4.56 (d, J = 9.7 Hz, 1H, H-1), 4.38 (dd, J = 10.5, 5.0 Hz, 1H, H-6a), 3.78 (t, J = 10.2 Hz, 1H, H-6b), 3.68 (dd, J = 9.2, 8.0 Hz, 1H, H-3/4), 3.63 (t, J = 9.0 Hz, 1H, H-3/4), 3.53 – 3.42 (m, 2H, H-2, H-5), 2.53 (d, J = 2.2 Hz, 1H, OH), 2.35 (s, 3H, CH_3). **$^{13}C\{^1H\}$ NMR** (101 MHz, $CDCl_3$): δ 138.9 (C), 138.3 (C), 137.4 (C), 134.0 (CH), 130.0 (CH), 129.2 (CH), 128.6 (CH), 128.4 (CH), 128.3 (CH), 128.0 (CH), 127.2 (C), 126.2 (CH), 101.4 (PhCH), 88.7 (C-1), 81.8 (C-3/4), 81.3 (C-3/4), 75.0 (PhCH_2), 72.3 (C-2/5) 70.9 (C-2/5), 68.8 (C-6), 21.3 (CH_3). NMR data were consistent with literature data.[3] **ESI-HRMS** for $C_{27}H_{29}O_5S$ $(M+H)^+$ calculated: 465.1730; found: 465.1731.

***p*-Tolyl 2-*O*-benzoyl-3-*O*-benzyl-4,6-*O*-benzylidene-1-thio-β-D-glucopyranoside S4a**

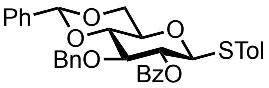

Under a N_2 atmosphere, a solution of **S3a** (4.05 g, 8.61 mmol) and DMAP (0.1 g, 0.8 mmol) in anhydrous CH_2Cl_2 (20 mL) was treated with anhydrous pyridine (1.7 mL, 22 mmol) followed by benzoyl chloride (2.6 mL, 22 mmol). The reaction was stirred at room temperature for 16 h and then quenched with H_2O (100 mL). The reaction mixture was diluted with CH_2Cl_2 (150 mL) and washed with 1 M HCl (75 mL) and saturated $NaHCO_3$ (150 mL). The organic layer was dried over anhydrous $MgSO_4$, filtered and concentrated *in vacuo* to give an off-white solid. The solid was washed with Et_2O/petroleum ether to give the title compound **S4a** as a white solid (4.76 g, 96% yield). R_f = 0.52, CH_2Cl_2. **^1H NMR** (400 MHz, $CDCl_3$): δ 8.06 – 7.96 (m, 2H, Ph), 7.60 (td, J = 7.3, 1.5 Hz, 1H, Ph), 7.52 – 7.31 (m, 9H, Ph), 7.16 – 6.99 (m, 7H, Ph), 5.60 (s, 1H, PhCH), 5.26 (dd, J = 10.1, 8.6 Hz, 1H, H-2), 4.80 (d, J = 11.9 Hz, 1H, CHHPh), 4.78 (d, J = 10.1 Hz, 1H, H-1), 4.65 (d, J = 11.9 Hz, 1H, CHHPh), 4.41 (dd, J = 10.5, 5.0 Hz, 1H, H-6a), 3.87 (t, J = 8.9 Hz, 1H, H-3), 3.84 (t, J = 10.3 Hz, 1H, H-6b), 3.79 (t, J = 9.3 Hz, 1H, H-4), 3.54 (td, J = 9.6, 5.0 Hz, 1H, H-5), 2.32 (s, 3H, CH_3). **$^{13}C\{^1H\}$ NMR** (101 MHz, $CDCl_3$): δ 165.2 (C=O), 138.7 (C), 137.8 (C), 137.3 (C), 133.8 (CH), 133.3 (CH), 130.7 (C), 130.1 (CH), 130.0 (C), 129.8 (CH), 129.2 (CH), 128.5 (CH), 128.4 (CH), 128.3 (CH), 128.2 (CH), 127.7 (CH), 126.2 (CH), 101.4 (PhCH), 87.3 (C-1), 81.6 (C-4), 79.5 (C-3), 74.4 (PhCH_2), 72.2 (C-2), 70.7 (C-5), 68.8 (C-6), 21.3 (CH_3). NMR data were consistent with literature data.[4] **ESI-HRMS** for $C_{34}H_{33}O_6S$ $(M+H)^+$ calculated: 569.1992; found: 569.1991.

***p*-Tolyl 2-*O*-benzoyl-3-*O*-benzyl-1-thio-β-D-glucopyranoside S5a**

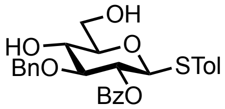

A solution of **S4a** (7.60 g, 13.4 mmol) in MeOH (130 mL) was treated with TsOH.H_2O (0.25 g, 1.3 mmol) and heated to reflux. TLC analysis (8:2; CH_2Cl_2/EtOAc; R_f = 0.18) after 2 h showed complete consumption of starting material. The reaction mixture was diluted with CH_2Cl_2 (200 mL) and washed with saturated $NaHCO_3$ (100 mL), dried over anhydrous $MgSO_4$, filtered and concentrated *in vacuo* to give an off-white solid. The solid was washed with Et_2O/petroleum ether to give the title compound **S5a** as a white solid (6.23 g, 97% yield). **^{1}H NMR** (400 MHz, $CDCl_3$): δ 8.14 – 8.04 (m, 2H, Ph), 7.66 – 7.57 (m, 1H, Ph), 7.48 (t, *J* = 7.7 Hz, 2H, Ph), 7.37 – 7.28 (m, 2H, Ph), 7.23 – 7.14 (m, 5H, Ph), 7.08 (d, *J* = 7.9 Hz, 2H, Ph), 5.26 – 5.18 (m, 1H, H-2), 4.77 (d, *J* = 10.0 Hz, 1H, H-1), 4.71 (d, *J* = 11.4 Hz, 1H, C*H*HPh), 4.58 (d, *J* = 11.4 Hz, 1H, C*H*HPh), 3.92 (ddd, *J* = 12.0, 6.2, 3.4 Hz, 1H, H-6a), 3.80 (ddd, *J* = 12.0, 7.1, 5.1 Hz, 1H, H-6b), 3.75 – 3.63 (m, 2H, H-3, H-4), 3.45 (ddd, *J* = 8.7, 5.0, 3.2 Hz, 1H, H-5), 2.54 (d, *J* = 2.3 Hz, 1H, OH), 2.31 (s, 3H, CH_3), 2.12 (t, *J* = 6.7 Hz, 1H, OH). **^{13}C{^{1}H} NMR** (101 MHz, $CDCl_3$): δ 165.3 (C=O), 138.5 (C), 137.8 (C), 133.5 (CH), 133.3 (CH), 130.0 (CH), 129.89 (C), 129.86 (CH), 128.8 (C), 128.68 (CH), 128.65 (CH), 128.2 (CH), 86.8 (C-1), 84.1 (C-3), 79.5 (C-5), 74.9 (Ph*C*H_2), 72.5 (C-2), 70.5 (C-4), 62.8 (C-6), 21.3 (CH_3). NMR data consistent with literature data.[5] **ESI-HRMS** for $C_{27}H_{27}O_6S$ $(M-H)^-$ calculated: 479.1534; found: 479.1537.

Benzyl (*p*-tolyl 2-*O*-benzoyl-3-*O*-benzyl-1-thio-β-D-glucopyranosyluronate) 4a

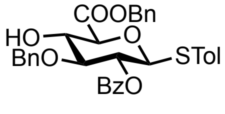

A solution of diol **S5a** (1.0 g, 2.1 mmol) in 2:1 CH_2Cl_2/H_2O (10 mL) was treated with PIDA (1.67 g, 5.20 mmol) and TEMPO (65 mg, 0.42 mmol) at 0 °C. The reaction mixture was then stirred at room temperature for 30 min. The reaction was quenched with saturated $Na_2S_2O_3$ (80 mL) and diluted with EtOAc (100 mL). The layers were separated and the aqueous layer was then acidified with conc HCl (pH adjusted to 3) and extracted with EtOAc (2 x 100 mL). The combined organic layers were dried over anhydrous $MgSO_4$, filtered and concentrated *in vacuo* to give a yellow slurry. Under a N_2 atmosphere, the slurry was re-dissolved in anhydrous DMF

(5 mL) and treated with K_2CO_3 (575 mg, 4.16 mmol) and BnBr (0.50 mL, 4.2 mmol). The suspension was stirred at room temperature for 3.5 h. TLC analysis (97:3; CH_2Cl_2:Et_2O; R_f = 0.5) at this point showed complete consumption of starting material. The reaction was quenched with H_2O (100 mL) and the product was extracted with CH_2Cl_2 (3 x 75 mL). The combined organic layers were dried over anhydrous $MgSO_4$, filtered and concentrated *in vacuo* to give an orangish-brown syrup. After purification by column chromatography (100:0 to 97:3; CH_2Cl_2:Et_2O) an off-white solid was obtained which, when washed with Et_2O/petroleum ether gave **4a** as a white solid (824 mg, 68% over 2 steps). $[\alpha]_D^{21}$ = +7.8 (c 0.5, $CHCl_3$). **^{1}H NMR** (400 MHz, $CDCl_3$): δ 8.07 – 7.99 (m, 2H, Ph), 7.60 (t, *J* = 7.4 Hz, 1H, Ph), 7.52 – 7.30 (m, 9H, Ph), 7.18 – 7.08 (m, 5H, Ph), 6.96 (d, *J* = 7.8 Hz, 2H, Ph), 5.27 (s, 2H, 2 x C*H*HPh), 5.21 (t, *J* = 9.6 Hz, 1H, H-2), 4.81 – 4.62 (m, 3H, 2 x C*H*HPh, H-1), 4.03 (td, *J* = 9.2, 2.7 Hz, 1H, H-4), 3.93 (d, *J* = 9.8 Hz, 1H, H-5), 3.71 (t, *J* = 8.9 Hz, 1H, H-3), 3.02 (d, *J* = 2.7 Hz, 1H, OH), 2.29 (s, 3H). **^{13}C{^{1}H} NMR** (101 MHz, $CDCl_3$): δ 168.9 (C=O), 165.2 (C=O), 138.7 (C), 137.8 (C), 135.1 (C), 134.0 (CH), 133.4 (CH), 130.0 (CH), 129.9 (C), 129.7 (CH), 128.8 (CH), 128.7 (CH), 128.6 (CH), 128.4 (CH), 128.2 (CH), 127.9 (CH), 87.2 (C-1), 82.4 (C-3), 77.8 (C-5), 74.9 (Ph*C*H_2), 72.1 (C-4), 71.6 (C-2), 67.7 (Ph*C*H_2), 21.3 (CH_3). **ESI-HRMS** for $C_{34}H_{36}O_7SN$ $(M+NH_4)^+$ calculated: 602.2207; found: 602.2207.

***p*-Tolyl 4,6-*O*-benzylidene-3-*O*-(*p*-methylbenzyl)-1-thio-β-D-glucopyranoside S3b**

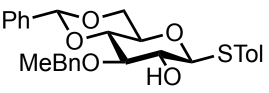

Under a N_2 atmosphere, a suspension of **S2** (2.00 g, 5.34 mmol) and Bu_2SnO (1.73 g, 6.94 mmol) in anhydrous toluene (20 mL) was heated to reflux using a Dean-Stark apparatus. The solution was stirred at reflux for 15 h. The reaction was then cooled down and concentrated *in vacuo* to give a pale yellow syrup. Under a N_2 atmosphere, the syrup was re-dissolved in anhydrous DMF, treated with CsF (1.63 g, 10.7 mmol) and *p*-MeBnBr (1.98 g, 10.7 mmol) and was stirred at 118 °C. TLC analysis (CH_2Cl_2; R_f = 0.34) after 5 h showed complete consumption of starting material. The reaction mixture was cooled down to room temperature, filtered through a silica plug and washed with CH_2Cl_2. The filtrate was concentrated *in vacuo* to give a brownish slurry. After purification by column chromatography (CH_2Cl_2), the title compound **S3b** was obtained as a white solid (1.89 g, 74% yield). $[\alpha]_D^{21}$ = -46.8 (c 0.5, $CHCl_3$). **^{1}H NMR** (400 MHz, $CDCl_3$): δ 7.53 – 7.33 (m,7H, Ph), 7.24 (dd, *J* = 6.9, 1.6 Hz, 2H, Ph), 7.12 (d, *J* =

7.8 Hz, 4H, Ph), 5.55 (s, 1H, PhC*H*), 4.90 (d, *J* = 11.3 Hz, 1H, C*H*HPh), 4.73 (d, *J* = 11.3 Hz, 1H, C*H*HPh), 4.55 (d, *J* = 9.7 Hz, 1H, H-1), 4.37 (dd, *J* = 10.5, 5.0 Hz, 1H, H-6a), 3.77 (t, *J* = 10.2 Hz, 1H, H-6b), 3.70 – 3.57 (m, 2H, H-3, H-4), 3.52 – 3.41 (m, 2H, H-5, H-2), 2.51 (d, *J* = 2.2 Hz, 1H, OH), 2.34 (s, 3H, CH_3), 2.32 (s, 3H, CH_3). **^{13}C{^{1}H} NMR** (101 MHz, $CDCl_3$): δ 138.9 (C), 137.8 (C), 137.4 (C), 135.3 (C), 134.0 (CH), 129.9 (CH), 129.3 (CH), 129.1 (CH), 128.42 (CH), 128.39 (CH), 127.4 (C), 126.1 (CH), 101.4 (Ph*C*H), 88.6 (C-1), 81.5 (C-3), 81.3 (C-4), 74.8 (Ph*C*H_2), 72.2 (C-2), 70.9 (C-5), 68.8 (C-6), 21.33 (CH_3), 21.31 (CH_3). **ESI-HRMS** for $C_{28}H_{34}O_5SN$ $(M+NH_4)^+$ calculated: 496.2152; found: 496.2155.

***p*-Tolyl 2-*O*-benzoyl-4,6-*O*-benzylidene-3-*O*-(*p*-methylbenzyl)-1-thio-β-D-glucopyranoside S4b**

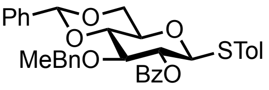

Under a N_2 atmosphere, a solution of **S3b** (1.89 g, 3.95 mmol) and DMAP (49 mg, 0.400 mmol) in anhydrous CH_2Cl_2 (13 mL) was treated with anhydrous pyridine (0.75 mL, 10.0 mmol) followed by benzoyl chloride (1.2 mL, 10.0 mmol). The reaction was stirred at room temperature for 15 h and then quenched with H_2O (75 mL). The reaction mixture was diluted with CH_2Cl_2 (150 mL) and washed with 1 M HCl (50 mL) and saturated $NaHCO_3$ (100 mL). The organic layer was dried over anhydrous $MgSO_4$, filtered and concentrated *in vacuo* to give an off-white solid. The solid was washed with petroleum ether to give the title compound **S4b** as a white solid (2.01 g, 87% yield). R_f = 0.38, CH_2Cl_2. $[\alpha]_D^{21}$ = +39.7 (c 0.5, $CHCl_3$). **^{1}H NMR** (400 MHz, $CDCl_3$): δ 8.00 (dd, *J* = 8.2, 1.4 Hz, 2H, Ph), 7.64 – 7.58 (m, 1H, Ph), 7.51 – 7.44 (m, 4H, Ph), 7.42 – 7.35 (m, 3H, Ph), 7.35 – 7.30 (m, 2H, Ph), 7.08 (d, *J* = 7.8 Hz, 2H, Ph), 6.99 (d, *J* = 7.9 Hz, 2H, Ph), 6.84 (d, *J* = 7.8 Hz, 2H, Ph), 5.59 (s, 1H, PhC*H*), 5.23 (dd, *J* = 10.0, 8.6 Hz, 1H, H-2), 4.76 (d, *J* = 10.0 Hz, 1H, H-1), 4.75 (d, *J* = 11.8 Hz, 1H, C*H*HPh), 4.60 (d, *J* = 11.8 Hz, 1H, C*H*HPh), 4.41 (dd, *J* = 10.5, 5.0 Hz, 1H, H-6a), 3.86 (t, *J* = 9.0 Hz, 1H, H-3), 3.87 – 3.74 (m, 2H, H-6b, H-4), 3.53 (td, *J* = 9.6, 5.0 Hz, 1H, H-5), 2.32 (s, 3H, CH_3), 2.20 (s, 3H, CH_3). **^{13}C{^{1}H} NMR** (101 MHz, $CDCl_3$): δ 165.1 (C=O), 138.6 (C), 137.35 (C), 137.34 (C), 134.8 (C), 133.8 (CH), 133.3 (CH), 130.1 (CH), 130.0 (C), 129.8 (CH), 129.2 (CH), 129.0 (CH), 128.5 (CH), 128.41 (CH), 128.39 (CH), 126.2 (CH), 101.4 (Ph*C*H), 87.3 (C-1), 81.6 (C-4), 79.2 (C-3), 74.2 (Ph*C*H_2), 72.2 (C-2), 70.7 (C-5), 68.8 (C-6), 21.3 (CH_3), 21.2 (CH_3). **ESI-HRMS** for $C_{35}H_{38}O_6SN$ $(M+NH_4)^+$ calculated: 600.2414; found: 600.2410.

p-Tolyl 2-O-benzoyl-3-O-(p-methylbenzyl)-1-thio-β-D-glucopyranoside S5b

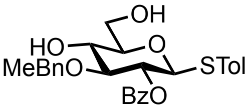

A solution of **S4b** (2.01 g, 3.45 mmol) in 9:1 MeOH/CH_2Cl_2 (130 mL) was treated with TsOH.H_2O (0.13 g, 0.68 mmol) and heated to reflux. TLC analysis (8:2; CH_2Cl_2/EtOAc; R_f = 0.2) after 4.5 h showed complete consumption of starting material. The reaction mixture was quenched with saturated $NaHCO_3$ (200 mL) and extracted with and CH_2Cl_2 (3 x 150 mL). The combined organic layers were dried over anhydrous $MgSO_4$, filtered and concentrated *in vacuo* to give an off-white solid. The solid was washed with Et_2O/petroleum ether to give the title compound **S5b** as a white solid (1.66 g, 97% yield). $[\alpha]_D^{21}$ = +9.4 (c 0.5, $CHCl_3$). **^{1}H NMR** (400 MHz, $CDCl_3$): δ 8.10 – 8.04 (m, 2H, Ph), 7.65 – 7.58 (m, 1H, Ph), 7.53 – 7.44 (m, 2H, Ph), 7.36 – 7.28 (m, 2H, Ph), 7.12 – 7.05 (m, 4H, Ph), 7.02 (d, *J* = 7.9 Hz, 2H, Ph), 5.25 – 5.16 (m, 1H, H-2), 4.77 (d, *J* = 10.0 Hz, 1H, H-1), 4.68 (d, *J* = 11.4 Hz, 1H, C*H*HPh), 4.51 (d, *J* = 11.4 Hz, 1H, C*H*HPh), 3.97 – 3.87 (m, 1H, H-6a), 3.84 – 3.74 (m, 1H, H-6b), 3.72 – 3.62 (m, 2H, H-3, H-4), 3.50 – 3.40 (m, 1H, H-5), 2.37 (d, *J* = 2.1 Hz, 1H, OH), 2.31 (s, 3H, CH_3), 2.26 (s, 3H, CH_3), 2.03 (t, *J* = 6.6 Hz, 1H, OH). **^{13}C{^{1}H} NMR** (101 MHz, $CDCl_3$): δ 165.3 (C=O), 138.5 (C), 138.0 (C), 134.8 (C), 133.5 (CH), 133.3 (CH), 130.0 (CH), 129.91 (C), 129.86 (CH), 129.4 (CH), 128.8 (C), 128.6 (CH), 128.3 (CH), 86.8 (C-1), 83.9 (C-3), 79.5 (C-5), 74.8 (Ph*C*H_2), 72.6 (C-2), 70.5 (C-4), 62.9 (C-6), 21.3 (2 x CH_3). **ESI-HRMS** for $C_{28}H_{29}O_6S$ $(M-H)^-$ calculated: 493.1690; found: 493.1692.

Benzyl (p-tolyl 2-O-benzoyl-3-O-(p-methylbenzyl)-1-thio-β-D-glucopyranosyluronate) 4b

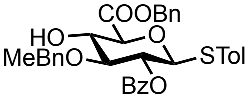

A suspension of the diol **5b** (800 mg, 1.62 mmol) in 2:1:1 CH_2Cl_2/MeCN/H_2O (40 mL) was treated with PIDA (1.30 g, 4.05 mmol) and TEMPO (51 mg, 0.32 mmol) at room temperature. The reaction mixture was stirred for 1.5 h during which the suspension became homogenous. The reaction was quenched with saturated $Na_2S_2O_3$ (60 mL) and diluted with EtOAc (80 mL). The layers were separated and the aqueous layer was then acidified with conc HCl (pH adjusted to 3) and extracted with EtOAc (2 x 80 mL). The combined organic layers were dried over anhydrous $MgSO_4$, filtered and concentrated *in vacuo* to give a yellow slurry. Under a N_2

atmosphere, the slurry was re-dissolved in anhydrous DMF (4 mL) and treated with K_2CO_3 (448 mg, 3.24 mmol) and BnBr (0.38 mL, 3.2 mmol). The suspension was stirred at room temperature for 2 h. TLC analysis (97:3; CH_2Cl_2:Et_2O; R_f = 0.4) at this point showed complete consumption of starting material. The reaction was quenched with H_2O (80 mL) and the product was extracted with CH_2Cl_2 (3 x 100 mL). The combined organic layers were dried over anhydrous $MgSO_4$, filtered and concentrated *in vacuo* to give an orangish-brown syrup. After purification by column chromatography (100:0 to 97:3; CH_2Cl_2:Et_2O) an off-white solid was obtained which, when washed with Et_2O/petroleum ether gave **4b** as a white solid (704 mg, 73% over 2 steps). $[\alpha]_D^{21}$ = +10.6 (c 0.5, $CHCl_3$). **^{1}H NMR** (400 MHz, $CDCl_3$): δ 8.05 – 7.98 (m, 2H, Ph), 7.64 – 7.56 (m, 1H, Ph), 7.51 – 7.30 (m, 9H, Ph), 7.03 (d, *J* = 7.9 Hz, 2H, Ph), 6.99 – 6.94 (m, 2H, Ph), 6.92 (d, *J* = 7.8 Hz, 2H, Ph), 5.27 (s, 2H, 2 x C*H*HPh), 5.18 (dd, *J* = 10.0, 9.1 Hz, 1H,H-2), 4.71 (d, *J* = 10.0 Hz, 1H, H-1), 4.68 (d, *J* = 11.5 Hz, 1H, C*H*HPh), 4.64 (d, *J* = 11.5 Hz, 1H, C*H*HPh), 4.01 (ddd, *J* = 9.8, 8.7, 2.6 Hz, 1H, H-4), 3.92 (d, *J* = 9.8 Hz, 1H, H-5), 3.69 (t, *J* = 8.9 Hz, 1H, H-3), 2.96 (d, *J* = 2.6 Hz, 1H, OH), 2.29 (s, 3H, CH_3), 2.22 (s, 3H, CH_3). **^{13}C{^{1}H} NMR** (101 MHz, $CDCl_3$) δ 168.8 (C=O), 165.2 (C=O), 138.6 (C), 137.6 (C), 135.1 (C), 134.8 (C), 134.0 (CH), 133.3 (CH), 130.0 (CH), 129.9 (C), 129.7 (CH), 129.2 (CH), 128.8 (CH), 128.7 (CH), 128.5 (CH), 128.43 (CH), 128.36 (CH), 128.2 (C), 87.2 (C-1), 82.2 (C-3), 77.9 (C-5), 74.8 (Ph*C*H_2), 72.1 (C-4), 71.6 (C-2), 67.7 (Ph*C*H_2), 21.29 (CH_3), 21.25 (CH_3). **ESI-HRMS** for $C_{35}H_{38}O_7SN$ $(M+NH_4)^+$ calculated: 616.2363; found: 616.2362.

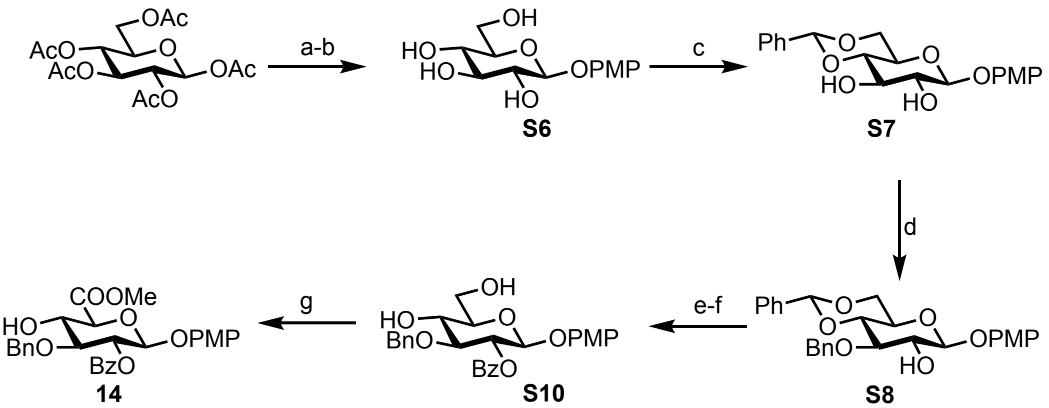

Scheme S2. Synthesis of *p*-methoxyphenol GlcA acceptor 35. Reagents: (a) *p*-methoxyphenol, $BF_3.Et_2O$, CH_2Cl_2, 0 °C – rt (b) Na_2CO_3, MeOH, quantitative over 2 steps (c) $PhCH(OMe)_2$, CSA, MeCN/DMF (3.5:1), 60 °C, 71% (d) (1) Bu_2SnO, toluene, reflux (2) BnBr, CsF, DMF, 80 °C, 56% (e) BzCl, pyridine, DMAP, CH_2Cl_2, rt (f) $TsOH.H_2O$, MeOH, reflux, 96% over 2 steps (g) (1) TEMPO, PIDA, CH_2Cl_2/H_2O (2:1), 0 °C – rt (2) MeI, K_2CO_3, DMF, 72% over 2 steps.

***p*-Methoxyphenyl β-D-glucopyranoside S6**

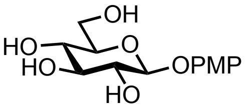

Under a N_2 atmosphere, a solution of pentaacetate glucose (20 g, 51 mmol) and *p*-methoxyphenol (7.29 g, 58.7 mmol) in anhydrous CH_2Cl_2 (150 mL), was stirred at 0 °C for 30 min. $BF_3.Et_2O$ (16 mL, 0.13 mol) was then slowly added and the reaction was stirred at room temperature. TLC analysis after 3 h showed complete consumption of staring material (9:1; , R_f = 0.5). The reaction was quenched with saturated $NaHCO_3$ (400 mL). The product was extracted with CH_2Cl_2 (2 x 100 mL). The combined organic layers were washed with H_2O (200 mL), dried over anhydrous $MgSO_4$, filtered and concentrated *in vacuo* to give a white solid. **^{1}H NMR** (400 MHz, $CDCl_3$): δ 6.98 – 6.91 (m, 2H, Ph), 6.85 – 6.79 (m, 2H, Ph), 5.31 – 5.12 (m, 3H, H-2, H-3, H-4), 4.95 (d, *J* = 7.6, 1H, H-1), 4.29 (dd, *J* = 12.3, 5.2 Hz, 1H, H-6a), 4.17 (dd, *J* = 12.3, 2.5 Hz, 1H), H-6b, 3.83 – 3.78 (m, 1H, H-5), 3.78 (s, 3H, OCH_3), 2.085 (s, 3H, CH_3), 2.077 (s, 3H, CH_3), 2.04 (s, 3H, CH_3), 2.03 (s, 3H, CH_3). NMR data were consistent with literature data.[6] A solution of the white solid in bench MeOH (180 mL) was treated with Na_2CO_3 (1.6 g, 15 mmol) and stirred at room temperature for 16 h. The reaction mixture was neutralised with resin IR-120, filtered and concentrated *in vacuo* to give a pinkish-white solid. The solid was washed with Et_2O to give a white solid (quantitative). The crude material **S6** was used in the next step without further purification.

***p*-Methoxyphenyl 4,6-*O*-benzylidene-β-D-glucopyranoside S7**

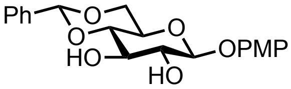

Under a N_2 atmosphere, a solution of tetraol **S6** (12 g, 42 mmol) in anhydrous MeCN and DMF (3.5:1 = 105 mL) was treated with *p*-TsOH.H_2O (0.80 g, 4.2 mmol), followed by benzaldehyde dimethyl acetal (32 mL, 0.21 mol) and stirred at 60 °C for 2.5 h. The reaction mixture was quenched with Et_3N (0.60 mL, 4.2 mmol) and concentrated *in vacuo* to give an off-white paste which was washed with MeOH to give the title compound **S7** as a white solid (11.2 g, 71% yield). R_f = 0.62, EtOAc. **^{1}H NMR** (400 MHz, DMSO): δ 7.47 – 7.42 (m, 2H, Ph), 7.41 – 7.34 (m, 3H, Ph), 7.02 – 6.96 (m, 2H, Ph), 6.89 – 6.82 (m, 2H, Ph), 5.60 (s, 1H, PhC*H*), 5.61 – 5.56 (m, 1H, 2-OH), 5.43 (d, *J* = 5.1 Hz, 1H, 3-OH), 4.97 (d, *J* = 7.7 Hz, 1H, H-1), 4.20 (dd, *J* = 10.0, 4.8 Hz, 1H, H-6a), 3.75 – 3.67 (m, 1H, H-6b), 3.71 (s, 3H, CH_3), 3.63 – 3.50 (m, 2H, H-

5, H-3), 3.46 (t, J = 9.2 Hz, 1H, H-4), 3.38 – 3.30 (m, 1H, H-2). **^{13}C{^{1}H} NMR** (101 MHz, DMSO): δ 154.5 (C), 151.0 (C), 137.7 (C), 128.8 (CH), 128.0 (CH), 126.3 (CH), 117.8 (CH), 114.5 (CH), 101.7 (C-1), 100.7 (Ph*C*H), 80.4 (C-4), 74.2 (C-2), 72.8 (C-3), 67.9 (C-6), 65.7 (C-5), 55.4 (OCH_3). NMR data were consistent with literature data.[7] **ESI-HRMS** for $C_{20}H_{21}O_7$ $(M-H)^-$ calculated: 373.1293; found: 373.1294.

***p*-Methoxyphenyl 3-*O*-benzyl-4,6-*O*-benzylidene-β-D-glucopyranoside S8**

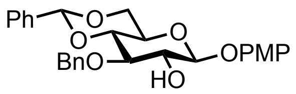

Under a N_2 atmosphere, a suspension of diol **S7** (10 g, 27 mmol) and Bu_2SnO (8.74 g, 35.1 mmol) in anhydrous toluene (200 mL) was heated to reflux using a Dean-Stark apparatus. The solution was stirred at reflux for 15 h. The reaction was then cooled down and concentrated *in vacuo* to give a brownish syrup. Under a N_2 atmosphere, the syrup was re-dissolved in anhydrous DMF (40 mL), treated with CsF (8.2 g, 54 mmol) and BnBr (6.4 mL, 54 mmol) and was stirred at 80 °C. TLC analysis (CH_2Cl_2; R_f = 0.3) after 4 h showed complete consumption of starting material. The reaction mixture was cooled down to room temperature, filtered through a silica plug and washed with CH_2Cl_2. The filtrate was concentrated *in vacuo* to give a yellow solid. The yellow solid was washed successively with MeOH and then Et_2O/CH_2Cl_2 (1:1) to give the title compound **S8** as a white solid (7.05 g, 56% yield). **^{1}H NMR** (400 MHz, $CDCl_3$): δ 7.53 – 7.46 (m, 2H, Ph), 7.42 – 7.27 (m, 8H, Ph), 7.04 – 6.97 (m, 2H, Ph), 6.86 – 6.76 (m, 2H, Ph), 5.59 (s, 1H, PhC*H*), 4.99 (d, J = 11.7 Hz, 1H, C*H*HPh), 4.90 (d, J = 7.5 Hz, 1H, H-1), 4.82 (d, J = 11.7 Hz, 1H, C*H*HPh), 4.37 (dd, J = 10.5, 5.0 Hz, 1H, H-6a), 3.87 – 3.69 (m, 7H, H-6b, H-2, H-3, H-4, OCH_3), 3.53 (ddd, J = 10.0, 8.7, 4.9 Hz, 1H, H-5), 2.57 (d, J = 2.5 Hz, 1H, OH). **^{13}C{^{1}H} NMR** (101 MHz, $CDCl_3$): δ 155.8 (C), 151.1 (C), 138.4 (C), 137.3 (C), 129.2 (CH), 128.6 (CH), 128.4 (CH), 128.2 (CH), 128.0 (CH), 126.2 (CH), 118.9 (CH), 114.7 (CH), 102.7 (C-1), 101.5 (Ph*C*H), 81.3 (C-3/4), 80.4 (C-3/4), 74.9 (Ph*C*H_2), 74.2 (C-2), 68.8 (C-6), 66.7 (C-5), 55.8 (OCH_3). NMR data were consistent with literature data.[8] **ESI-HRMS** for $C_{27}H_{28}O_7Na$ $(M+Na)^+$ calculated: 487.1727; found: 487.1727.

***p*-Methoxyphenyl 2-*O*-benzoyl-3-*O*-benzyl-β-D-glucopyranoside S10**

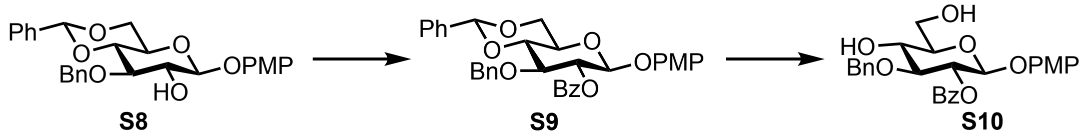

Under a N_2 atmosphere, a solution of **S8** (7.05 g, 15.2 mmol) and DMAP (0.1 g, 0.8 mmol) in anhydrous CH_2Cl_2 and anhydrous pyridine (2:1 = 76 mL) followed by benzoyl chloride (5.4 mL, 46 mmol). The reaction was stirred at room temperature for 13 h and then quenched with MeOH. The reaction mixture was concentrated *in vacuo* to give an off-white solid. The solid was re-dissolved in CH_2Cl_2 (150 mL) and washed with H_2O (300 mL). The organic layer was dried over anhydrous $MgSO_4$, filtered and concentrated *in vacuo* to give **S9** as a white solid. The solid was washed with Et_2O/petroleum ether (1:2) to give a white solid. R_f = 0.5, CH_2Cl_2. **^{1}H NMR** (400 MHz, $CDCl_3$): δ 8.01 (dd, *J* = 8.3, 1.4 Hz, 2H, Ph), 7.63 – 7.56 (m, 1H, Ph), 7.56 – 7.49 (m, 2H, Ph), 7.49 – 7.34 (m, 5H, Ph), 7.20 – 7.04 (m, 5H, Ph), 6.92 – 6.83 (m, 2H, Ph), 6.80 – 6.68 (m, 2H, Ph), 5.64 (s, 1H, PhC*H*), 5.53 (dd, *J* = 8.8, 7.8 Hz, 1H, H-2), 5.07 (d, *J* = 7.9 Hz, 1H, H-1), 4.85 (d, *J* = 12.0 Hz, 1H, C*H*HPh), 4.72 (d, *J* = 12.1 Hz, 1H, C*H*HPh), 4.42 (dd, *J* = 10.5, 5.0 Hz, 1H, H-6a), 3.99 – 3.83 (m, 3H, H-3, H-4, H-6b), 3.72 (s, 3H, OCH_3), 3.60 (td, *J* = 9.4, 4.9 Hz, 1H, H-5). **^{13}C{^{1}H} NMR** (101 MHz, $CDCl_3$): δ 165.2 (C=O), 155.8 (C), 151.3 (C), 137.9 (C), 137.3 (C), 133.3 (CH), 130.0 (CH), 129.9 (C), 129.2 (CH), 128.54 (CH), 128.45 (CH), 128.3 (CH), 128.2 (CH), 127.7 (CH), 126.2 (CH), 119.0 (CH), 114.7 (CH), 101.6 (C-1), 101.5 (Ph*C*H), 81.6 (C-3/4), 78.1 (C-3/4), 74.2 (Ph*C*H_2), 73.5 (C-2), 68.9 (C-6), 66.6 (C-5), 55.7 (OCH_3). NMR data were consistent with literature data.[9] **ESI-HRMS** for $C_{34}H_{36}O_8N$ $(M+NH_4)^+$ calculated: 586.2435; found: 586.2438.

A solution of the white solid **S9** in MeOH/CH_2Cl_2 (4:1 = 100) was treated with TsOH.H_2O (0.867 g, 4.56 mmol) and heated to reflux. TLC analysis (8:2; ; R_f = 0.2) after 2 h showed complete consumption of starting material. The reaction mixture was diluted with CH_2Cl_2 (150 mL) and washed with saturated $NaHCO_3$ (150 mL), dried over anhydrous $MgSO_4$, filtered and concentrated *in vacuo* to give a white solid. The solid was washed with Et_2O/petroleum ether (1:1) to give **S10** as a white solid (7.0 g, 96% over 2 steps). **^{1}H NMR** (400 MHz, $CDCl_3$): δ 8.09 – 8.00 (m, 2H, Ph), 7.63 – 7.54 (m, 1H, Ph), 7.48 – 7.41 (m, 2H, Ph), 7.21 (s, 5H, Ph), 6.91 – 6.82 (m, 2H,Ph), 6.77 – 6.69 (m, 2H, Ph), 5.48 (dd, *J* = 9.4, 7.9 Hz, 1H, H-2), 5.04 (d, *J* = 7.9 Hz, 1H,H-1), 4.77 (d, *J* = 11.5 Hz, 1H, C*H*HPh, C*H*HPh), 4.65 (d, *J* = 11.5 Hz, 1H), 3.96 (ddd, *J* = 11.9, 5.8, 3.4 Hz, 1H, H-6a), 3.90 – 3.79 (m, 2H, H-6a, H-4), 3.76 (t, *J* = 9.2 Hz, 1H,

H-3), 3.72 (s, 3H, OCH_3), 3.54 (ddd, *J* = 9.5, 5.0, 3.4 Hz, 1H, H-5), 2.75 (d, *J* = 3.3 Hz, 1H, OH), 2.26 (t, *J* = 6.6 Hz, 1H, OH). **^{13}C{^{1}H} NMR** (101 MHz, $CDCl_3$): δ 165.4 (C=O), 155.7 (C), 151.3 (C), 137.9 (C), 133.4 (CH), 129.9 (CH), 129.8 (C), 128.7 (CH), 128.6 (CH), 128.2 (CH), 128.1 (CH), 118.6 (CH), 114.7 (CH), 100.9 (C-1), 82.5 (C-3), 75.7 (C-5), 74.7 (Ph*C*H_2), 73.6 (C-2), 70.5 (C-4), 62.6 (C-6), 55.8 (OCH_3). NMR data consistent with literature data.[10] **ESI-HRMS** for $C_{27}H_{27}O_8$ $(M-H)^-$ calculated: 479.1711; found: 479.1717.

Methyl (*p*-methoxyphenyl 2-*O*-benzoyl-3-*O*-benzyl-β-D-glucopyranosyluronate) 14

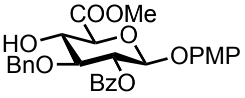

A solution of the diol **S10** (7.00 g, 15 mmol) in 2:1 CH_2Cl_2/H_2O (73 mL) was treated with PIDA (11.8 g, 36.5 mmol) and TEMPO (456 mg, 2.92 mmol) at 0 °C. The reaction mixture was then stirred at room temperature for 30 min. The reaction mixture went from clear orange to brown by this time. The reaction was quenched after 1 h with saturated $Na_2S_2O_3$ (500 mL) and diluted with EtOAc (300 mL). The layers were seperated and the aqueous layer was then acidified with conc HCl (pH adjusted to 3) and extracted with EtOAc (5 x 100 mL). The combined organic layers were dried over anhydrous $MgSO_4$, filtered and concentrated *in vacuo* to give a brown paste. **^{1}H NMR** (400 MHz, $CDCl_3$) selected signals: δ 6.94 – 6.83 (m, 2H, Ph), 6.82 – 6.63 (m, 2H, Ph), 5.48 (dd, *J* = 9.3, 7.8 Hz, 1H, H-2), 5.06 (d, *J* = 7.8 Hz, 1H, H-1), 4.84 (d, *J* = 11.7 Hz, 1H, C*H*HPh), 4.76 (d, *J* = 11.7 Hz, 1H, C*H*HPh), 4.13 (dd, *J* = 9.7, 8.5 Hz, 1H, H-4), 4.05 (d, *J* = 9.7 Hz, 1H, H-5), 3.84 – 3.77 (m, 1H, H-3), 3.71 (s, 3H, OCH_3).

Under a N_2 atmosphere, the paste was re-dissolved in anhydrous DMF (30 mL) and treated with K_2CO_3 (6.01 g, 43.8 mmol) and MeI (2.70 mL, 43.8 mmol). The suspension was stirred at room temperature for 2 h. TLC analysis (97:3; CH_2Cl_2:Et_2O ; R_f = 0.5) at this point showed complete consumption of starting material. The reaction was quenched with H_2O (500 mL) and the product was extracted with Et_2O (4 x 100 mL). The combined organic layers were dried over anhydrous $MgSO_4$, filtered and concentrated *in vacuo* to give an orangish-brown syrup. After purification by column chromatography (100:0 to 90:10; CH_2Cl_2:Et_2O) an off-white solid was obtained which, when washed with Et_2O/petroleum ether (2:1) gave **14** as a white solid (5.63 g, 72% over 2 steps).

^{1}H NMR (400 MHz, $CDCl_3$): δ 8.05 – 7.96 (m, 2H, Ph), 7.66 – 7.54 (m, 1H, Ph), 7.51 – 7.38 (m, 2H, Ph), 7.22 – 7.04 (m, 5H, Ph), 6.98 – 6.87 (m, 2H, Ph, Ph), 6.83 – 6.65 (m, 2H), 5.48

(dd, $J = 9.3$, 7.7 Hz, 1H, H-2), 5.02 (d, $J = 7.6$ Hz, 1H, H-1), 4.81 (d, $J = 11.7$ Hz, 1H, C*H*HPh), 4.76 (d, $J = 11.7$ Hz, 1H, C*H*HPh), 4.17 (ddd, $J = 9.7$, 8.8, 2.7 Hz, 1H, H-4), 4.01 (d, $J = 9.7$ Hz, 1H, H-5), 3.83 (s, 3H, OCH_3), 3.78 (t, $J = 9.1$ Hz, 1H, H-3), 3.73 (s, 3H, OCH_3), 3.11 (d, $J = 2.7$ Hz, 1H, OH). **$^{13}C\{^1H\}$ NMR** (101 MHz, $CDCl_3$): δ 169.6 (C=O), 165.2 (C=O), 155.9 (C), 151.4 (C), 137.9 (C), 133.4 (CH), 130.0 (CH), 129.8 (C), 128.6 (CH), 128.5 (CH), 128.2 (CH), 127.9 (CH), 119.0 (CH), 114.6 (CH), 101.4 (C-1), 80.8 (C-3), 74.6 (Ph*C*H_2), 74.4 (C-5), 73.0 (C-2), 72.0 (C-4), 55.8 (OCH_3), 53.1 (OCH_3). NMR data consistent with literature data.[10] **ESI-HRMS** for $C_{28}H_{28}O_9Na$ $(M+Na)^+$ calculated: 531.1626; found: 531.1619.

Synthesis of Glucuronic Glycal Acceptor

4,6-*O*-[Bis(tert-butyl)silylene]-1,2-dideoxy-D-arabino-1-hexenopyranose 26

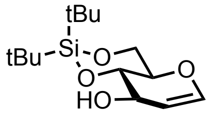

Under a N_2 atmosphere, a solution of D-glucal (1.50 g, 10.3 mmol) in anhydrous DMF (21 mL) was treated with anhydrous pyridine (1.7 mL, 21 mmol). The solution was cooled to -40 °C and then slowly treated with (3.7 mL, 11 mmol). The reaction was slowly warmed to room temperature over 1.5 h. TLC analysis (CH_2Cl_2; $R_f = 0.35$) at this point showed complete consumption of starting material. The reaction mixture was diluted with Et_2O (150 mL) and washed with H_2O (300 mL). The layers were separated and the aqueous layer was extracted with Et_2O (2 x 150 mL). The combined organic layers were dried over anhydrous MgSO4, filtered and concentrated *in vacuo* to give a pale yellow syrup. Purification by column chromatography (100:0 to 95:5;) gave **26** as an off-white solid (2.16 g, 73%). **^1H NMR** (400 MHz, $CDCl_3$): δ 6.27 (dd, $J = 6.1$, 1.9 Hz, 1H, H-1), 4.76 (dd, $J = 6.1$, 1.9 Hz, 1H, H-2), 4.30 (ddt, $J = 7.2$, 3.6, 1.9 Hz, 1H, H-3), 4.18 (dd, $J = 10.2$, 4.8 Hz, 1H, H-6a), 3.97 (t, $J = 10.2$ Hz, 1H, H-6b), 3.92 (dd, $J = 10.1$, 7.2 Hz, 1H, H-4), 3.84 (td, $J = 10.2$, 4.9 Hz, 1H, H-5), 2.36 (d, $J = 3.4$ Hz, 1H, OH), 1.07 (s, 9H, $SiC(CH_3)_3$), 1.00 (s, 9H, $SiC(CH_3)_3$). **$^{13}C\{^1H\}$ NMR** (101 MHz, $CDCl_3$): δ 143.8 (C-1), 103.1 (C-2), 77.5 (C-4), 72.4 (C-5), 70.4 (C-3), 65.9 (C-6), 27.6 (SiC(*C*H_3)$_3$), 27.1 (SiC(*C*H_3)$_3$), 22.9 (Si*C*(CH_3)$_3$), 20.0 (Si*C*(CH_3)$_3$). NMR data were consistent with literature data.[11] **ESI-HRMS** for $C_{14}H_{26}O_4SiNa$ $(M+Na)^+$ calculated: 309.1493; found: 309.1494.

3-*O*-Benzyl-1,2-dideoxy-D-arabino-1-hexenopyranose 28

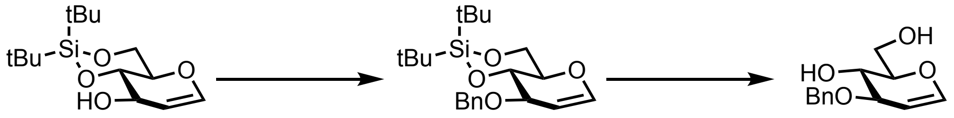

26 27 28

Under a N_2 atmosphere, a solution of glucopyranoside **26** (4.00 g, 14.0 mmol) in anhydrous DMF (35 mL) was cooled to 0 °C and NaH (60% dispersion in mineral oil) (1.1 g, 28 mmol) was added. After 15 minutes, benzyl bromide (2.5 mL, 21 mmol) was added dropwise and the reaction mixture was left to stir at room temperature for 14 h. The reaction was quenched with MeOH, diluted with CH_2Cl_2 (75 mL) and washed with H_2O (175 mL). The aqueous layer was washed with CH_2Cl_2 (3 x 75 mL). The organic layers were combined dried over anhydrous $MgSO_4$, filtered and concentrated *in vacuo* to give a yellow syrup. Crude **27** was used in the next step without further purification.

The crude syrup was dissolved in a 1 M THF solution of TBAF (28 mL, 28 mmol) and stirred at room temperature for 2.5 h. The reaction was then diluted with CH_2Cl_2 (75 mL) and washed with H_2O (150 mL). The aqueous layer was washed with CH_2Cl_2 (4 x 50 mL). The organic layers were combined, dried over anhydrous $MgSO_4$, filtered and concentrated *in vacuo* to give a white solid. Purification by column chromatography (50:50 to 5:95; CH_2Cl_2/Et_2O) gave **28** as a white solid (2.04 g, 62% over 2 steps). R_f = 0.3, 50:50; CH_2Cl_2/Et_2O. **^{1}H NMR** (400 MHz, $CDCl_3$): δ 7.39 – 7.27 (m, 5H, Ph), 6.37 (dd, *J* = 6.1, 1.6 Hz, 1H, H-1), 4.86 (dd, *J* = 6.2, 2.3 Hz, 1H, H-2), 4.71 (d, *J* = 11.6 Hz, 1H, C*H*HPh), 4.57 (d, *J* = 11.6 Hz, 1H, C*H*HPh), 4.13 – 4.07 (m, 1H, H-3), 3.99 – 3.91 (m, 1H, H-4), 3.94 – 3.83 (m, 3H, H-6a, H-6b, H-5), 2.70 (d, *J* = 3.1 Hz, 1H, OH), 2.23 (app s, 1H, OH). **^{13}C{^{1}H} NMR** (101 MHz, $CDCl_3$): δ 144.8 (C-1), 138.3 (C), 128.7 (CH), 128.1 (CH), 128.0 (CH), 100.2 (C-2), 77.9 (C-5), 76.4 (C-3), 70.9 (Ph*C*H_2), 68.6 (C-4), 62.1 (C-6). NMR data were consistent with literature data.[12] **ESI-HRMS** for $C_{13}H_{15}O_4$ $(M-H)^-$ calculated: 235.0976; found: 235.0976.

Methyl 3-*O*-benzyl-D-glucuronal 29

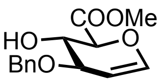

A solution of the diol **53** (2.03 g, 8.59 mmol) in 2:1 CH_2Cl_2/H_2O (43 mL) was treated with PIDA (6.93 g, 21.5 mmol) and TEMPO (269 mg, 1.72 mmol) at 0 °C. The reaction mixture

was then stirred at room temperature for 2 h. The reaction was quenched with saturated $Na_2S_2O_3$ (200 mL) and diluted with EtOAc (100 mL). The layers were separated and the aqueous layer was then acidified with conc HCl (pH adjusted to 3) and extracted with EtOAc (5 x 75 mL). The combined organic layers were dried over anhydrous $MgSO_4$, filtered and concentrated *in vacuo* to give a brown syrup. Under a N_2 atmosphere, the syrup was re-dissolved in anhydrous DMF (17 mL) and treated with K_2CO_3 (5.9 g, 43 mmol) and MeI (1.6 mL, 26 mmol). The suspension was stirred at room temperature for 15 h. The reaction was quenched with H_2O (200 mL) and the product was extracted with CH_2Cl_2 (4 x 100 mL). The combined organic layers were dried over anhydrous $MgSO_4$, filtered and concentrated *in vacuo* to give an orangish-brown syrup. Purification by column chromatography (10:90 to 70:30; Et_2O/petroleum ether) gave **29** as a yellow syrup (1.0 g, 44% over 2 steps). $R_f = 0.29$, 70:30; Et_2O/petroleum ether. $[\alpha]_D^{21} = +51.2$ (c 0.5, $CHCl_3$). **^{1}H NMR** (400 MHz, $CDCl_3$): δ 7.36 – 7.27 (m, 5H, Ph), 6.61 (dd, J = 6.2, 0.7 Hz, 1H, H-1), 4.99 (ddd, J = 6.1, 4.7, 1.3 Hz, 1H, H-2), 4.63 (dd, J = 4.3, 1.1 Hz, 1H, H-5), 4.58 (d, J = 11.5 Hz, 1H, C*H*HPh), 4.53 (d, J = 11.5 Hz, 1H, C*H*HPh), 4.39 (dddd, J = 8.0, 4.4, 3.6, 1.3 Hz, 1H, H-4), 3.85 (ddt, J = 4.6, 3.6, 0.9 Hz, 1H, H-3), 3.61 (s, 3H, OCH_3), 2.22 (d, J = 8.2 Hz, 1H, OH). **^{13}C{^{1}H} NMR** (101 MHz, $CDCl_3$): δ 168.6 (C=O), 145.1 (C-1), 138.0 (C), 128.5 (CH), 128.0 (CH), 127.9 (CH), 99.1 (C-2), 75.4 (C-5), 70.4 (C-3), 70.2 (Ph*C*H_2), 67.1 (C-4), 52.4 (OCH_3). **ESI-HRMS** for $C_{14}H_{16}O_5Na$ $(M+Na)^+$ calculated: 287.0890; found: 287.0882.

Synthesis of Glucosamine Donors

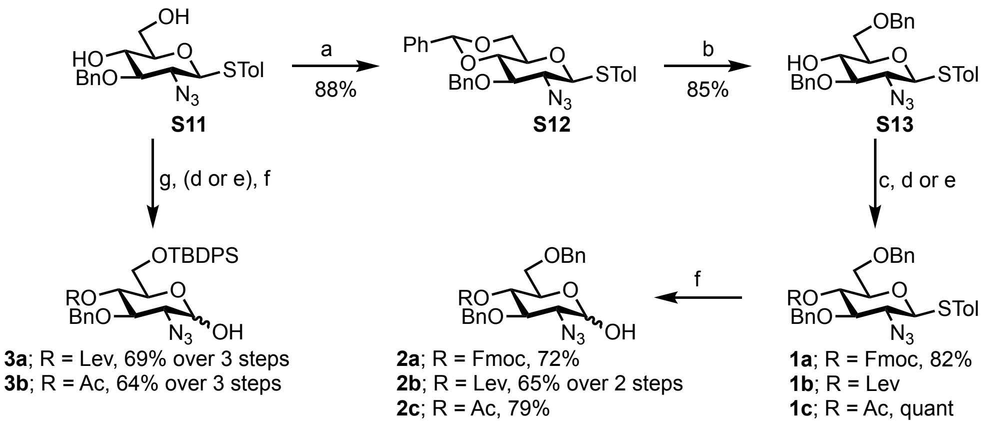

Scheme S3. Synthesis of glucosamine donors. Reagents: (a) $PhCH(OMe)_2$, CSA, MeCN, 60 °C (b) TFA, Et_3SiH, CH_2Cl_2, 0 °C (c) FmocCl, pyridine, DMAP, CH_2Cl_2, rt (d) LevOH, 2-chloro-1-methylpyridinium iodide or DCC, DIPEA, DMAP, CH_2Cl_2, rt (e) Ac_2O, pyridine, DMAP, CH_2Cl_2, rt (f) NBS, acetone/H_2O, rt (g) TBDPSCl, imidazole, DMAP, DMF, rt.

***p*-Tolyl 2-azido-3-*O*-benzyl-4,6-*O*-benzylidene-2-deoxy-1-thio-β-D-glucopyranoside S12**

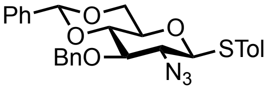

Under a N_2 atmosphere, a solution of *p*-tolyl 2-azido-3-*O*-benzyl-2-deoxy-1-thio-β-D-glucopyranoside **S11** (2.00 g, 4.98 mmol) in anhydrous MeCN (10 mL) was treated with CSA (231 mg, 0.996 mmol), followed by benzaldehyde dimethyl acetal (1.1 mL, 7.5 mmol) and stirred at 60 °C for 5 h. The reaction mixture was concentrated *in vacuo*, re-dissolved in CH_2Cl_2 (150 mL), washed with saturated NaHCO3 (90 mL), dried over anhydrous MgSO4, filtered and concentrated *in vacuo* to give an off-white solid. The solid was washed with Et_2O/petroleum ether to give the title compound **S12** as a white solid (2.15 g, 88% yield). R_f = 0.7, CH_2Cl_2. **^{1}H NMR** (400 MHz, $CDCl_3$): δ 7.56 – 7.27 (m, 12H, Ph), 7.15 (d, *J* = 7.8 Hz, 2H, Ph), 5.56 (s, 1H, PhC*H*), 4.90 (d, *J* = 10.9 Hz, 1H, C*H*HPh), 4.77 (d, *J* = 10.9 Hz, 1H, C*H*HPh), 4.42 (d, *J* = 10.2 Hz, 1H, H-1), 4.40 – 4.35 (m, 1H, H, H-6a), 3.77 (t, *J* = 10.3 Hz, 1H, H-6b), 3.68 – 3.55 (m, 2H, H-3, H-4), 3.43 (td, *J* = 9.3, 5.2 Hz, 1H, H-5), 3.32 (dd, *J* = 10.2, 8.7 Hz, 1H, H-2), 2.35 (s, 3H, CH_3). **^{13}C{^{1}H} NMR** (101 MHz, $CDCl_3$): δ 139.3 (C), 137.7 (C), 137.2 (C), 134.7 (CH), 130.1 (CH), 129.2 (CH), 128.6 (CH), 128.49 (CH), 128.45 (CH), 128.2 (CH), 126.6 (C), 126.1 (CH), 101.4 (Ph*C*H), 86.7 (C-1), 81.4 (C-4), 81.1 (C-3), 75.4 (Ph*C*H_2), 70.6 (C-5), 68.6 (C-6), 64.6 (C-2), 21.3 (CH_3). NMR data were consistent with literature data.[13] **ESI-HRMS** for $C_{27}H_{28}O_4N_3S$ $(M+H)^+$ calculated: 490.1795; found: 490.1798.

***p*-Tolyl 2-azido-3,6-di-*O*-benzyl-2-deoxy-1-thio-β-D-glucopyranoside S13**

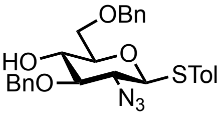

Based on the literature procedure,[14] under a N_2 atmosphere, a solution of **S12** (2.14 g, 4.37 mmol) in anhydrous CH_2Cl_2 (15 mL) was cooled to 0 °C and treated slowly with triethylsilane (4.1 mL, 26 mmol) followed by trifluoroacetic acid (2.0 mL, 26 mmol). The reaction mixture was stirred at 0 °C for 1 h. The reaction was quenched with saturated $NaCHO_3$ (150 mL) and diluted with CH_2Cl_2 (200 mL). The organic layer was washed with brine (100 mL), dried over anhydrous $MgSO_4$, filtered and concentrated *in vacuo* to give a yellow syrup. Purification by column chromatography (CH_2Cl_2; R_f = 0.3,) gave **S13** as a clear syrup (1.83 g, 85% yield). **^{1}H NMR** (400 MHz, $CDCl_3$): δ 7.46 (dd, *J* = 8.3, 2.0 Hz, 2H, Ph), 7.42 – 7.27 (m, 10H, Ph), 7.08

(dd, J = 8.3, 2.0 Hz, 2H, Ph), 4.89 (d, J = 11.0 Hz, 1H, C*H*HPh), 4.81 (d, J = 11.0 Hz, 1H, C*H*HPh), 4.60 (d, J = 11.9 Hz, 1H, C*H*HPh), 4.55 (d, J = 11.9 Hz, 1H, C*H*HPh), 4.37 (d, J = 9.9 Hz, 1H, H-1), 3.78 (dd, J = 10.4, 4.8 Hz, 1H, H-6a), 3.73 (dd, J = 10.4, 4.4 Hz, 1H, H-6b), 3.60 (td, J = 9.1, 2.6 Hz, 1H, H-4), 3.43 (dt, J = 9.4, 4.7 Hz, 1H, H-5), 3.36 (t, J = 9.0 Hz, 1H, H-3), 3.27 (t, J = 9.6 Hz, 1H, H-2), 2.68 (d, J = 2.5 Hz, 1H, OH), 2.32 (s, 3H, CH_3). **^{13}C{^{1}H} NMR** (101 MHz, $CDCl_3$): δ 138.9 (C), 138.0 (C), 137.8 (C), 134.3 (CH), 129.9 (CH), 128.8 (CH), 128.6 (CH), 128.4 (CH), 128.3 (CH), 128.0 (CH), 127.9 (CH), 127.2 (C), 86.3 (C-1), 84.8 (C-3), 78.1 (C-5), 75.6 (Ph*C*H_2), 73.9 (Ph*C*H_2), 72.2 (C-4), 70.5 (C-6), 64.5 (C-2), 21.3 (CH_3). NMR data were consistent with literature data.[13] **ESI-HRMS** for $C_{27}H_{29}O_4N_3SNa$ $(M+Na)^+$ calculated: 514.1771; found: 514.1761.

***p*-Tolyl 2-azido-3,6-di-*O*-benzyl-2-deoxy-4-*O*-fluorenylmethoxycarbonyl-1-thio-β-D-glucopyranoside 1a**

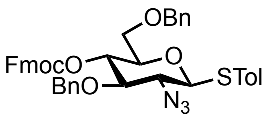

Under a N_2 atmosphere, a solution of **S13** (1.83 g, 3.72 mmol), DMAP (0.1 g, 0.8 mmol) and FmocCl (1.92 g, 7.44 mmol) in anhydrous CH_2Cl_2 (12 mL) was treated with anhydrous pyridine (0.6 mL, 8 mmol). The reaction was stirred at room temperature for 5 h and then diluted with Et_2O (100 mL) and washed with H_2O (100 mL). The organic layer was dried over anhydrous $MgSO_4$, filtered and concentrated *in vacuo* to give a yellow syrup. Purification by column chromatography (1:2 to 3:1; CH_2Cl_2/petroleum ether) gave **1a** as a white solid (2.18 g, 82% yield). R_f = 0.7, CH_2Cl_2. $[\alpha]_D^{21}$ = -92.8 (c 0.5, $CHCl_3$) **^{1}H NMR** (400 MHz, $CDCl_3$): δ 7.75 (dq, J = 7.5, 1.0 Hz, 2H, Ph), 7.56 (dq, J = 7.5, 0.9 Hz, 1H, Ph), 7.51 (dq, J = 7.5, 0.9 Hz, 1H, Ph), 7.49 – 7.43 (m, 2H, Ph), 7.38 (tdt, J = 7.5, 1.8, 0.9 Hz, 2H, Ph), 7.34 – 7.17 (m, 12H, Ph), 7.09 – 7.02 (m, 2H, Ph), 4.82 – 4.75 (m, 1H, H-4), 4.76 (d, J = 10.9 Hz, 1H, C*H*HPh), 4.65 (d, J = 10.9 Hz, 1H, C*H*HPh), 4.54 (d, J = 11.9 Hz, 1H, C*H*HPh), 4.52 (d, J = 11.9 Hz, 1H, C*H*HPh), 4.37 (d, J = 10.1 Hz, 1H, H-1), 4.34 (dd, J = 10.5, 7.1 Hz, 1H, Fmoc-CH_2), 4.28 (dd, J = 10.4, 7.2 Hz, 1H, Fmoc-CH_2), 4.11 (t, J = 7.1 Hz, 1H, Fmoc-CH), 3.67 – 3.59 (m, 3H, H-6a, H-6b, H-5), 3.54 (t, J = 9.3 Hz, 1H, H-3), 3.33 (dd, J = 10.1, 9.3 Hz, 1H, H-2), 2.32 (s, 3H, CH_3). **^{13}C{^{1}H} NMR** (101 MHz, $CDCl_3$): δ 154.4 (C=O), 143.4 (C), 143.2 (C), 141.44 (C), 141.40 (C), 139.1 (C), 138.1 (C), 137.3 (C), 134.4 (CH), 130.0 (CH), 128.52 (CH), 128.47 (CH), 128.2 (CH), 128.11 (CH), 128.08 (CH), 128.07 (CH), 127.8 (CH), 127.7 (CH), 127.32 (CH), 127.31

(CH), 126.8 (C), 125.2 (CH), 125.1 (CH), 120.24 (CH), 120.22 (CH), 86.1 (C-1), 82.5 (C-3), 77.4 (C-5), 75.7 (Ph*C*H$_2$), 75.2 (C-4), 73.7 (Ph*C*H$_2$), 70.3 (Fmoc-*C*H$_2$), 69.5 (C-6), 64.6 (C-2), 46.8 (Fmoc-*C*H), 21.3 (CH$_3$). **ESI-HRMS** for $C_{42}H_{43}O_6N_4S$ $(M+NH_4)^+$ calculated: 731.2898; found: 731.2908.

2-Azido-3,6-di-*O*-benzyl-2-deoxy-4-*O*-fluorenylmethoxycarbonyl-α/β-D-glucopyranose 2a

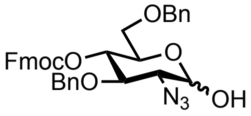

A solution of **1a** (892 mg, 1.25 mmol) in 9:1 acetone/ H_2O (13 mL) and treated with NBS (667 mg, 3.75 mmol) at room temperature. After 2 h the reaction was quenched with saturated $Na_2S_2O_3$ (80 mL) and diluted with CH_2Cl_2 (80 mL). The organic layer was washed with H_2O (60 mL), dried over anhydrous $MgSO_4$, filtered and concentrated *in vacuo* to give a yellow paste. Purification by column chromatography (100:0 to 95:5, CH_2Cl_2/Et_2O) afforded the hydrolysed product **2a** as a white solid (559 mg, 72% yield, α/β = 59:41). R_f = 0.34, 95:5; CH_2Cl_2/Et_2O.

The following were observed for α and β anomers: **^{1}H NMR** (400 MHz, CDCl$_3$): δ 7.76 (d, *J* = 7.7 Hz, 2H, Ph), 7.54 (dt, *J* = 11.0, 7.2 Hz, 2H, Ph), 7.39 (t, *J* = 7.5 Hz, 2H, Ph), 7.33 – 7.14 (m, 12H, Ph), 4.50 (s, 2H, 2 x C*H*HPh), 4.40 – 4.24 (m, 2H, Fmoc-CH$_2$), 4.15 – 4.07 (m, 1H, Fmoc-CH), 3.59 – 3.48 (m, 2H, H-6a, H-6b). **^{13}C{^{1}H} NMR** (101 MHz, CDCl$_3$): δ 141.44 (C), 141.41 (C), 137.6 (C), 137.53 (C), 137.51 (C), 128.54 (CH), 128.50 (CH), 128.49 (CH), 128.09 (CH), 128.07 (CH), 128.04 (CH), 128.02 (CH), 127.99 (CH), 127.97 (CH), 127.94 (CH), 127.34 (CH), 127.32 (CH), 125.24 (CH), 125.18 (CH), 125.12 (CH), 125.09 (CH), 120.24 (CH), 120.22 (CH), 70.3 (Fmoc-*C*H$_2$), 46.8 (Fmoc-*C*H).

α-anomer

^{1}H NMR (400 MHz, CDCl$_3$): δ 5.30 (t, *J* = 3.5 Hz, 1H, H-1), 4.87 (t, *J* = 9.7 Hz, 1H, H-4), 4.80 (d, *J* = 11.0 Hz, 1H, C*H*HPh), 4.68 (d, *J* = 11.0 Hz, 1H, C*H*HPh), 4.27 – 4.17 (m, 1H, H-5), 4.09 – 4.02 (m, 1H, H-3), 3.59 (d, *J* = 3.3 Hz, 1H, OH), 3.49 – 3.41 (m, 1H, H-2). **^{13}C{^{1}H} NMR** (101 MHz, CDCl$_3$): δ 154.5 (C=O), 143.4 (C), 143.25 (C), 92.0 (C-1), 77.7 (C-3), 75.8 (C-4), 75.31 (Ph*C*H$_2$), 73.77 (Ph*C*H$_2$), 69.1 (C-6), 68.9 (C-5), 63.5 (C-2).

β-anomer

^{1}H NMR (400 MHz, $CDCl_3$): δ 4.82 (t, *J* = 9.3 Hz, 1H, H-4), 4.78 (d, *J* = 11.2 Hz, 1H, C*H*HPh), 4.64 (d, *J* = 11.2 Hz, 1H, C*H*HPh), 4.55 (dd, *J* = 7.4, 5.2 Hz, 1H, H-1), 4.03 (d, *J* = 5.5 Hz, 1H, OH), 3.67 – 3.58 (m, 1H, H-5), 3.44 – 3.35 (m, 2H, H-3, H-2). **$^{13}C\{^1H\}$ NMR** (101 MHz, $CDCl_3$) δ 154.4 (C=O), 143.30 (C), 143.2 (C), 96.3 (C-1), 80.3 (C-3), 75.28 (C-4 or Ph*C*H_2), 75.2 (C-4 or Ph*C*H_2), 73.82 (Ph*C*H_2), 73.2 (C-5), 69.2 (C-6), 66.9 (C-2). NMR data were consistent with literature data.[15]

ESI-HRMS for $C_{35}H_{33}O_7N_3Na$ $(M+Na)^+$ calculated: 630.2211; found: 630.2211.

2-Azido-3,6-di-*O*-benzyl-2-deoxy-4-*O*-levulinoyl-α/β-D-glucopyranose 2b

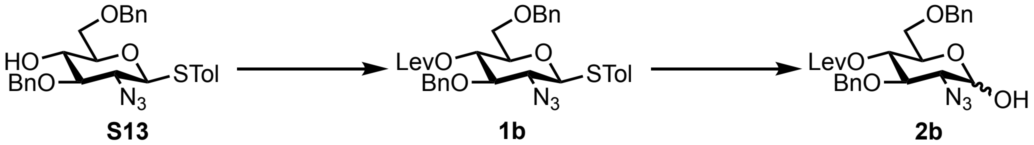

Under a N_2 atmosphere, a solution of **S13** (0.34 g, 0.700 mmol), DMAP (10 mg, 0.08 mmol), levulinic acid (0.16 g, 1.4 mmol) and 2-chloro-1-methylpyridinium iodide (0.36 g, 1.4 mmol) in anhydrous CH_2Cl_2 (1.4 mL) was treated with anhydrous DIPEA (0.24 mL, 1.4 mmol). The reaction was stirred at room temperature for 5 h and then diluted with Et_2O (50 mL). The reaction mixture was washed with H_2O (20 mL), 1 M HCl (20 mL) and saturated $NaHCO_3$ (30 mL). The organic layer was dried over anhydrous $MgSO_4$, filtered and concentrated *in vacuo* to give a yellow syrup. The crude material was used in the next step without further purification. R_f = 0.36, 2:1; petroleum ether/Et_2O. **^{1}H NMR** (400 MHz, $CDCl_3$): δ 7.51 – 7.45 (m, 2H, Ph), 7.40 – 7.20 (m, 10H, Ph), 7.05 (d, *J* = 7.7 Hz, 2H, Ph), 4.93 (dd, *J* = 9.3, 8.0 Hz, 1H, H-4), 4.78 (d, *J* = 11.3 Hz, 1H, C*H*HPh), 4.66 (d, *J* = 11.3 Hz, 1H, C*H*HPh), 4.52 (s, 2H, 2 x C*H*HPh), 4.38 (d, *J* = 10.1 Hz, 1H, H-1), 3.64 – 3.53 (m, 3H, H-6a, H-6b, H-5), 3.50 (t, *J* = 9.3 Hz, 1H, H-3), 3.33 (dd, *J* = 10.1, 9.3 Hz, 1H, H-2), 2.58 (dd, *J* = 9.3, 6.4 Hz, 2H, Lev-CH_2), 2.46 – 2.34 (m, 2H, Lev-CH_2), 2.32 (s, 3H, CH_3), 2.12 (s, 3H, CH_3).

The crude material **1b** was dissolved in 9:1 acetone/ H_2O (7 mL) and treated with NBS (374 mg, 2.10 mmol) at room temperature. After 5 h the reaction was quenched with saturated $Na_2S_2O_3$ (30 mL) and diluted with CH_2Cl_2 (50 mL). The organic layer was washed with H_2O (20 mL), dried over anhydrous $MgSO_4$, filtered and concentrated *in vacuo* to give a yellow syrup. Purification by column chromatography (100:0 to 90:10, CH_2Cl_2/Et_2O) afforded the

hydrolysed product **2b** as a syrup (221 mg, 65% yield over 2 steps, α/β = 67:33) along with **1b** (80 mg, 19% recovered). R_f = 0.19, 95:5; CH_2Cl_2/Et_2O.

The following were observed for α and β anomers: **^{1}H NMR** (400 MHz, $CDCl_3$): δ 7.39 – 7.25 (m, 10H, Ph), 3.56 – 3.47 (m, 2H, H-6a, H-6b), 2.71 – 2.60 (m, 2H, Lev-CH_2), 2.60 – 2.49 (m, 2H, Lev-CH_2), 2.48 – 2.34 (m, 2H, Lev-CH_2), 2.33 – 2.23 (m, 2H, Lev-CH_2), 2.13 (s, 3H, Lev-CH_3). **^{13}C{^{1}H} NMR** (101 MHz, $CDCl_3$): δ 137.9 (C), 128.56 (CH), 128.55 (CH), 128.51 (CH), 128.48 (CH), 128.3 (CH), 128.2 (CH), 128.09 (CH), 128.07 (CH), 127.99 (CH), 127.97 (CH), 127.93 (CH), 127.88 (CH).

α-anomer

^{1}H NMR (400 MHz, $CDCl_3$): δ 7.39 – 7.25 (m, 10H, Ph), 5.30 (t, *J* = 3.5 Hz, 1H, H-1), 5.03 (dd, *J* = 10.3, 9.2 Hz, 1H, H-4), 4.81 (d, *J* = 11.2 Hz, 1H, C*H*HPh), 4.66 (d, *J* = 11.2 Hz, 1H, C*H*HPh), 4.52 (s, 2H, 2 x C*H*HPh), 4.14 (ddd, *J* = 10.3, 5.5, 3.3 Hz, 1H, H-5), 4.00 (dd, *J* = 10.2, 9.1 Hz, 1H, H-3), 3.65 (d, *J* = 2.7 Hz, 1H, OH), 3.44 (ddd, *J* = 10.1, 3.5, 1.4 Hz, 1H, H-2), 2.13 (s, 3H, CH_3). **^{13}C{^{1}H} NMR** (101 MHz, $CDCl_3$) δ 206.34 (C=O), 171.73 (C=O), 137.8 (C), 92.0 (C-1), 77.7 (C-3), 74.97 (Ph*C*H_2), 73.7 (Ph*C*H_2), 71.4 (C-4), 69.2 (C-5), 69.1 (C-6), 63.7 (C-2), 37.9 (Lev-*C*H_2), 29.89 (Lev-*C*H_3), 28.0 (Lev-*C*H_2).

β-anomer

^{1}H NMR (400 MHz, $CDCl_3$): δ 4.96 (ddd, *J* = 9.4, 6.4, 2.8 Hz, 1H, H-4), 4.80 (d, *J* = 11.5 Hz, 1H, C*H*HPh), 4.63 (d, *J* = 11.5 Hz, 1H, C*H*HPh), 4.56 (td, *J* = 5.5, 2.1 Hz, 1H, H-1), 4.52 (s, 1H, 2 x C*H*HPh), 4.07 (d, *J* = 5.4 Hz, 1H, OH), 3.59 – 3.54 (m, 1H, H-5), 3.41 – 3.36 (m, 2H, H-2, H-3), 2.13 (s, 3H, CH_3). **^{13}C{^{1}H} NMR** (101 MHz, $CDCl_3$) δ 206.30 (C=O), 171.74 (C=O), 137.7 (C), 96.3 (C-1), 80.4 (C-3), 75.01 (Ph*C*H_2), 73.8 (Ph*C*H_2), 73.6 (C-5), 70.9 (C-4), 69.3 (C-6), 67.1 (C-2), 37.8 (Lev-*C*H_2), 29.87 (Lev-*C*H_3), 27.9 (Lev-*C*H_2).

ESI-HRMS for $C_{25}H_{29}O_7N_3Na$ $(M+Na)^+$ calculated: 506.1898; found: 506.1884.

***p*-Tolyl 4-*O*-acetyl-2-azido-3,6-di-*O*-benzyl-2-deoxy-1-thio-β-D-glucopyranoside 1c**

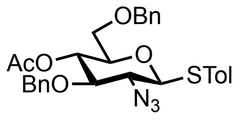

A solution of **S13** (0.34 g, 0.700 mmol) and DMAP (10 mg, 0.08 mmol) in anhydrous CH_2Cl_2 (1.4 mL) was treated with pyridine (0.11 mL, 1.4 mmol) followed by acetic anhydride (0.13 mL, 1.4 mmol) and was stirred at room temperature for 2 h. The reaction mixture was diluted with CH_2Cl_2 (50 mL) and washed with H_2O (20 mL), 1 M HCl (20 mL) and saturated $NaHCO_3$

(30 mL). The organic layer was dried over anhydrous $MgSO_4$, filtered and concentrated *in vacuo* to give a white solid. The crude material **1c** was used in the next step without further purification. R_f = 0.5, CH_2Cl_2. **^{1}H NMR** (400 MHz, $CDCl_3$): δ 7.46 (d, J = 7.9 Hz, 2H, Ph), 7.39 – 7.21 (m, 10H, Ph), 7.06 (d, J = 7.9 Hz, 2H, Ph), 4.95 – 4.86 (m, 1H, H-4), 4.79 (d, J = 11.2 Hz, 1H, C*H*HPh), 4.62 (d, J = 11.2 Hz, 1H, C*H*HPh), 4.51 (s, 2H, 2 x C*H*HPh), 4.37 (d, J = 10.1 Hz, 1H, H-1), 3.59 – 3.51 (m, 3H, H-6a, H-6b, H-5), 3.48 (t, J = 9.3 Hz, 1H, H-3), 3.37 – 3.28 (m, 1H, H-2), 2.32 (s, 3H, CH_3), 1.85 (s, 3H, CH_3). **^{13}C{^{1}H} NMR** (101 MHz, $CDCl_3$): δ 169.8 (C=O), 139.1 (C), 138.0 (C), 137.6 (C), 134.4 (CH), 130.0 (CH), 128.7 (CH), 128.5 (CH), 128.17 (CH), 128.16 (CH), 127.9 (CH), 127.8 (CH), 126.8 (C), 86.0 (C-1), 82.7 (C-3), 77.8 (C-5), 75.5 (Ph*C*H_2), 73.7 (Ph*C*H_2), 70.8 (C-4), 69.7 (C-6), 64.7 (C-2), 21.3 (CH_3), 20.9 (CH_3). NMR data were consistent with literature data.[16] **ESI-HRMS** for $C_{29}H_{35}O_5N_4S$ $(M+NH_4)^+$ calculated: 551.2323; found: 551.2325.

4-*O*-Acetyl-2-azido-3,6-di-*O*-benzyl-2-deoxy-α/β-D-glucopyranose 2c

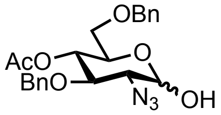

A solution of **1c** (277 mg, 0.519 mmol) in 9:1 acetone/ H_2O (7 mL) and treated with NBS (278 mg, 1.56 mmol) at room temperature. After 4 h the reaction was quenched with saturated $Na_2S_2O_3$ (30 mL) and diluted with CH_2Cl_2 (50 mL). The organic layer was washed with H_2O (20 mL), dried over anhydrous $MgSO_4$, filtered and concentrated *in vacuo* to give a yellow syrup. Purification by column chromatography (2:1 to 1:1, petroleum ether/Et_2O) afforded the hydrolysed product **2c** as a white solid (176 mg, 79% yield, α/β = 61:39) along with starting material **1c** (39 mg, 14% recovered). R_f = 0.26, 95:5; CH_2Cl_2/Et_2O.

The following were observed for α and β anomers: **^{1}H NMR** (400 MHz, $CDCl_3$): δ 7.40 – 7.26 (m, 10H, Ph), 4.51 (s, 2H, 2 x C*H*HPh). **^{13}C{^{1}H} NMR** (101 MHz, $CDCl_3$): δ 128.63 (CH), 128.62 (CH), 128.57 (CH), 128.5 (CH), 128.20 (CH), 128.17 (CH), 128.12 (CH), 128.08 (CH), 128.02 (CH), 127.98 (CH).

α-anomer

^{1}H NMR (400 MHz, $CDCl_3$): δ 7.40 – 7.26 (m, 10H, Ph), 5.31 (t, J = 3.6 Hz, 1H, H-1), 5.01 (t, J = 9.7 Hz, 1H, H-4), 4.82 (d, J = 11.1 Hz, 1H, C*H*HPh), 4.63 (d, J = 11.1 Hz, 1H, C*H*HPh), 4.51 (s, 2H, 2 x C*H*HPh), 4.14 (ddd, J = 9.8, 5.8, 3.3 Hz, 1H, H-5), 3.99 (t, J = 9.7 Hz, 1H, H-3), 3.56 (d, J = 3.5 Hz, 1H, OH), 3.50 – 3.42 (m, 3H, H-6a, H-6b, H-2), 1.86 (s, 3H, CH_3).

$^{13}C\{^1H\}$ NMR (101 MHz, $CDCl_3$): δ 169.83 (C=O), 137.74 (C), 137.6 (C), 92.0 (C-1), 77.8 (C-3), 75.0 (Ph*C*H_2), 73.7 (Ph*C*H_2), 71.2 (C-4), 69.25 (C-5), 69.16 (C-6), 63.7 (C-2), 20.91 (CH_3).

β-anomer

^1H NMR (400 MHz, $CDCl_3$): δ 4.98 – 4.90 (m, 1H, H-4), 4.81 (d, *J* = 11.4 Hz, 1H, C*H*HPh), 4.61 (d, *J* = 11.4 Hz, 1H, C*H*HPh), 4.61 – 4.54 (m, 1H, H-1), 3.96 (d, *J* = 5.4 Hz, 1H, OH), 3.59 – 3.48 (m, 3H, H-5, H-6a, H-6b), 3.42 – 3.37 (m, 2H, H-2, H-3), 1.84 (s, 3H, CH_3).
$^{13}C\{^1H\}$ NMR (101 MHz, $CDCl_3$) δ 169.76 (C=O), 137.75 (C), 137.5 (C), 96.3 (C-1), 80.5 (C-3), 75.1 (Ph*C*H_2), 73.8 (Ph*C*H_2), 73.6 (C-5), 70.7 (C-4), 69.32 (C-6), 67.1 (C-2), 20.87 (CH_3). NMR data were consistent with literature data.[17]

ESI-HRMS for $C_{22}H_{29}O_6N_4$ $(M+NH_4)^+$ calculated: 445.2082; found: 445.2082.

2-Azido-3-*O*-benzyl-6-*O*-*tert*-butyldiphenylsilyl-2-deoxy-4-*O*-levulinoyl-α/β-D-glucopyranose 3a

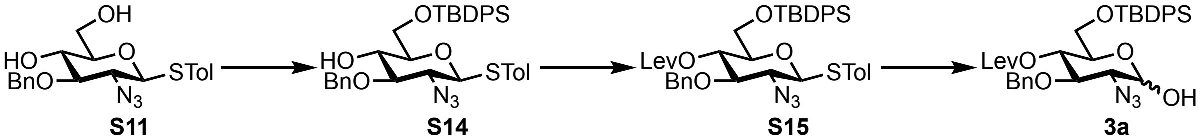

Under a N_2 atmosphere, a solution **S11** (3.58, 8.92 mmol) and imidazole (1.82 g, 26.8 mmol) in anhydrous DMF (18 mL) was treated with TBDPSCl (3.5 mL, 13 mmol) and left to stir at room temperature for 1 h. The reaction mixture was diluted with Et_2O (150 mL) and washed with H_2O (100 mL), dried over anhydrous $MgSO_4$, filtered and concentrated *in vacuo* to give a pale yellow syrup. R_f = 0.76, CH_2Cl_2.

Under a N_2 atmosphere, a solution of crude **S14**, DMAP (109 mg, 0.892 mmol) and levulinic acid (2.08 g, 17.9 mmol) in anhydrous CH_2Cl_2 (24 mL) was treated DCC (5.53 g, 26.8 mmol). The off-white reaction mixture was stirred at room temperature for 2 h and then filtered through celite. The filtrate was concentrated *in vacuo* to give an orangish syrup. The crude material was used in the next step without further purification. R_f = 0.62, CH_2Cl_2.

The crude material **S15** was dissolved in 4:1 acetone/ H_2O (45 mL) and treated with TCCA (2.08 g, 8.93 mmol) at 0°C. After 2 h the reaction was diluted with CH_2Cl_2 (100 mL) and washed with washed with H_2O (200 mL). The organic layer was dried over anhydrous $MgSO_4$, filtered and concentrated *in vacuo* to give a syrup. Purification by column chromatography

(100:0 to 95:5, CH_2Cl_2/Et_2O) afforded the hydrolysed product **3a** as a syrup (549 mg, 69% yield over 3 steps, α/β = 56:44). R_f = 0.21, 98:2; CH_2Cl_2/Et_2O.

The following were observed for α and β anomers: **^{1}H NMR** (400 MHz, $CDCl_3$): δ 7.75 – 7.59 (m, 4H, Ph), 7.48 – 7.26 (m, 11H, Ph), 3.74 – 3.66 (m, 2H, H-6a, H-6b), 2.62 – 2.51 (m, 2H, Lev-CH_2), 2.40 – 2.22 (m, 2H, Lev-CH_2). **^{13}C{^{1}H} NMR** (101 MHz, $CDCl_3$): δ 136.0 (CH), 135.9 (CH), 135.84 (CH), 135.80 (CH), 134.1 (C), 133.8 (C), 133.6 (C), 133.3 (C), 129.84 (CH), 129.79 (CH), 129.76 (CH), 129.7 (CH), 128.55 (CH), 128.54 (CH), 128.2 (CH), 128.1 (CH), 128.0 (CH), 127.73 (CH), 127.70 (CH), 127.67 (CH), 75.0 (Ph*C*H_2).

α-anomer

^{1}H NMR (400 MHz, $CDCl_3$): δ 5.27 (d, *J* = 3.5 Hz, 1H, H-1), 5.15 (t, *J* = 9.7 Hz, 1H, H-4), 4.79 (d, *J* = 11.1 Hz, 1H, C*H*HPh), 4.66 (d, *J* = 11.1 Hz, 1H, C*H*HPh), 4.04 – 3.94 (m, 1H, H-5), 3.98 (t, *J* = 9.7 Hz, 1H, H-3), 3.49 – 3.40 (m, 1H, H-2), 2.77 (dd, *J* = 3.4, 1.4 Hz, 1H, OH), 2.122 (s, 3H, Lev-CH_3), 1.03 (s, 9H, $SiC(CH_3)_3$). **^{13}C{^{1}H} NMR** (101 MHz, $CDCl_3$): δ 206.20 (C=O), 171.4 (C=O), 137.8 (C), 92.1 (C-1), 77.9 (C-3), 71.0 (C-5), 70.7 (C-4), 63.8 (C-2), 62.9 (C-6), 37.89 (Lev-*C*H_2), 29.93 (Lev-*C*H_3), 28.0 (Lev-*C*H_2), 26.92 (SiC(*C*H_3)$_3$), 19.42 (Si*C*(CH_3)$_3$).

β-anomer

^{1}H NMR (400 MHz, $CDCl_3$): δ 5.00 (dd, *J* = 10.0, 8.7 Hz, 1H, H-4), 4.78 (d, *J* = 11.4 Hz, 1H, C*H*HPh), 4.63 (d, *J* = 11.4 Hz, 1H, C*H*HPh), 4.45 (dd, *J* = 7.5, 5.2 Hz, 1H, H-1), 3.44 – 3.31 (m, 3H, H-5, H-3, H-2), 2.99 (dd, *J* = 5.2, 1.2 Hz, 1H, OH), 2.117 (s, 3H, Lev-CH_3), 1.04 (s, 9H, $SiC(CH_3)_3$). **^{13}C{^{1}H} NMR** (101 MHz, $CDCl_3$): δ 206.18 (C=O), 171.5 (C=O), 137.9 (C), 96.2 (C-1), 80.6 (C-3), 75.3 (C-5), 70.3 (C-4), 67.3 (C-2), 63.2 (C-6), 37.85 (Lev-*C*H_2), 29.90 (Lev-*C*H_3), 27.9 (Lev-*C*H_2), 26.93 (SiC(*C*H_3)$_3$), 19.38 (Si*C*(CH_3)$_3$).

ESI-HRMS for $C_{34}H_{41}O_7N_3SiNa$ $(M+Na)^+$ calculated: 654.2606; found: 654.2617.

4-*O*-Acetyl-2-azido-3-*O*-benzyl-6-*O*-*tert*-butyldiphenylsilyl-2-deoxy-α/β-D-glucopyranose 3b

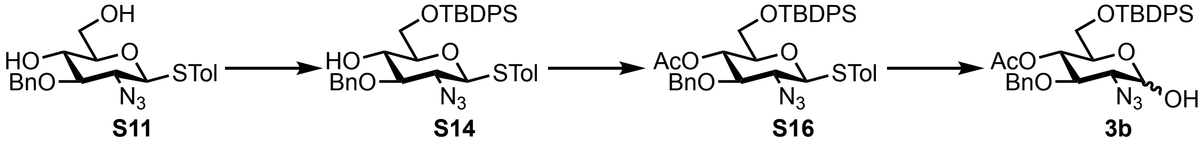

Under a N_2 atmosphere, a solution **S11** (500 mg, 1.25 mmol) and imidazole (255 mg, 3.75 mmol) in anhydrous DMF (2.5 mL) was treated with TBDPSCl (0.49 mL, 1.9 mmol) and left to stir at room temperature for 1 h. The reaction mixture was diluted with Et_2O (150 mL) and

washed with H_2O (100 mL), dried over anhydrous $MgSO_4$, filtered and concentrated *in vacuo* to give a pale yellow syrup. R_f = 0.76, CH_2Cl_2.

A solution of **S14** (370 mg, 0.578 mmol) and DMAP (8 mg, 0.06 mmol) in anhydrous CH_2Cl_2 (2.5 mL) was treated with pyridine (0.19 mL, 2.5 mmol) followed by acetic anhydride (0.24 mL, 2.5 mmol) and was stirred at room temperature for 1.5 h. The reaction mixture was diluted with CH_2Cl_2 (100 mL) and washed with 1 M HCl (40 mL) and saturated $NaHCO_3$ (60 mL). The organic layer was dried over anhydrous $MgSO_4$, filtered and concentrated *in vacuo* to give a white solid. The crude material was used in the next step without further purification. R_f = 0.71, 20:80; Hex/CH_2Cl_2. **^{1}H NMR** (400 MHz, $CDCl_3$): δ 7.75 – 7.63 (m, 4H, Ph), 7.51 (d, *J* = 8.1 Hz, 2H, Ph), 7.45 – 7.26 (m, 11H, Ph), 7.04 (d, *J* = 8.0 Hz, 2H, Ph), 5.04 (t, *J* = 9.7 Hz, 1H, H-3), 4.79 (d, *J* = 11.1 Hz, 1H, C*H*HPh), 4.59 (d, *J* = 11.1 Hz, 1H, C*H*HPh), 4.36 (d, *J* = 10.0 Hz, 1H, H-1), 3.74 (dd, *J* = 11.6, 2.1 Hz, 1H, H-6a), 3.66 (dd, *J* = 11.6, 4.6 Hz, 1H, H-6b), 3.47 (t, *J* = 9.3 Hz, 1H, H-2/3), 3.42 (ddd, *J* = 10.2, 4.7, 2.2 Hz, 1H, H-5), 3.36 (t, *J* = 9.7 Hz, 1H, H-2/3), 2.31 (s, 3H, CH_3), 1.80 (s, 3H, CH_3), 1.05 (s, 9H, $SiC(CH_3)_3$).

The crude material **S16** was dissolved in 9:1 acetone/ H_2O (6.3 mL) and treated with NBS (890 mg, 5.01 mmol) at room temperature. After 5 h the reaction was quenched with saturated $Na_2S_2O_3$ (80 mL) and diluted with Et_2O (100 mL). The organic layer was washed with H_2O (80 mL), dried over anhydrous $MgSO_4$, filtered and concentrated *in vacuo*. Purification by column chromatography (100:0 to 97:3, CH_2Cl_2/Et_2O) afforded the hydrolysed product **3b** as a syrup (460 mg, 64% yield over 3 steps, α/β = 63:37). R_f = 0.48, CH_2Cl_2.

The following were observed for α and β anomers: **^{1}H NMR** (400 MHz, $CDCl_3$): δ 7.71 – 7.62 (m, 4H, Ph), 7.51 – 7.26 (m, 11H, Ph), 3.73 – 3.60 (m, 2H, H-6a, H-6b). **^{13}C{^{1}H} NMR** (101 MHz, $CDCl_3$): δ 129.9 (CH), 129.83 (CH), 129.81 (CH), 129.79 (CH), 128.63 (CH), 128.61 (CH), 128.3 (CH), 128.13 (CH), 128.08 (CH), 128.06 (CH), 127.8 (CH), 127.74 (CH), 127.71 (CH).

α-anomer

^{1}H NMR (400 MHz, $CDCl_3$): δ 5.28 (t, *J* = 3.5 Hz, 1H, H-1), 5.12 (dd, *J* = 10.2, 9.2 Hz, 1H, H-4), 4.81 (d, *J* = 11.0 Hz, 1H, C*H*HPh), 4.62 (d, *J* = 11.0 Hz, 1H, C*H*HPh), 4.03 – 3.3.97 (t, *J* = 9.6 Hz, 1H, H-3), 3.47 (ddd, *J* = 10.3, 3.5, 1.4 Hz, 1H, H-2), 2.74 (dd, *J* = 3.4, 1.3 Hz, 1H, OH), 1.83 (s, 3H, CH_3), 1.04 (s, 9H, $SiC(CH_3)_3$). **^{13}C{^{1}H} NMR** (101 MHz, $CDCl_3$): δ 169.53 (C=O), 137.69 (C), 135.89 (CH) , 135.81 (CH), 133.6 (C), 133.5 (C), 92.1 (C-1), 78.0 (C-3), 75.03 (Ph*C*H_2), 71.1 (C-5), 70.4 (C-4), 63.8 (C-2), 63.0 (C-6), 26.92 (SiC(*C*H_3)$_3$), 20.9 (CH_3), 19.4 (Si*C*(CH_3)$_3$).

β-anomer

^{1}H NMR (400 MHz, $CDCl_3$): δ 5.01 – 4.93 (m, 1H, H-4), 4.80 (d, J = 11.3 Hz, 1H, C*H*HPh), 4.59 (d, J = 11.3 Hz, 1H, C*H*HPh), 4.46 (dd, J = 7.5, 5.1 Hz, 1H, H-1), 3.45 – 3.31 (m, 3H, H-5, H-3, H-2), 2.98 (d, J = 5.1 Hz, 1H, OH), 1.80 (s, 3H, CH_3), 1.04 (s, 9H, $SiC(CH_3)_3$). **^{13}C{^{1}H} NMR** (101 MHz, $CDCl_3$): δ 169.51 (C=O), 137.74 (C), 136.0 (CH), 135.78 (CH), 134.0 (C), 133.2 (C), 96.2 (C-1), 80.7 (C-3), 75.4 (C-5), 75.02 (Ph*C*H_2), 70.0 (C-4), 67.3 (C-2), 63.3 (C-6), 26.94 (SiC(*C*H_3)$_3$), 20.8 (CH_3), 19.3 (Si*C*(CH_3)$_3$).

ESI-HRMS for $C_{31}H_{37}O_6N_3SiNa$ $(M+Na)^+$ calculated: 598.2344; found: 598.2327.

2-Azido-3-*O*-benzyl-6-*O*-*tert*-butyldiphenylsilyl-2-deoxy-4-*O*-levulinoyl-1-*O*-trichloroacetimidoyl-β-D-glucopyranose S17

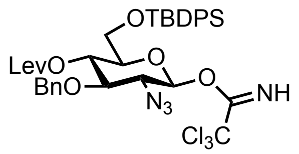

Under a N_2 atmosphere, a solution of **3a** (380 mg, 0.601 mmol) and K_2CO_3 (125 mg, 0.902 mmol) in anhydrous CH_2Cl_2 (3 mL) was treated with CCl_3CN (0.30 mL, 3.0 mmol) and stirred at room temperature. After 15 h the reaction mixture was concentrated to half the volume and purified by column chromatography (R_f = 0.74, 96:4; CH_2Cl_2/Et_2O with 0.5% Et_3N) to give a pale yellow syrup which was treated with petroleum ether/Et_2O to precipitate out β-**S17** (301 mg, 65% yield). **^{1}H NMR** (400 MHz, $CDCl_3$): δ 8.76 (s, 1H, NH), 7.71 – 7.61 (m, 4H, Ph), 7.45 – 7.26 (m, 11H, Ph), 5.72 (d, J = 8.5 Hz, 1H, H-1), 5.30 – 5.22 (m, 1H, H-4), 4.83 (d, J = 11.4 Hz, 1H, C*H*HPh), 4.71 (d, J = 11.4 Hz, 1H, C*H*HPh), 3.80 – 3.67 (m, 3H, H-2, H-6a, H-6b), 3.62 – 3.52 (m, 2H, H-5, H-3), 2.67 – 2.57 (m, 2H, Lev-CH_2), 2.43 – 2.33 (m, 2H, Lev-CH_2), 2.13 (s, 3H, Lev-CH_3), 1.01 (s, 9H, $SiC(CH_3)_3$). **^{13}C{^{1}H} NMR** (101 MHz, $CDCl_3$): δ 206.0 (C=O), 171.3 (C=O), 161.0 (C=O), 137.8 (C), 135.9 (CH), 135.8 (CH), 133.44 (C), 133.42 (C), 129.71 (CH), 129.67 (CH), 128.6 (CH), 128.2 (CH), 128.1 (CH), 127.72 (CH), 127.69 (CH), 96.6 (C-1), 90.7 (CCl3), 80.7 (C-3), 75.7 (C-5), 75.1 (Ph*C*H_2), 69.6 (C-4), 65.6 (C-2), 62.2 (C-6), 37.9 (Lev-*C*H_2), 29.9 (Lev-*C*H_3), 28.0 (Lev-*C*H_2), 26.7 (SiC(*C*H_3)$_3$), 19.4 (Si*C*(CH_3)$_3$). **ESI-HRMS** for $C_{36}H_{41}O_7N_4Cl_3SiNa$ $(M+Na)^+$ calculated: 797.1702; found: 797.1706.

Glycosylations

General Procedure A

In a multi-neck RBF, freshly activated 4Å molecular sieves, diphenyl sulfoxide (2.8 eq) and TTBP (3 eq) were placed under three cycles of vacuum and N_2. A stock solution of the hemiacetal donor in anhydrous CH_2Cl_2 (0.02 M, 1 eq) was then added to the flask. The reagents were pre-dried under a N_2 atmosphere for 0.5-1 h. The solution was cooled down to -60 °C (using $CHCl_3$ and dry ice) and then treated with triflic anhydride (1.4 eq). The solution was allowed to gradually warm up to -40 °C over a period of 1 h. A stock solution of the glucuronic acceptor in anhydrous CH_2Cl_2 (0.1 M, 0.8 eq) was then added to the flask at -40 °C. The reaction mixture was gradually warmed up to room temperature. After 5 h, the reaction mixture was filtered and washed with H_2O and saturated $NaHCO_3$. The organic layer was dried over anhydrous $MgSO_4$, filtered and concentrated *in vacuo* to give a pale yellow paste.

General Procedure B

In a multi-neck RBF, freshly activated 4Å molecular sieves, diphenyl sulfoxide (2.8 eq) and TTBP (3 eq) were placed under three cycles of vacuum and N_2. A stock solution of the hemiacetal donor in anhydrous CH_2Cl_2 (0.05 M, 1 eq) was then added to the flask. The reagents were pre-dried under a N_2 atmosphere for 0.5 h. The solution was cooled down to -60 °C (using $CHCl_3$ and dry ice) and then treated with triflic anhydride (1.4 eq). The solution was allowed to gradually warm up to -48 °C over a period of 1 h. A stock solution of the glucuronic acceptor in anhydrous CH_2Cl_2 (0.3 M, 2 eq) was then added to the flask at -60 °C. Once the $CHCl_3$/dry ice cooling bath warms up to -48 °C, the cooling bath was replaced with a MeCN/dry ice cooling bath. The reaction mixture was gradually warmed up to -10 °C. After 8 h (i.e. after addition of the acceptor), the reaction mixture was filtered and washed with H_2O and saturated $NaHCO_3$. The organic layer was dried over anhydrous $MgSO_4$, filtered and concentrated *in vacuo* to give a pale yellow paste.

Note: After completion of reaction, wash the molecular sieves with plenty of CH_2Cl_2 *to avoid loss of material.*

_p_-Tolyl benzyl (2-azido-3,6-di-_O_-benzyl-2-deoxy-4-_O_-fluorenylmethoxycarbonyl-α-D-glucopyranosyl)-(1→4)-(2-_O_-benzoyl-3-_O_-benzyl-1-thio-β-D-glucopyranosyluronate) 5a

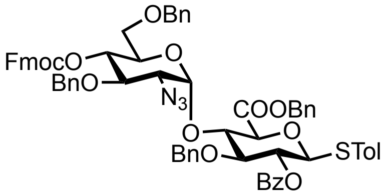

Following the general procedure A, hemiacetal donor **2a** (100 mg, 0.165 mmol), glucuronic acceptor **4a** (77 mg, 0.13 mmol), diphenyl sulfoxide (93 mg, 0.46 mmol), TTBP (123 mg, 0.495 mmol) and triflic anhydride (39 μL, 0.23 mmol) were used. Purification by column chromatography (100:0 to 97:3; CH_2Cl_2/Et_2O) gave **5a** a cloudy syrup (22 mg, 14% yield). $[\alpha]_D^{21}$ = +16.6 (c 0.5, $CHCl_3$) **^{1}H NMR** (400 MHz, $CDCl_3$): δ 8.11 – 8.02 (m, 2H, Ph), 7.73 (dd, *J* = 7.6, 2.8 Hz, 2H, Ph), 7.62 – 7.51 (m, 3H, Ph), 7.50 – 7.42 (m, 5H, Ph), 7.42 – 6.97 (m, 25H, Ph), 5.51 (d, *J* = 3.8 Hz, 1H, H-1'), 5.31 – 5.25 (m, 1H, H-2), 5.19 (s, 2H, 2 x C*H*HPh), 5.03 (t, *J* = 9.7 Hz, 1H, H-4'), 4.79 (d, *J* = 9.8 Hz, 1H, H-1), 4.73 (d, *J* = 10.1 Hz, 1H, C*H*HPh), 4.69 (d, *J* = 11.0 Hz, 1H, C*H*HPh), 4.64 (d, *J* = 10.1 Hz, 1H, C*H*HPh), 4.60 (d, *J* = 11.0 Hz, 1H, C*H*HPh), 4.49 (d, *J* = 12.0 Hz, 1H, C*H*HPh), 4.41 (d, *J* = 12.0 Hz, 1H, C*H*HPh), 4.31 – 4.21 (m, 3H, Fmoc-CH_2, H-4), 4.12 (d, *J* = 9.4 Hz, 1H, H-5), 4.07 (t, *J* = 7.2 Hz, 1H, Fmoc-CH), 3.99 (t, *J* = 8.6 Hz, 1H, H-3), 3.89 (dd, *J* = 10.4, 9.2 Hz, 1H, H-3'), 3.75 – 3.66 (m, 1H, H-5'), 3.57 (dd, *J* = 10.6, 3.7 Hz, 1H, H-6a), 3.48 (dd, *J* = 10.6, 3.0 Hz, 1H, H-6b), 3.32 (dd, *J* = 10.4, 3.8 Hz, 1H, H-2), 2.32 (s, 3H, CH_3). **^{13}C{^{1}H} NMR** (101 MHz, $CDCl_3$): δ 167.7 (C=O), 165.1 (C=O), 154.3 (C=O), 143.5 (C), 143.4 (C), 141.42 (C), 141.39 (C), 138.8 (C), 137.9 (C), 137.5 (C), 137.2 (C), 135.0 (C), 134.1 (CH), 133.6 (CH), 130.0 (CH), 129.8 (CH), 128.8 (CH), 128.7 (CH), 128.5 (CH), 128.4 (CH), 128.0 (CH), 127.8 (CH), 127.7 (CH), 127.31 (CH), 127.28 (CH), 125.2 (CH), 125.1 (CH), 120.21 (CH), 120.19 (CH), 97.6 (C-1'), 86.8 (C-1), 84.0 (C-3), 78.1 (C-5), 77.7 (C-3'), 75.6 (C-4'), 75.1 (Ph*C*H_2), 75.0 (Ph*C*H_2), 74.7 (C-4), 73.8 (Ph*C*H_2), 72.2 (C-2), 70.1 (Fmoc-*C*H_2), 69.3 (C-5'), 68.5 (C-6'), 67.8 (Ph*C*H_2), 62.7 (C-2'), 46.8 (Fmoc-*C*H), 21.3 (CH_3). **ESI-HRMS** for $C_{69}H_{67}O_{13}N_4S$ $(M+NH_4)^+$ calculated: 1191.4420; found: 1191.4405.

***p*-Tolyl benzyl (2-azido-3,6-di-*O*-benzyl-2-deoxy-4-*O*-levulinoyl-α-D-glucopyranosyl)-(1→4)-(2-*O*-benzoyl-3-*O*-benzyl-1-thio-β-D-glucopyranosyluronate) 8a**

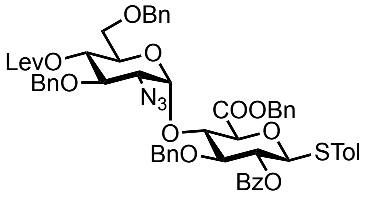

Following the general procedure A, hemiacetal donor **2b** (80 mg, 0.17 mmol), glucuronic acceptor **4a** (77 mg, 0.13 mmol), diphenyl sulfoxide (93 mg, 0.46 mmol), TTBP (123 mg, 0.495 mmol) and triflic anhydride (39 μL, 0.23 mmol) were used. Purification by column chromatography (98:2 to 95:5; CH_2Cl_2/Et_2O) gave **8a** and **4a** as an inseparable mixture as a cloudy syrup (78 mg, ^{1}H NMR spectrum of the syrup showed **8a**:**4a** = 61:39), calculated yield **8a** = 41% and **4a** = 27%. R_f = 0.5, 97:3; CH_2Cl_2/Et_2O.

α-anomer

^{1}H NMR (400 MHz, $CDCl_3$) selected signals: δ 5.51 (d, *J* = 3.4 Hz, 1H, H-1'), 5.33 – 5.11 (m, 2H, H-2, H-4'), 4.79 (d, *J* = 9.9 Hz, 1H, H-1), 4.41 (s, 2H, 2 x C*H*HPh), 4.26 (t, *J* = 9.0 Hz, 1H, H-4), 4.13 (d, *J* = 9.5 Hz, 1H, H-5), 4.03 – 3.96 (m, 1H, H-3), 3.88 (t, *J* = 9.8 Hz, 1H, H-3'), 3.71 – 3.62 (m, 1H, H-5'), 3.52 (dd, *J* = 10.4, 3.3 Hz, 1H, H-6a'), 3.44 (dd, *J* = 10.4, 2.9 Hz, 1H, H-6b'), 3.32 (dd, *J* = 10.3, 3.4 Hz, 1H, H-2'), 2.58 – 2.51 (m, 2H, Lev-CH_2), 2.33 – 2.25 (m, 2H, Lev-CH_2), 2.31 (s, 3H, CH_3), 2.09 (s, 3H, Lev-CH_3). **^{13}C{^{1}H} NMR** (101 MHz, $CDCl_3$) selected signals: δ 206.3 (C=O), 171.4 (C=O), 167.7 (C=O), 165.1 (C=O), 138.8 (C), 138.0 (C), 137.2 (C), 135.0 (C), 134.1 (CH), 97.7 (C-1', $^1J_{1CH}$ = 179.0 Hz, from coupled HSQC), 86.7 (C-1, $^1J_{1CH}$ = 158.4 Hz, from coupled HSQC), 84.0 (C-3), 78.3 (C-5), 77.7 (C-3'), 74.8 (Ph*C*H_2 or C-4), 74.7 (Ph*C*H_2 or C-4), 73.8 (Ph*C*H_2), 72.2 (C-2), 71.2 (C-4'), 69.6 (C-5'), 68.5 (C-6'), 67.6 (Ph*C*H_2), 62.8 (C-2'), 37.8 (Lev-*C*H_2), 29.9 (Lev-*C*H_3), 28.0 (Lev-*C*H_2), 21.3 (CH_3). **ESI-HRMS** for $C_{59}H_{63}O_{13}N_4S$ $(M+NH_4)^+$ calculated: 1067.4107; found: 1067.4111.

***p*-Tolyl benzyl (4-*O*-acetyl-2-azido-3,6-di-*O*-benzyl-2-deoxy-α-D-glucopyranosyl)-(1→4)-(2-*O*-benzoyl-3-*O*-benzyl-1-thio-β-D-glucopyranosyluronate) 9a and benzyl (1,2,4-*O*-orthobenzoyl-3-*O*-benzyl-α-D-glucopyranosyluronate) 6a**

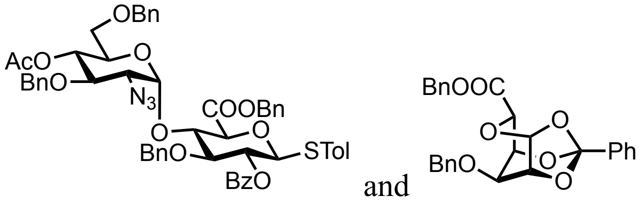

Following the general procedure A, hemiacetal donor **2c** (70.5 mg, 0.165 mmol), glucuronic acceptor **4a** (77 mg, 0.13 mmol), diphenyl sulfoxide (93 mg, 0.46 mmol), TTBP (123 mg, 0.495 mmol) and triflic anhydride (39 μL, 0.23 mmol) were used. Purification by column chromatography (2:1 to 1:1; petroleum ether/Et2O) gave an inseparable mixture of **9a** along with other side products (acceptor, ortho-ester of acceptor, 1,6-anhydro sugar of donor) as a cloudy syrup. ^{1}H NMR spectrum of fraction 1 (56 mg) showed **9a**:**6a:12** = 55:17:28, and fraction 2 (31 mg) showed **9a**:**4a** = 65:35, calculated yield **9a** = 52% (α/β = 89:11). R_f = 0.5, 1:1; petroleum ether/Et_2O.

9a α-anomer

^{1}H NMR (400 MHz, $CDCl_3$) selected signals: δ 5.51 (d, *J* = 3.8 Hz, 1H, H-1’), 5.33 – 5.25 (m, 1H, H-2), 5.20 – 5.12 (m, 1H, H-4’), 4.83 – 4.76 (m, 1H, H-1), 4.65 (d, *J* = 10.2 Hz, 1H, C*H*HPh), 4.56 (d, *J* = 11.0 Hz, 1H, C*H*HPh), 4.46 (d, *J* = 11.8 Hz, 1H, C*H*HPh), 4.37 (d, *J* = 11.8 Hz, 1H, C*H*HPh), 4.31 – 4.22 (m, 1H, H-4), 4.14 (d, *J* = 9.5 Hz, 1H, H-5), 4.03 – 3.97 (m, 1H, H-3), 3.85 (t, *J* = 9.8 Hz, 1H, H-3’), 3.65 (dt, *J* = 10.1, 3.7 Hz, 1H, H-5’), 3.49 (dd, *J* = 10.4, 4.1 Hz, 1H, H-6a’), 3.39 (dd, *J* = 10.4, 3.2 Hz, 1H, H-6b’), 3.33 (dd, *J* = 10.4, 3.8 Hz, 1H, H-2’), 2.31 (s, 3H, CH_3), 1.81 (s, 1H), 1.78 (s, 3H, CH_3). **^{13}C{^{1}H} NMR** (101 MHz, $CDCl_3$) selected signals: δ 169.4 (C=O), 167.7 (C=O), 165.1 (C=O), 138.8 (C), 137.7 (C), 137.2 (C), 135.0 (C), 134.1 (CH), 97.7 (C-1’, $^{1}J_{CH}$ = 179.0 Hz, from coupled HSQC), 86.7 (C-1, $^{1}J_{CH}$ = 157.2 Hz, from coupled HSQC), 84.0 (C-3), 78.2 (C-5), 77.8 (C-3’), 74.8 (PhCH_2 or C-4), 74.7 (PhCH_2 or C-4), 73.8 (PhCH_2), 72.2 (C-2), 71.1 (C-4’), 69.6 (C-5’), 68.7 (C-6’), 67.6 (PhCH_2), 62.8 (C-2), 20.9 (CH_3).

9a β-anomer

^{1}H NMR (400 MHz, $CDCl_3$) selected signal: δ 4.39 (d, *J* = 7.9 Hz, 1H, H-1’). **^{13}C{^{1}H} NMR** (101 MHz, $CDCl_3$) selected signal: δ 101.5 (C-1’), 87.4 (C-1).

ESI-HRMS for $C_{56}H_{59}O_{12}N_4S$ $(M+NH_4)^+$ calculated: 1011.3845; found: 1011.3858.

6a

^{1}H NMR (400 MHz, $CDCl_3$) selected signals: δ 6.05 (d, *J* = 4.9 Hz, 1H, H-1), 5.10 (s, 1H, H-5), 5.05 (dd, *J* = 4.7, 2.1 Hz, 1H, H-4), 4.65 – 4.59 (m, 1H, H-2), 4.10 (dd, *J* = 4.7, 2.1 Hz, 1H, H-3). **^{13}C{^{1}H} NMR** (101 MHz, $CDCl_3$) selected signals: δ 97.7 (C-1), 75.7 (C-5) , 73.5 (C-4), 72.8 (C-2), 69.5 (C-3). **ESI-HRMS** for $C_{27}H_{25}O_7$ $(M+H)^+$ calculated: 461.1595; found: 461.1598.

***p*-Tolyl benzyl (2-azido-3,6-di-*O*-benzyl-2-deoxy-4-*O*-levulinoyl-α-D-glucopyranosyl)-(1→4)-(2-*O*-benzoyl-3-*O*-(*p*-methylbenzyl)-1-thio-β-D-glucopyranosyluronate) 8b**

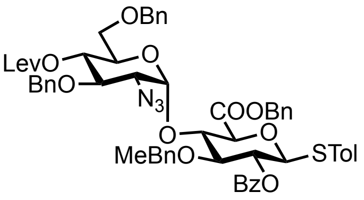

Following the general procedure A, hemiacetal donor **2b** (79.8 mg, 0.165 mmol), glucuronic acceptor **4b** (79 mg, 0.13 mmol), diphenyl sulfoxide (93 mg, 0.46 mmol), TTBP (123 mg, 0.495 mmol) and triflic anhydride (39 µL, 0.23 mmol) were used. Purification by column chromatography (100:0 to 97:3; CH_2Cl_2/Et_2O) gave **8b** and **4b** as an inseparable mixture as a cloudy syrup (20 mg, ^{1}H NMR spectrum of the syrup showed **8b**:**4b** = 61:39), calculated yield **8b** = 11%. R_f = 0.38, 97:3; CH_2Cl_2/Et_2O. **^{1}H NMR** (400 MHz, $CDCl_3$) selected signals: δ 5.52 (d, *J* = 3.6 Hz, 1H, H-1'), 5.31 – 5.23 (m, 1H, H-2), 5.22 – 5.15 (m, 1H, H-4'), 4.78 (d, *J* = 9.8 Hz, 1H, H-1), 4.41 (s, 2H, 2 x C*H*HPh), 4.24 (t, *J* = 9.0 Hz, 1H, H-4), 4.12 (d, *J* = 9.5 Hz, 1H, H-5), 4.01 – 3.92 (m, 1H, H-3), 3.87 (t, *J* = 9.8 Hz, 1H, H-3'), 3.69 – 3.61 (m, 1H, H-5'), 3.52 (dd, *J* = 10.5, 3.7 Hz, 1H, H-6a'), 3.43 (dd, *J* = 10.5, 3.1 Hz, 1H, H-6b'), 3.32 (dd, *J* = 10.4, 3.6 Hz, 1H, H-2'), 2.54 (td, *J* = 6.4, 2.5 Hz, 2H, Lev-CH_2), 2.32 – 2.26 (m, 2H, Lev-CH_2), 2.09 (s, 3H, Lev-CH_3). **^{13}C{^{1}H} NMR** (101 MHz, $CDCl_3$) selected signals: δ 206.3 (C=O), 171.4 (C=O), 167.7 (C=O), 165.0 (C=O), 138.7 (C), 138.0 (C), 137.8 (C), 137.7 (C), 135.0 (C), 134.1 (CH), 97.7 (C-1'), 86.7 (C-1), 83.9 (C-3), 78.3 (C-5), 77.7 (C-3'), 73.8 (Ph*C*H_2), 72.2 (C-2), 71.3 (C-4'), 69.6 (C-5'), 68.5 (C-6'), 67.6 (Ph*C*H_2), 62.8 (C-2'), 37.8 (Lev-*C*H_2), 29.9 (Lev-*C*H_3), 28.0 (Lev-*C*H_2), 21.3 (CH_3). **ESI-HRMS** for $C_{60}H_{65}O_{13}N_4S$ $(M+NH_4)^+$ calculated: 1081.4263; found: 1081.4258.

***p*-Tolyl benzyl (4-*O*-acetyl-2-azido-3,6-di-*O*-benzyl-2-deoxy-α-D-glucopyranosyl)-(1→4)-(2-*O*-benzoyl-3-*O*-(*p*-methylbenzyl)-1-thio-β-D-glucopyranosyluronate) 9b**

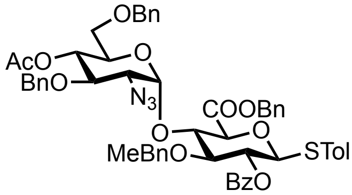

Following the general procedure A, hemiacetal donor **2c** (70.5 mg, 0.165 mmol), glucuronic acceptor **4b** (79 mg, 0.13 mmol), diphenyl sulfoxide (93 mg, 0.46 mmol), TTBP (123 mg, 0.495 mmol) and triflic anhydride (39 μL, 0.23 mmol) were used Purification by column chromatography (100:0 to 97:3; CH_2Cl_2/Et_2O gave an inseparable mixture of **9b** along with other side products (acceptor, ortho-ester of acceptor) as a cloudy syrup. ^{1}H NMR spectrum of fraction 1 (60 mg) showed **9b**:**7b** = 73:27, and fraction 2 (15 mg) showed **9b**:**4b** = 50:50, calculated yield **9b** = 45% (α/β =83:17). R_f = 0.4, 97:3; CH_2Cl_2/Et_2O.

α-anomer

^{1}H NMR (400 MHz, $CDCl_3$) selected signals: δ 5.52 (d, *J* = 3.7 Hz, 1H, H-1'), 5.30 – 5.23 (m, 1H, H-), 5.21 – 5.12 (m, 1H, H-4'), 4.78 (d, *J* = 9.8 Hz, 1H, H-1), 4.61 (d, *J* = 10.1 Hz, 1H, C*H*HPh), 4.56 (d, *J* = 10.9 Hz, 1H, C*H*HPh), 4.46 (d, *J* = 11.8 Hz, 1H, C*H*HPh), 4.37 (d, *J* = 11.8 Hz, 1H, C*H*HPh), 4.28 – 4.21 (m, 1H, H-4), 4.13 (d, *J* = 9.5 Hz, 1H, H-5), 3.99 (t, *J* = 8.6 Hz, 1H, H-3), 3.85 (t, *J* = 9.8 Hz, 1H, H-3'), 3.65 (dt, *J* = 10.1, 3.6 Hz, 1H, H-5'), 3.48 (dd, *J* = 10.4, 4.0 Hz, 1H, H-6a'), 3.39 (dd, *J* = 10.4, 3.1 Hz, 1H, H-6b'), 3.33 (dd, *J* = 10.4, 3.7 Hz, 1H, H-2), 2.31 (s, 3H, CH_3), 2.23 (s, 3H, CH_3), 1.78 (s, 3H, CH_3). **^{13}C{^{1}H} NMR** (101 MHz, $CDCl_3$) selected signals: δ 169.4 (C=O), 167.7 (C=O), 165.0 (C=O), 97.6 (C-1', $^1J_{CH}$ = 179.0 Hz, from coupled HSQC), 86.7 (C-1, $^1J_{CH}$ = 157.4 Hz, from coupled HSQC), 83.9 (C-3), 78.2 (C-5), 77.8 (C-3'), 73.8 (PhCH_2) , 72.2 (C-2), 71.2 (C-4'), 69.6 (C-5'), 68.7 (C-6'), 67.6 (PhCH_2), 62.8 (C-2'), 21.3 (CH_3), 21.2, 20.9.

β-anomer

^{1}H NMR (400 MHz, $CDCl_3$) selected signals: δ 4.40 – 4.35 (m , 1H, H-1', from HSQC), 4.09 (d, *J* = 9.6 Hz, 1H, H-5).

ESI-HRMS for $C_{57}H_{61}O_{12}N_4S$ $(M+NH_4)^+$ calculated: 1025.4001; found: 1025.4001.

***p*-Tolyl benzyl (2-azido-3-*O*-benzyl-6-*O*-*tert*-butyldiphenylsilyl-2-deoxy-4-*O*-levulinoyl-α-D-glucopyranosyl)-(1→4)-(2-*O*-benzoyl-3-*O*-benzyl-1-thio-β-D-glucopyranosyluronate) 10a**

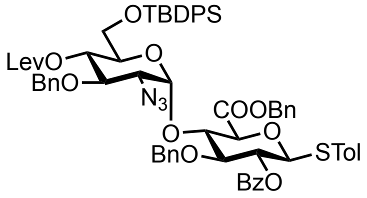

Following the general procedure A, hemiacetal donor **3a** (90.0 mg, 0.142 mmol), glucuronic acceptor **4a** (66.7 mg, 0.114 mmol), diphenyl sulfoxide (80.5 mg, 0.398 mmol), TTBP (106 mg, 0.426 mmol) and triflic anhydride (33 μL, 0.20 mmol) were used. Purification by column chromatography (100:0 to 97:3; CH_2Cl_2/Et_2O) gave **10a** as a white solid (62 mg, 45% yield). R_f = 0.5, 98:2; CH_2Cl_2/Et_2O . $[\alpha]_D^{21}$ = +33.3 (c 0.5, $CHCl_3$).

Scale-up

Following the general procedure A, hemiacetal donor **3a** (500 mg, 0.791 mmol), glucuronic acceptor **4a** (370 mg, 0.633 mmol), diphenyl sulfoxide (447 mg, 2.21 mmol), TTBP (589 mg, 2.37 mmol) and triflic anhydride (0.18 mL, 1.1 mol) were used. Purification by column chromatography (100:0 to 99:1; CH_2Cl_2/Et_2O) gave **10a** as a white solid (316 mg, 42% yield).

^{1}H NMR (400 MHz, $CDCl_3$): δ 8.11 – 8.02 (m, 2H), 7.67 – 7.56 (m, 6H), 7.47 (t, *J* = 7.8 Hz, 2H), 7.42 – 7.25 (m, 17H), 7.22 – 7.12 (m, 5H), 6.99 (d, *J* = 7.9 Hz, 2H), 5.45 (d, *J* = 3.7 Hz, 1H, H-1'), 5.35 (t, *J* = 9.7 Hz, 1H, H-4'), 5.29 (dd, *J* = 9.8, 8.7 Hz, 1H, H-2), 5.03 (d, *J* = 12.5 Hz, 1H, C*H*HPh), 4.94 (d, *J* = 12.5 Hz, 1H, C*H*HPh), 4.79 – 4.74 (m, 2H, H-1, C*H*HPh), 4.71 (d, *J* = 11.0 Hz, 1H, C*H*HPh), 4.66 (d, *J* = 10.2 Hz, 1H, C*H*HPh), 4.59 (d, *J* = 11.0 Hz, 1H, C*H*HPh), 4.20 (dd, *J* = 9.4, 8.4 Hz, 1H, H-4), 4.10 (d, *J* = 9.3 Hz, 1H, H-5), 3.99 (t, *J* = 8.5 Hz, 1H, H-3), 3.88 (dd, *J* = 10.4, 9.2 Hz, 1H, H-3'), 3.70 (dd, *J* = 12.0, 2.4 Hz, 1H, H-6a'), 3.66 – 3.57 (m, 2H, H-6b', H-5), 3.31 (dd, *J* = 10.4, 3.7 Hz, 1H, H-2'), 2.60 (t, *J* = 6.7 Hz, 2H, Lev-CH_2), 2.36 (t, *J* = 6.7 Hz, 2H, Lev-CH_2), 2.30 (s, 3H, CH_3), 2.12 (s, 3H, Lev-CH_3), 1.01 (s, 9H, $SiC(CH_3)_3$). **^{13}C{^{1}H} NMR** (101 MHz, $CDCl_3$): δ 206.1 (C=O), 171.0 (C=O), 167.5 (C=O), 165.1 (C=O), 138.7 (C), 137.8 (C), 137.3 (C), 135.92 (CH), 135.91 (CH), 134.9 (C), 134.1 (CH), 133.6 (C or CH), 133.5 (C or CH), 133.3 (C), 130.0 (CH), 129.8 (CH), 129.74 (CH), 129.71 (CH), 128.74 (CH), 128.69 (CH), 128.6 (CH), 128.52 (CH), 128.51 (CH), 128.14 (CH), 128.13 (CH), 128.02 (CH), 127.96 (CH), 127.9 (CH), 127.72 (CH), 127.65 (C or CH), 97.7 (C-1', $^1J_{1CH}$ = 177.9 Hz, from coupled HSQC), 86.6 (C-1, $^1J_{1CH}$ = 157.0 Hz, from coupled HSQC), 83.8 (C-3), 78.3 (C-5), 77.9 (C-3'), 74.7 (PhCH_2), 74.6 (PhCH_2), 74.5 (C-4), 71.9 (C-

2), 71.2 (C-5'), 70.0 (C-4'), 67.4 (Ph*C*H_2), 63.0 (C-2'), 61.7 (C-6'), 37.9 (Lev-*C*H_2), 29.9 (Lev-*C*H_3), 28.0 (Lev-*C*H_2), 26.9 (SiC(*C*H_3)$_3$), 21.3 (CH_3), 19.4 (Si*C*(CH_3)$_3$). **ESI-HRMS** for $C_{68}H_{75}O_{13}N_4SSi$ $(M+NH_4)^+$ calculated: 1215.4815; found: 1215.4800.

***p*-Tolyl benzyl (4-*O*-acetyl-2-azido-3-*O*-benzyl-6-*O*-*tert*-butyldiphenylsilyl-2-deoxy-α-D-glucopyranosyl)-(1→4)-(2-*O*-benzoyl-3-*O*-benzyl-1-thio-β-D-glucopyranosyluronate) 11a**

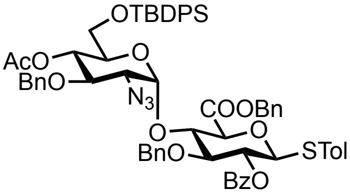

Following the general procedure A, hemiacetal donor **3b** (81.8 mg, 0.142 mmol), glucuronic acceptor **4a** (66.7 mg, 0.114 mmol), diphenyl sulfoxide (80.5 mg, 0.398 mmol), TTBP (106 mg, 0.426 mmol) and triflic anhydride (33 μL, 0.20 mmol) were used. Purification by column chromatography (100:0 to 99:1; CH_2Cl_2/Et_2O) gave **11a** as a white solid (60 mg, 46% yield). R_f = 0.65, CH_2Cl_2. $[\alpha]_D^{21}$ = +31.4 (c 0.5, $CHCl_3$). **^{1}H NMR** (400 MHz, $CDCl_3$): δ 8.14 – 8.04 (m, 2H, Ph), 7.69 – 7.55 (m, 5H, Ph), 7.47 (t, *J* = 7.8 Hz, 2H, Ph), 7.43 – 7.12 (m, 23H, Ph), 6.99 (d, *J* = 7.9 Hz, 2H, Ph), 5.46 (d, *J* = 3.7 Hz, 1H, H-1'), 5.35 (t, *J* = 9.5 Hz, 1H, H-4'), 5.29 (t, *J* = 9.2 Hz, 1H, H-2), 5.02 (d, *J* = 12.4 Hz, 1H, C*H*HPh), 4.94 (d, *J* = 12.4 Hz, 1H, C*H*HPh), 4.82 – 4.74 (m, 2H, C*H*HPh, H-1), 4.74 (d, *J* = 11.0 Hz, 1H, C*H*HPh), 4.67 (d, *J* = 10.3 Hz, 1H, C*H*HPh), 4.54 (d, *J* = 11.0 Hz, 1H, C*H*HPh), 4.21 (t, *J* = 8.9 Hz, 1H, H-4), 4.10 (d, *J* = 9.3 Hz, 1H, H-5), 3.99 (t, *J* = 8.5 Hz, 1H, H-3), 3.86 (t, *J* = 9.8 Hz, 1H, H-3'), 3.72 – 3.66 (m, 1H, H-6a'), 3.62 – 3.53 (m, 2H, H-5', H-6b'), 3.35 (dd, *J* = 10.3, 3.6 Hz, 1H, H-2'), 2.30 (s, 3H, CH_3), 1.86 (s, 3H, CH_3), 1.02 (s, 9H, SiC(CH_3)$_3$). **^{13}C{^{1}H} NMR** (101 MHz, $CDCl_3$): δ 169.0 (C=O), 167.5 (C=O), 165.1 (C=O), 138.7 (C), 137.6 (C), 137.3 (C), 135.90 (CH), 135.89 (CH), 134.9 (C), 134.1 (CH), 133.6 (CH), 133.4 (C), 133.2 (C), 130.0 (CH), 129.77 (CH), 129.75 (CH), 129.74 (CH), 128.72 (CH), 128.69 (CH), 128.62 (CH), 128.59 (CH), 128.5 (CH), 128.2 (CH), 128.10 (CH), 128.07 (CH), 128.02 (CH), 127.97 (CH), 127.8 (CH), 127.6 (C or CH), 97.7 (C-1', $^1J_{CH}$ = 178.0 Hz, from coupled HSQC), 86.6 (C-1, $^1J_{CH}$ = 155.6 Hz, from coupled HSQC), 83.8 (C-3), 78.4 (C-5), 78.1 (C-3'), 74.7 (Ph*C*H_2), 74.6 (Ph*C*H_2 or C-4), 74.5 (Ph*C*H_2 or C-4), 71.9 (C-2), 71.2 (C-5'), 69.7 (C-4'), 67.4 (Ph*C*H_2), 63.0 (C-2'), 61.7 (C-6'), 26.9 (SiC(*C*H_3)$_3$), 21.3 (CH_3), 21.0 (CH_3), 19.3 (Si*C*(CH_3)$_3$). **ESI-HRMS** for $C_{65}H_{71}O_{12}N_4SSi$ $(M+NH_4)^+$ calculated: 1159.4553; found: 1159.4549.

***p*-Tolyl benzyl (2-azido-3-*O*-benzyl-6-*O*-*tert*-butyldiphenylsilyl-2-deoxy-4-*O*-levulinoyl-α-D-glucopyranosyl)-(1→4)-(2-*O*-benzoyl-3-*O*-(*p*-methylbenzyl)-1-thio-β-D-glucopyranosyluronate) 10b**

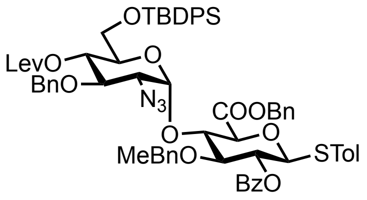

Following the general procedure A, hemiacetal donor **3a** (88 mg, 0.14 mmol), glucuronic acceptor **4b** (67.1 mg, 0.112 mmol), diphenyl sulfoxide (81 mg, 0.40 mmol), TTBP (106 mg, 0.426 mmol) and triflic anhydride (33 µL, 0.20 mmol) were used. Purification by column chromatography (100:0 to 99:1; CH_2Cl_2/Et_2O) gave **10b** and **6b** as an inseparable mixture as a cloudy syrup (68 mg, ^{1}H NMR spectrum of the syrup showed **10b**:**6b** = 57:43), calculated yield **10b** = 38% and **6b** = 30%. R_f = 0.68, 98:2; CH_2Cl_2/Et_2O. **^{1}H NMR** (400 MHz, $CDCl_3$) selected signals: δ 5.47 (d, *J* = 3.6 Hz, 1H, H-1'), 5.36 (t, *J* = 9.7 Hz, 1H, H-4'), 5.27 (t, *J* = 9.2 Hz, 1H, H-2), 5.03 (d, *J* = 12.4 Hz, 1H, C*H*HPh), 4.94 (d, *J* = 12.4 Hz, 1H, C*H*HPh), 4.76 (d, *J* = 9.8 Hz, 1H, H-1), 4.72 (d, *J* = 10.2 Hz, 1H, C*H*HPh), 4.72 (d, *J* = 11.0 Hz, 1H, C*H*HPh), 4.64 – 4.57 (m, 2H, 2 x C*H*HPh), 4.19 (t, *J* = 8.9 Hz, 1H, H-4), 4.11 – 4.06 (m, 1H, H-5), 3.97 (t, *J* = 8.5 Hz, 1H, H-3), 3.88 (t, *J* = 9.8 Hz, 1H, H-3'), 3.70 (dd, *J* = 12.1, 2.5 Hz, 1H, H-6a'), 3.64 – 3.54 (m, 2H, H-6b', H-5'), 3.32 (dd, *J* = 10.3, 3.7 Hz, 1H, H-2'), 2.60 (t, *J* = 6.6 Hz, 2H, Lev-CH_2), 2.36 (t, *J* = 6.7 Hz, 2H, Lev-CH_2), 2.30 (s, 3H, CH_3), 2.24 (s, 3H, CH_3), 2.12 (s, 3H, Lev-CH_3), 1.01 (s, 9H, $SiC(CH_3)_3$). **$^{13}C\{^1H\}$ NMR** (101 MHz, $CDCl_3$) selected signals: δ 206.1 (C=O), 171.0 (C=O), 167.5 (C=O), 165.1 (C=O), 138.7 (C), 137.8 (C), 137.7 (C), 97.6 (C-1', $^1J_{1CH}$ = 179.0 Hz, from coupled HSQC), 86.7 (C-1, $^1J_{1CH}$ = 157.0 Hz, from coupled HSQC), 83.8 (C-3), 78.4 (C-5), 77.9 (C-3'), 74.7 (Ph*C*H_2), 74.5 (Ph*C*H_2), 74.4 (C-4), 72.0 (C-2), 71.1 (C-5'), 70.0 (C-4'), 67.4 (Ph*C*H_2), 63.0 (C-2'), 61.7 (C-6'), 37.9 (Lev-*C*H_2), 29.9 (Lev-*C*H_3), 28.0 (Lev-*C*H_2), 26.9 (SiC(*C*H_3)$_3$), 21.3 (CH_3), 19.4 (Si*C*(CH_3)$_3$). **ESI-HRMS** for $C_{69}H_{74}O_{14}N_3SSi$ $(M+NH_4)^+$ calculated: 1229.4972; found: 1229.4964.

***p*-Tolyl benzyl (4-*O*-acetyl-2-azido-3-*O*-benzyl-6-*O*-*tert*-butyldiphenylsilyl-2-deoxy-α-D-glucopyranosyl)-(1→4)-(2-*O*-benzoyl-3-*O*-(*p*-methylbenzyl)-1-thio-β-D-glucopyranosyluronate) 11b**

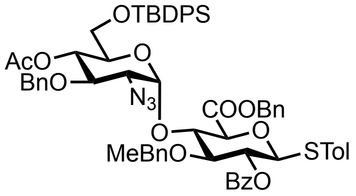

Following the general procedure A, hemiacetal donor **3b** (81.8 mg, 0.142 mmol), glucuronic acceptor **4b** (67.1 mg, 0.112 mmol), diphenyl sulfoxide (80.5 mg, 0.398 mmol), TTBP (106 mg, 0.426 mmol) and triflic anhydride (33 µL, 0.20 mmol) were used. Purification by column chromatography (CH_2Cl_2; R_f = 0.57) gave **11b** as a white solid (62 mg, 48% yield). R_f = 0.74, CH_2Cl_2. $[\alpha]_D^{21}$ = +38.3 (c 0.5, $CHCl_3$).

Scale up

Following the general procedure A, hemiacetal donor **3b** (450 mg, 0.782 mmol), glucuronic acceptor **4b** (312 mg, 0.521 mmol), diphenyl sulfoxide (443 mg, 2.19 mmol), TTBP (584 mg, 2.35 mmol) and triflic anhydride (0.18 mL, 1.1 mmol) were used. Purification by column chromatography (CH_2Cl_2; R_f = 0.57) gave **11b** as a white solid (234 mg, 39% yield).

^{1}H NMR (400 MHz, $CDCl_3$): δ 8.11 – 8.01 (m, 2H, Ph), 7.67 – 7.55 (m, 5H, Ph), 7.50 – 7.43 (m, 2H, Ph), 7.43 – 7.21 (m, 18H, Ph), 7.09 – 6.91 (m, 6H, Ph), 5.47 (d, *J* = 3.7 Hz, 1H, H-1'), 5.35 (t, *J* = 9.7 Hz, 1H, H-4'), 5.27 (t, *J* = 9.2 Hz, 1H, H-2), 5.02 (d, *J* = 12.4 Hz, 1H, C*H*HPh), 4.94 (d, *J* = 12.4 Hz, 1H, C*H*HPh), 4.79 – 4.70 (m, 3H, 2 x C*H*HPh, H-1), 4.62 (d, *J* = 10.1 Hz, 1H, C*H*HPh), 4.54 (d, *J* = 10.9 Hz, 1H, C*H*HPh), 4.22 – 4.17 (m, 1H, H-4), 4.09 (d, *J* = 9.4 Hz, 1H, H-5), 3.98 (t, *J* = 8.5 Hz, 1H, H-3), 3.86 (t, *J* = 9.8 Hz, 1H, H-3'), 3.72 – 3.64 (m, 1H, H-6a'), 3.62 – 3.50 (m, 2H, H-5', H-6b'), 3.35 (dd, *J* = 10.3, 3.7 Hz, 1H, H-2'), 2.30 (s, 3H, CH_3), 2.24 (s, 3H, CH_3), 1.86 (s, 3H, CH_3), 1.02 (s, 9H, $SiC(CH_3)_3$). **^{13}C{^{1}H} NMR** (101 MHz, $CDCl_3$): δ 169.0 (C=O), 167.6 (C=O), 165.1 (C=O), 138.7 (C), 137.7 (C), 137.6 (C), 135.91 (CH), 135.89 (CH), 134.94 (C), 134.28 (C), 134.1 (CH), 133.5 (CH), 133.4 (C), 133.2 (C), 130.0 (CH), 129.79 (CH), 129.77 (CH), 129.7 (CH), 129.2 (CH), 128.72 (CH), 128.66 (CH), 128.63 (CH), 128.58 (CH), 128.20 (CH), 128.17 (CH), 128.1 (CH), 127.8 (CH), 127.7 (C or CH), 97.6 (C-1', $^1J_{CH}$ = 177.4 Hz, from coupled HSQC), 86.7 (C-1, $^1J_{CH}$ = 156.6 Hz, from coupled HSQC), 83.7 (C-3), 78.3 (C-5), 78.1 (C-3'), 74.8 (Ph*C*H$_2$), 74.50 (Ph*C*H$_2$ or C-4), 74.47 (Ph*C*H$_2$ or C-4), 72.0 (C-2), 71.2 (C-5'), 69.7 (C-4'), 67.4 (Ph*C*H$_2$), 63.0 (C-2'), 61.7

(C-6'), 26.9 (SiC(CH_3)$_3$), 21.31 (CH_3), 21.26 (CH_3), 21.0 (CH_3), 19.3 (Si*C*(CH_3)$_3$). **ESI-HRMS** for $C_{66}H_{73}O_{12}N_4SSi$ $(M+NH_4)^+$ calculated: 1173.4709; found: 1173.4694.

***p*-Methoxyphenyl methyl (2-azido-3-*O*-benzyl-6-*O*-*tert*-butyldiphenylsilyl-2-deoxy-4-*O*-levulinoyl-α-D-glucopyranosyl)-(1→4)-(2-*O*-benzoyl-3-*O*-benzyl-β-D-glucopyranosyluronate) 15**

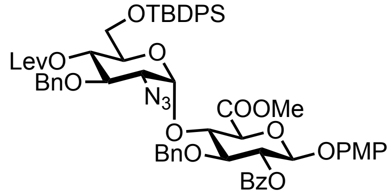

Following the general procedure B, hemiacetal donor **3a** (100 mg, 0.158 mmol), glucuronic acceptor **14** (161 mg, 0.316 mmol), diphenyl sulfoxide (89.4 mg, 0.442 mmol), TTBP (118 mg, 0.442 mmol) and triflic anhydride (37 μL, 0.22 mmol) were used. Purification by column chromatography (100:0 to 99:1; CH_2Cl_2/Et_2O) gave **15** as a white solid (79 mg, 45% yield).

Gram-scale

Following the general procedure B, hemiacetal donor **3a** (3.00 g, 4.75 mmol), glucuronic acceptor **14** (4.80 g, 9.44 mmol), diphenyl sulfoxide (2.69 g, 13.3 mmol), TTBP (3.55 g, 14.3 mmol) and triflic anhydride (1.1 mL, 6.7 mmol) were used. Purification by column chromatography (100:0 to 99:1; CH_2Cl_2/Et_2O) gave **15** as a white solid (3.59 g, 67% yield). R_f = 0.56, 98:2; CH_2Cl_2/Et_2O. $[\alpha]_D^{21}$ = +44.6 (c 0.5, $CHCl_3$). **^{1}H NMR** (400 MHz, $CDCl_3$): δ 8.10 – 8.01 (m, 2H, Ph), 7.71 – 7.60 (m, 4H, Ph), 7.61 – 7.53 (m, 1H, Ph), 7.49 – 7.17 (m, 18H, Ph), 6.95 – 6.81 (m, 2H, Ph), 6.78 – 6.64 (m, 2H, Ph), 5.58 (dd, *J* = 8.3, 7.0 Hz, 1H, H-2), 5.50 (d, *J* = 3.6 Hz, 1H, H-1'), 5.33 (t, *J* = 9.6 Hz, 1H, H-4'), 5.13 (d, *J* = 7.0 Hz, 1H, H-1), 4.82 (d, *J* = 10.6 Hz, 1H, C*H*HPh), 4.80 – 4.73 (m, 2H, 2 x C*H*HPh), 4.65 (d, *J* = 11.1 Hz, 1H, C*H*HPh), 4.39 (t, *J* = 8.6 Hz, 1H, H-4), 4.21 (d, *J* = 8.9 Hz, 1H, H-5), 4.08 (t, *J* = 8.3 Hz, 1H, H-3), 3.90 (dd, *J* = 10.4, 9.1 Hz, 1H, H-3'), 3.73 – 3.68 (m, 1H, H-6a'), 3.72 (s, 3H, OCH_3), 3.65 (dd, *J* = 11.6, 3.1 Hz, 1H, H-6b'), 3.60 (dt, *J* = 10.2, 2.7 Hz, 1H, H-5'), 3.51 (s, 3H, OCH_3), 3.35 (dd, *J* = 10.4, 3.6 Hz, 1H, H-2'), 2.62 (t, *J* = 6.7 Hz, 2H, Lev-CH_2), 2.41 – 2.33 (m, 2H, Lev-CH_2), 2.14 (s, 3H, Lev-CH_3), 1.03 (s, 9H, SiC(CH_3)$_3$). **^{13}C{^{1}H} NMR** (101 MHz, $CDCl_3$): δ 206.1 (C=O), 171.1 (C=O), 168.4 (C=O), 165.2 (C=O), 155.8 (C), 151.1 (C), 137.8 (C), 137.4 (C), 135.94 (CH), 135.88 (CH), 133.6 (CH), 133.5 (C), 133.3 (C), 129.9 (CH), 129.8 (CH), 129.6 (C), 128.7 (CH), 128.5 (CH), 128.2 (CH), 128.02 (CH), 127.98 (CH), 127.7 (CH), 118.9 (CH), 114.6 (CH), 100.7 (C-1), 97.7 (C-1'), 81.9 (C-3), 77.7 (C-3'), 74.9 (C-5), 74.6 (PhCH_2), 74.4

(C-4), 74.0 (Ph*C*H_2), 73.3 (C-2), 71.3 (C-5'), 70.1 (C-4'), 63.1 (C-2'), 61.8 (C-6'), 55.7 (OCH_3), 52.7 (OCH_3), 37.9 (Lev-*C*H_2), 29.9 (Lev-*C*H_3), 28.0 (Lev-*C*H_2), 26.9 (SiC(*C*H_3)$_3$), 19.4 (Si*C*(CH_3)$_3$). **ESI-HRMS** for $C_{62}H_{71}O_{15}N_4Si$ $(M+NH_4)^+$ calculated: 1139.4680; found: 1139.4711.

Methyl (2-azido-3-*O*-benzyl-6-*O*-*tert*-butyldiphenylsilyl-2-deoxy-4-*O*-levulinoyl-α-D-glucopyranosyl)-(1→4)-(3-*O*-benzyl-D-glucuronal) 30

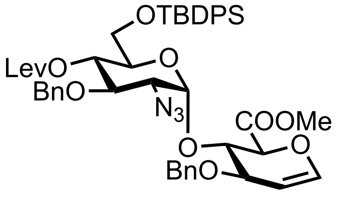

Following the general procedure B, hemiacetal donor **3a** (1.21 g, 1.92 mmol), glucuronic acceptor **29** (1.01 g, 3.84 mmol), diphenyl sulfoxide (1.09 g, 5.38 mmol), TTBP (1.63 g, 5.76 mmol) and triflic anhydride (0.40 mL, 2.7 mmol) were used. Purification by column chromatography (100:0 to 99:1; CH_2Cl_2/Et_2O) gave **30** as a white solid (1.01 g, 60% yield). R_f = 0.24, 99:1; CH_2Cl_2/Et_2O. $[\alpha]_D^{21}$ = +39.0 (c 0.5, $CHCl_3$). **^{1}H NMR** (400 MHz, $CDCl_3$): δ 7.73 – 7.62 (m, 4H, Ph), 7.46 – 7.24 (m, 16H, Ph), 6.67 (d, *J* = 6.3 Hz, 1H, H-1), 5.18 (d, *J* = 3.6 Hz, 1H, H-1'), 5.12 – 5.03 (m, 2H, H-4', H-2), 4.99 (dd, *J* = 2.6, 1.4 Hz, 1H, H-5), 4.77 (d, *J* = 11.1 Hz, 1H, C*H*HPh), 4.65 (d, *J* = 11.1 Hz, 1H, C*H*HPh), 4.56 (d, *J* = 11.2 Hz, 1H, C*H*HPh), 4.46 (d, *J* = 11.2 Hz, 1H, C*H*HPh), 4.46 – 4.40 (m, 1H, H-4), 4.00 – 3.88 (m, 3H, H-5', H-3', H-3), 3.68 (dd, *J* = 11.5, 5.3 Hz, 1H, H-6a'), 3.62 (dd, *J* = 11.5, 2.3 Hz, 1H, H-6b'), 3.46 (s, 3H, OCH_3), 3.32 (dd, *J* = 10.4, 3.6 Hz, 1H, H-2'), 2.55 (t, *J* = 6.7 Hz, 2H, Lev-CH_2), 2.34 (dd, *J* = 6.9, 5.8 Hz, 2H, Lev-CH_2), 2.10 (s, 3H, Lev-CH_3), 1.02 (s, 9H, SiC(CH_3)$_3$). **^{13}C{^{1}H} NMR** (101 MHz, $CDCl_3$): δ 206.0 (C=O), 171.4 (C=O), 168.0 (C=O), 145.6 (C-1), 137.8 (C), 135.9 (CH), 135.8 (CH), 133.3 (C), 129.8 (CH), 129.7 (CH), 128.53 (C), 128.47 (C), 128.2 (CH), 128.0 (CH), 127.92 (CH), 127.91 (CH), 127.8 (CH), 127.7 (CH), 99.4 (C-1'), 98.5 (C-2), 77.4 (C-3'), 74.7 (Ph*C*H_2), 74.6 (C-4), 73.6 (C-5), 71.8 (C-5'), 70.6 (C-4'), 69.7 (Ph*C*H_2), 67.5 (C-3), 62.83 (C-2'), 62.77 (C-6'), 52.1 (OCH_3), 37.9 (Lev-*C*H_2), 29.9 (Lev-*C*H_3), 28.0 (Lev-*C*H_2), 26.8 (SiC(*C*H_3)$_3$), 19.3 (Si*C*(CH_3)$_3$). **ESI-HRMS** for $C_{49}H_{63}O_{12}N_4Si$ $(M+CH_3OH+NH_4)^+$ calculated: 927.4206; found: 927.4223.

Donor side products

Table S1. 1,6-anhydro 12 diagnostic ^{1}H and ^{13}C NMR chemical shifts (in ppm, 400 MHz, $CDCl_3$)

	H-1	H-2	H-3	H-6a	H-6b	C-1	C-2	C-3	C-6
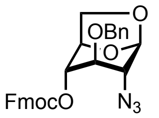	5.55	3.30	3.69	4.16	3.79	100.9	60.3	76.3	65.6
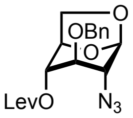	5.52[18]	3.24	3.62	4.17	3.76	100.8	60.1	76.2	65.5
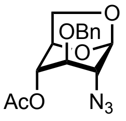	5.52[19]	3.24	3.60	4.20	3.78	100.8	59.9	76.2	65.5

Table S2. Glycal 13 diagnostic ^{1}H and ^{13}C NMR chemical shifts (in ppm, 400 MHz, $CDCl_3$)

	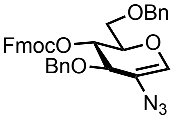	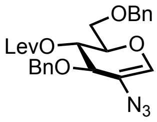	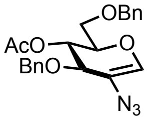	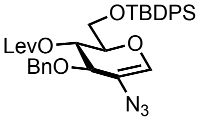	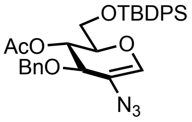
**H-1**	6.49	6.45	6.46	6.29	6.30
**C-1**		134.9	135.0	135.0	135.2

2-Azido-3-*O*-benzyl-6-*O*-*tert*-butyldiphenylsilyl-2-deoxy-4-*O*-levulinoyl-D-glucal 13a

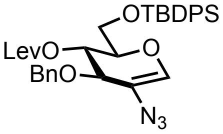

*R*f = 0.78, 99:1; ; CH_2Cl_2/Et_2O. **^{1}H NMR** (400 MHz, $CDCl_3$) selected signals; δ 6.28 (s, 1H, H-1), 5.49 (t, *J* = 2.4 Hz, 1H, H-4), 4.77 (d, *J* = 11.0 Hz, 1H, C*H*HPh), 4.59 (d, *J* = 11.0 Hz, 1H, C*H*HPh), 4.27 (dddd, *J* = 7.0, 6.1, 2.5, 1.7 Hz, 1H, H-5), 3.95 – 3.83 (m, 3H, H-3,H-6a, H-6b), 2.77 (dd, *J* = 7.2, 5.6 Hz, 2H, Lev-CH_2), 2.60 (dd, *J* = 6.9, 5.6 Hz, 2H, Lev-CH_2), 2.20 (s, 3H, Lev-CH_3), 1.04 (s, 9H, $SiC(CH_3)_3$). **^{13}C{^{1}H} NMR** (101 MHz, $CDCl_3$) selected signals: δ 206.3 (C=O), 171.8 (C=O), 135.0 (C-1), 114.7 (C-2), 76.5 (C-5), 71.6 (Ph*C*H_2), 70.9 (C-3), 66.7 (C-4), 61.0 (C-6), 38.0 (Lev-*C*H_2), 29.9 (Lev-*C*H_3), 28.2 (Lev-*C*H_2), 27.0 (SiC(*C*H_3)$_3$), 19.3 (Si*C*(CH_3)$_3$). **ESI-HRMS** for $C_{34}H_{45}O_7N_4Si$ $(M+H_2O+NH_4)^+$ calculated: 649.3052; found: 649.3041.

Acceptor side products

The following side-products were isolated in quantities enough for NMR characterisation however an accurate yield could not be obtained due to difficulties encountered during purification.

Benzyl (1,2,4-*O*-orthobenzoyl-3-*O*-(*p*-methylbenzyl)-α-D-glucopyranosyluronate) 6b

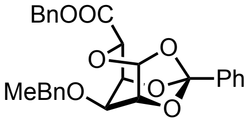

R_f = 0.57, 97:3; CH_2Cl_2/Et_2O. $[\alpha]_D^{22}$ = +70.9 (c 0.25, $CHCl_3$). **^{1}H NMR** (400 MHz, $CDCl_3$): δ 7.66 – 7.55 (m, 2H, Ph), 7.43 – 7.26 (m, 8H, Ph), 7.17 – 7.06 (m, 4H, Ph), 6.03 (d, *J* = 4.9 Hz, 1H, H-1), 5.22 (d, *J* = 12.1 Hz, 1H, C*H*HPh), 5.11 – 5.04 (m, 2H, C*H*HPh, H-5), 5.02 (dd, *J* = 4.8, 2.1 Hz, 1H, H-4), 4.59 (dt, *J* = 4.5, 2.1 Hz, 1H, H-2), 4.40 (s, 2H, 2 x C*H*HPh), 4.07 (dd, *J* = 4.8, 2.1 Hz, 1H, H-3), 2.32 (s, 3H, CH_3). **^{13}C{^{1}H} NMR** (101 MHz, $CDCl_3$): δ 168.6 (C=O), 138.0 (C), 135.4 (C), 134.2 (C), 133.4 (C), 130.2 (CH), 129.4 (CH), 128.74 (CH), 128.67 (CH), 128.6 (CH), 128.3 (CH), 127.9 (CH), 126.1 (CH), 118.2 (C), 97.7 (C-1, $^1J_{1CH}$ = 186.4 Hz, from coupled HSQC), 75.7 (C-5) , 73.5 (C-4), 72.9 (C-2), 72.0 (Ph*C*H_2), 69.2 (C-3), 67.4 (Ph*C*H_2), 21.3 (CH_3). **ESI-HRMS** for $C_{28}H_{27}O_7$ $(M+H)^+$ calculated: 475.1751; found: 475.1747.

Benzyl (1,4-anhydro-2-*O*-benzoyl-3-*O*-benzyl-β-D-glucopyranosyluronate) 7a

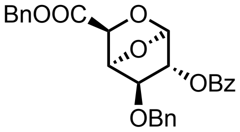

R_f = 0.58, 97:3; CH_2Cl_2/Et_2O. $[\alpha]_D^{22}$ = -2.7 (c 0.2, $CHCl_3$). ^{1}H NMR (400 MHz, $CDCl_3$): δ 8.05 – 7.99 (m, 2H, Ph), 7.61 – 7.55 (m, 1H, Ph), 7.49 – 7.40 (m, 2H, Ph), 7.35 – 7.24 (m, 8H, Ph), 7.24 – 7.15 (m, 2H, Ph), 5.71 (app d, *J* = 1.1 Hz, 1H, H-1), 5.19 (d, *J* = 2.0 Hz, 1H, H-2), 5.10 (dd, *J* = 4.7, 3.5 Hz, 1H, H-4), 4.91 (d, *J* = 12.2 Hz, 1H, C*H*HPh), 4.74 (d, *J* = 12.2 Hz, 1H, C*H*HPh), 4.58 (d, *J* = 11.3 Hz, 1H, C*H*HPh), 4.46 (d, *J* = 3.6 Hz, 1H, H-5), 4.43 (d, *J* = 11.3 Hz, 1H, C*H*HPh),, 4.16 (ddt, *J* = 4.7, 1.9, 0.9 Hz, 1H, H-3). **^{13}C{^{1}H} NMR** (101 MHz, $CDCl_3$): δ 168.0 (C=O), 165.4 (C=O), 136.8 (C), 135.4 (C), 133.6 (CH), 130.0 (CH), 129.4 (C), 128.63 (CH), 128.60 (CH), 128.59 (CH), 128.53 (CH), 128.48 (CH), 128.4 (CH), 128.3 (CH), 104.5 (C-1), 83.6 (C-3), 78.3 (C-2), 77.4 (C-4), 75.0 (C-5), 73.5 (Ph*C*H_2), 67.0 (Ph*C*H_2). **ESI-HRMS** for $C_{27}H_{28}O_7N$ $(M+NH_4)^+$ calculated: 478.1860; found: 478.1858.

Benzyl (1,4-anhydro-2-*O*-benzoyl-3-*O*-(*p*-methylbenzyl)-β-D-glucopyranosyluronate) 7b

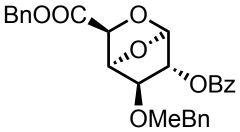

R_f = 0.51, 97:3; CH_2Cl_2/Et_2O. $[\alpha]_D^{22}$ = -9.9 (c 0.25, $CHCl_3$). **^{1}H NMR** (400 MHz, $CDCl_3$): δ 8.04 – 7.98 (m, 2H, Ph), 7.63 – 7.54 (m, 1H, Ph), 7.45 (t, *J* = 7.7 Hz, 2H, Ph), 7.36 – 7.27 (m, 3H), 7.26 – 7.19 (m, 2H, Ph), 7.15 (d, *J* = 8.1 Hz, 2H, Ph), 7.07 (d, *J* = 7.9 Hz, 2H, Ph), 5.70 (s, 1H, H-1), 5.17 (d, *J* = 2.0 Hz, 1H, H-2), 5.08 (t, *J* = 4.2 Hz, 1H, H-4), 4.93 (d, *J* = 12.2 Hz, 1H, C*H*HPh), 4.78 (d, *J* = 12.2 Hz, 1H, C*H*HPh), 4.52 (d, *J* = 11.2 Hz, 1H, C*H*HPh), 4.45 (d, *J* = 3.5 Hz, 1H, H-5), 4.39 (d, *J* = 11.2 Hz, 1H, C*H*HPh), 4.14 (ddt, *J* = 4.9, 2.0, 1.0 Hz, 1H, H-3), 2.27 (s, 3H,CH_3). **$^{13}C\{^1H\}$ NMR** (101 MHz, $CDCl_3$): δ 168.1 (C=O), 165.4 (C=O), 138.0 (C), 135.5 (C), 133.8 (C), 133.6 (CH), 130.0 (CH), 129.2 (CH), 128.7 (CH), 128.6 (CH), 128.4 (C), 104.5 (C-1, $^1J_{1CH}$ = 189.9 Hz, from coupled HSQC), 83.3 (C-3), 78.3 (C-2), 77.4 (C-4), 75.0 (C-5), 73.3 (PhCH_2), 67.0 (PhCH_2), 21.3 (CH_3). **ESI-HRMS** for $C_{28}H_{27}O_7$ $(M+H)^+$ calculated: 475.1751; found: 475.1749.

Transformations of Disaccharide Building Block with GlcA Reducing End

Methyl (2-azido-3-*O*-benzyl-6-*O*-*tert*-butyldiphenylsilyl-2-deoxy-4-*O*-levulinoyl-α-D-glucopyranosyl)-(1→4)-(2-*O*-benzoyl-3-*O*-benzyl-α/β-D-glucopyranosyluronate) 16

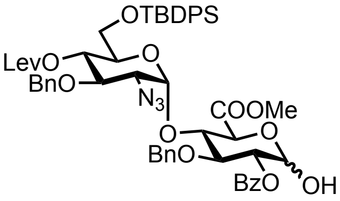

A solution of **15** (2.32 g, 2.07 mmol) in Tol/MeCN/H_2O (1:1.5:1; 41 mL) was treated with CAN (5.40 g, 9.85 mmol) and stirred at room temperature. After 30 minutes the reaction was diluted with EtOAc (75 mL) and washed with H_2O (175 mL). The layers were separated and the product was extracted with EtOAc (3 x 50 mL). The combined organic layers were dried over anhydrous $MgSO_4$, filtered and concentrated *in vacuo* to give a brown paste. Purification

by column chromatography (97:3 to 90:10; ; CH_2Cl_2/Et_2O) gave **16** as a orangish foam (1.60 g, 76% yield, α/β = 80:20). R_f = 0.28, 95:5; CH_2Cl_2/Et_2O.

The following were observed for α and β anomers: **^{1}H NMR** (400 MHz, $CDCl_3$): δ 8.15 – 8.02 (m, 2H, Ph), 7.71 – 7.62 (m, 4H, Ph), 7.60 – 7.53 (m, 1H, Ph), 7.49 – 7.43 (m, 2H, Ph), 7.42 – 7.18 (m, 16H, Ph), 2.61 (t, *J* = 6.7 Hz, 2H, Lev-CH_2), 2.47 – 2.33 (m, 2H, Lev-CH_2), 2.14 (s, 3H, Lev-CH_3), 1.02 (s, 9H, $SiC(CH_3)_3$).

α-anomer

^{1}H NMR (400 MHz, $CDCl_3$): δ 5.65 (dd, *J* = 5.9, 3.1 Hz, 1H, H-1), 5.38 (d, *J* = 3.6 Hz, 1H, H-1'), 5.29 – 5.21 (m, 1H, H-4'), 5.15 (ddd, *J* = 8.0, 3.1, 0.9 Hz, 1H, H-2), 4.85 (s, 2H, 2 x C*H*HPh), 4.70 (d, *J* = 7.5 Hz, 1H, H-5), 4.54 (d, *J* = 11.0 Hz, 1H, C*H*HPh), 4.44 (d, *J* = 11.0 Hz, 1H, C*H*HPh), 4.36 (dd, *J* = 8.0, 7.0 Hz, 1H, H-3), 4.19 (t, *J* = 7.3 Hz, 1H, H-4), 3.81 (dd, *J* = 10.3, 9.2 Hz, 1H, H-3'), 3.73 – 3.60 (m, 3H, H-5', H-6a', H-6b'), 3.54 (s, 3H, OCH_3), 3.31 (dd, *J* = 10.3, 3.6 Hz, 1H, H-2'), 3.14 (dd, *J* = 5.8, 0.9 Hz, 1H, OH). **^{13}C{^{1}H} NMR** (101 MHz, $CDCl_3$) selected signals: δ 206.1 (C=O), 171.1 (C=O), 169.4 (C=O), 165.8 (C=O), 137.8 (C), 137.6 (C), 136.0 (CH), 135.9 (CH), 98.6 (C-1'), 90.1 (C-1), 77.8 (C-3 and C-3'), 75.3 (C-4), 74.65 (Ph*C*H_2), 74.5 (Ph*C*H_2), 72.5 (C-2), 71.9 (C-5), 71.3 (C-5'), 70.3 (C-4'), 63.1 (C-2'), 62.0 (C-6'), 52.5 (OCH_3), 37.9 (Lev-*C*H_2), 29.9 (Lev-*C*H_3), 28.0 (Lev-*C*H_2), 26.9 (SiC(*C*H_3)$_3$), 19.40 (Si*C*(CH_3)$_3$).

β-anomer

^{1}H NMR (400 MHz, $CDCl_3$) selected signals: δ 5.41 (d, *J* = 3.6 Hz, 1H, H-1'), 5.21 (dd, *J* = 8.3, 6.9 Hz, 1H, H-2), 4.93 (dd, *J* = 9.4, 6.8 Hz, 1H, H-1), 4.83 (s, 2H, 2 x C*H*HPh), 4.56 (d, *J* = 11.0 Hz, 1H, C*H*HPh), 4.30 – 4.22 (m, 2H, H-5, H-4), 4.15 – 4.05 (m, 1H, H-3), 3.97 (d, *J* = 9.4 Hz, 1H, OH), 3.86 (dd, *J* = 10.4, 9.3 Hz, 1H, H-3'), 3.56 (s, 3H, OCH_3), 3.38 – 3.30 (m, 1H, H-2'). **^{13}C{^{1}H} NMR** (101 MHz, $CDCl_3$) selected signals: δ 169.3 (C=O), 166.7 (C=O), 98.1 (C-1'), 95.8 (C-1), 80.6 (C-3), 52.8 (OCH_3), 19.43 (Si*C*(CH_3)$_3$).

ESI-HRMS for $C_{55}H_{61}O_{14}N_3SiNa$ $(M+Na)^+$ calculated: 1038.3815; found: 1038.3812.

Methyl (2-azido-3-*O*-benzyl-6-*O*-*tert*-butyldiphenylsilyl-2-deoxy-4-*O*-levulinoyl-α-D-glucopyranosyl)-(1→4)-(2-*O*-benzoyl-3-*O*-benzyl-1-*O*-trichloroacetimidoyl-α/β-D-glucopyranosyluronate) 17

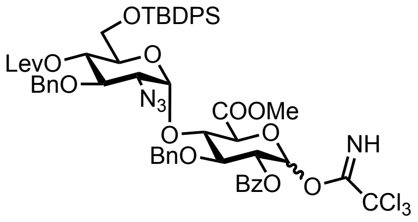

Under a N_2 atmosphere, a solution of **16** (652 mg, 0.642 mmol) and K_2CO_3 (267 mg, 1.93 mmol) in anhydrous CH_2Cl_2 (2.1 mL) was treated with CCl_3CN (0.32 mL, 3.2 mmol) and stirred at room temperature. After 15 h the reaction mixture was concentrated to half the volume and purified by column chromatography (98:2; CH_2Cl_2/Et_2O with 0.5% Et_3N) to give **17** as a white paste (712 mg, 95%, α/β = 35:65). R_f = 0.74, 97:3; CH_2Cl_2/Et_2O.

The following were observed for α and β anomers: **^{1}H NMR** (400 MHz, $CDCl_3$): δ 8.04 – 7.98 (m, 2H, Ph), 7.68 – 7.60 (m, 4H, Ph), 7.61 – 7.52 (m, 1H, Ph), 7.47 – 7.26 (m, 13H, Ph), 7.24 – 7.12 (m, 5H, Ph), 3.72 – 3.60 (m, 2H, H-6a', H-6b'), 2.62 (t, *J* = 7.0 Hz, 2H, Lev-CH_2), 2.38 (td, *J* = 6.6, 1.9 Hz, 2H, Lev-CH_2), 1.03 (s, 9H, $SiC(CH_3)_3$). **^{13}C{^{1}H} NMR** (101 MHz, $CDCl_3$): δ 206.1 (C=O), 171.1 (C=O), 129.9 (CH), 129.8 (CH), 129.7 (CH), 128.7 (CH), 128.58 (CH), 128.55 (CH), 128.1 (CH), 128.01 (CH), 127.96 (CH), 127.8 (CH), 127.72 (CH), 127.71 (CH), 61.8 (C-6'), 38.0 (Lev-$\mathit{C}H_2$), 29.9 (Lev-$\mathit{C}H_3$), 28.0 (Lev-$\mathit{C}H_2$), 26.9 ($SiC(\mathit{C}H_3)_3$), 19.4 ($Si\mathit{C}(CH_3)_3$).

α-anomer

^{1}H NMR (400 MHz, $CDCl_3$): δ 8.61 (s, 1H, NH), 6.65 (d, *J* = 3.5 Hz, 1H, H-1), 5.55 (d, *J* = 3.7 Hz, 1H, H-1'), 5.44 (dd, *J* = 9.5, 3.5 Hz, 1H, H-2), 5.34 (dd, *J* = 10.1, 9.2 Hz, 1H, H-4'), 4.92 (d, *J* = 10.7 Hz, 1H, C*H*HPh), 4.86 (d, *J* = 10.7 Hz, 1H, C*H*HPh), 4.78 (d, *J* = 11.1 Hz, 1H, C*H*HPh), 4.64 (d, *J* = 11.1 Hz, 1H, C*H*HPh), 4.53 (d, *J* = 9.6 Hz, 1H, H-5), 4.40 (dd, *J* = 9.5, 8.6 Hz, 1H, H-3), 4.24 (dd, *J* = 9.6, 8.6 Hz, 1H, H-4), 3.93 (dd, *J* = 10.4, 9.2 Hz, 1H, H-3'), 3.61 – 3.54 (m, 1H, H-5'), 3.56 (s, 3H, OCH_3), 3.31 (dd, *J* = 10.4, 3.7 Hz, 1H, H-2'), 2.13 (s, 3H, Lev-CH_3). **^{13}C{^{1}H} NMR** (101 MHz, $CDCl_3$): δ 168.4 (C=O), 165.4 (C=O), 160.5 (C=NH), 137.9 (C), 137.5 (C), 135.95 (CH), 135.90 (CH), 133.73 (CH), 133.5 (C), 133.4 (C), 129.1 (C), 98.2 (C-1'), 93.4 (C-1), 90.8 (CCl_3), 79.5 (C-3), 77.6 (C-3'), 75.1 (Ph$\mathit{C}H_2$), 74.66 (Ph$\mathit{C}H_2$), 74.32 (C-4), 73.0 (C-5), 72.2 (C-2), 71.2 (C-5'), 70.1 (C-4'), 62.9 (C-2'), 52.81 (OCH_3).

β-anomer

^{1}H NMR (400 MHz, $CDCl_3$): δ 8.65 (s, 1H, NH), 6.10 (d, *J* = 6.5 Hz, 1H, H-1), 5.61 (t, *J* = 6.8 Hz, 1H, H-2), 5.45 (d, *J* = 3.6 Hz, 1H, H-1'), 5.35 (t, *J* = 9.6 Hz, 1H, H-4'), 4.79 (s, 2H, 2 x C*H*HPh), 4.73 (d, *J* = 11.1 Hz, 1H, C*H*HPh), 4.61 (d, *J* = 11.1 Hz, 1H, C*H*HPh), 4.44 (dd, *J* = 8.7, 7.3 Hz, 1H, H-4), 4.37 (d, *J* = 8.7 Hz, 1H, H-5), 4.11 (t, *J* = 7.3 Hz, 1H, H-3), 3.88 (dd, *J* = 10.4, 9.4 Hz, 1H, H-3'), 3.66 – 3.60 (m, 1H, H-5'), 3.57 (s, 3H, OCH_3), 3.35 (dd, *J* = 10.4, 3.6 Hz, 1H, H-2'), 2.14 (s, 3H, Lev-CH_3). **^{13}C{^{1}H} NMR** (101 MHz, $CDCl_3$) δ 168.3 (C=O), 164.9 (C=O), 161.2 (C=NH), 137.8 (C), 137.3 (C), 135.92 (CH), 135.87 (CH), 133.68 (CH), 133.6 (C), 133.3 (C), 129.4 (C), 97.9 (C-1'), 95.7 (C-1), 90.5 (CCl_3), 81.3 (C-3), 77.7 (C-3'), 75.0 (C-5), 74.68 (Ph*C*H_2), 74.26 (C-4), 73.7 (Ph*C*H_2), 71.6 (C-2), 71.3 (C-5'), 70.0 (C-4'), 63.1 (C-2'), 52.79 (OCH_3).

ESI-HRMS for $C_{57}H_{65}O_{14}N_5SiCl_3$ $(M+NH_4)^+$ calculated: 1176.3357; found: 1176.3344.

_N_-Benzyloxycarbonyl-3-aminopropyl methyl (2-azido-3-_O_-benzyl-6-_O_-_tert_-butyldiphenylsilyl-2-deoxy-4-_O_-levulinoyl-α-D-glucopyranosyl)-(1→4)-(2-_O_-benzoyl-3-_O_-benzyl-β-D-glucopyranosyluronate) 18

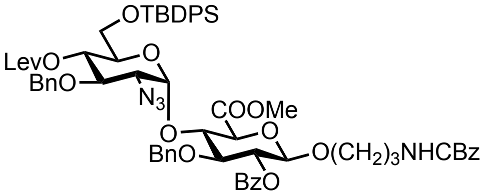

In a two-neck RBF, imidate **17** (350 mg, 0.302 mmol), 3-amino-*N*-benzyloxycarbonylpropanol (76 mg, 0.362 mmol) and freshly activated 4Å molecular sieves, were placed under three cycles of vacuum and N_2. The solids were dissolved in anhydrous CH_2Cl_2 (6 mL) and pre-dried for 0.5-1 h. The solution was cooled down to -15 °C (using ice and NaCl) and then treated with TMSOTf (11 μL, 60 μmol). The reaction was allowed to gradually warm up to room temperature over a period of 3 h. The reaction was then quenched with Et_3N, diluted with CH_2Cl_2 (20 mL) and washed with H_2O (25 mL). The layers were separated and the product was extracted with CH_2Cl_2 (2 x 20 mL). The combined organic layers were dried over anhydrous $MgSO_4$, filtered and concentrated *in vacuo* to give an off-white foam. Purification by column chromatography (97:3 to 96:4; CH_2Cl_2/Et_2O) gave **18** as a white solid (254 mg, 70% yield, β-only). R_f = 0.35, 97:3; CH_2Cl_2/Et_2O. $[\alpha]_D^{21}$ = +39.7 (c 0.5, $CHCl_3$). **^{1}H NMR** (400 MHz, $CDCl_3$): δ 8.05 – 7.99 (m, 2H, Ph), 7.69 – 7.62 (m, 4H, Ph), 7.59 – 7.51 (m, 1H, Ph), 7.50 – 7.27 (m, 18H, Ph), 7.24 – 7.12 (m, 5H, Ph), 5.46 (d, *J* = 3.6 Hz, 1H, H-1'), 5.37 – 5.29 (m, 2H, H-2, H-4'), 5.07 (t, *J* = 6.0 Hz, 1H, NH), 5.05 (s, 2H, CBz-CH_2), 4.81 – 4.71 (m, 3H,

3 x C*H*HPh), 4.65 (d, *J* = 7.0 Hz, 1H, H-1), 4.64 (d, *J* = 11.1 Hz, 1H, C*H*HPh), 4.27 (dd, *J* = 9.2, 8.2 Hz, 1H, H-4), 4.11 (d, *J* = 9.2 Hz, 1H, H-5), 4.01 (t, *J* = 8.2 Hz, 1H, H-3), 3.94 – 3.86 (m, 1H, Linker-OC*H*H), 3.89 (dd, *J* = 10.3, 9.2 Hz, 1H, H-3'), 3.68 (dd, *J* = 11.6, 2.2 Hz, 1H, H-6a'), 3.63 (dd, *J* = 11.6, 3.0 Hz, 1H, H-6b'), 3.60 – 3.46 (m, 2H, H-5', Linker-OC*H*H), 3.54 (s, 3H, OCH_3), 3.32 (dd, *J* = 10.3, 3.6 Hz, 1H, H-2'), 3.24 – 3.05 (m, 2H, Linker-NCH_2), 2.62 (t, *J* = 6.7 Hz, 2H, Lev-CH_2), 2.37 (t, *J* = 6.4 Hz, 2H, Lev-CH_2), 2.14 (s, 3H, Lev-CH_3), 1.77 – 1.64 (m, 2H, Linker-CH_2), 1.02 (s, 9H, $SiC(CH_3)_3$). **$^{13}C\{^1H\}$ NMR** (101 MHz, $CDCl_3$): δ 206.2 (C=O), 171.1 (C=O), 168.8 (C=O), 165.3 (C=O), 156.6 (CBz-C=O), 137.8 (C), 137.4 (C), 137.0 (C), 135.93 (CH), 135.88 (CH), 133.6 (CH), 133.5 (C), 133.3 (C), 129.9 (CH), 129.8 (CH), 129.7 (CH), 129.6 (C), 128.7 (CH), 128.58 (CH), 128.55 (CH), 128.53 (CH), 128.2 (CH), 128.12 (CH), 128.09 (CH), 128.0 (CH), 127.73 (CH), 127.71 (CH), 100.9 (C-1), 97.5 (C-1'), 82.1 (C-3), 77.7 (C-3'), 74.6 (Ph*C*H_2, C-5), 74.2 (C-4), 73.9 (Ph*C*H_2), 73.3 (C-2), 71.2 (C-5'), 70.0 (C-4'), 67.3 (Linker-O*C*H_2), 66.5 (CBz-*C*H_2), 63.0 (C-2'), 61.8 (C-6'), 52.7 (OCH_3), 38.1 (Linker-N*C*H_2), 37.9 (Lev-*C*H_3), 29.9 (Lev-*C*H_3), 29.5 (Linker-*C*H_2), 28.0 (Lev-*C*H_2), 26.9 (SiC(*C*H_3)$_3$), 19.4 (Si*C*(CH_3)$_3$). **ESI-HRMS** for $C_{66}H_{78}O_{16}N_5Si^+$ $(M+NH_4)^+$ calculated: 1224.5207; found: 1224.5217.

***N*-Benzyloxycarbonyl-3-aminopropyl methyl (2-azido-3-*O*-benzyl-6-*O*-*tert*-butyldiphenylsilyl-2-deoxy-α-D-glucopyranosyl)-(1→4)-(2-*O*-benzoyl-3-*O*-benzyl-β-D-glucopyranosyluronate) 19**

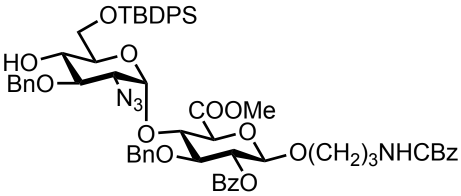

A solution of **18** (230 mg, 0.190 mmol) and N_2H_4.AcOH (87 mg, 0.95 mmol) in bench CH_2Cl_2/MeOH (1:1; 1.9 mL) was stirred at room temperature for 4 h. The reaction was diluted with CH_2Cl_2 (10 mL) and washed with H_2O (25 mL). The layers were separated and the product was extracted with CH_2Cl_2 (3 x 10 mL). The combined organic layers were dried over anhydrous $MgSO_4$, filtered and concentrated *in vacuo* to give a syrup. Purification by column chromatography (90:10; CH_2Cl_2/Et_2O) gave **19** as a white solid (185 mg, 88% yield). R_f = 0.21, 1:1; petroleum ether/Et_2O. $[\alpha]_D^{21}$ = +39.3 (c 0.5, $CHCl_3$). **^{1}H NMR** (400 MHz, $CDCl_3$): δ 8.05 – 7.97 (m, 2H, PH), 7.70 – 7.59 (m, 4H, Ph), 7.59 – 7.51 (m, 1H, Ph), 7.47 – 7.27 (m, 18H, Ph), 7.22 – 7.12 (m, 5H, Ph), 5.39 (d, *J* = 3.7 Hz, 1H, H-1'), 5.31 (dd, *J* = 8.2, 7.0 Hz, 1H, H-

2), 5.11 – 5.01 (m, 1H, NH), 5.04 (s, 2H, CBz-CH_2), 4.90 (d, *J* = 11.0 Hz, 1H, C*H*HPh), 4.85 (d, *J* = 11.0 Hz, 1H, C*H*HPh), 4.78 (d, *J* = 10.7 Hz, 1H, C*H*HPh), 4.70 (d, *J* = 10.7 Hz, 1H, C*H*HPh), 4.64 (d, *J* = 7.0 Hz, 1H, H-1), 4.25 (dd, *J* = 9.3, 8.4 Hz, 1H, H-4), 4.07 (d, *J* = 9.3 Hz, 1H, H-5), 3.97 (t, *J* = 8.3 Hz, 1H, H-3), 3.95 – 3.86 (m, 1H, Linker-OC*H*H), 3.91 (dd, *J* = 10.7, 3.6 Hz, 1H, H-6a'), 3.82 – 3.70 (m, 3H, H-4', H-3', H-6b'), 3.61 (s, 3H, OCH_3), 3.53 (p, *J* = 5.6 Hz, 1H, Linker-OC*H*H), 3.45 (ddd, *J* = 8.9, 4.9, 3.2 Hz, 1H, H-5'), 3.25 – 3.07 (m, 2H, Linker-NCH_2), 3.20 (dd, *J* = 9.7, 3.7 Hz, 1H, H-2'), 2.76 (d, *J* = 2.2 Hz, 1H, OH), 1.78 – 1.60 (m, 2H, Linker-CH_2), 1.06 (s, 9H, $SiC(CH_3)_3$). **$^{13}C\{^1H\}$ NMR** (101 MHz, $CDCl_3$): δ 168.8 (C=O), 165.2 (C=O), 156.6 (CBz-C=O), 138.3 (C), 137.4 (C), 137.0 (C), 135.77 (CH), 135.76 (CH), 133.6 (CH), 132.9 (C), 132.8 (C), 130.12 (CH), 130.10 (CH), 129.9 (CH), 129.6 (C), 128.70 (CH), 128.68 (CH), 128.6 (CH), 128.5 (CH), 128.3 (CH), 128.13 (CH), 128.09 (CH), 128.04 (CH), 128.01 (CH), 127.98 (CH), 127.9 (CH), 101.0 (C-1), 97.6 (C-1'), 82.1 (C-3), 79.3 (C-3'), 75.3 (Ph*C*H_2), 74.6 (C-5), 74.4 (C-4), 74.3 (Ph*C*H_2), 73.6 (C-2), 73.1 (C-4'), 71.2 (C-5'), 67.3 (Linker-O*C*H_2), 66.5 (CBz-*C*H_2), 64.3 (C-6'), 62.8 (C-2'), 52.8 (OCH_3), 38.1 (Linker-N*C*H_2), 29.5 (Linker-*C*H_2), 27.0 (SiC(*C*H_3)$_3$), 19.4 (Si*C*(CH_3)$_3$). **ESI-HRMS** for $C_{61}H_{68}O_{14}N_4SiNa^+$ $(M+Na)^+$ calculated: 1131.4394; found: 1131.4402.

***N*-Benzyloxycarbonyl-3-aminopropyl methyl (2-azido-3-*O*-benzyl-6-*O*-*tert*-butyldiphenylsilyl-2-deoxy-4-*O*-levulinoyl-α-D-glucopyranosyl)-(1→4)-(2-*O*-benzoyl-3-*O*-benzyl-β-D-glucopyranosyluronate)-(1→4)-(2-azido-3-*O*-benzyl-6-*O*-*tert*-butyldiphenylsilyl-2-deoxy-α-D-glucopyranosyl)-(1→4)-(2-*O*-benzoyl-3-*O*-benzyl-β-D-glucopyranosyluronate) 20**

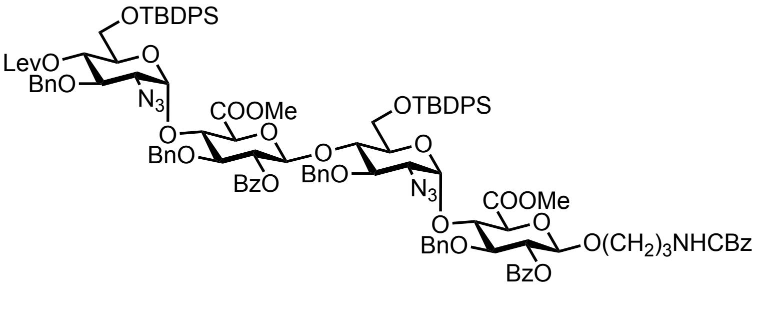

In a two-neck RBF, imidate **17** (214 mg, 0.184 mmol), acceptor **19** (170 mg, 0.153 mmol) and freshly activated 4Å molecular sieves, were placed under three cycles of vacuum and N_2. The solids were dissolved in anhydrous CH_2Cl_2 (3.1 mL) and pre-dried for 0.5-1 h. The solution was cooled down to -15 °C (using ice and NaCl) and then treated with TMSOTf (6.7 µL, 37 µmol). The reaction was allowed to gradually warm up to room temperature over a period of 3 h. The reaction was then quenched with Et_3N, diluted with CH_2Cl_2 (20 mL) and washed with

H_2O (25 mL). The layers were separated and the product was extracted with CH_2Cl_2 (2 x 20 mL). The combined organic layers were dried over anhydrous $MgSO_4$, filtered and concentrated *in vacuo* to give an off-white foam. Purification by column chromatography (97:3 to 96:4; CH_2Cl_2/Et_2O) gave **20** as a white solid (80 mg, 25% yield). R_f = 0.53, 97:3; CH_2Cl_2/Et_2O. **^{1}H NMR** (400 MHz, $CDCl_3$): δ 8.04 – 7.08 (m, 55H, Ph), 5.51 (d, J = 3.6 Hz, 1H, H-1'''), 5.43 – 5.34 (m, 3H, H-1', H-2'', H-4'''), 5.33 – 5.24 (m, 1H, H-2), 5.16 (d, J = 10.4 Hz, 1H, C*H*HPh), 5.03 (s, 2H, CBz-CH_2), 5.03 – 4.94 (m, 1H, NH), 4.93 (d, J = 8.2 Hz, 1H, H-1''), 4.86 – 4.66 (m, 5H, 5 x C*H*HPh), 4.61 – 4.50 (m, 3H, 2 x C*H*HPh, H-1), 4.21 (t, J = 9.2 Hz, 1H, H-4''), 4.15 (t, J = 9.5 Hz, 1H, H-4'), 4.09 (t, J = 9.0 Hz, 1H, H-4), 3.99 – 3.88 (m, 4H, H-3''', H-3, H-5'', H-5), 3.86 – 3.76 (m, 3H, H-6a', H-6b', Linker-OC*H*H), 3.75 – 3.58 (m, 4H, H-3'', H-3', H-6a''', H-6b'''), 3.54 – 3.42 (m, 2H, H-5''', Linker-OC*H*H), 3.39 (s, 3H, OCH_3), 3.32 (dd, J = 10.4, 3.6 Hz, 1H, H-2'''), 3.18 (dd, J = 10.4, 3.9 Hz, 1H, H-2'), 3.15 – 3.01 (m, 3H, Linker-NCH_2, H-5'), 2.89 (s, 3H, OCH_3), 2.64 (t, J = 6.6 Hz, 2H, Lev-CH_2), 2.41 (t, J = 6.6 Hz, 2H, Lev-CH_2), 2.14 (s, 3H, Lev-CH_3), 1.74 – 1.49 (m, 2H, Linker-CH_2), 1.12 (s, 9H, $SiC(CH_3)_3$), 1.02 (s, 9H, $SiC(CH_3)_3$). **$^{13}C\{^1H\}$ NMR** (101 MHz, $CDCl_3$): δ 206.1 (C=O), 171.1 (C=O), 168.5 (C=O), 167.8 (C=O), 165.2 (C=O), 164.7 (C=O), 156.5 (CBz-C=O), 138.2 (C), 137.8 (C), 137.3 (C), 137.2 (C), 137.0 (C), 136.1 (CH), 135.92 (CH), 135.89 (CH), 135.8 (CH), 134.1 (C), 133.63 (CH), 133.59 (CH), 133.5 (C), 133.4 (C), 132.3 (C), 130.3 (CH), 130.1 (CH), 129.8 (CH), 129.71 (CH), 129.69 (CH), 129.5 (C), 129.1 (C), 128.8 (CH), 128.7 (CH), 128.6 (CH), 128.54 (CH), 128.49 (CH), 128.4 (CH), 128.3 (CH), 128.20 (CH), 128.07 (CH), 128.06 (CH), 127.99 (CH), 127.97 (CH), 127.92 (CH), 127.88 (CH), 127.69 (CH), 127.68 (CH), 127.64 (CH), 100.9 (C-1, $^1J_{CH}$ = 161.0 Hz, from coupled HSQC), 100.2 (C-1'', $^1J_{CH}$ = 166.0 Hz, from coupled HSQC), 97.6 (C-1''', $^1J_{CH}$ = 179.0 Hz, from coupled HSQC), 97.3 (C-1', $^1J_{CH}$ = 179.0 Hz, from coupled HSQC), 83.0 (C-3''), 82.6 (C-3), 77.4 (C-3'), 77.3 (C-3'''), 76.3 (C-4'), 75.9 (Ph*C*H_2), 74.8 (C-5''), 74.6 (Ph*C*H_2), 74.5 (C-4''), 74.41 (Ph*C*H_2), 74.36 (Ph*C*H_2), 74.0 (C-5), 73.7 (C-4), 73.6 (C-2), 73.3 (C-2''), 71.6 (C-5'), 71.1 (C-5'''), 70.0 (C-4'''), 67.4 (Linker-O*C*H_2), 66.5 (CBz-*C*H_2), 62.9 (C-2'''), 62.7 (C-2'), 61.7 (C-6'''), 60.6 (C-6'), 52.5 (OCH_3), 51.9 (OCH_3), 38.1 (Linker-N*C*H_2), 37.9 (Lev-*C*H_2), 29.9 (Lev-*C*H_3), 29.5 (Linker-*C*H_2), 28.0 (Lev-*C*H_2), 27.1 ($SiC(CH_3)_3$), 26.9 ($SiC(CH_3)_3$), 19.5 ($SiC(CH_3)_3$), 19.4 ($SiC(CH_3)_3$). **ESI-HRMS** for $C_{116}H_{135}O_{27}N_9Si_2^+$ $(M+2NH_4)^{2+}$ calculated: 1070.9497; found: 1070.9510.

***p*-Methoxyphenyl methyl (2-azido-3-*O*-benzyl-6-*O*-chloroacetyl-2-deoxy-4-*O*-levulinoyl-α-D-glucopyranosyl)-(1→4)-(2-*O*-benzoyl-3-*O*-benzyl-β-D-glucopyranosyluronate) 21**

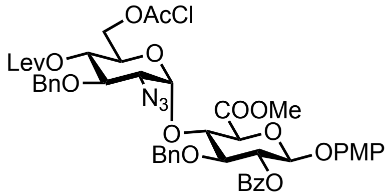

Under a N_2 atmosphere, a solution of disaccharide **15** (1.43 g, 1.27 mmol) in anhydrous pyridine (6 mL) was cooled to 0 °C and treated slowly with HF.pyridine (1.4 mL). The reaction was stirred at 0 °C for 15 min and then warmed up to room temperature. TLC analysis (98:2; CH_2Cl_2/Et_2O, baseline) after 2 h showed complete consumption of starting material. The reaction mixture was diluted with CH_2Cl_2 (25 mL) and washed with saturated $NaHCO_3$ (25 mL). The layers were separated and the aqueous layer was extracted with CH_2Cl_2 (3 x 25 mL). The combined organic layers were dried over anhydrous MgSO4, filtered and concentrated *in vacuo* to give a pale yellow syrup which was used in the next step with further purification.

Under a N_2 atmosphere, a solution of the syrup and DMAP (16 mg, 0.13 mmol) in anhydrous CH_2Cl_2 (3 mL) was treated with anhydrous pyridine (0.21 mL, 2.54 mmol), followed by chloroacetyl chloride (0.21 mL, 2.54 mmol) at room temperature. TLC analysis (95:5; CH_2Cl_2/Et_2O, R_f = 0.44) after 1 h showed complete consumption of starting material. The reaction mixture was diluted with CH_2Cl_2 (25 mL) and washed with H_2O (25 mL). The layers were separated and the aqueous layer was extracted with CH_2Cl_2 (3 x 25 mL). The combined organic layers were dried over anhydrous MgSO4, filtered and concentrated *in vacuo* to give a brown syrup. Purification by column chromatography (90:10; CH_2Cl_2/Et_2O) gave **21** as an off-white foam (1.19 g, 97% yield). $[\alpha]_D^{21}$ = +32.1 (c 0.5, $CHCl_3$).

^{1}H NMR (400 MHz, $CDCl_3$): δ 8.10 – 8.02 (m, 2H, Ph), 7.63 – 7.54 (m, 1H, Ph), 7.49 – 7.39 (m, 2H, Ph), 7.39 – 7.27 (m, 5H, Ph), 7.24 – 7.18 (m, 5H, Ph), 6.94 – 6.85 (m, 2H, Ph), 6.82 – 6.66 (m, 2H, Ph), 5.58 (d, *J* = 3.8 Hz, 1H, H-1'), 5.55 (dd, *J* = 8.1, 6.4 Hz, 1H, H-2), 5.17 (d, *J* = 6.3 Hz, 1H, H-1), 5.00 (dd, *J* = 10.3, 9.2 Hz, 1H, H-4'), 4.80 (d, *J* = 10.6 Hz, 1H, C*H*HPh), 4.78 (d, *J* = 11.1 Hz, 1H, C*H*HPh), 4.76 (d, *J* = 10.6 Hz, 1H, C*H*HPh), 4.68 (d, *J* = 11.1 Hz, 1H, C*H*HPh), 4.48 (t, *J* = 8.8 Hz, 1H, H-4), 4.27 (dd, *J* = 12.4, 2.3 Hz, 1H, H-6a'), 4.24 – 4.19 (m, 2H, H-6b', H-5), 4.19 – 4.05 (m, 3H, AcCl-CH_2, H-3), 3.90 (dd, *J* = 10.3, 9.2 Hz, 1H, H-3'), 3.79 – 3.71 (m, 1H, H-5'), 3.74 (s, 3H, OCH_3), 3.70 (s, 3H, OCH_3), 3.35 (dd, *J* = 10.3, 3.8 Hz, 1H, H-2'), 2.78 – 2.60 (m, 2H, Lev-CH_2), 2.57 – 2.45 (m, 1H, Lev-CH*H*), 2.45 – 2.33 (m, 1H, Lev-CH*H*), 2.16 (s, 3H, Lev-CH_3). **^{13}C{^{1}H} NMR**(101 MHz, $CDCl_3$): δ 206.3 (C=O),

171.8 (C=O), 168.6 (C=O), 167.4 (C=O), 165.2 (C=O), 155.8 (C), 151.0 (C), 137.6 (C), 137.3 (C), 133.7 (CH), 129.9 (CH), 129.5 (C), 128.7 (CH), 128.6 (CH), 128.5 (CH), 128.10 (CH), 128.08 (CH), 128.0 (CH), 127.9 (CH), 118.7 (CH), 114.6 (CH), 100.7 (C-1), 97.6 (C-1'), 82.0 (C-3), 77.4 (C-3'), 75.0 (Ph*C*H_2), 74.5 (Ph*C*H_2), 74.37 (C-4 and C-5), 74.1 (C-2), 70.3 (C-4'), 68.9 (C-5'), 63.5 (C-6'), 62.9 (C-2'), 55.8 (OCH_3), 53.0 (OCH_3), 41.1 (AcCl-*C*H_2), 37.9 (Lev-*C*H_2), 29.8 (Lev-*C*H_3), 28.0 (Lev-*C*H_2). **ESI-HRMS** for $C_{48}H_{54}O_{16}N_4Cl$ $(M+NH_4)^+$ calculated: 977.3218; found: 977.3223.

***p*-Methoxyphenyl methyl (2-azido-3-*O*-benzyl-6-*O*-chloroacetyl-2-deoxy-α-D-glucopyranosyl)-(1→4)-(2-*O*-benzoyl-3-*O*-benzyl-β-D-glucopyranosyluronate) 22**

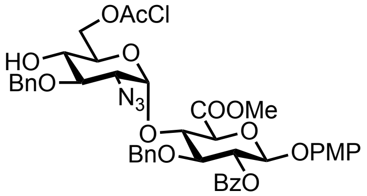

A solution of **21** (1.17 g, 1.22 mol) and N_2H_4.AcOH (561 mg, 6.10 mmol) in bench CH_2Cl_2/MeOH (1:1; 6 mL) was stirred at room temperature for 2.5 h. The reaction was diluted with CH_2Cl_2 (25 mL) and washed with H_2O (25 mL). The layers were separated and the product was extracted with CH_2Cl_2 (3 x 25 mL). The combined organic layers were dried over anhydrous $MgSO_4$, filtered and concentrated *in vacuo* to give a pale yellow syrup. Purification by column chromatography (90:10; CH_2Cl_2/Et_2O, $R_f = 0.63$) gave **22** as an off-white foam (844 mg, 80% yield). $[\alpha]_D^{21} = +33.6$ (c 0.5, $CHCl_3$). **^{1}H NMR** (400 MHz, $CDCl_3$): δ 8.10 – 8.01 (m, 2H, Ph), 7.61 – 7.55 (m, 1H, Ph), 7.48 – 7.42 (m, 2H, Ph), 7.42 – 7.28 (m, 5H, Ph), 7.24 – 7.16 (m, 5H, Ph), 6.94 – 6.86 (m, 2H, Ph), 6.80 – 6.73 (m, 2H, Ph), 5.59 – 5.50 (m, 2H, H-2, H-1'), 5.16 (d, *J* = 6.5 Hz, 1H, H-1), 4.91 (d, *J* = 11.1 Hz, 1H, C*H*HPh), 4.81 (d, *J* = 10.6 Hz, 1H, C*H*HPh), 4.79 (d, *J* = 11.1 Hz, 1H, C*H*HPh), 4.76 (d, *J* = 10.6 Hz, 1H, C*H*HPh), 4.51 – 4.44 (m, 2H, H-4, H-6a'), 4.41 (dd, *J* = 12.1, 2.3 Hz, 1H, H-6b'), 4.20 (d, *J* = 9.0 Hz, 1H, H-5), 4.16 (d, *J* = 15.0 Hz, 1H, AcCl-CH*H*), 4.11 (d, *J* = 15.0 Hz, 1H, AcCl-CH*H*), 4.09 (t, *J* = 8.4 Hz, 1H, H-3), 3.77 – 3.69 (m, 1H, H-3'), 3.74 (s, 3H, OCH_3), 3.69 (s, 3H, OCH_3), 3.60 (ddd, *J* = 10.1, 4.1, 2.3 Hz, 1H, H-5'), 3.45 (ddd, *J* = 10.1, 8.6, 3.7 Hz, 1H, H-4'), 3.25 (dd, *J* = 10.3, 3.8 Hz, 1H, H-2'), 2.56 (d, *J* = 3.7 Hz, 1H, OH). **^{13}C{^{1}H} NMR** (101 MHz, $CDCl_3$): δ 168.6 (C=O), 168.2 (C=O), 165.2 (C=O), 155.8 (C), 151.0 (C), 137.8 (C), 137.3 (C), 133.6 (CH), 129.9 (CH), 129.5 (C), 128.9 (CH), 128.7 (CH), 128.5 (CH), 128.4 (CH), 128.3 (CH), 128.0 (2 x CH), 118.7 (CH), 114.6 (CH), 100.8 (C-1), 97.9 (C-1'), 82.1 (C-3), 79.5 (C-3'), 75.4 (Ph*C*H_2), 74.5

(Ph*C*H_2), 74.4 (C-5), 74.3 (C-4), 74.0 (C-2), 70.6 (C-5'), 70.4 (C-4'), 64.3 (C-6'), 62.9 (C-2'), 55.8 (OCH_3), 53.0 (OCH_3), 40.9 (AcCl-*C*H_2). **ESI-HRMS** for $C_{43}H_{44}O_{14}N_3ClNa$ $(M+Na)^+$ calculated: 884.2404; found: 884.2414.

***p*-Methoxyphenyl methyl (2-azido-3-*O*-benzyl-6-*O*-chloroacetyl-2-deoxy-4-*O*-fluorenylmethoxycarbonyl-α-D-glucopyranosyl)-(1→4)-(2-*O*-benzoyl-3-*O*-benzyl-β-D-glucopyranosyluronate) 23**

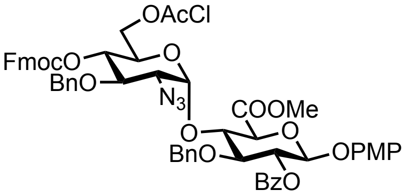

Under a N_2 atmosphere, a solution of **22** (2.08 g, 2.41 mmol) and FmocCl (6.24 g, 24.1 mmol) in anhydrous CH_2Cl_2 (24 mL) was treated with anhydrous pyridine (0.49 mL, 6.03 mmol). The reaction was stirred at room temperature for 19 h and then diluted with CH_2Cl_2 (25 mL) and washed successively with 1M HCl (80 mL) and H_2O (60 mL). The layers were separated and the product was extracted with CH_2Cl_2 (3 x 100 mL). The combined organic layers were dried over anhydrous $MgSO_4$, filtered and concentrated *in vacuo* to give a pink syrup. Purification by column chromatography (100:0 to 95:5; CH_2Cl_2/Et_2O) gave **23** as a white foam (2.31 g, 89% yield). R_f = 0.77; CH_2Cl_2/Et_2O. $[\alpha]_D^{21}$ = +19.7 (c 0.43, $CHCl_3$). **^{1}H NMR** (400 MHz, $CDCl_3$): δ 8.08 – 8.02 (m, 2H, Ph), 7.79 – 7.72 (m, 2H, Ph), 7.62 – 7.51 (m, 3H, Ph), 7.48 – 7.43 (m, 2H, Ph), 7.41 – 7.36 (m, 2H, Ph), 7.33 – 7.24 (m, 3H, Ph), 7.25 – 7.17 (m, 9H, Ph), 6.94 – 6.88 (m, 2H, Ph), 6.80 – 6.75 (m, 2H, Ph), 5.56 (d, *J* = 3.7 Hz, 1H, H-1'), 5.54 (dd, *J* = 7.9, 6.1 Hz, 1H, H-2), 5.18 (d, *J* = 6.1 Hz, 1H, H-1), 4.82 (dd, *J* = 10.4, 9.2 Hz, 1H, H-4'), 4.79 (d, *J* = 10.6 Hz, 1H, C*H*HPh), 4.76 (d, *J* = 10.6 Hz, 1H, C*H*HPh), 4.72 (d, *J* = 10.9 Hz, 1H, C*H*HPh), 4.62 (d, *J* = 10.9 Hz, 1H, C*H*HPh), 4.54 – 4.45 (m, 2H, Fmoc-CH*H*, H-4), 4.33 (dd, *J* = 10.5, 7.1 Hz, 1H, Fmoc-CH*H*), 4.30 – 4.26 (m, 2H, H-6a', H-6b'), 4.22 – 4.16 (m, 1H, Fmoc-CH), 4.21 (d, *J* = 8.8 Hz, 1H, H-5), 4.15 (d, *J* = 15.3 Hz, 1H, AcCl-CH*H*), 4.11 (d, *J* = 15.3 Hz, 1H, AcCl-CH*H*), 4.09 (t, *J* = 8.2 Hz, 1H, H-3), 3.91 (dd, *J* = 10.4, 9.0 Hz, 1H, H-3'), 3.83 (ddd, *J* = 10.4, 4.0, 2.6 Hz, 1H, H-5'), 3.75 (s, 3H, OCH_3), 3.67 (s, 3H, OCH_3), 3.34 (dd, *J* = 10.3, 3.7 Hz, 1H, H-2'). **^{13}C{^{1}H} NMR** (101 MHz, $CDCl_3$): δ 168.7 (C=O), 167.4 (C=O), 165.2 (C=O), 155.8 (C), 154.4 (C=O), 151.0 (C), 143.3 (C), 143.1 (C), 141.46 (C), 141.45 (C), 137.3 (C), 137.2 (C), 133.7 (CH), 130.0(CH), 129.5 (C), 128.7 (CH), 128.54 (CH), 128.52 (CH), 128.11 (CH), 128.09 (CH), 128.03 (CH), 128.01 (CH), 127.9 (CH), 127.37 (CH), 127.36 (CH), 125.2 (CH), 125.0 (CH), 120.3 (CH), 120.2 (CH), 118.6 (CH), 114.6 (CH), 100.6 (C-1),

97.7 (C-1’), 81.7 (C-3), 77.4 (C-3’), 75.3 (Ph*C*H$_2$), 74.6 (C-4), 74.51 (C-4’), 74.46 (Ph*C*H$_2$), 74.3 (C-5), 74.1 (C-2), 70.5 (Fmoc-*C*H$_2$), 68.5 (C-5’), 63.3 (C-6’), 62.8 (C-2’), 55.8 (OCH$_3$), 53.0 (OCH$_3$), 46.8 (Fmoc-*C*H), 41.0 (AcCl-*C*H$_2$). **ESI-HRMS** for $C_{58}H_{58}O_{16}N_4Cl$ $(M+NH_4)^+$ calculated: 1101.3531; found: 1101.3539.

Methyl (2-azido-3-*O*-benzyl-6-*O*-chloroacetyl-2-deoxy-4-*O*-fluorenylmethoxycarbonyl-α-D-glucopyranosyl)-(1→4)-(2-*O*-benzoyl-3-*O*-benzyl-α/β-D-glucopyranosyluronate) S18

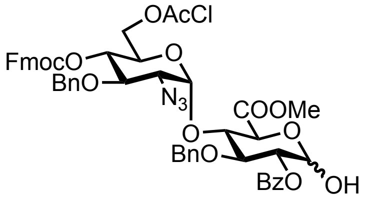

A solution of **23** (624 mg, 0.575 mmol) in Tol/MeCN/H_2O (1:1.5:1; 12 mL) was cooled to 0 °C and treated with CAN (1.58 g, 2.88 mmol). The reaction was left to stir at 0 °C. After 30 minutes the reaction was diluted with EtOAc (40 mL) and washed with H_2O (40 mL). The layers were separated and the product was extracted with EtOAc (3 x 40 mL). The combined organic layers were dried over anhydrous $MgSO_4$, filtered and concentrated *in vacuo* to give an orange syrup. Purification by column chromatography (2:1; hex/EtOAc, $R_f = 0.36$) gave **S18** as an orange foam (439 mg, 78% yield, α/β = 89:11). $R_f = 0.33$, 95:5; CH_2Cl_2/Et_2O.

α-anomer

^{1}H NMR (400 MHz, $CDCl_3$): δ 8.13 – 8.04 (m, 2H, Ph), 7.82 – 7.73 (m, 2H, Ph), 7.65 – 7.52 (m, 2H, Ph), 7.49 – 7.36 (m, 5H, Ph), 7.34 – 7.16 (m, 10H, Ph), 7.16 – 7.07 (m, 2H, Ph), 5.68 (dd, J = 7.0, 2.9 Hz, 1H, H-1), 5.33 (d, J = 3.7 Hz, 1H, H-1’), 5.11 (dd, J = 6.9, 2.9 Hz, 1H, H-2), 4.86 – 4.76 (m, 3H, 2 x C*H*HPh, H-5), 4.72 (dd, J = 10.3, 9.1 Hz, 1H, H-4’), 4.61 (dd, J = 10.6, 6.3 Hz, 1H, Fmoc-CH*H*), 4.41 (dd, J = 10.6, 6.7 Hz, 1H, Fmoc-CH*H*), 4.38 – 4.33 (m, 2H, H-3, C*H*HPh), 4.32 – 4.20 (m, 4H, H-6a’, H-4, C*H*HPh, Fmoc-CH*H*), 4.20 – 4.11 (m, 3H, AcCl-CH$_2$, H-6b’), 4.07 (ddd, J = 10.3, 5.7, 2.1 Hz, 1H, H-5’), 3.70 (s, 3H, OCH$_3$), 3.65 (dd, J = 10.3, 9.1 Hz, 1H, H-3’), 3.25 (dd, J = 10.3, 3.7 Hz, 1H, H-2’), 3.19 (d, J = 7.2 Hz, 1H, OH). **^{13}C{^{1}H} NMR** (101 MHz, $CDCl_3$): δ 169.4 (C=O), 167.7 (C=O), 165.8 (C=O), 154.5 (C=O), 143.22 (C), 143.18 (C), 141.54 (C), 141.52 (C), 137.4 (C), 137.3 (C), 133.7 (CH), 130.0 (CH), 129.4 (C), 129.0 (CH), 128.6 (CH), 128.5 (CH), 128.16 (CH), 128.15 (CH), 128.1 (CH), 128.0 (CH), 127.8 (CH), 127.7 (CH), 127.41 (CH), 127.40 (CH), 125.1 (CH), 124.9 (CH), 120.32 (CH), 120.30 (CH), 98.8 (C-1’, $^1J_{1CH}$ = 175.5 Hz, from coupled HSQC), 89.7 (C-1, $^1J_{1CH}$ = 175.4 Hz, from coupled HSQC), 77.3 (C-3’), 76.8 (C-3), 76.0 (C-4), 75.2 (Ph*C*H$_2$), 74.8 (C-

4'), 74.4 (Ph*C*H_2), 71.9 (C-5), 71.8 (C-2), 70.3 (Fmoc-*C*H_2), 68.5 (C-5'), 63.7 (C-6'), 62.8 (C-2'), 52.8 (OCH_3), 47.0 (Fmoc-*C*H), 41.2 (AcCl-*C*H_2).

β-anomer

^{1}H NMR (400 MHz, $CDCl_3$) selected signals: δ 5.43 (d, *J* = 3.7 Hz, 1H, H-1'), 5.20 (dd, *J* = 7.8, 6.4 Hz, 1H, H-2), 4.97 (dd, *J* = 9.1, 6.5 Hz, 1H, H-1), 3.77 (s, 3H, OCH_3), 3.31 (dd, *J* = 10.3, 3.7 Hz, 1H, H-2'). **^{13}C{^{1}H} NMR** (101 MHz, $CDCl_3$) selected signal: δ 95.8 (C-1). **ESI-HRMS** for $C_{51}H_{48}O_{15}N_3ClNa$ $(M+Na)^+$ calculated: 1000.2666; found: 1000.2676.

Diethyl methyl (2-azido-3-*O*-benzyl-6-*O*-chloroacetyl-2-deoxy-4-*O*-fluorenylmethoxycarbonyl-α-D-glucopyranosyl)-(1→4)-(2-*O*-benzoyl-3-*O*-benzyl-β-D-glucopyranosyluronate) phosphate 24

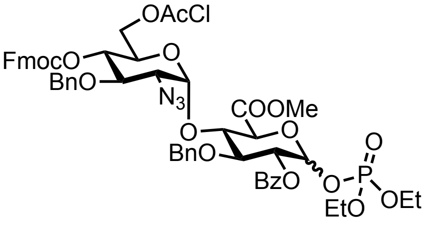

Under a N_2 atmosphere, a mixture of **S18** (395 mg, 0.404 mmol) and K_2CO_3 (112 mg, 0.808 mmol) in anhydrous CH_2Cl_2 (1.3 mL) was treated with diethyl chlorophosphate (0.07 mL, 0.81 mmol) at 0 °C. After 5 minutes Cs_2CO_3 (132 mg, 0.404 mmol) was added to the reaction mixture. The reaction was then stirred at room temperature for 2.5 h. TLC analysis (1:1; hex/EtOAc, R_f = 0.5) showed complete consumption of starting material. The reaction mixture was directly purified by column chromatography (1:1; hex/EtOAc with 0.5% Et_3N) to give **24** as a white foam (380 mg, 84% yield, α/β = 97:3). **^{1}H NMR** (400 MHz, $CDCl_3$): δ 8.08 – 8.03 (m, 2H, Ph), 7.80 – 7.71 (m, 2H, Ph), 7.63 – 7.53 (m, 3H, Ph), 7.48 – 7.42 (m, 2H, Ph), 7.44 – 7.35 (m, 2H, Ph), 7.31 – 7.26 (m, 2H, Ph), 7.25 – 7.13 (m, 10H, Ph), 5.53 – 5.48 (m, 2H, H-1, H-1'), 5.44 (dd, *J* = 8.3, 7.4 Hz, 1H, H-2), 4.81 (dd, *J* = 10.3, 9.3 Hz, 1H, H-4'), 4.79 – 4.69 (m, 3H, 3 x C*H*HPh), 4.64 (d, *J* = 10.9 Hz, 1H, C*H*HPh), 4.51 (dd, *J* = 10.5, 6.6 Hz, 1H, Fmoc-CH*H*), 4.36 – 4.28 (m, 2H, Fmoc-CH*H*, H-4), 4.26 (app d, *J* = 3.3 Hz, 2H, H-6a', H-6b'), 4.23 – 4.16 (m, 1H, Fmoc-CH), 4.20 (d, *J* = 9.4 Hz, 1H, H-5), 4.16 – 4.06 (m, 4H, AcCl-CH_2, Et-OCH_2), 4.02 (t, *J* = 8.3 Hz, 1H, H-3), 3.90 (dd, *J* = 10.3, 9.4 Hz, 1H, H-3'), 3.87 – 3.80 (m, 2H, Et-OCH_2), 3.75 (dt, *J* = 10.4, 3.3 Hz, 1H, H-5'), 3.34 (dd, *J* = 10.3, 3.7 Hz, 1H, H-2'), 1.30 (td, *J* = 7.1, 1.1 Hz, 3H, Et-CH_3), 0.97 (td, *J* = 7.1, 1.1 Hz, 3H, Et-CH_3). **^{13}C{^{1}H} NMR** (101 MHz, $CDCl_3$): δ 168.1 (C=O), 167.3 (C=O), 165.0 (C=O), 154.4 (C=O), 143.3 (C), 143.1 (C),

141.47 (C), 141.45 (C), 137.2 (C), 137.1 (C), 133.8 (CH), 130.0 (CH), 129.2 (C), 128.8 (CH), 128.54 (CH), 128.53 (2 x CH), 128.13 (CH), 128.10 (CH), 128.1 (CH), 127.9 (CH), 127.4 (2 x CH), 125.2 (CH), 125.0 (CH), 120.3 (CH), 120.2 (CH), 97.6 (C-1'), 96.4 (d, *J* = 4.9 Hz, C-1), 81.9 (C-3), 77.3 (C-3'), 75.4 (Ph*C*H_2), 74.94 (C-4), 74.86 (Ph*C*H_2), 74.5 (C-5), 74.4 (C-4'), 73.2 (d, *J* = 8.4 Hz, C-2), 70.5 (Fmoc-*C*H_2), 68.6 (C-5'), 64.7 (d, *J* = 6.2 Hz, Et-O*C*H_2), 64.5 (d, *J* = 6.0 Hz, Et-O*C*H_2), 63.2 (C-6'), 62.8 (C-2'), 53.1 (OCH_3), 46.8 (Fmoc-*C*H), 41.0 (AcCl-*C*H_2), 16.0 (d, *J* = 7.3 Hz, Et-O*C*H_3), 15.7 (d, *J* = 7.0 Hz, Et-O*C*H_3). **^{31}P NMR** (162 MHz, $CDCl_3$): δ -2.80 (s, 1P, α), -3.29 (q, *J* = 7.7 Hz, 1P, β). **ESI-HRMS** for $C_{55}H_{61}O_{18}N_4ClP$ $(M+NH_4)^+$ calculated: 1131.3402; found: 1131.3414.

***p*-Methoxyphenyl methyl (2-azido-3-*O*-benzyl-6-*O*-chloroacetyl-2-deoxy-4-*O*-fluorenylmethoxycarbonyl-α-D-glucopyranosyl)-(1→4)-(2-*O*-benzoyl-3-*O*-benzyl-β-D-glucopyranosyluronate)-(1→4)-(2-azido-3-*O*-benzyl-6-*O*-chloroacetyl-2-deoxy-D-glucopyranosyl)-(1→4)-(2-*O*-benzoyl-3-*O*-benzyl-β-D-glucopyranosyluronate 25**

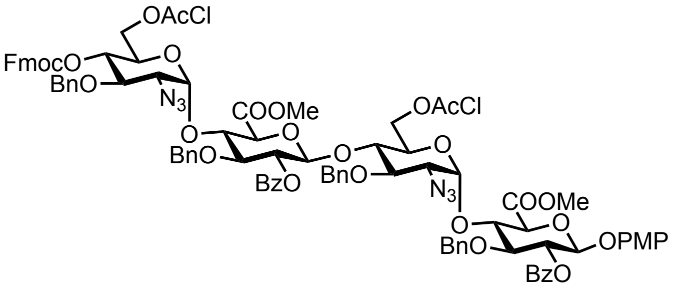

Phosphate **24** (266 mg, 0.239 mmol) and freshly activated 4Å molecular sieves, were placed under three cycles of vacuum and N_2. The solid were dissolved in anhydrous CH_2Cl_2 (4.8 mL) and pre-dried for 1 h. The solution was cooled down to -45 °C (using dry-ice and MeCN) and then treated with TMSOTf (65 μL, 0.36 mmol). After 15 min, a 0.1 M solution of the acceptor **22** in anhydrous CH_2Cl_2 (0.16 mL, 0.159 mmol) was added to the reaction. The reaction was allowed to gradually warm up to room temperature over a period of 4 h. TLC analysis (1:1; hex/EtOAc, R_f = 0.63) showed complete consumption of donor **24**. The reaction was quenched with Et_3N (30 μL) and concentrated *in vacuo* to give an off-white foam. Purification by column chromatography (column 1 to remove unreacted acceptor: 97:3, CH_2Cl_2/Et_2O, R_f = 0.67; column 2 to remove donor side products: 2:1; hex/EtOAc, R_f = 0.29) gave **25** as a white foam (175 mg, 60% yield, β-only). **^{1}H NMR** (400 MHz, $CDCl_3$): δ 8.14 – 8.09 (m, 2H, Ph), 8.06 – 8.00 (m, 2H, Ph), 7.80 – 7.71 (m, 2H, Ph), 7.65 – 7.33 (m, 15H, Ph), 7.34 – 7.25 (m, 3H, Ph), 7.25 – 7.07 (m, 14H, Ph), 6.90 – 6.82 (m, 2H, Ph), 6.78 – 6.70 (m, 2H, Ph), 5.54 – 5.46 (m,

3H, H-2, H-1', H-1'''), 5.43 (dd, *J* = 8.9, 7.8 Hz, 1H, H-2''), 5.15 (d, *J* = 10.8 Hz, 1H, C*H*HPh), 5.07 (d, *J* = 6.7 Hz, 1H, H-1), 4.81 (dd, *J* = 10.4, 9.2 Hz, 1H, H-4'''), 4.80 – 4.59 (m, 8H, 7 x C*H*HPh, H-1''), 4.51 (dd, *J* = 10.5, 6.7 Hz, 1H, Fmoc-CH*H*), 4.36 – 4.25 (m, 7H, H-6a', H-6a''', Fmoc-CH*H*, H-4, H-4'', H-6b', H-6b'''), 4.23 – 4.16 (m, 1H, Fmoc-CH), 4.14 – 3.97 (m, 7H, 2 x AcCl-CH_2, H-5, H-3, H-5''), 3.97 – 3.92 (m, 1H, H-3''), 3.93 – 3.86 (m, 1H, H-3'''), 3.82 – 3.76 (m, 2H, H-3', H-4'), 3.74 (s, 3H, OCH_3), 3.74 – 3.69 (m, 1H, H-5'''), 3.56 (s, 3H, OCH_3), 3.46 (dt, *J* = 9.7, 2.7 Hz, 1H, H-5'), 3.35 (dd, *J* = 10.3, 3.7 Hz, 1H, H-2'''), 3.24 (dd, *J* = 9.9, 3.9 Hz, 1H, H-2'), 3.17 (s, 3H, OCH_3). **$^{13}C\{^1H\}$ NMR** (101 MHz, $CDCl_3$): δ 168.3 (C=O), 168.0 (C=O), 167.3 (C=O), 167.1 (C=O), 165.1 (C=O), 164.8 (C=O), 155.8 (C), 154.3 (C=O), 151.0 (C), 143.3 (C), 143.1 (C), 141.5 (C), 141.4 (C), 138.2 (C), 137.3 (C), 137.2 (2 x C), 134.0 (CH), 133.6 (CH), 129.94 (CH), 129.91 (CH), 129.5 (C), 129.0 (CH), 128.9 (C), 128.7 (CH), 128.52 (CH), 128.50 (CH), 128.48 (CH), 128.47 (CH), 128.12 (CH), 128.09 (CH), 128.06 (CH), 127.99 (CH), 127.96 (CH), 127.95 (CH), 127.7 (CH), 127.4 (CH), 127.3 (CH), 125.2 (CH), 125.0 (CH), 120.3 (CH), 120.2 (CH), 118.8 (CH), 114.6 (CH), 101.2 (C-1'', $^1J_{1CH}$ = 164.0 Hz, from coupled HSQC), 100.8 (C-1, $^1J_{1CH}$ = 166.0 Hz, from coupled HSQC), 97.6, 97.4 (C-1', C-1''', $^1J_{1CH}$ = 179.0 Hz, from coupled HSQC), 82.5 (C-3''), 82.3 (C-3), 77.82, 77.80 (C-3', C-4'), 77.4 (C-3'''), 75.7 (Ph*C*H_2), 75.5 (C-4''), 75.3 (Ph*C*H_2), 75.1 (Ph*C*H_2), 74.7 (Ph*C*H_2), 74.6 (C-5''), 74.4 (C-4'''), 74.3 (C-4), 74.0 (C-5), 73.9 (C-2), 73.7 (C-2''), 70.5 (Fmoc-*C*H_2), 69.0 (C-5'), 68.5 (C-5'''), 63.3, 63.2 (C-6', C-6'''), 62.8 (C-2', C-2'''), 55.7 (OCH_3), 52.9 (OCH_3), 52.2 (OCH_3), 46.8 (Fmoc-*C*H), 41.0 (AcCl-*C*H_2), 40.9 (AcCl-*C*H_2). **ESI-HRMS** for $C_{94}H_{94}O_{28}N_7Cl_2$ $(M+NH_4)^+$ calculated: 1838.5518; found: 1838.5481.

References

(1) Pangborn, A. B.; Giardello, M. A.; Grubbs, R. H.; Rosen, R. K.; Timmers, F. J.; *Organometallics* **1996**, *15*, 1518–1520.

(2) Cai, C.; Dickinson, D. M.; Li, L.; Masuko, S.; Suflita, M.; Schultz, V.; Nelson, S. D.; Bhaskar, U.; Liu, J.; Linhardt, R. J.; *Org. Lett.* **2014**, *16*, 2240–2243.

(3) Chen, C. W.; Wang, C. C.; Li, X. R.; Witek, H.; Mong, K. K. T.; *Org. Biomol. Chem.* **2020**, *18*, 3135–3141.

(4) Wang, C. C.; Lee, J. C.; Luo, S. Y.; Kulkarni, S. S.; Huang, Y. W.; Lee, C. C.; Chang, K. L.; Hung, S. C.; *Nature* **2007**, *446*, 896–899.

(5) Zulueta, M. M. L.; Lin, S. Y.; Lin, Y. T.; Huang, C. J.; Wang, C. C.; Ku, C. C.; Shi, Z.; Chyan, C. L.; Irene, D.; Lim, L. H.; Tsai, T. I.; Hu, Y. P.; Arco, S. D.; Wong, C. H.; Hung, S. C.; *J. Am. Chem. Soc.* **2012**, *134*, 8988–8995.

(6) Slaghek, T. M.; Nakahara, Y.; Ogawa, T.; Kamerling, J. P.; Vliegenthart, J. F. G.; *Carbohydr. Res.* **1994**, *255*, 61–85.

(7) Nitz, M.; Bundle, D. R.; *J. Org. Chem.* **2000**, *65*, 3064–3073.

(8) Komarova, B. S.; Orekhova, M. V.; Tsvetkov, Y. E.; Nifantiev, N. E.; *Carbohydr. Res.* **2014**, *384*, 70–86.

(9) Karst, N.; Jacquinet, J. C.; *J. Chem. Soc. Perkin Trans. 1* **2000**, 2709–2717.

(10) Karst, N.; Jacquinet, J. C.; *Eur. J. Org. Chem.* **2002**, 815–825.

(11) Balmond, E. I.; Benito-Alifonso, D.; Coe, D. M.; Alder, R. W.; McGarrigle, E. M.; Galan, M. C.; *Angew. Chem. Int. Ed.* **2014**, *53*, 8190–8194.

(12) Hassan, H. H. A. M.; *Cent. Eur. J. Chem.* **2005**, *3* (4), 803–829.

(13) Mong, T. K.-K.; Huang, C.; Wong, C.; *J. Org. Chem.* **2003**, *68*, 2135–2142.

(14) DeNinno, M. P.; Etienne, J. B.; Duplantier, K. C.; *Tetrahedron Lett.* **1995**, *36*, 669–672.

(15) Wu, X.; Schmidt, R. R.; *Eur. J. Org. Chem.* **2004**, 2826–2832.

(16) Ngoje, G.; Li, Z.; *Org. Biomol. Chem.* **2013**, *11*, 1879–1886.

(17) Morelli, L.; Lay, L.; *Arkivoc* **2012**, *2013*, 166–184.

(18) Codée, J. D. C.; Stubba, B.; Schiattarella, M.; Overkleeft, H. S.; Van Boeckel, C. A. A.; Van Boom, J. H.; Van Der Marel, G. A.; *J. Am. Chem. Soc.* **2005**, *127*, 3767–3773.

(19) Sakairi, N.; Takahashi, S.; Wang, F.; Ueno, Y.; Kuzuhara, H.; *Bull. Chem. Soc. Jpn.* **1994**, *67*, 1756–1758.
