# Supplementary material for: d-Glucuronate and d-Glucuronate Glycal Acceptors for the Scalable Synthesis of d-GlcN-α-1,4-d-GlcA Disaccharides and Modular Assembly of Heparan Sulfate
Source: J Org Chem. 2023 Jul 17;88(15):11130–9. doi: 10.1021/acs.joc.3c01108 (PMC10407932; doi:10.1021/acs.joc.3c01108)

D-Glucuronate and D-glucuronate glycal acceptors for the scalable synthesis of D-GlcN-α-1,4-D-GlcA disaccharides and modular assembly of heparan sulfate

Imlirenla Pongener and Gavin J. Miller*

School of Chemical and Physical Sciences & Centre for Glycoscience, Keele University, Keele, Staffordshire, ST5 5BG, United Kingdom

*Email: g.j.miller@keele.ac.uk

Supporting Information 2
Spectra

Table of Contents

Synthesis of Glucuronic Acceptors S4
Compound S1 .... S4
Compound S2 .... S4
Compound S3a S5
Compound S4a S6
Compound S5a S7
Compound 4a S8
Compound S3b S11
Compound S4b S13
Compound S5b S16
Compound 4b S18
Compound S6a S21
Compound S7 .... S21
Compound S8 .... S22
Compound S9 .... S23
Compound S10 .... S24
Compound 14 .... S25
Synthesis of Glucuronic Glycal Acceptor S26
Compound S26 .... S26

Compound 28 .... S27
Compound 29 .... S28
Synthesis of Glucosamine Donors S31
Compound S12 .... S31
Compound S13 .... S32
Compound 1a S33
Compound 2a S35
Compound 1b S38
Compound 2b S38
Compound 1c S41
Compound 2c S42
Compound S15 .... S43
Compound 3a S45
Compound S16 .... S47
Compound 3b S48
Compound S17 .... S50
Glycosylations S53
Compound 5a S53
Compound 8a S55
Compound 8b S58
Compound 9a S61
Compound 9b S62
Compound 10a S65
Compound 10b S68
Compound 11a S71
Compound 11b S74
Compound 15 .... S77
Compound 30 .... S80
Glycosylation Side Products S83
Compound 6a S83
Compound 6b S85
Compound 7a S88
Compound 7b S91
Compound 13a S94
Transformations of Disaccharide Building Block S96
Compound 16 .... S96
Compound 17 .... S99

Compound 18 .... S101
Compound 19 .... S104
Compound 20 .... S106
Compound 21 .... S110
Compound 22 .... S113
Compound 23 .... S116
Compound S18 .... S119
Compound 24 .... S122
Compound 25 .... S126

Synthesis of Glucuronic Acceptors

Compound S1

^{1}H NMR (400 MHz, Chloroform-*d*) S1

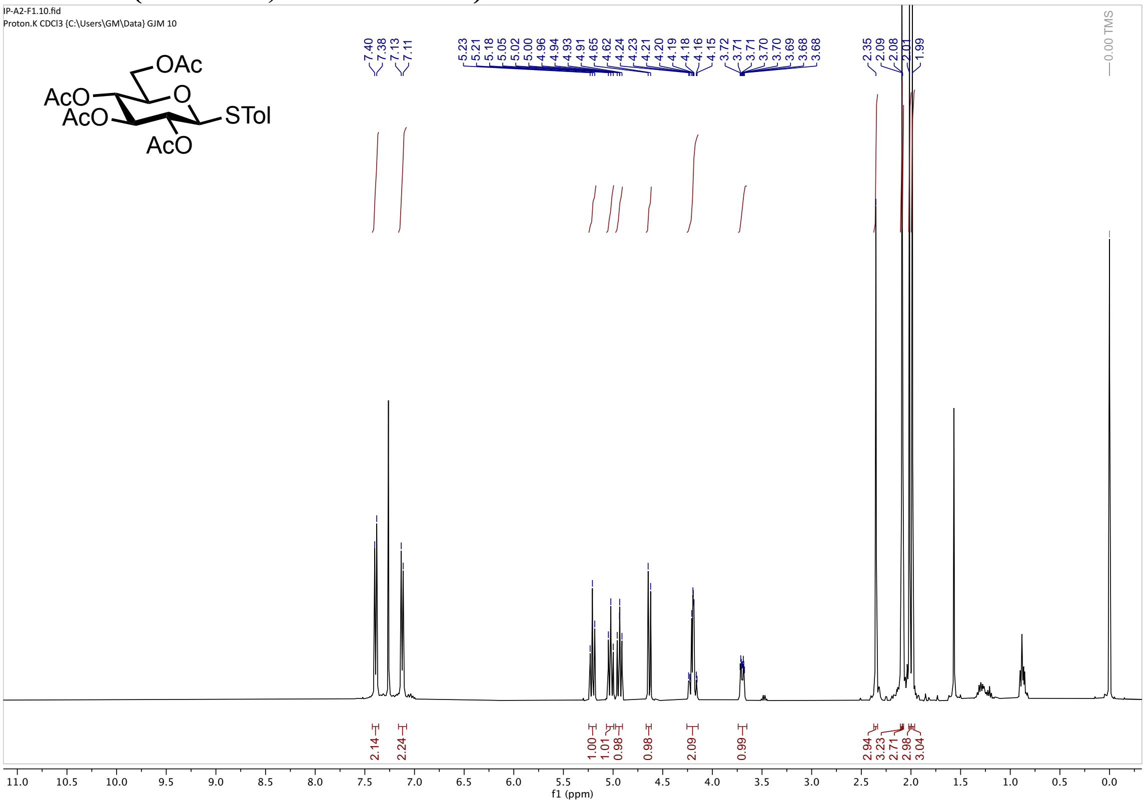

Compound S2

^{1}H NMR (400 MHz, Chloroform-*d*) S2

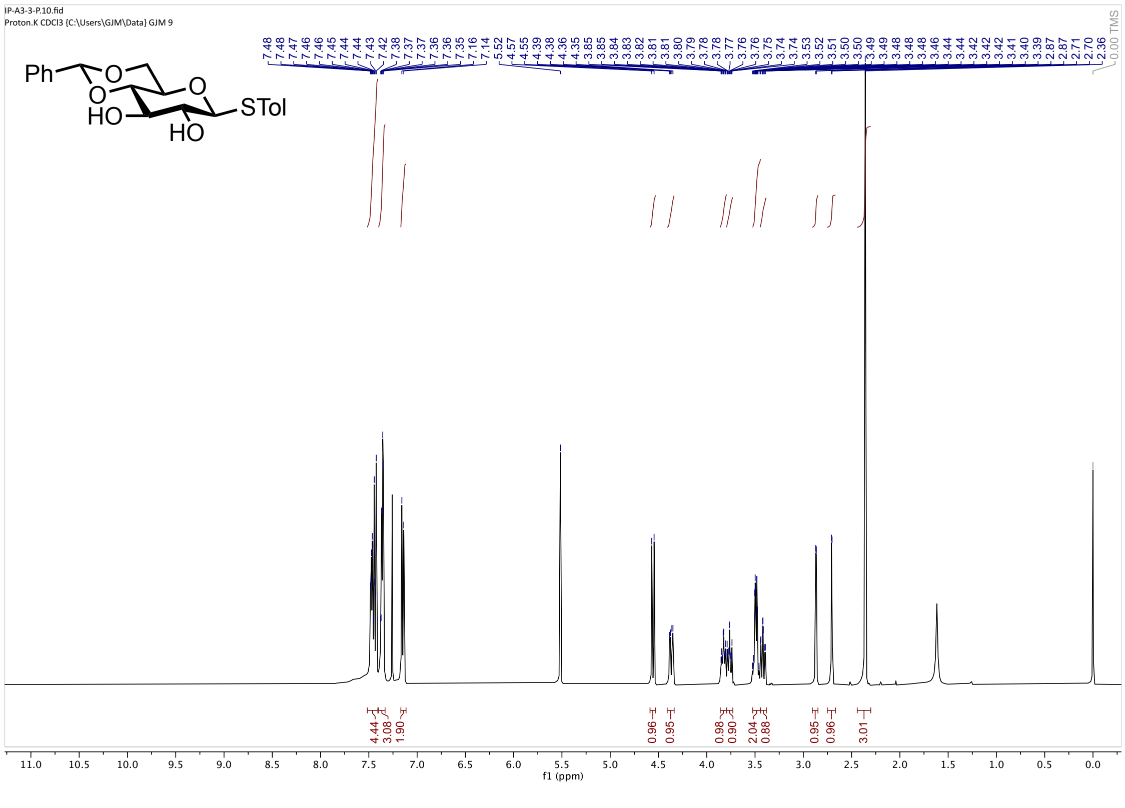

$^{13}C\{^1H\}$ NMR (101 MHz, Chloroform-*d*) S2

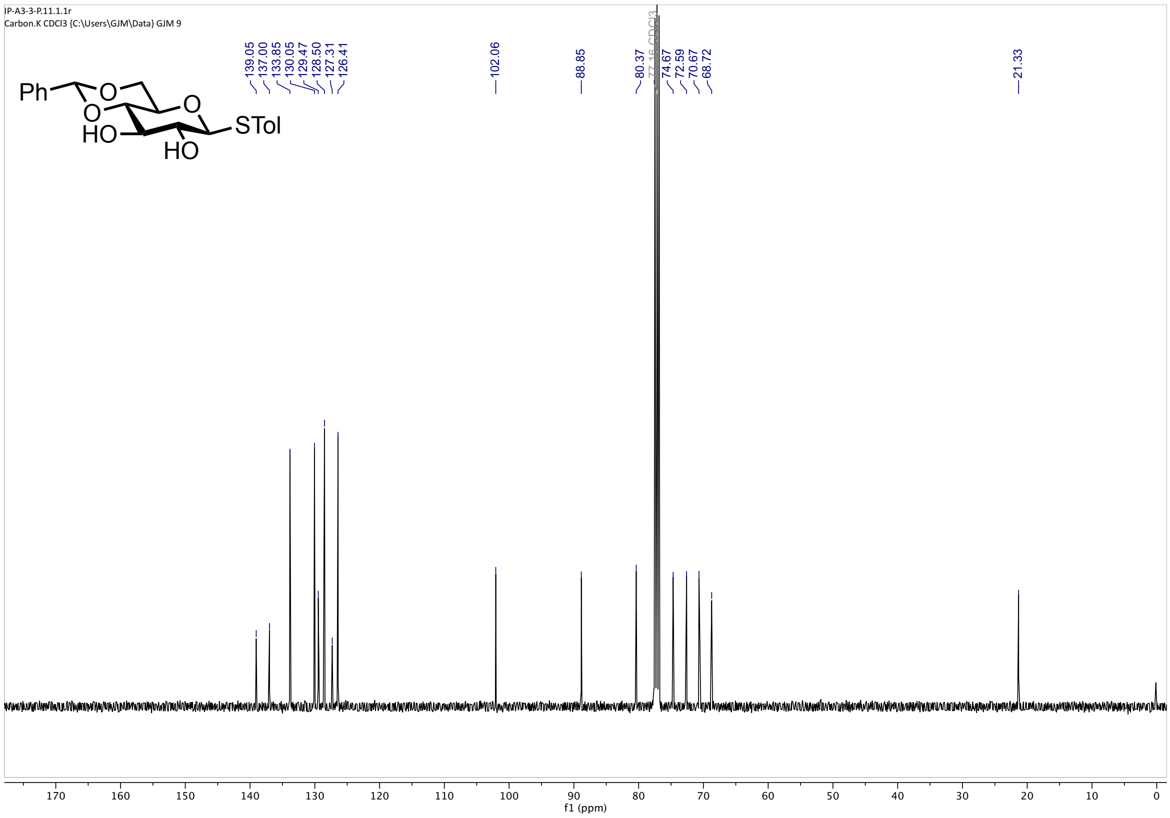

Compound S3a

^1H NMR (400 MHz, Chloroform-*d*) S3a

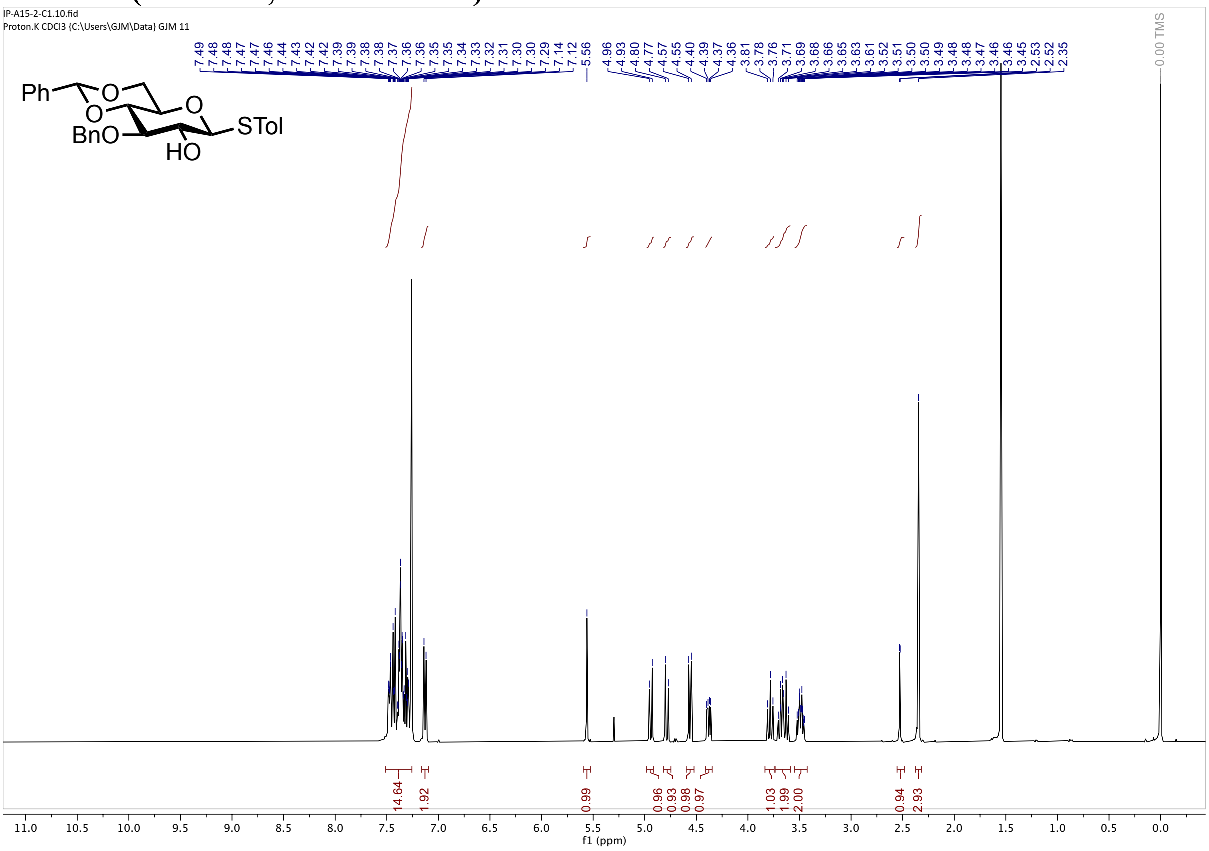

$^{13}C\{^1H\}$ NMR (101 MHz, Chloroform-*d*) S3a

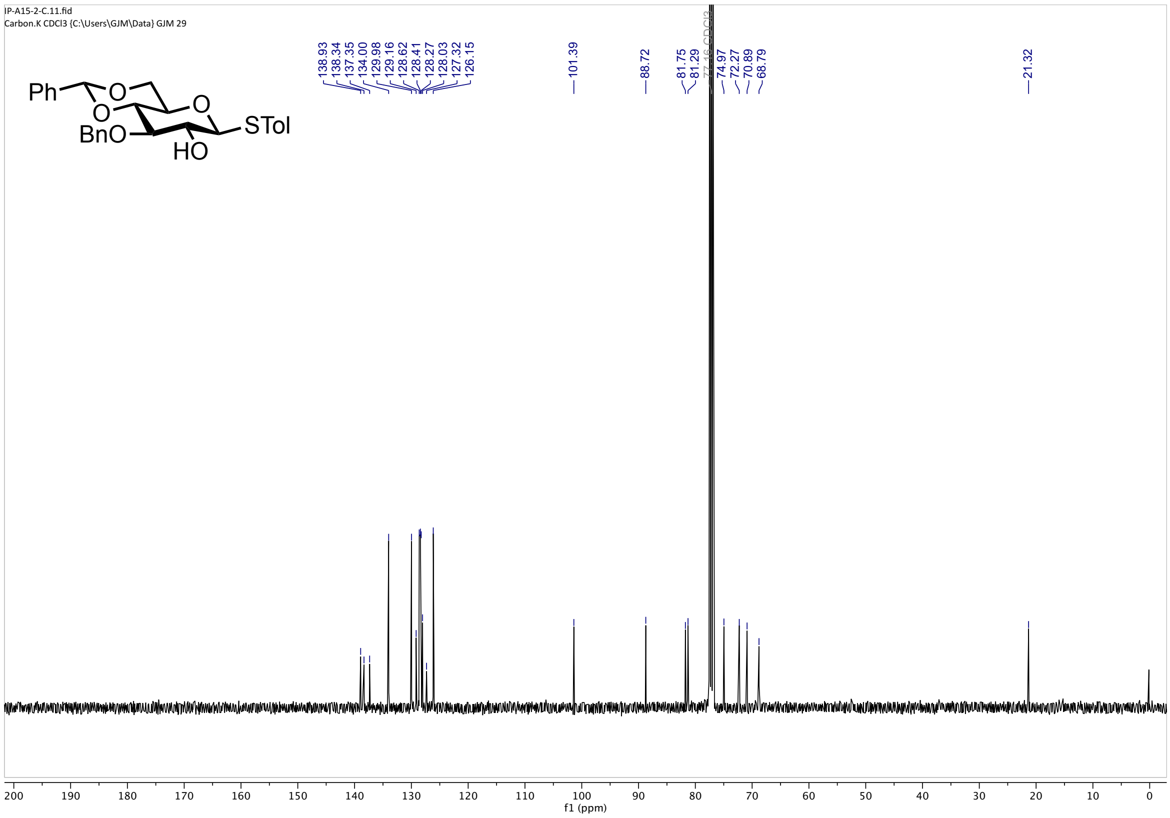

Compound S4a

^1H NMR (400 MHz, Chloroform-*d*) S4a

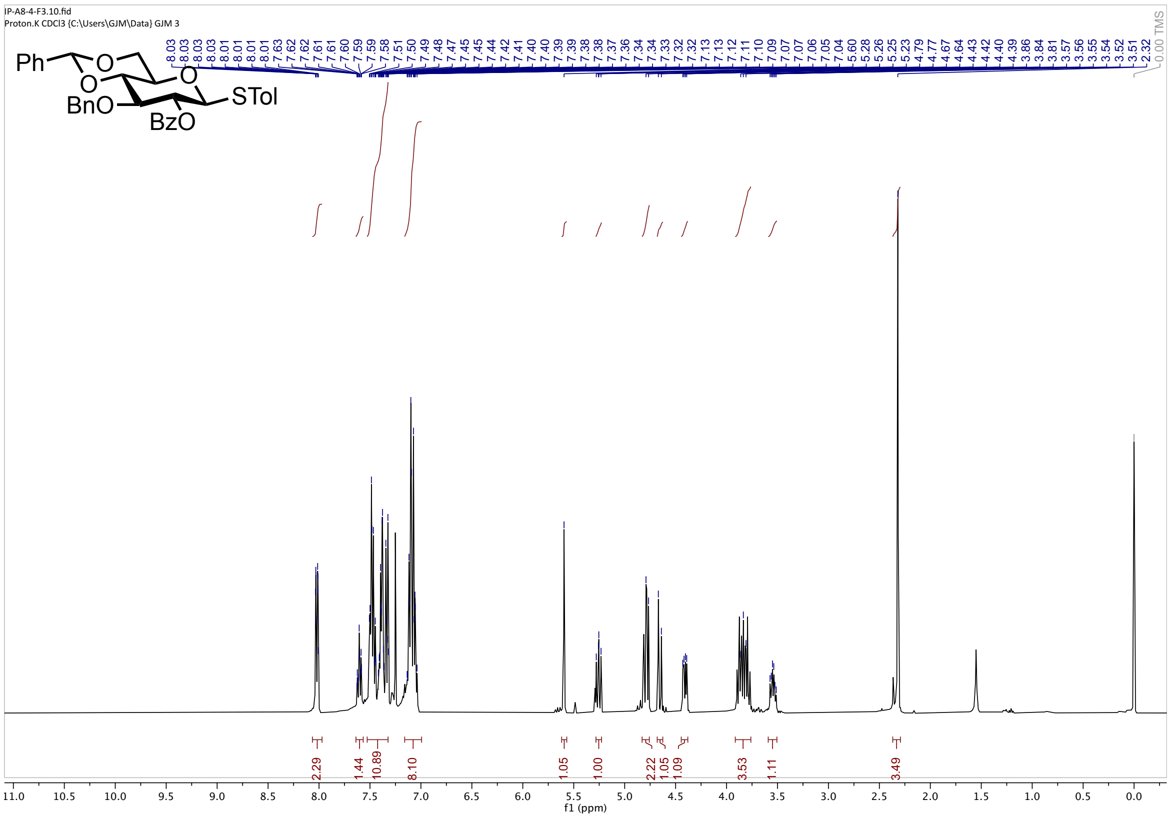

$^{13}C\{^1H\}$ NMR (101 MHz, Chloroform-*d*) S4a

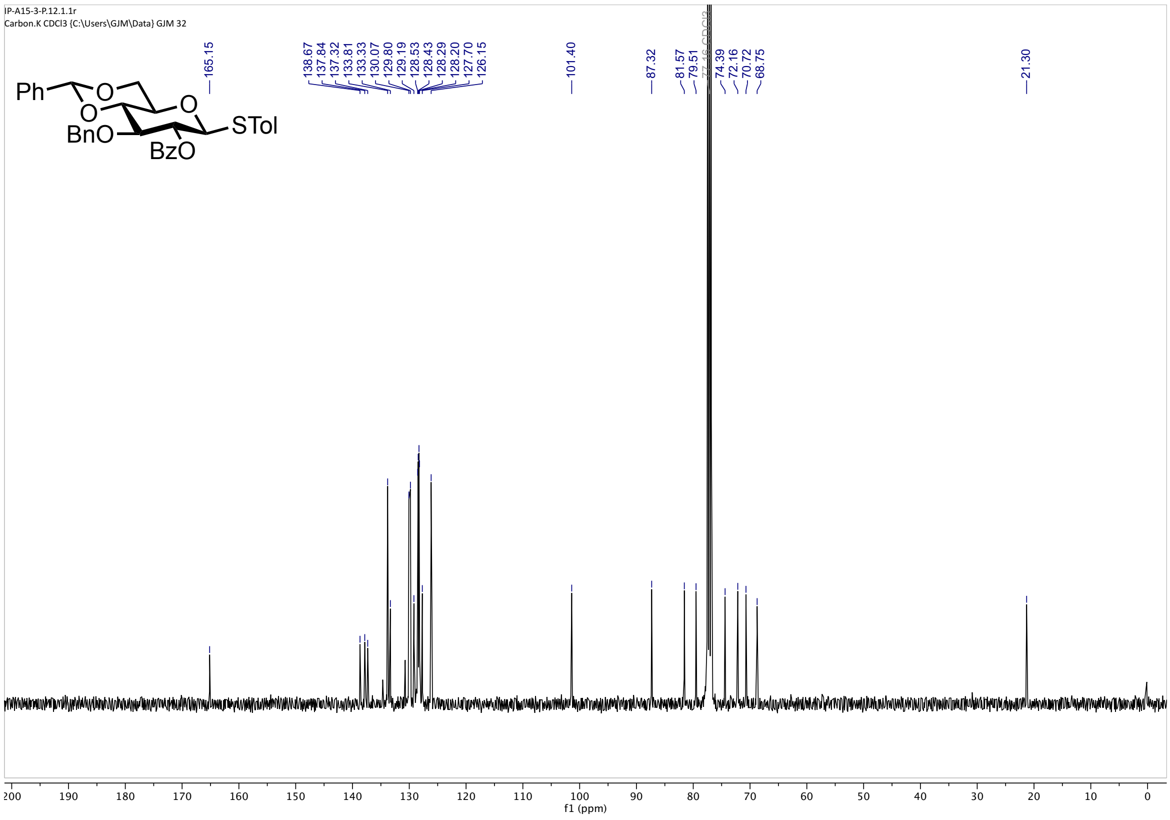

Compound S5a
^1H NMR (400 MHz, Chloroform-*d*) S5a

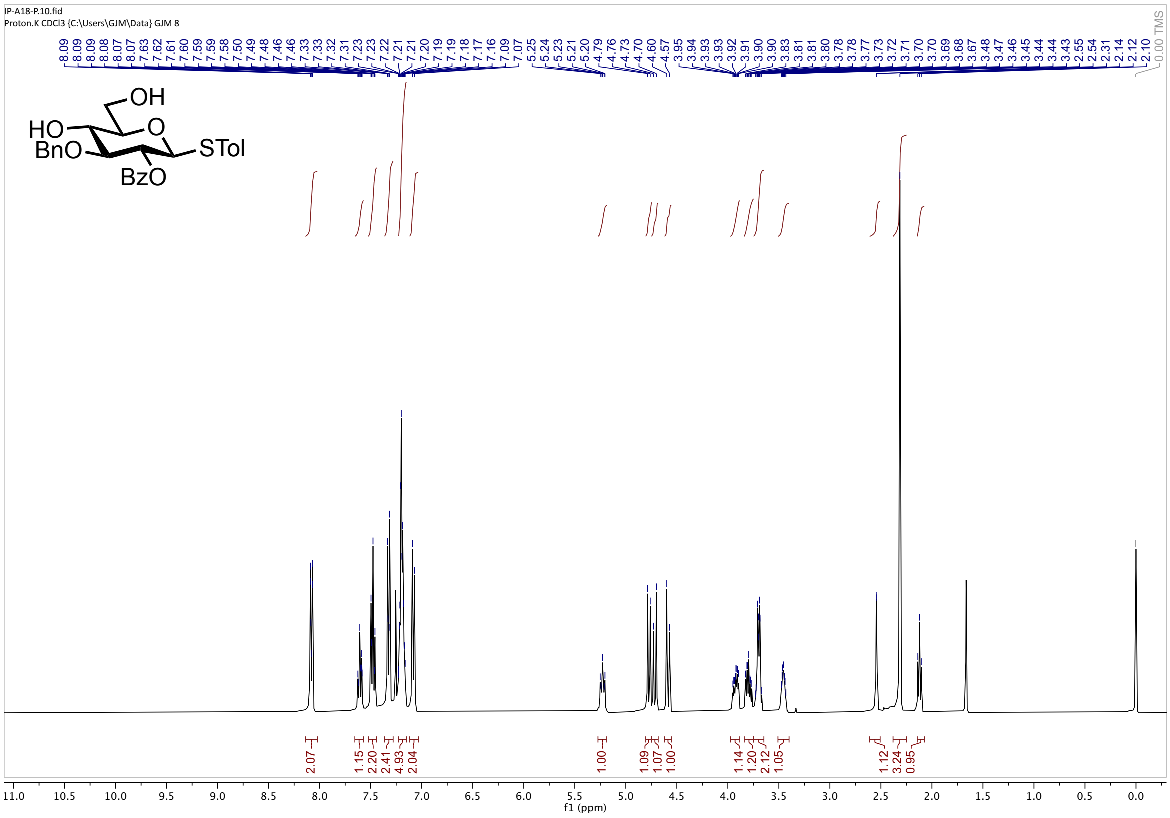

$^{13}C\{^1H\}$ NMR (101 MHz, Chloroform-*d*) S5a

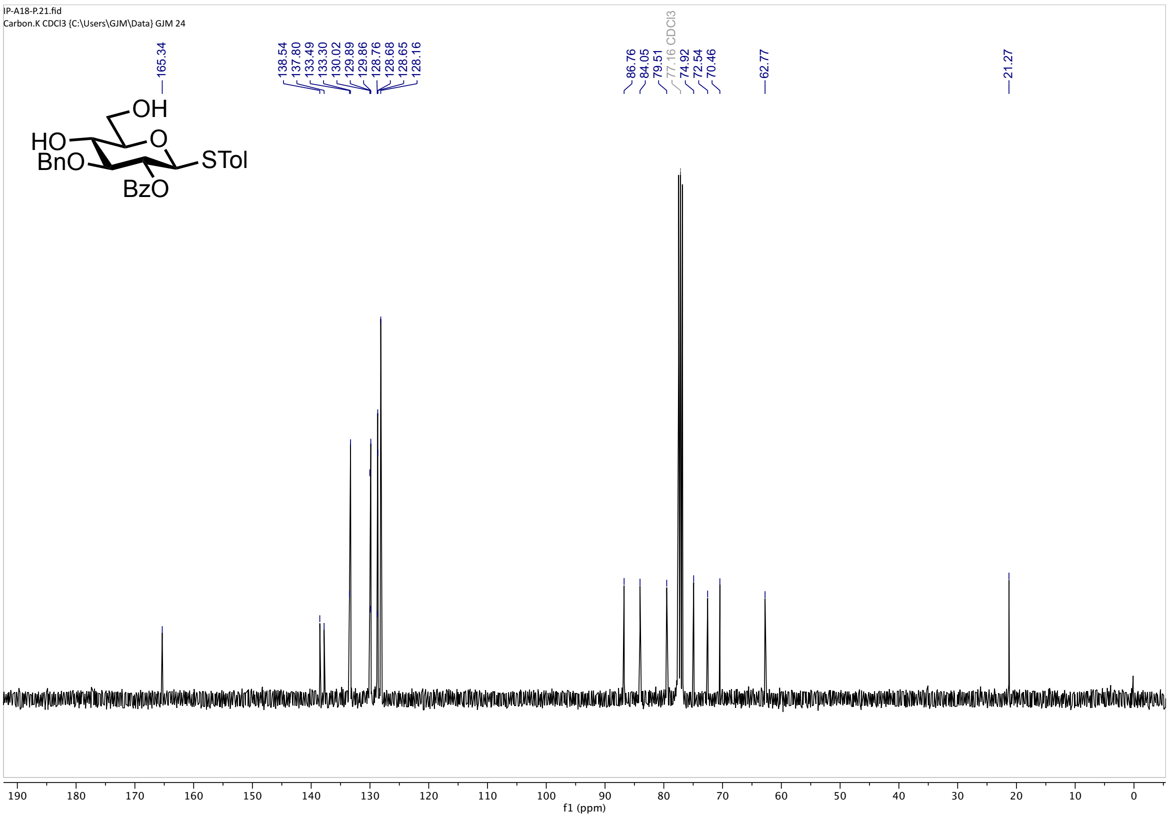

Compound 4a
^1H NMR (400 MHz, Chloroform-*d*) 4a

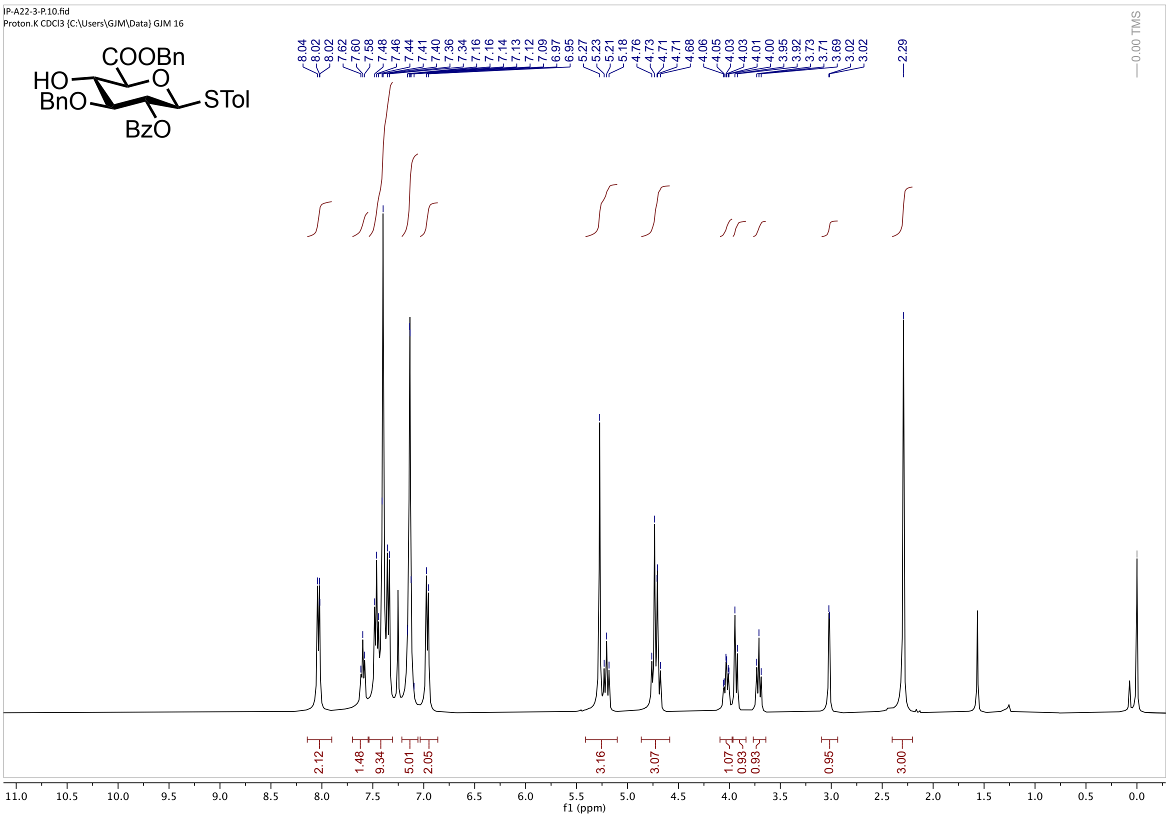

$^{13}C\{^{1}H\}$ NMR (101 MHz, Chloroform-*d*) 4a

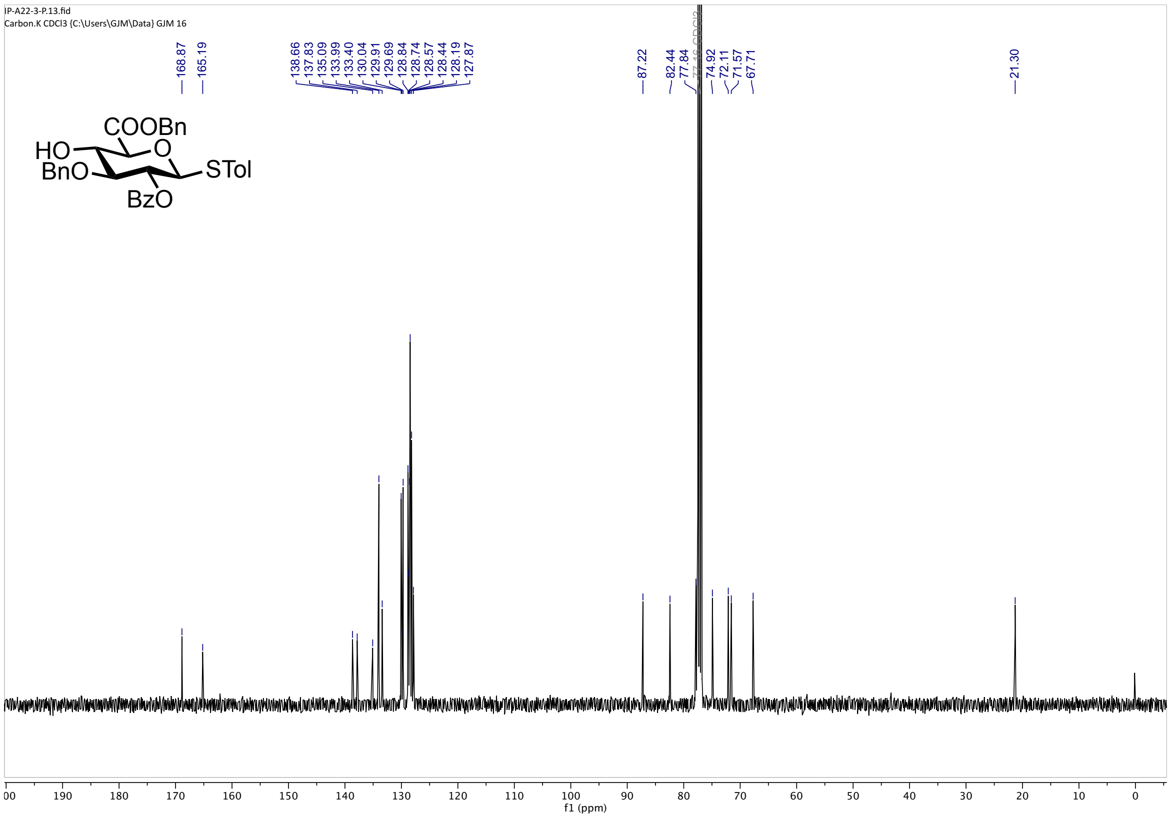

COSY NMR (400 MHz, Chloroform-*d*) 4a

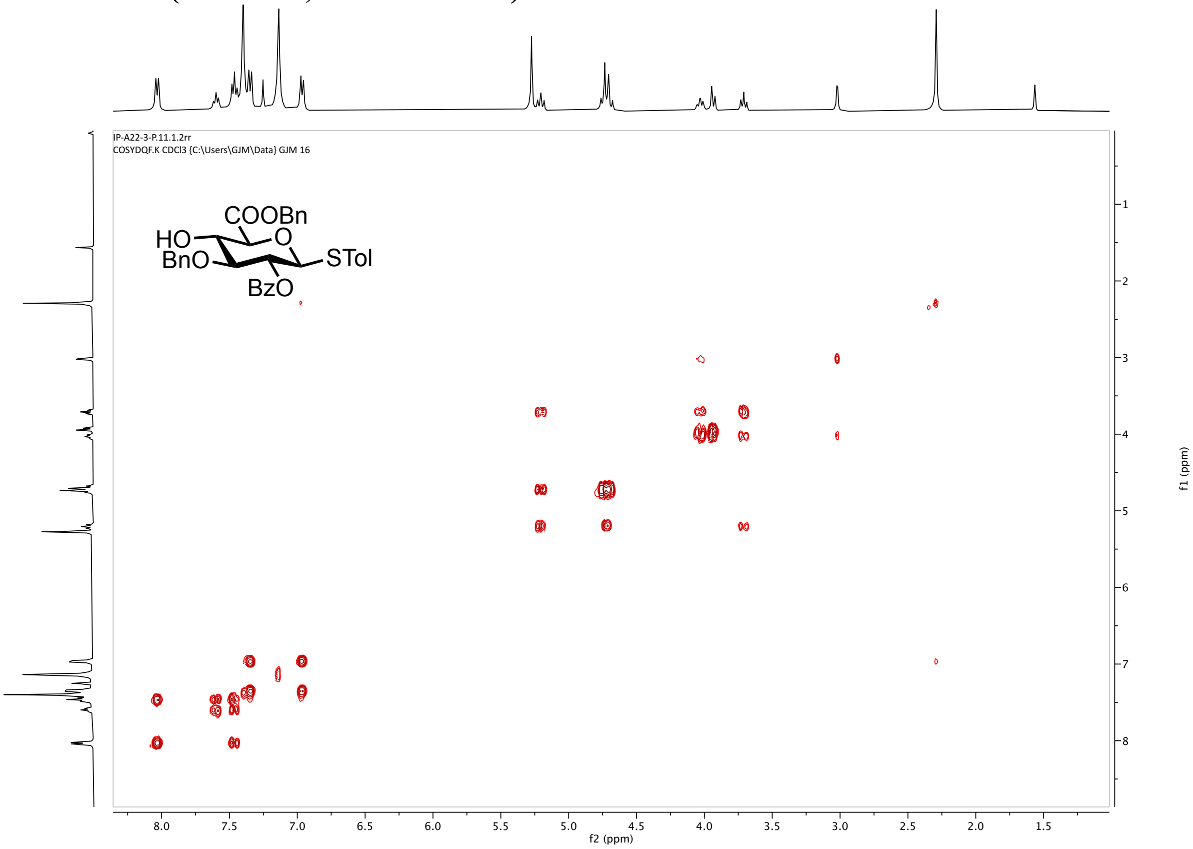

HSQC NMR (400 MHz x 101 MHz, Chloroform-*d*) 4a

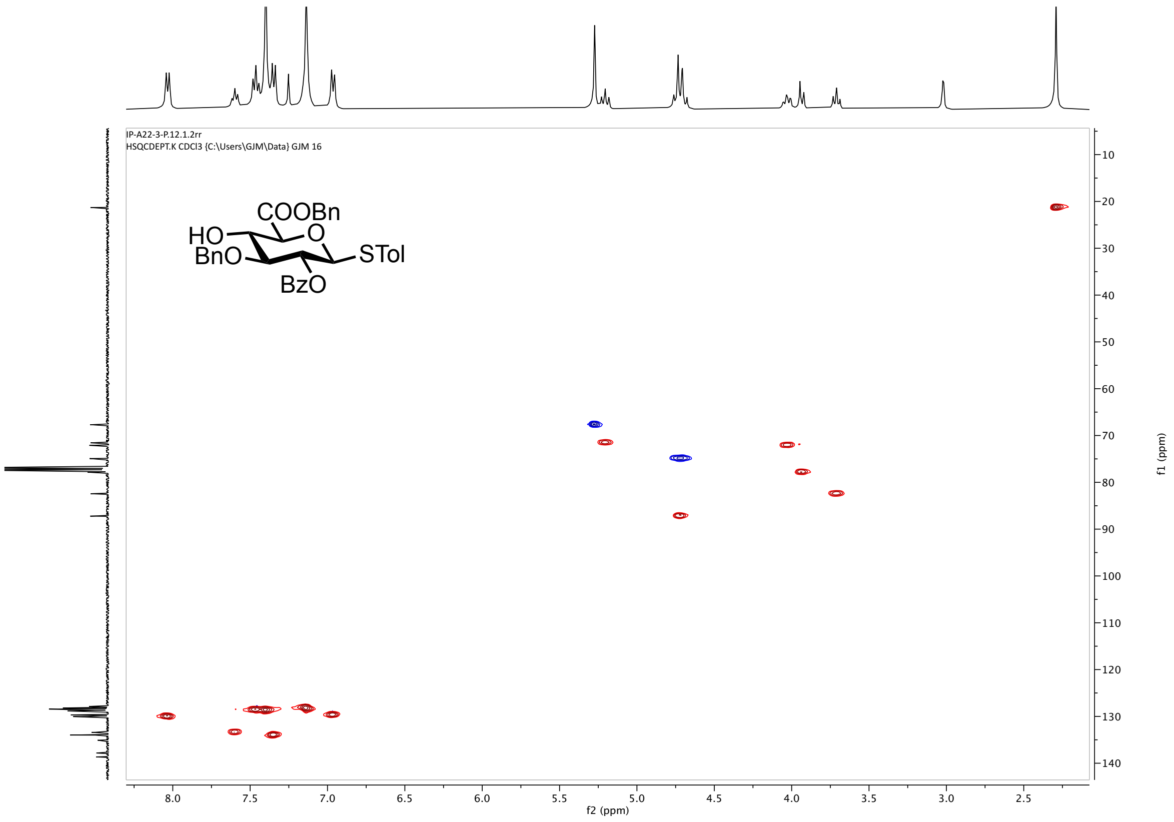

HMBC NMR (400 MHz x 101 MHz, Chloroform-*d*) 4a

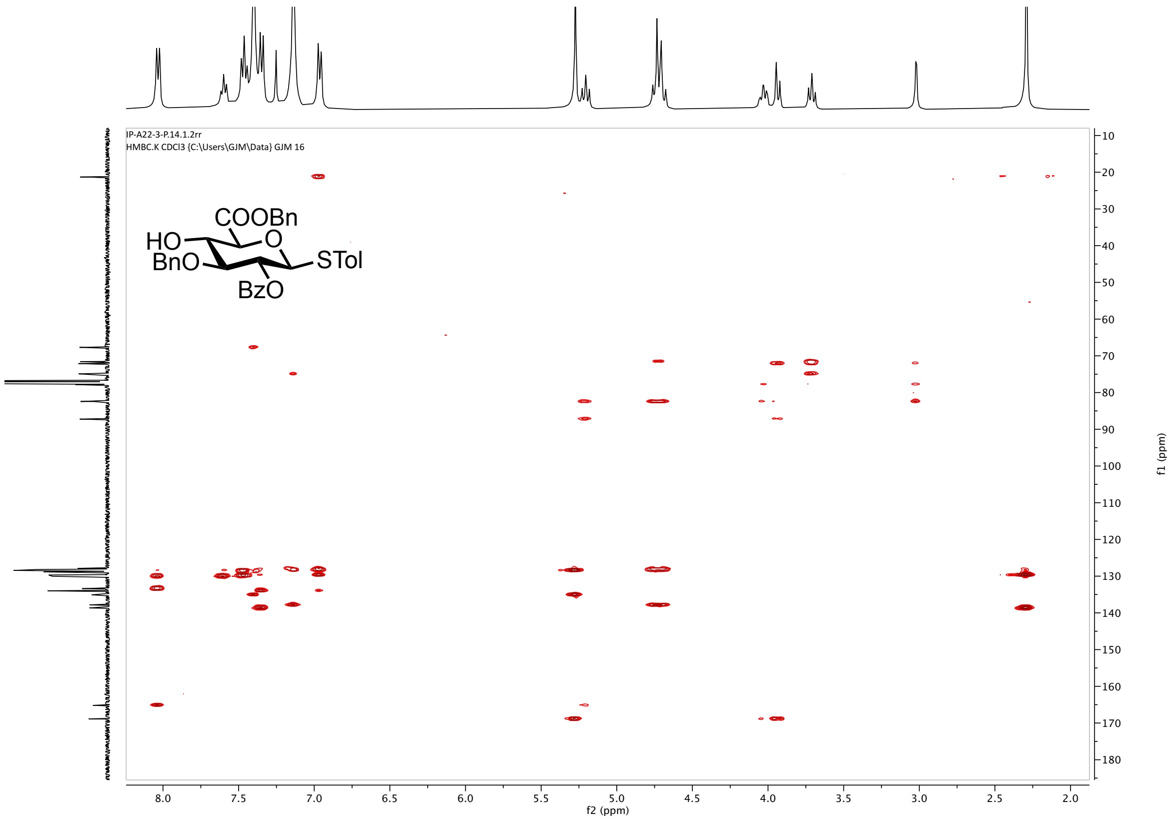

Compound S3b

^{1}H NMR (400 MHz, Chloroform-*d*) S3b

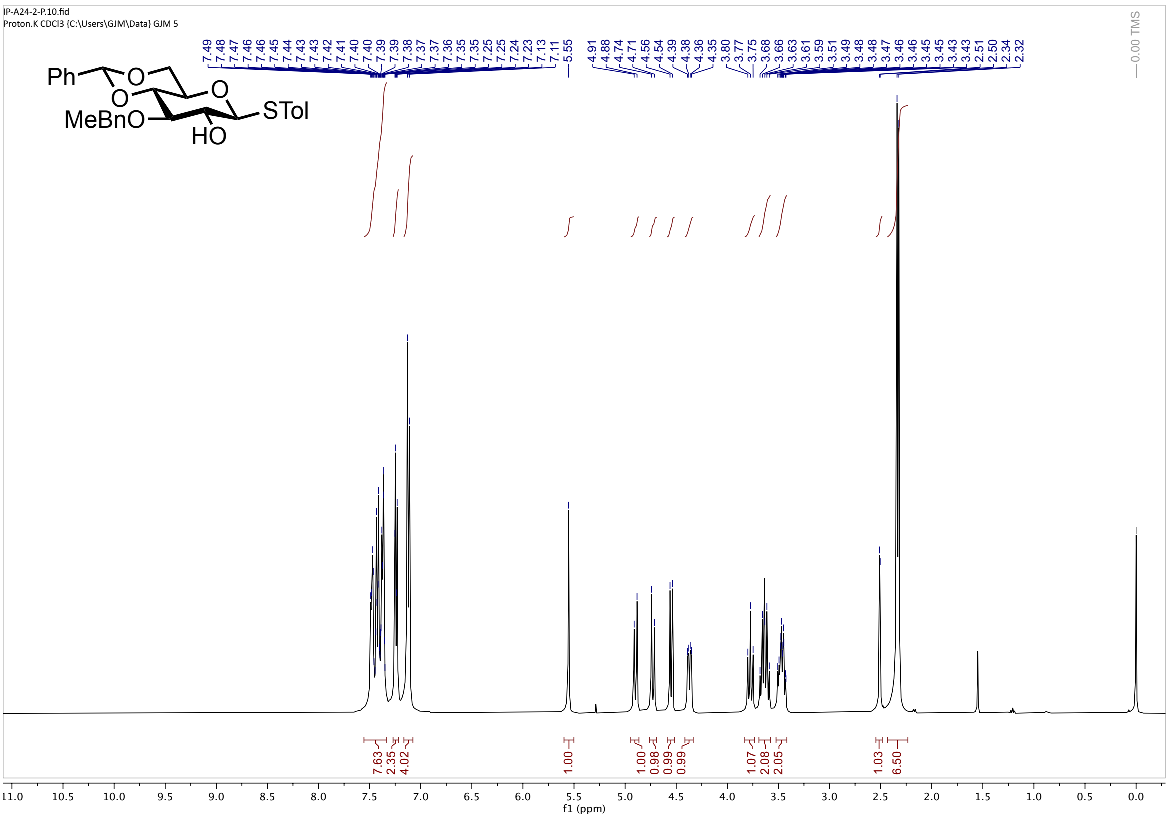

^{13}C{^{1}H} NMR (101 MHz, Chloroform-*d*) S3b

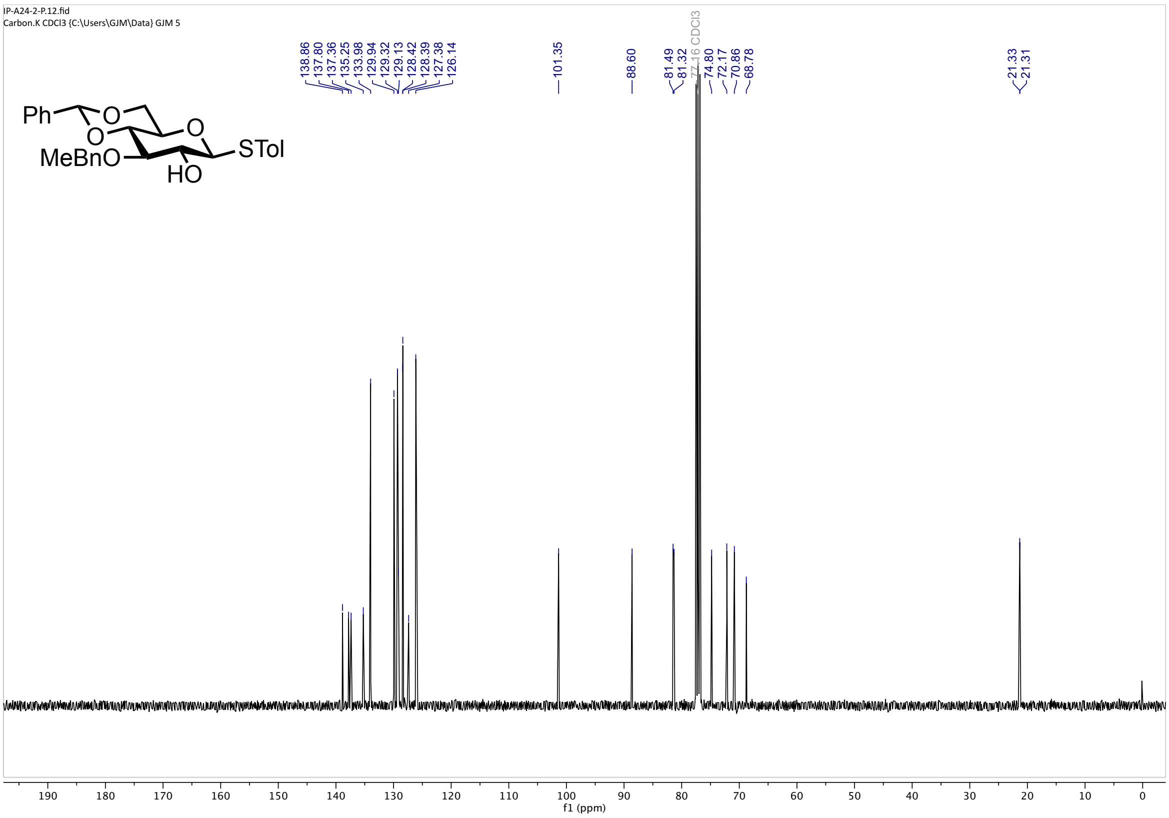

COSY NMR (400 MHz, Chloroform-*d*) S3b

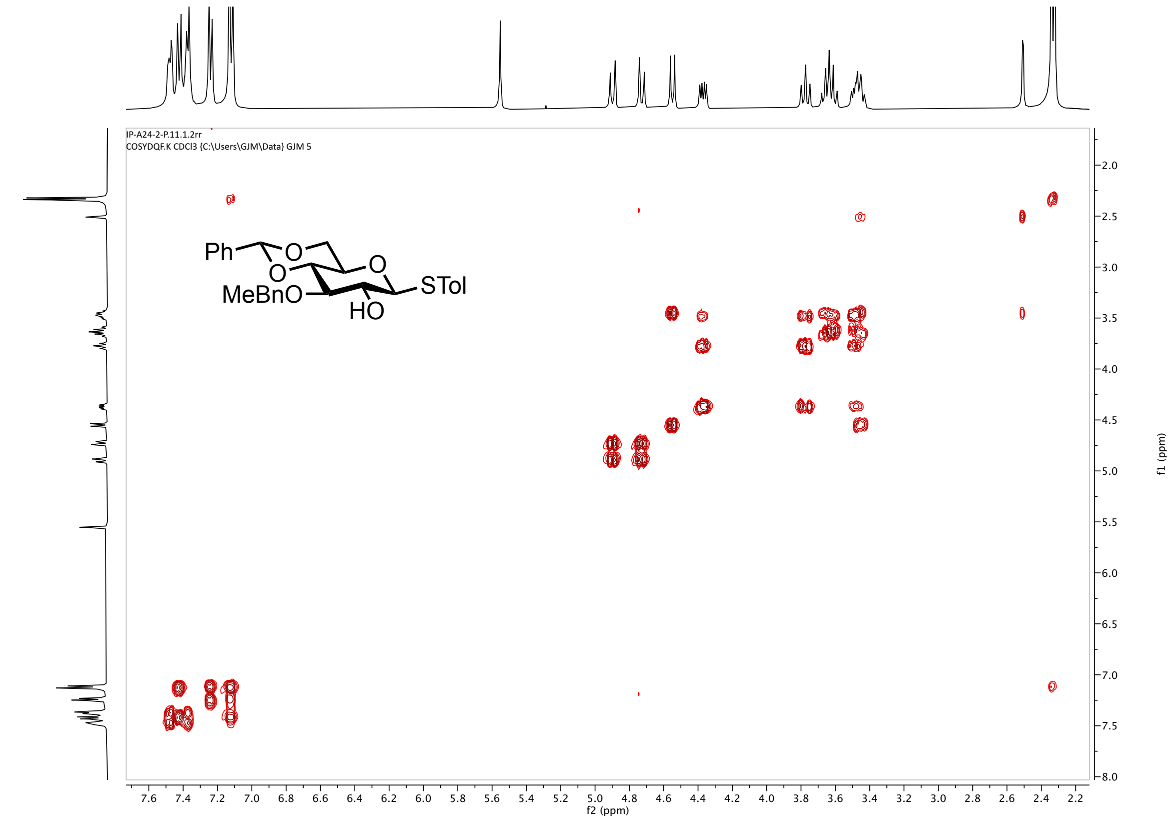

HSQC NMR (400 MHz x 101 MHz, Chloroform-*d*) S3b

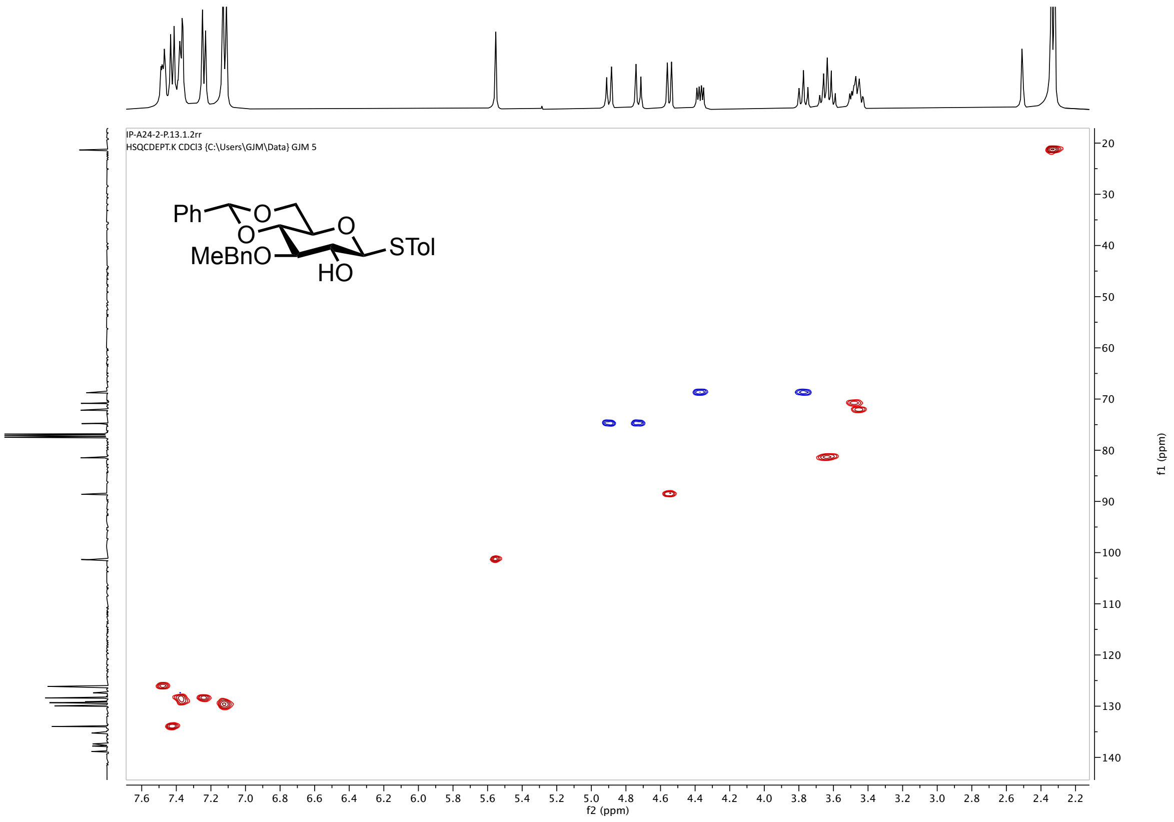

HMBC NMR (400 MHz x 101 MHz, Chloroform-*d*) S3b

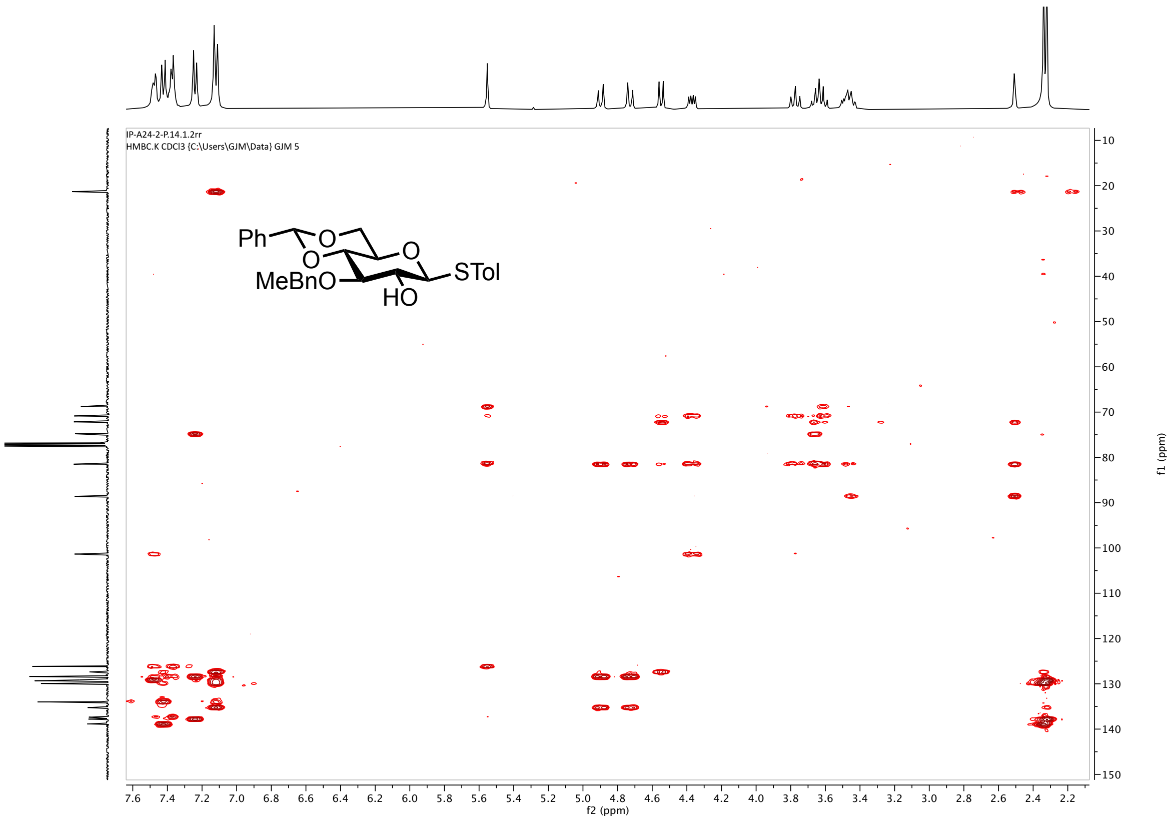

Compound S4b
^{1}H NMR (400 MHz, Chloroform-*d*) S4b

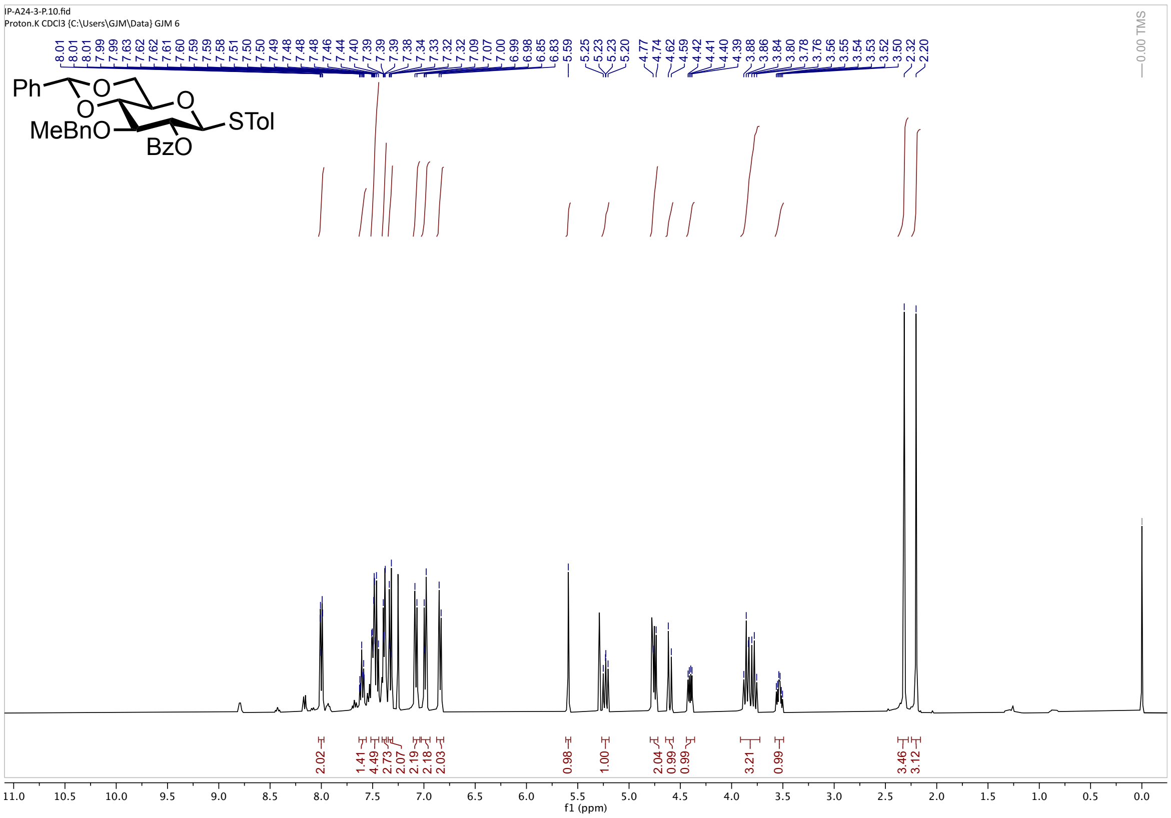

$^{13}C\{^{1}H\}$ NMR (101 MHz, Chloroform-*d*) S4b

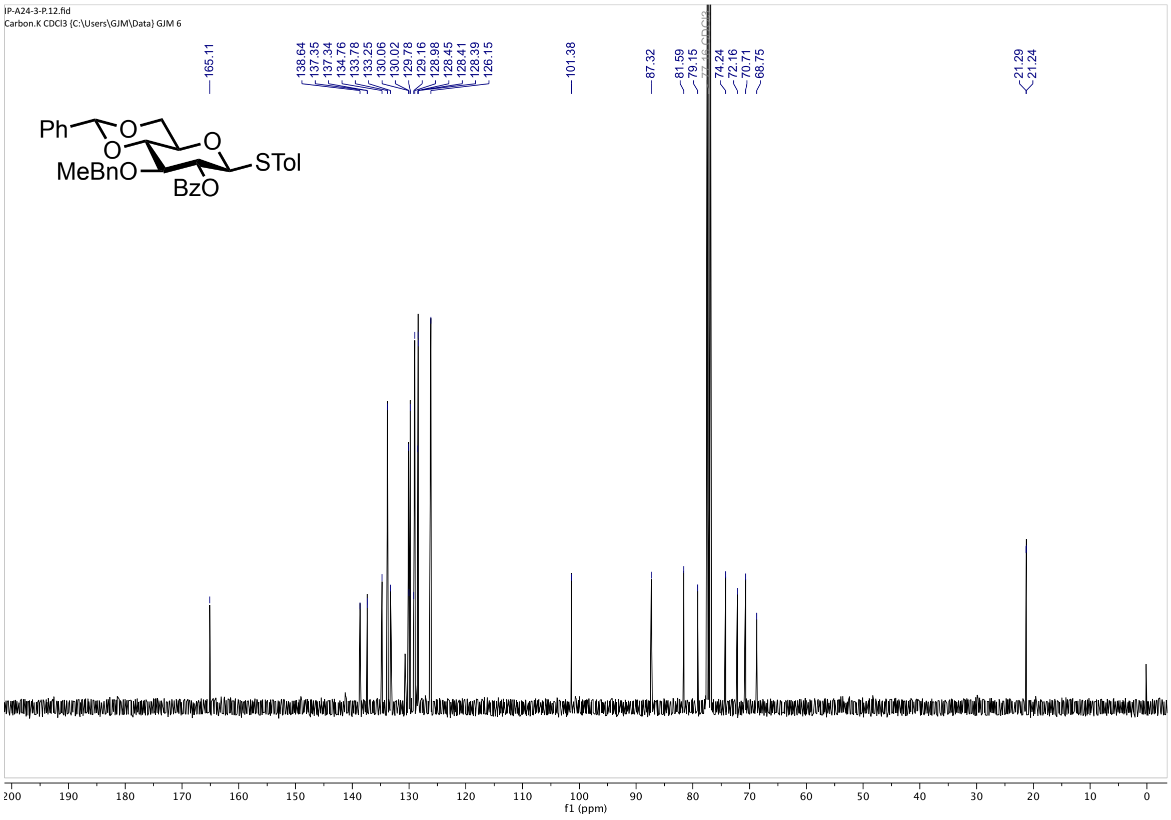

COSY NMR (400 MHz, Chloroform-*d*) S4b

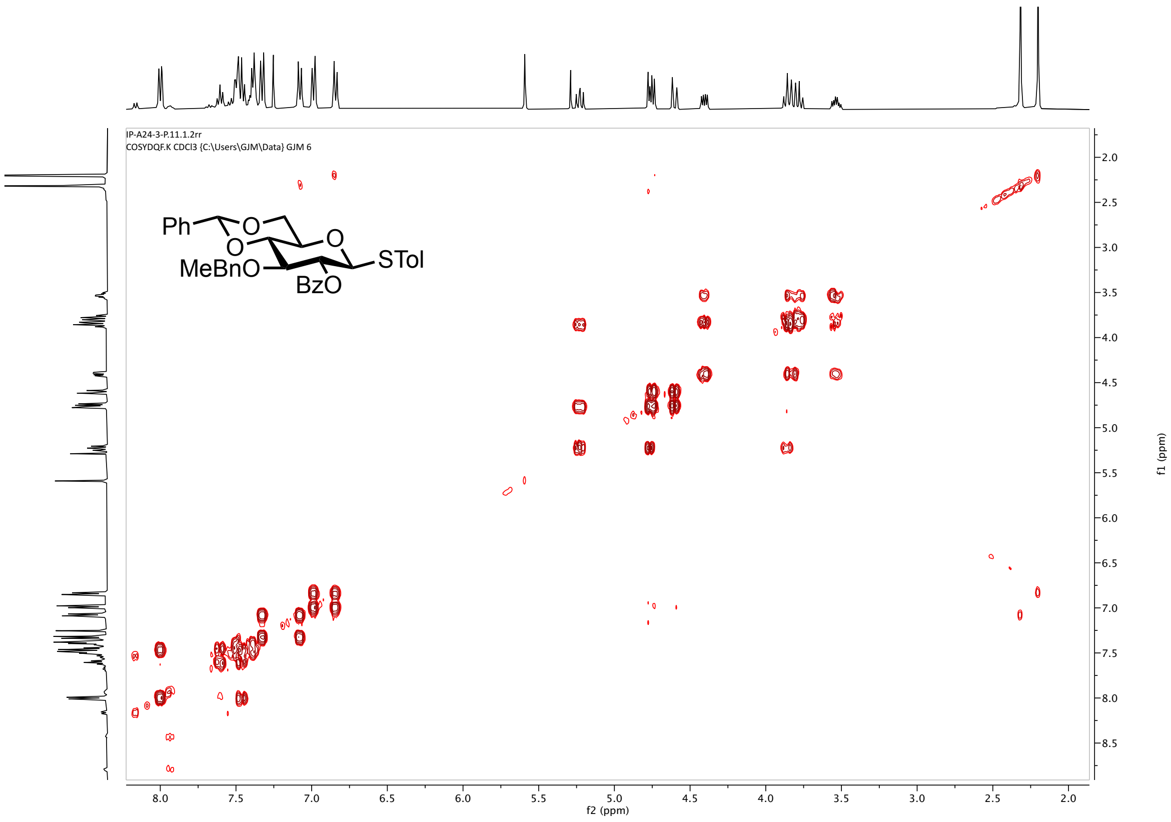

HSQC NMR (400 MHz x 101 MHz, Chloroform-*d*) S4b

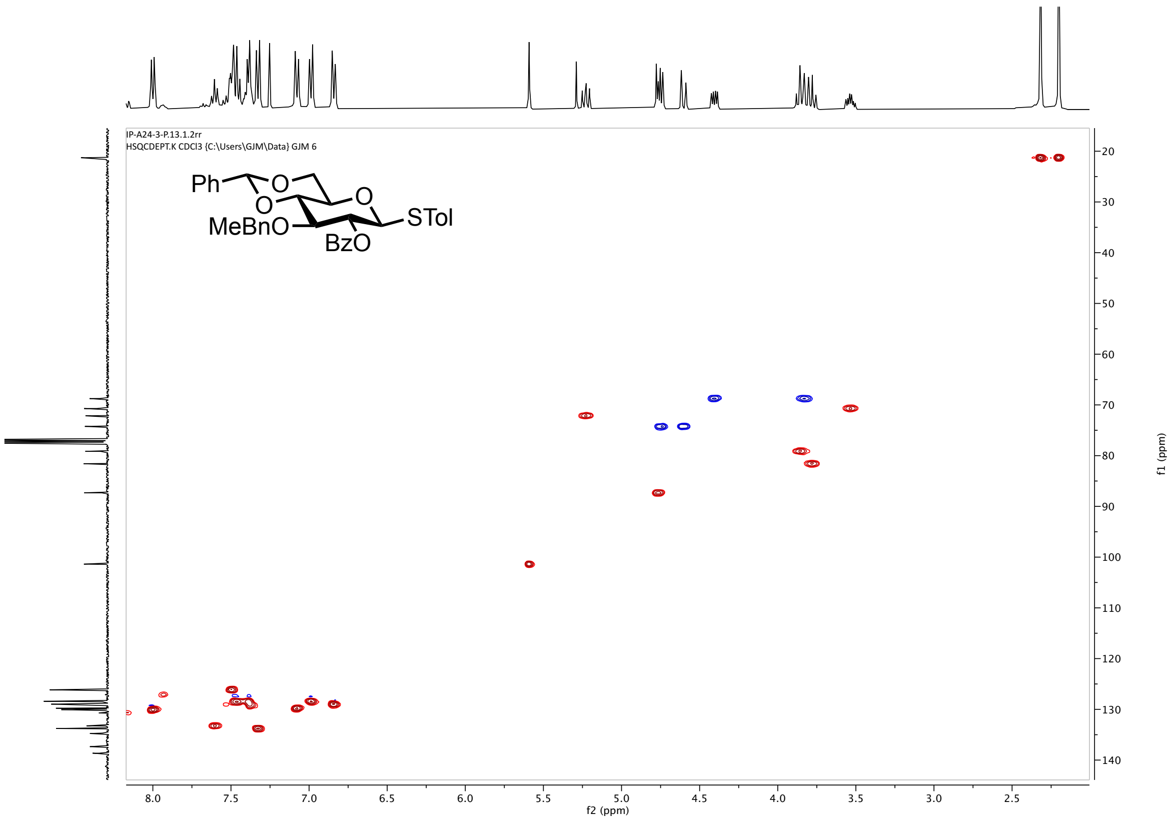

HMBC NMR (400 MHz x 101 MHz, Chloroform-*d*) S4b

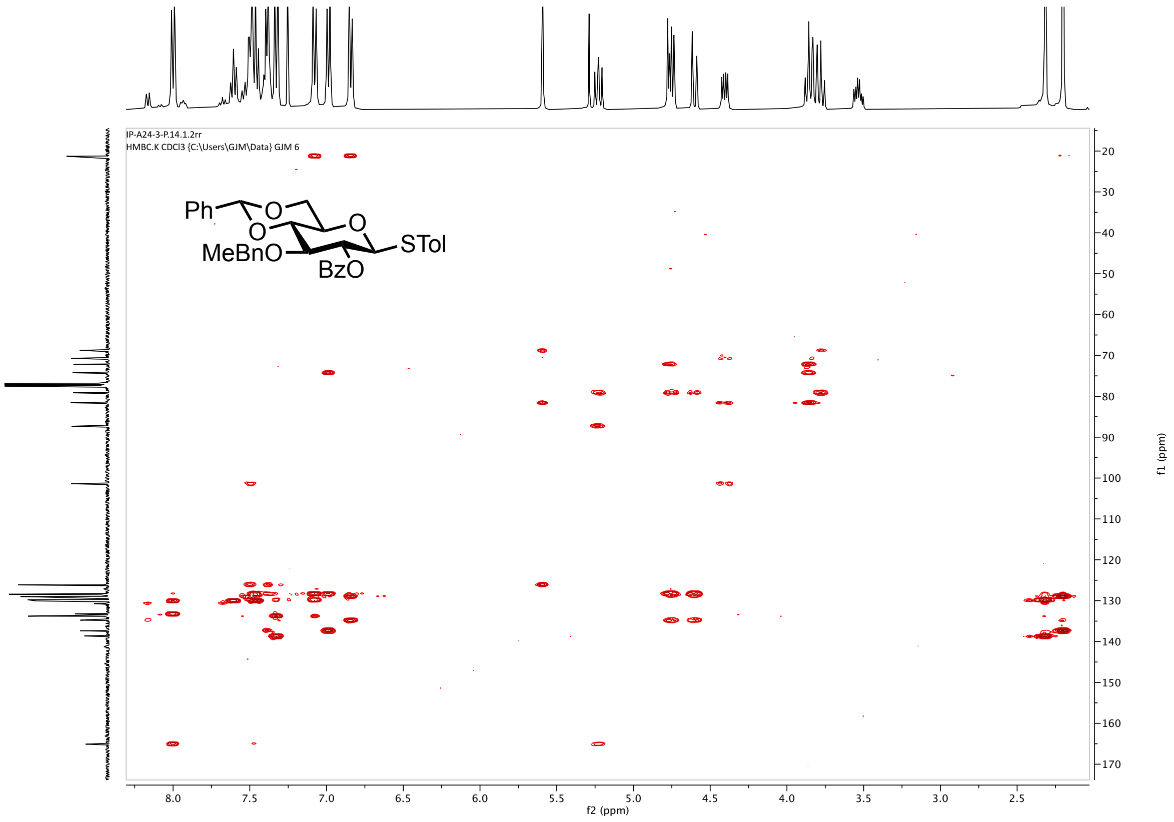

Compound S5b

^{1}H NMR (400 MHz, Chloroform-*d*) S5b

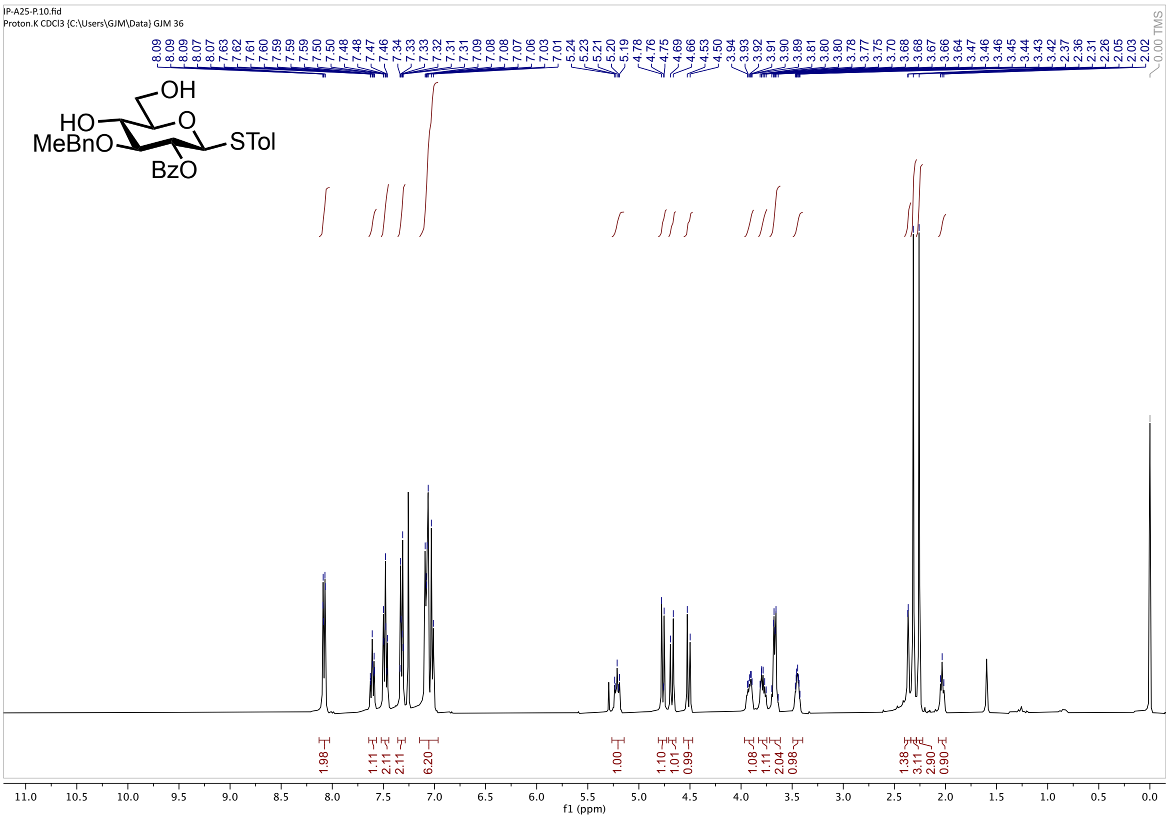

^{13}C{^{1}H} NMR (101 MHz, Chloroform-*d*) S5b

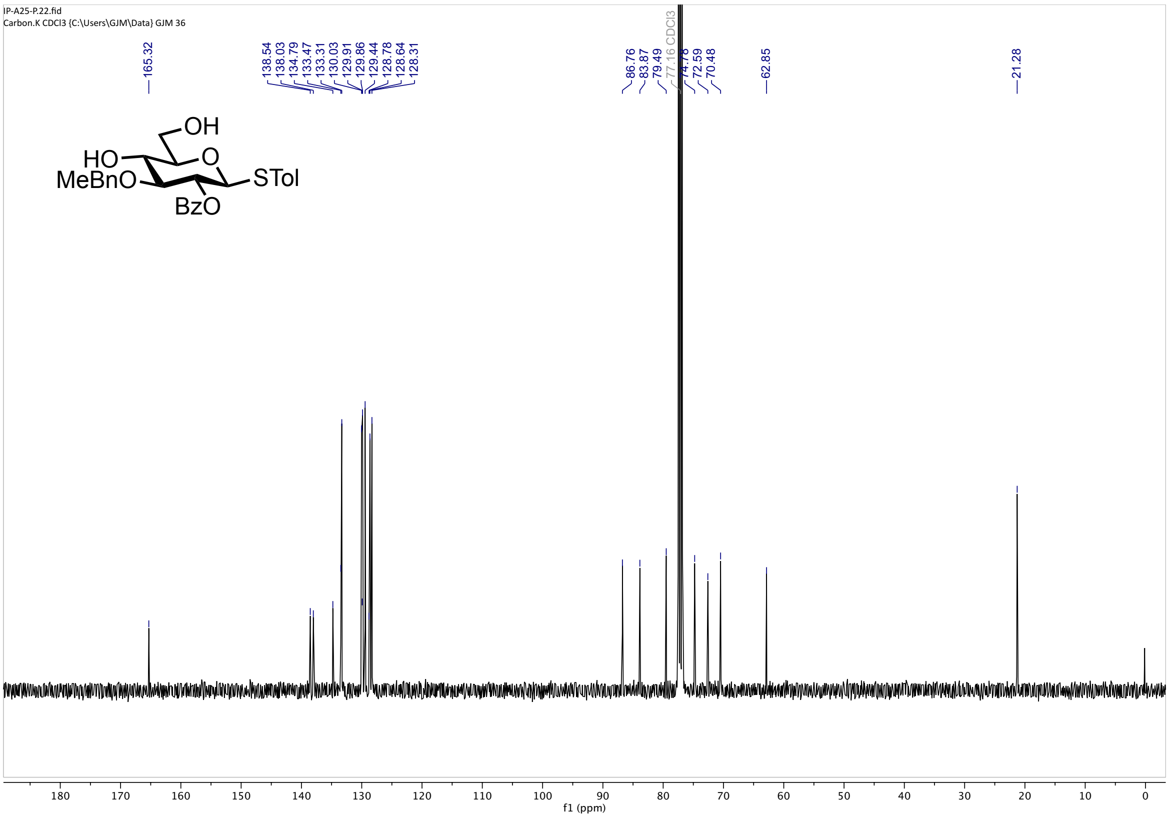

COSY NMR (400 MHz, Chloroform-*d*) S5b

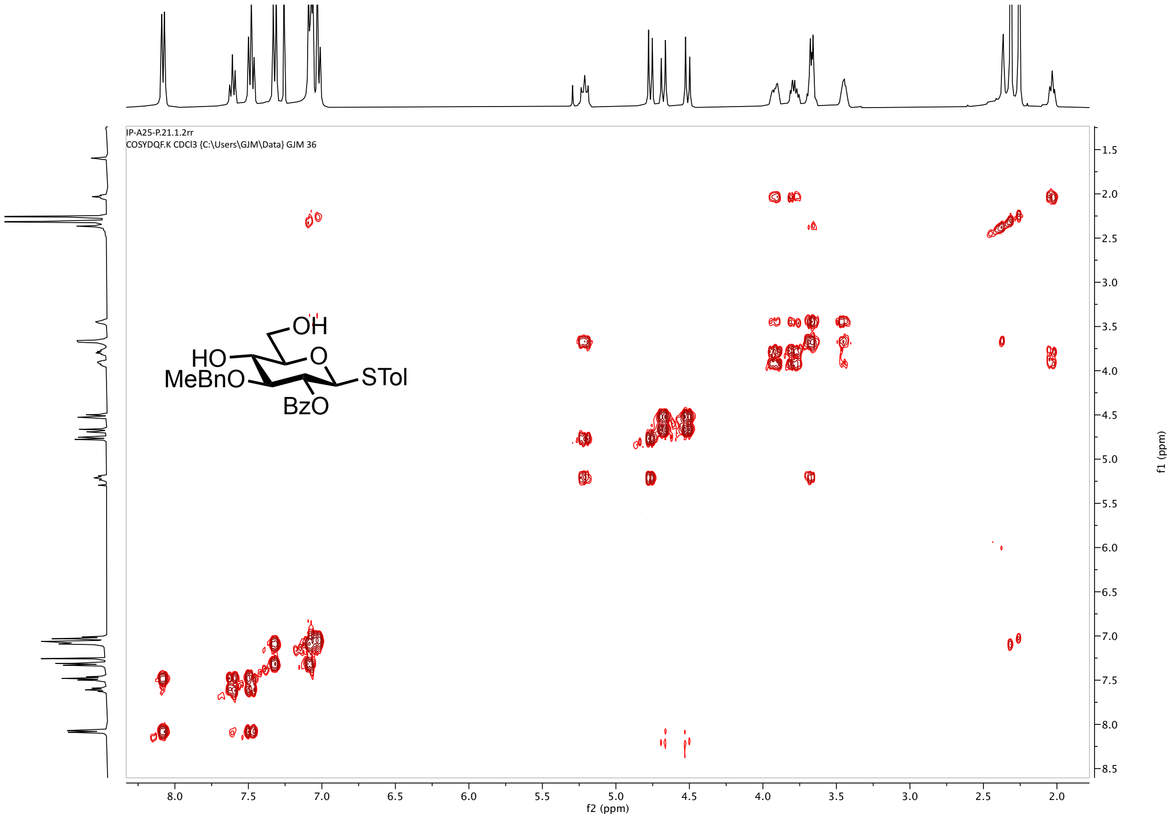

HSQC NMR (400 MHz x 101 MHz, Chloroform-*d*) S5b

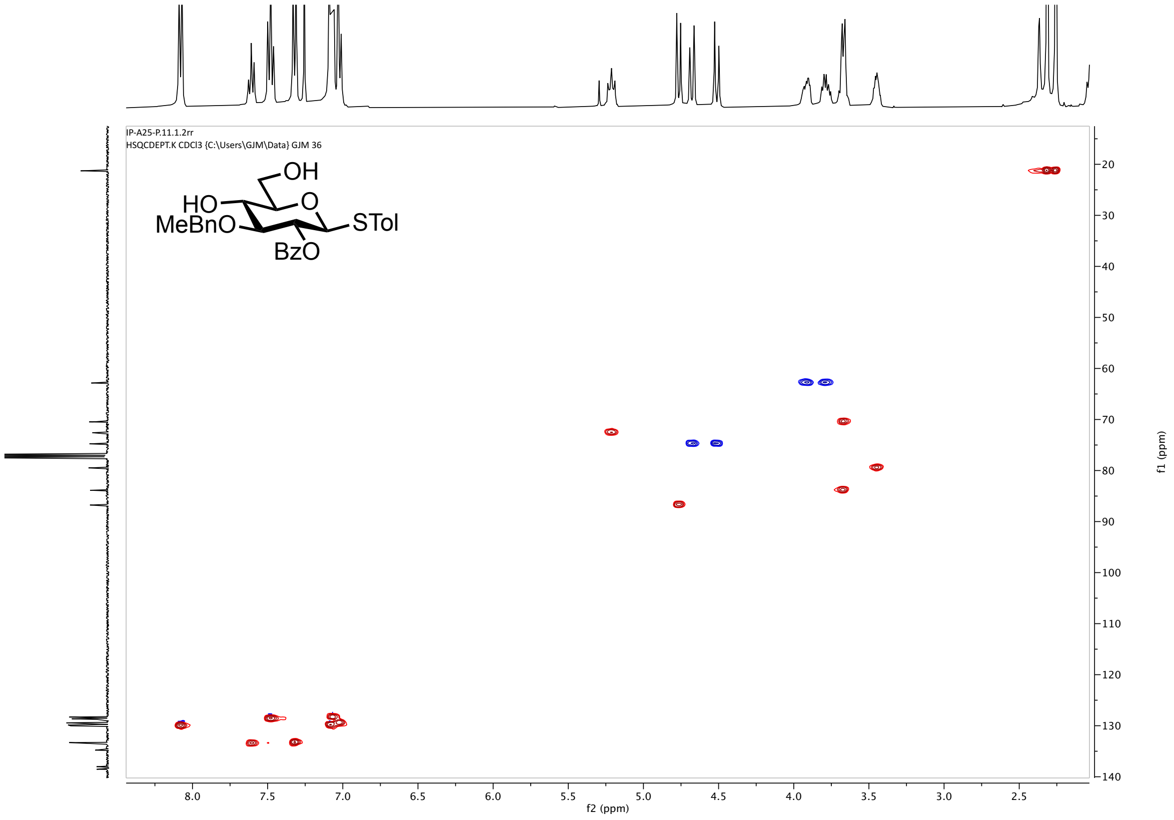

HMBC NMR (400 MHz x 101 MHz, Chloroform-*d*) S5b

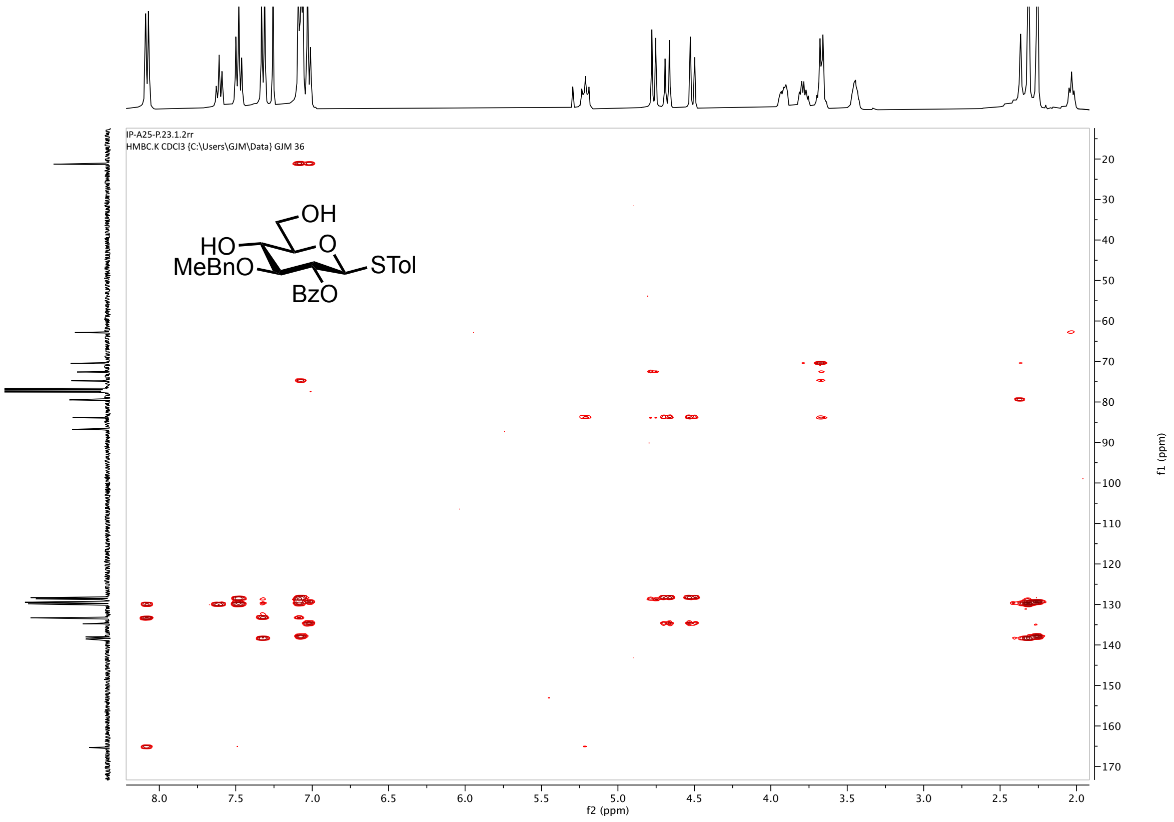

Compound 4b
^{1}H NMR (400 MHz, Chloroform-*d*) 4b

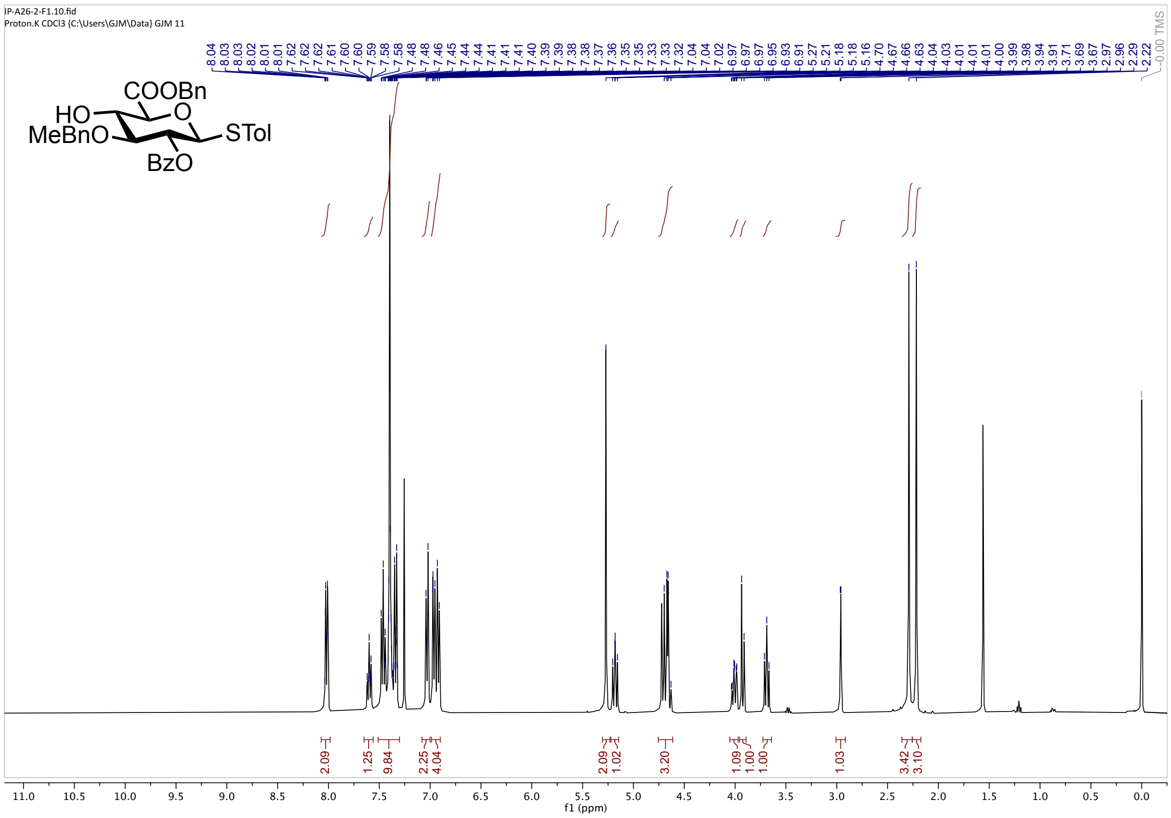

$^{13}C\{^1H\}$ NMR (101 MHz, Chloroform-*d*) 4b

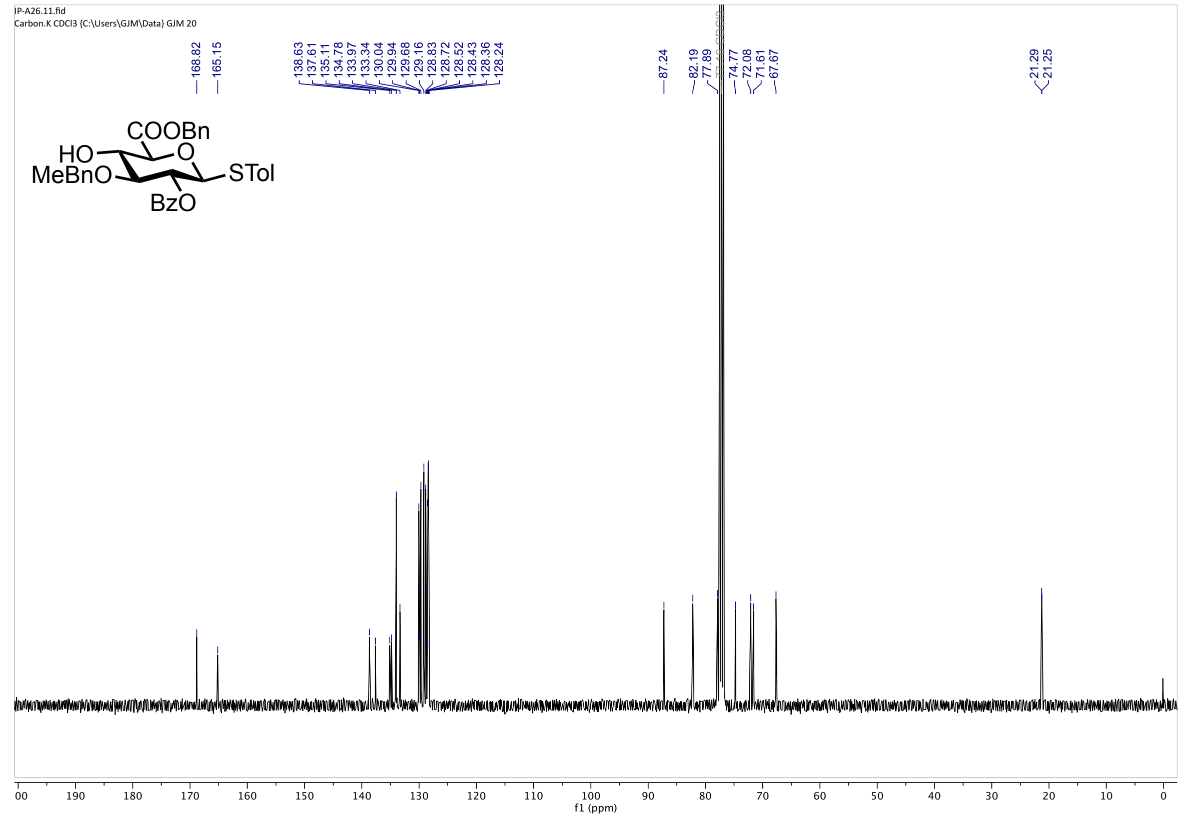

COSY NMR (400 MHz, Chloroform-*d*) 4b

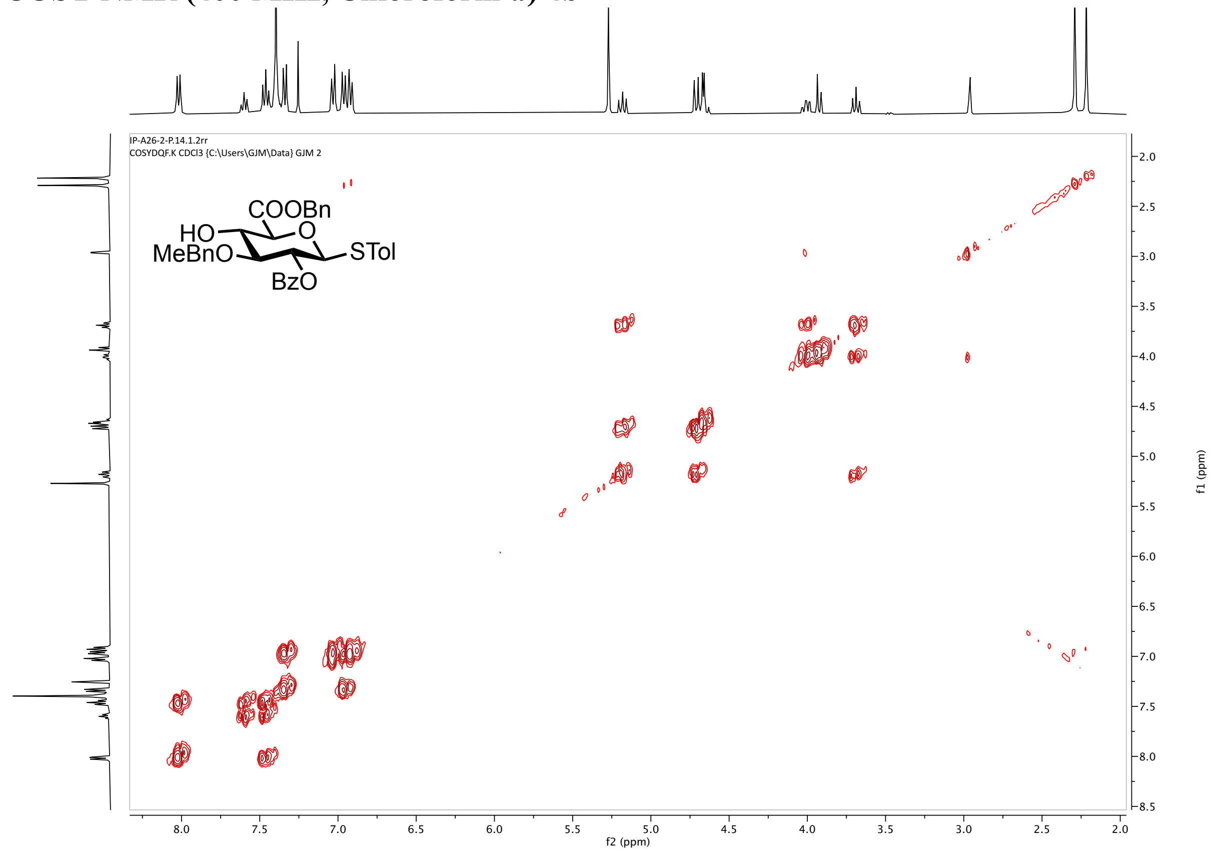

HSQC NMR (400 MHz x 101 MHz, Chloroform-*d*) 4b

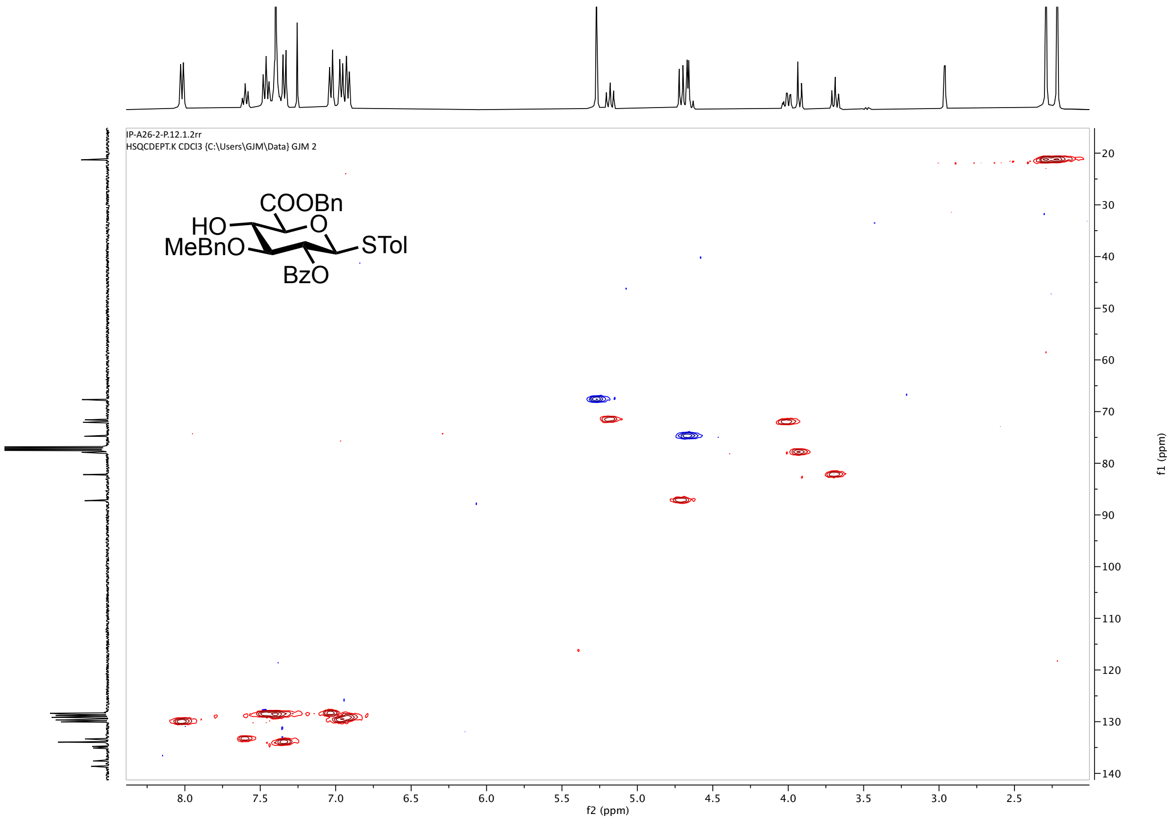

HMBC NMR (400 MHz x 101 MHz, Chloroform-*d*) 4b

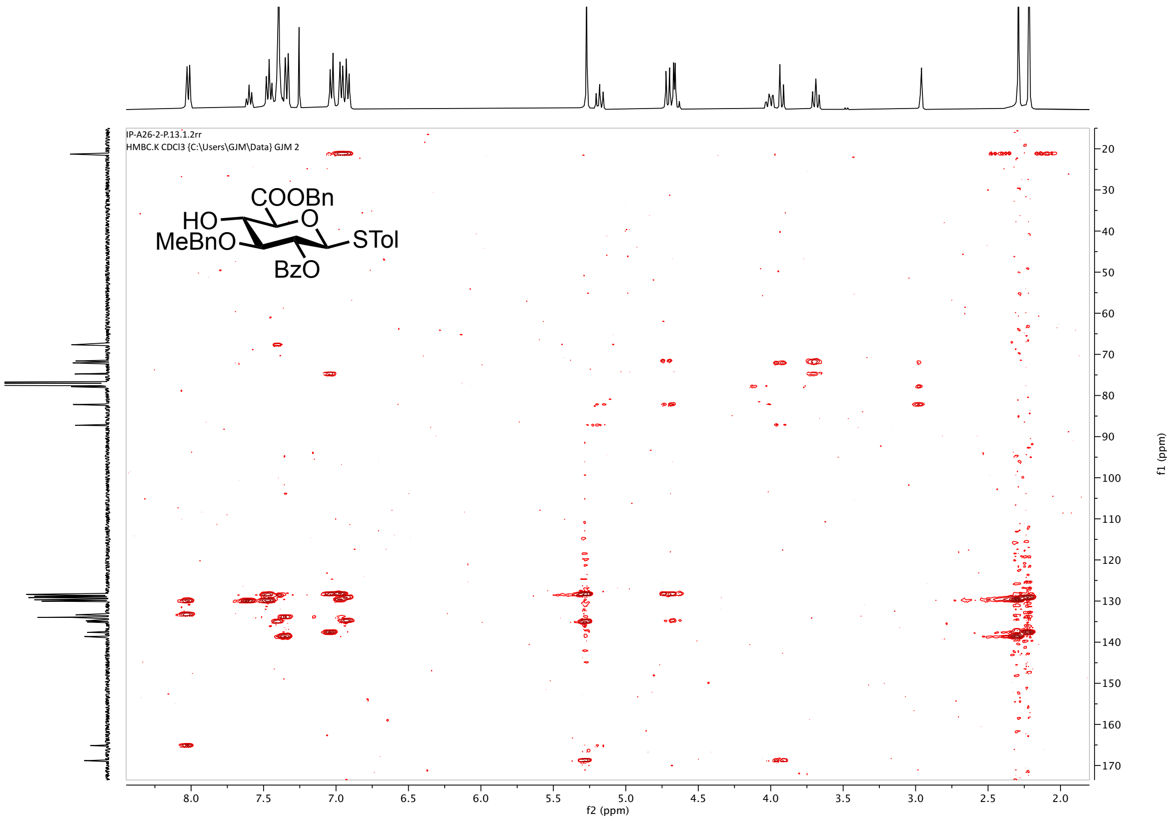

Compound S6a
^{1}H NMR (400 MHz, Chloroform-*d*) S6a

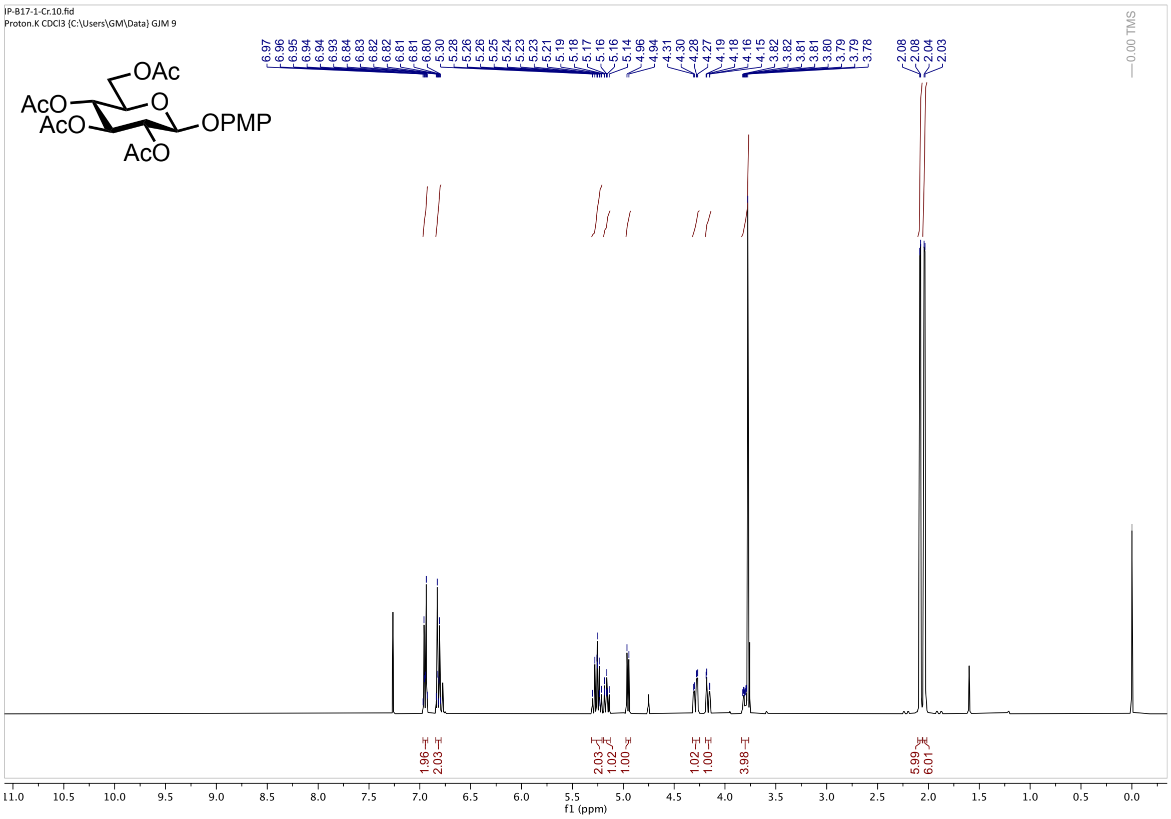

Compound S7
^{1}H NMR (400 MHz, DMSO) S7

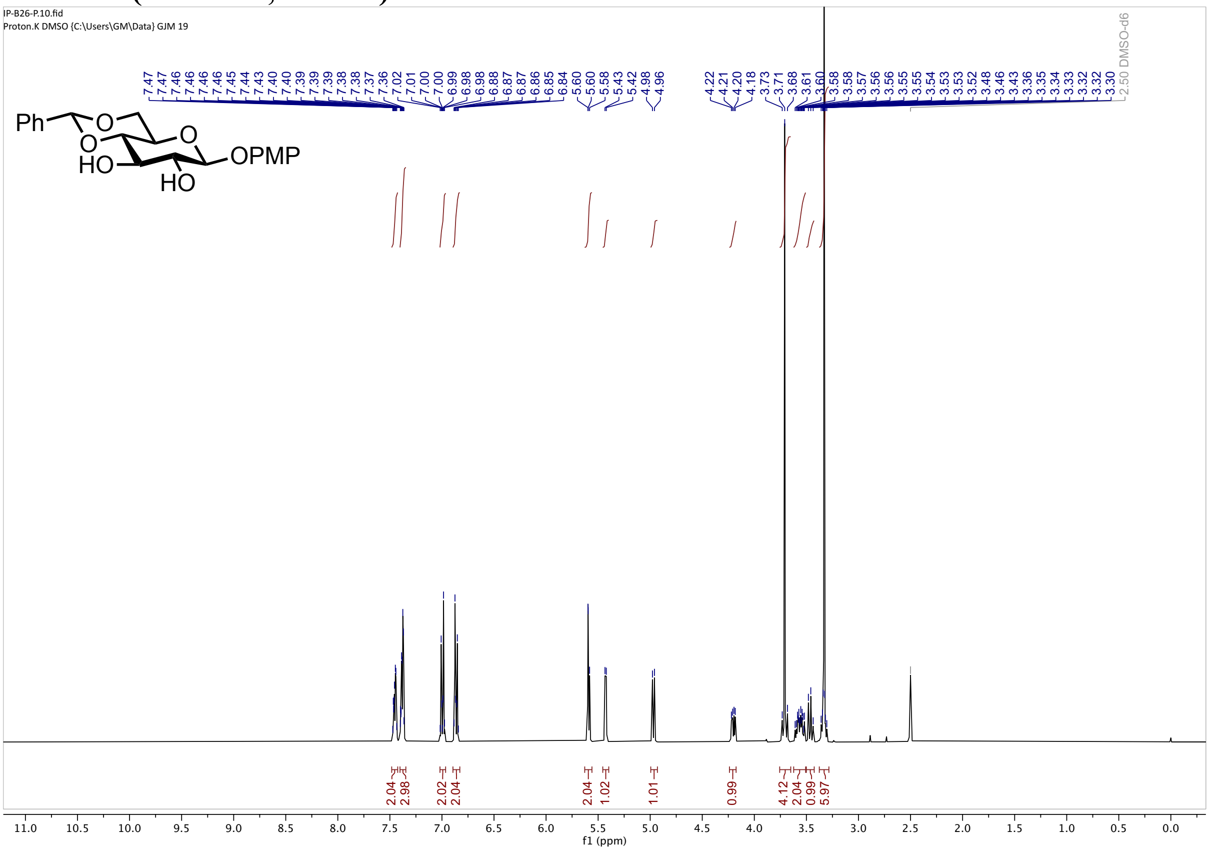

$^{13}C\{^{1}H\}$ NMR (101 MHz, DMSO) S7

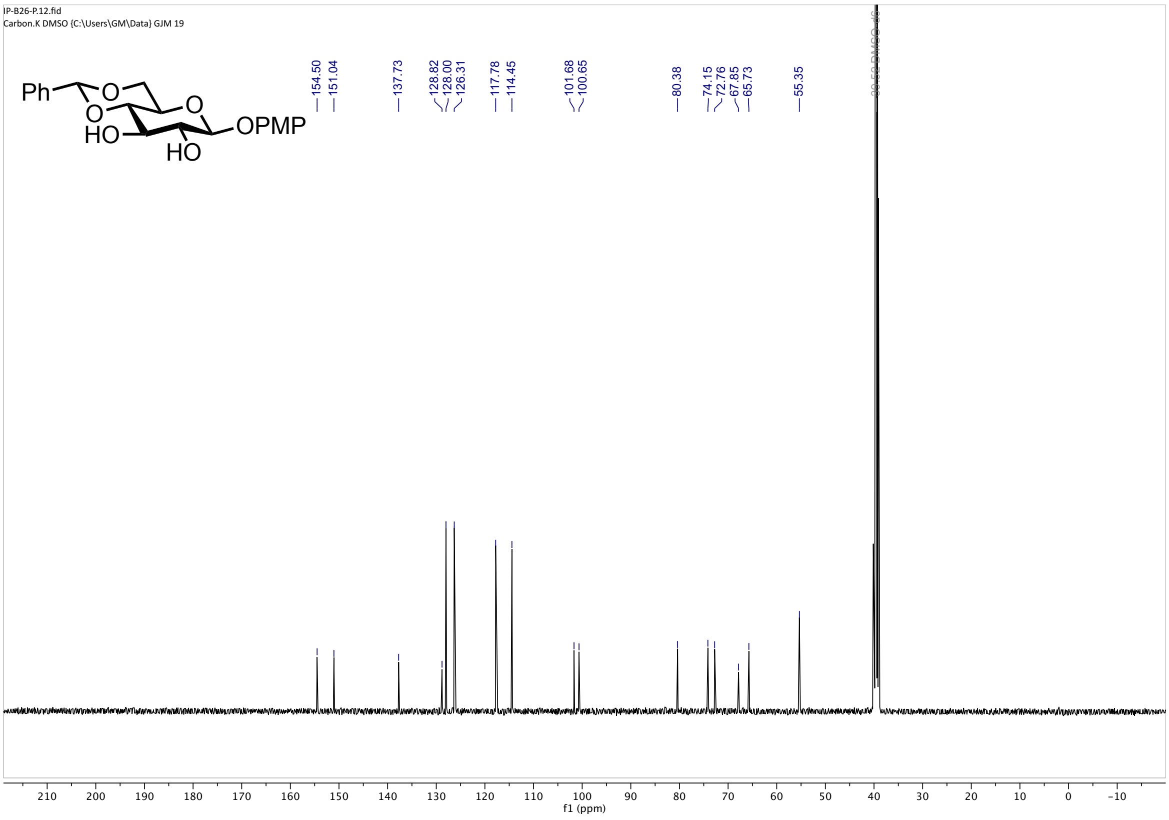

Compound S8

^{1}H NMR (400 MHz, Chloroform-*d*) S8

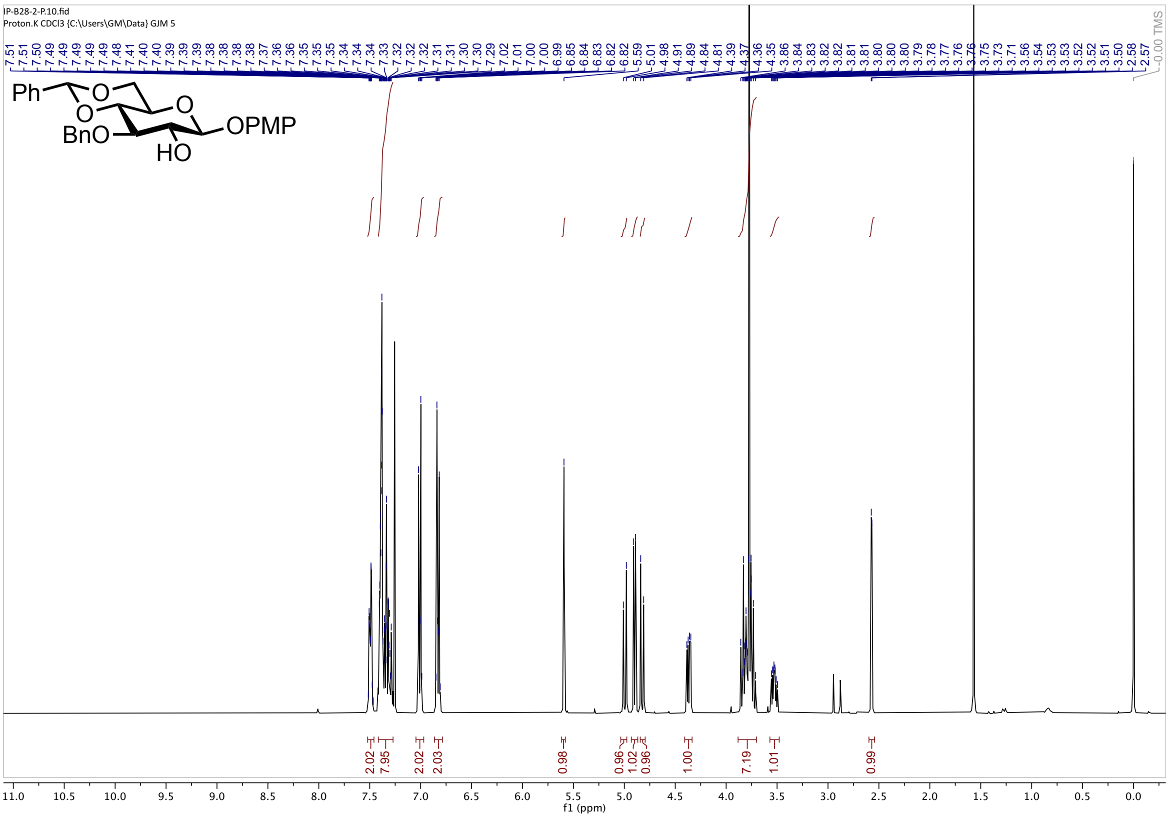

$^{13}C\{^1H\}$ NMR (101 MHz, Chloroform-*d*) S8

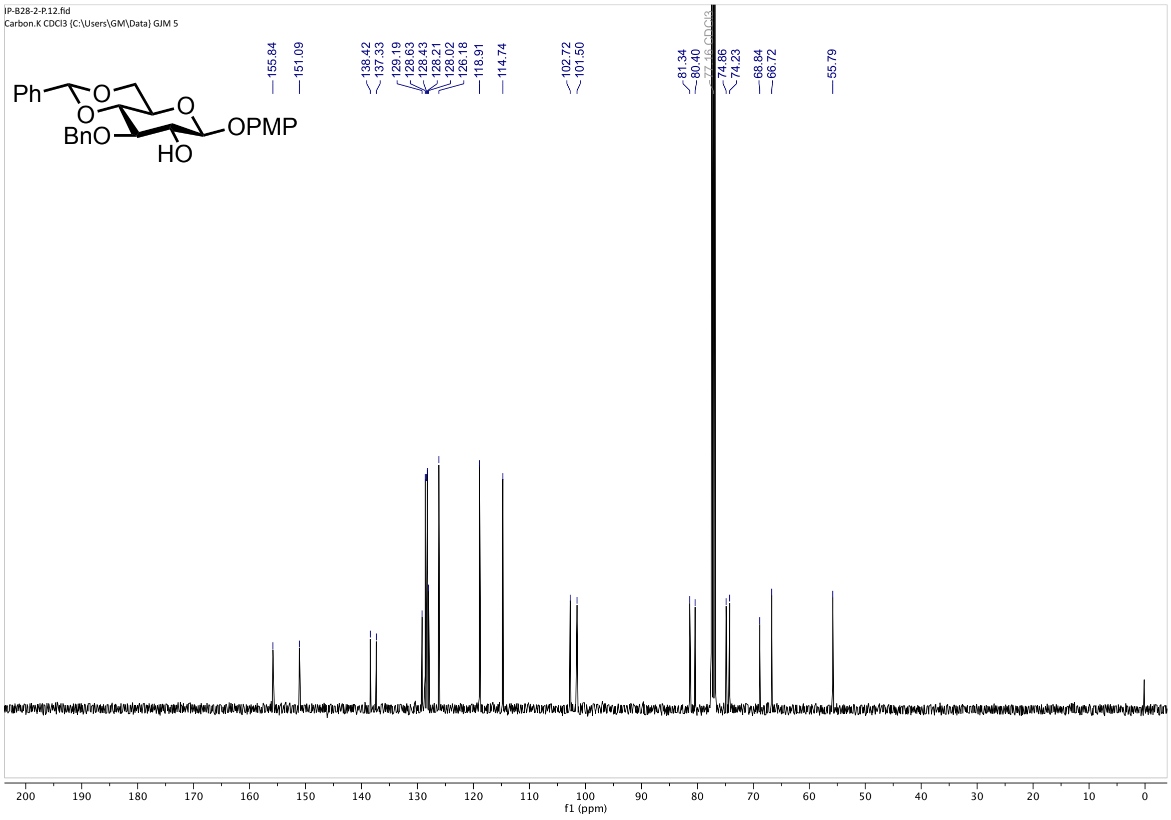

Compound S9

^1H NMR (400 MHz, Chloroform-*d*) S9

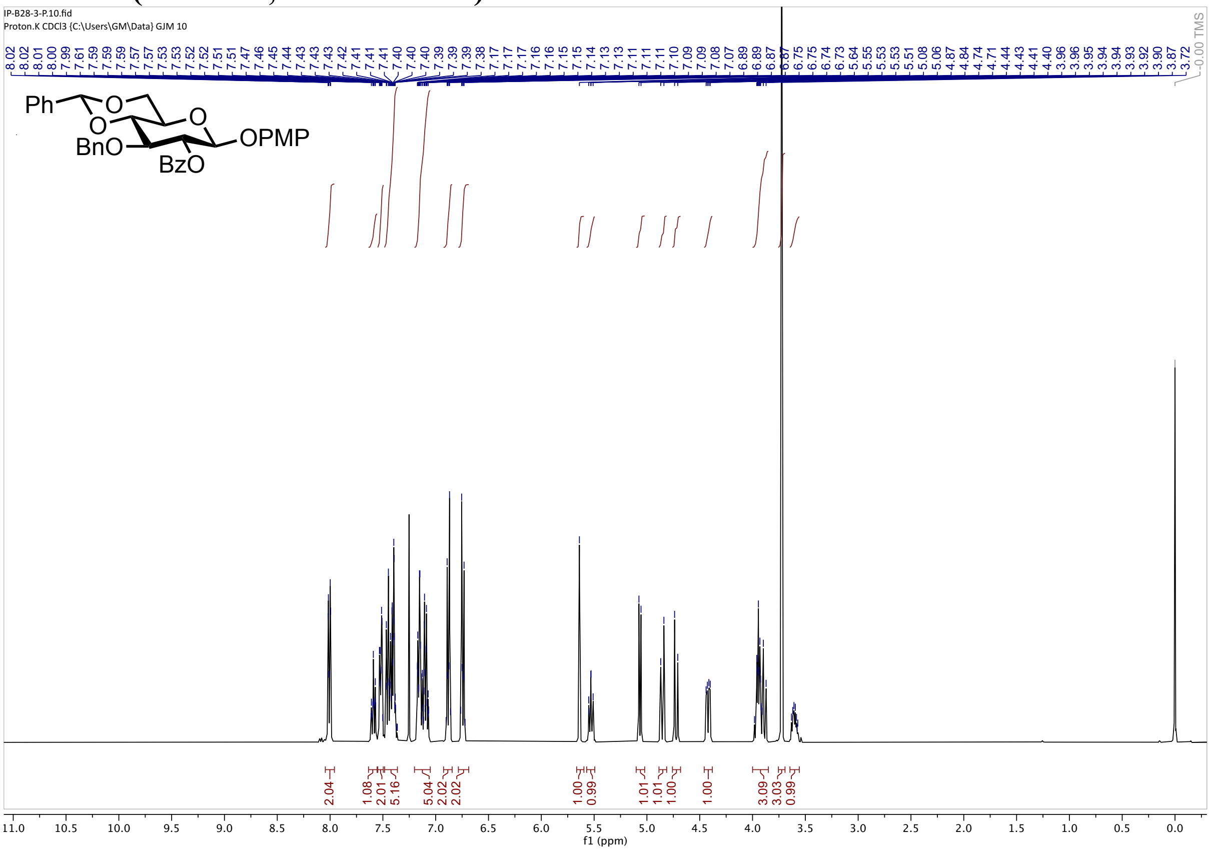

$^{13}C\{^1H\}$ NMR (101 MHz, Chloroform-*d*) S9

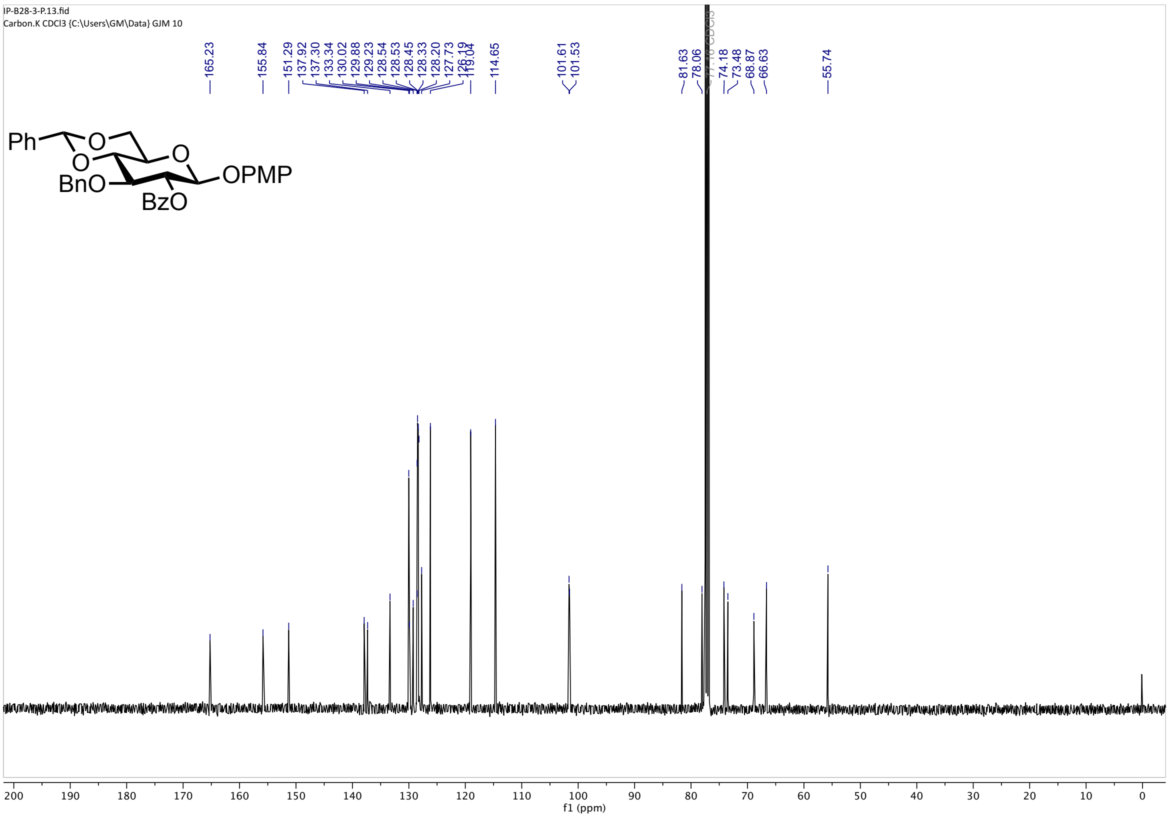

Compound S10

^1H NMR (400 MHz, Chloroform-*d*) S10

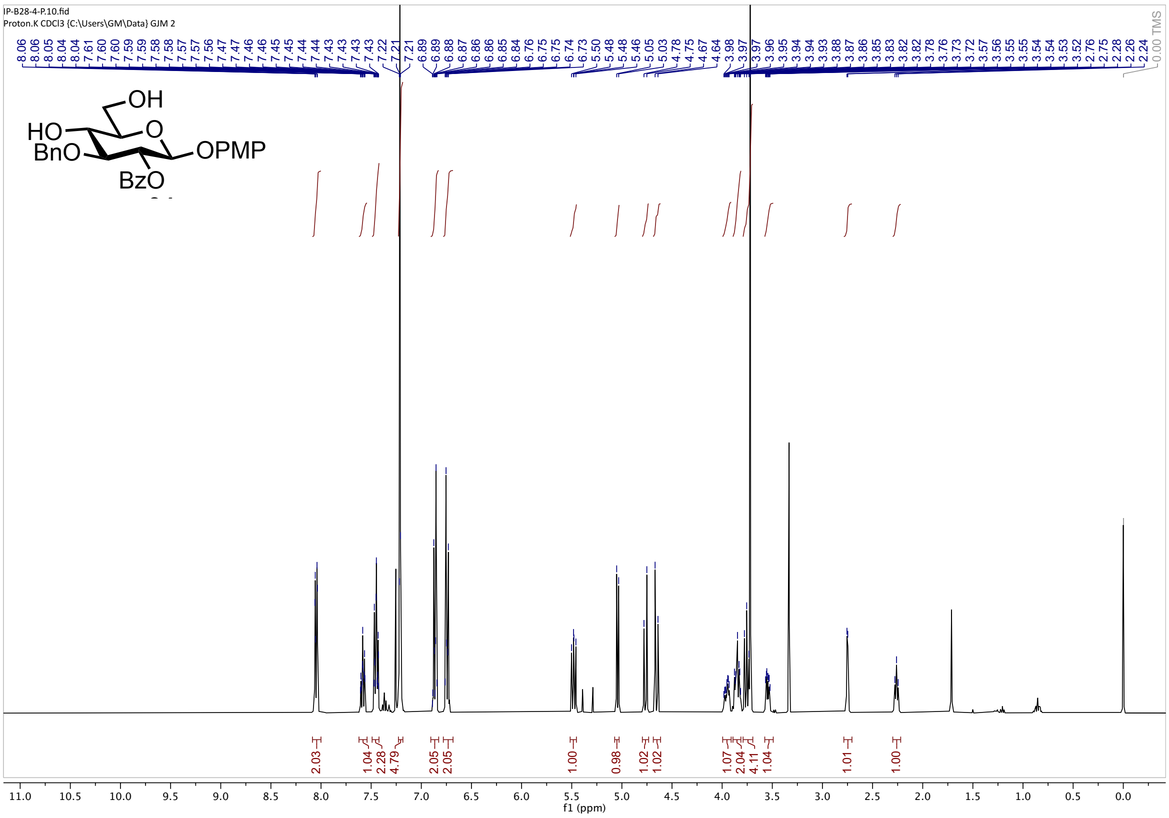

$^{13}C\{^{1}H\}$ NMR (101 MHz, Chloroform-*d*) S10

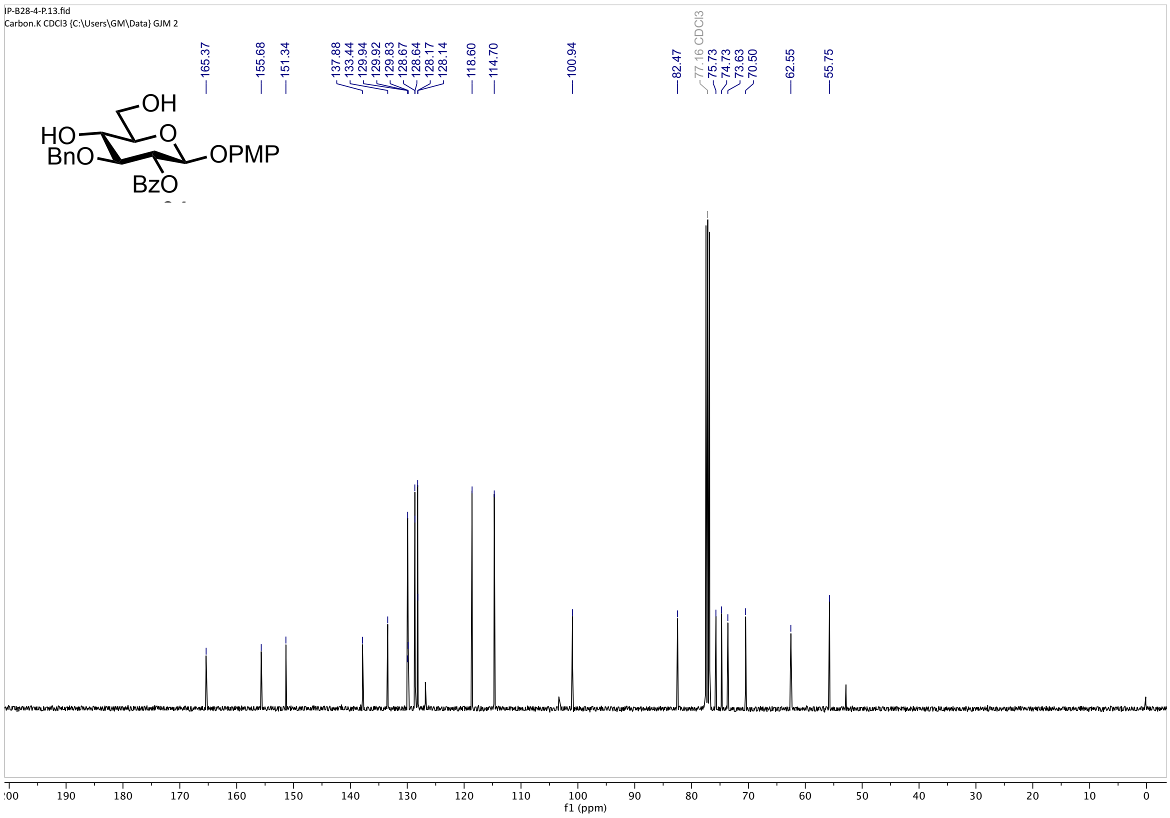

Compound 14

^{1}H NMR (400 MHz, Chloroform-*d*) 14

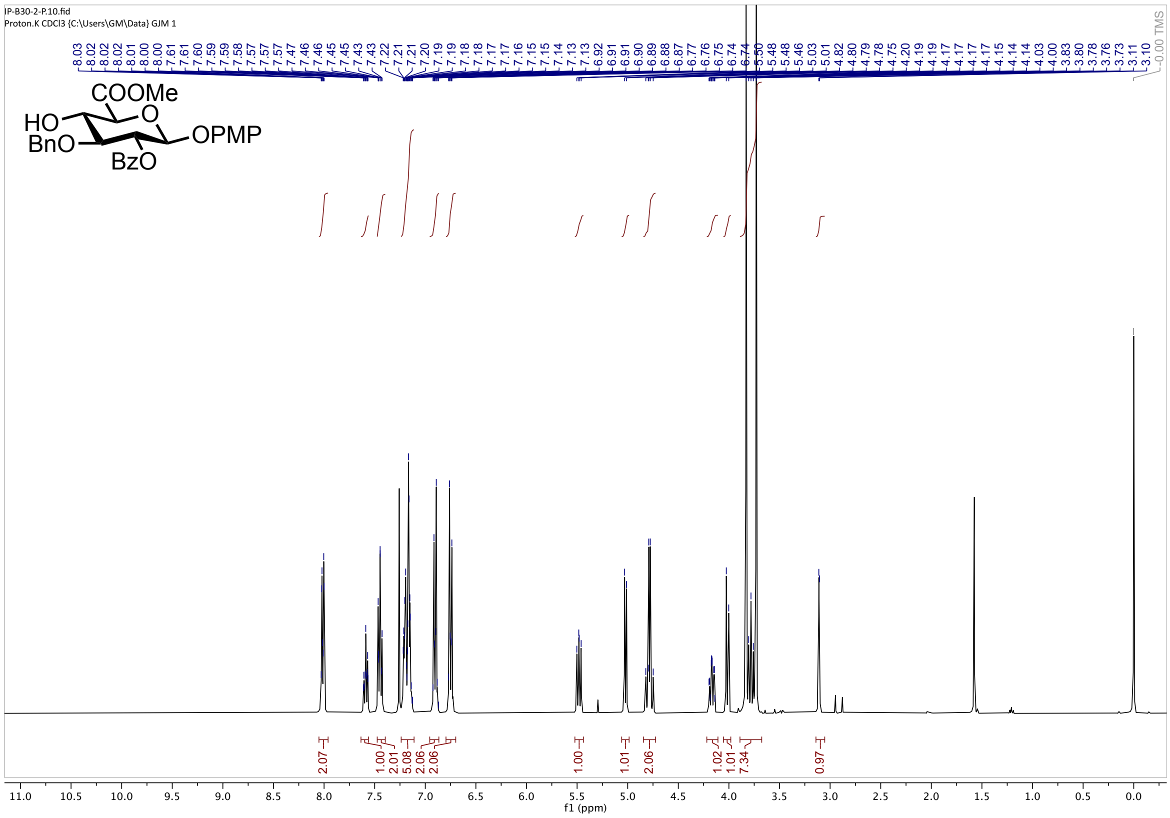

$^{13}C\{^1H\}$ NMR (101 MHz, Chloroform-*d*) 14

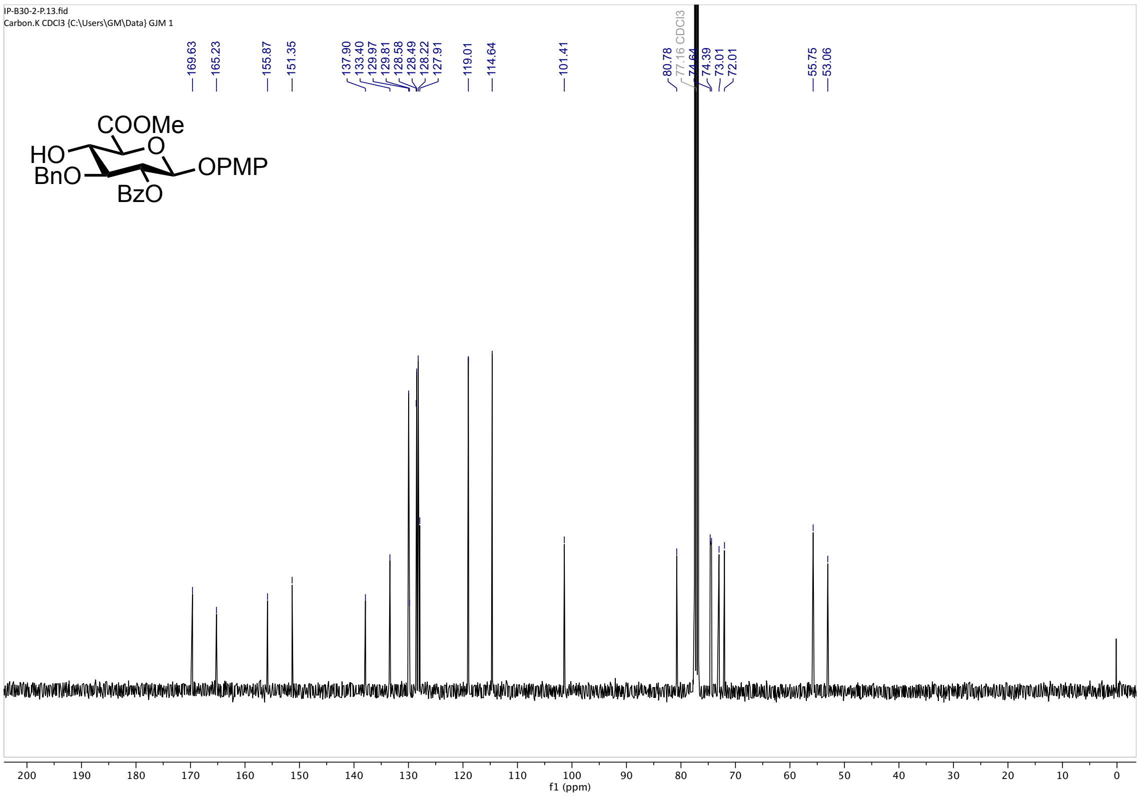

Synthesis of Glucuronic Glycal Acceptor

Compound S26

^1H NMR (400 MHz, Chloroform-*d*) 26

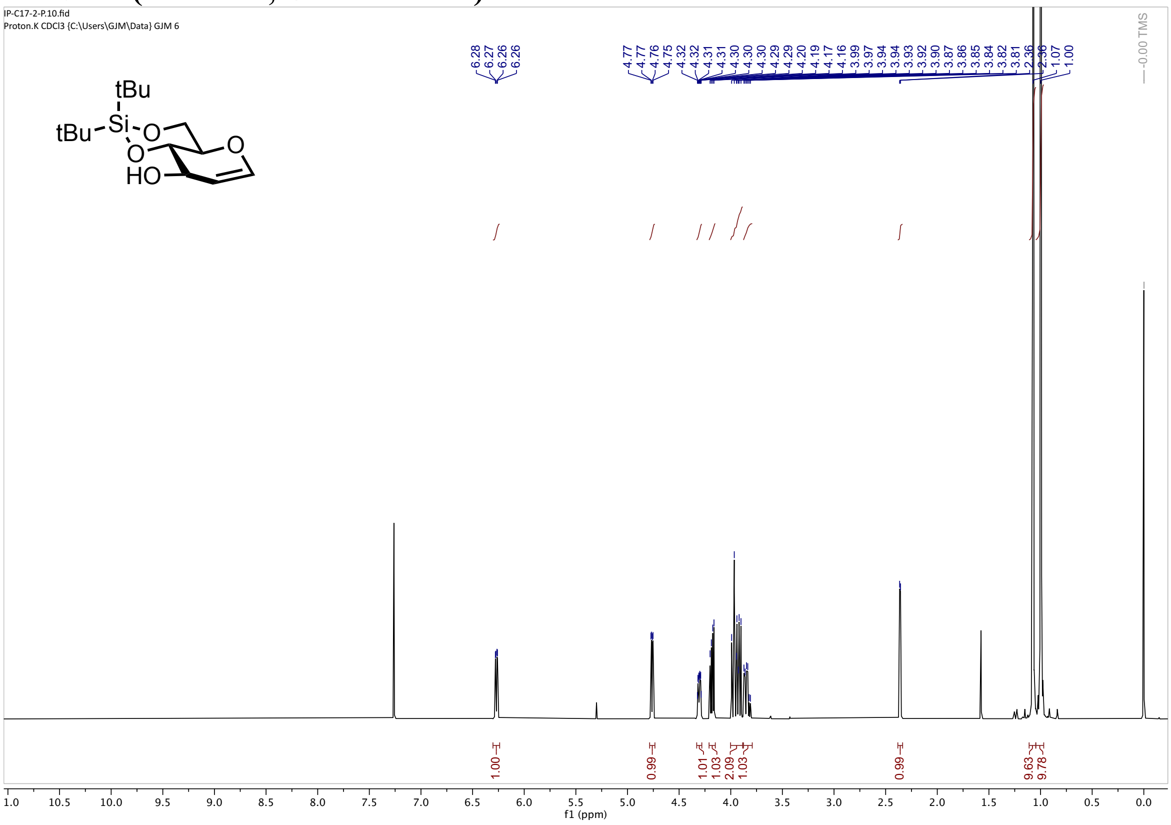

$^{13}C\{^{1}H\}$ NMR (101 MHz, Chloroform-*d*) 26

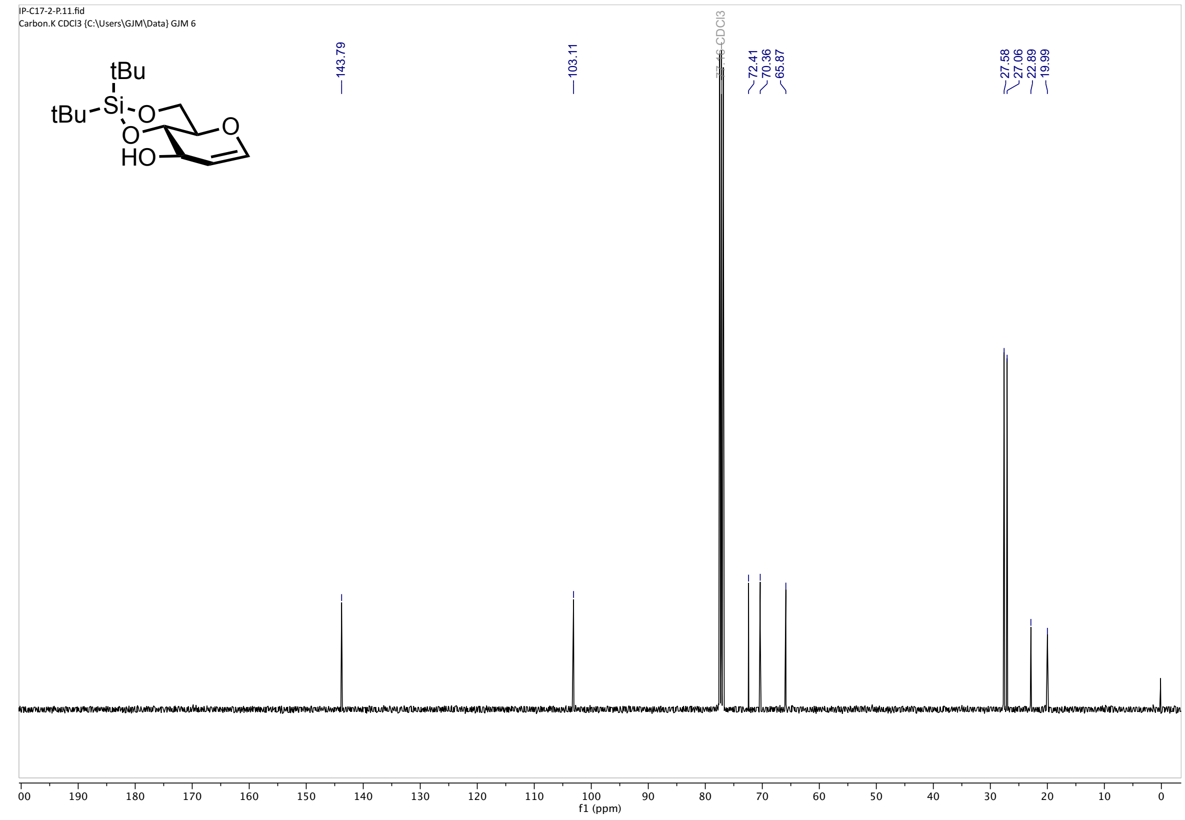

Compound 28

^{1}H NMR (400 MHz, Chloroform-*d*) 28

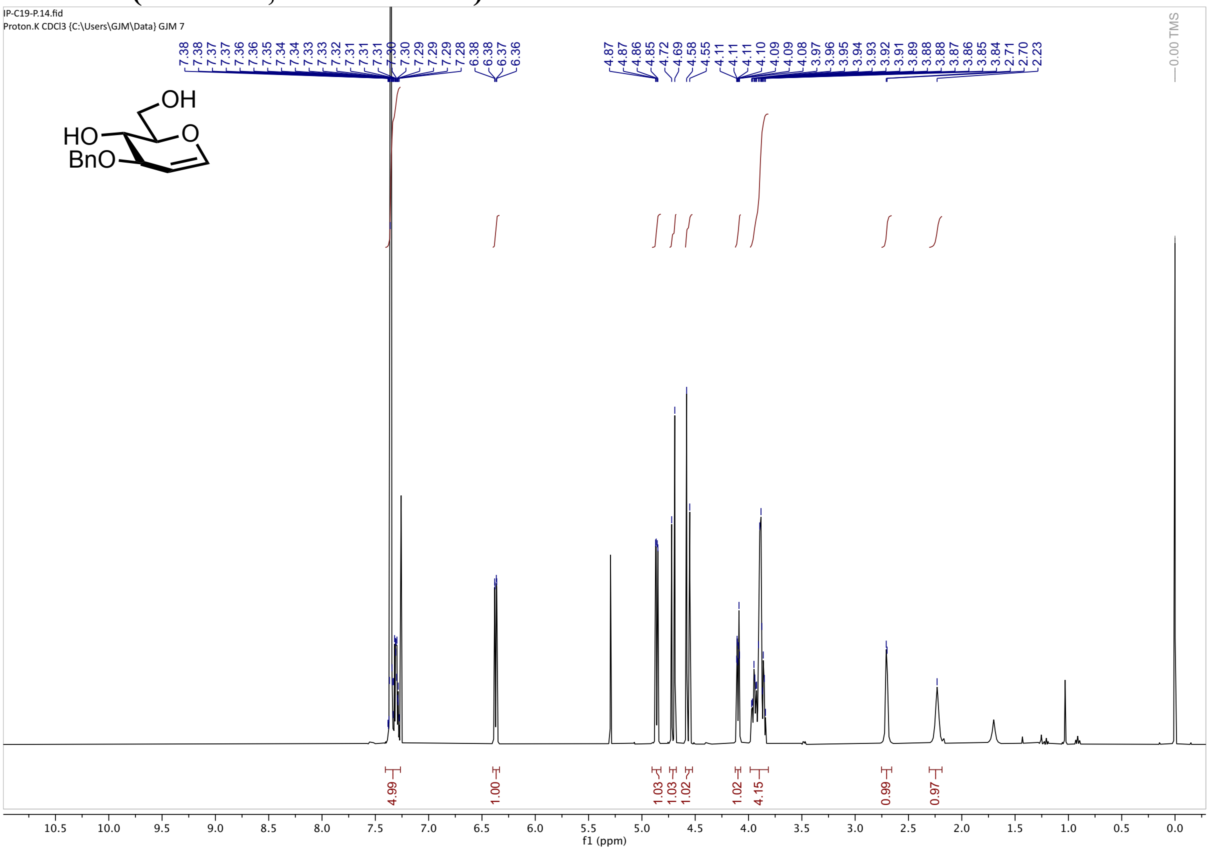

$^{13}C\{^1H\}$ NMR (101 MHz, Chloroform-*d*) 28

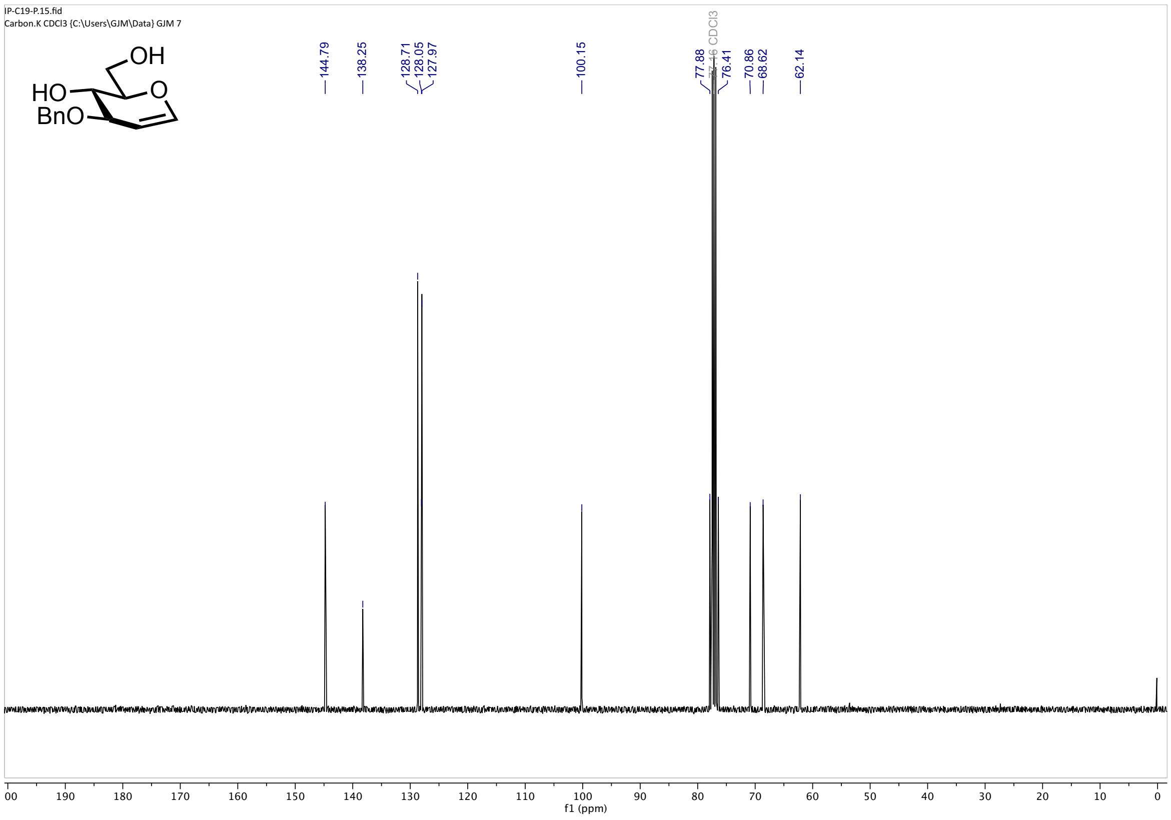

Compound 29

^1H NMR (400 MHz, Chloroform-*d*) 29

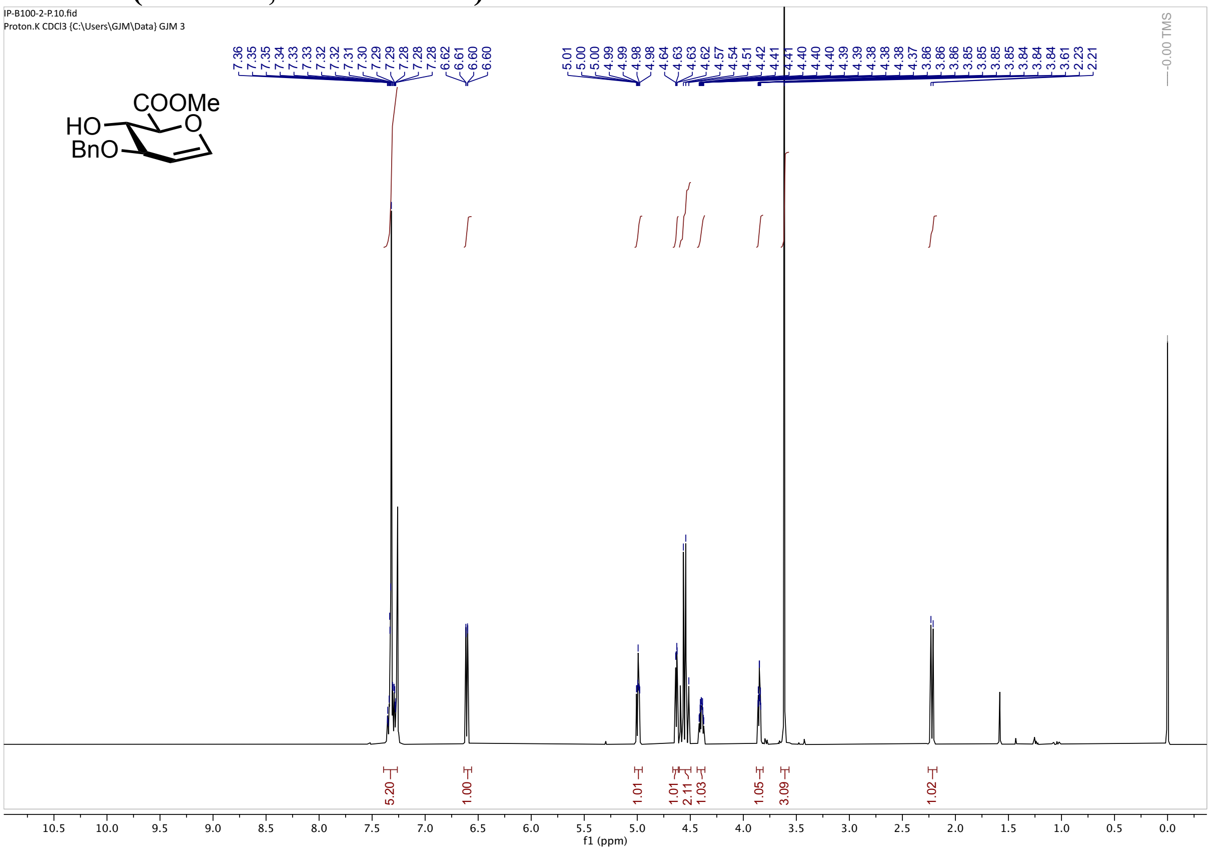

$^{13}C\{^{1}H\}$ NMR (101 MHz, Chloroform-*d*) 29

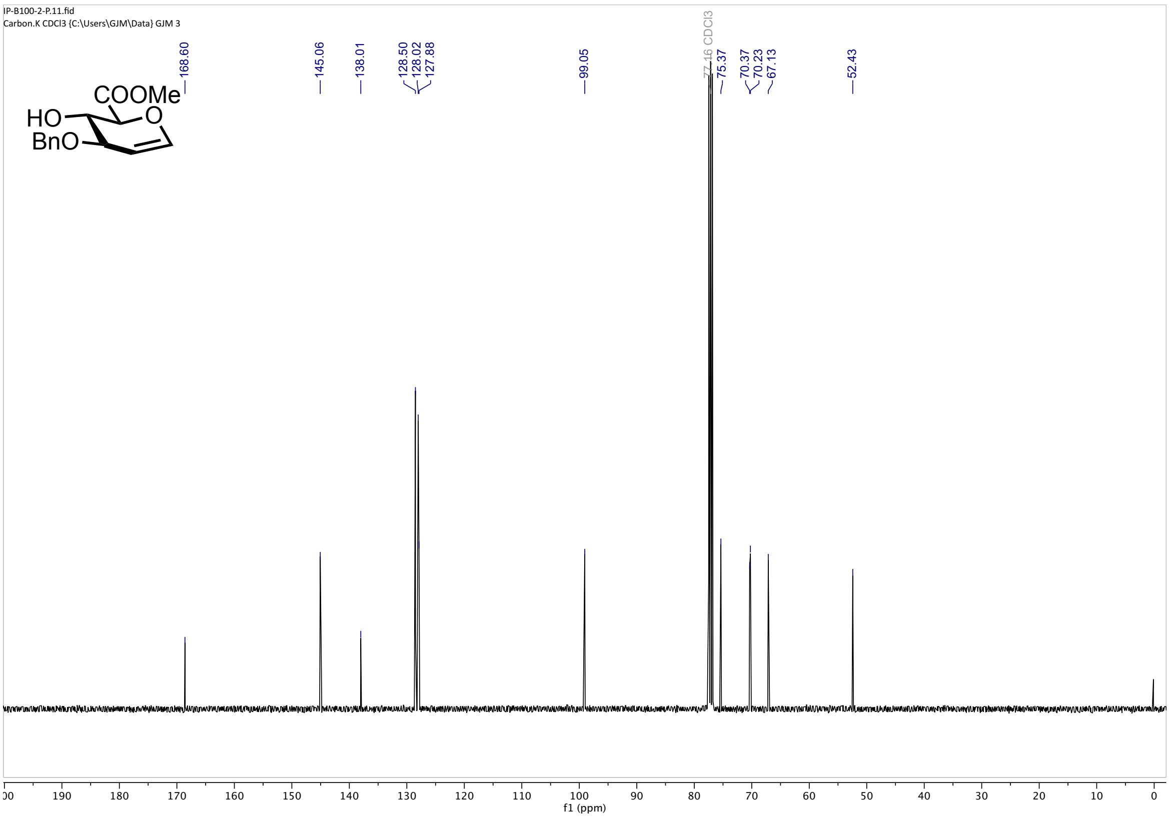

COSY NMR (400 MHz, Chloroform-*d*) 29

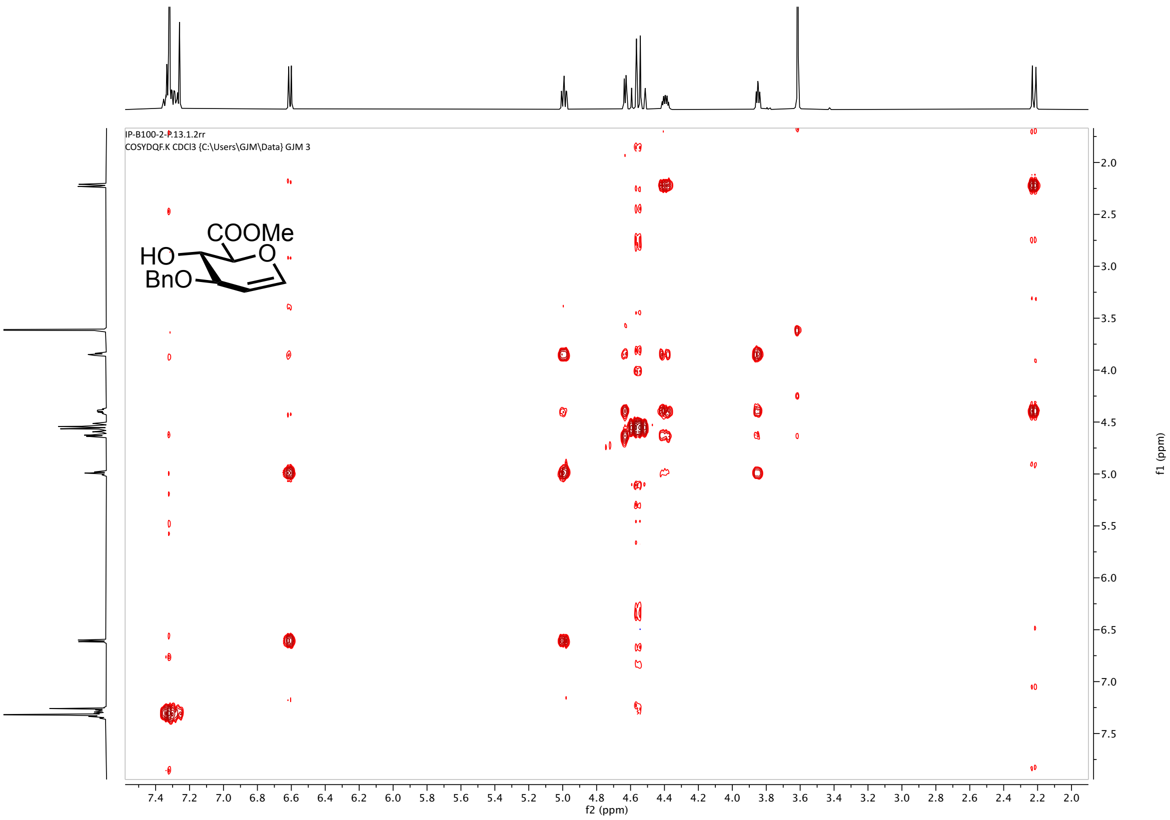

HSQC NMR (400 MHz x 101 MHz, Chloroform-*d*) 29

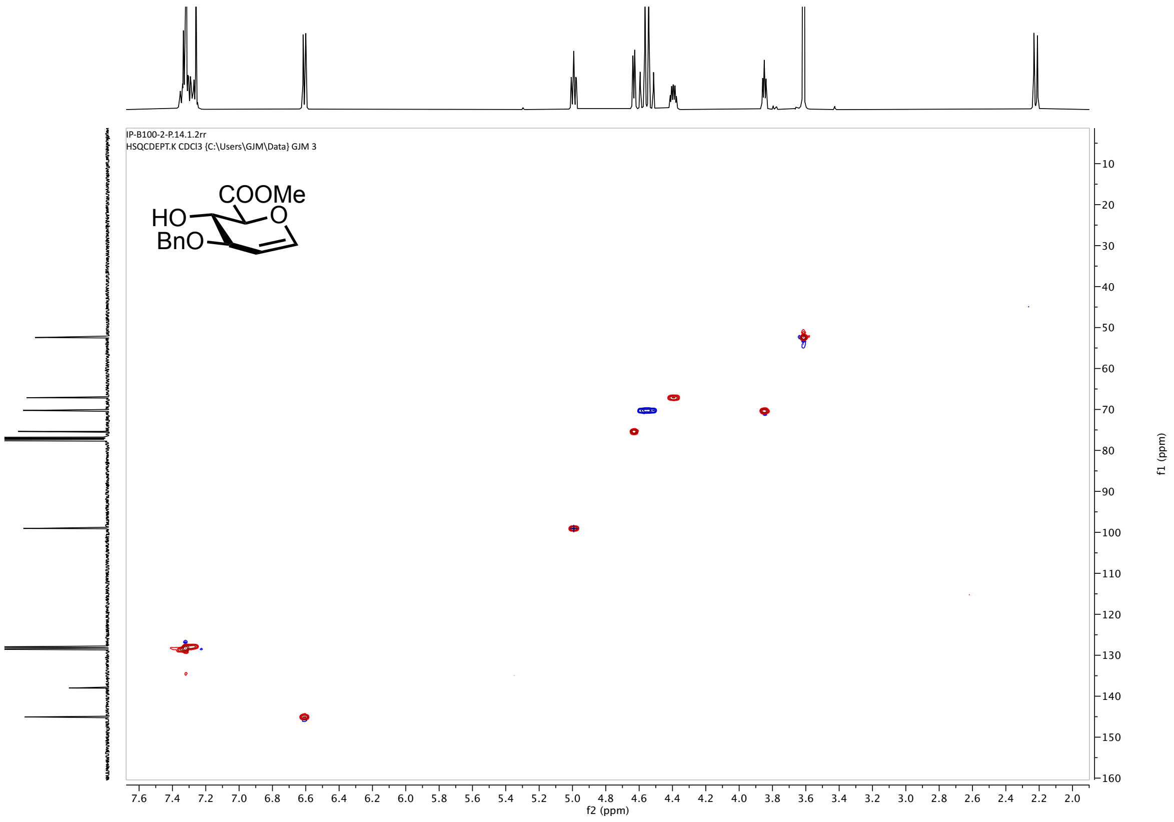

HMBC NMR (400 MHz x 101 MHz, Chloroform-*d*) 29

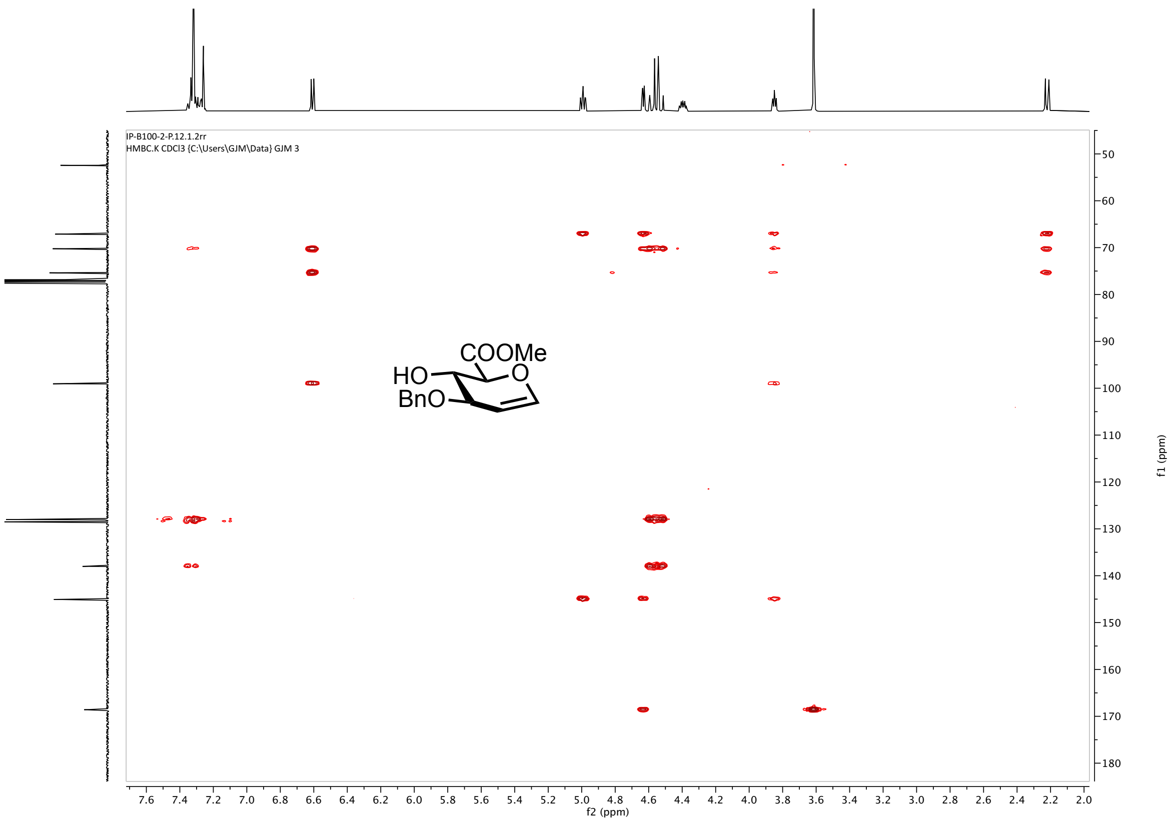

Synthesis of Glucosamine Donors

Compound S12

^{1}H NMR (400 MHz, Chloroform-*d*) S12

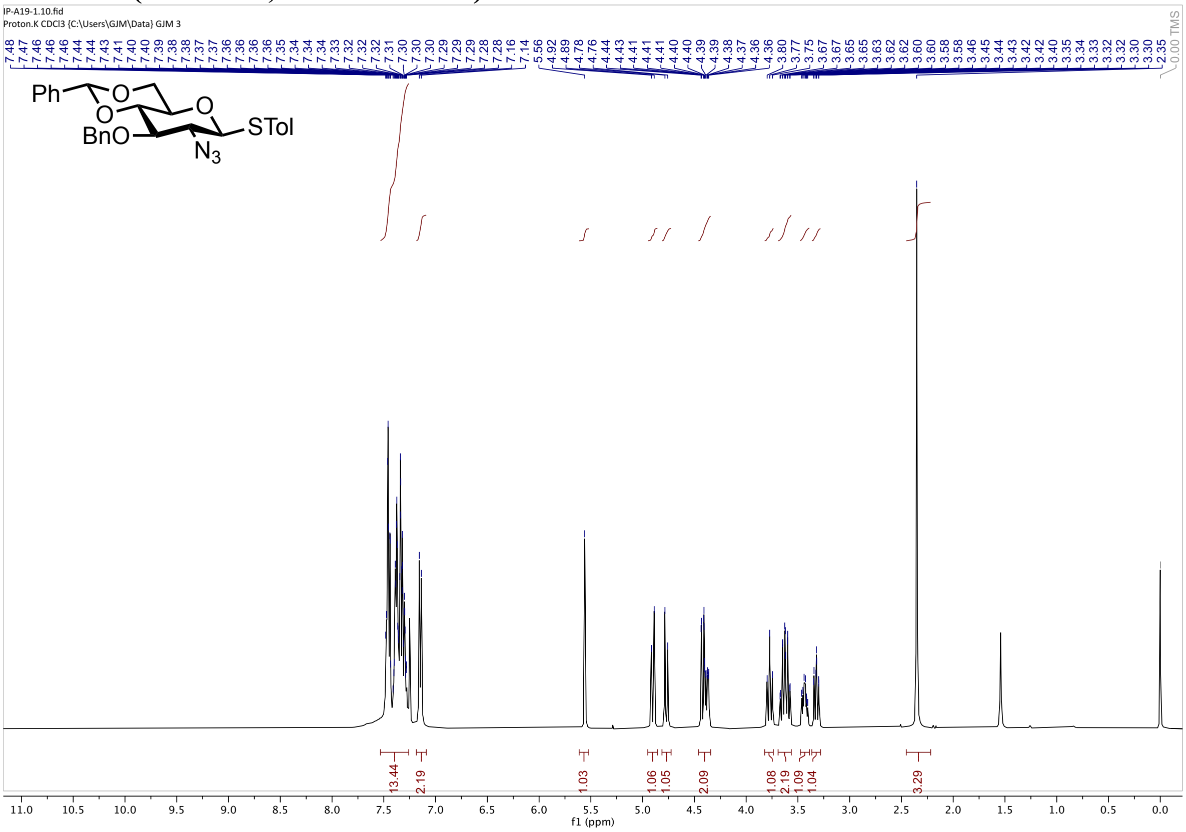

^{13}C{^{1}H} NMR (101 MHz, Chloroform-*d*) S12

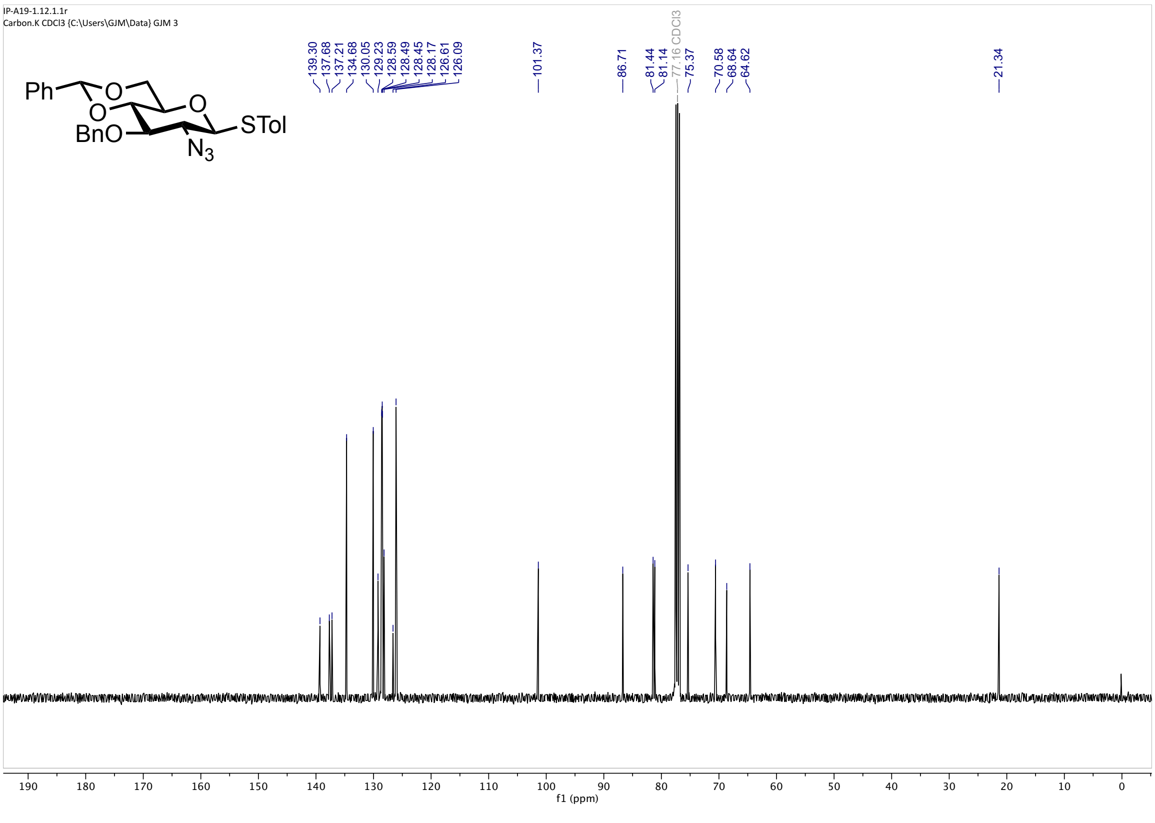

Compound S13

^{1}H NMR (400 MHz, Chloroform-*d*) S13

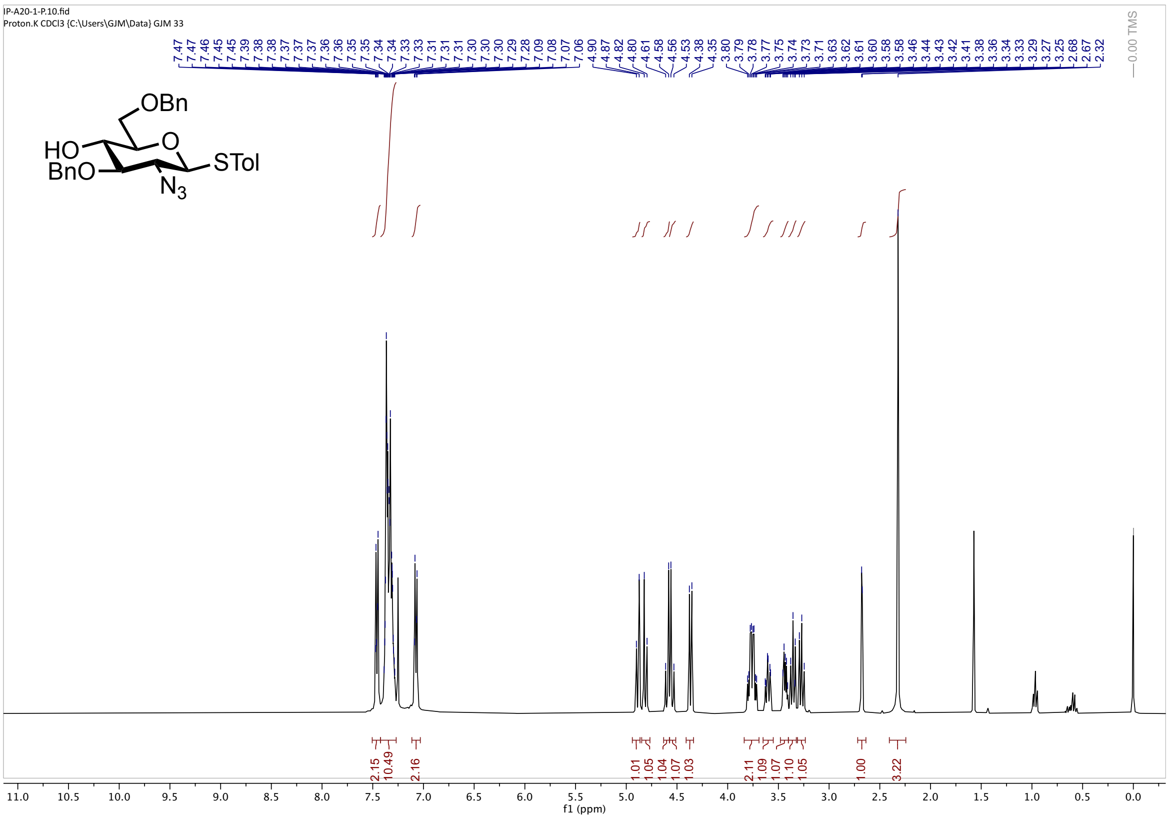

^{13}C{^{1}H} NMR (101 MHz, Chloroform-*d*) S13

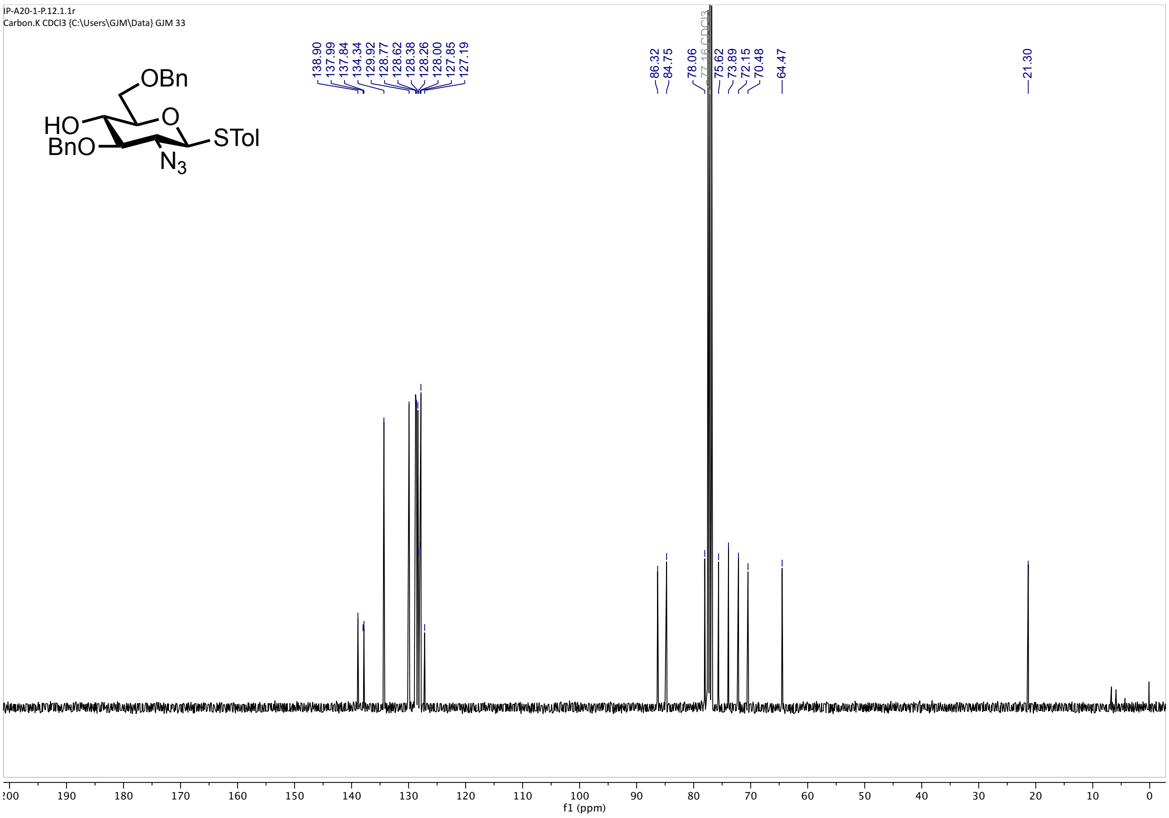

Compound 1a

^{1}H NMR (400 MHz, Chloroform-*d*) 1a

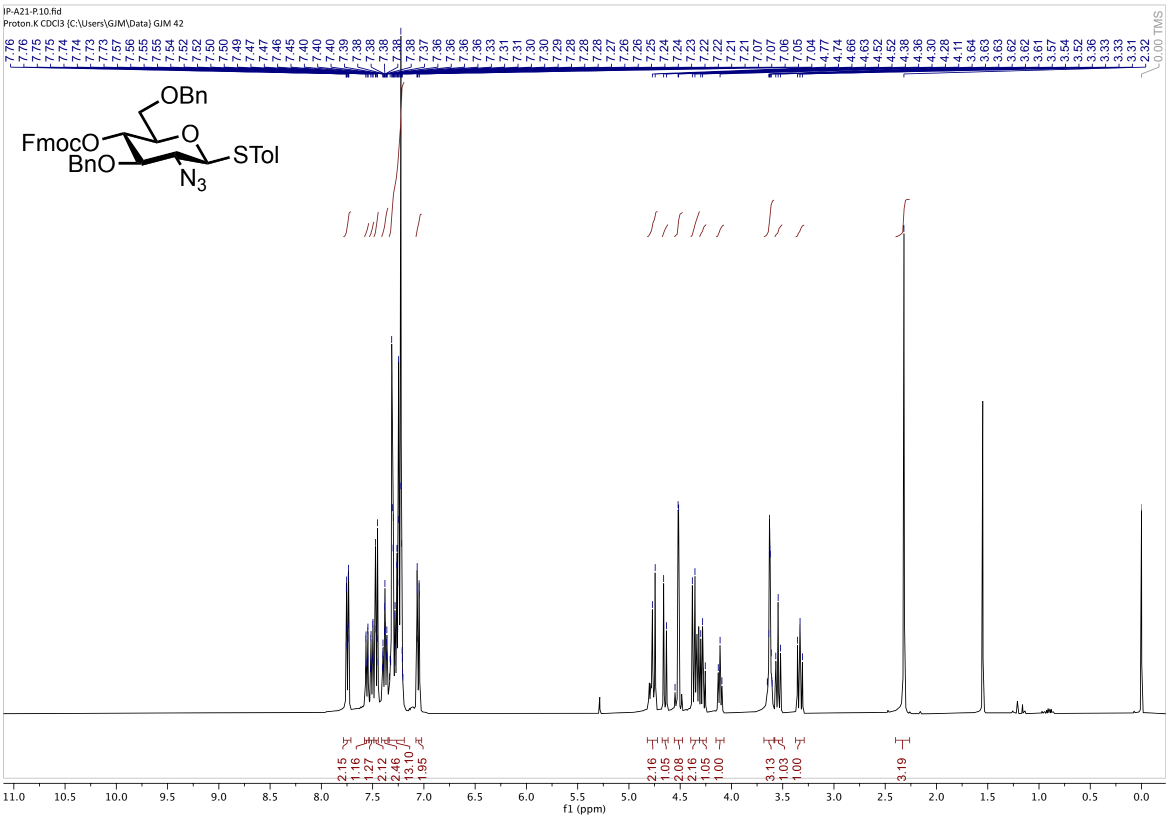

^{13}C{^{1}H} NMR (101 MHz, Chloroform-*d*) 1a

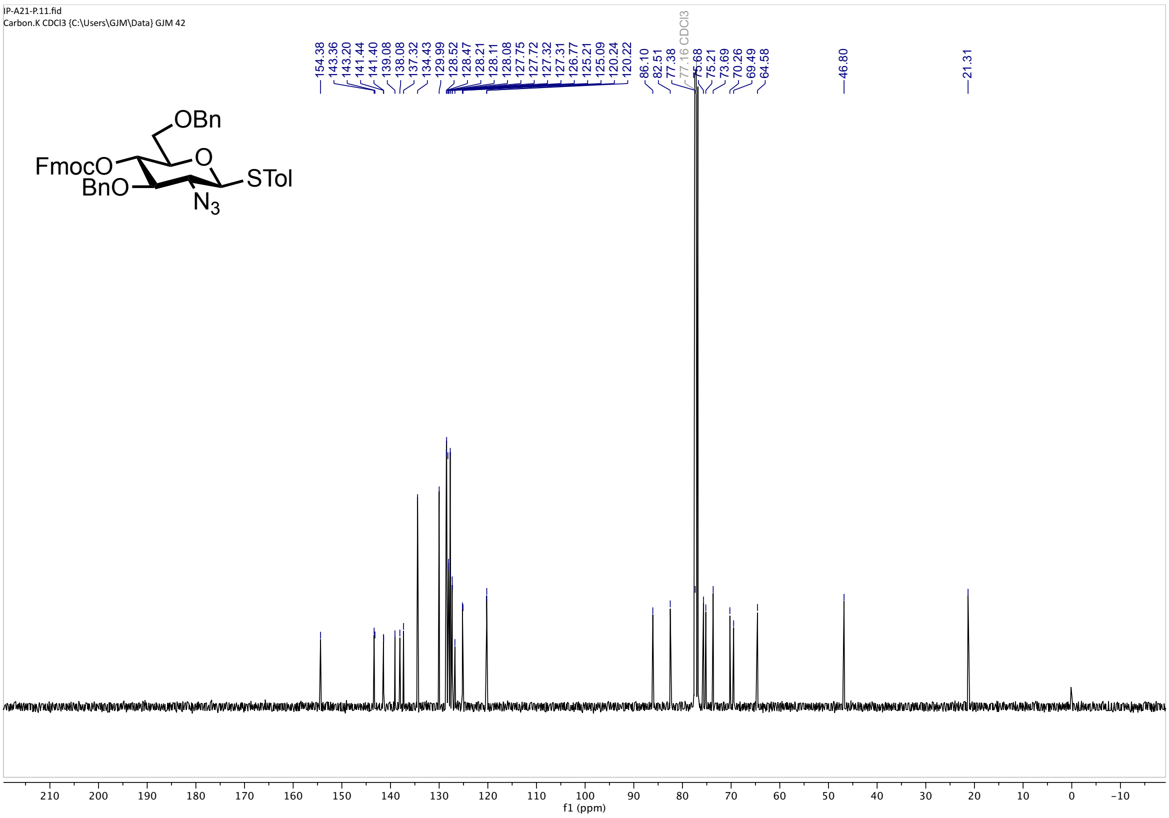

COSY NMR (400 MHz, Chloroform-*d*) 1a

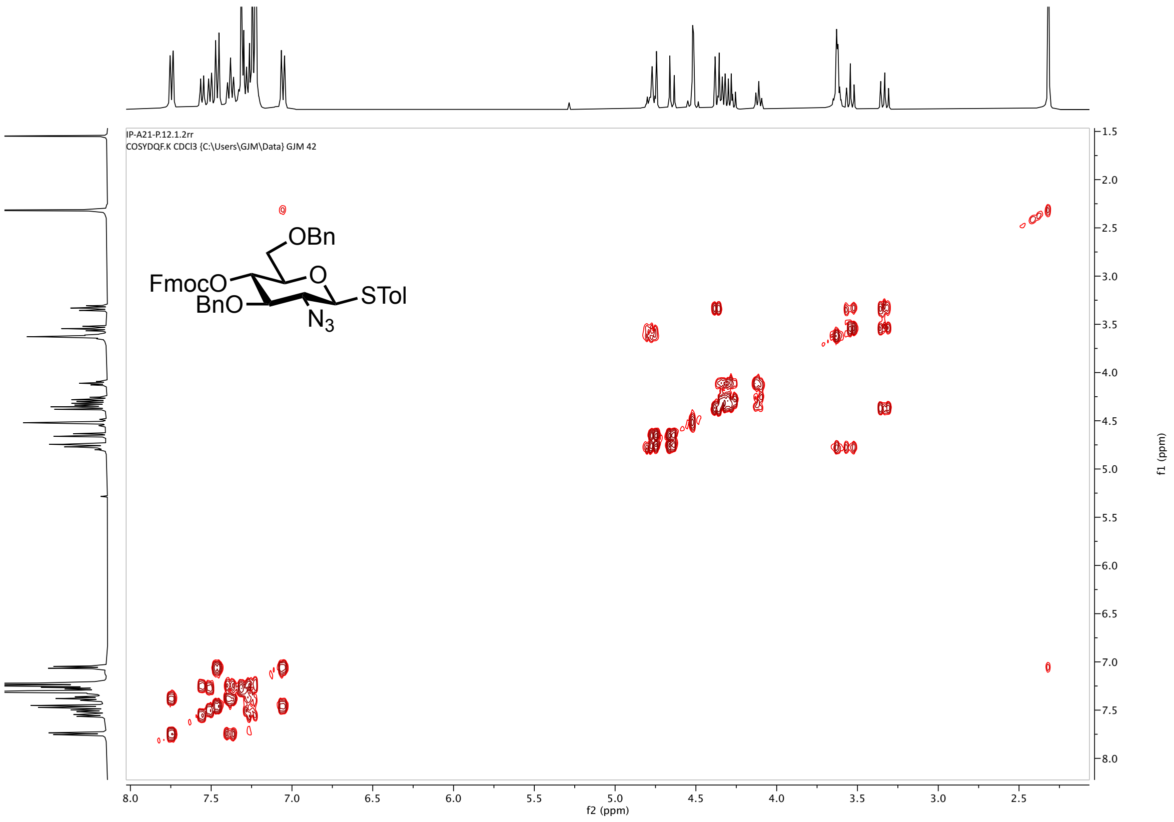

HSQC NMR (400 MHz x 101 MHz, Chloroform-*d*) 1a

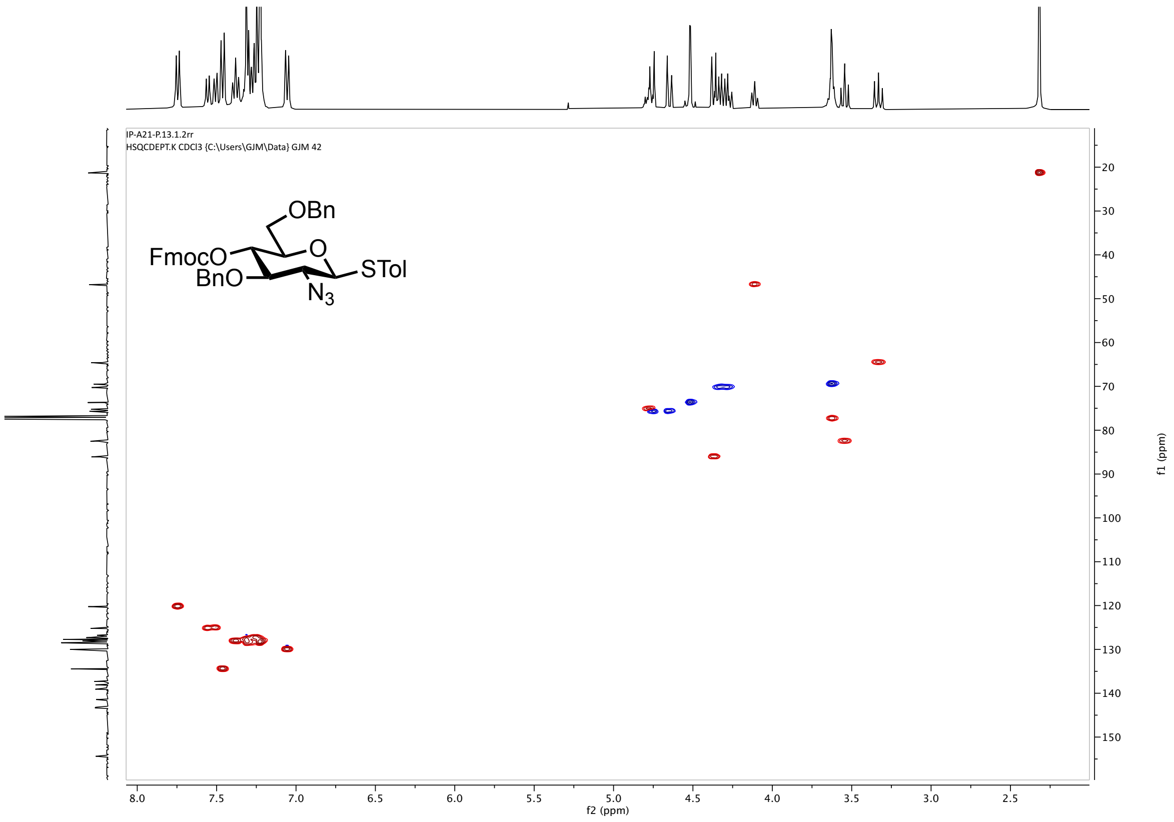

HMBC NMR (400 MHz x 101 MHz, Chloroform-*d*) 1a

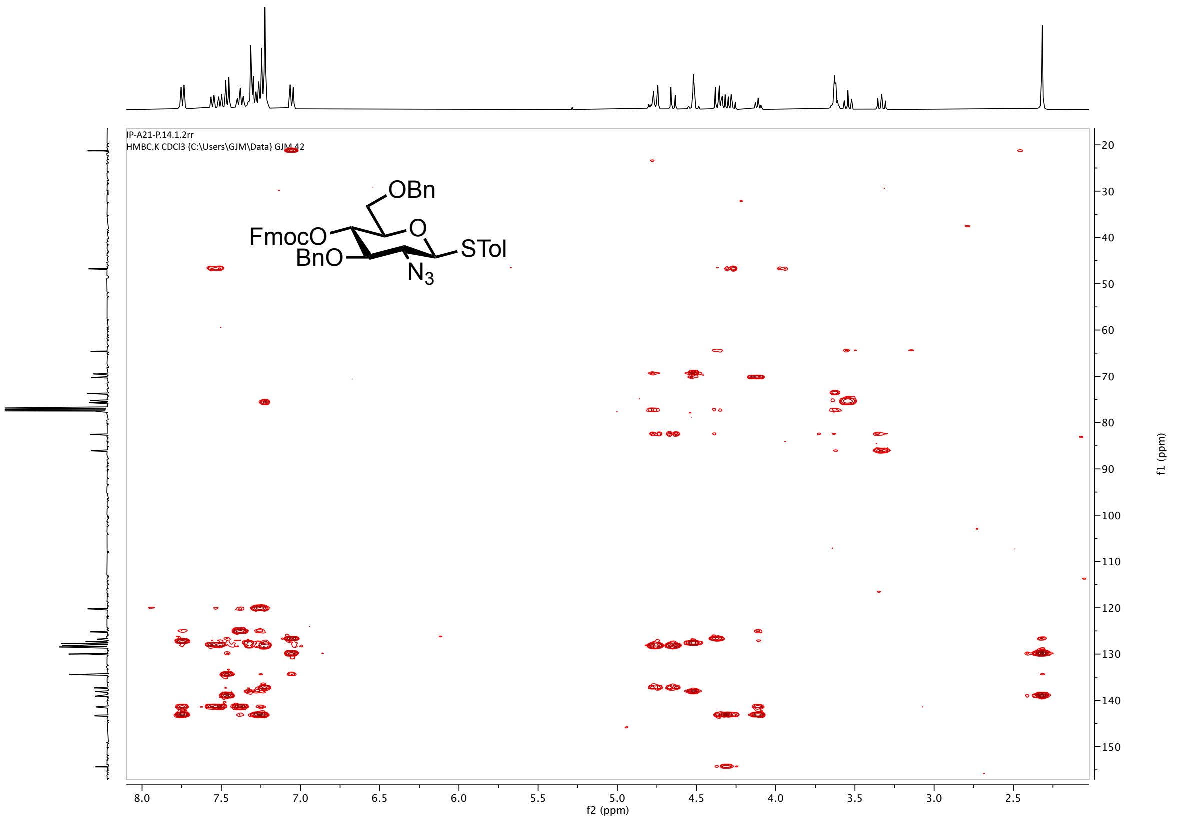

Compound 2a

^{1}H NMR (400 MHz, Chloroform-*d*) 2a

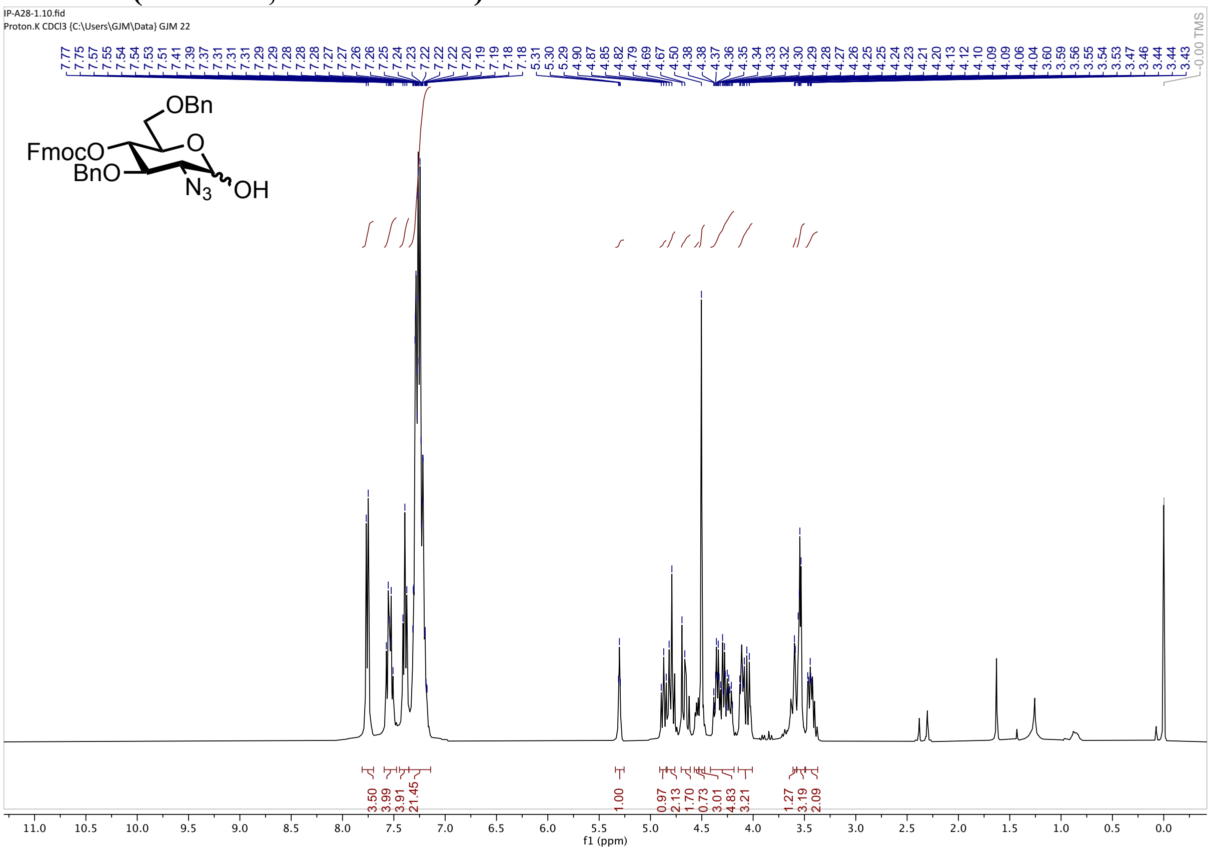

$^{13}C\{^1H\}$ NMR (101 MHz, Chloroform-*d*) 2a

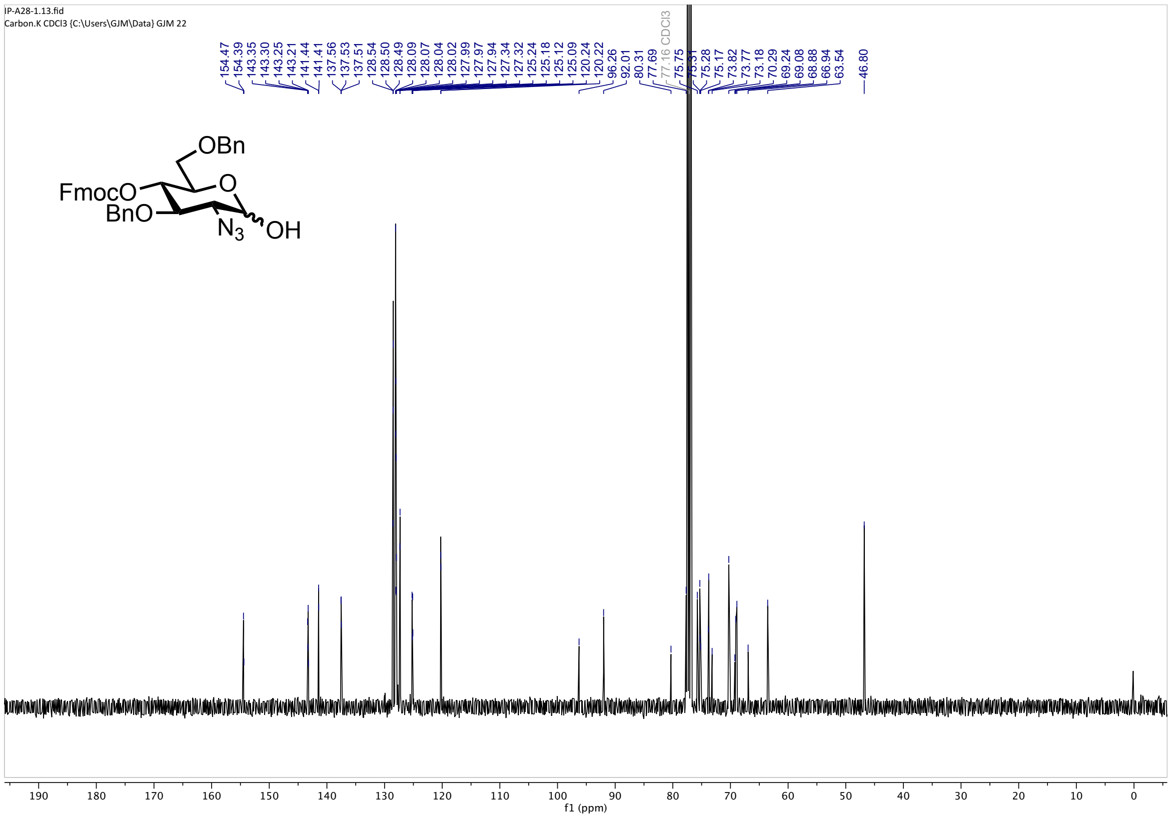

COSY NMR (400 MHz, Chloroform-*d*) 2a

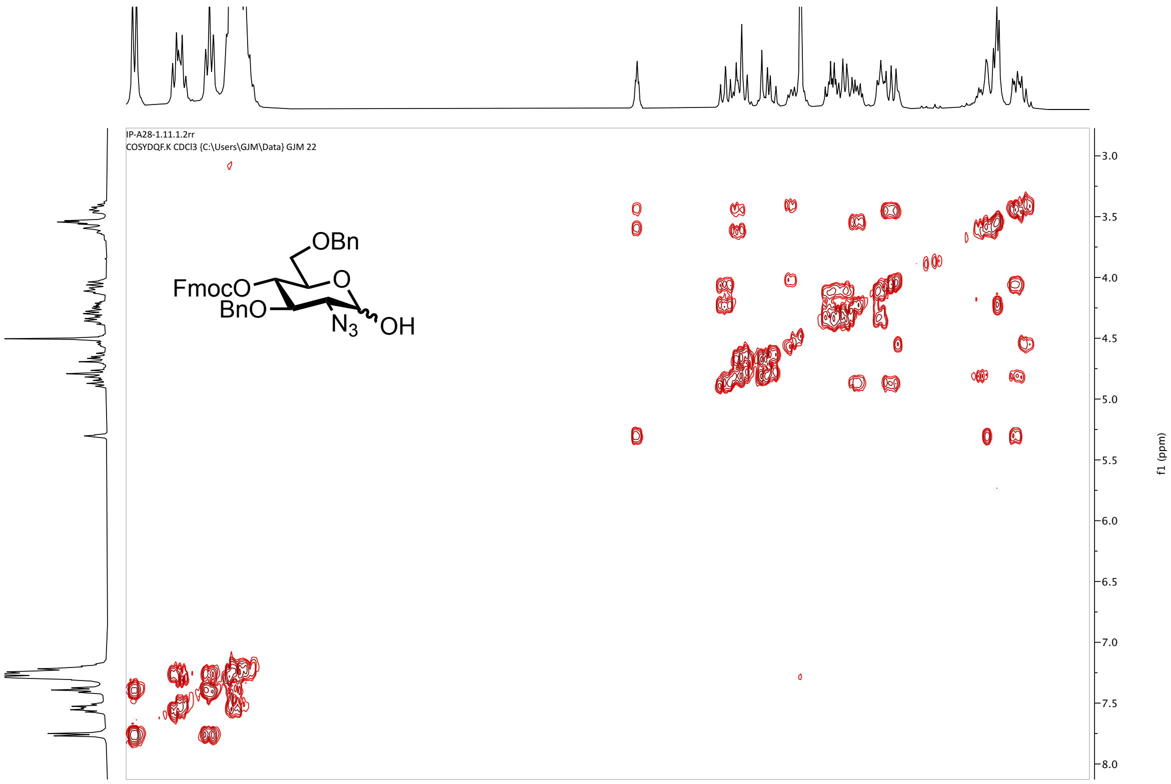

HSQC NMR (400 MHz x 101 MHz, Chloroform-*d*) 2a

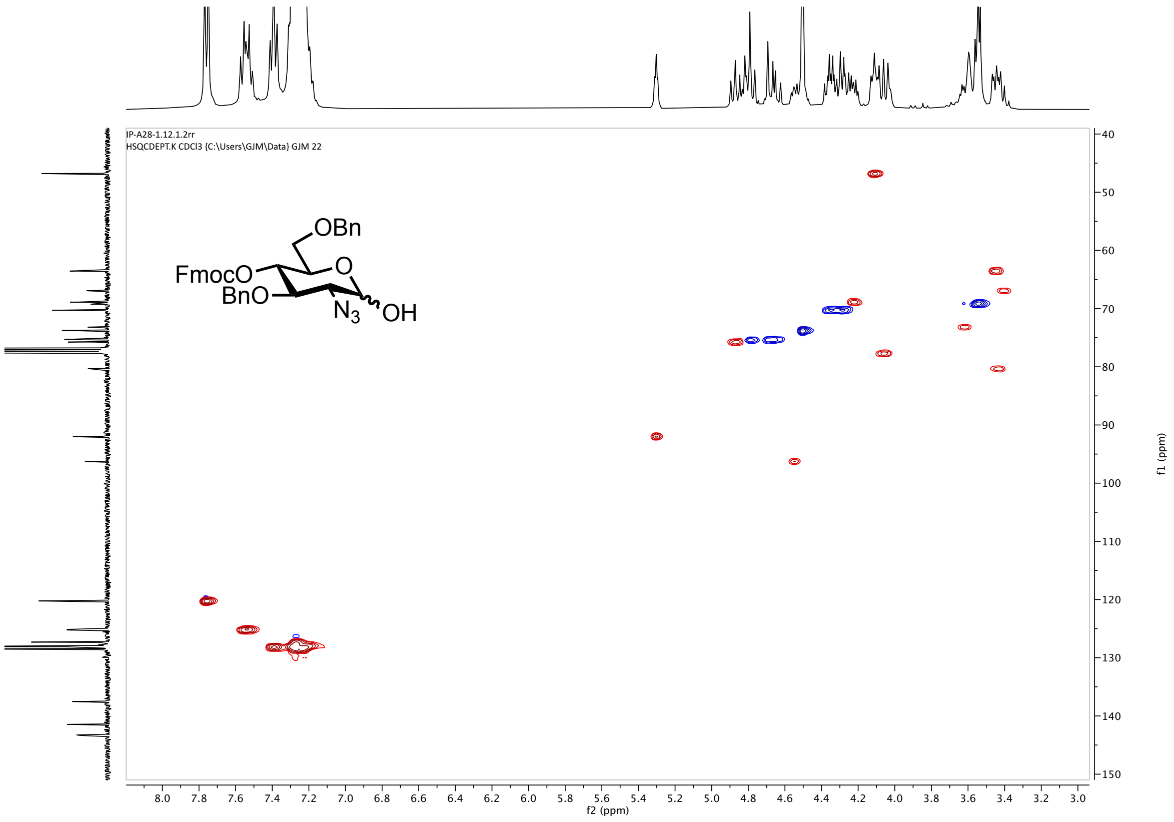

HMBC NMR (400 MHz x 101 MHz, Chloroform-*d*) 2a

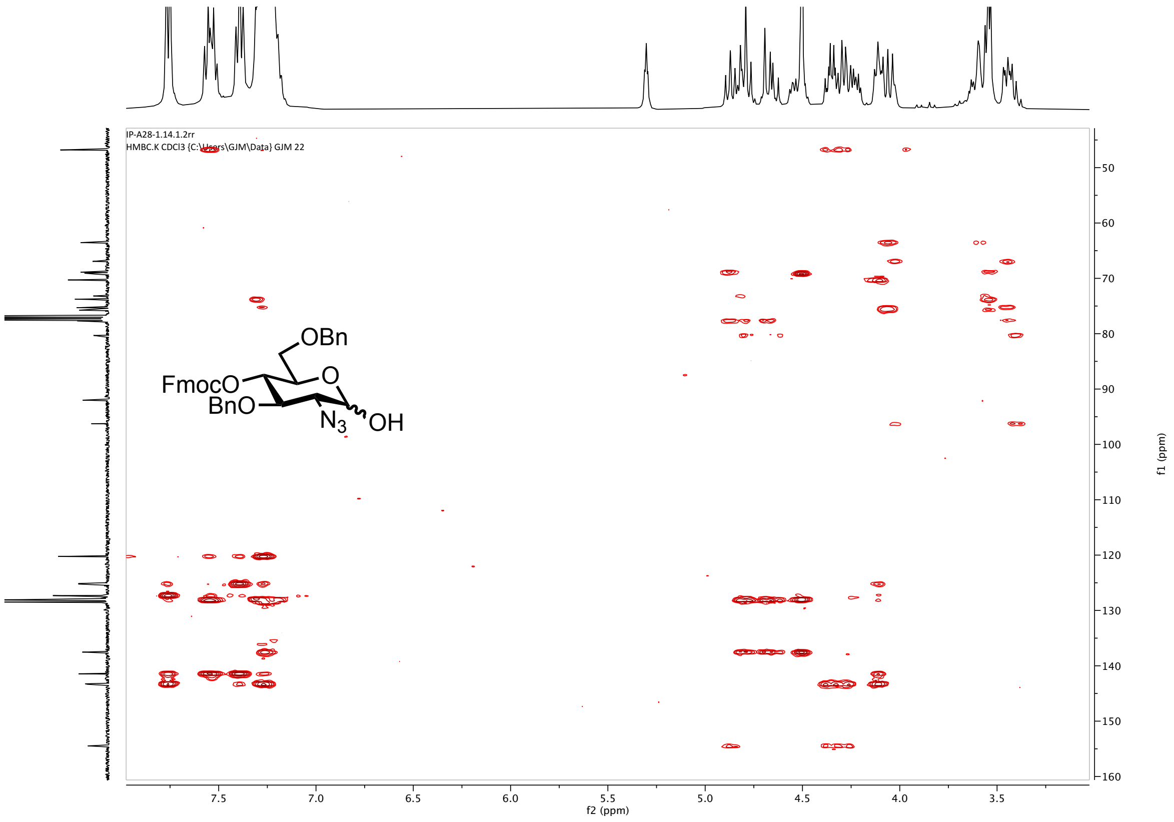

Compound 1b

^{1}H NMR (400 MHz, Chloroform-*d*) 1b crude

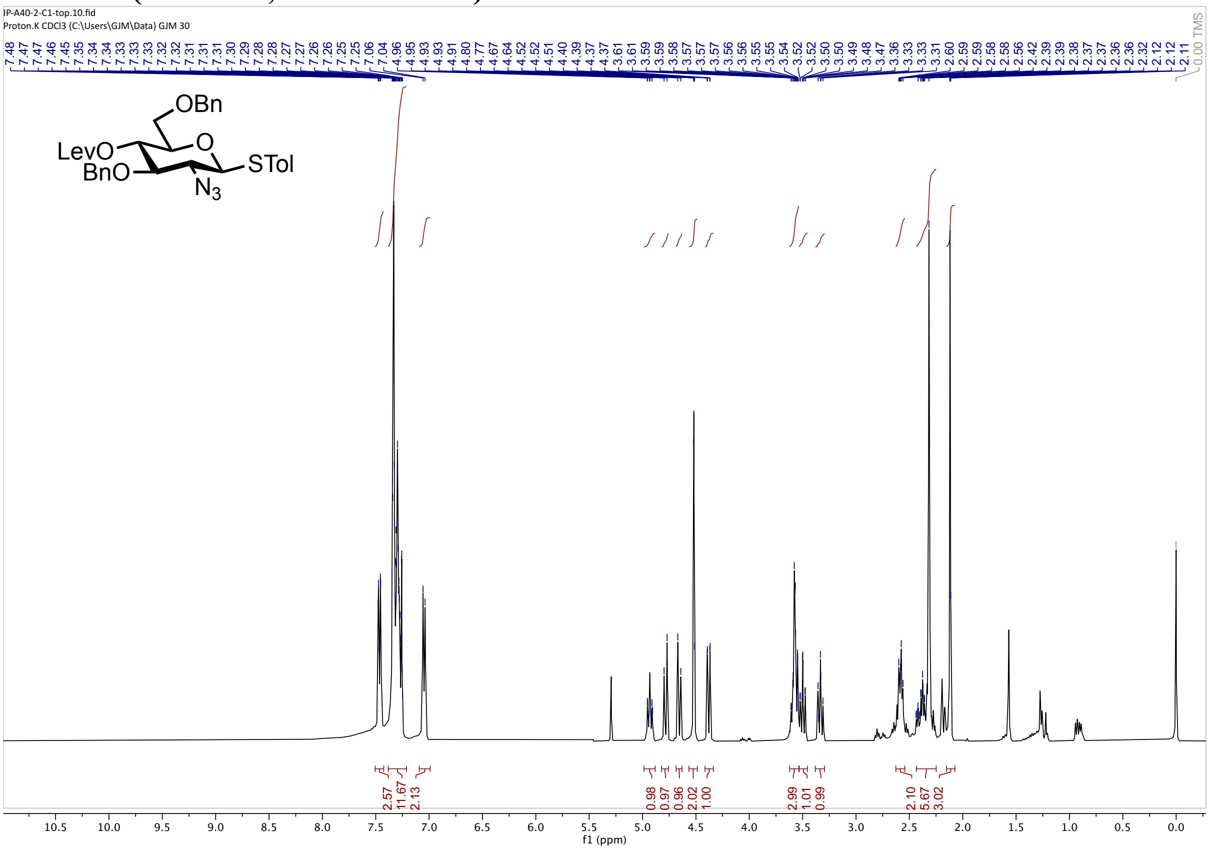

Compound 2b

^{1}H NMR (400 MHz, Chloroform-*d*) 2b

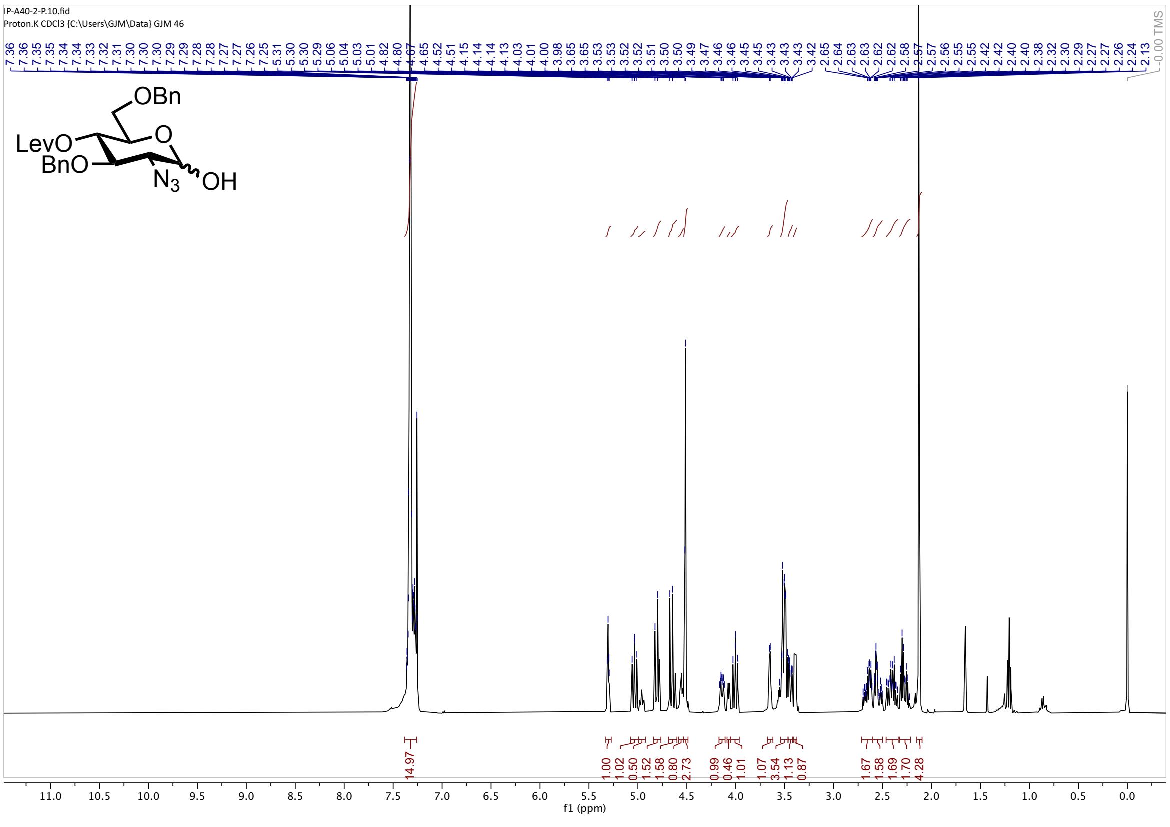

$^{13}C\{^1H\}$ NMR (101 MHz, Chloroform-*d*) 2b

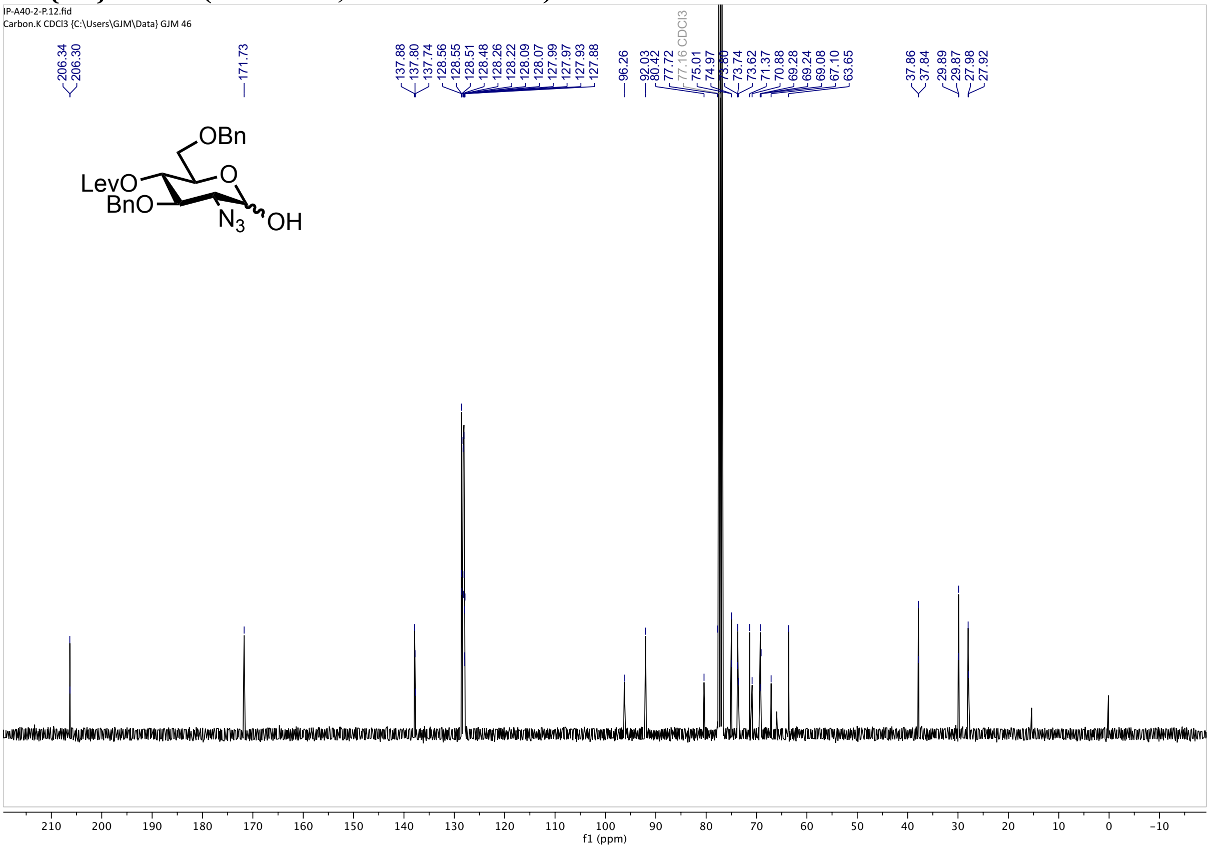

COSY NMR (400 MHz, Chloroform-*d*) 2b

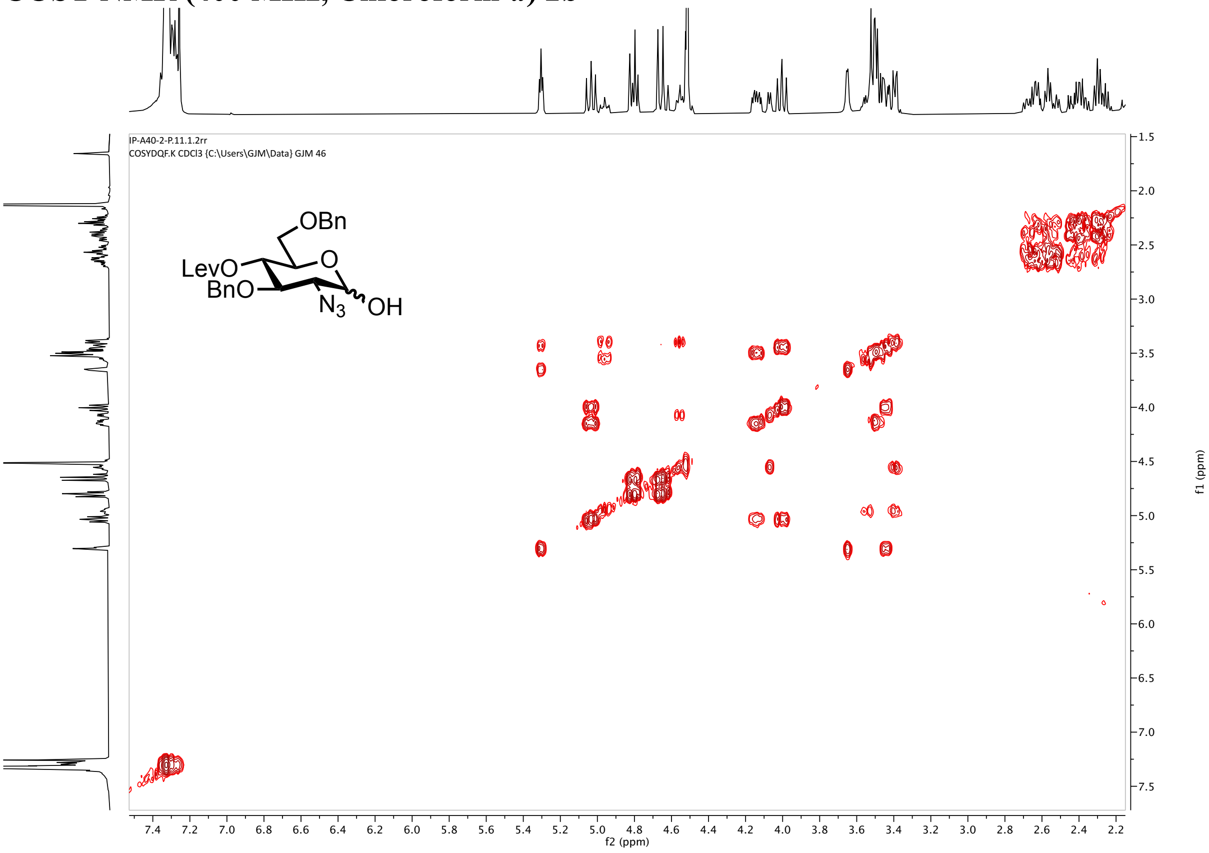

HSQC NMR (400 MHz x 101 MHz, Chloroform-*d*) 2b

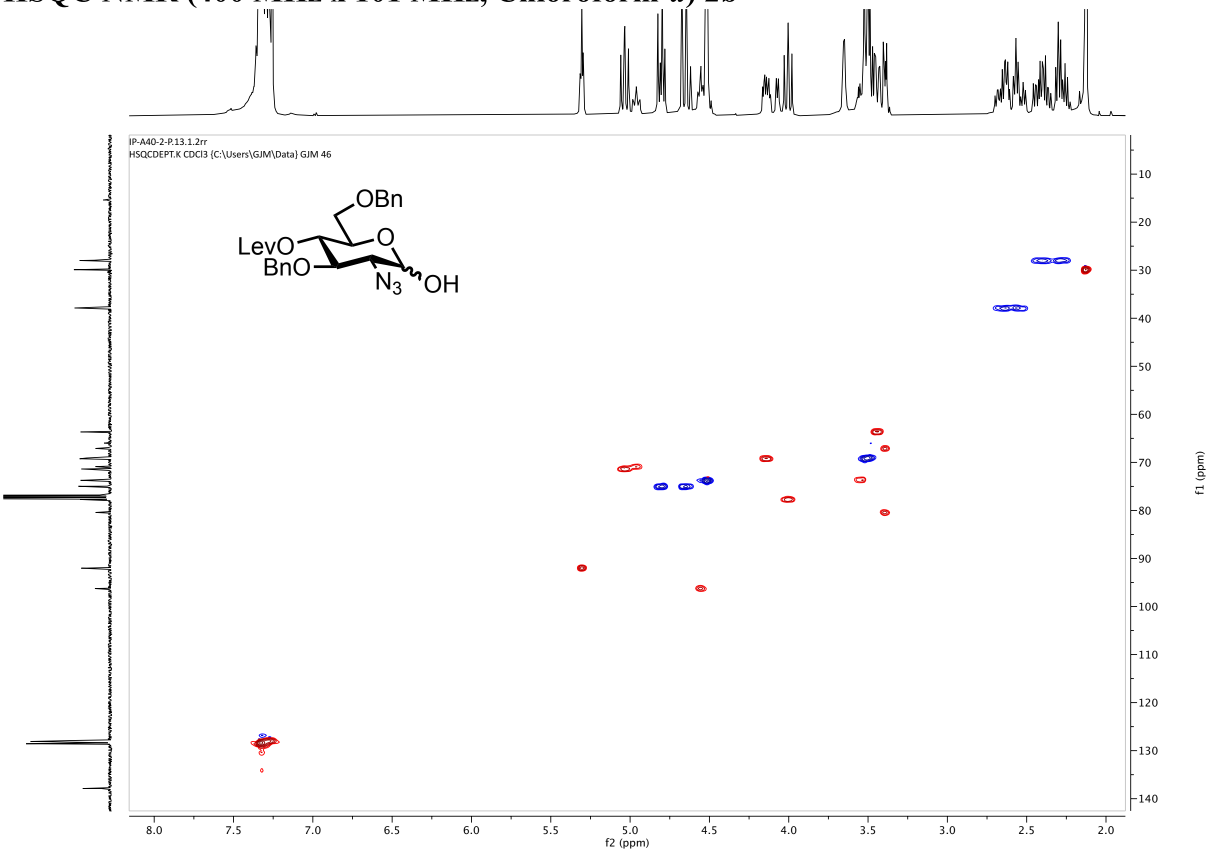

HMBC NMR (400 MHz x 101 MHz, Chloroform-*d*) 2b

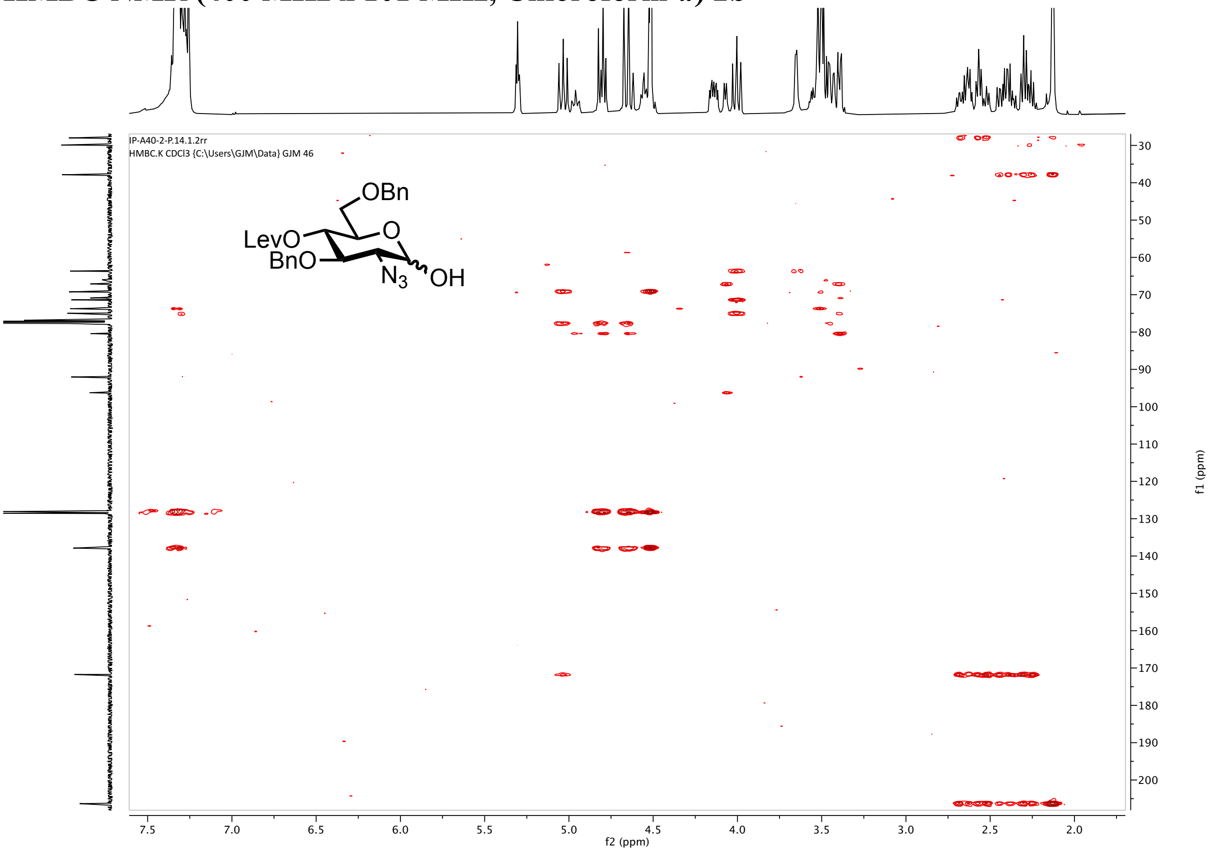

Compound 1c

^{1}H NMR (400 MHz, Chloroform-*d*) 1c

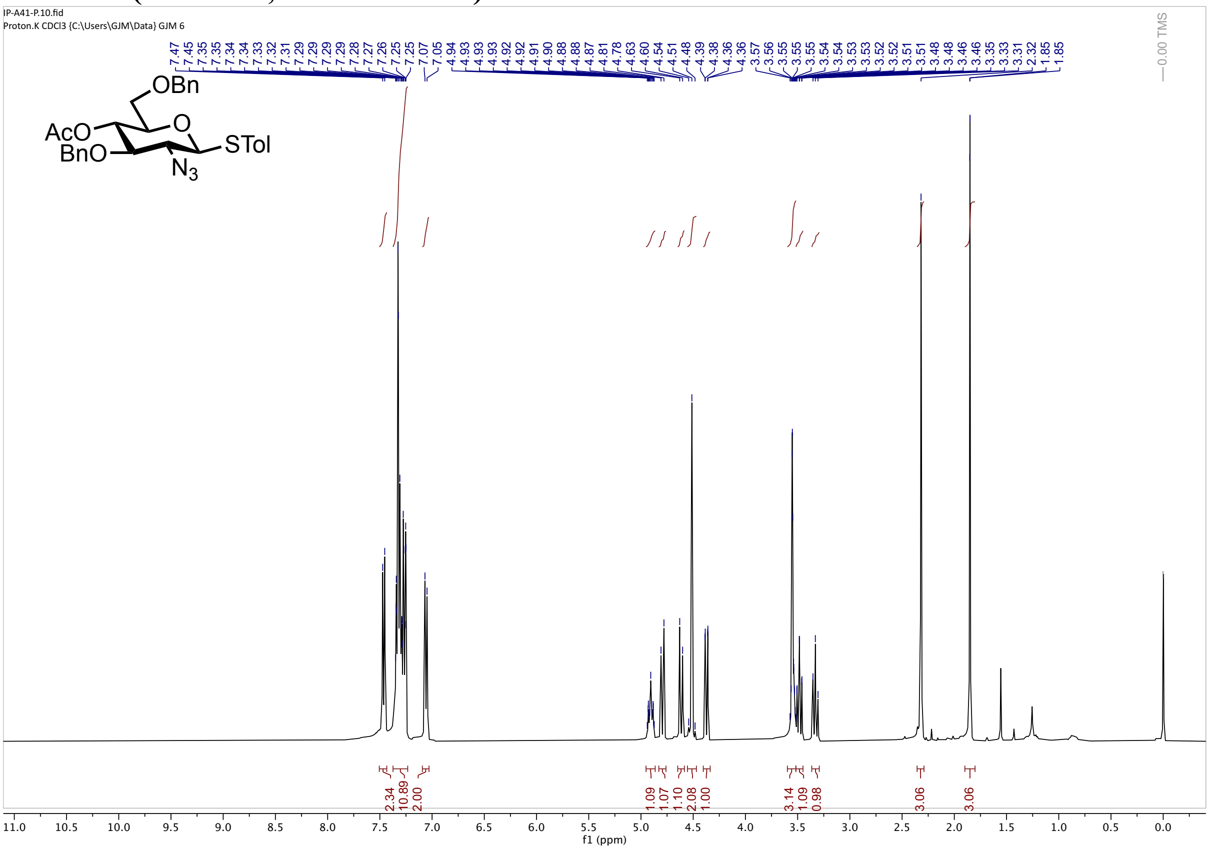

^{13}C{^{1}H} NMR (101 MHz, Chloroform-*d*) 1c

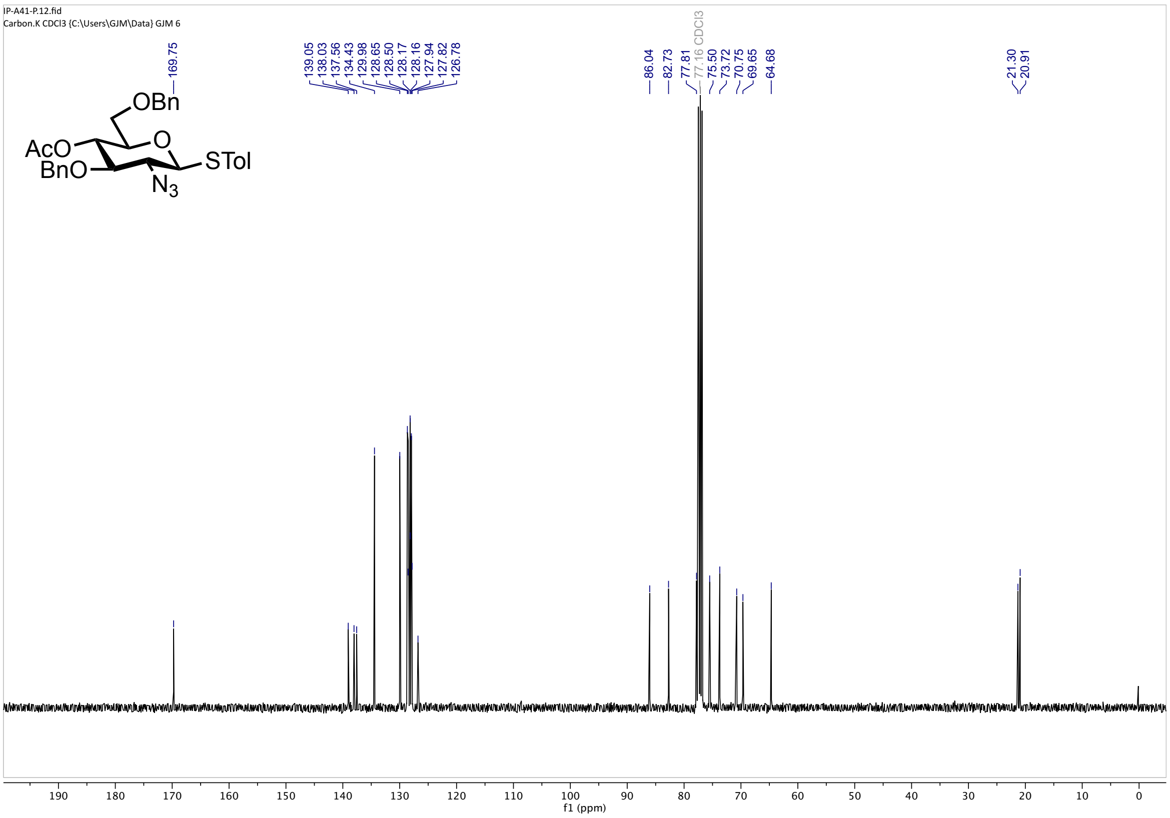

Compound 2c

^{1}H NMR (400 MHz, Chloroform-*d*) 2c

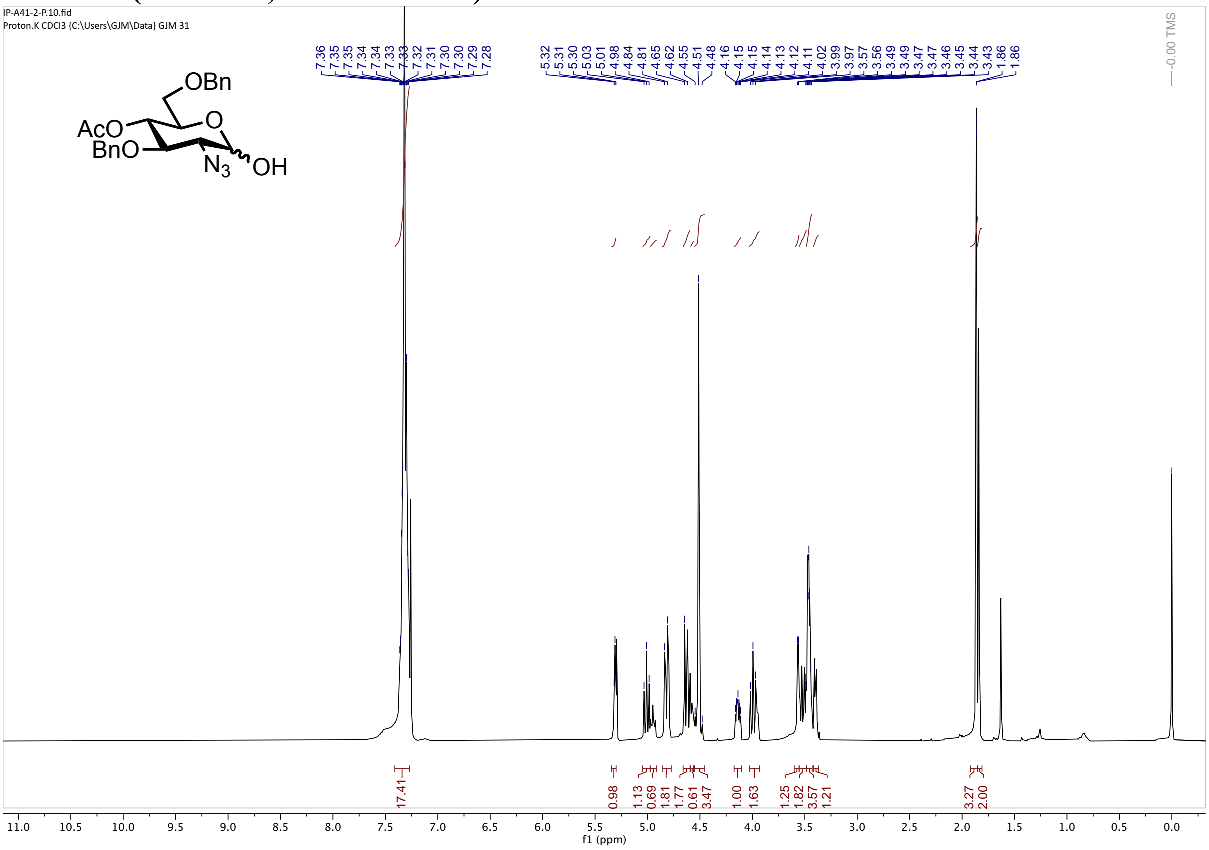

$^{13}C\{^1H\}$ NMR (101 MHz, Chloroform-*d*) 2c

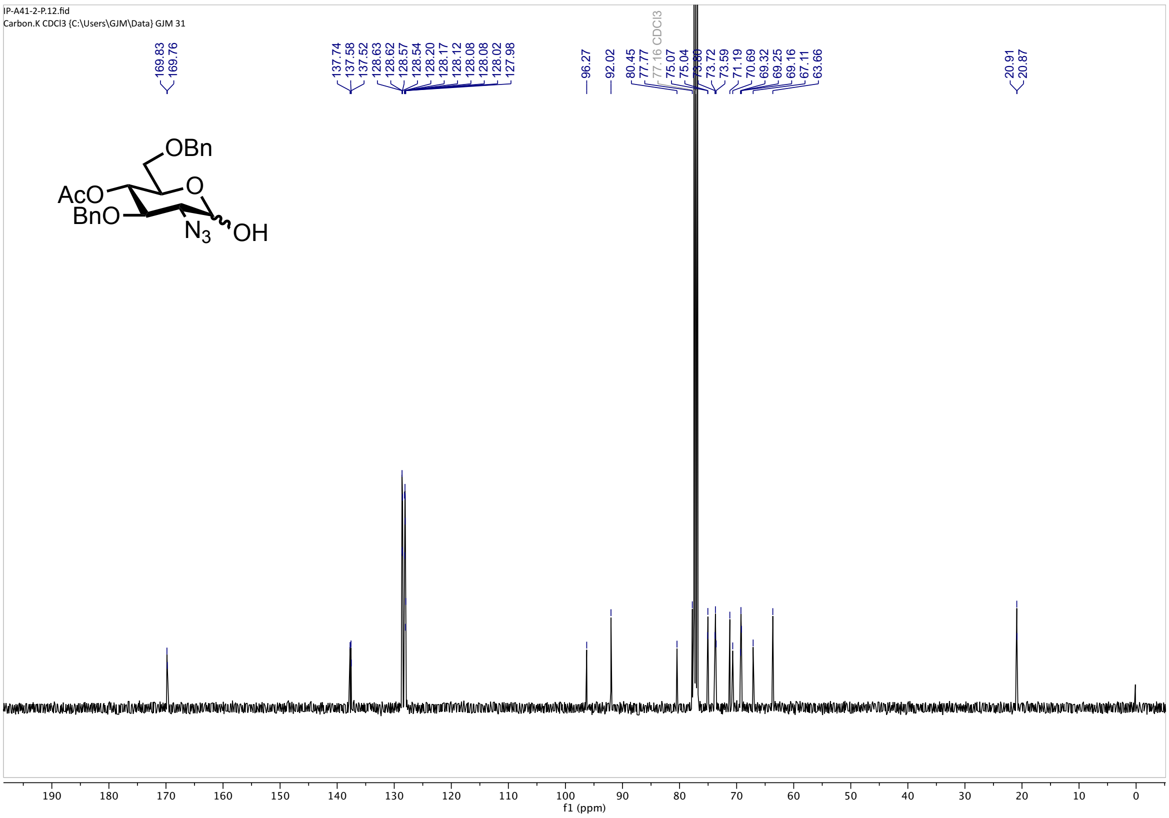

Compound S15

^{1}H NMR (400 MHz, Chloroform-*d*) S15

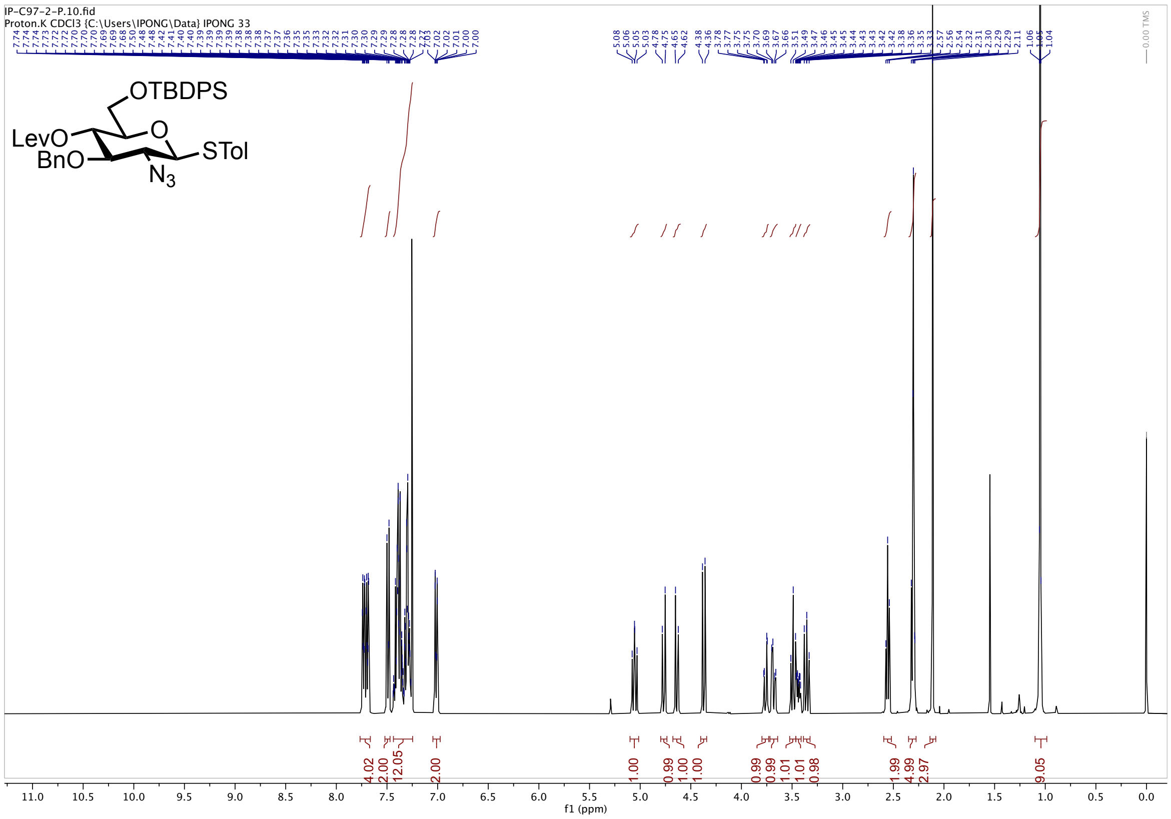

^{13}C{^{1}H} NMR (101 MHz, Chloroform-*d*) S15

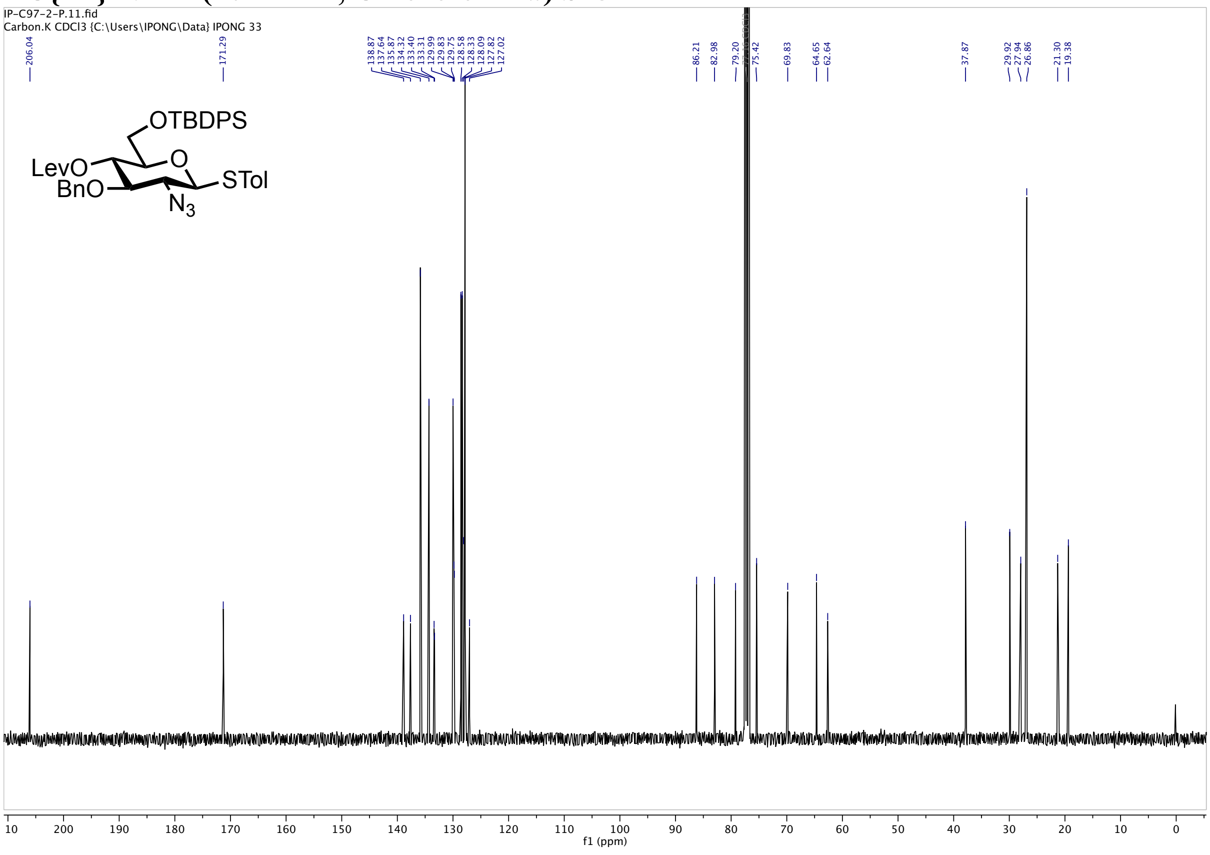

COSY NMR (400 MHz, Chloroform-*d*) S15

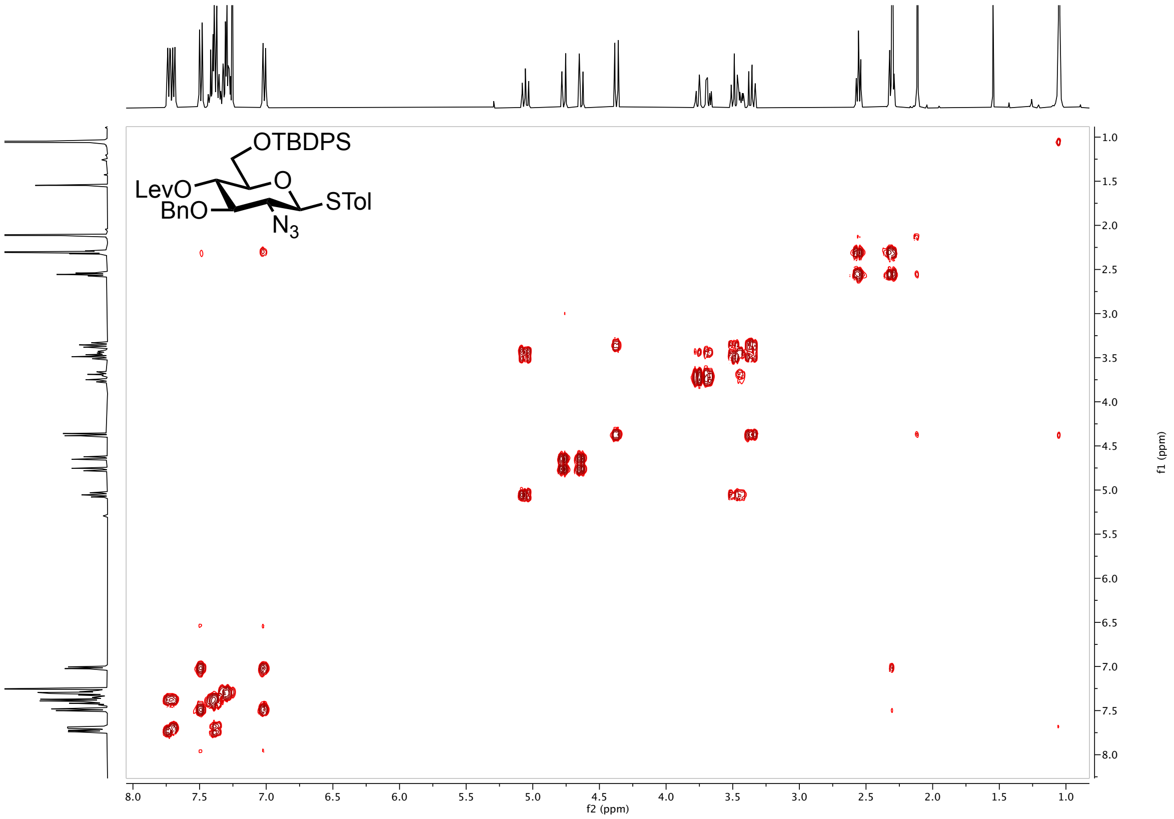

HSQC NMR (400 MHz x 101 MHz, Chloroform-*d*) S15

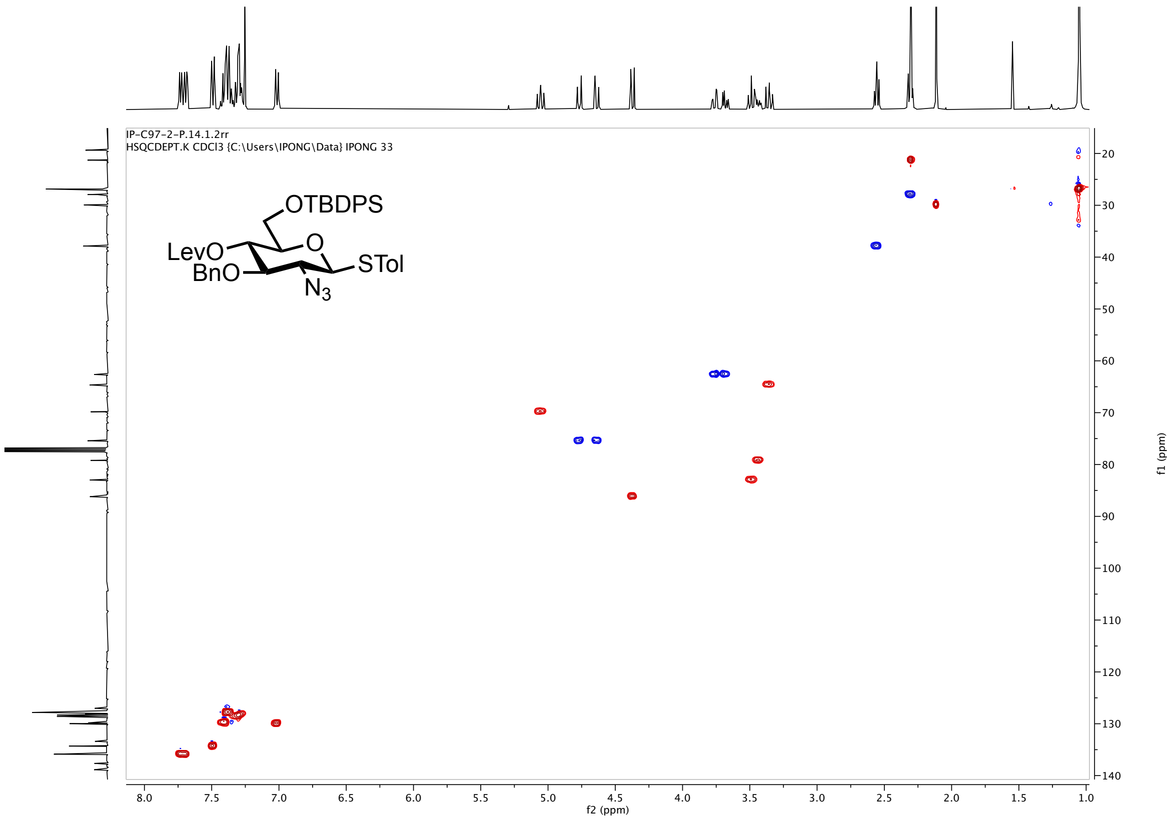

Compound 3a
^{1}H NMR (400 MHz, Chloroform-*d*) 3a

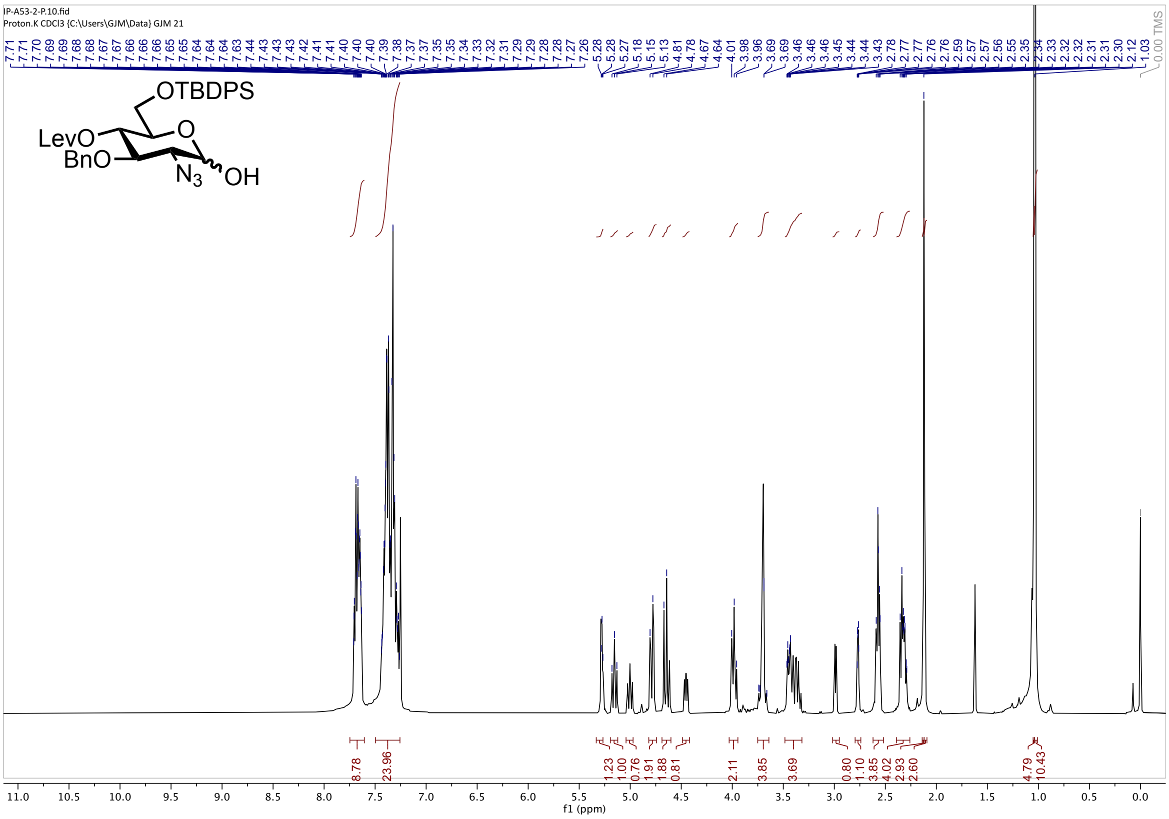

$^{13}C\{^1H\}$ NMR (101 MHz, Chloroform-*d*) 3a

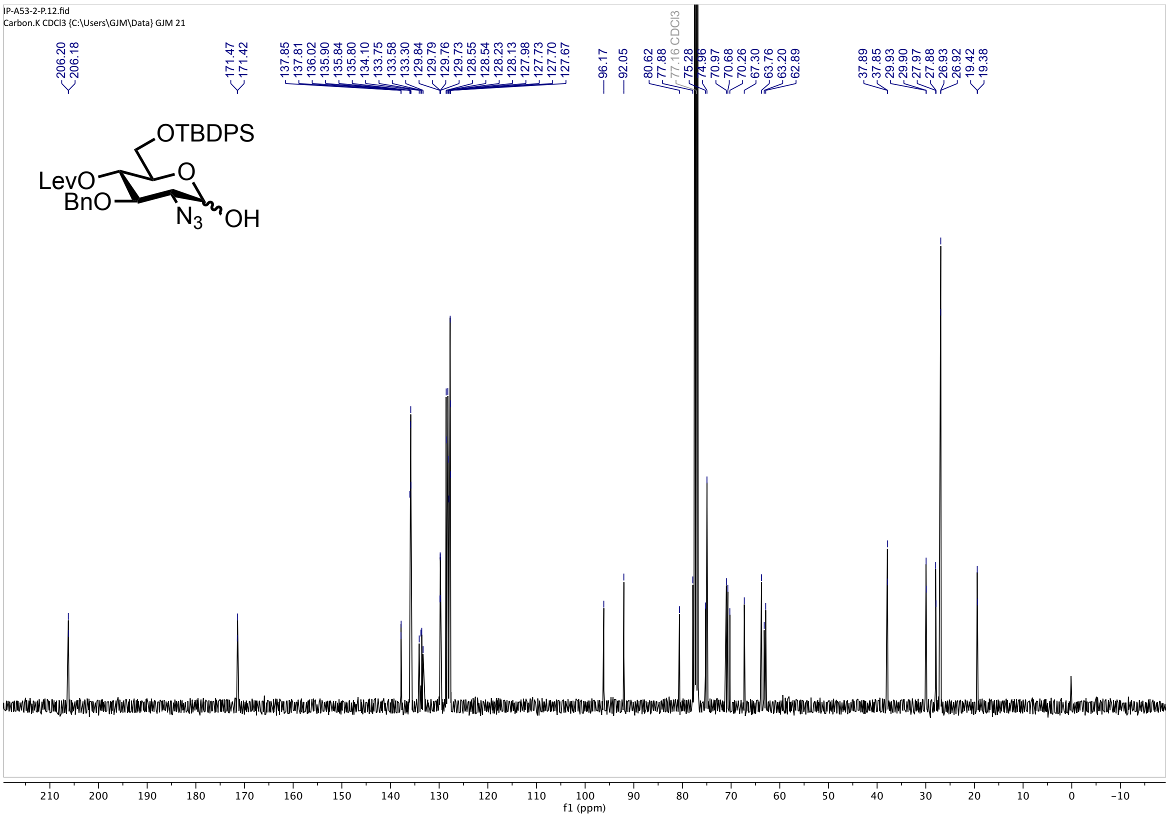

COSY NMR (400 MHz, Chloroform-*d*) 3a

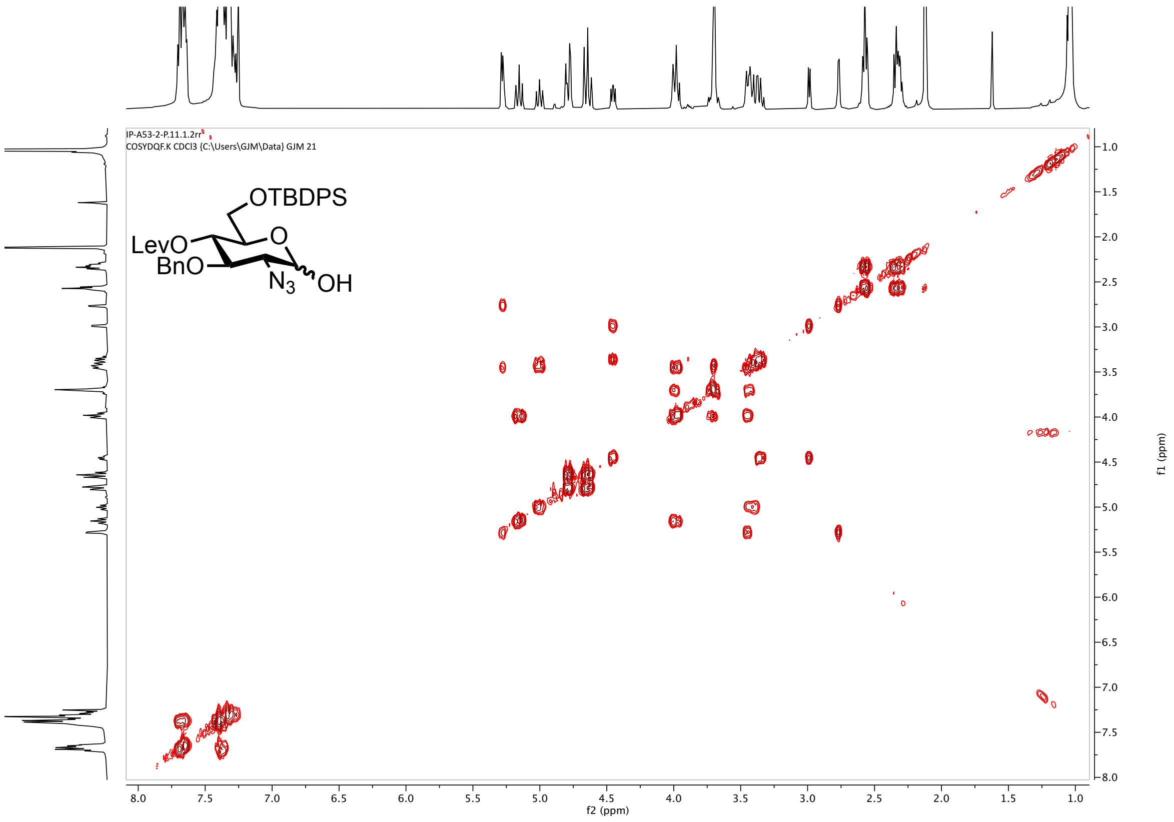

HSQC NMR (400 MHz x 101 MHz, Chloroform-*d*) 3a

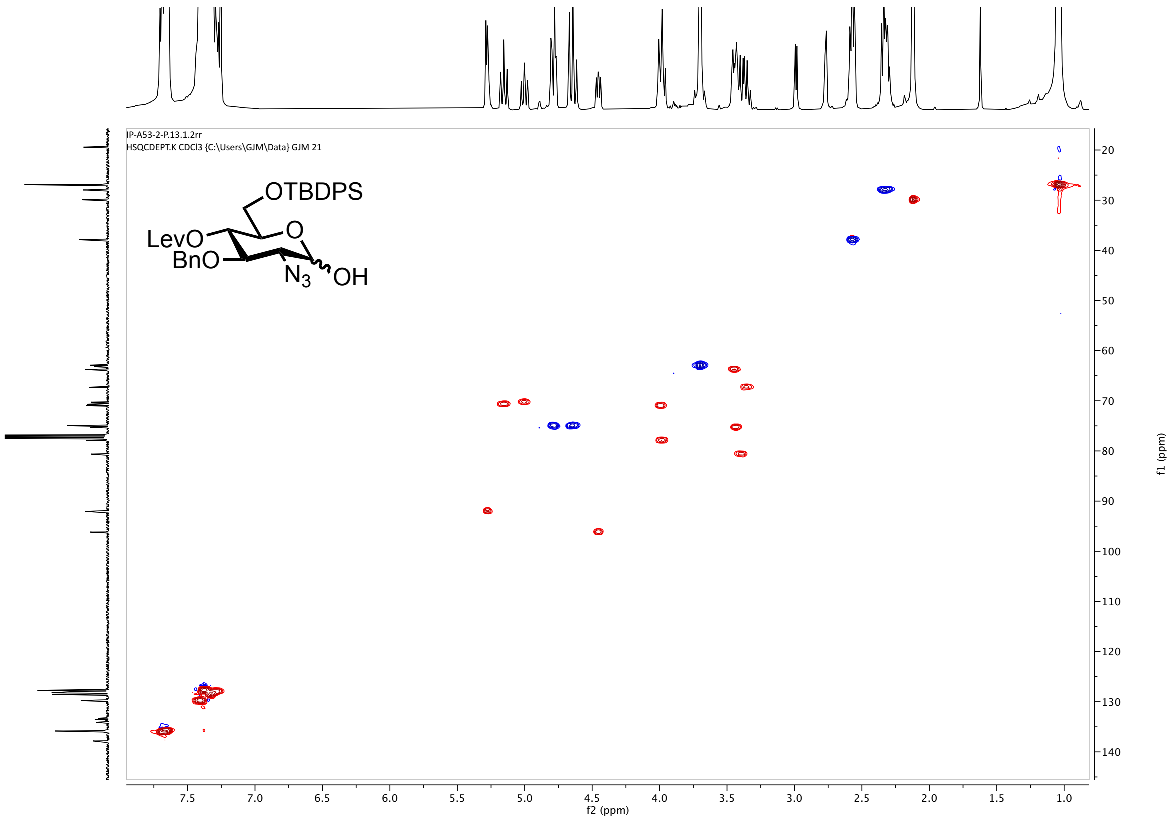

HMBC NMR (400 MHz x 101 MHz, Chloroform-*d*) 3a

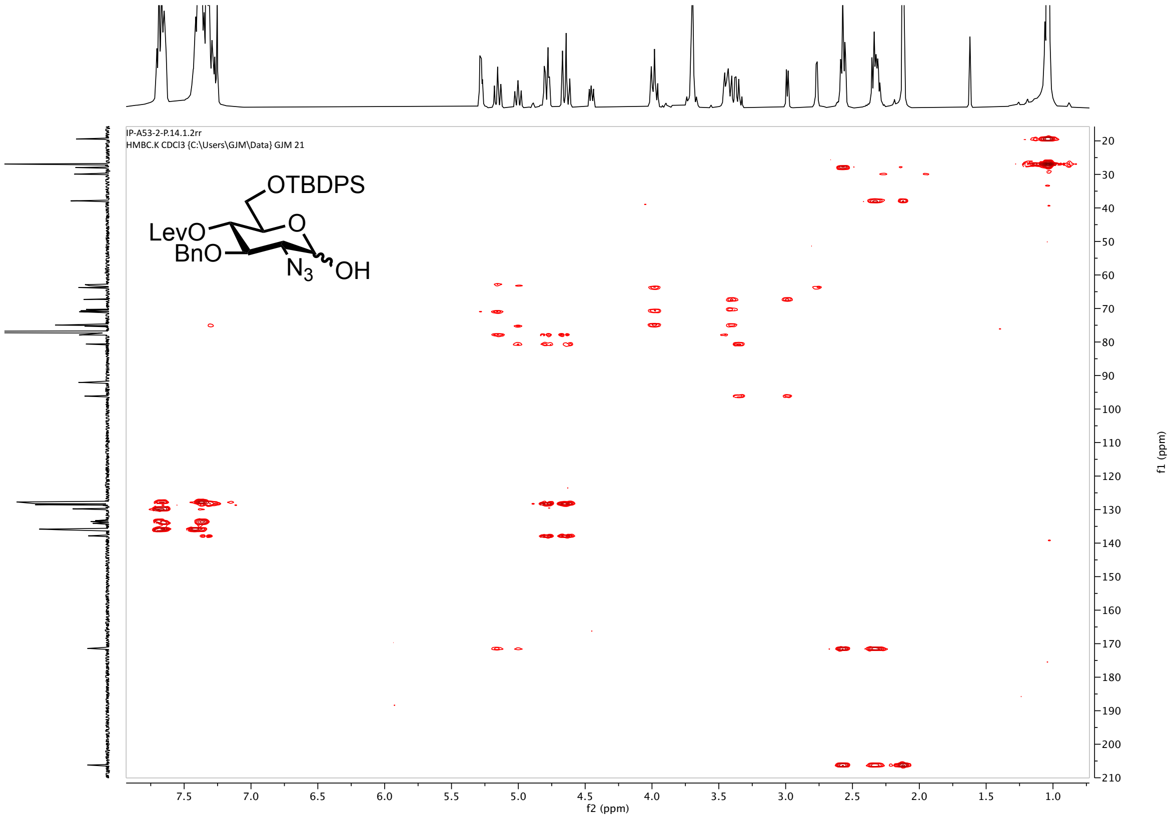

Compound S16

^{1}H NMR (400 MHz, Chloroform-*d*) S16

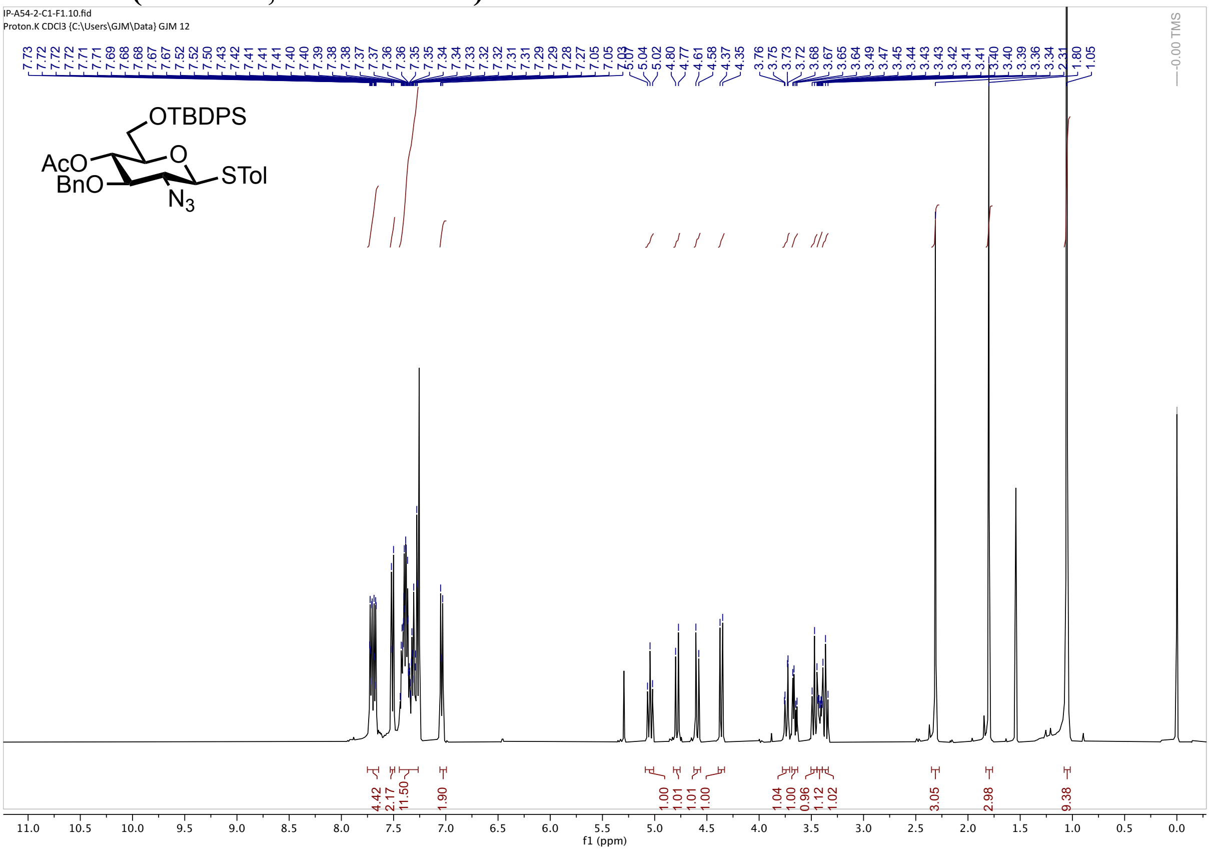

Compound 3b

^{1}H NMR (400 MHz, Chloroform-*d*) 3b

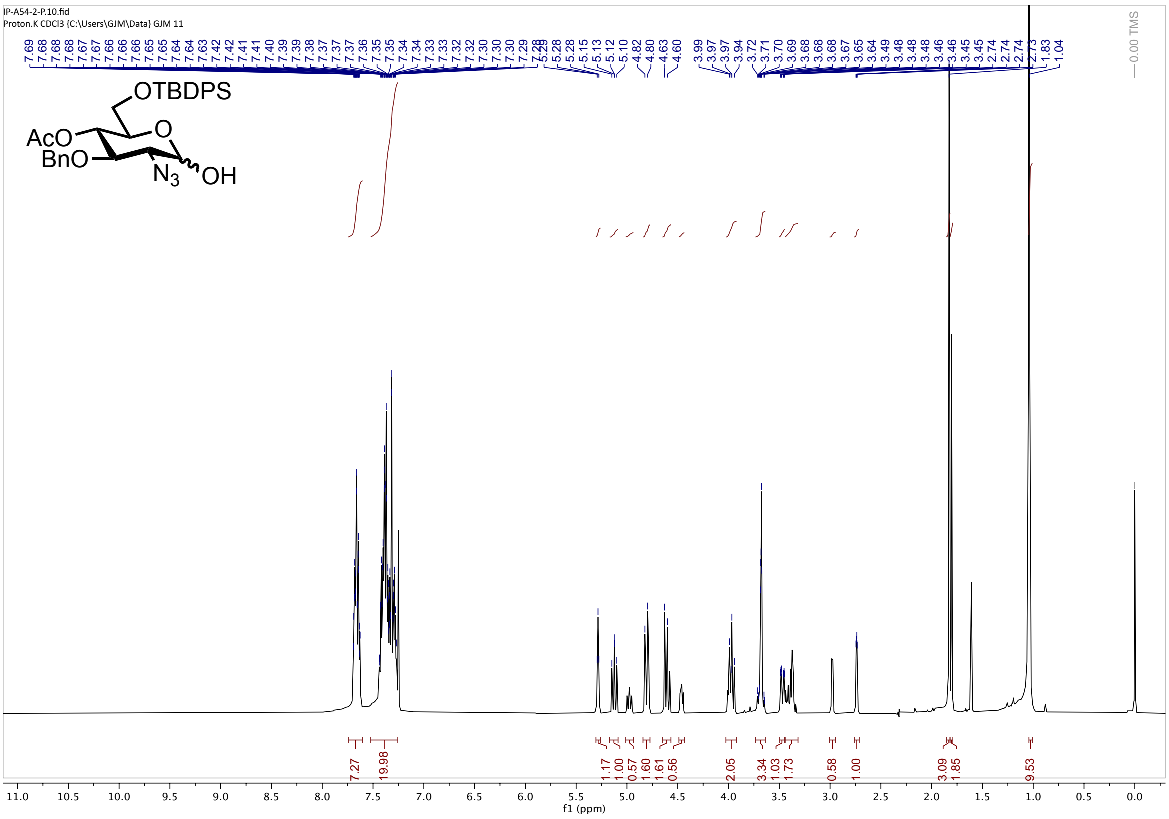

^{13}C{^{1}H} NMR (101 MHz, Chloroform-*d*) 3b

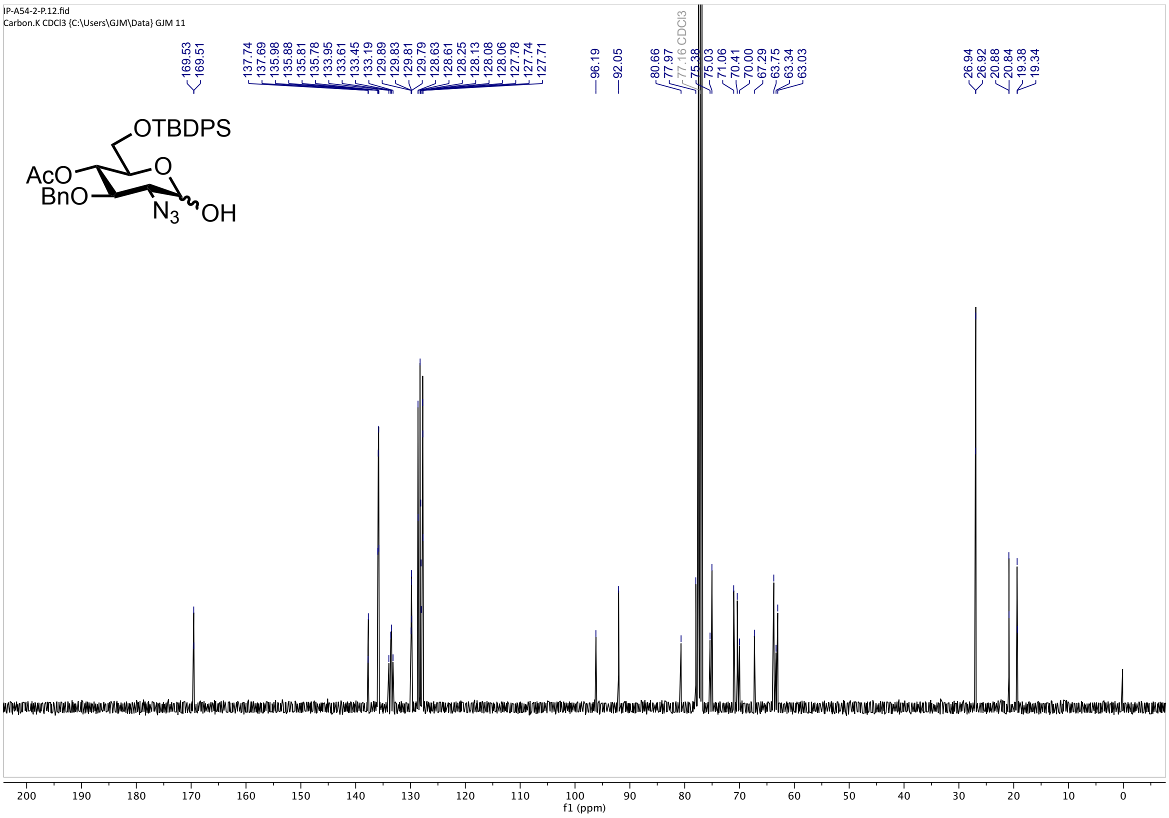

COSY NMR (400 MHz, Chloroform-*d*) 3b

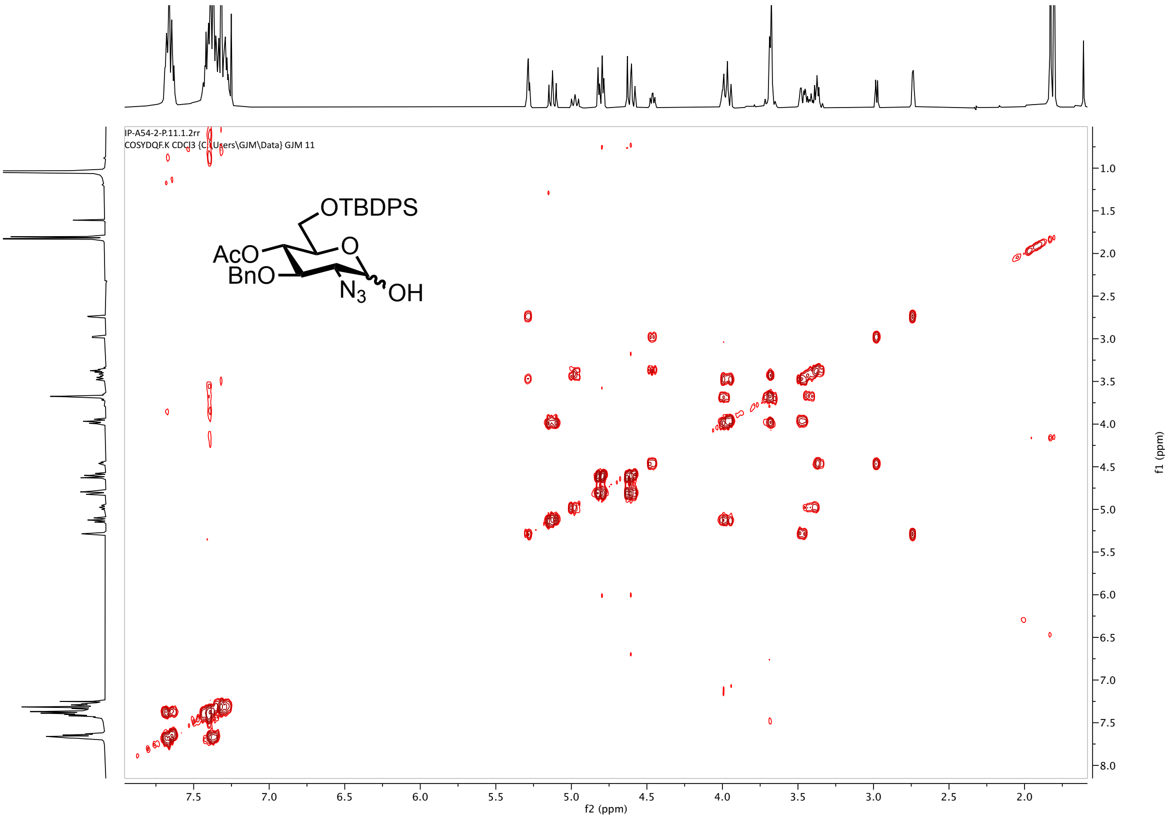

HSQC NMR (400 MHz x 101 MHz, Chloroform-*d*) 3b

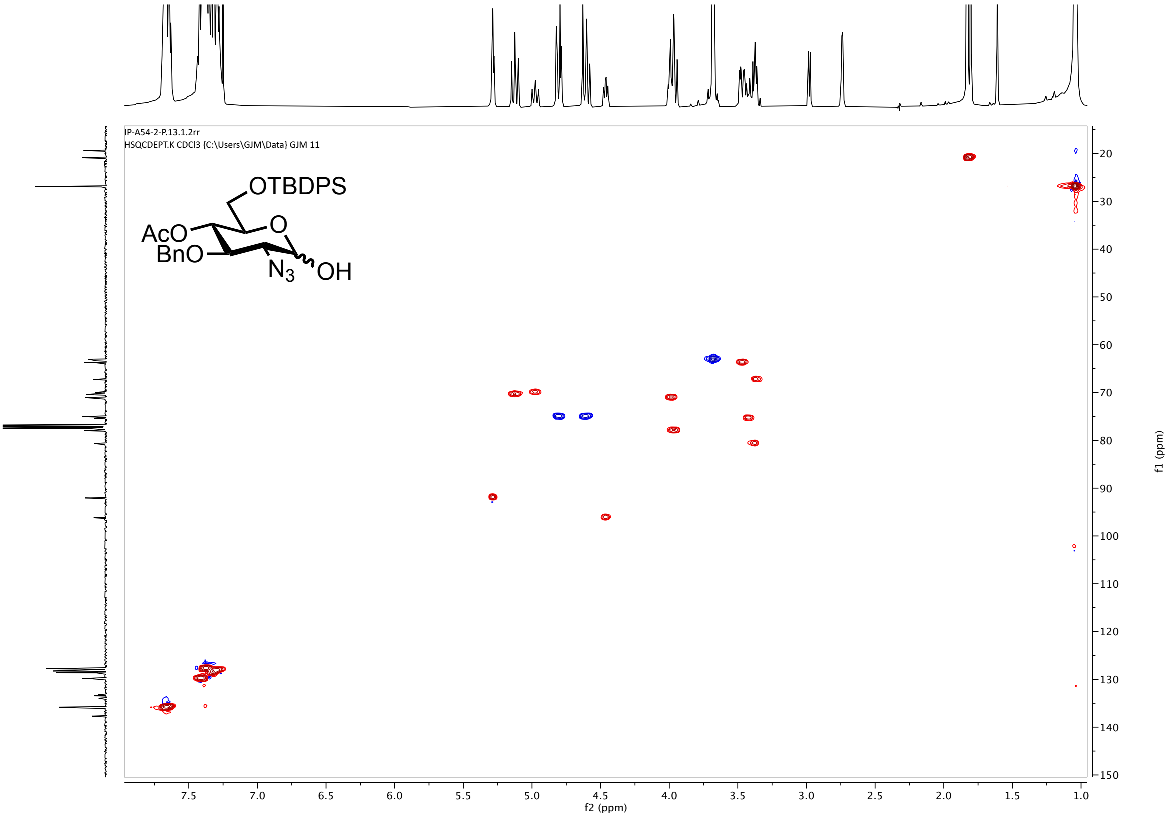

HMBC NMR (400 MHz x 101 MHz, Chloroform-*d*) 3b

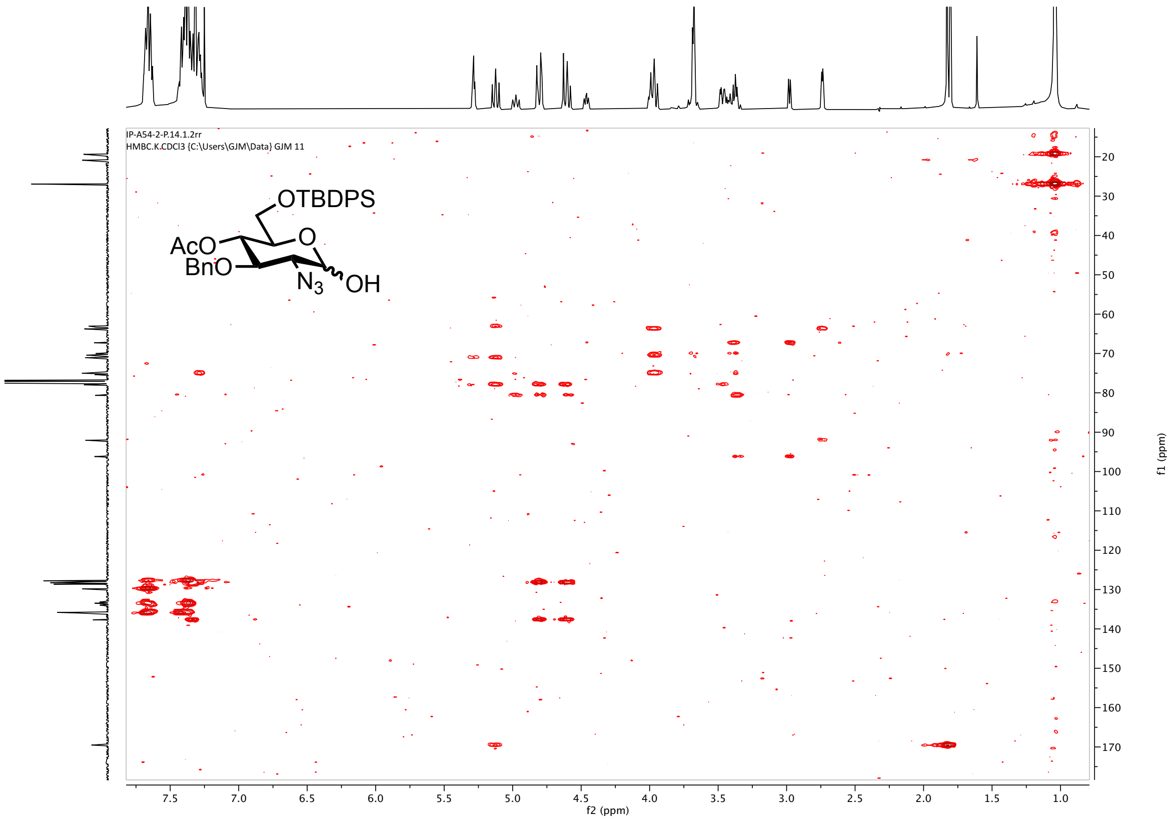

Compound S17

^{1}H NMR (400 MHz, Chloroform-*d*) S17

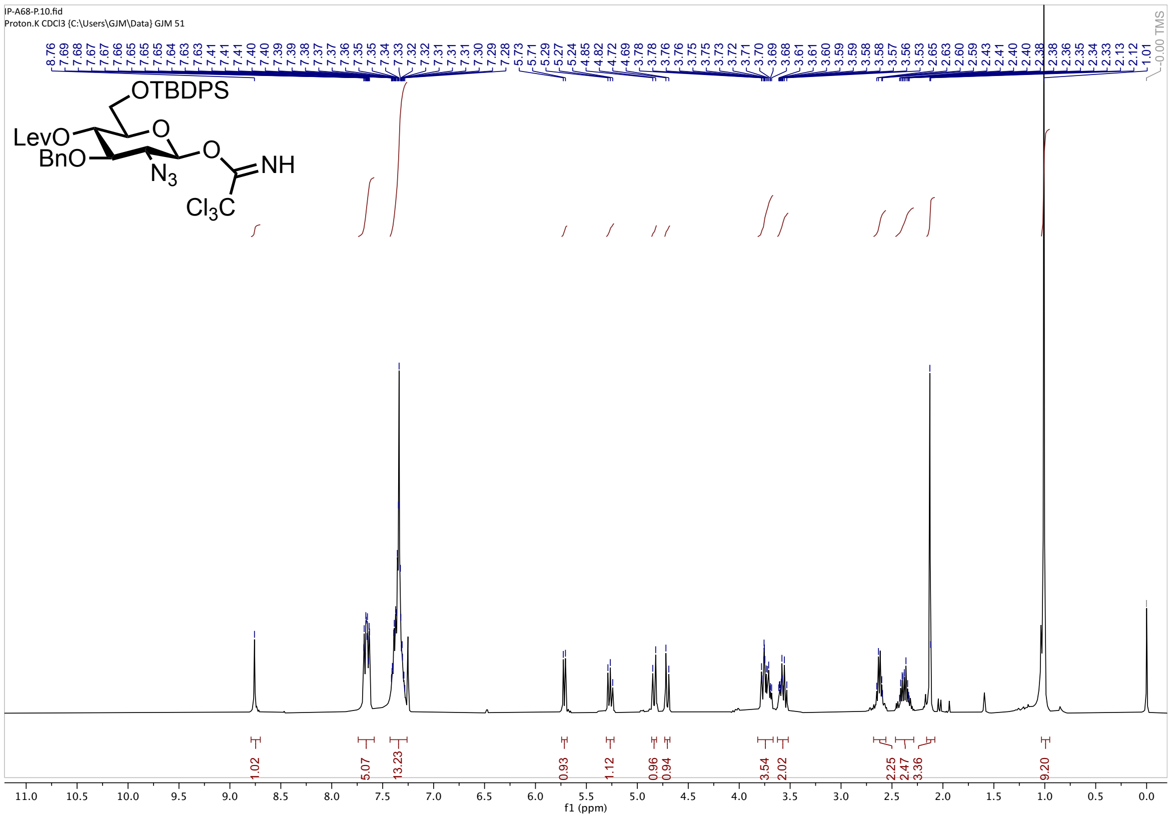

$^{13}C\{^{1}H\}$ NMR (101 MHz, Chloroform-*d*) S17

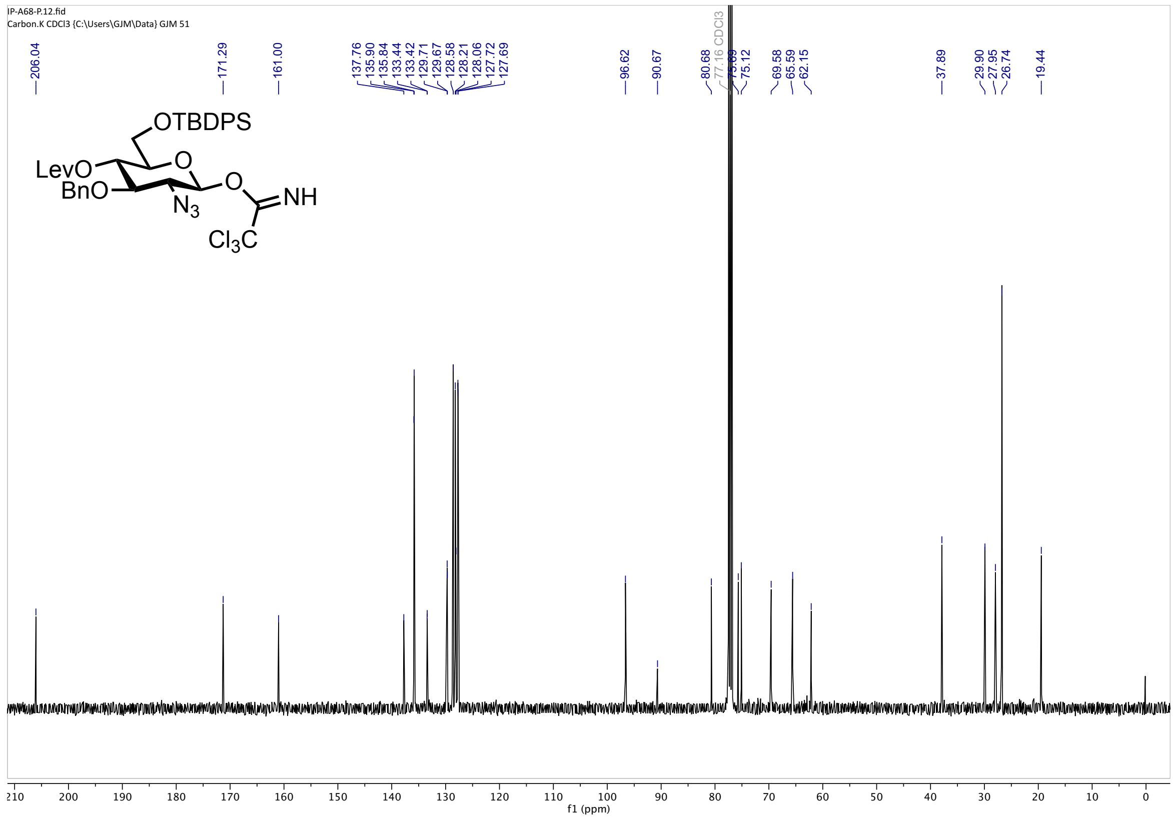

COSY NMR (400 MHz, Chloroform-*d*) S17

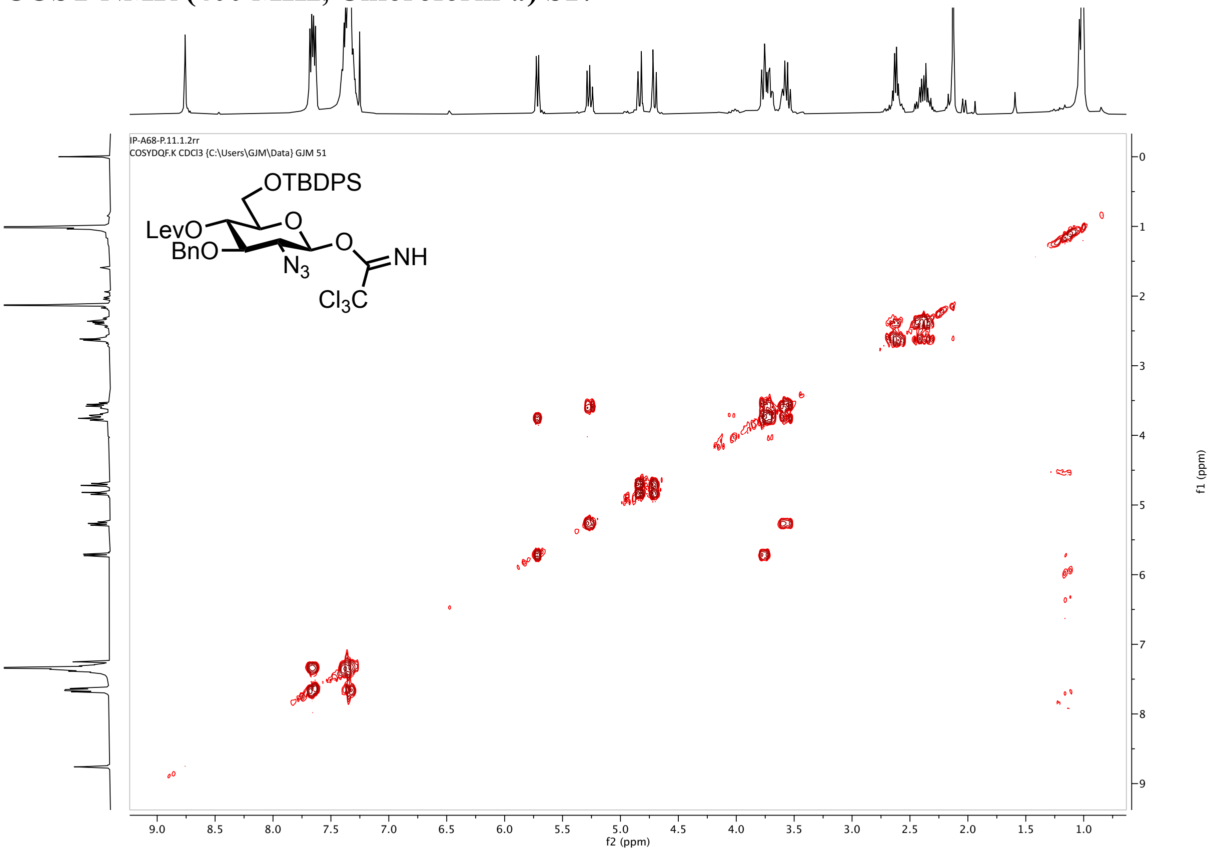

HSQC NMR (400 MHz x 101 MHz, Chloroform-*d*) S17

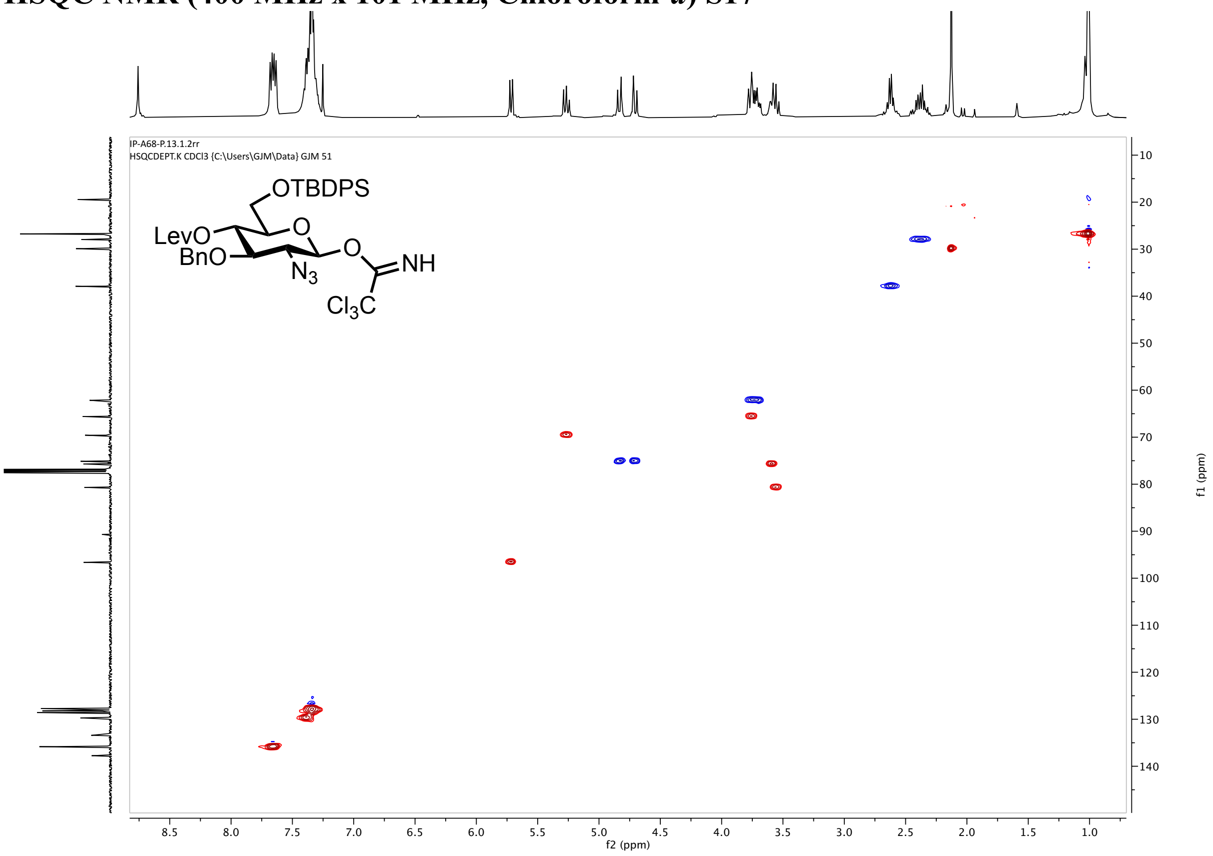

HMBC NMR (400 MHz x 101 MHz, Chloroform-*d*) S17

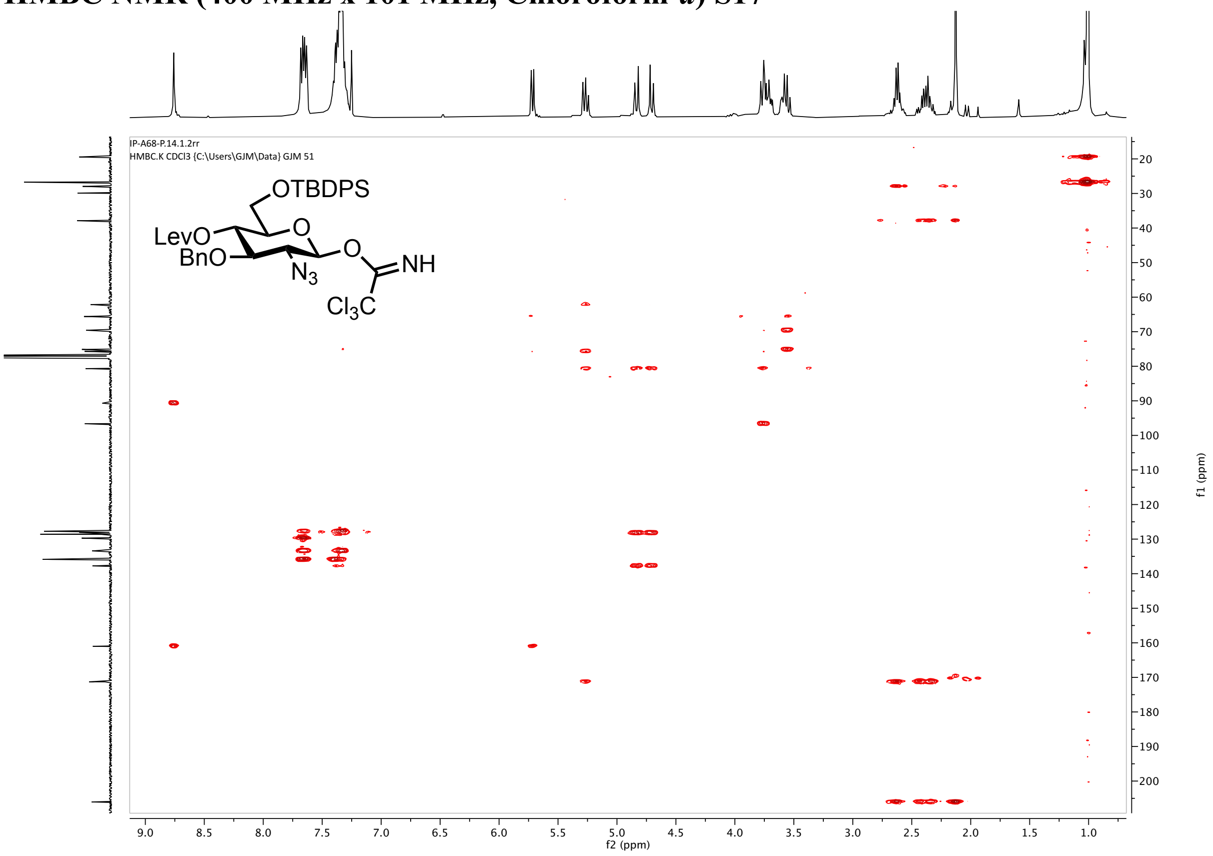

Glycosylations

Compound 5a

^{1}H NMR (400 MHz, Chloroform-*d*) 5a

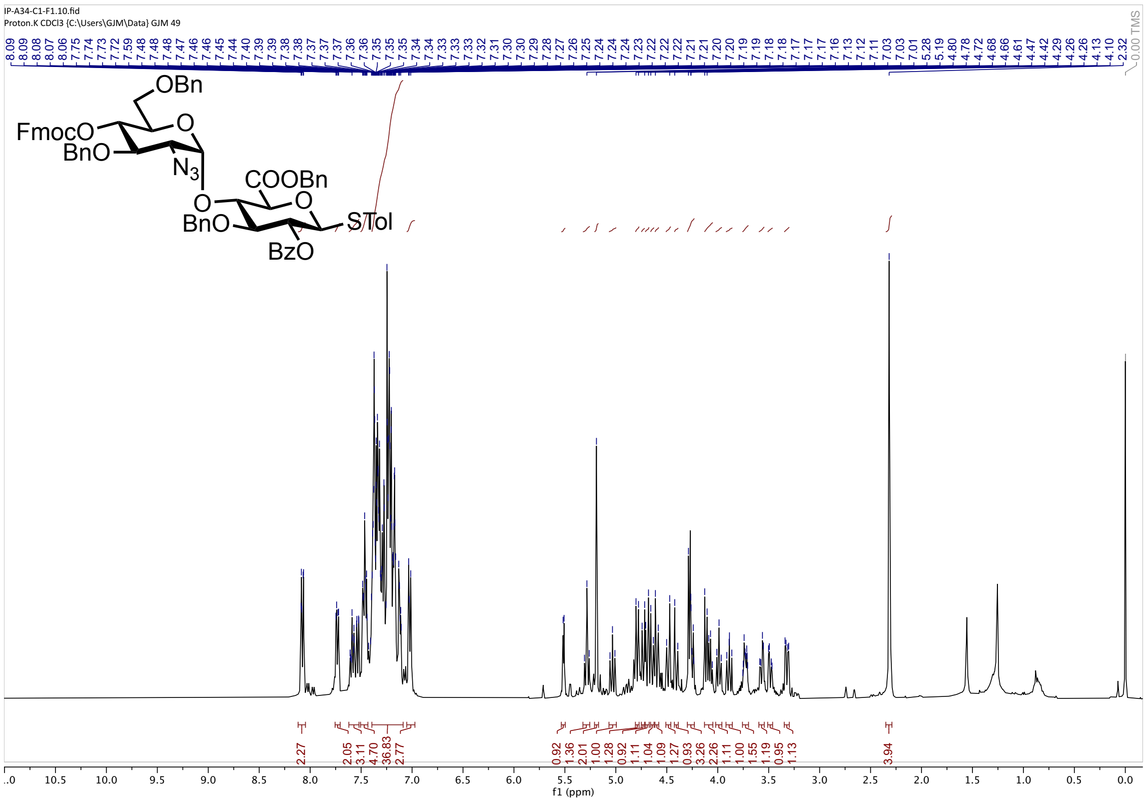

^{13}C{^{1}H} NMR (101 MHz, Chloroform-*d*) 5a

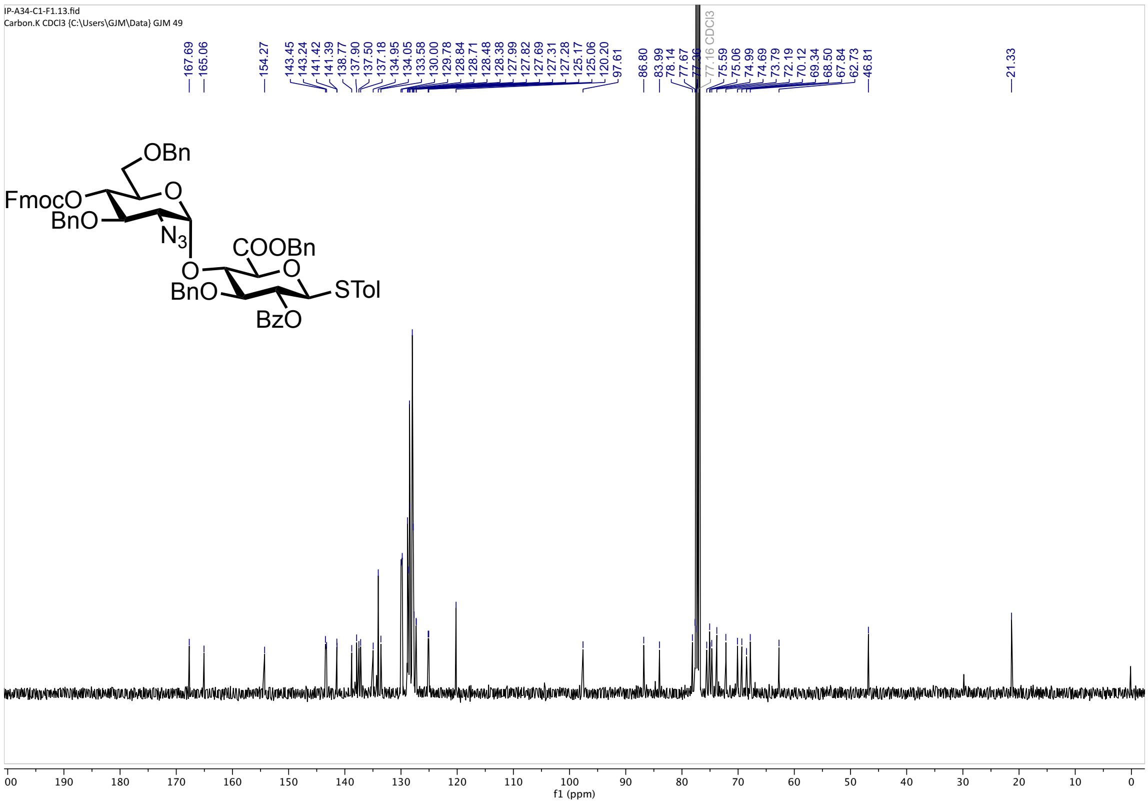

COSY NMR (400 MHz, Chloroform-*d*) 5a

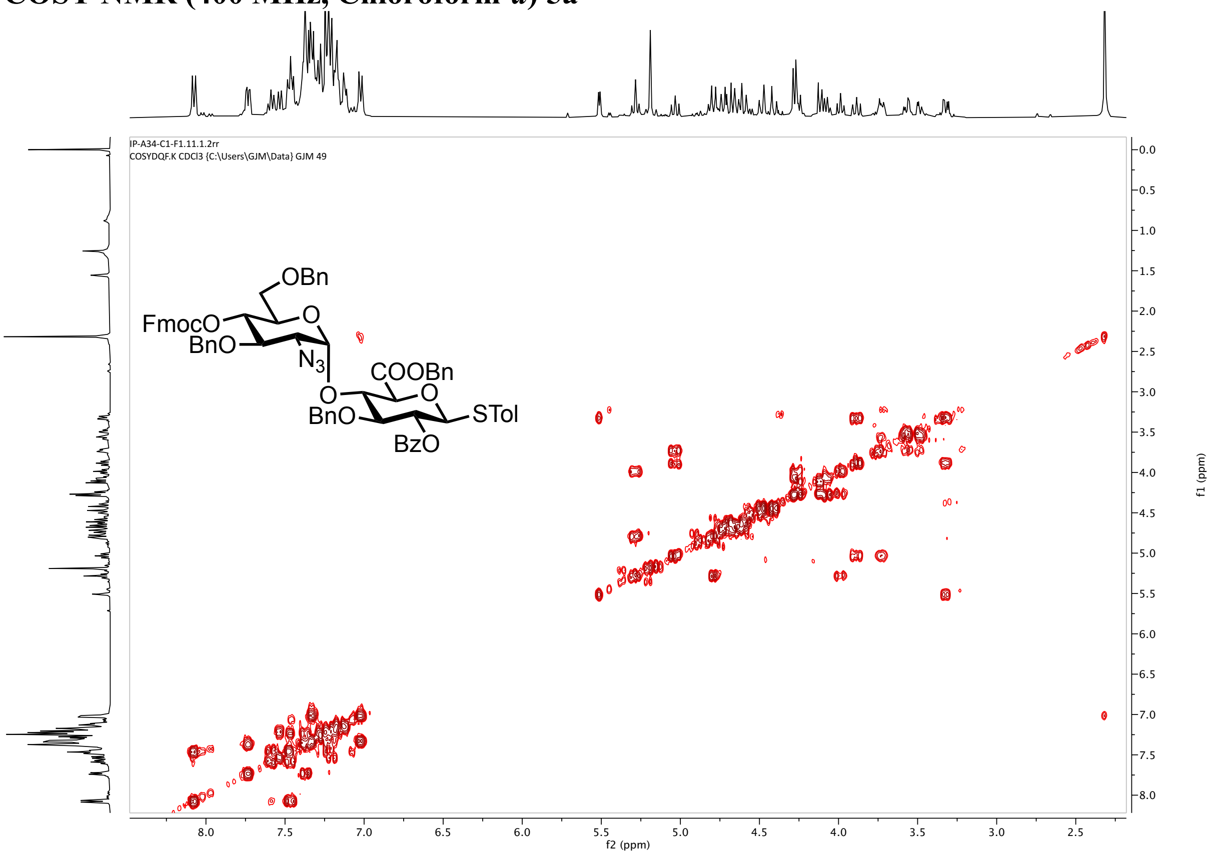

HSQC NMR (400 MHz x 101 MHz, Chloroform-*d*) 5a

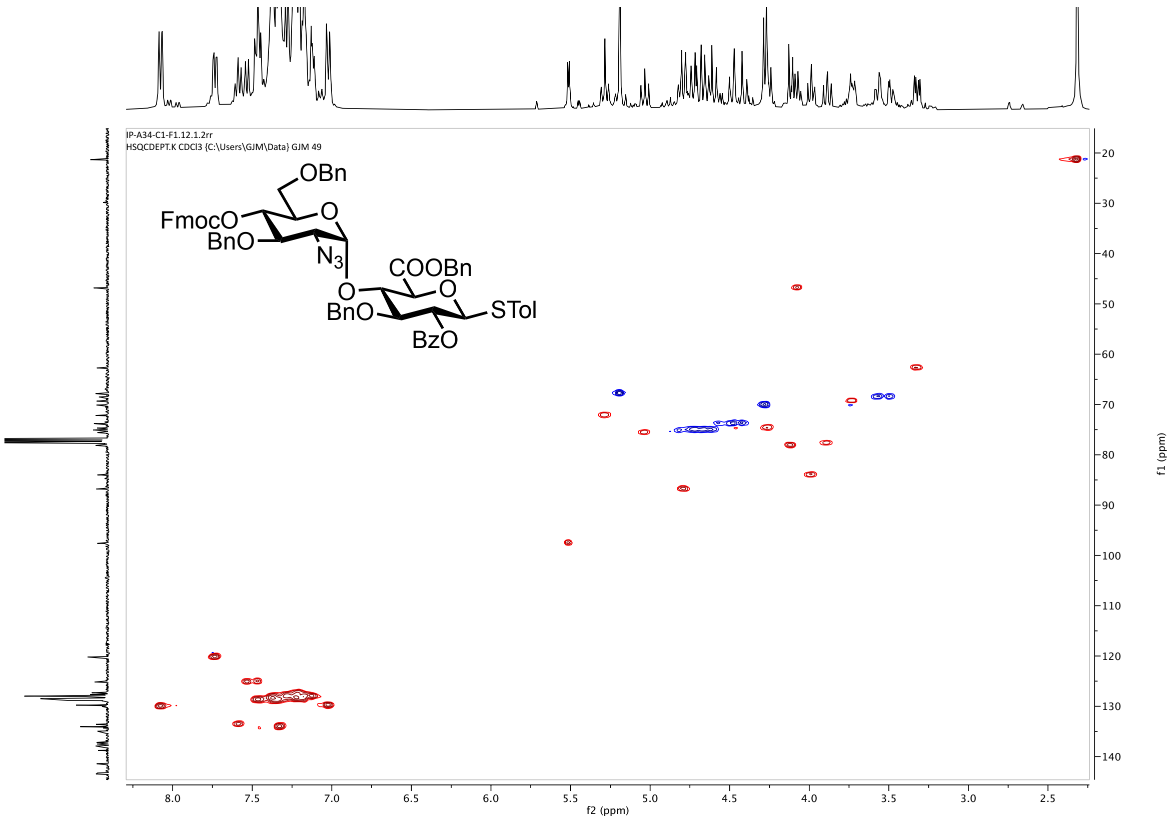

HMBC NMR (400 MHz x 101 MHz, Chloroform-*d*) 5a

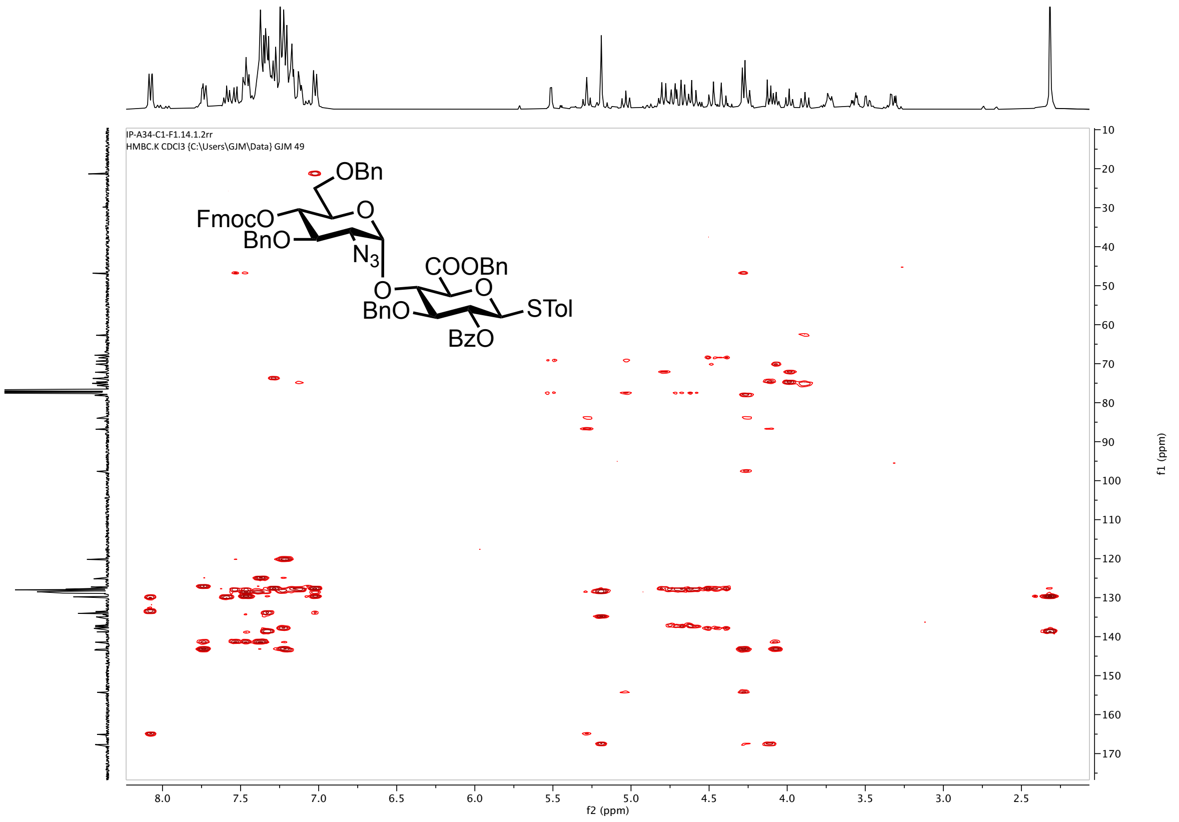

Compound 8a

^{1}H NMR (400 MHz, Chloroform-*d*) inseparable mixture of 8a:4a = 61:39

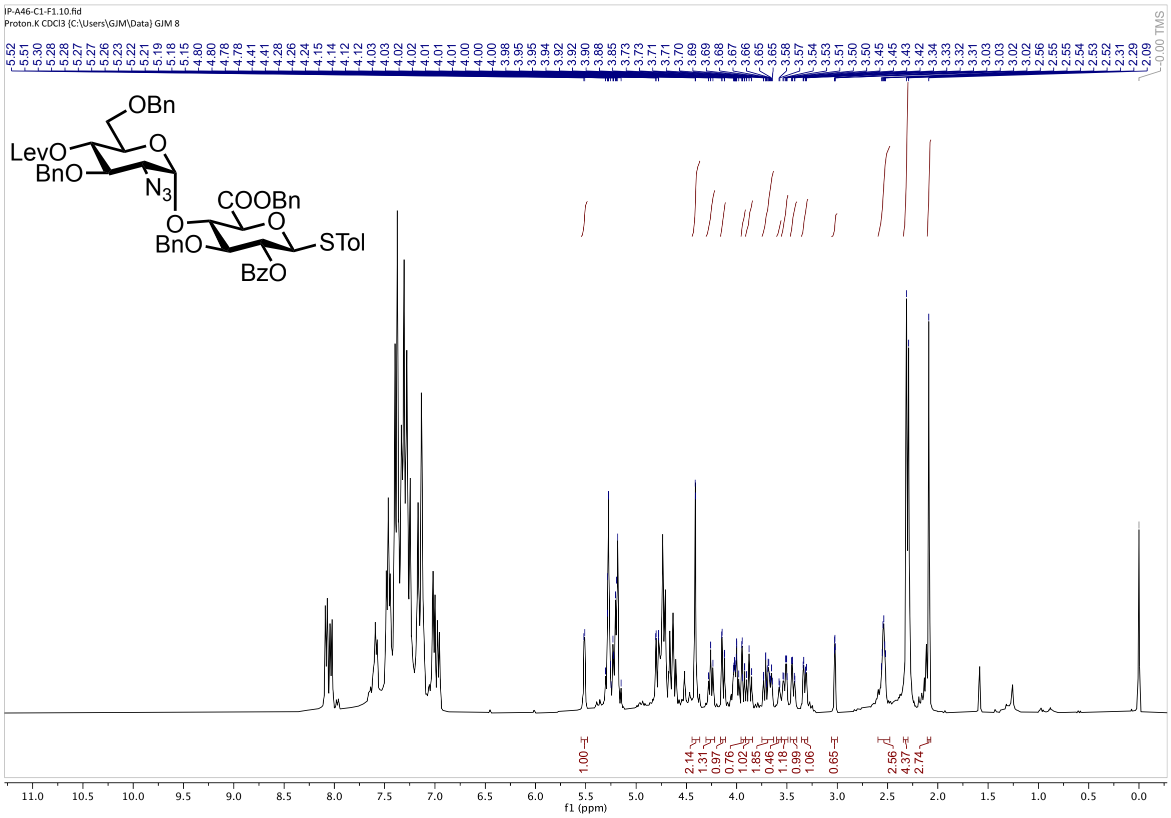

$^{13}C\{^1H\}$ NMR (101 MHz, Chloroform-*d*) inseparable mixture of 8a:4a = 61:39

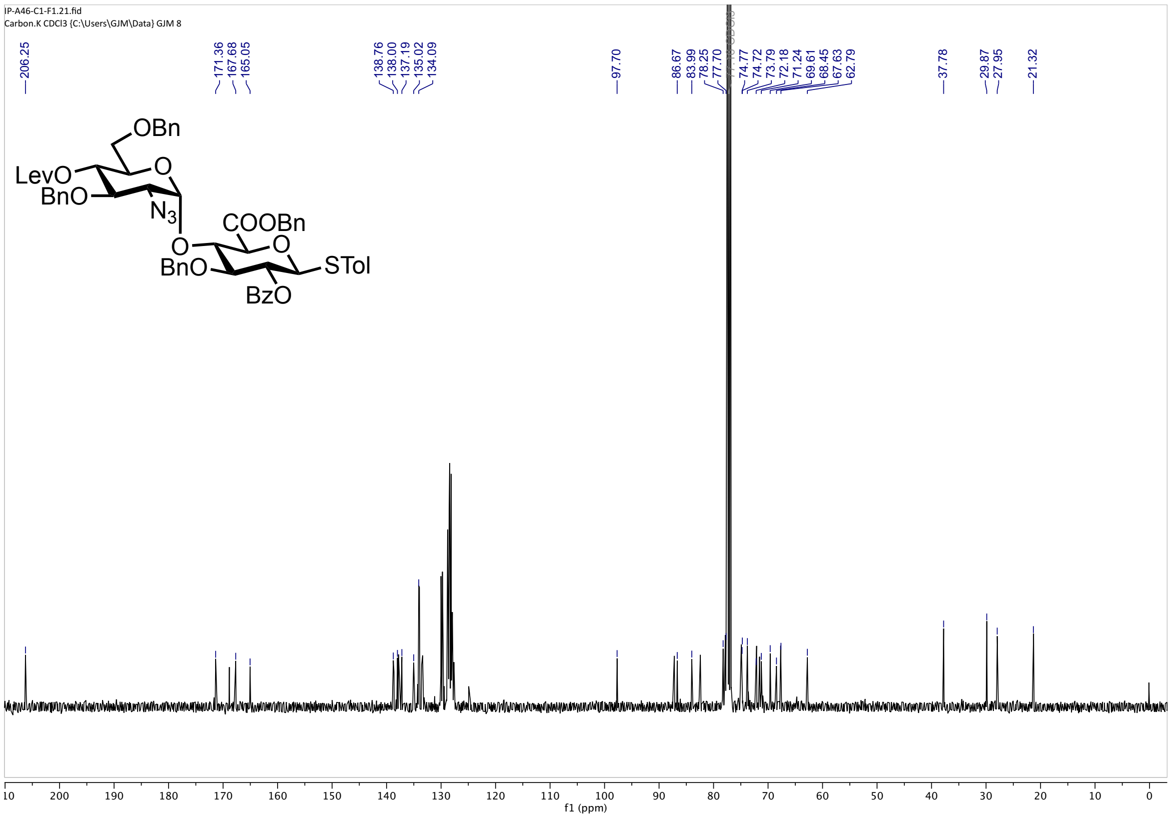

COSY NMR (400 MHz, Chloroform-*d*) inseparable mixture of 8a:4a = 61:39

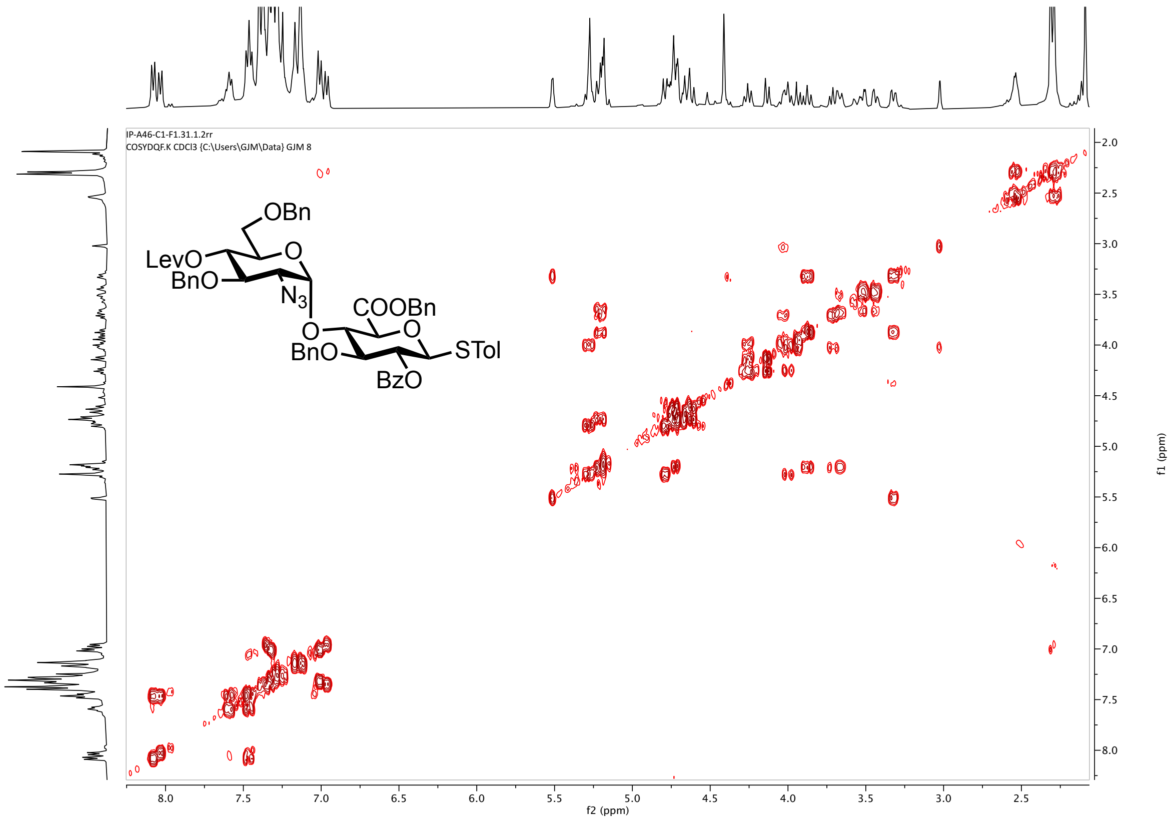

HSQC NMR (400 MHz x 101 MHz, Chloroform-*d*) inseparable mixture of 8a:4a = 61:39

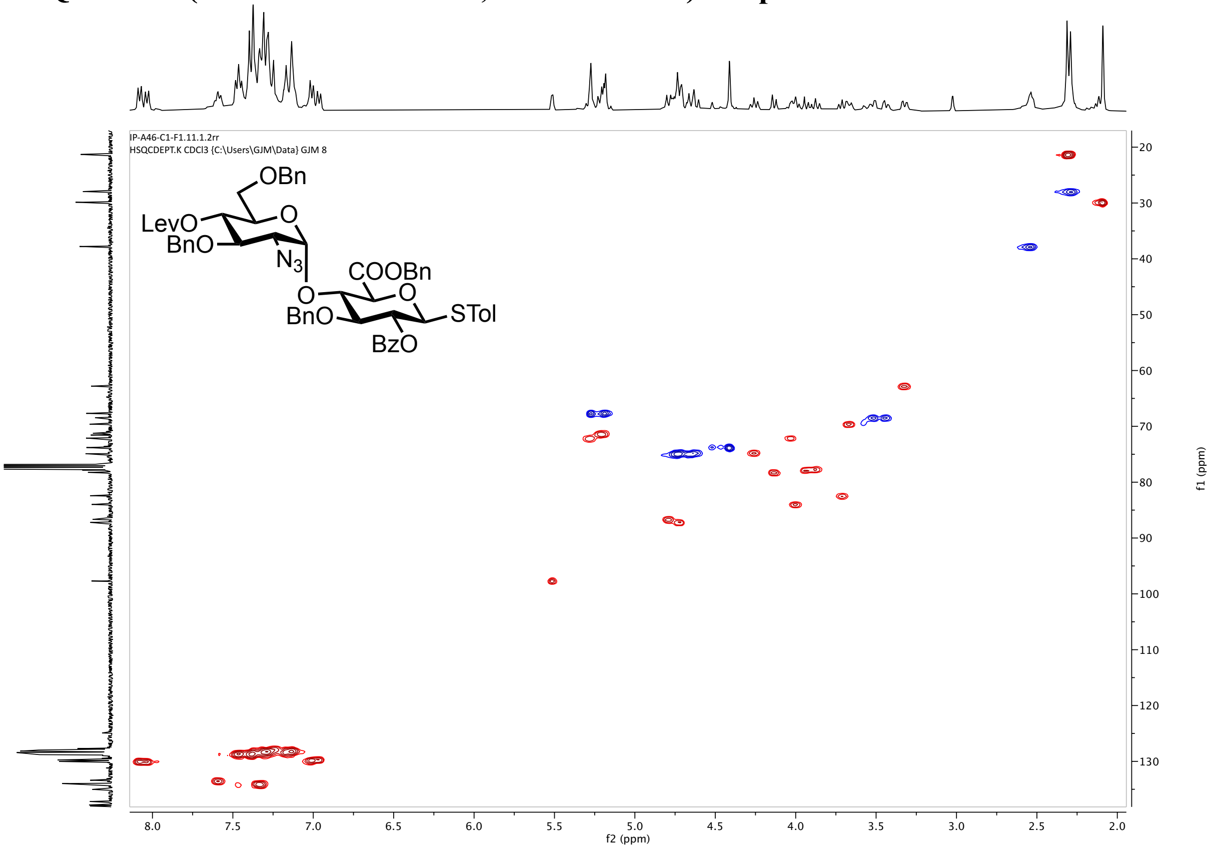

Coupled-HSQC NMR (400 MHz x 101 MHz, Chloroform-*d*) inseparable mixture of 8a:4a = 61:39

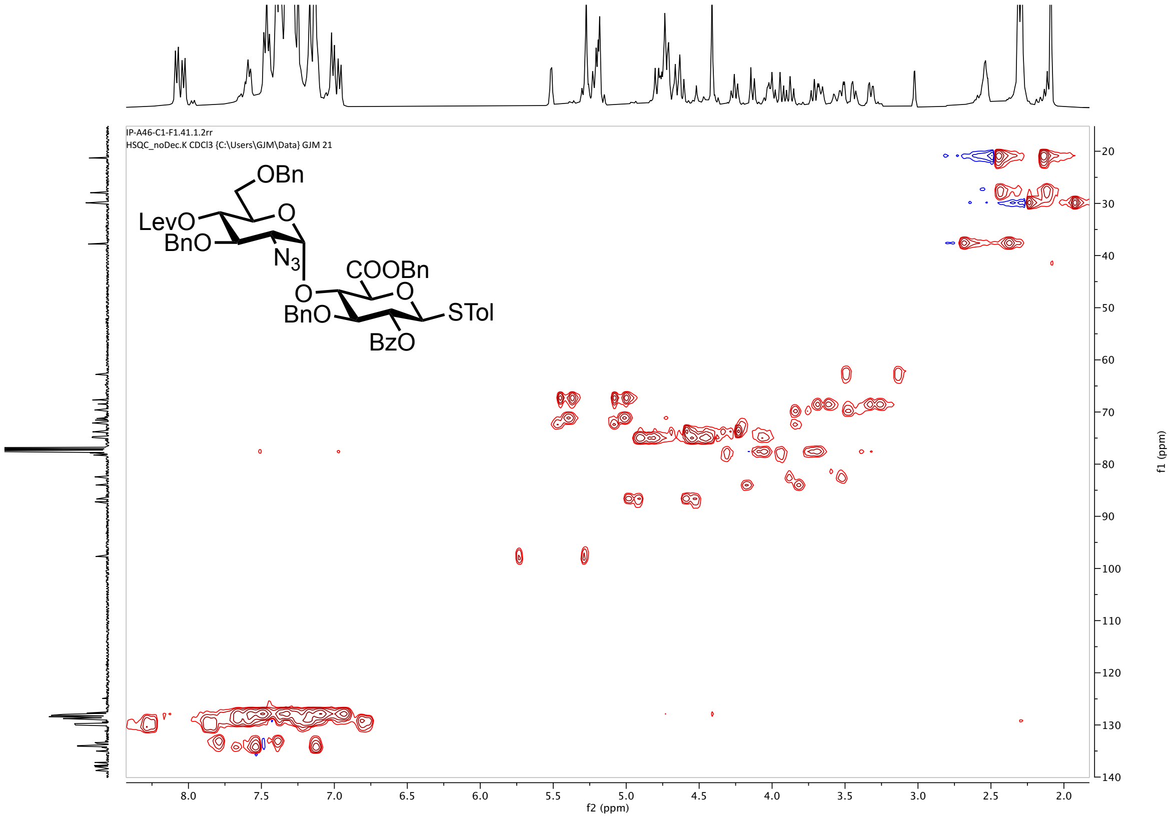

HMBC NMR (400 MHz x 101 MHz, Chloroform-*d*) inseparable mixture of 8a:4a = 61:39

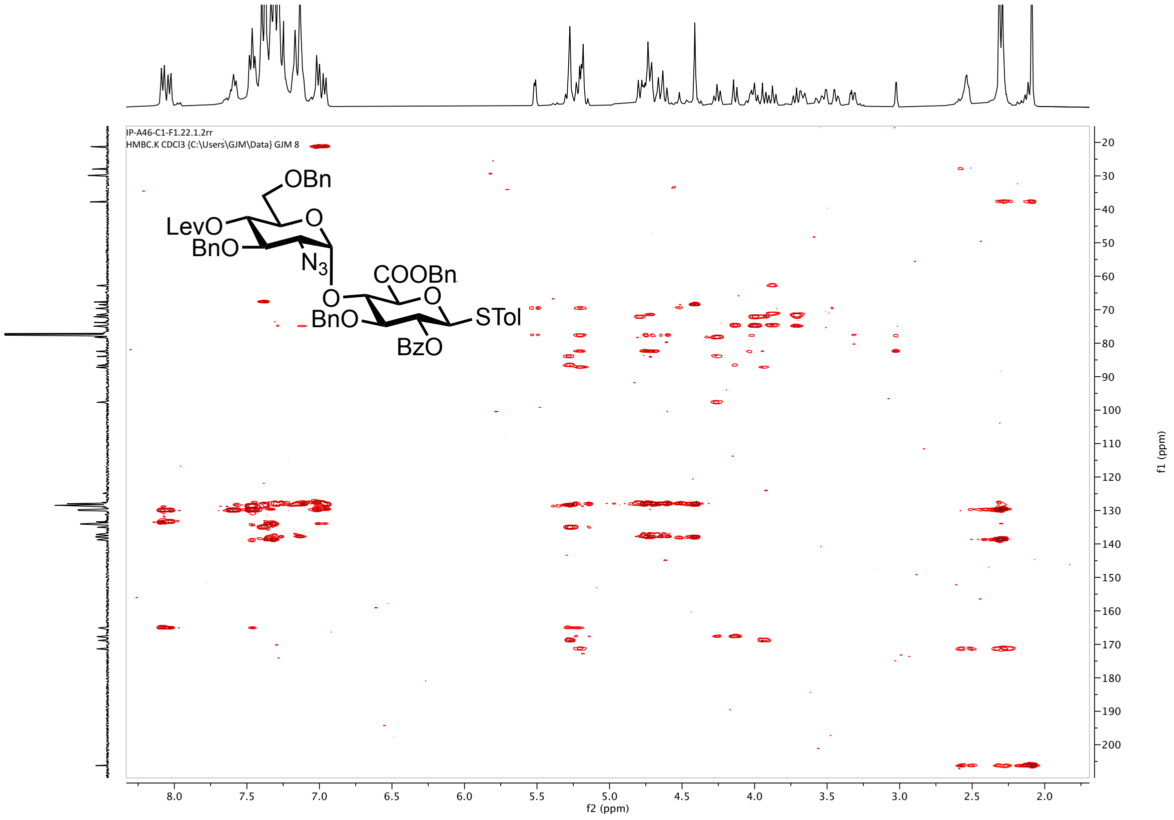

Compound 8b

^{1}H NMR (400 MHz, Chloroform-*d*) inseparable mixture of 8b:4b = 61:39

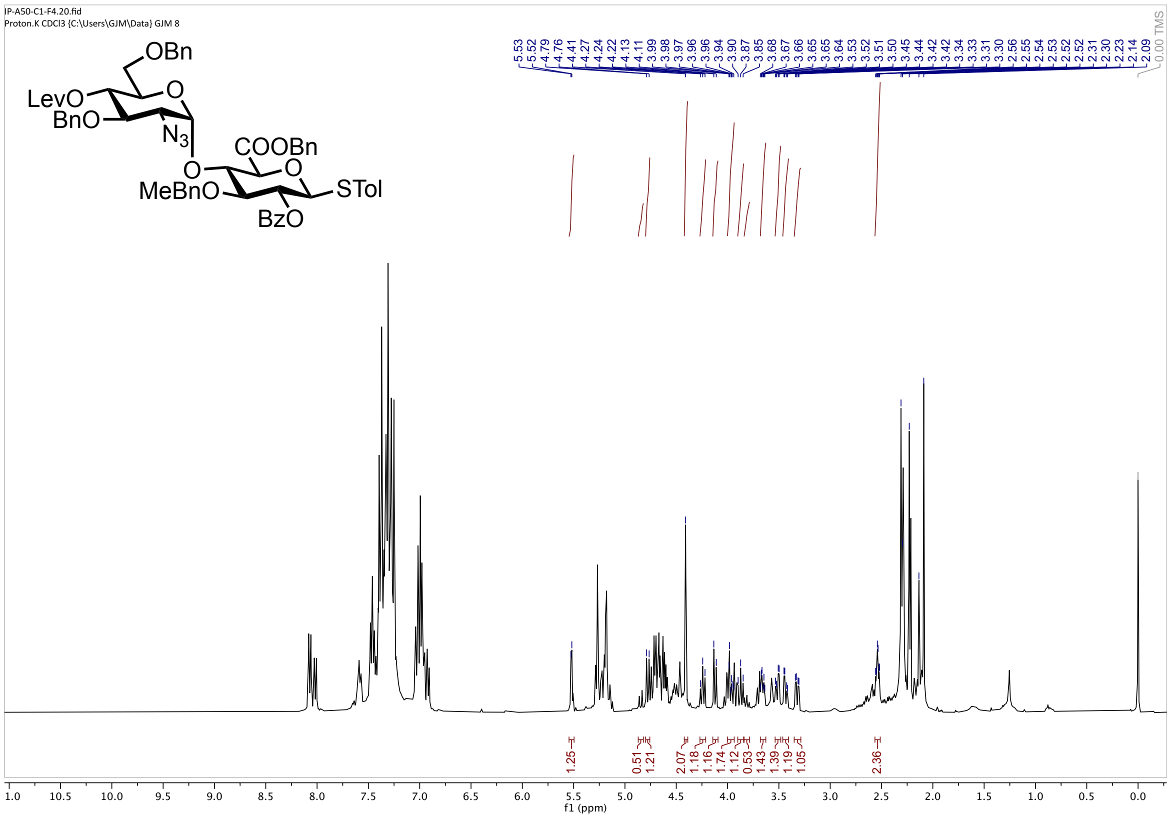

$^{13}C\{^{1}H\}$ NMR (101 MHz, Chloroform-*d*) inseparable mixture of 8b:4b = 61:39

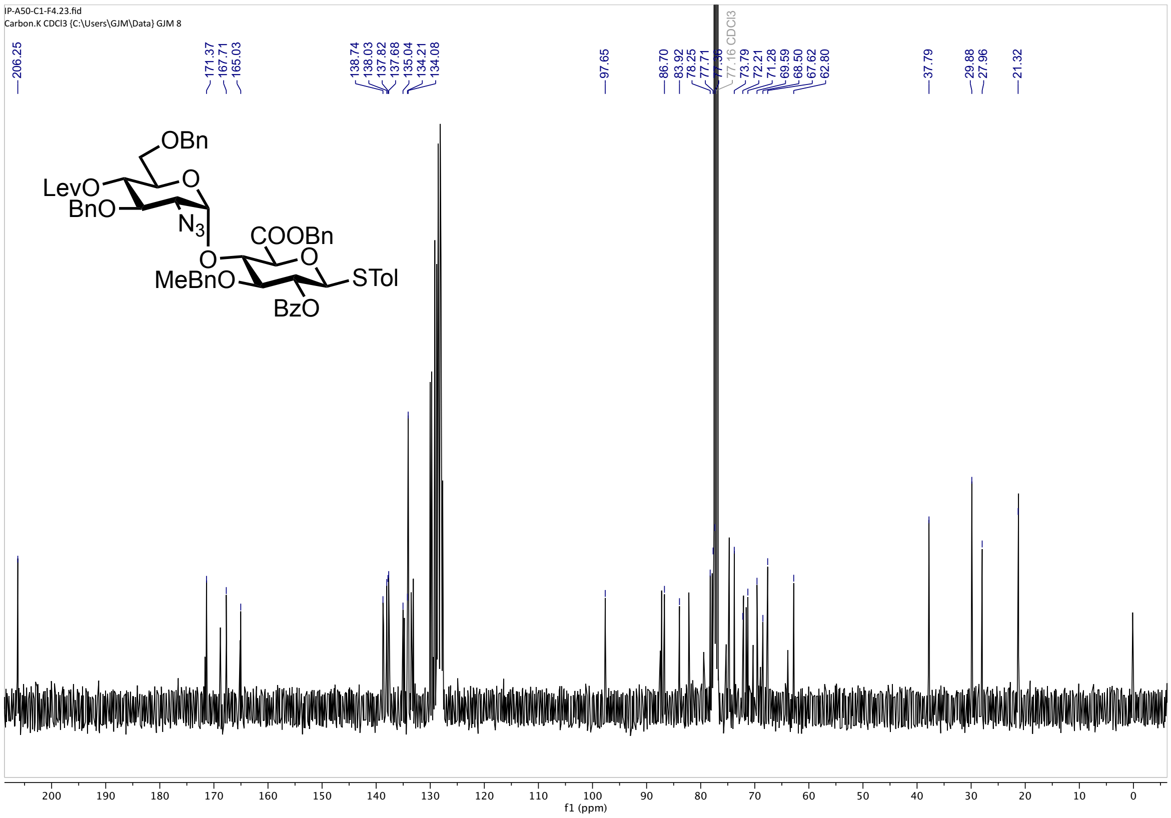

COSY NMR (400 MHz, Chloroform-*d*) inseparable mixture of 8b:4b = 61:39

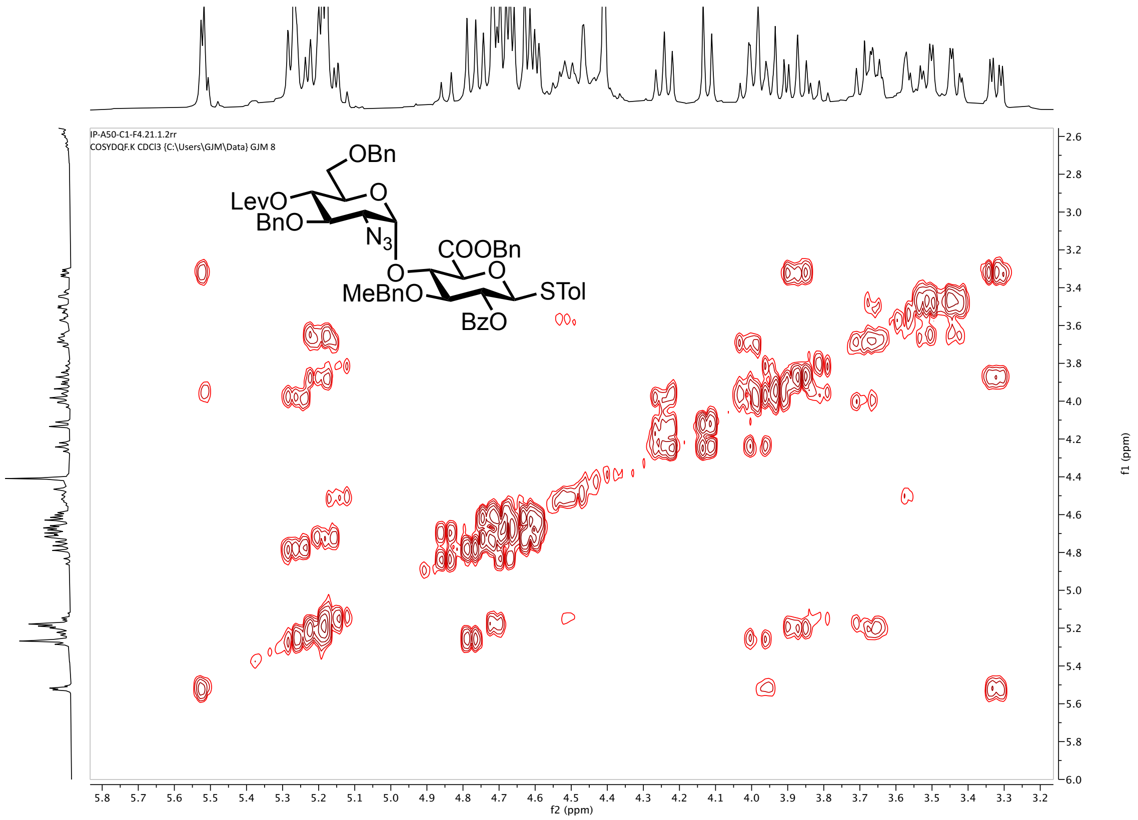

HSQC NMR (400 MHz x 101 MHz, Chloroform-*d*) inseparable mixture of 8b:4b = 61:39

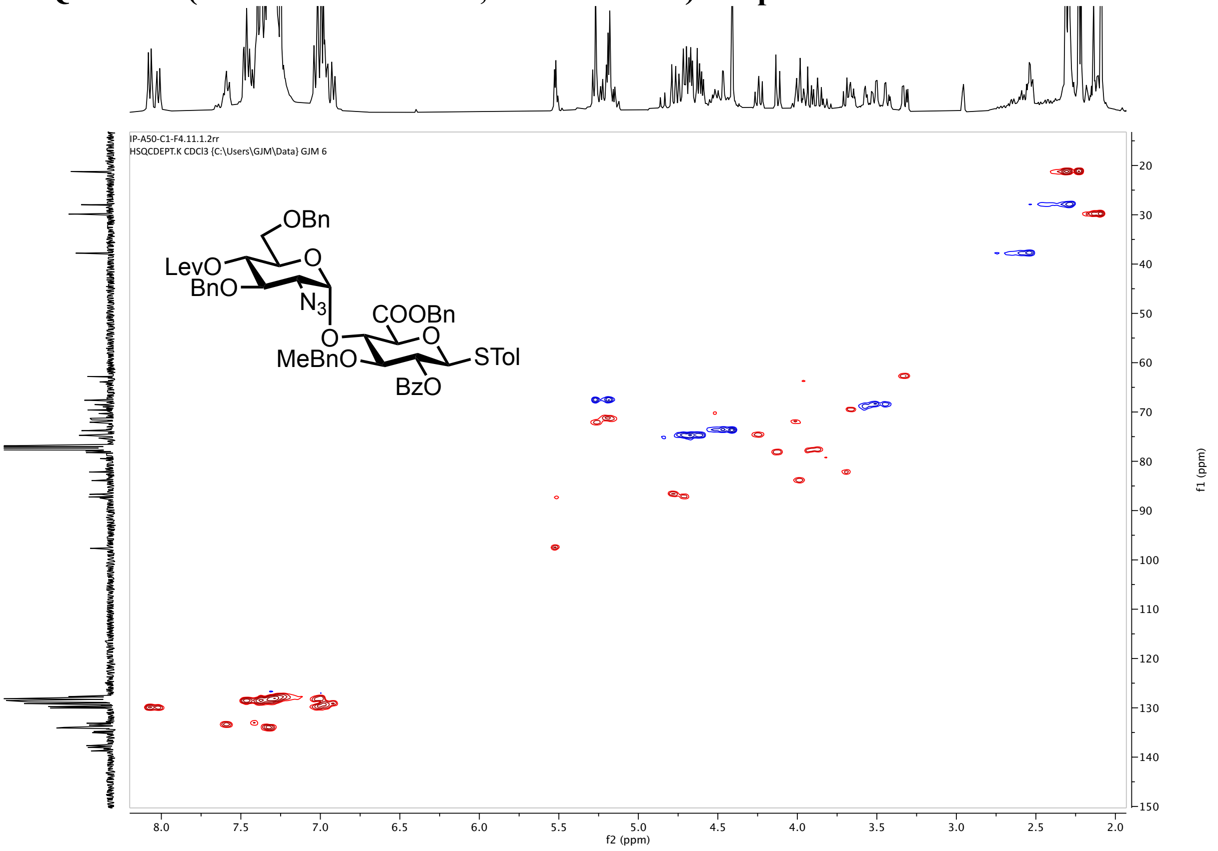

HMBC NMR (400 MHz x 101 MHz, Chloroform-*d*) inseparable mixture of 8b:4b = 61:39

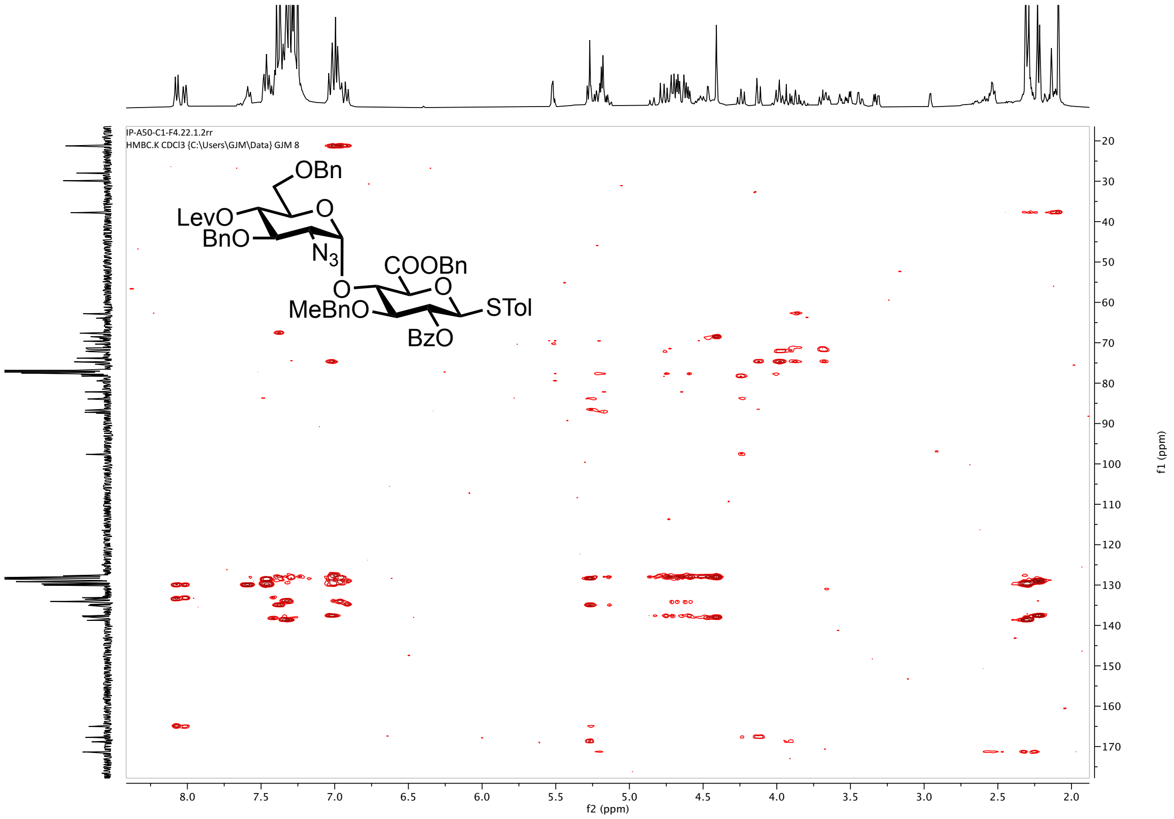

Compound 9a

^{1}H NMR (400 MHz, Chloroform-*d*) inseparable mixture of 9a:4a = 65:35

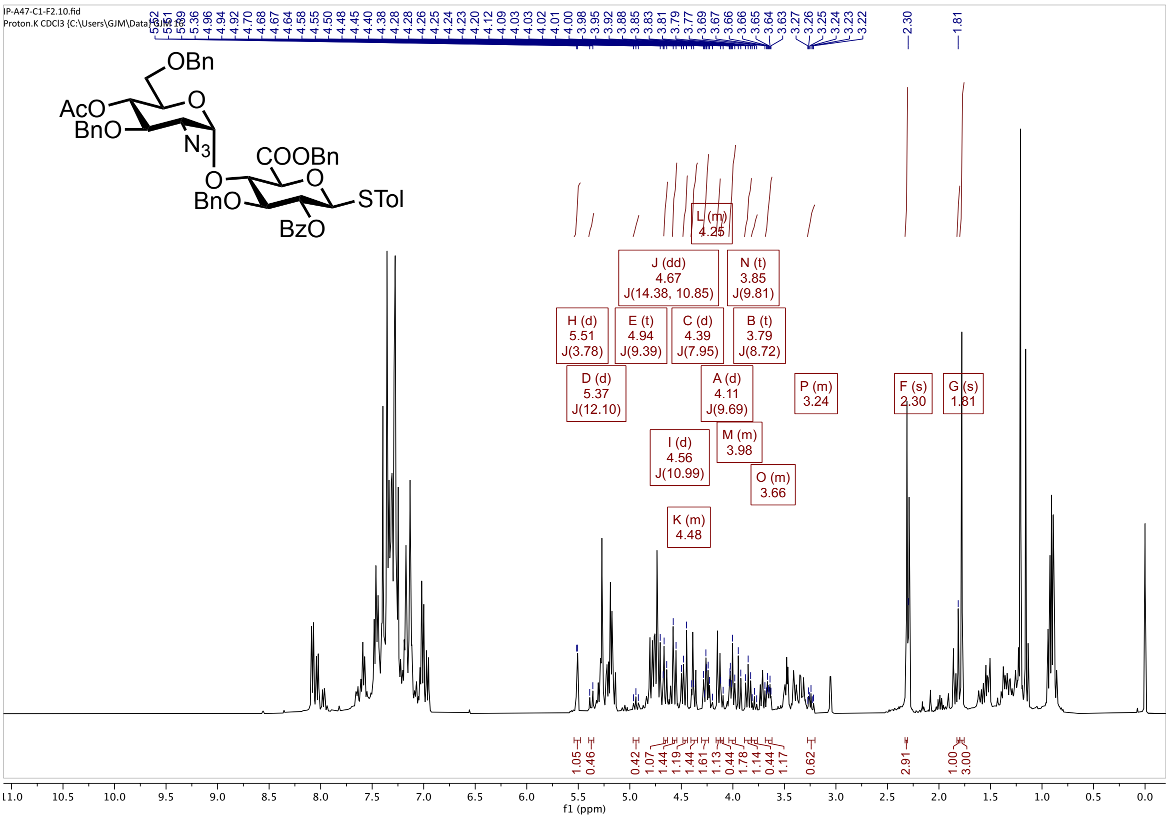

^{13}C{^{1}H} NMR (101 MHz, Chloroform-*d*) inseparable mixture of 9a:4a = 65:35

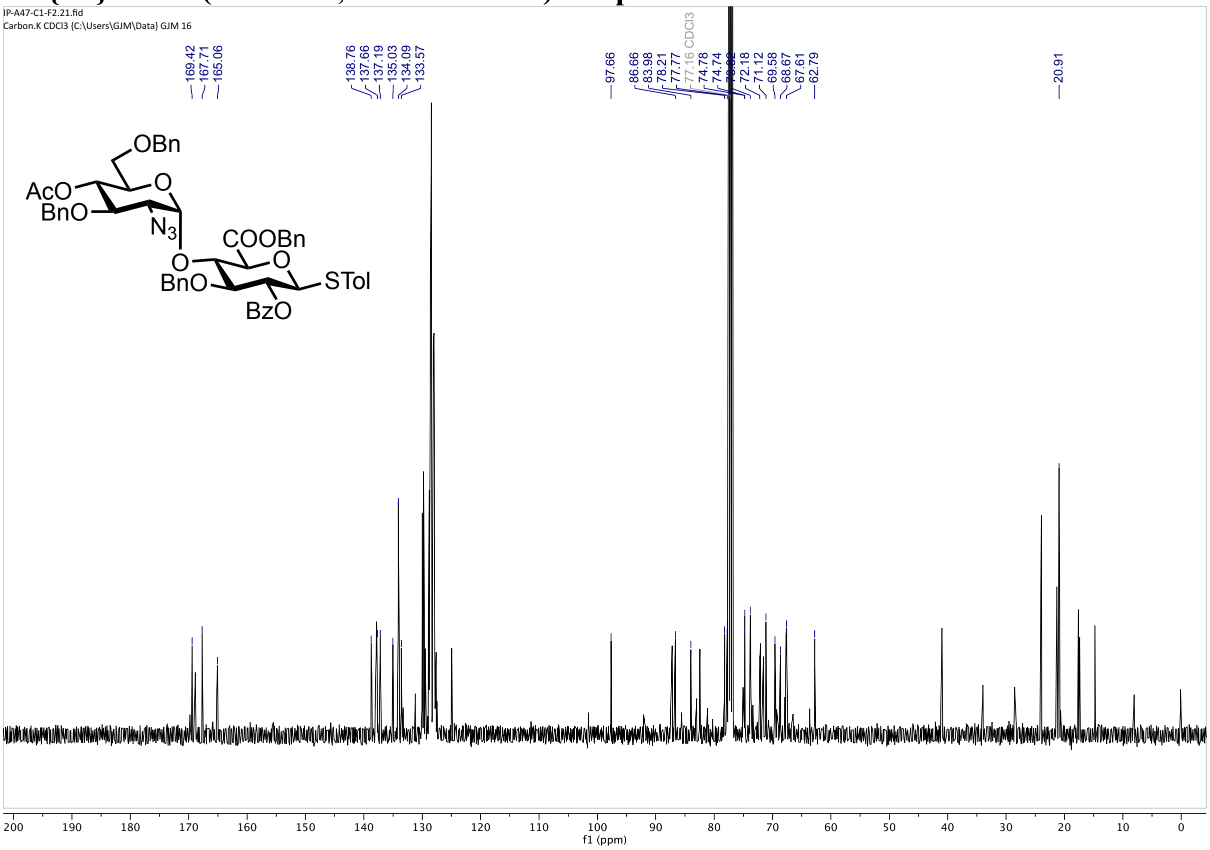

Compound 9b

^{1}H NMR (400 MHz, Chloroform-*d*) inseparable mixture of 9b:7b = 73:27

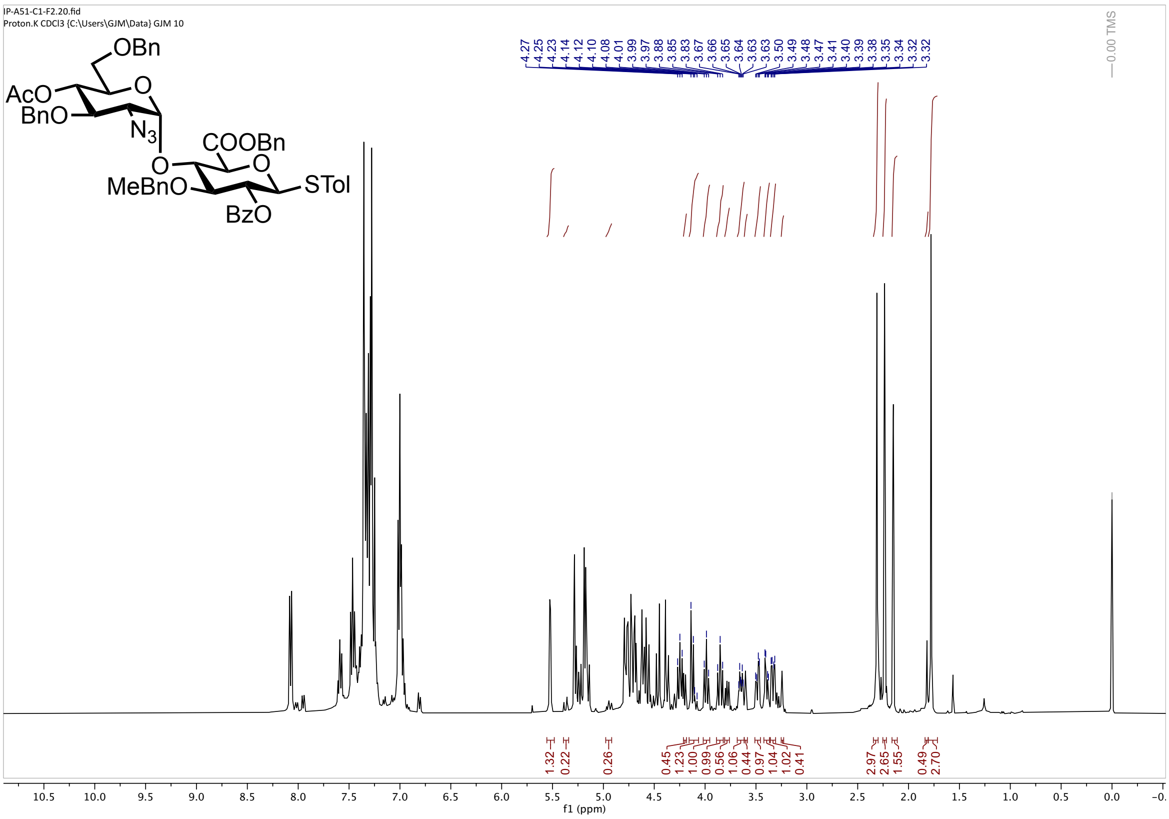

^{13}C{^{1}H} NMR (101 MHz, Chloroform-*d*) inseparable mixture of 9b:7b = 73:27

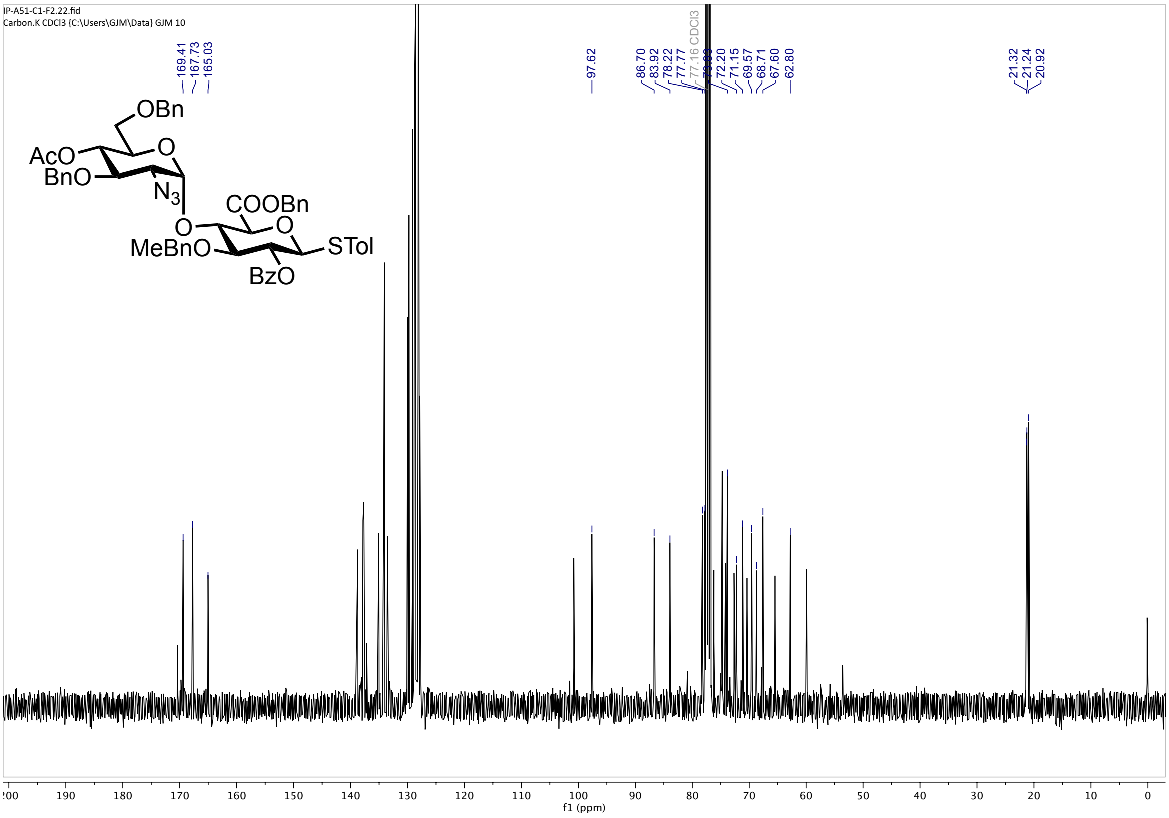

COSY NMR (400 MHz, Chloroform-*d*) inseparable mixture of 9b:7b = 73:27

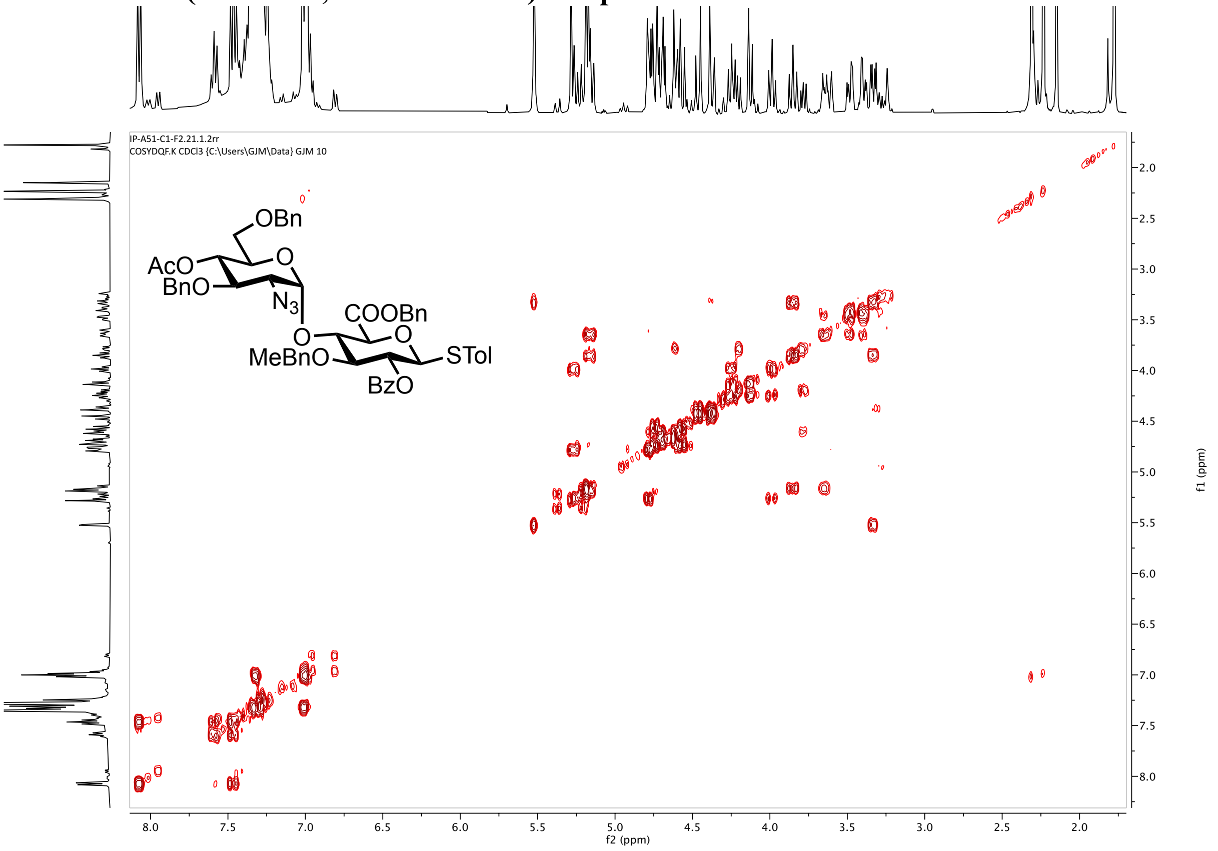

HSQC NMR (400 MHz x 101 MHz, Chloroform-*d*) inseparable mixture of 9b:7b = 73:27

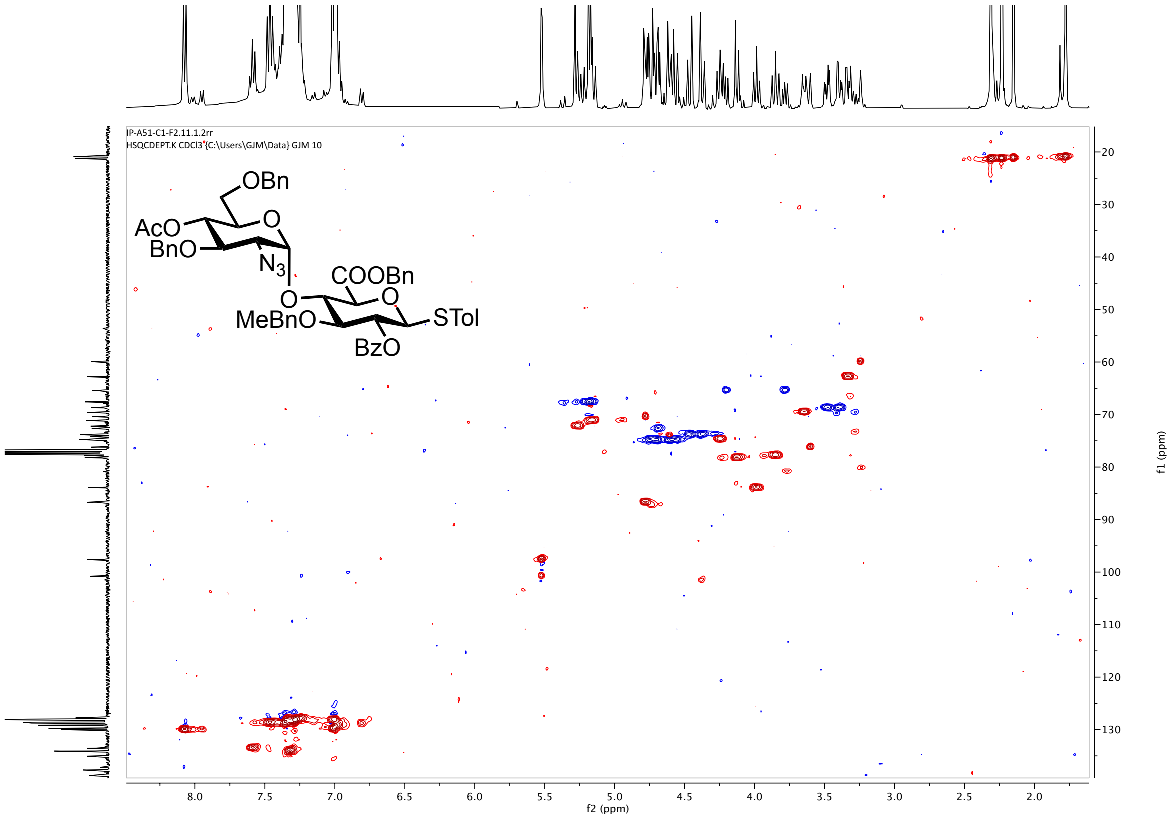

Coupled-HSQC NMR (400 MHz x 101 MHz, Chloroform-*d*) inseparable mixture of 9b:7b = 73:27

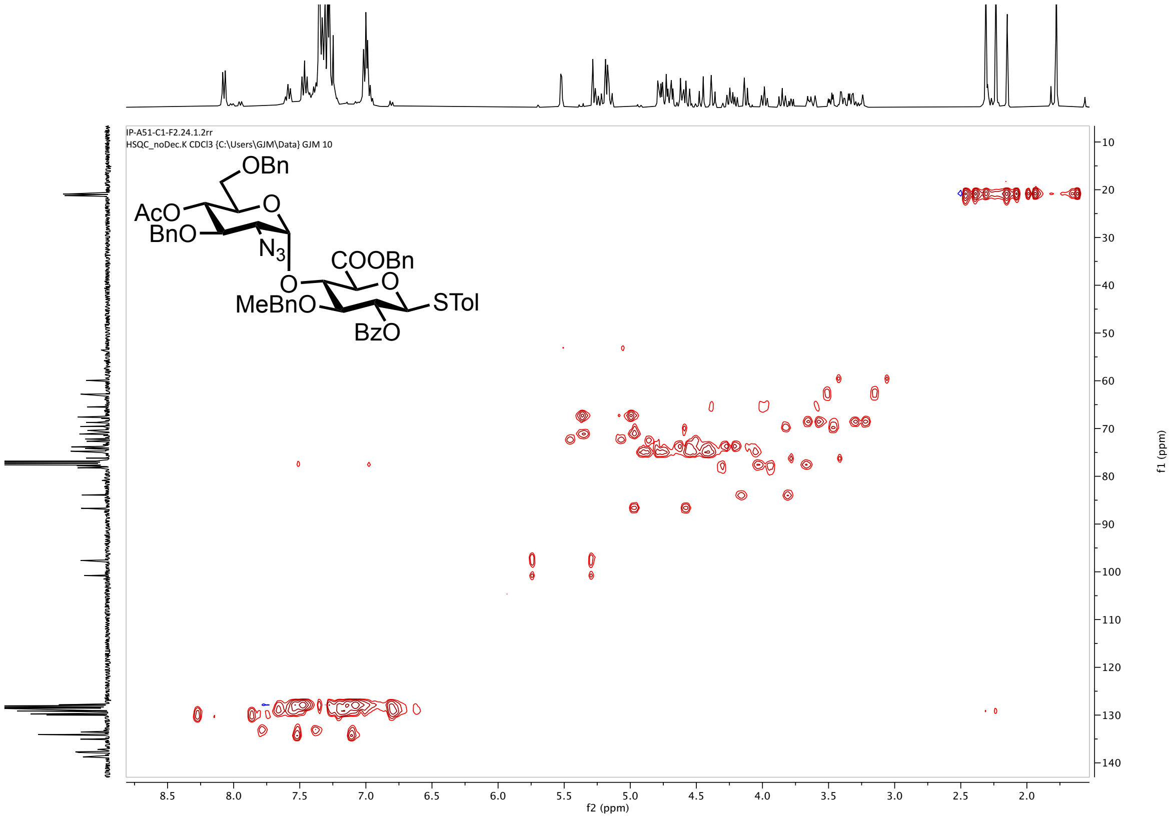

HMBC NMR (400 MHz x 101 MHz, Chloroform-*d*) inseparable mixture of 9b:7b = 73:27

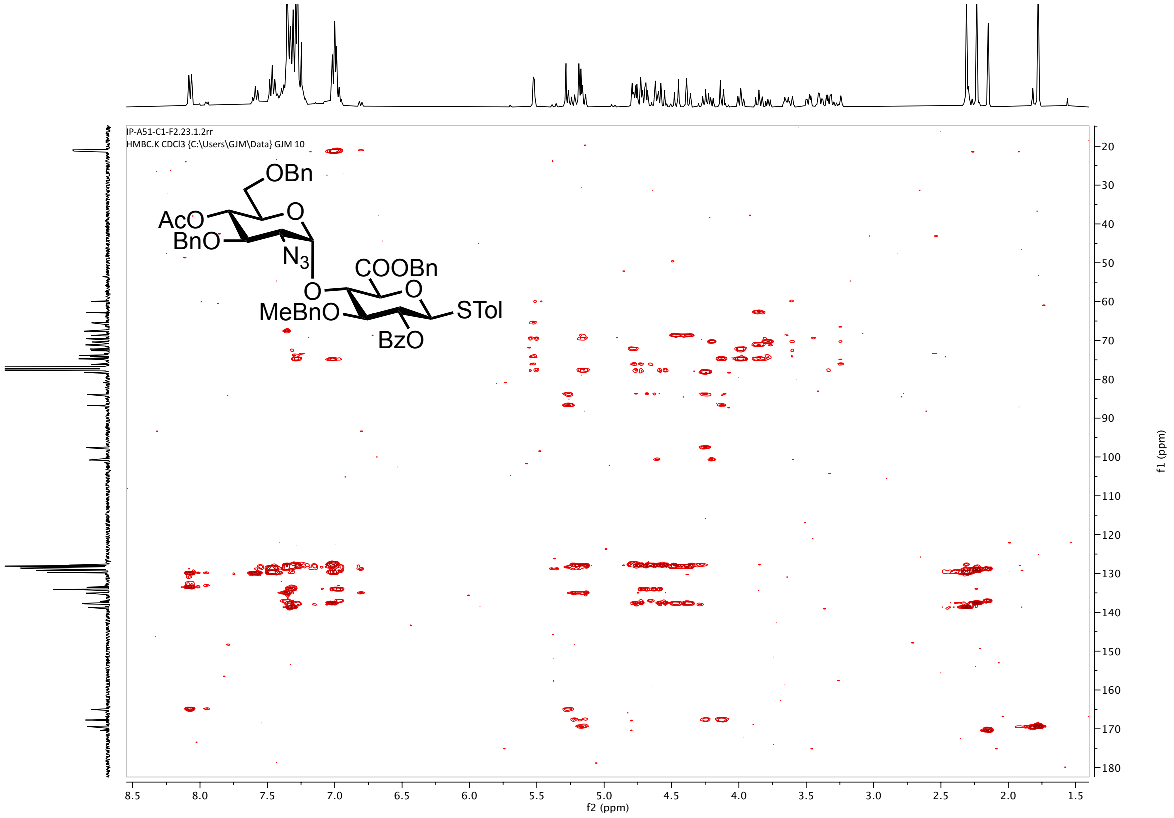

Compound 10a

^{1}H NMR (400 MHz, Chloroform-*d*) 10a

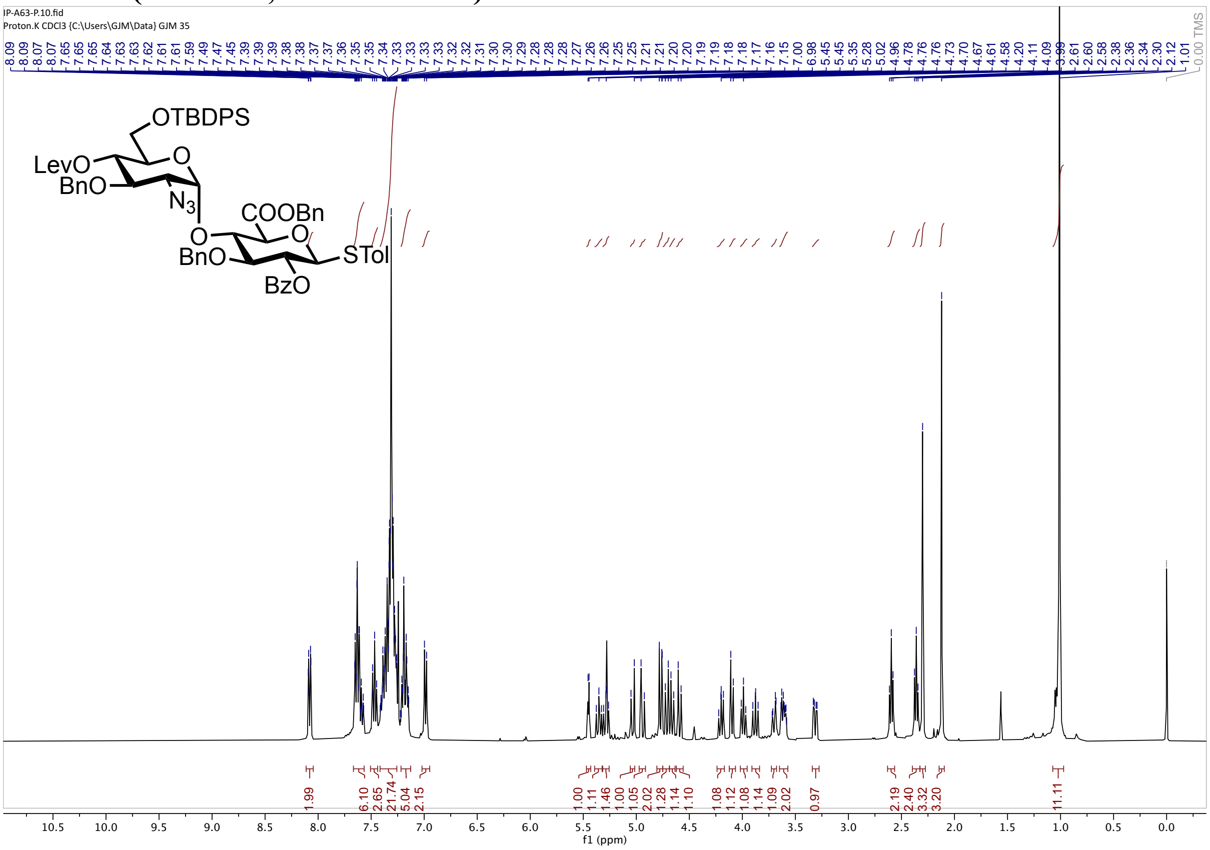

^{13}C{^{1}H} NMR (101 MHz, Chloroform-*d*) 10a

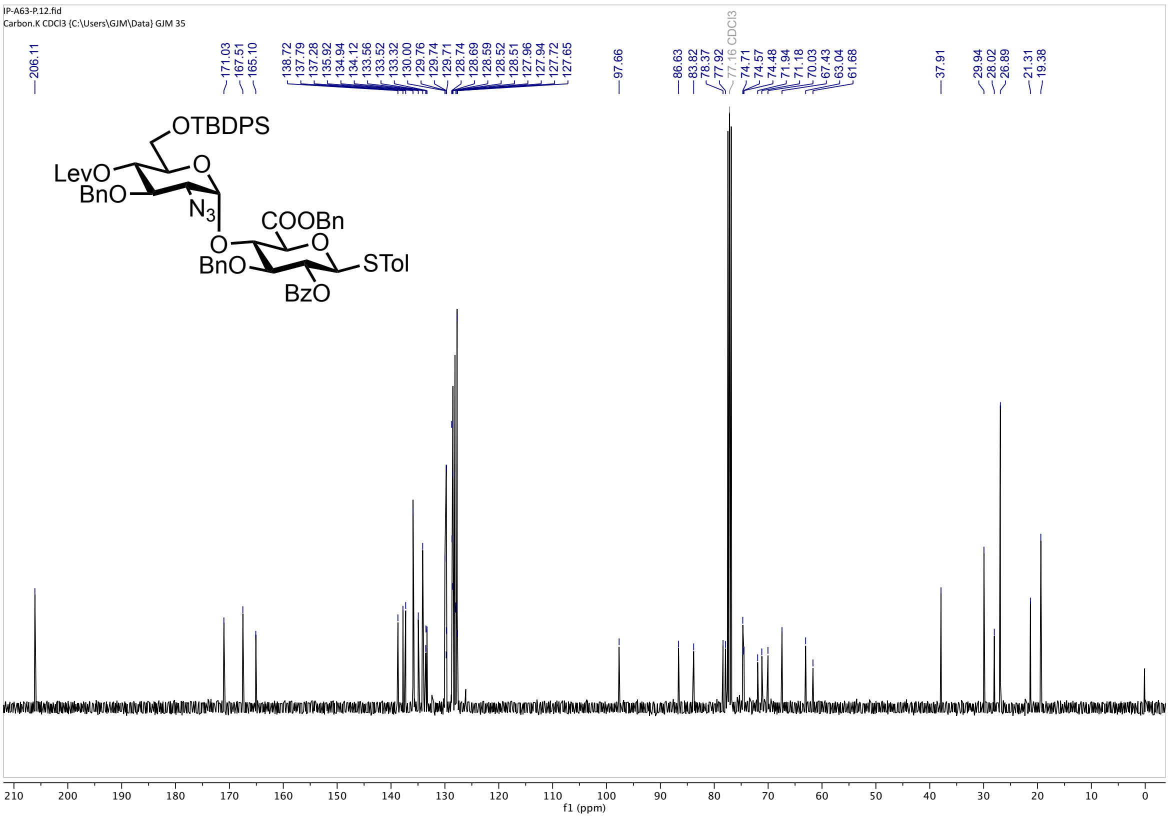

COSY NMR (400 MHz, Chloroform-*d*) 10a

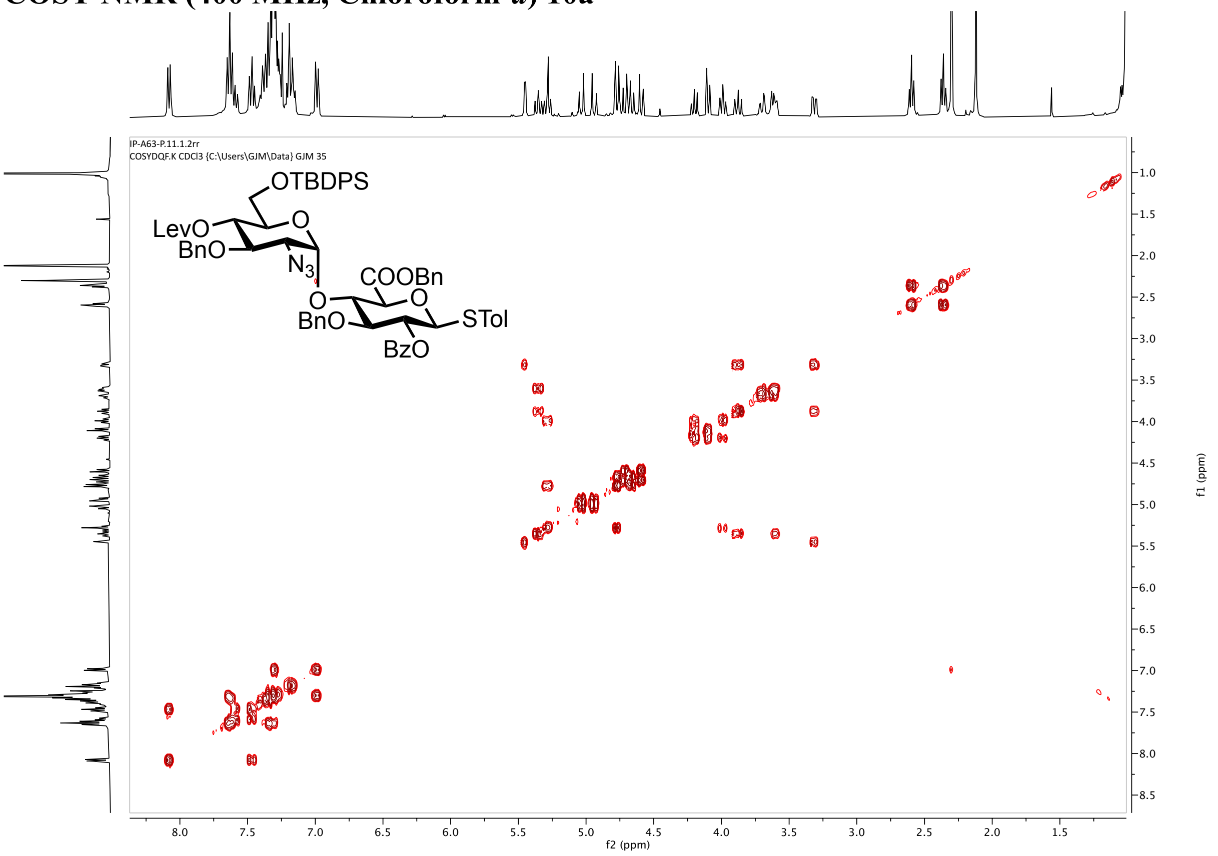

HSQC NMR (400 MHz x 101 MHz, Chloroform-*d*) 10a

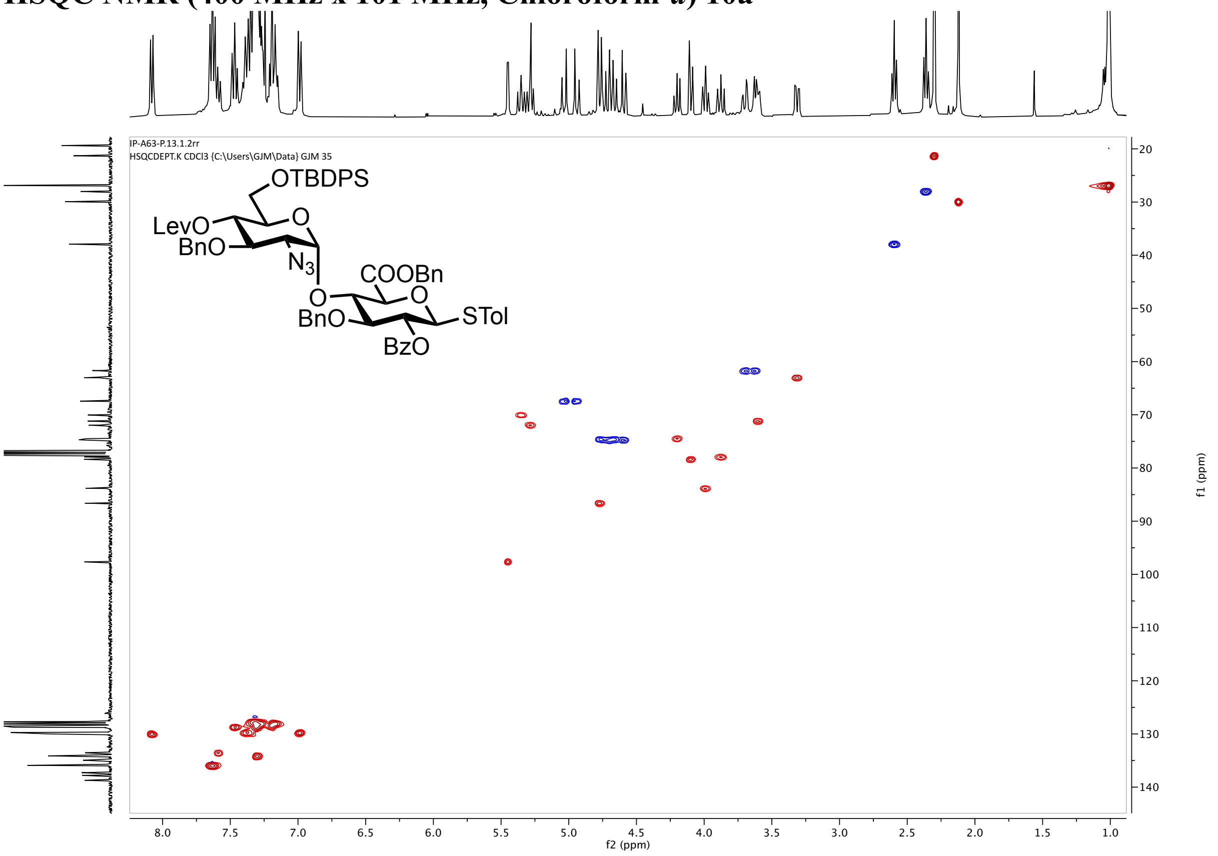

Coupled-HSQC NMR (400 MHz x 101 MHz, Chloroform-*d*) 10a

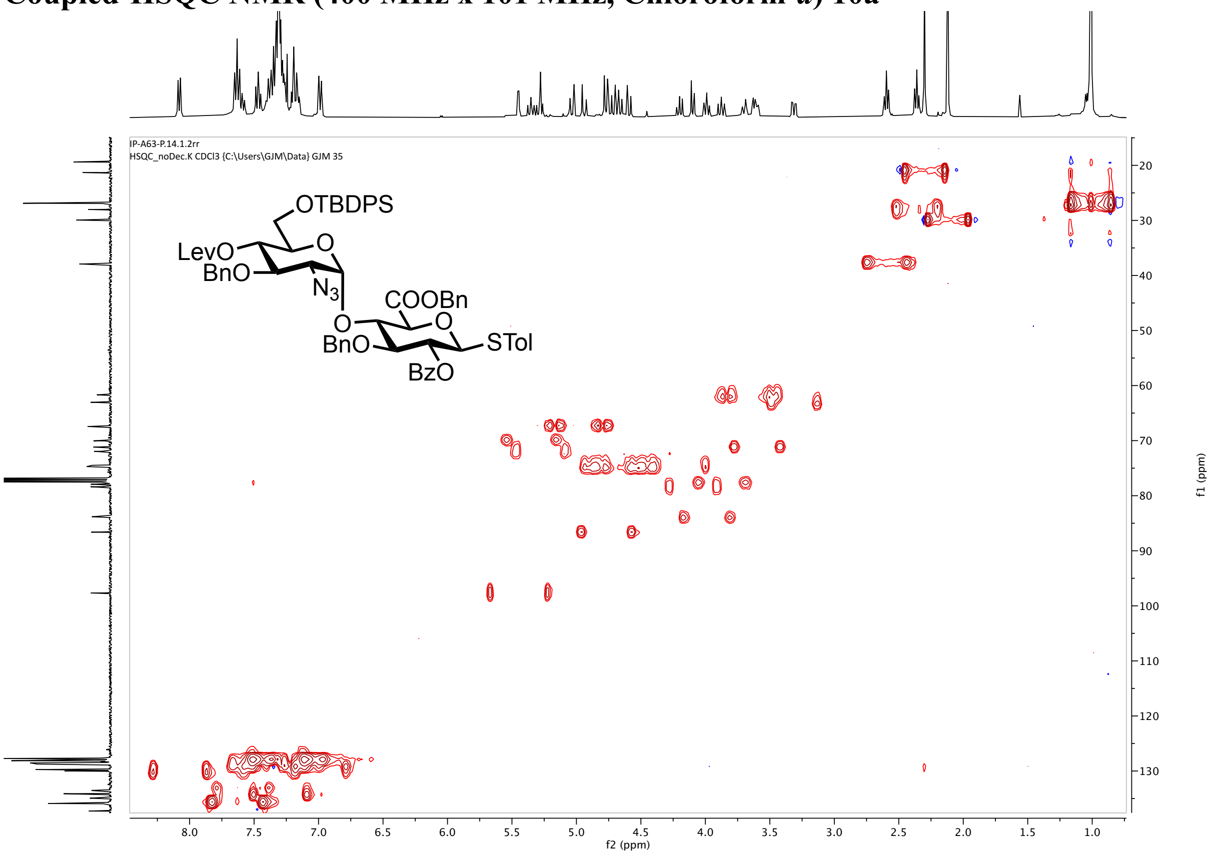

HMBC NMR (400 MHz x 101 MHz, Chloroform-*d*) 10a

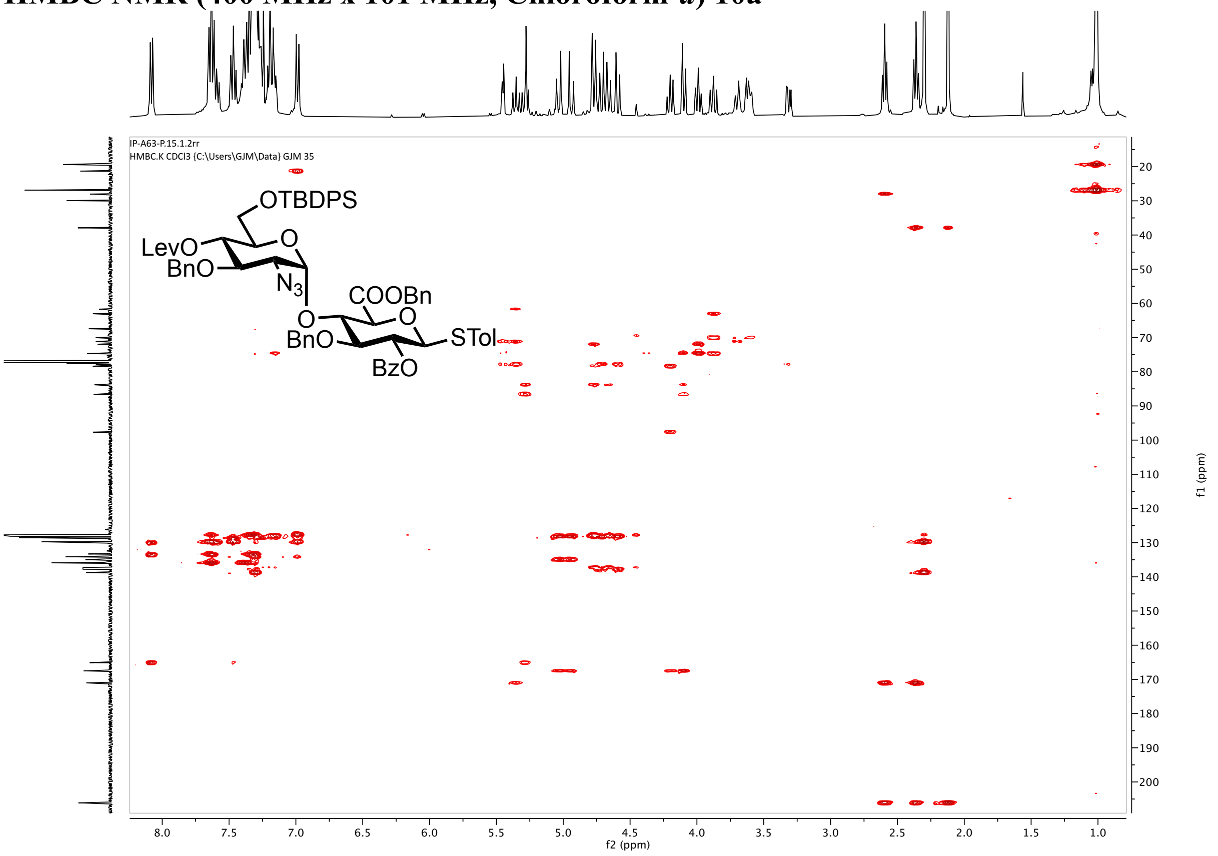

Compound 10b

^{1}H NMR (400 MHz, Chloroform-*d*) inseparable mixture of 10b:6b = 57:43

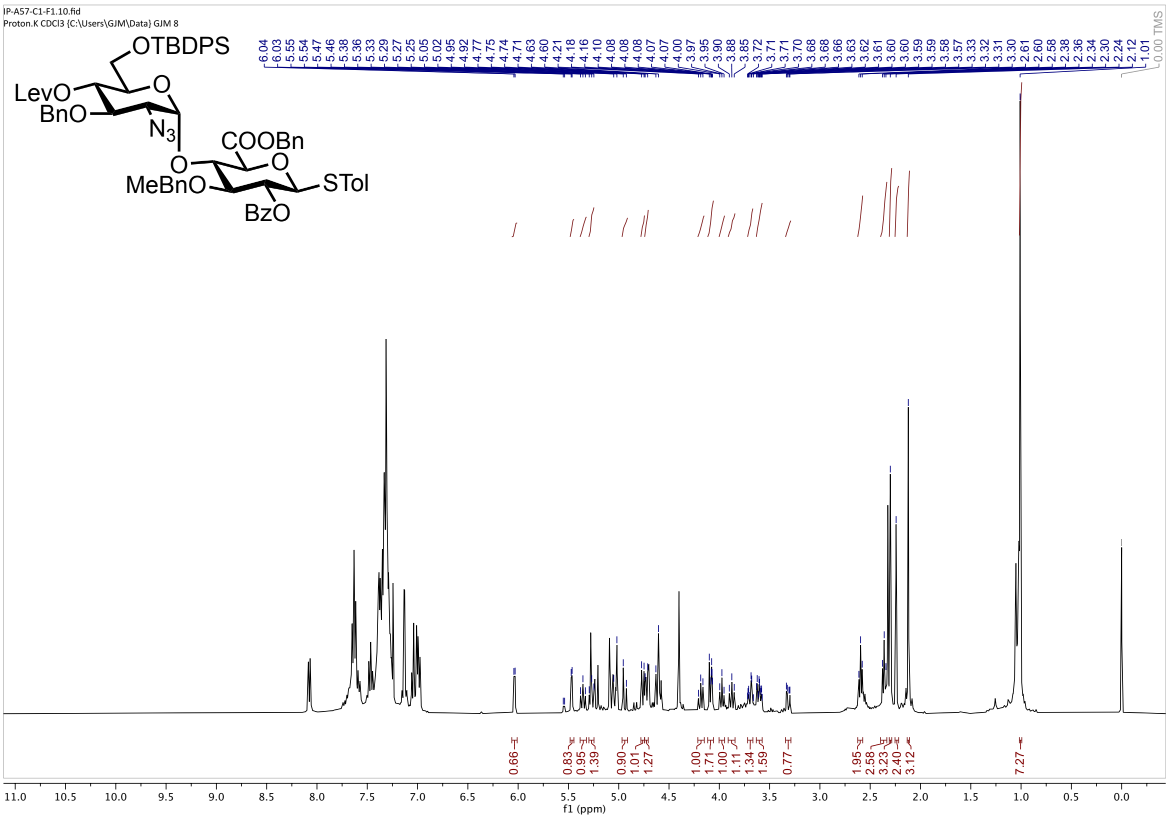

^{13}C{^{1}H} NMR (101 MHz, Chloroform-*d*) inseparable mixture of 10b:6b = 57:43

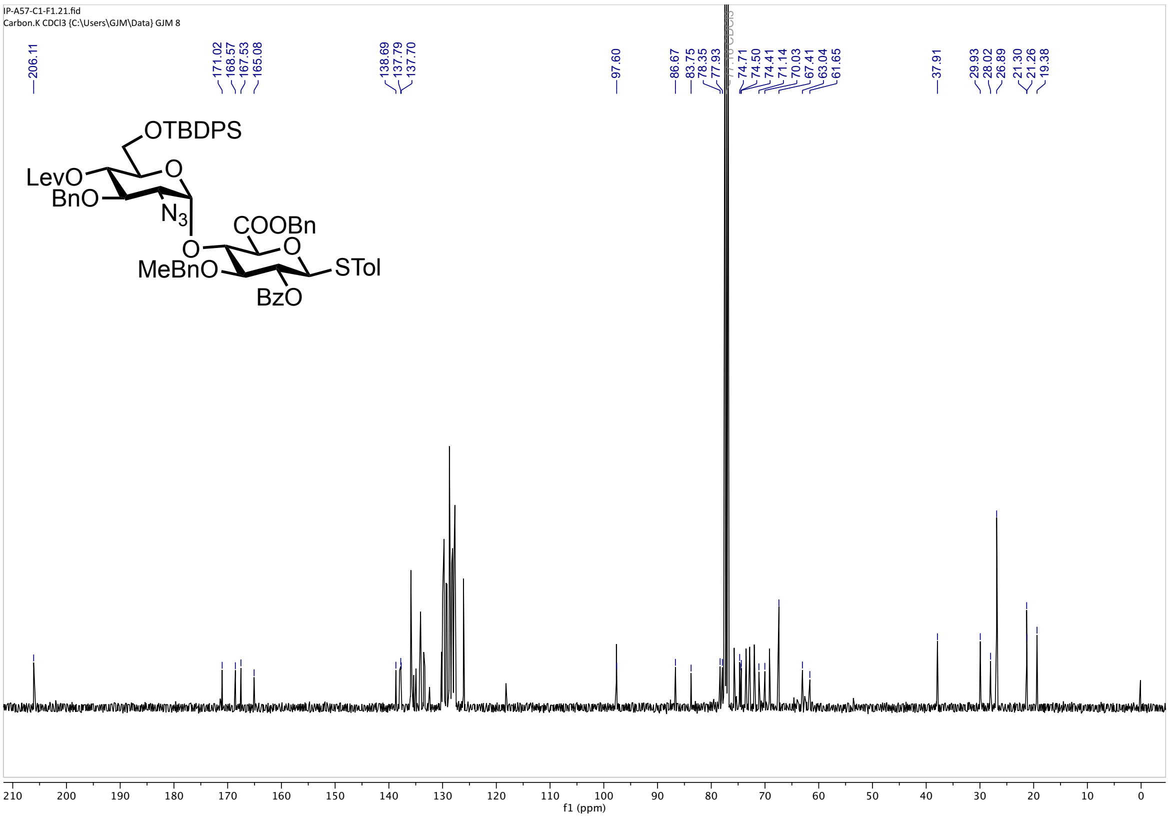

COSY NMR (400 MHz, Chloroform-*d*) inseparable mixture of 10b:6b = 57:43

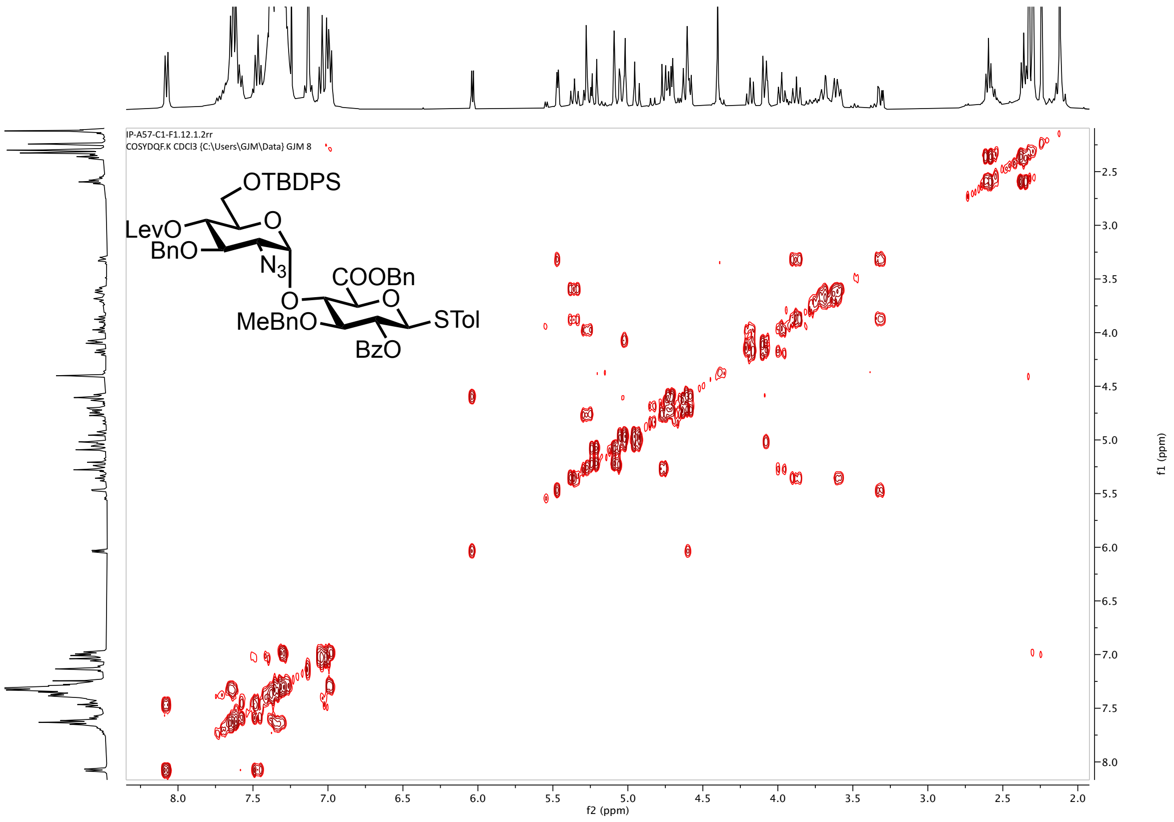

HSQC NMR (400 MHz x 101 MHz, Chloroform-*d*) inseparable mixture of 10b:6b = 57:43

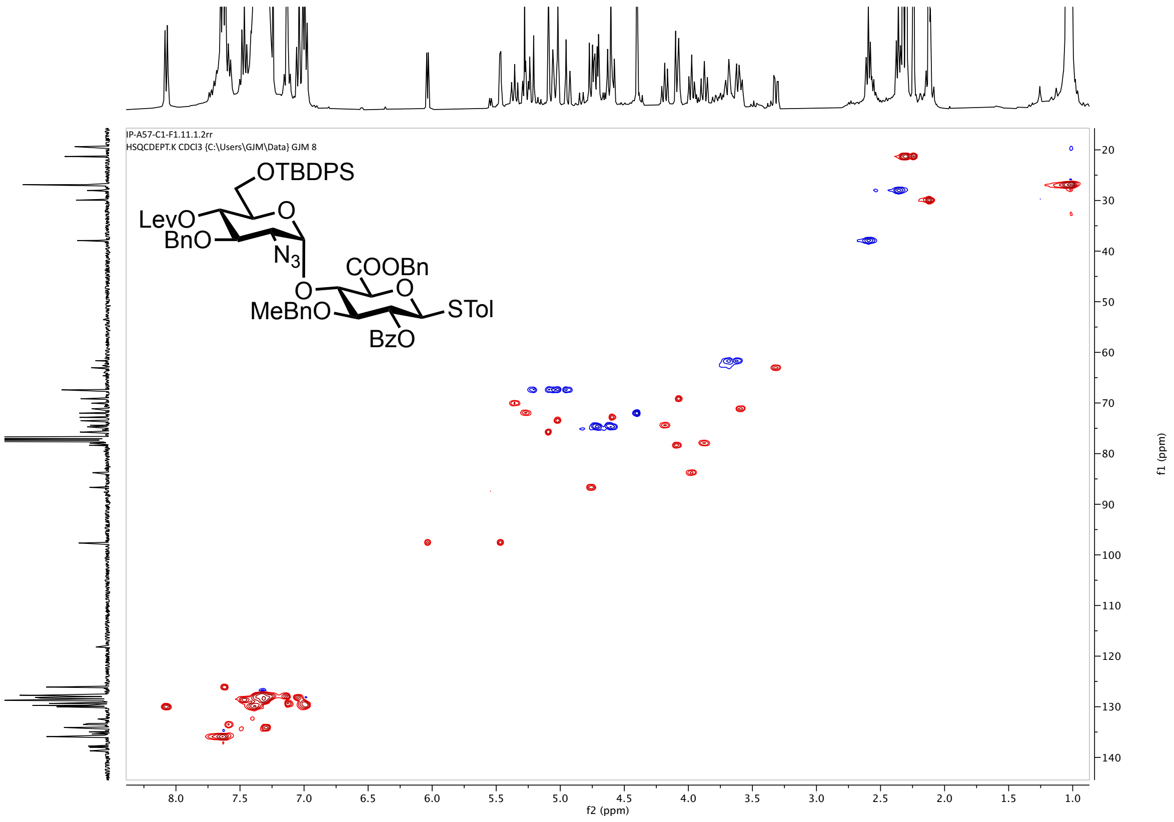

Coupled-HSQC NMR (400 MHz x 101 MHz, Chloroform-*d*) inseparable mixture of 10b:6b = 57:43

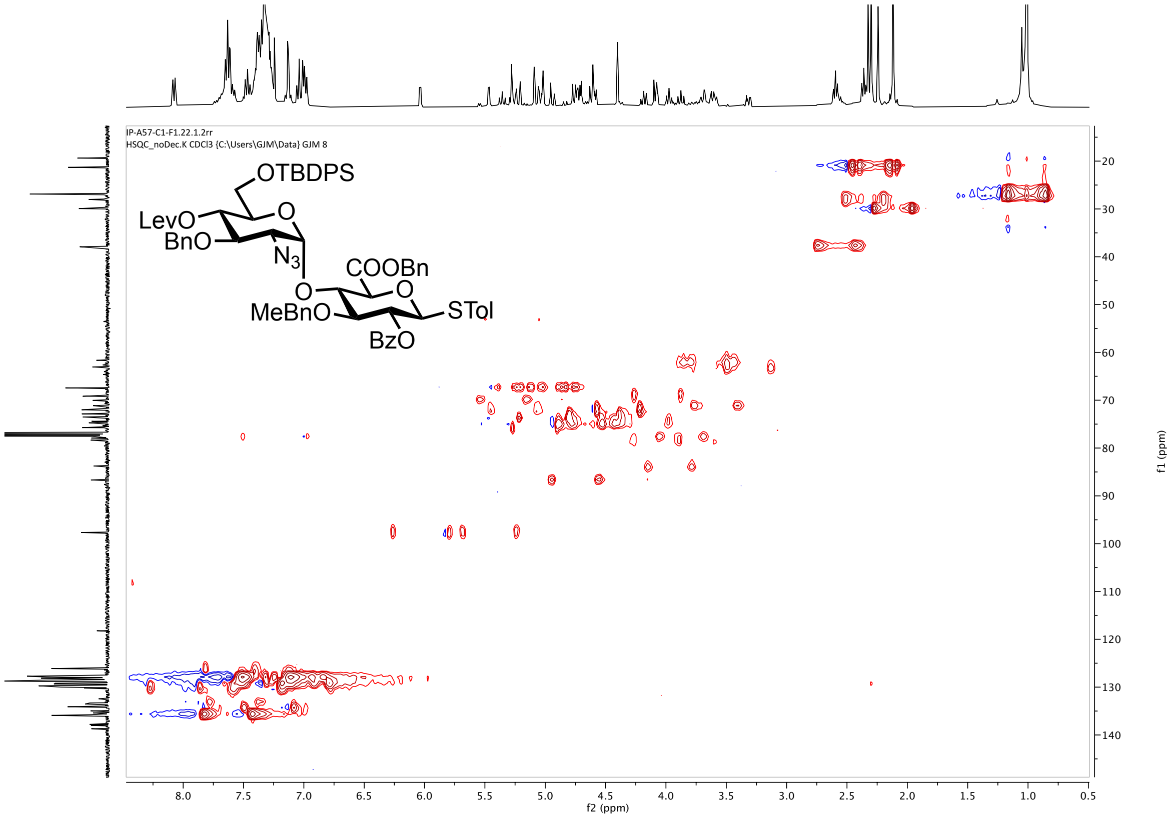

HMBC NMR (400 MHz x 101 MHz, Chloroform-*d*) inseparable mixture of 10b:6b = 57:43

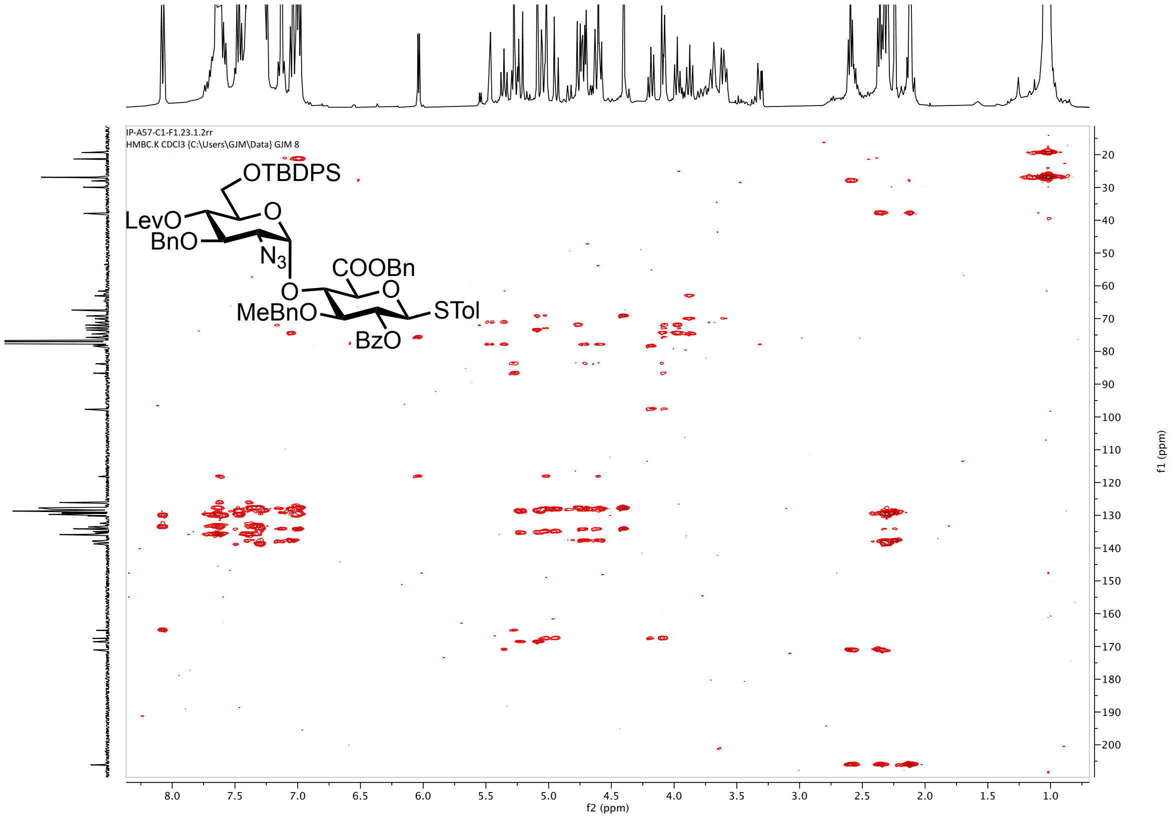

Compound 11a

^{1}H NMR (400 MHz, Chloroform-*d*) 11a

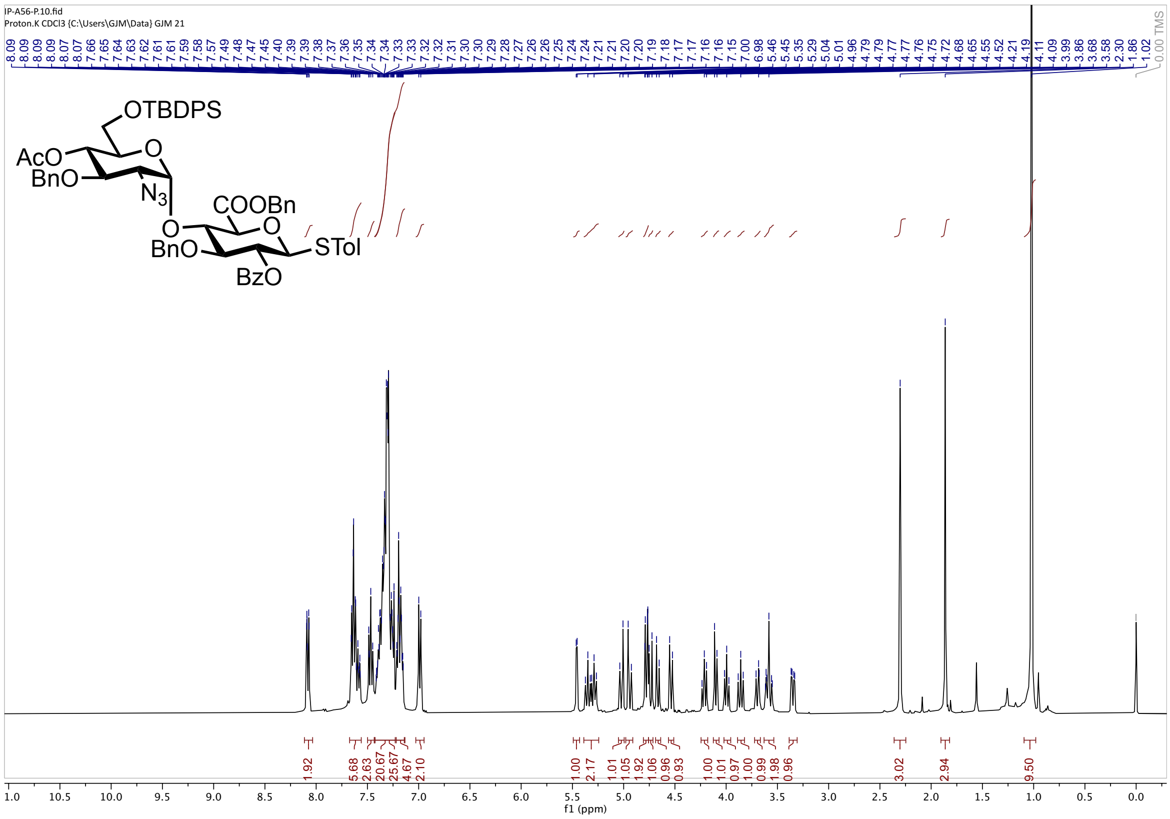

^{13}C{^{1}H} NMR (101 MHz, Chloroform-*d*) 11a

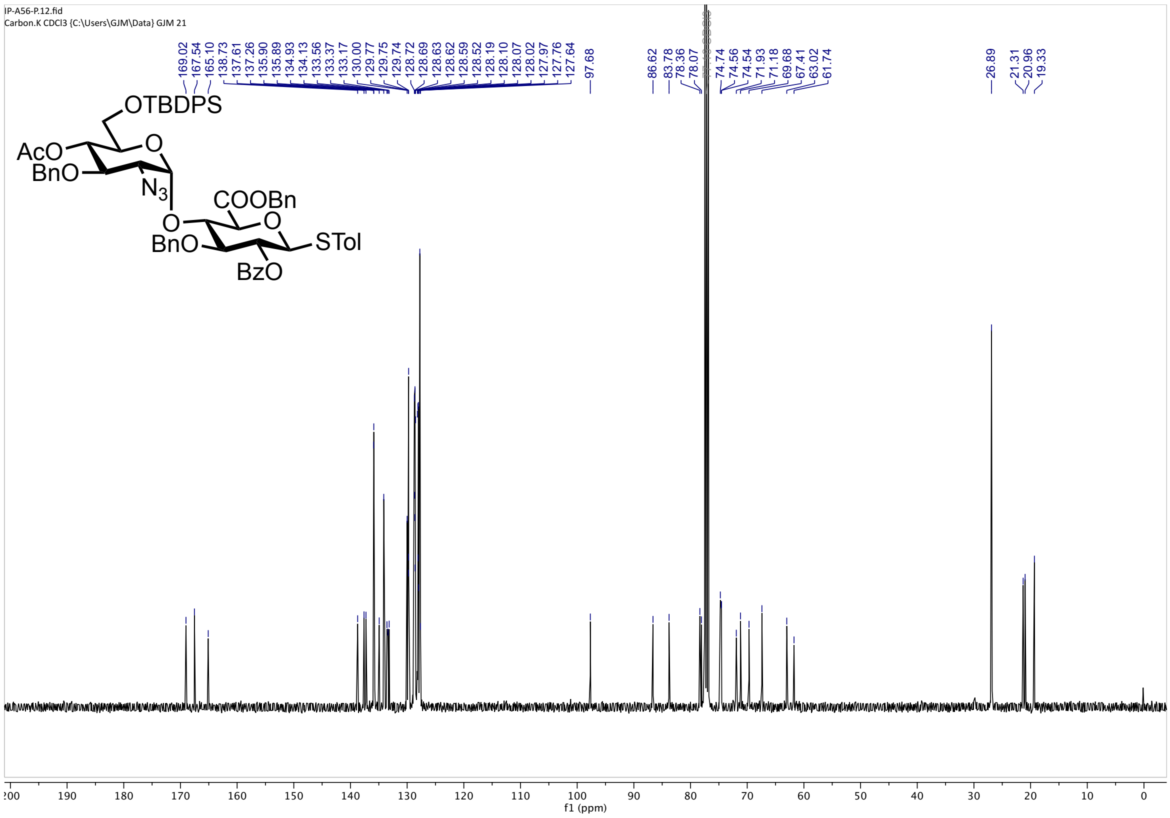

COSY NMR (400 MHz, Chloroform-*d*) 11a

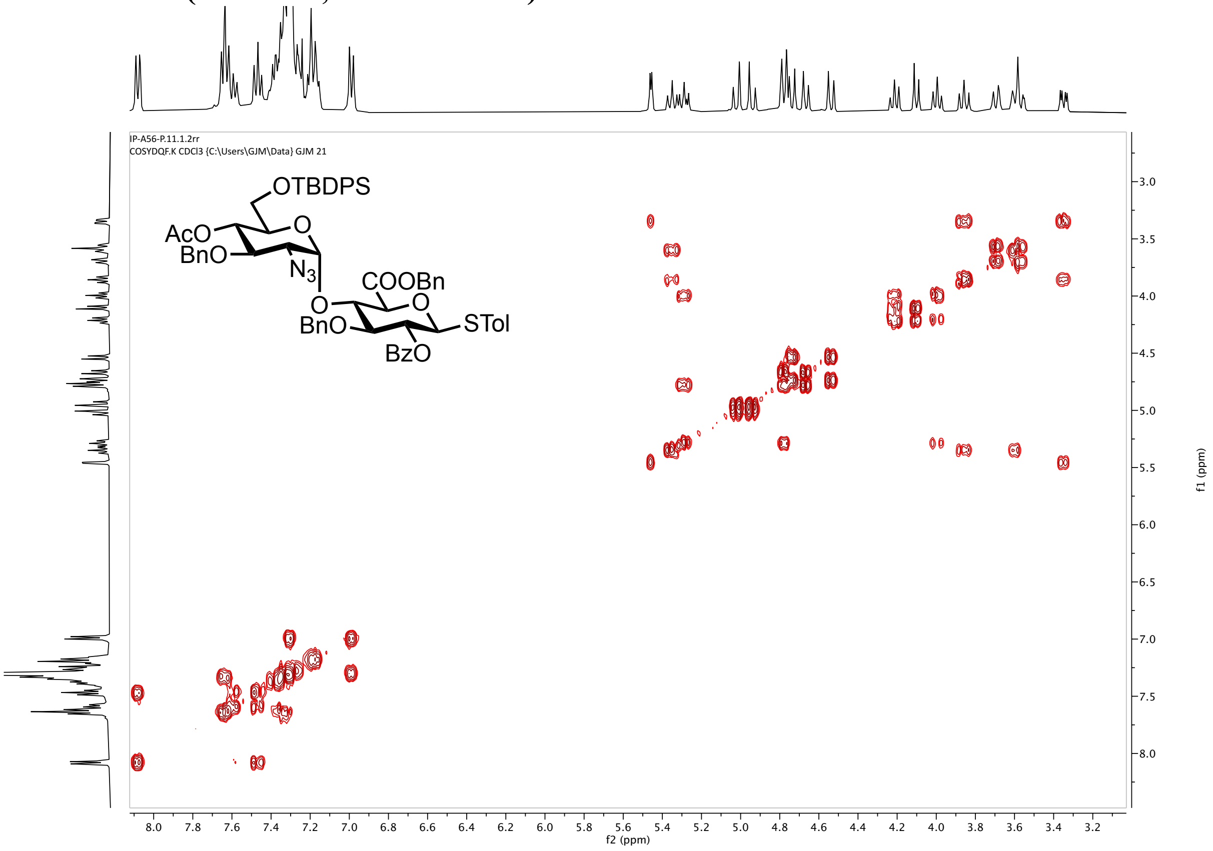

HSQC NMR (400 MHz x 101 MHz, Chloroform-*d*) 11a

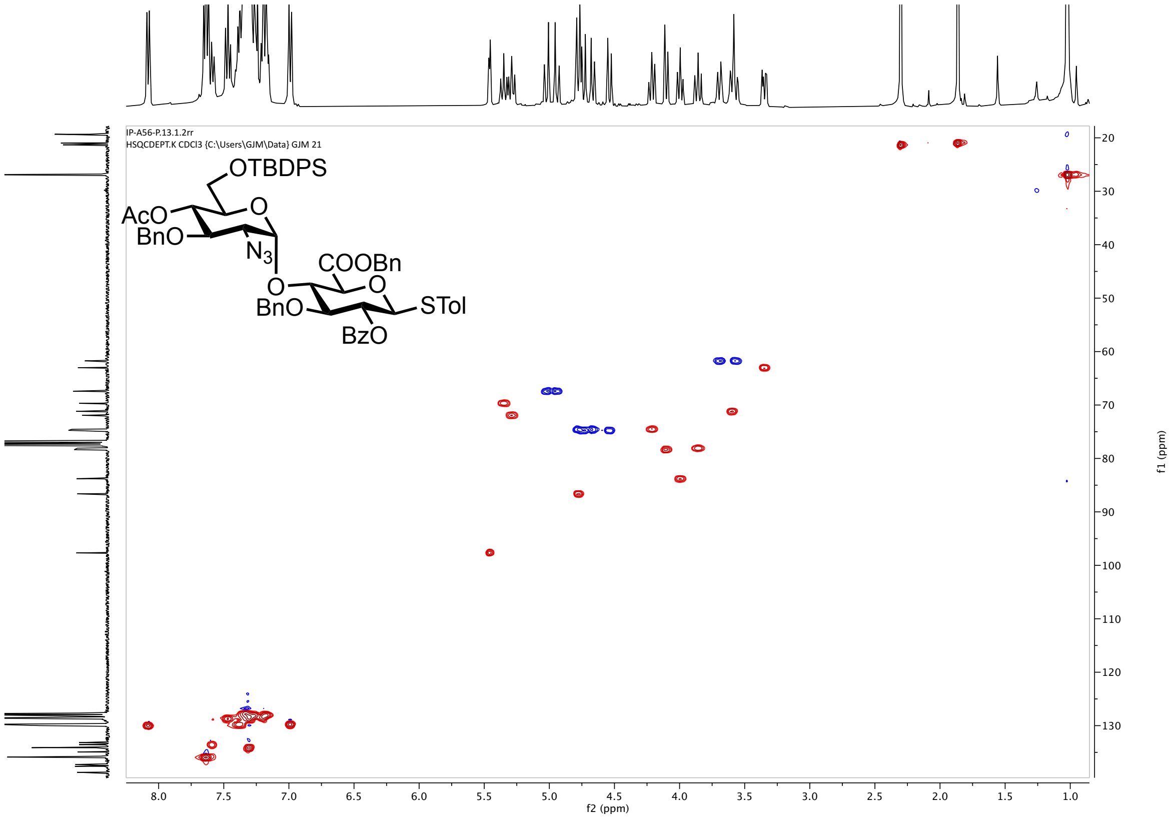

Coupled-HSQC NMR (400 MHz x 101 MHz, Chloroform-*d*) 11a

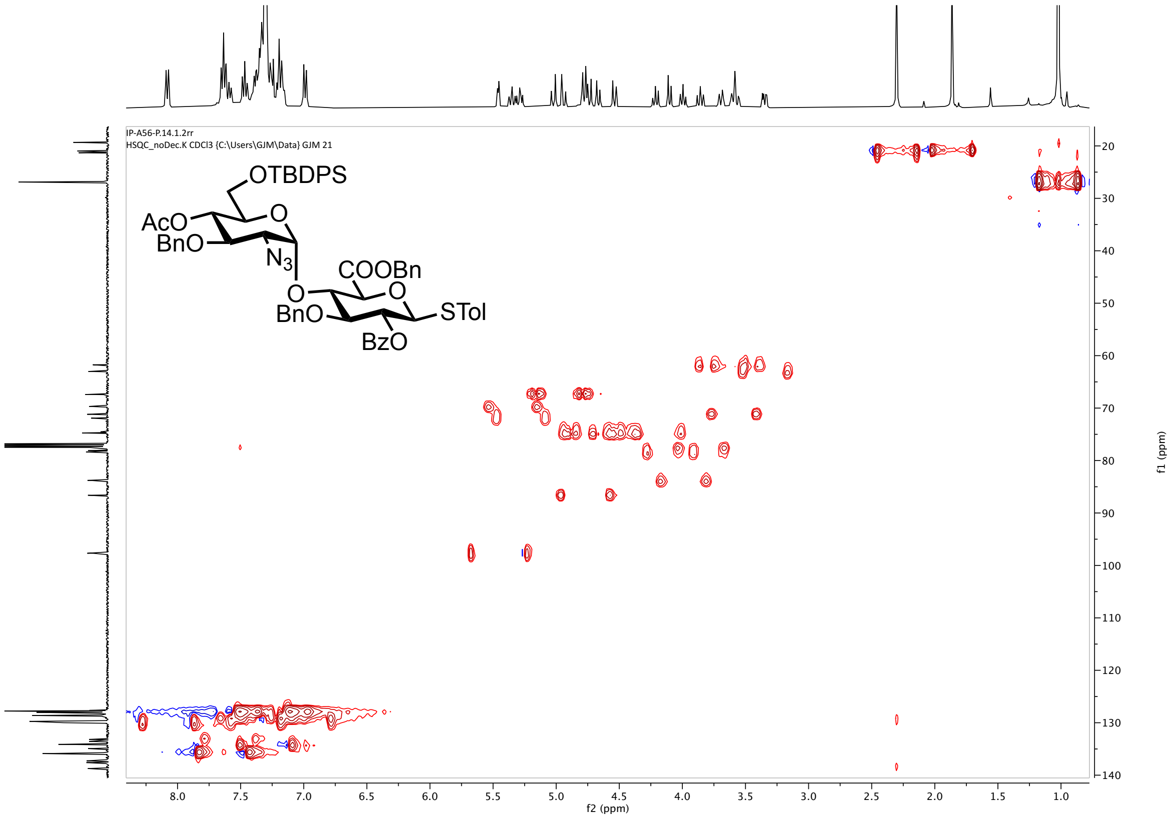

HMBC NMR (400 MHz x 101 MHz, Chloroform-*d*) 11a

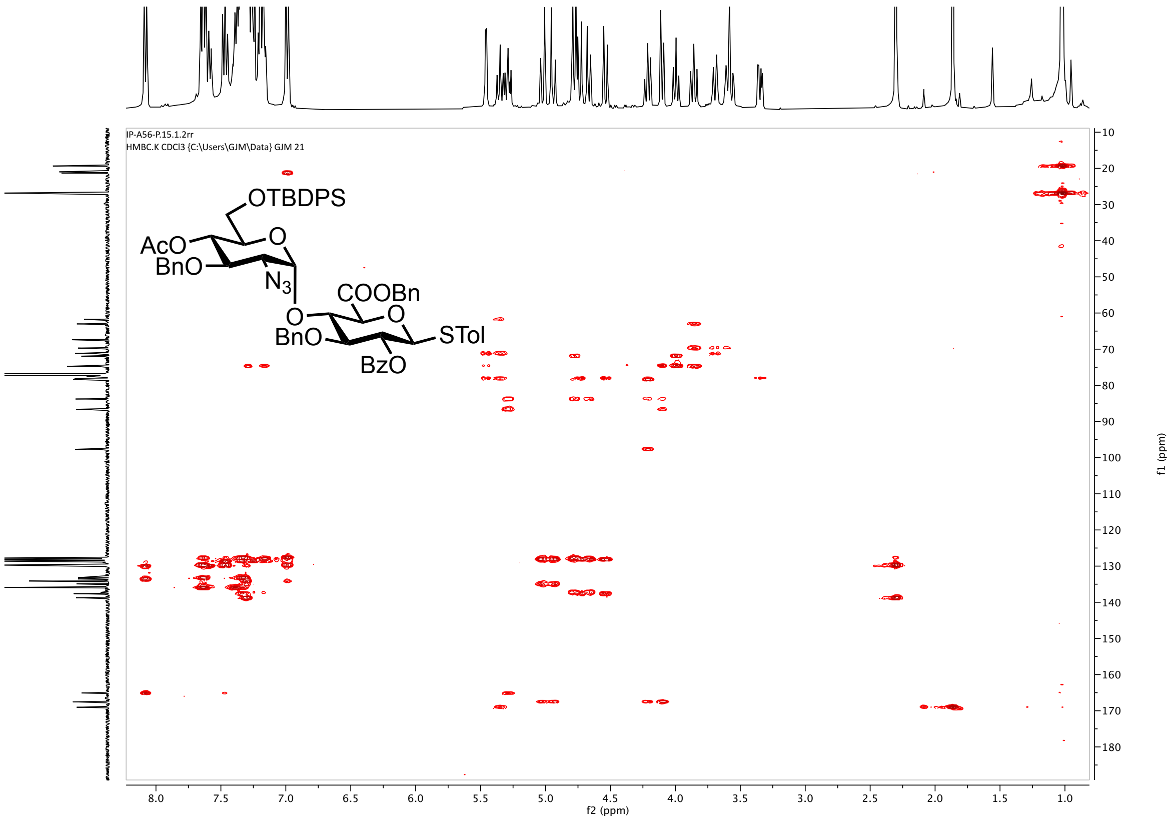

Compound 11b

^{1}H NMR (400 MHz, Chloroform-*d*) 11b

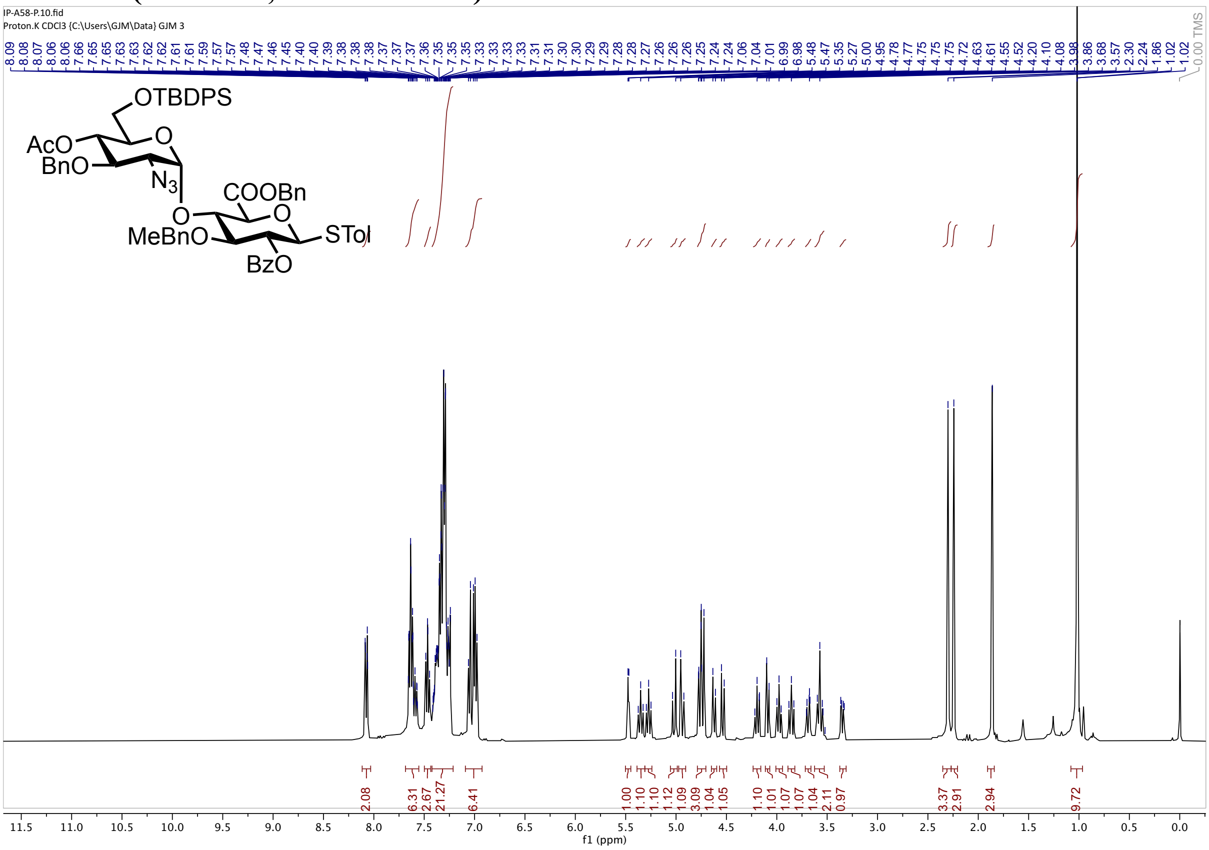

^{13}C{^{1}H} NMR (101 MHz, Chloroform-*d*) 11b

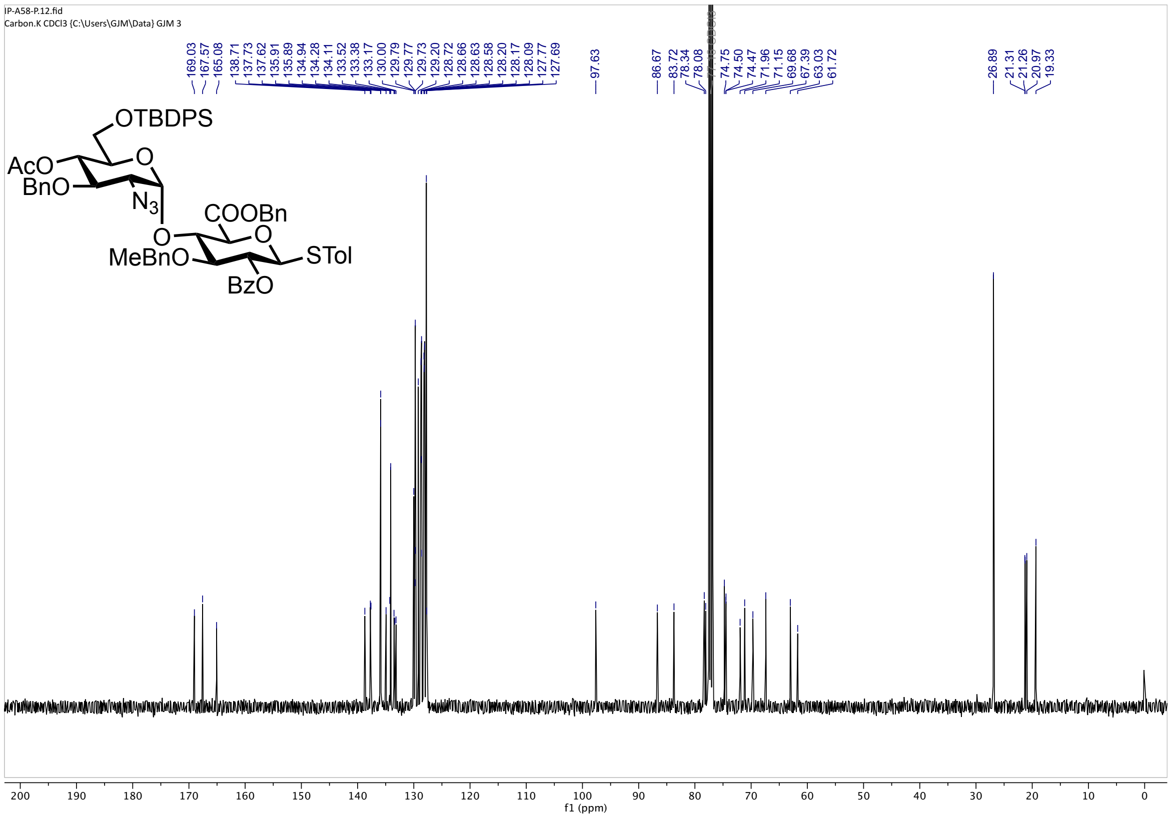

COSY NMR (400 MHz, Chloroform-*d*) 11b

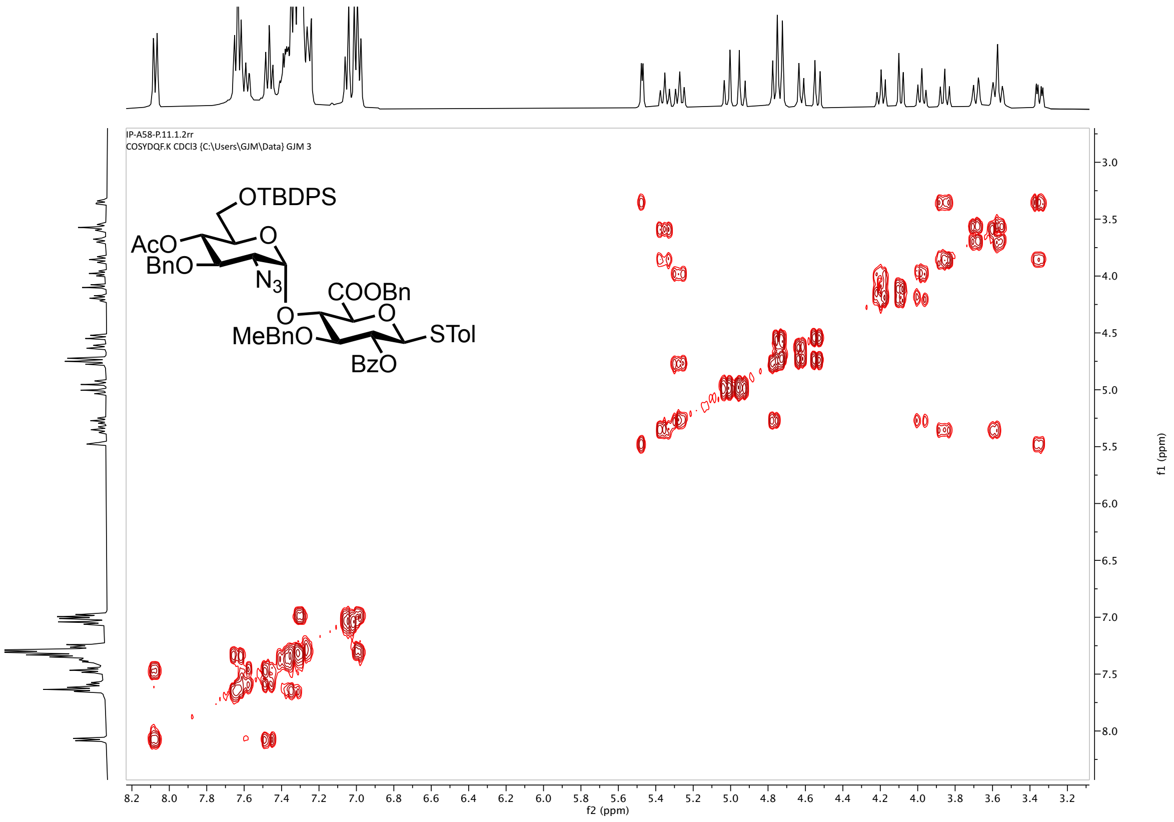

HSQC NMR (400 MHz x 101 MHz, Chloroform-*d*) 11b

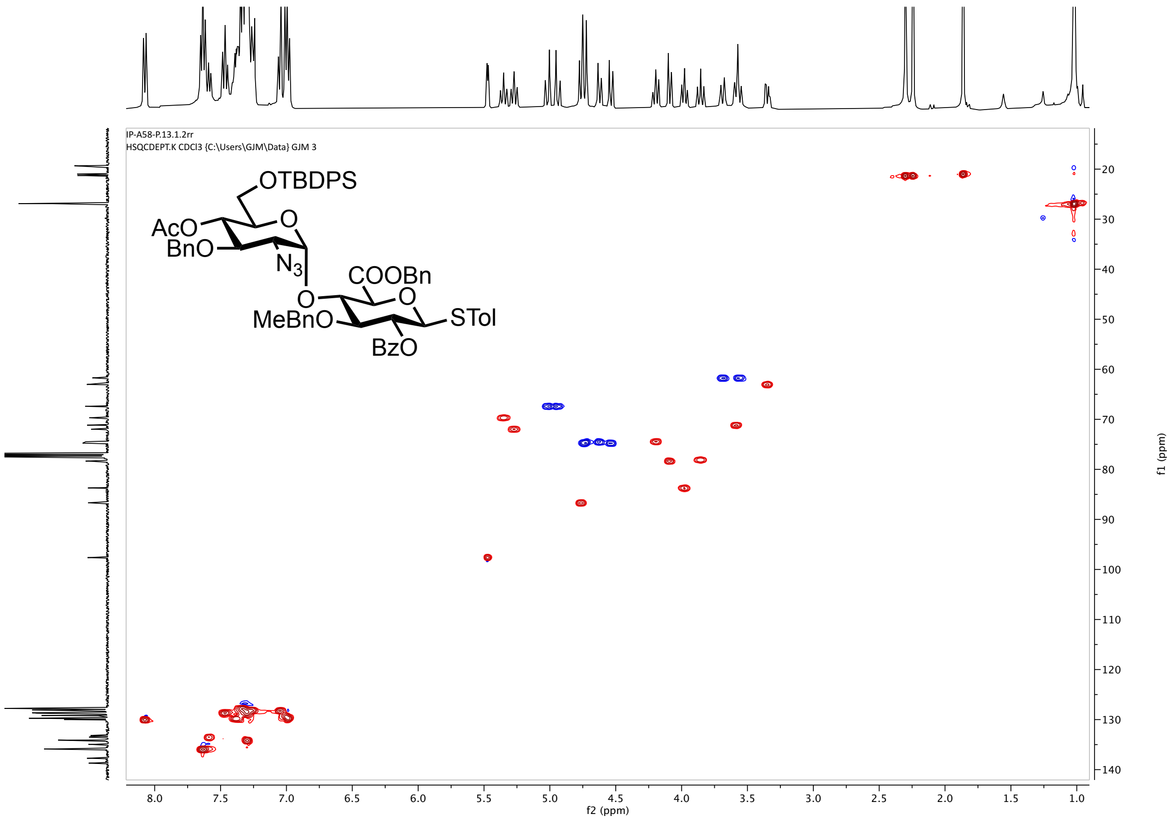

Coupled-HSQC NMR (400 MHz x 101 MHz, Chloroform-*d*) 11b

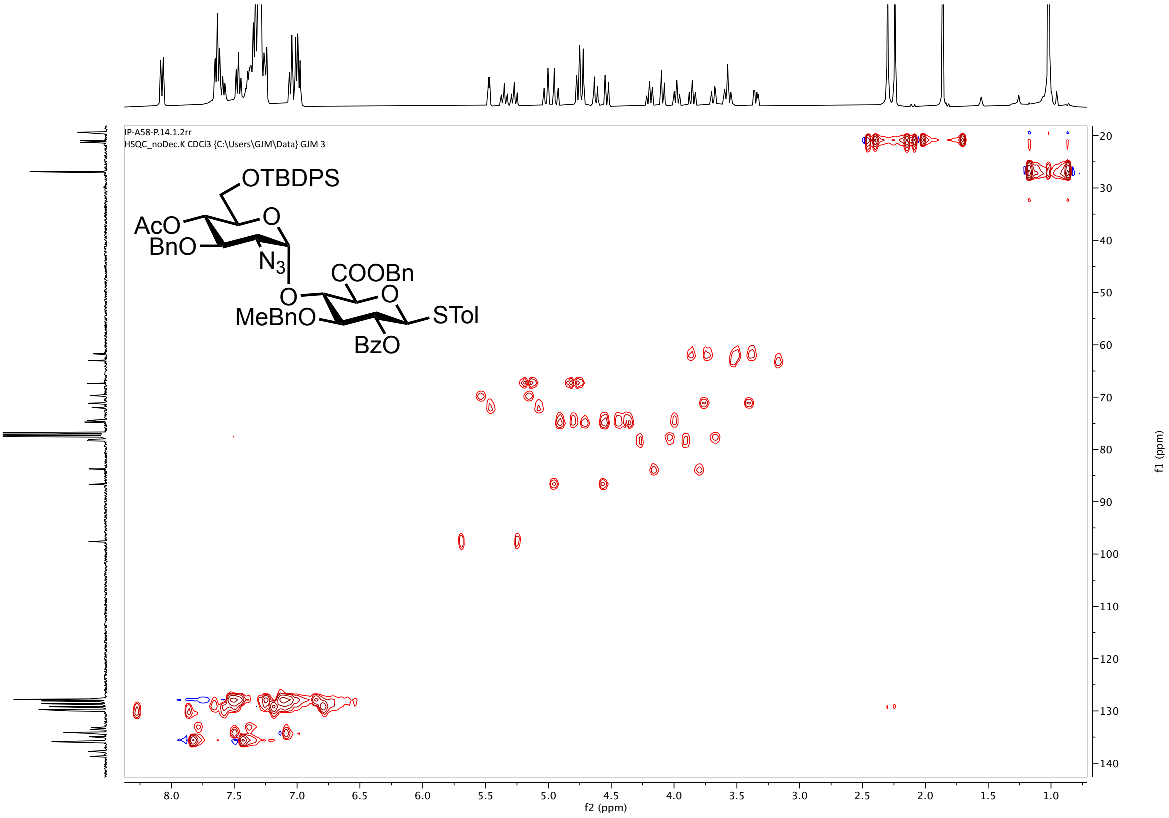

HMBC NMR (400 MHz x 101 MHz, Chloroform-*d*) 11b

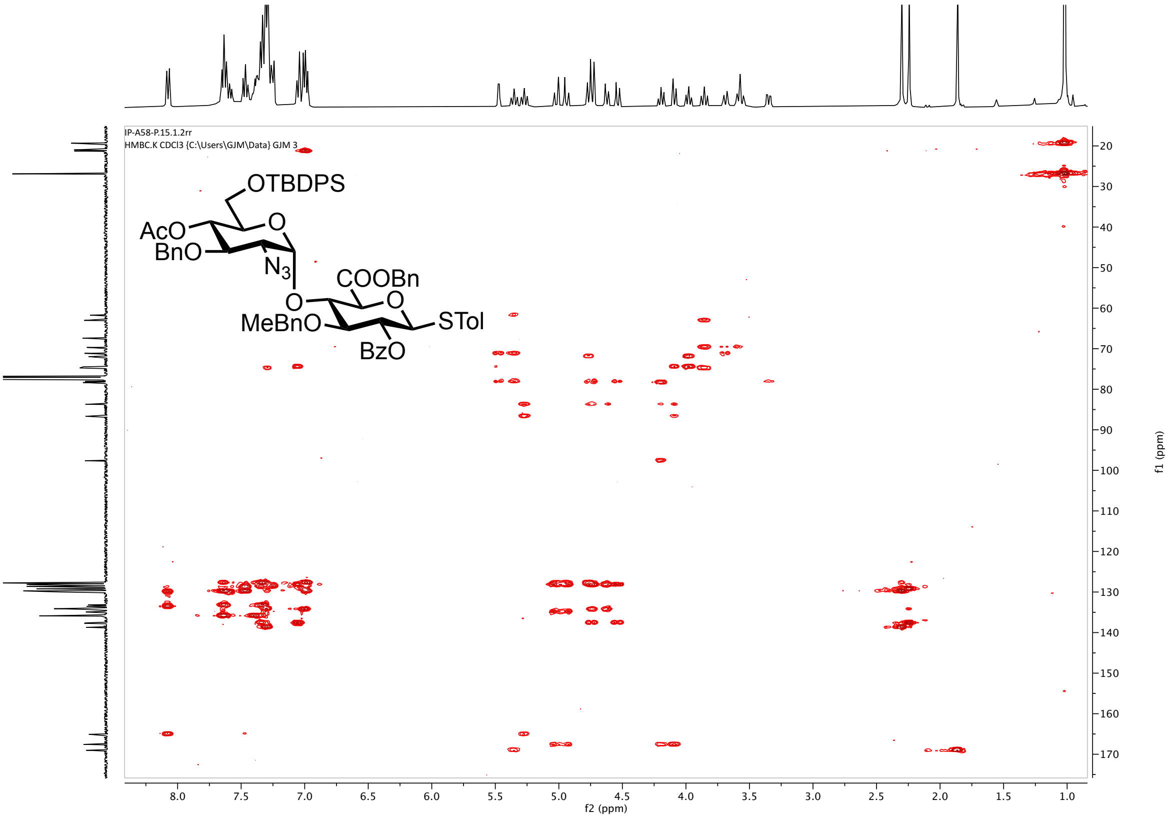

Compound 15

^{1}H NMR (400 MHz, Chloroform-*d*) 15

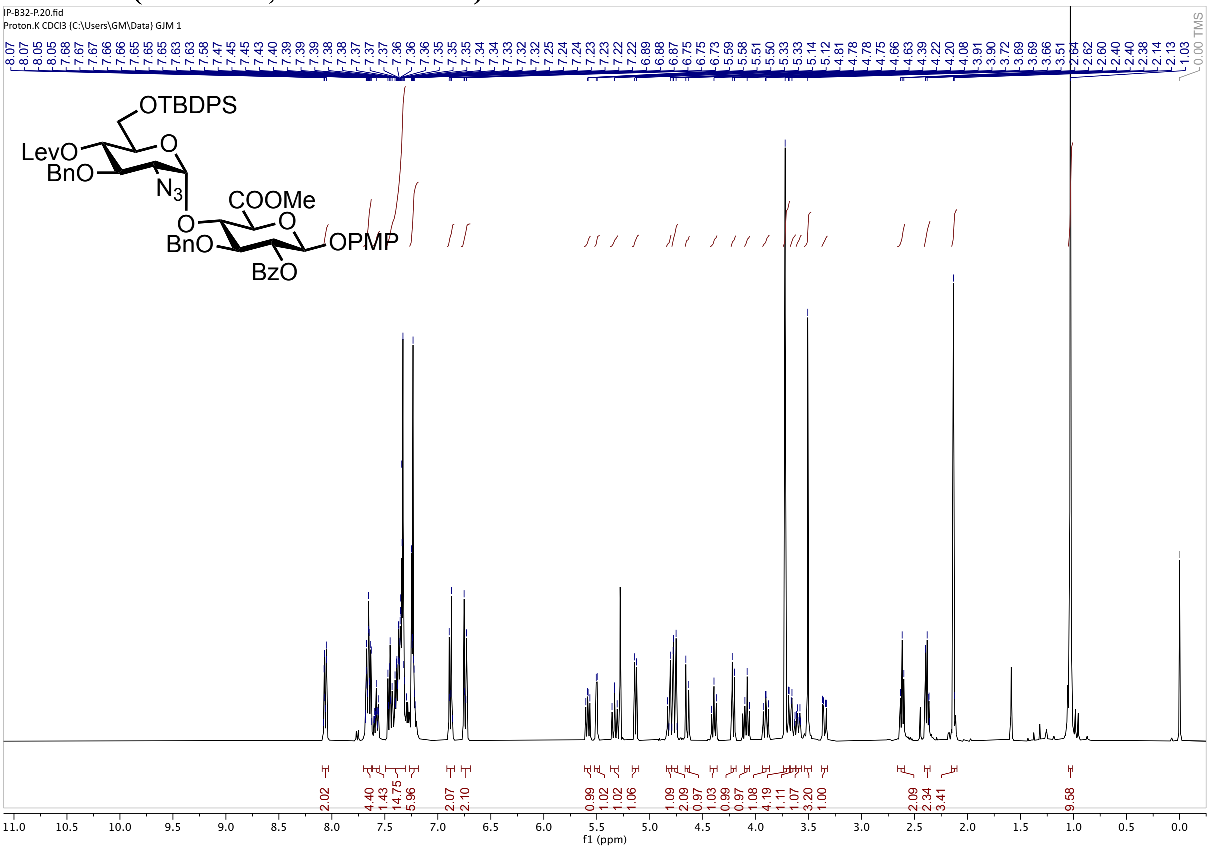

^{13}C{^{1}H} NMR (101 MHz, Chloroform-*d*) 15

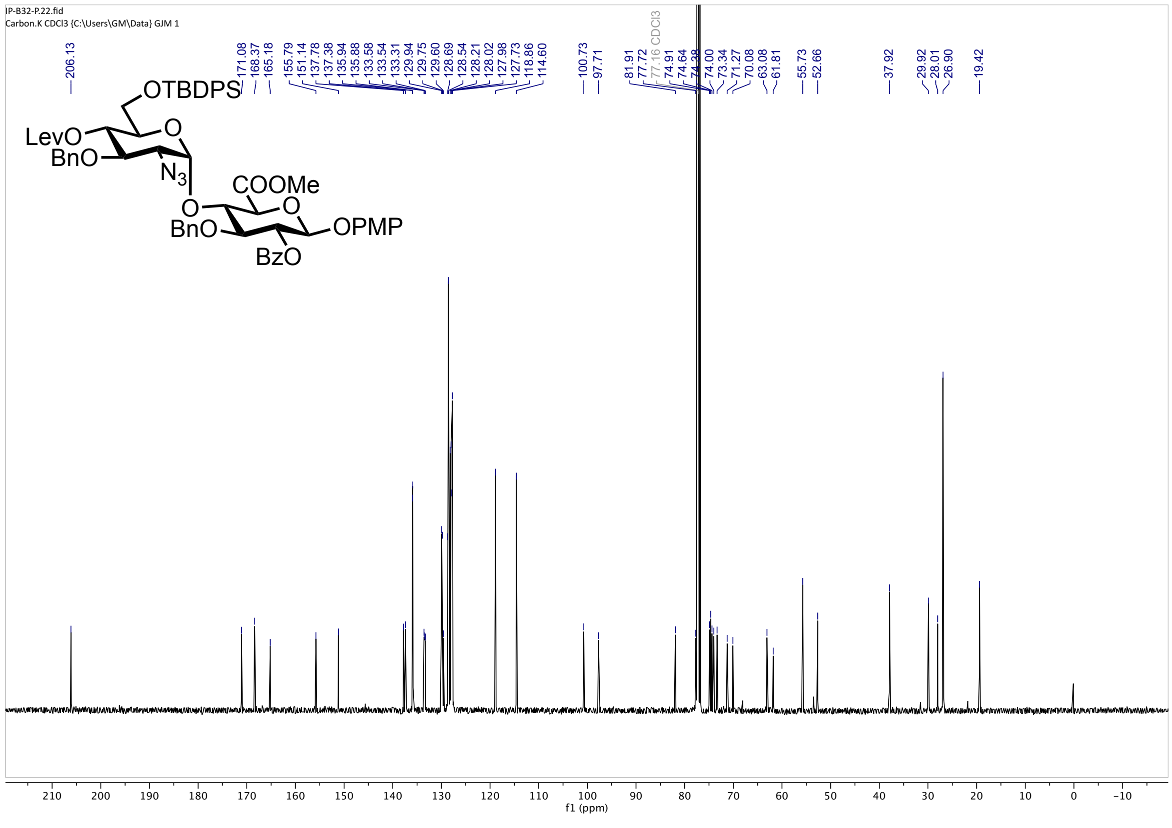

DEPT NMR (101 MHz, Chloroform-*d*) 15

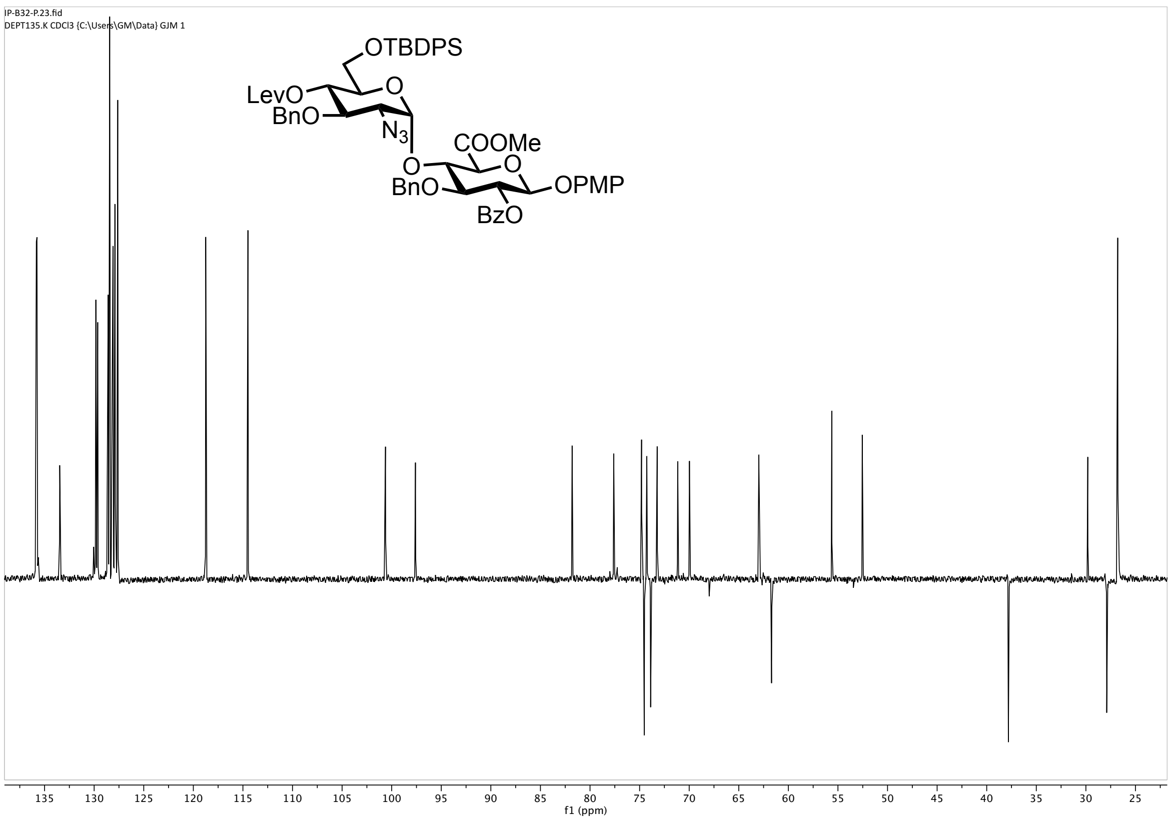

COSY NMR (400 MHz, Chloroform-*d*) 15

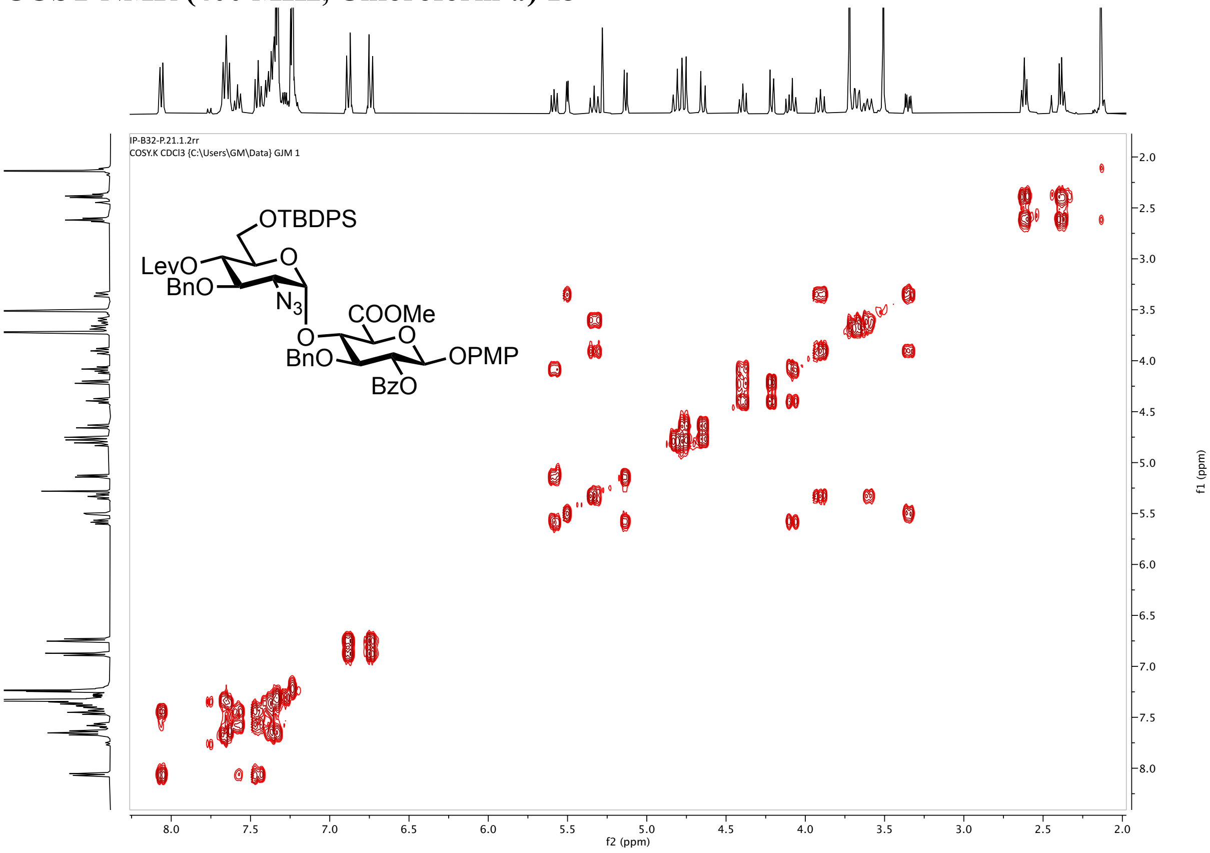

HSQC NMR (400 MHz x 101 MHz, Chloroform-*d*) 15

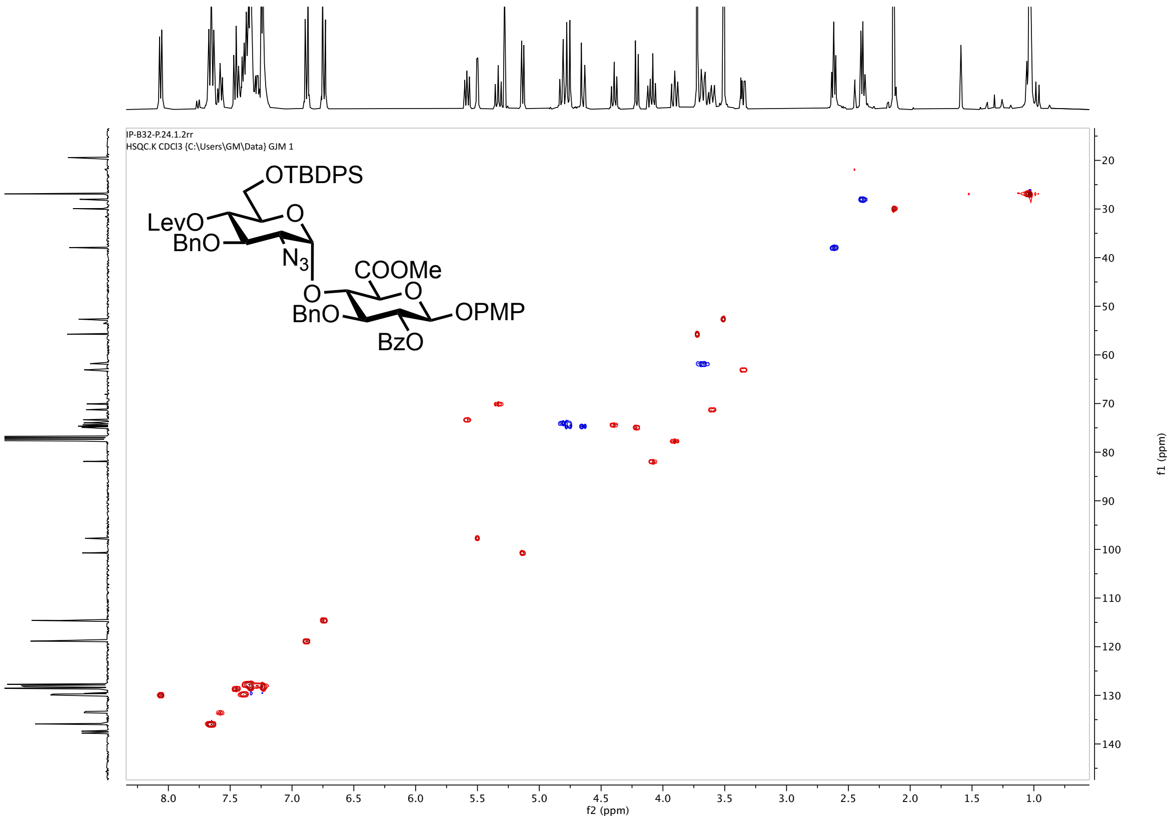

HMBC NMR (400 MHz x 101 MHz, Chloroform-*d*) 15

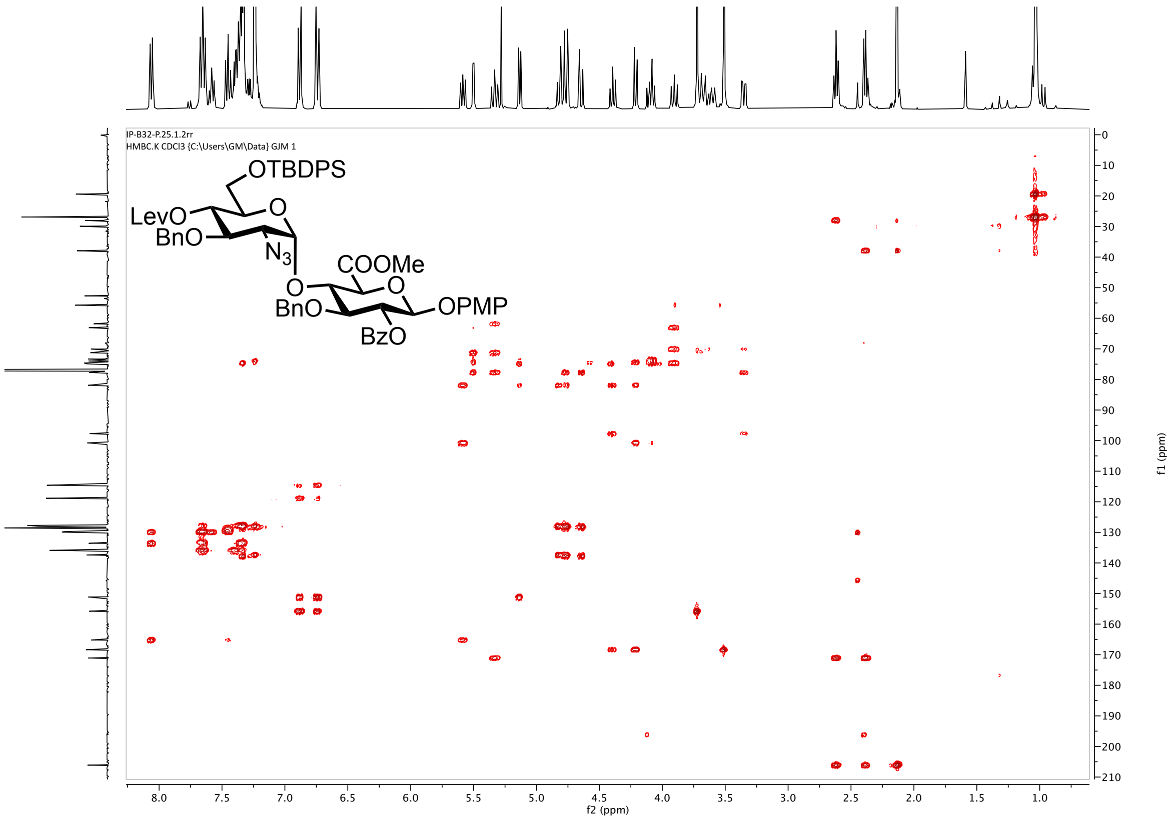

Compound 30

^{1}H NMR (400 MHz, Chloroform-*d*) 30

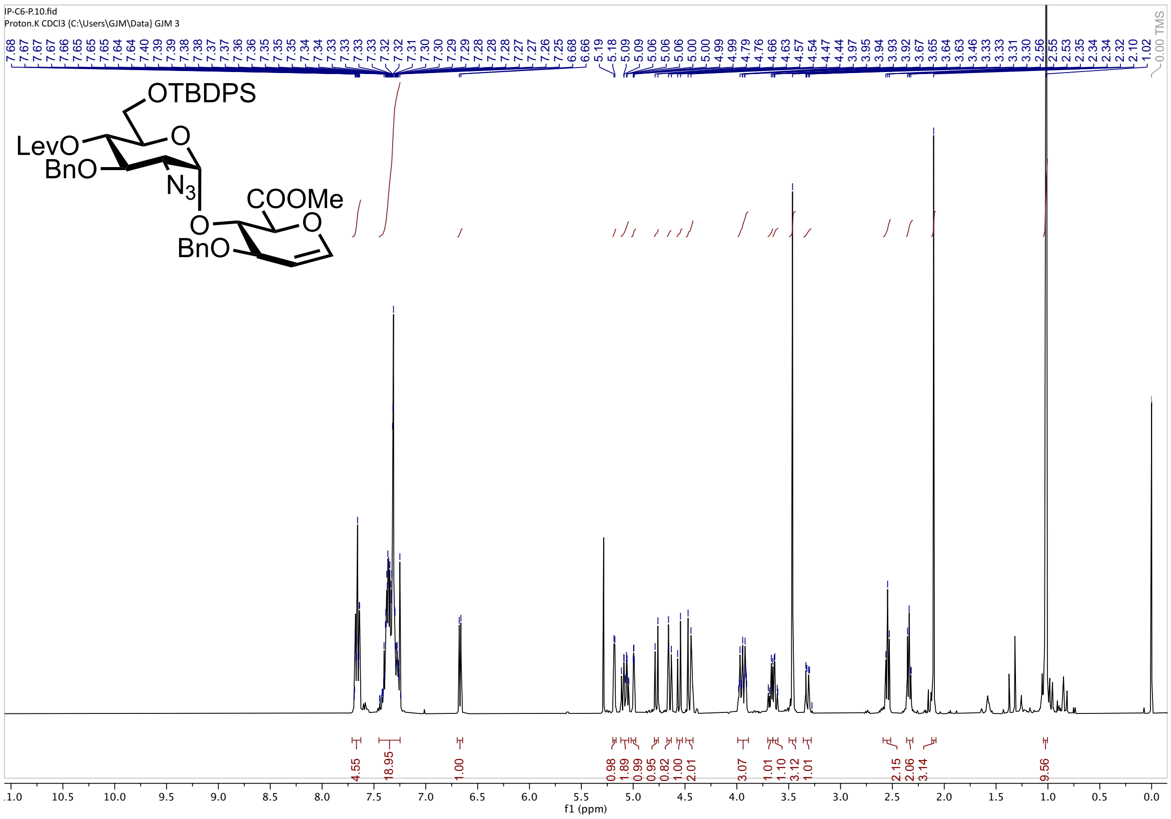

^{13}C{^{1}H} NMR (101 MHz, Chloroform-*d*) 30

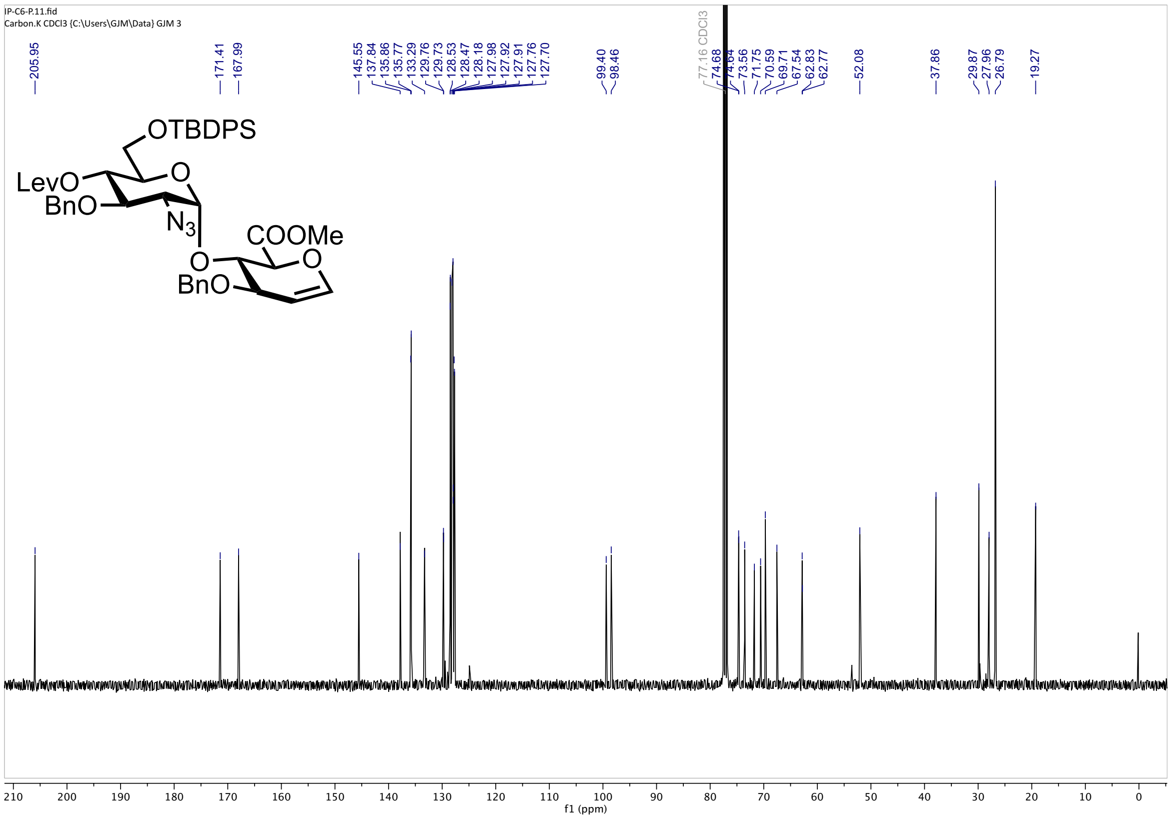

DEPT NMR (101 MHz, Chloroform-*d*) 30

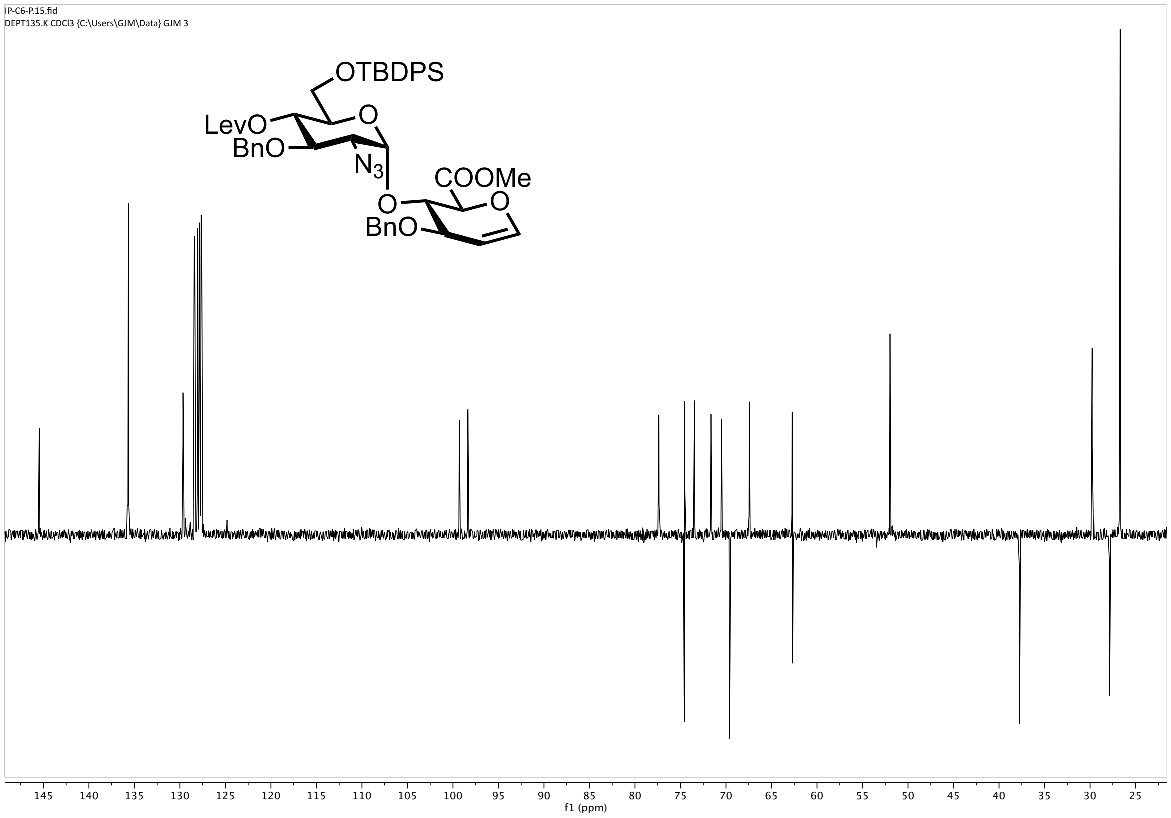

COSY NMR (400 MHz, Chloroform-*d*) 30

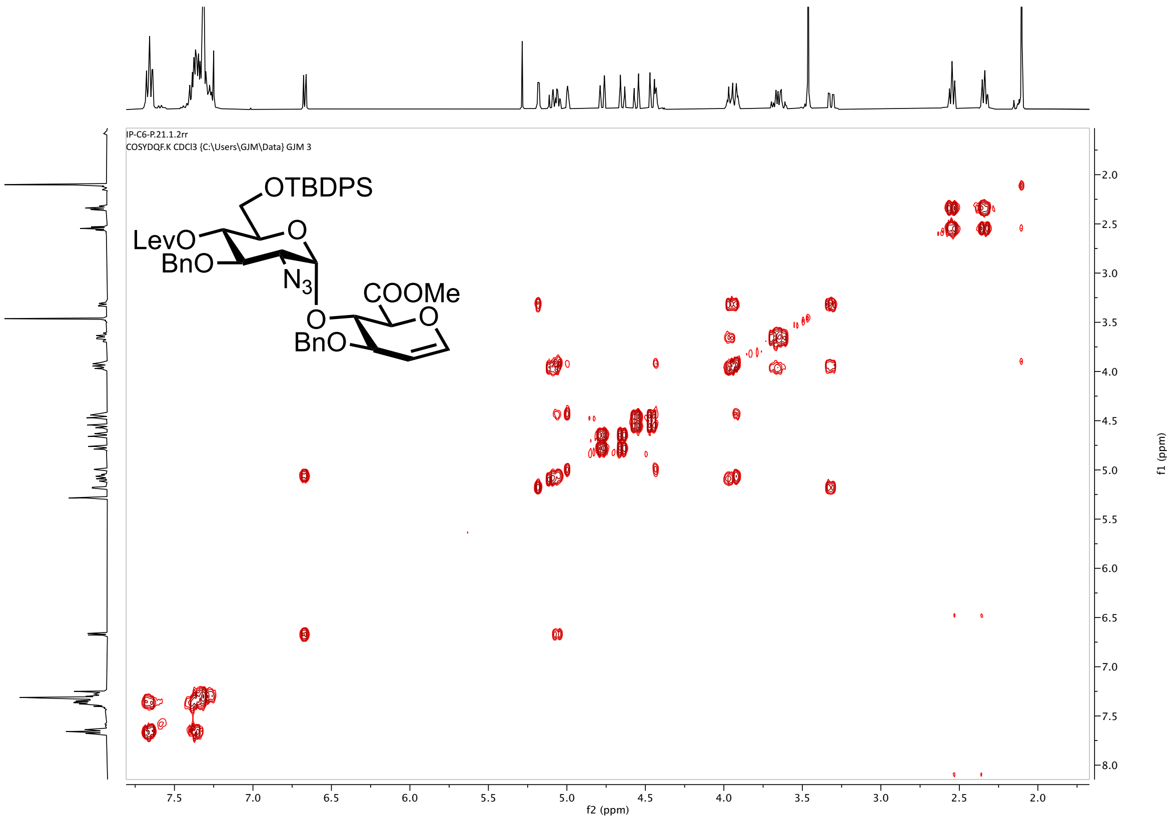

HSQC NMR (400 MHz x 101 MHz, Chloroform-*d*) 30

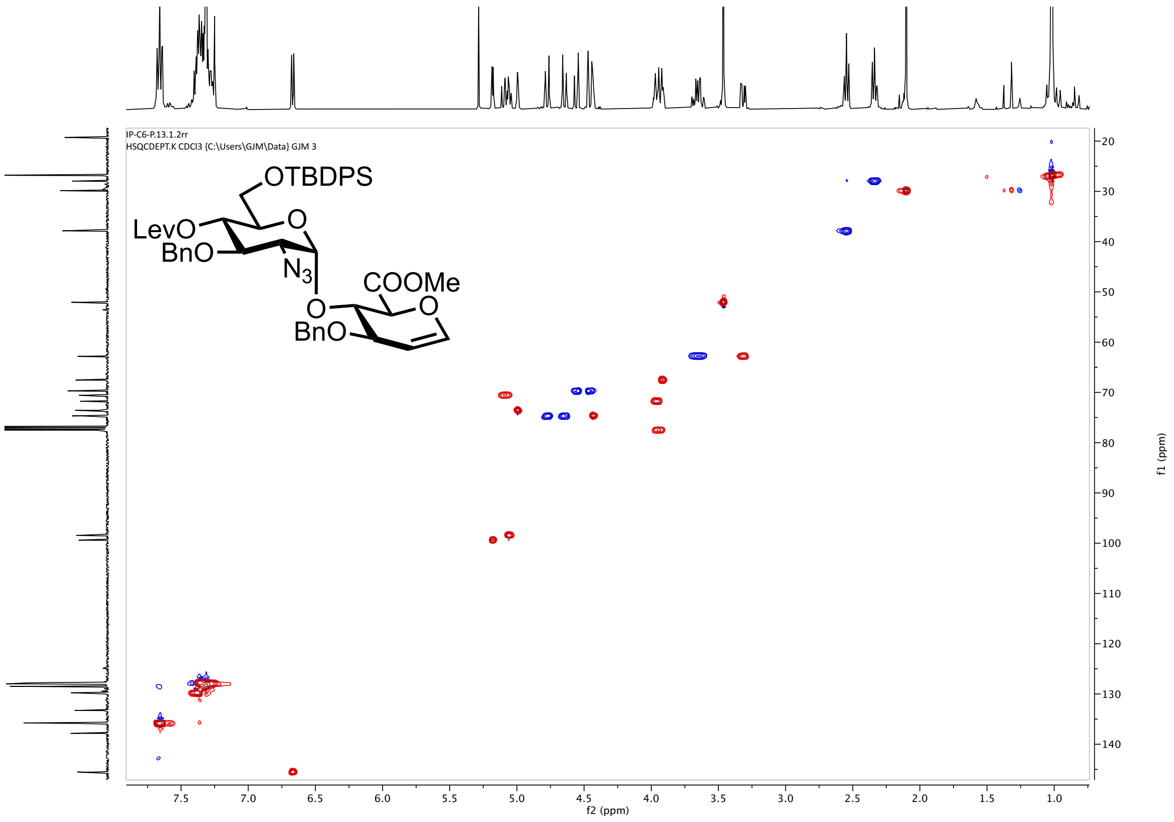

Coupled-HSQC NMR (400 MHz x 101 MHz, Chloroform-*d*) 30

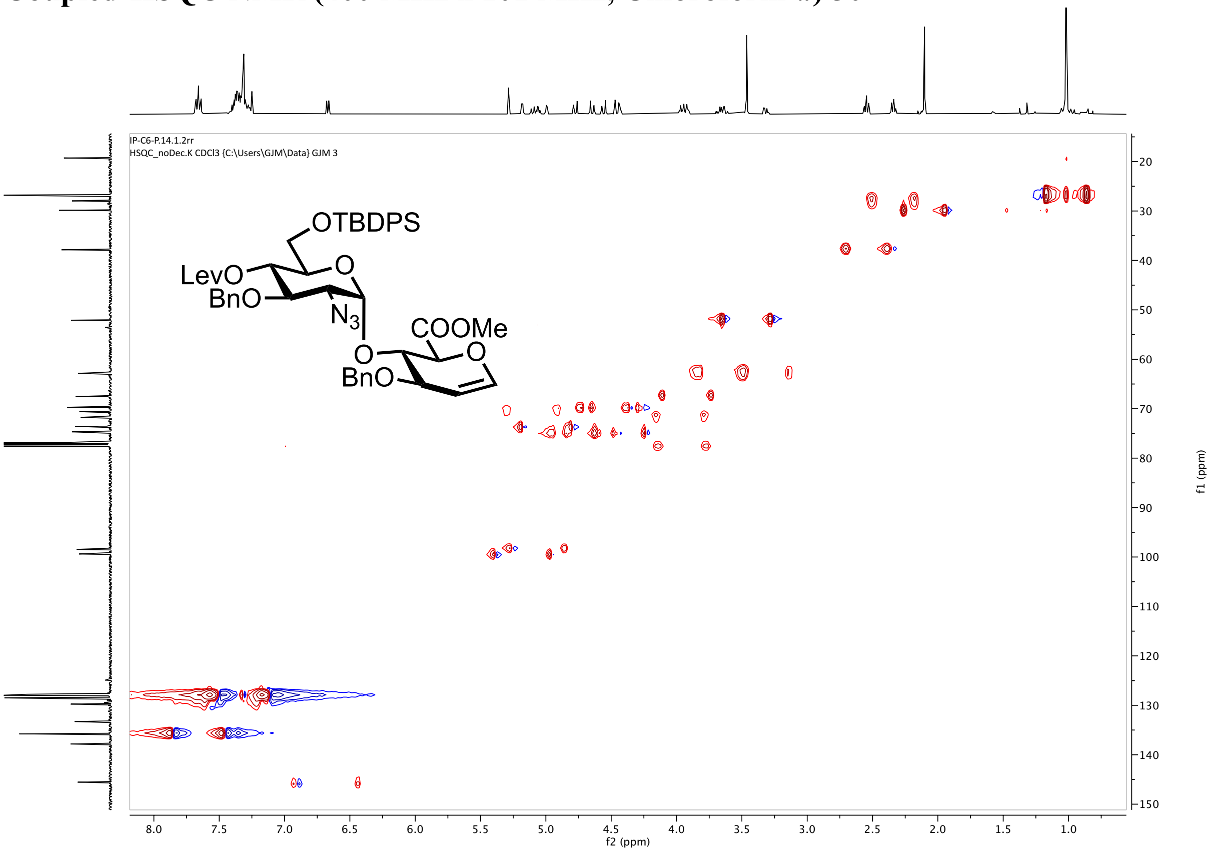

HMBC NMR (400 MHz x 101 MHz, Chloroform-*d*) 30

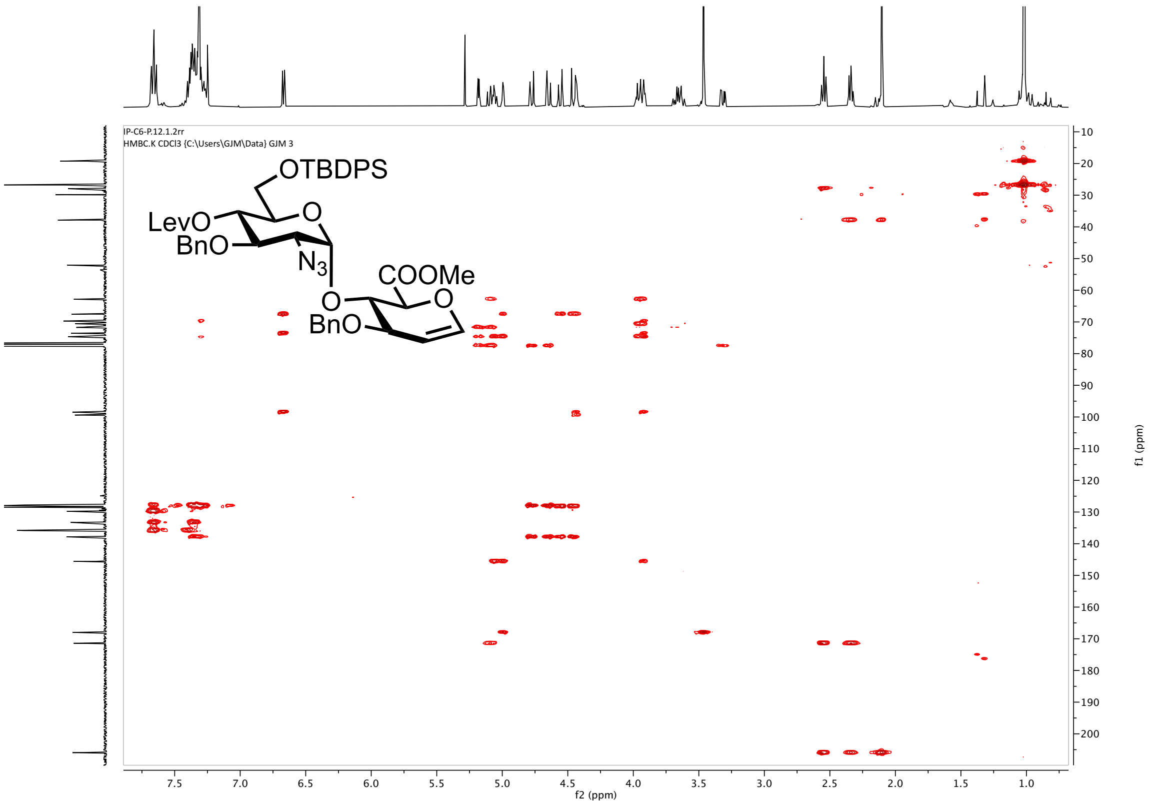

Glycosylation Side Products

Compound 6a

^{1}H NMR (400 MHz, Chloroform-*d*) inseparable mixture of 9a:6a:12 = 55:17:28

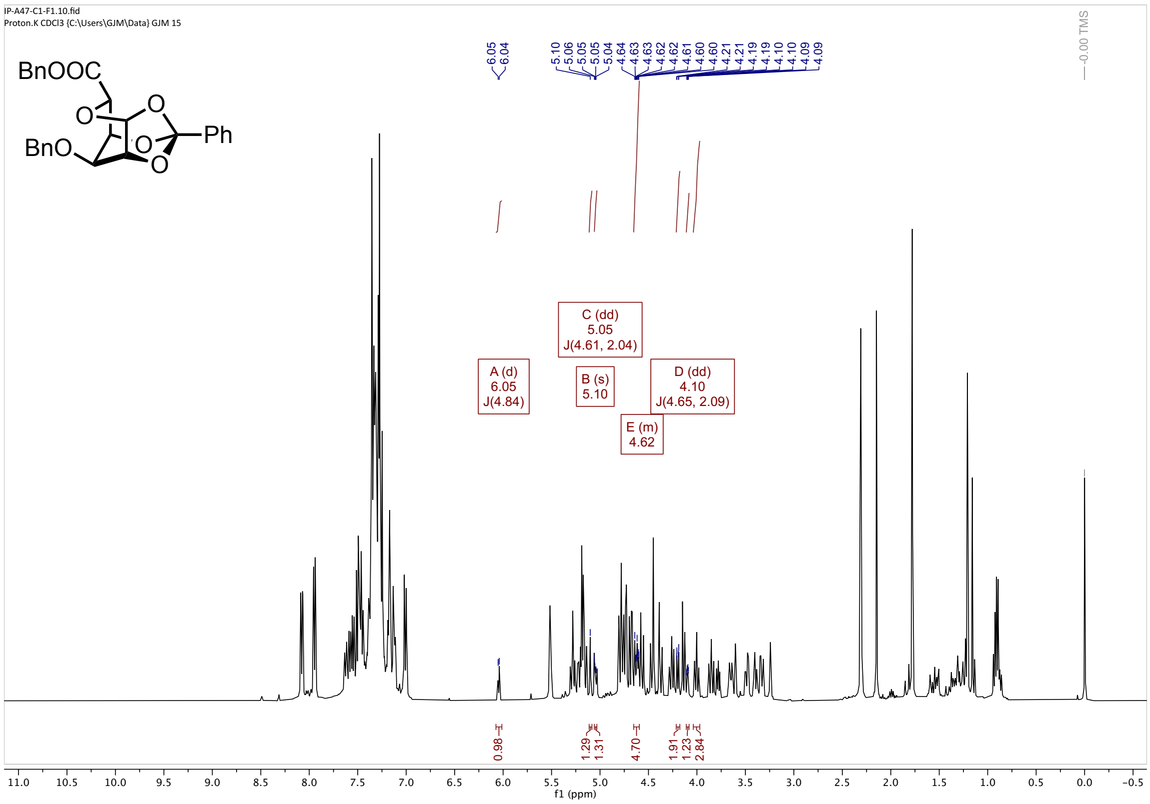

$^{13}C\{^{1}H\}$ NMR (101 MHz, Chloroform-*d*) inseparable mixture of 9a:6a:12 = 55:17:28

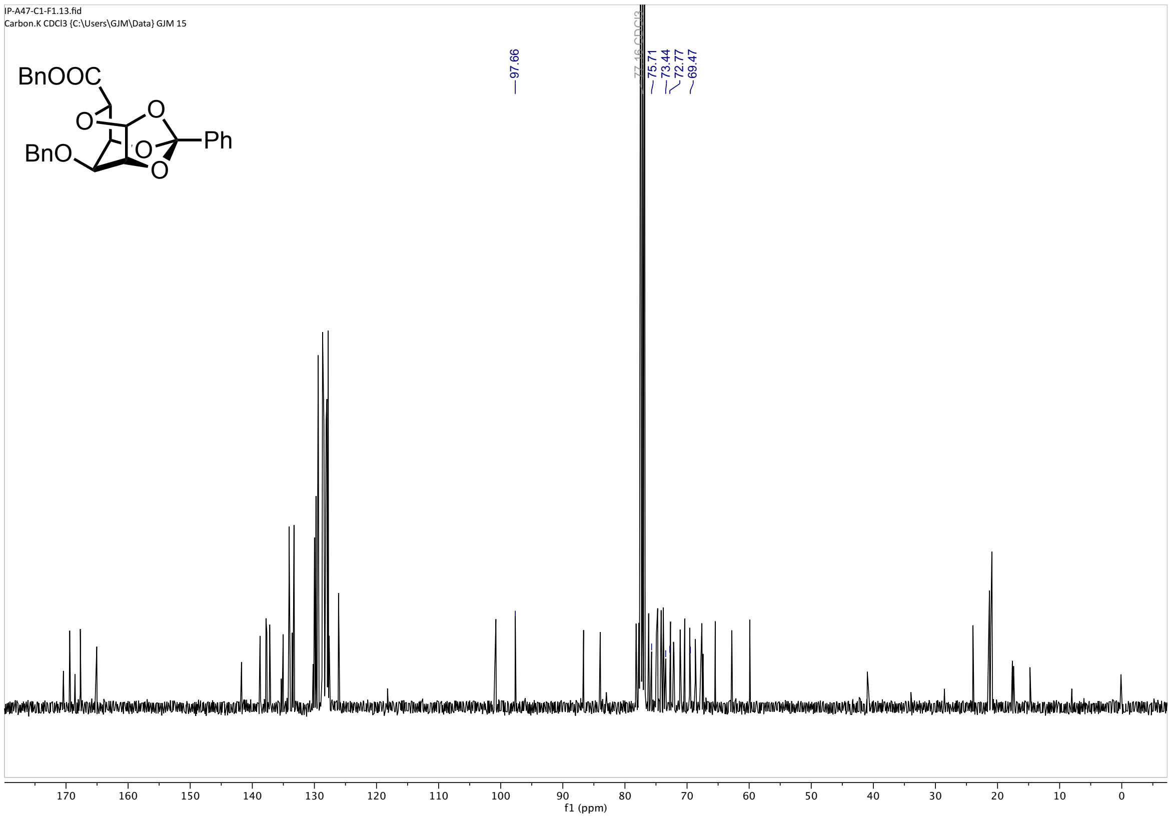

COSY NMR (400 MHz, Chloroform-*d*) inseparable mixture of 9a:6a:12 = 55:17:28

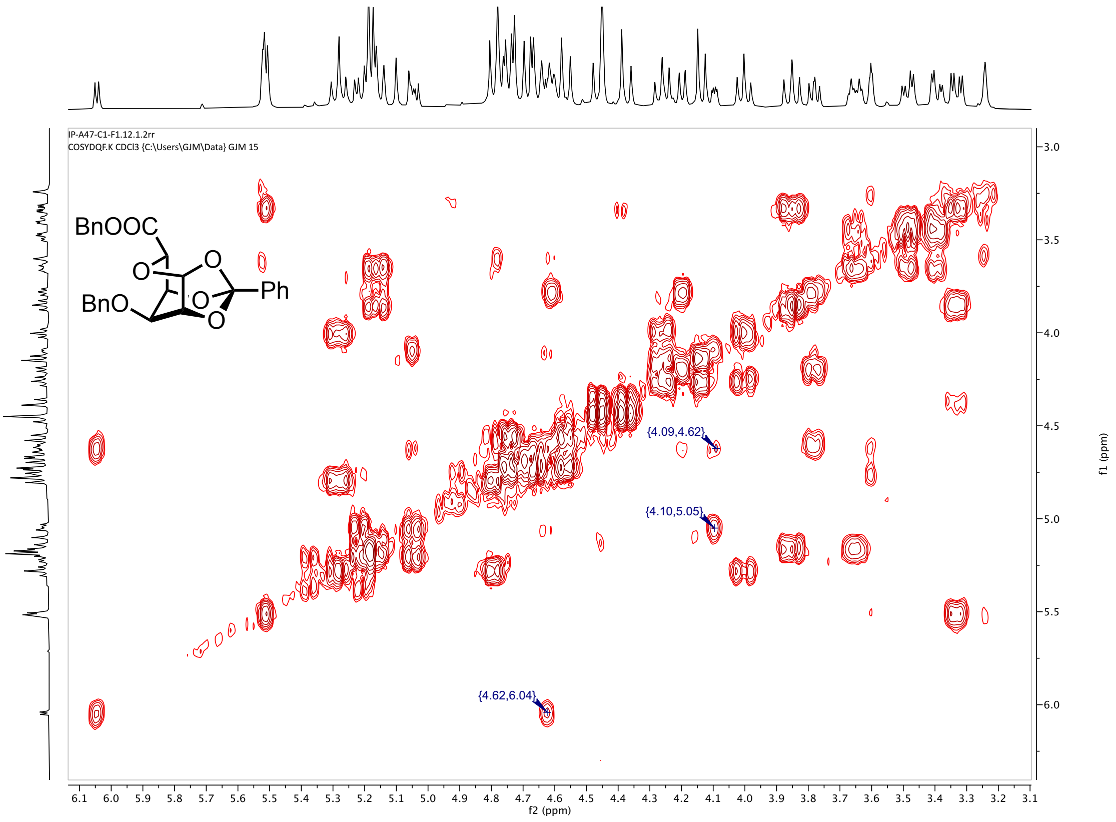

HSQC NMR (400 MHz x 101 MHz, Chloroform-*d*) inseparable mixture of 9a:6a:12 = 55:17:28

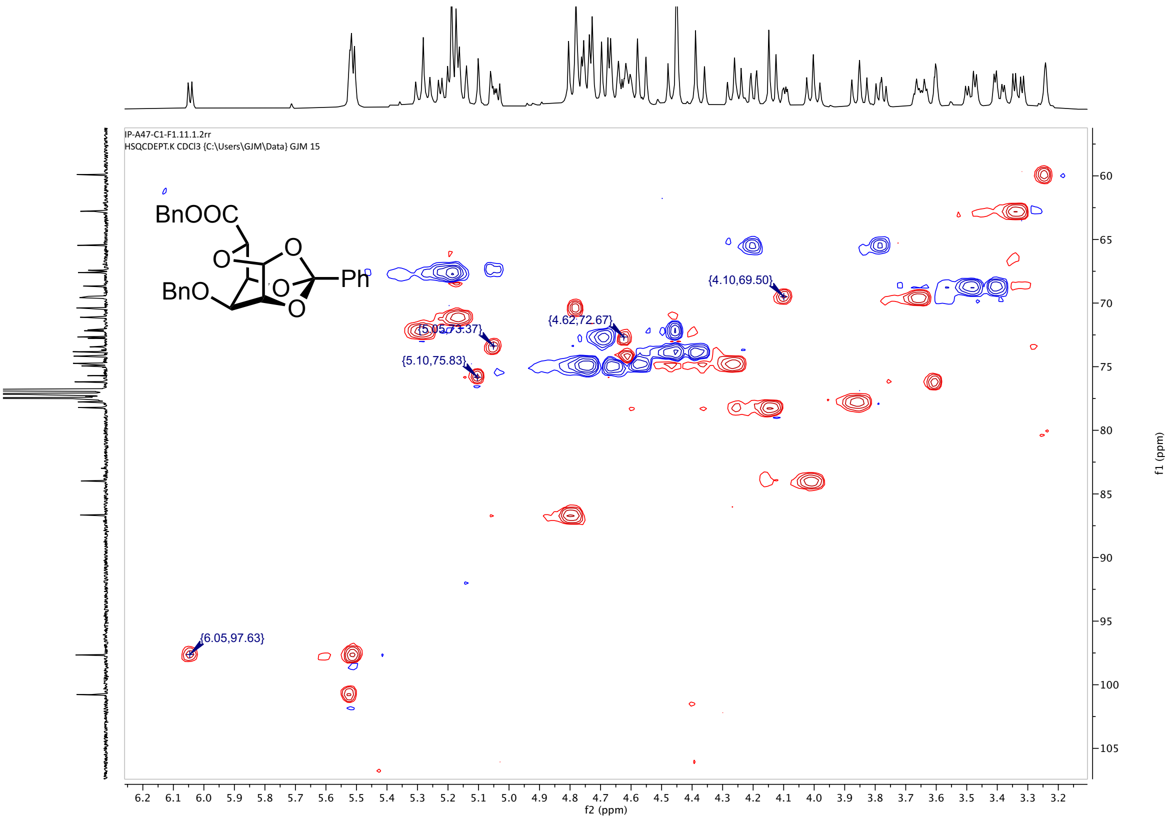

Compound 6b

^{1}H NMR (400 MHz, Chloroform-*d*) 6b

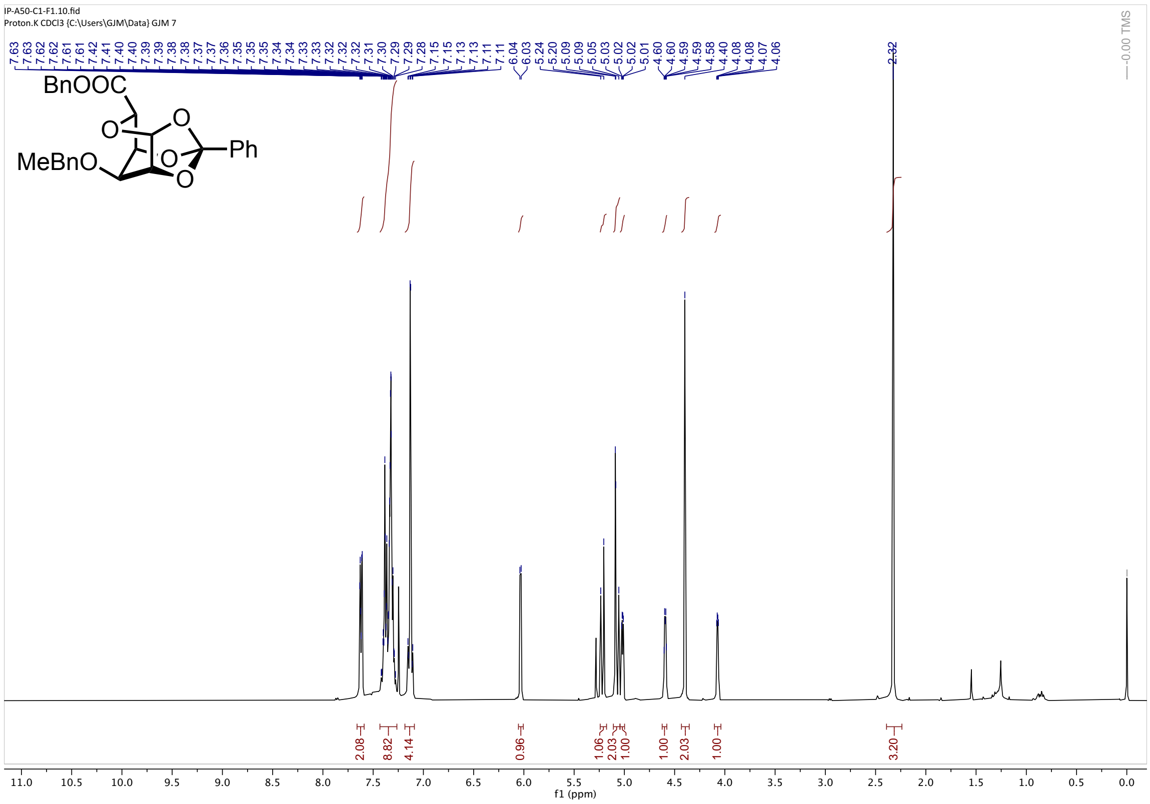

$^{13}C\{^{1}H\}$ NMR (101 MHz, Chloroform-*d*) 6b

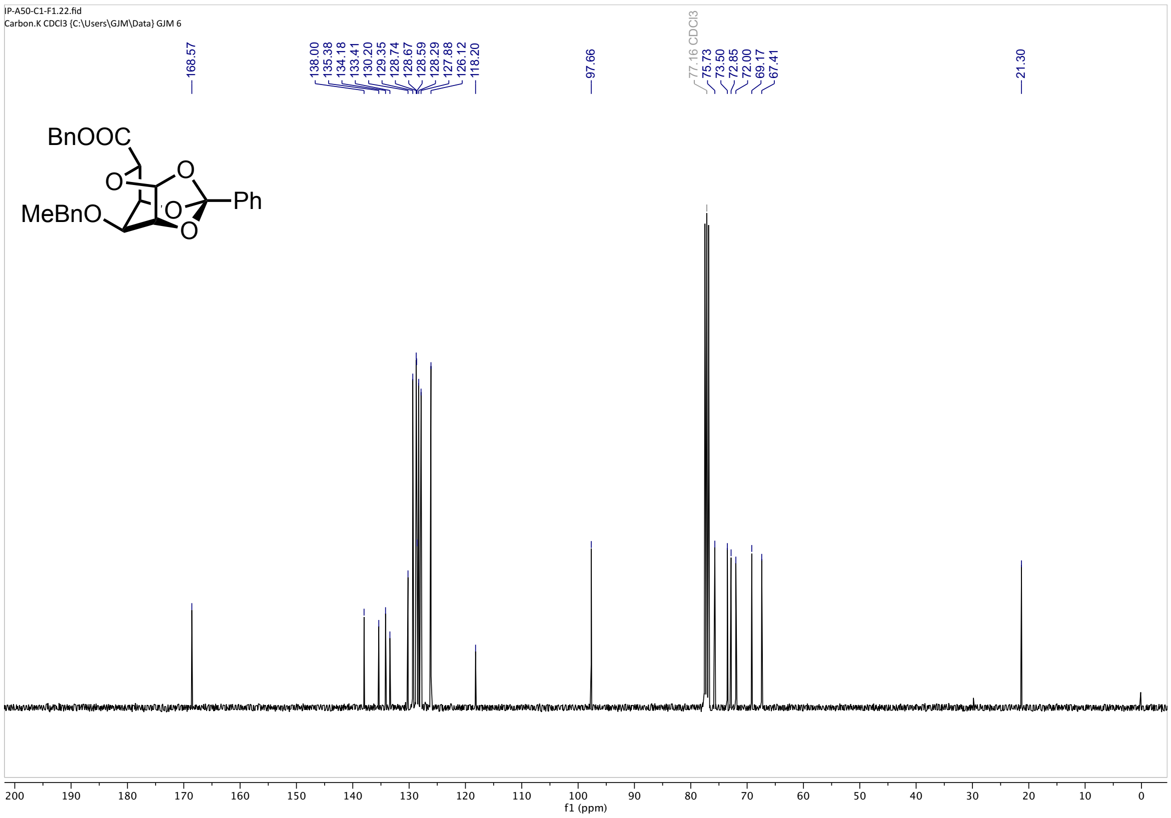

COSY NMR (400 MHz, Chloroform-*d*) 6b

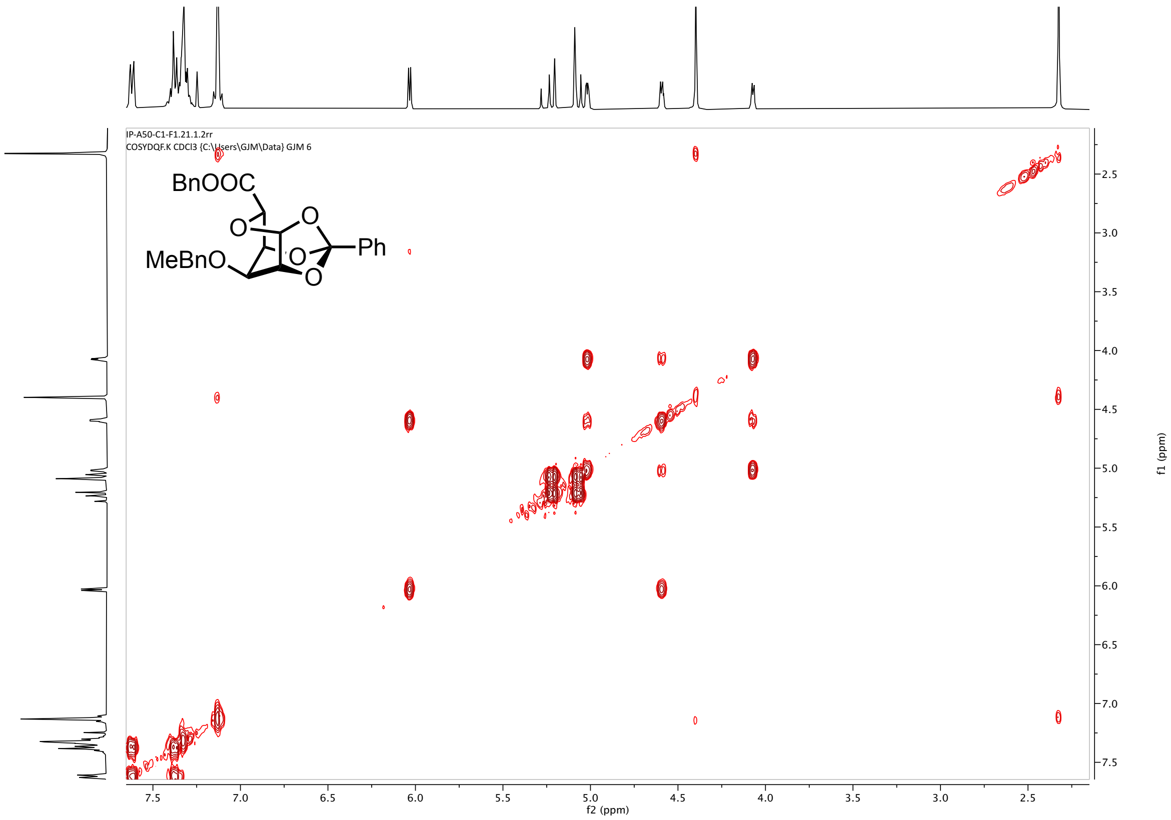

HSQC NMR (400 MHz x 101 MHz, Chloroform-*d*) 6b

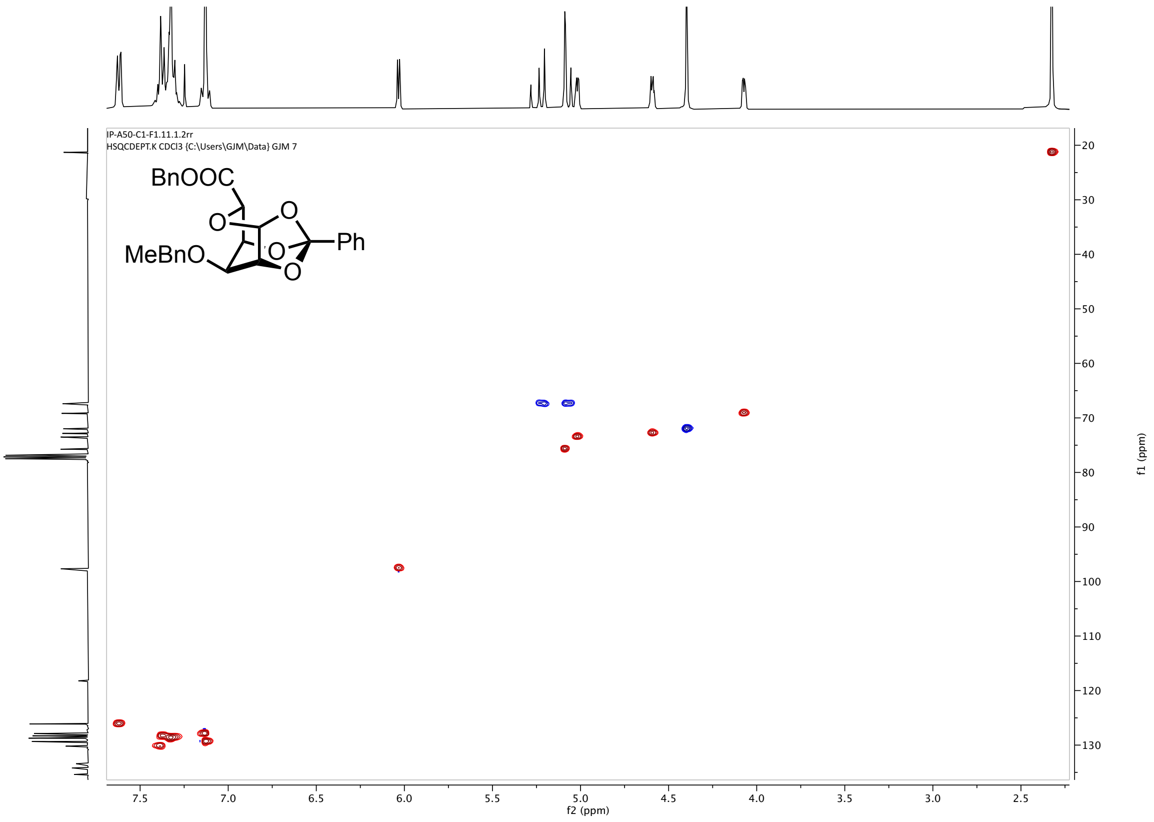

Coupled-HSQC NMR (400 MHz x 101 MHz, Chloroform-*d*) 6b

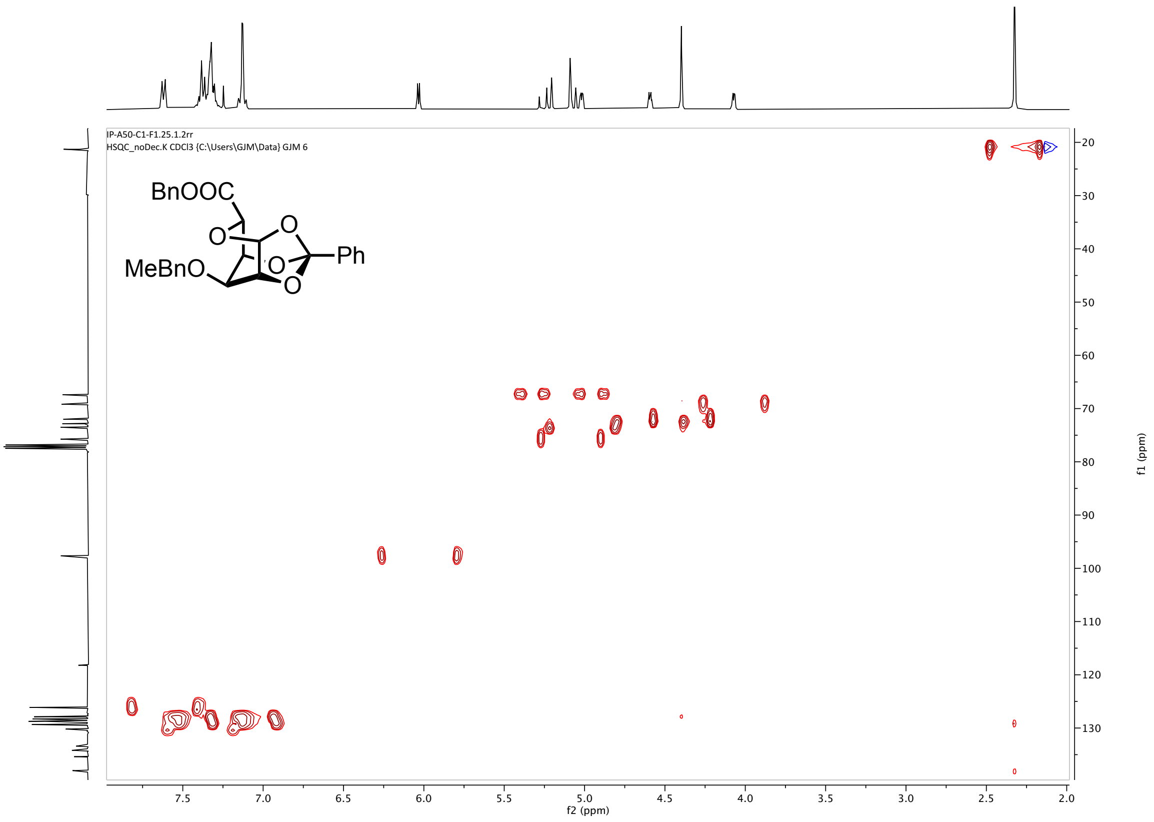

HMBC NMR (400 MHz x 101 MHz, Chloroform-*d*) 6b

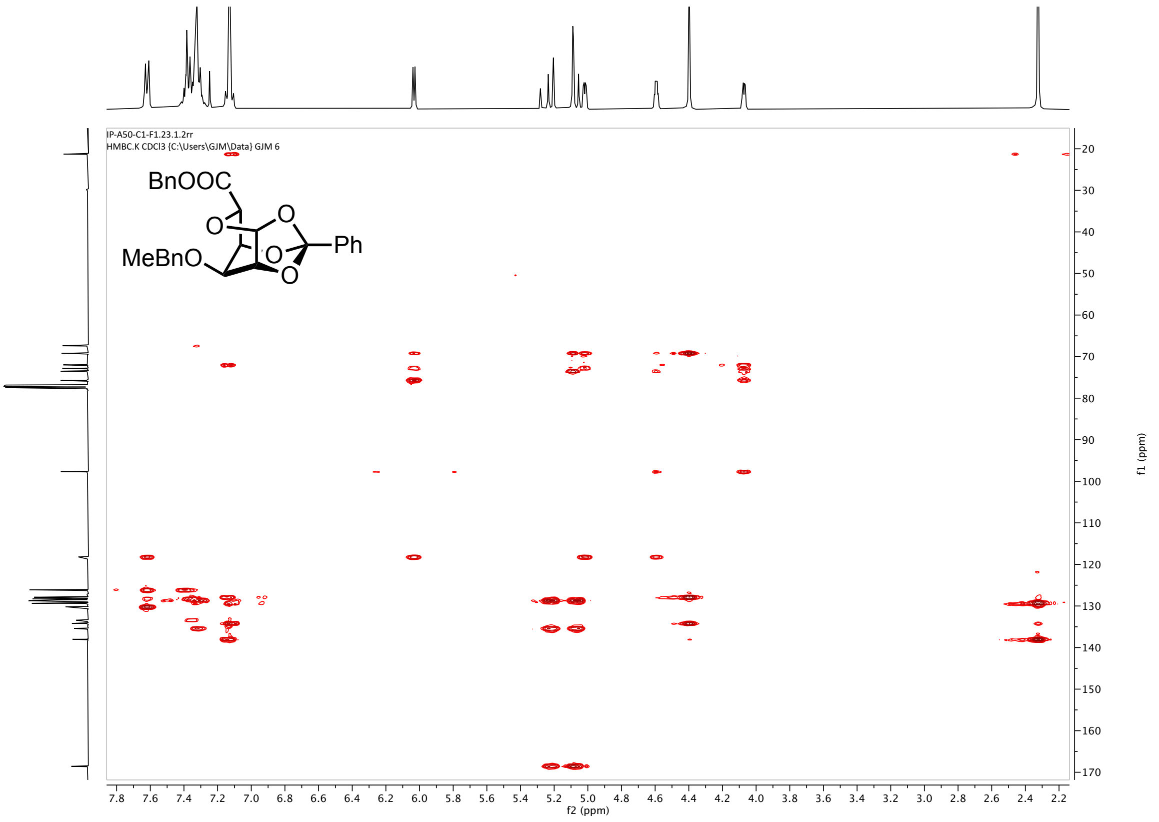

Compound 7a

^{1}H NMR (400 MHz, Chloroform-*d*) 7a

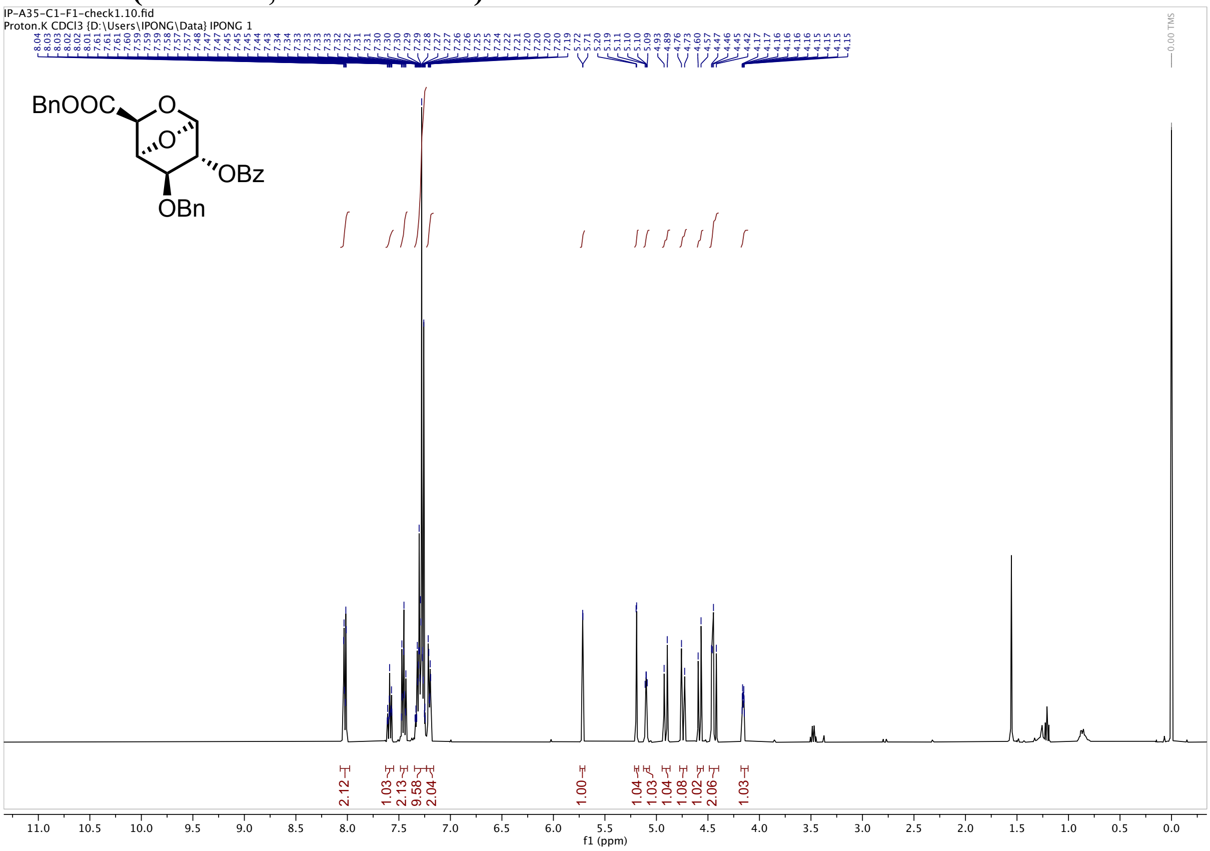

$^{13}C\{^{1}H\}$ NMR (101 MHz, Chloroform-*d*) 7a

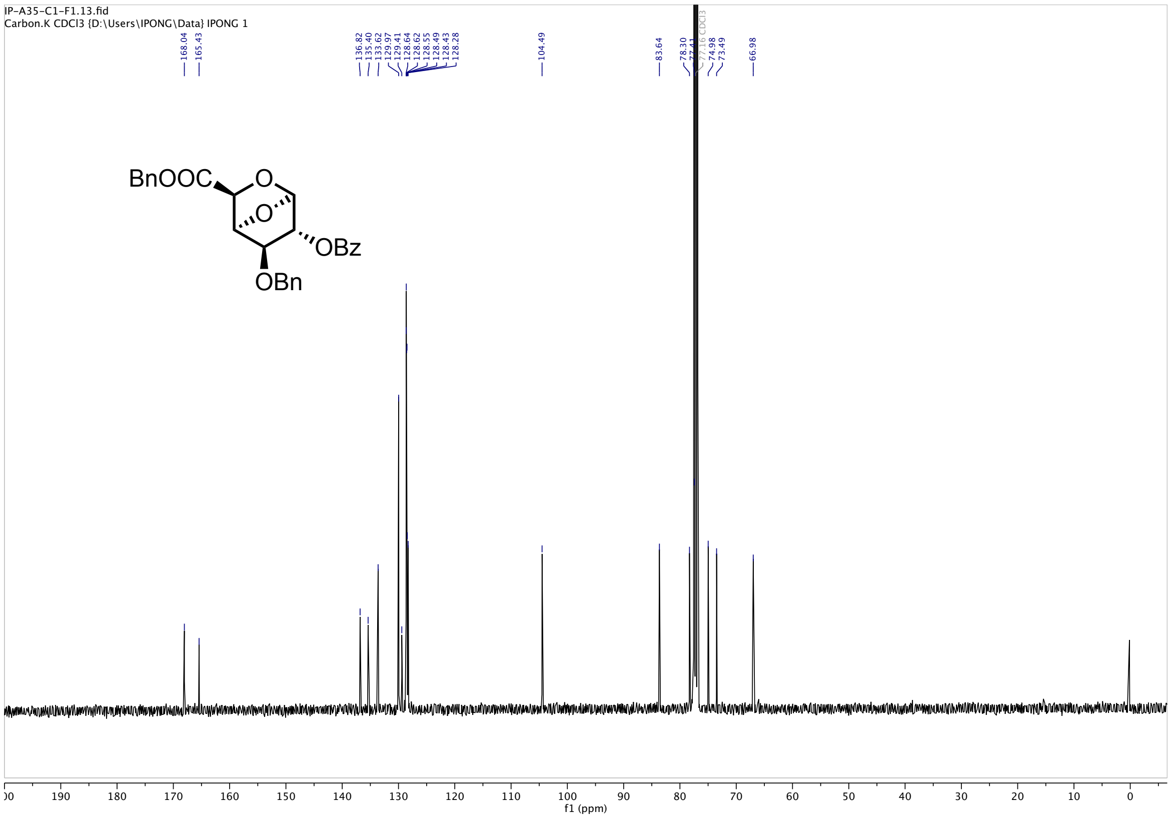

COSY NMR (400 MHz, Chloroform-*d*) 7a

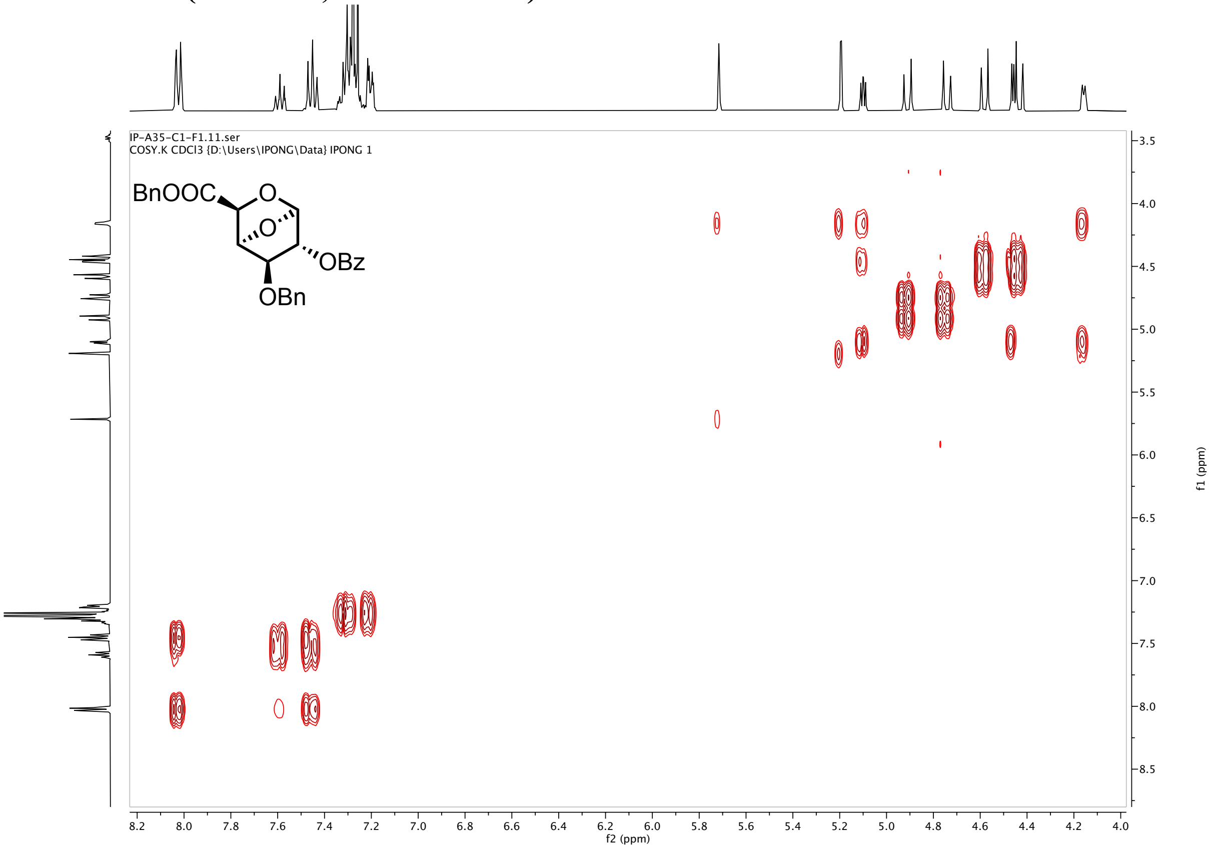

HSQC NMR (400 MHz x 101 MHz, Chloroform-*d*) 7a

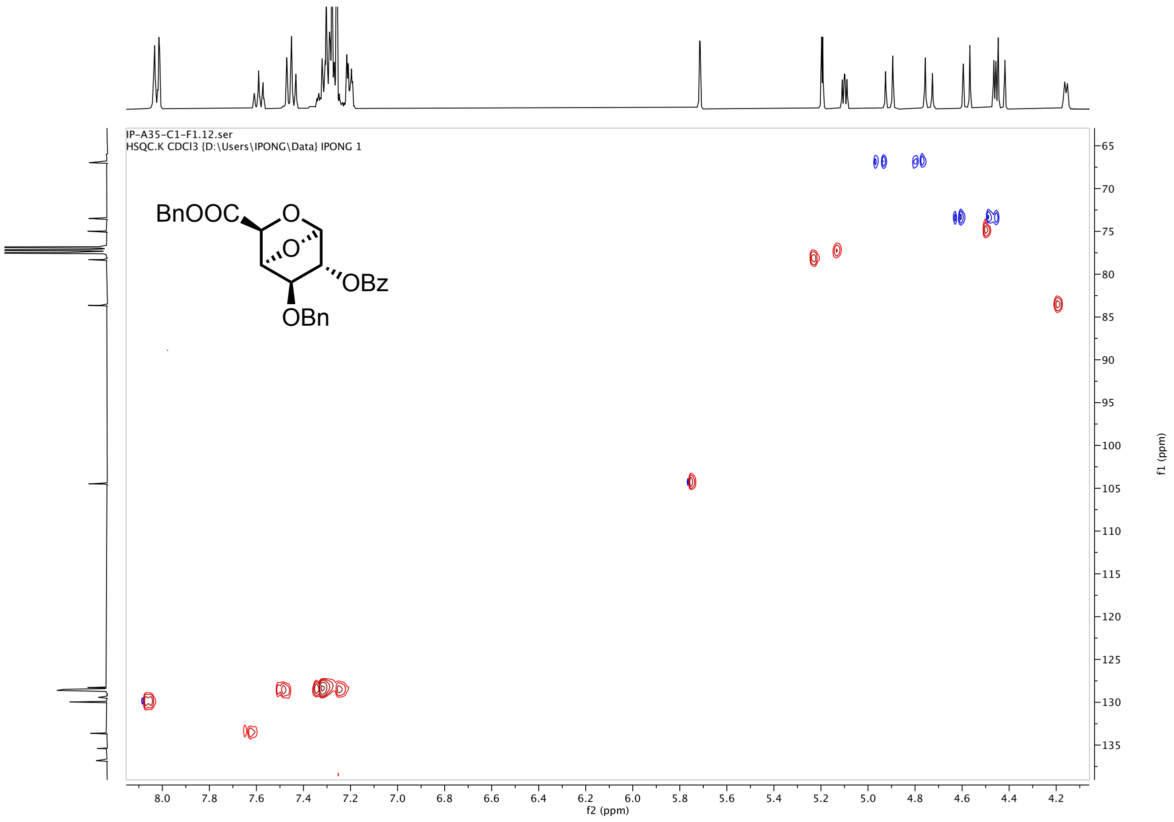

HMBC NMR (400 MHz x 101 MHz, Chloroform-*d*) 7a

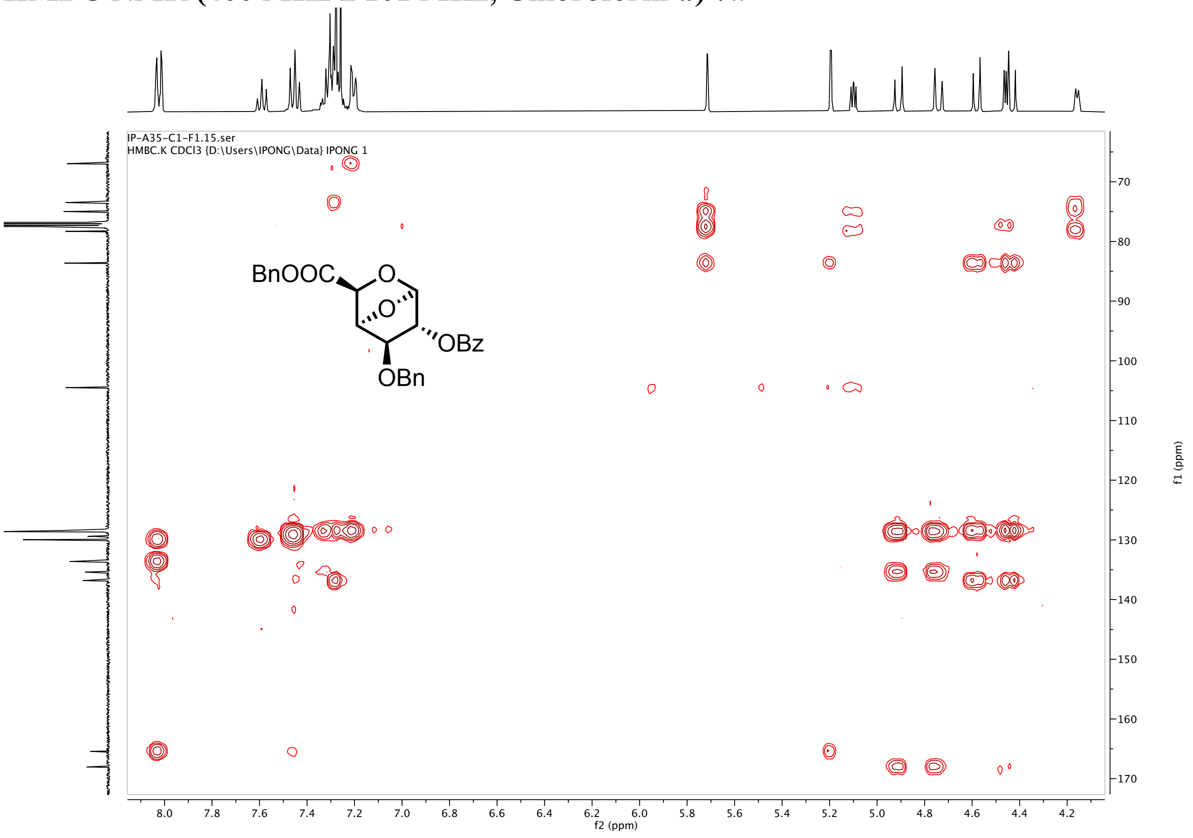

Compound 7b

^{1}H NMR (400 MHz, Chloroform-*d*) 7b

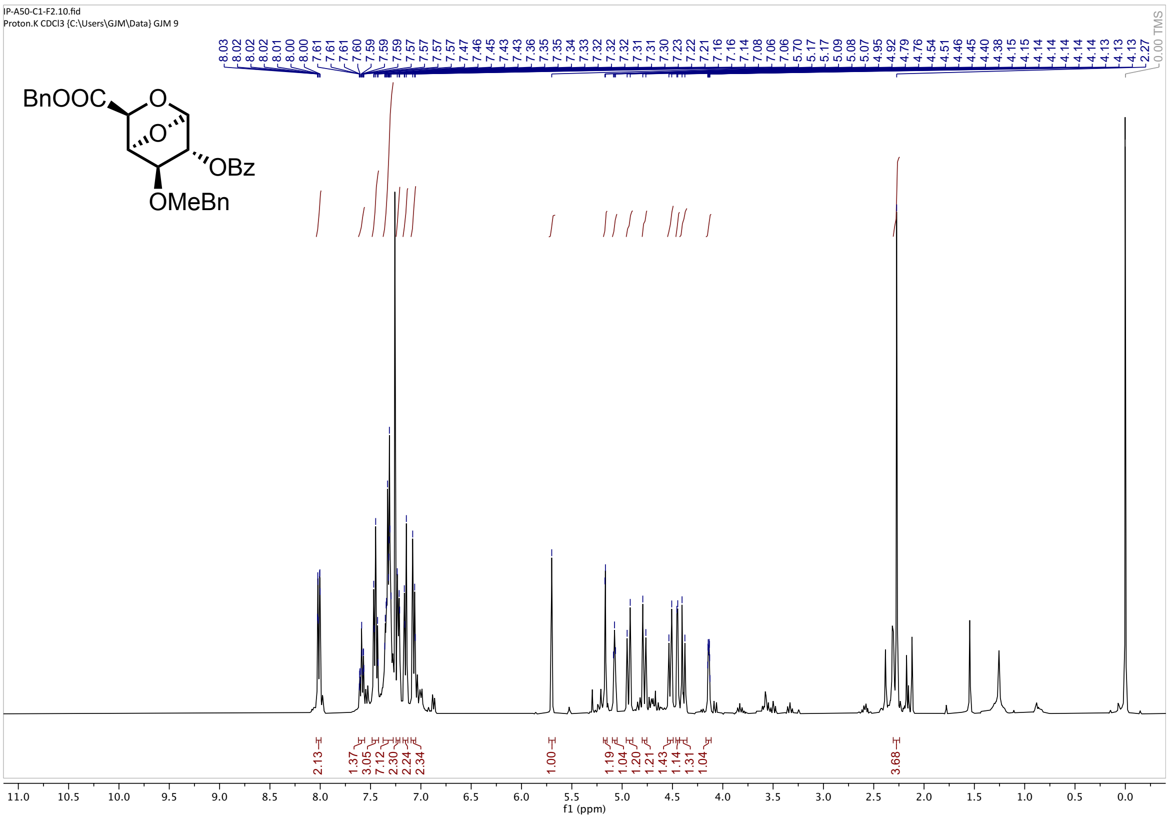

^{13}C{^{1}H} NMR (101 MHz, Chloroform-*d*) 7b

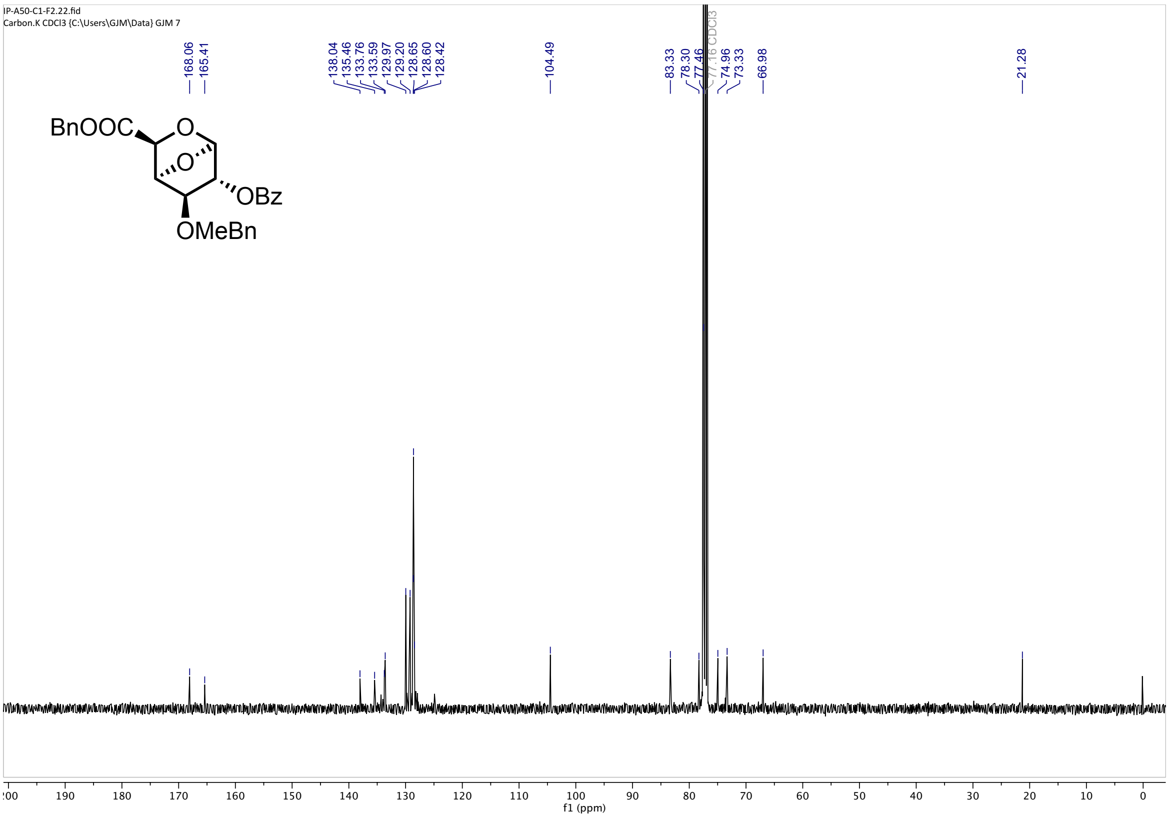

COSY NMR (400 MHz, Chloroform-*d*) 7b

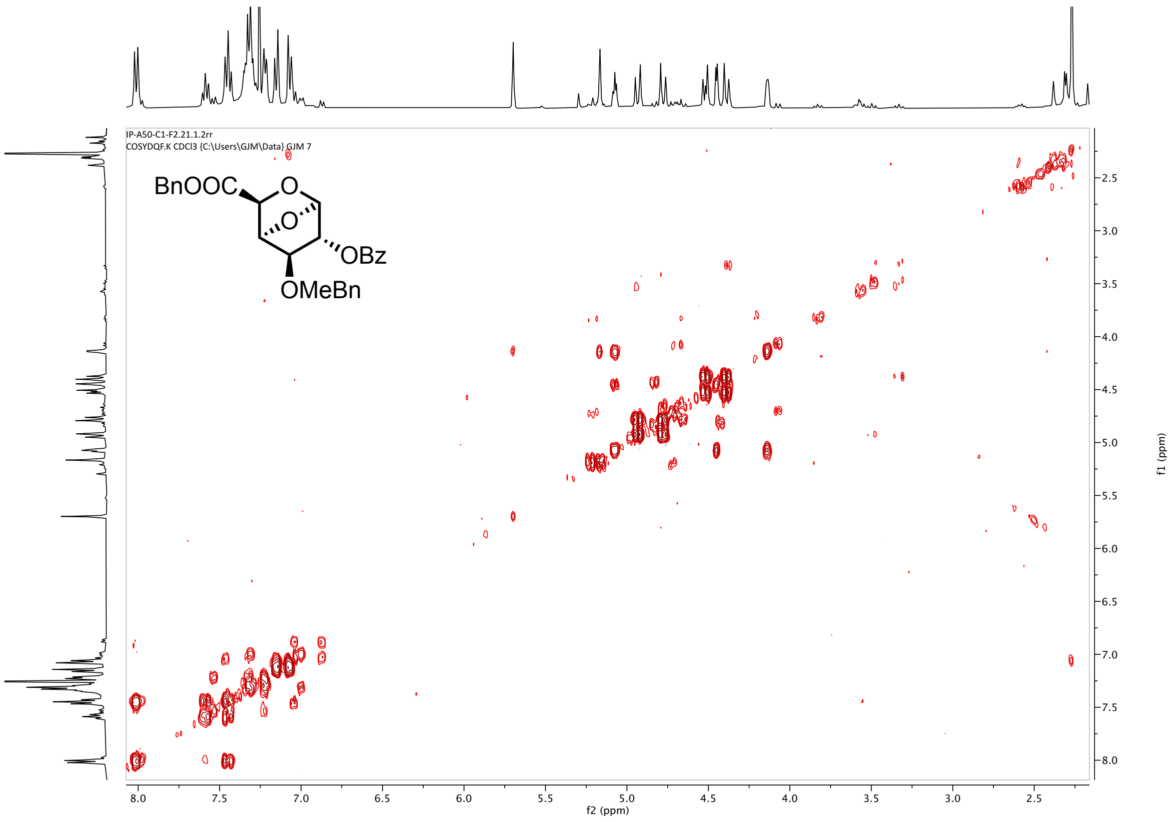

HSQC NMR (400 MHz x 101 MHz, Chloroform-*d*) 7b

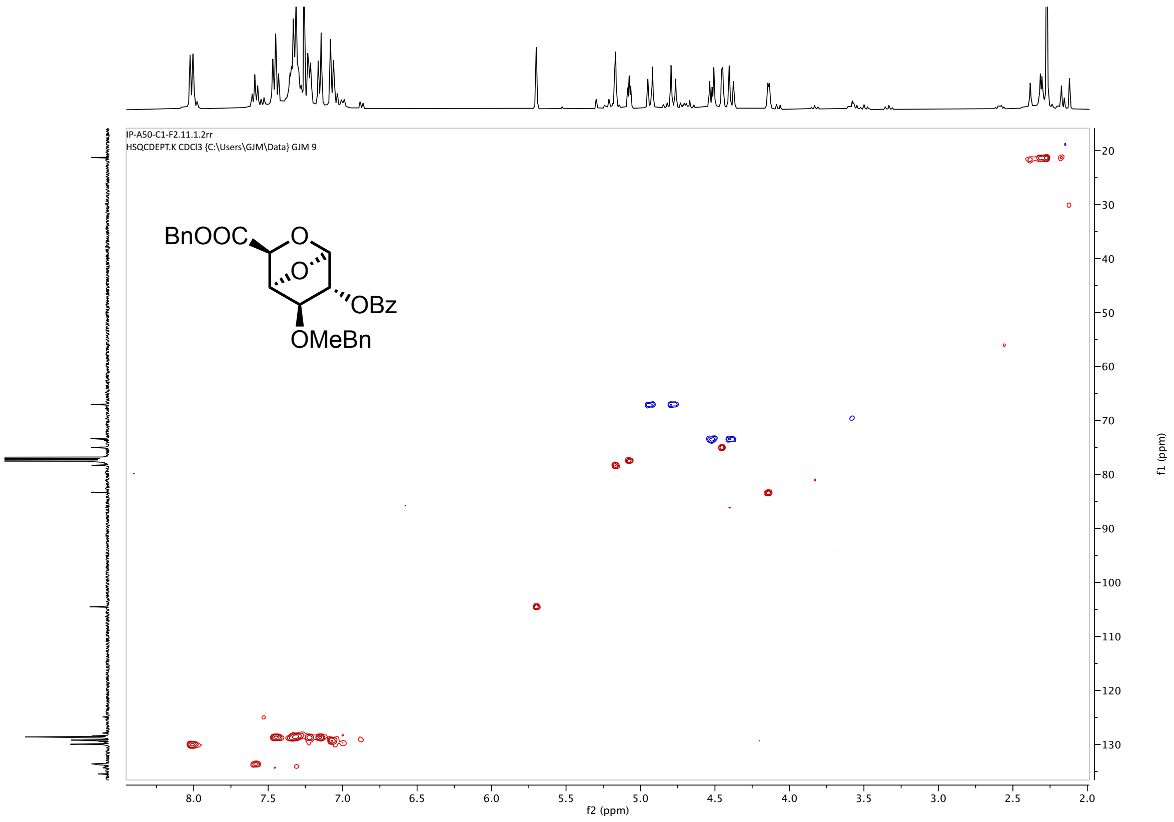

Coupled-HSQC NMR (400 MHz x 101 MHz, Chloroform-*d*) 7b

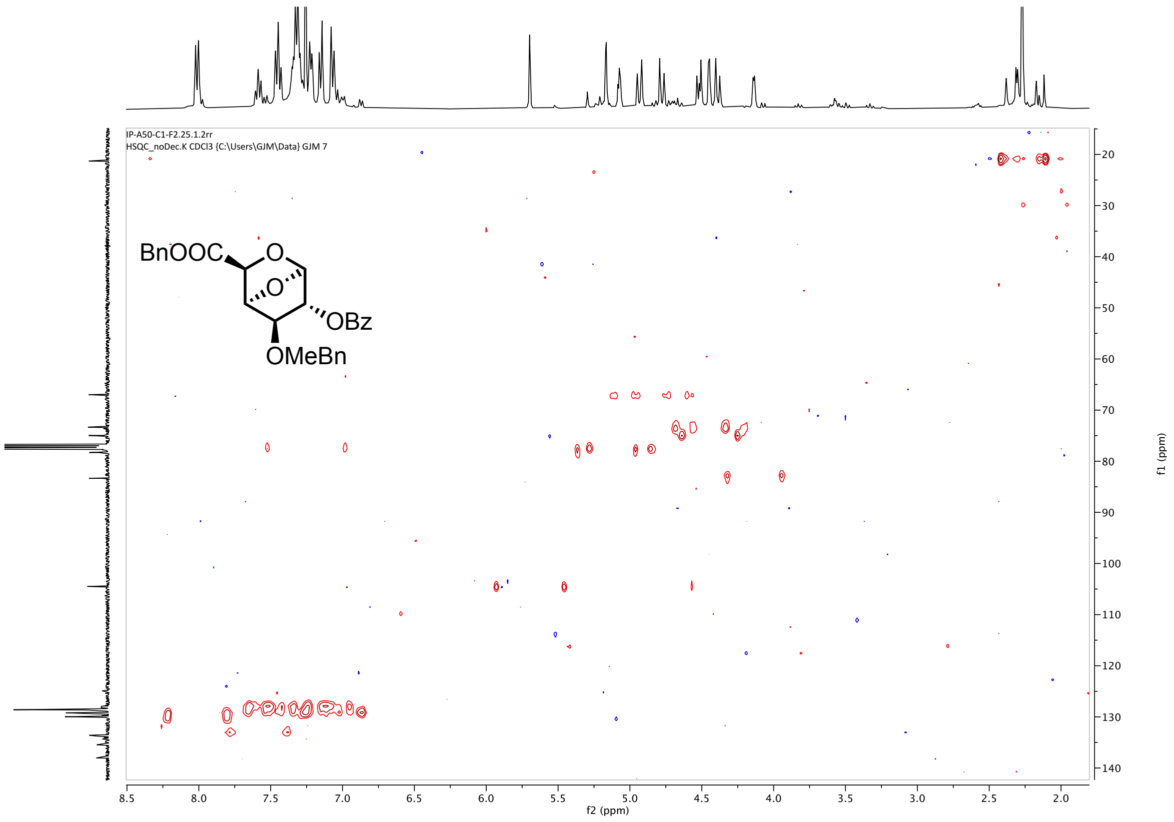

HMBC NMR (400 MHz x 101 MHz, Chloroform-*d*) 7b

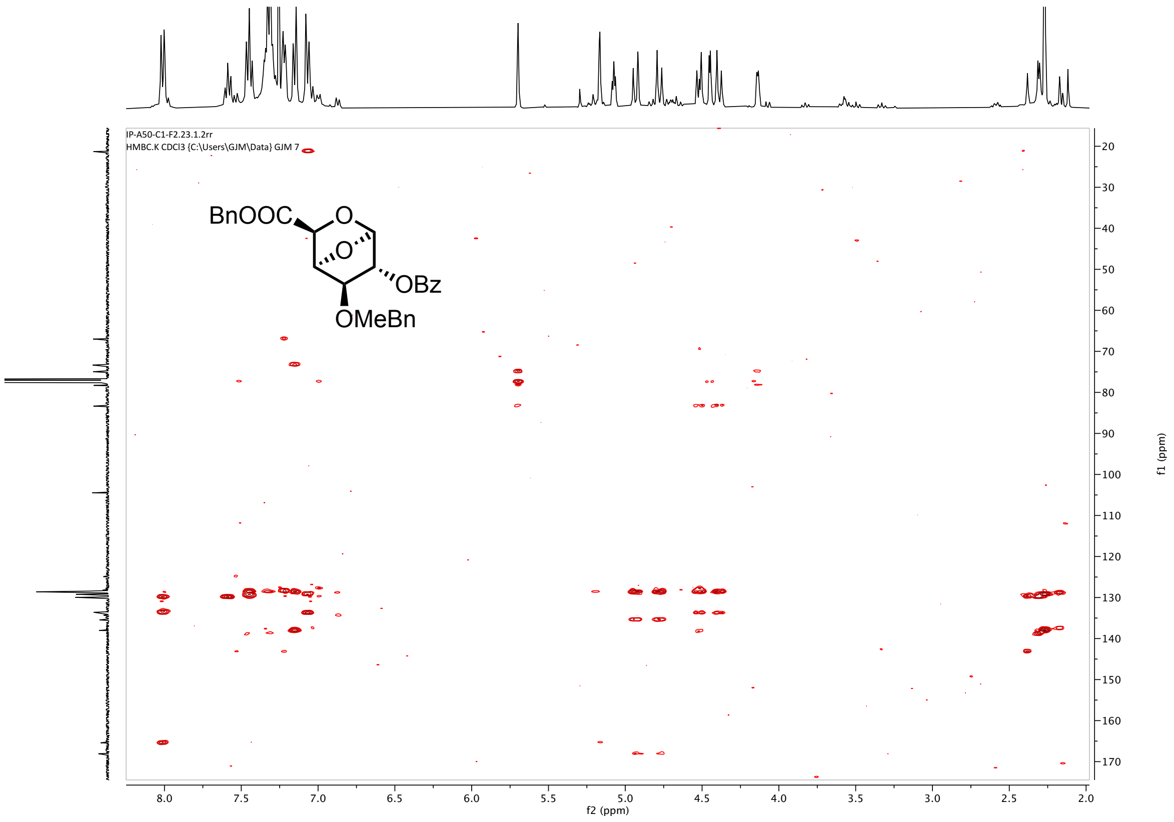

Compound 13a

^{1}H NMR (400 MHz, Chloroform-*d*) 13a

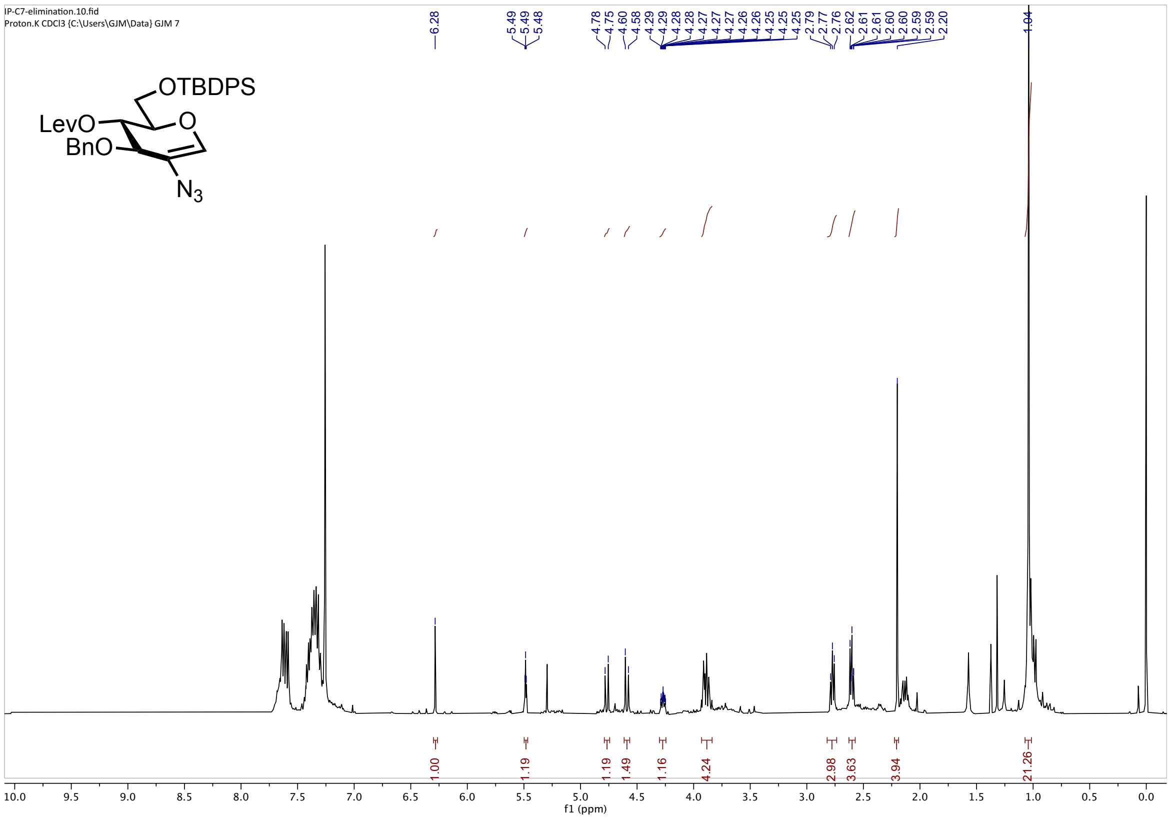

$^{13}C\{^{1}H\}$ NMR (101 MHz, Chloroform-*d*) 13a

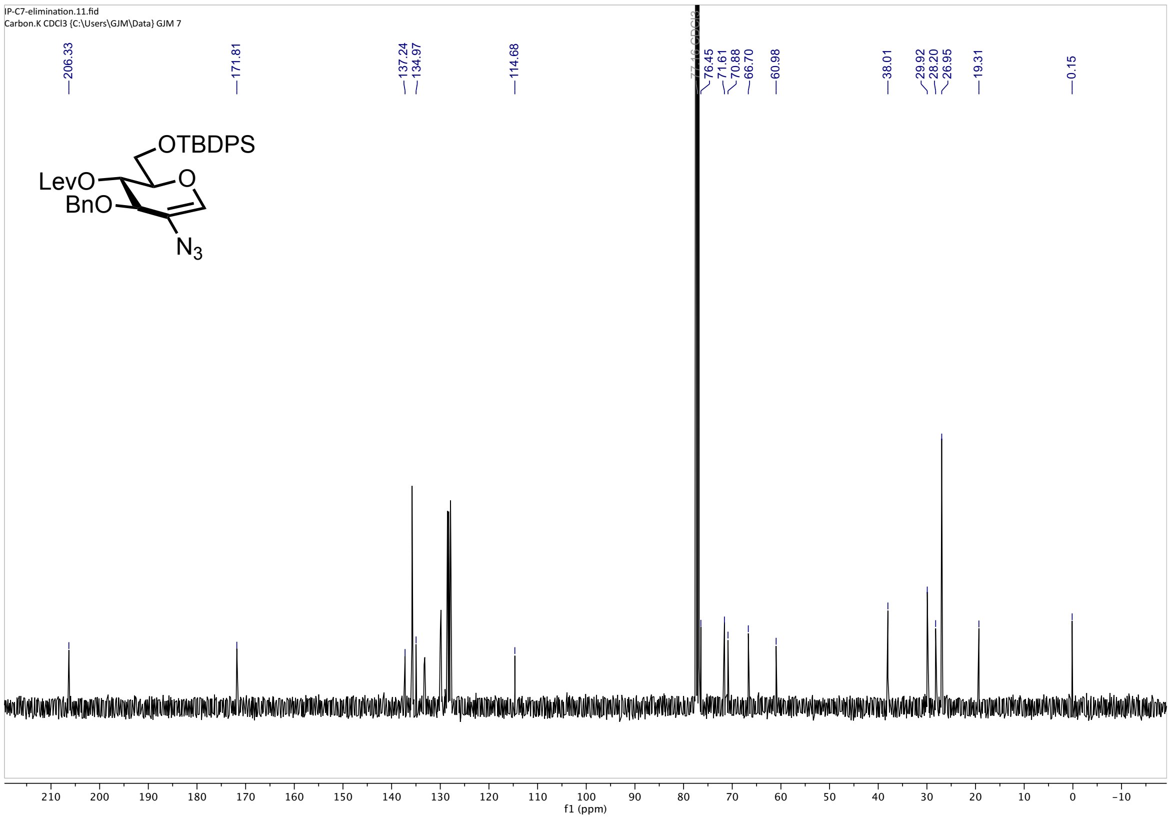

COSY NMR (400 MHz, Chloroform-*d*) 13a

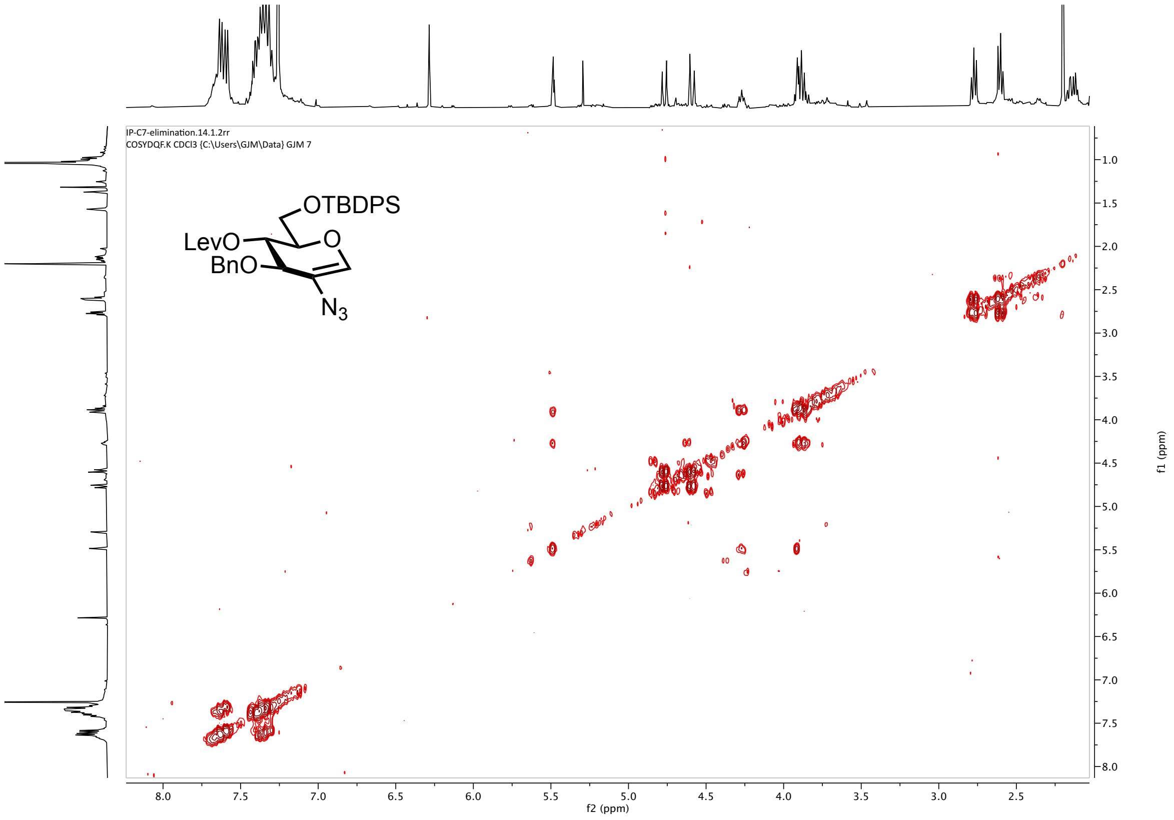

HSQC NMR (400 MHz x 101 MHz, Chloroform-*d*) 13a

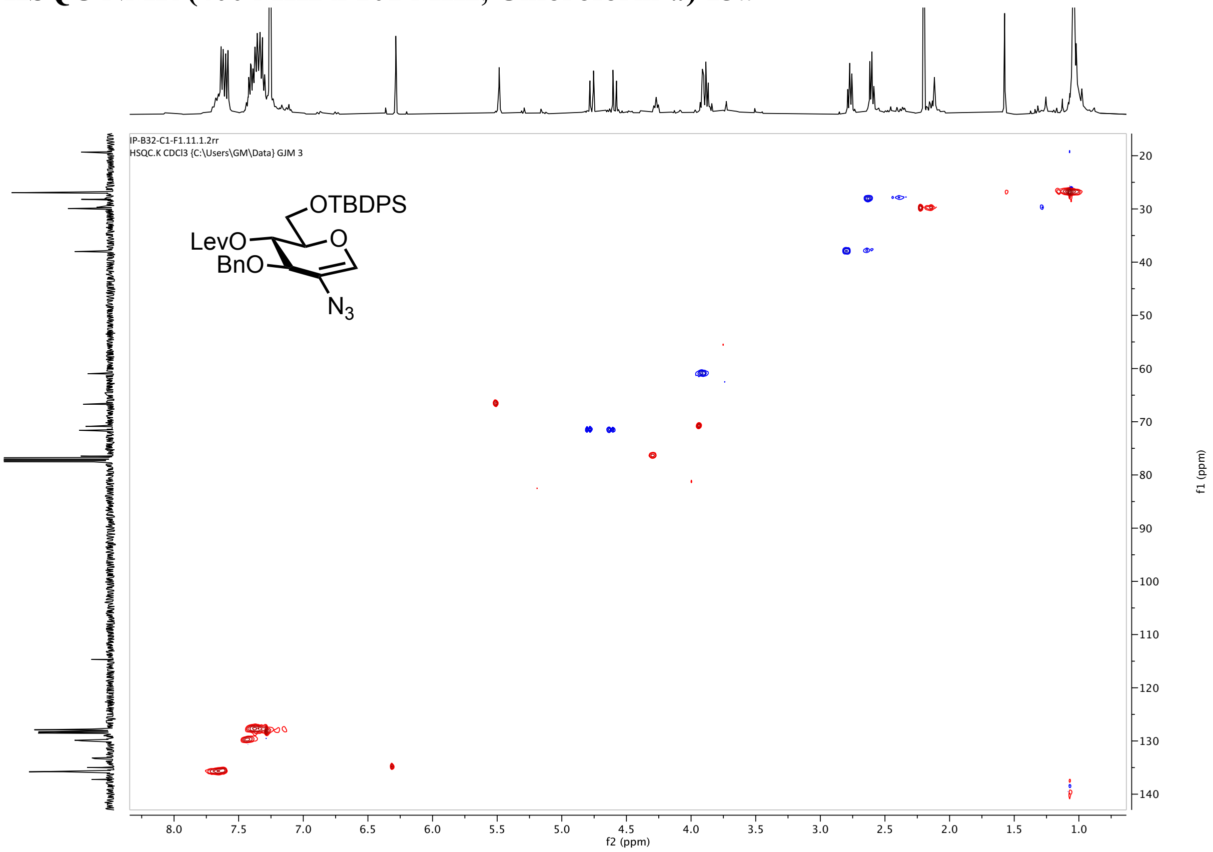

HMBC NMR (400 MHz x 101 MHz, Chloroform-*d*) 13a

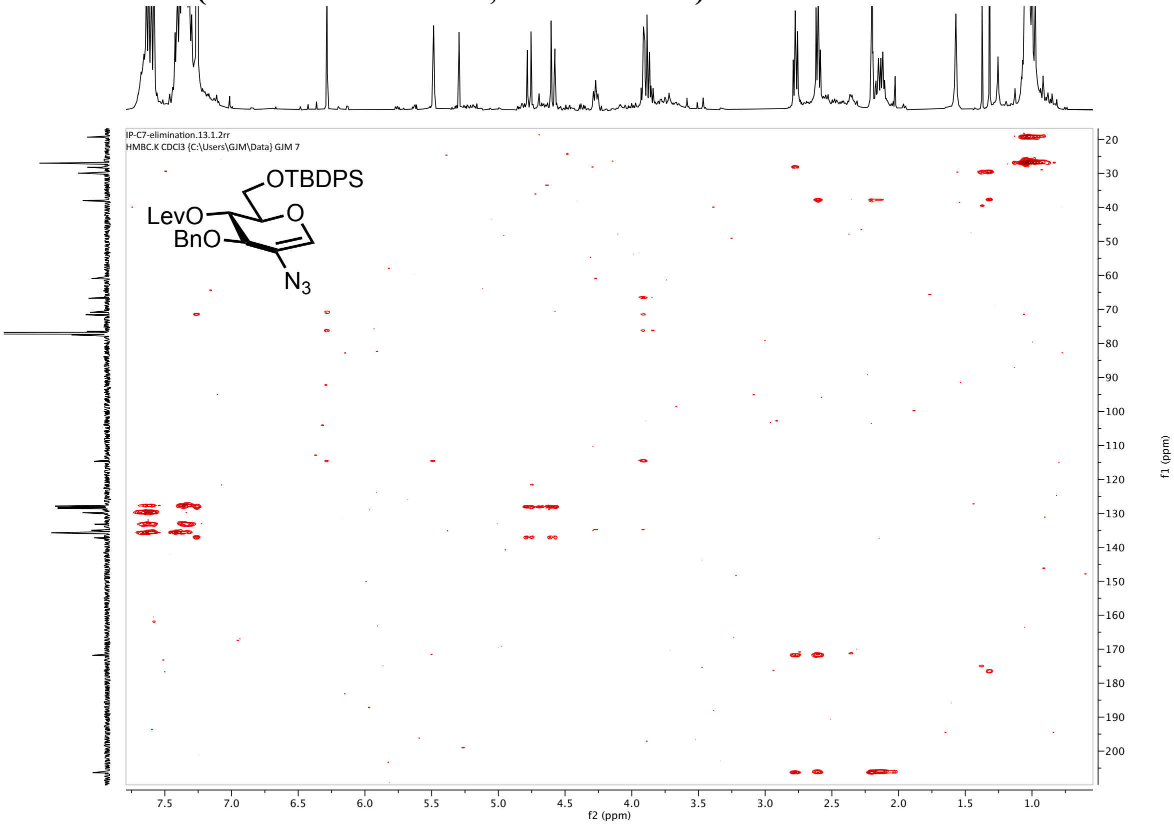

Transformations of Disaccharide Building Block

Compound 16

^{1}H NMR (400 MHz, Chloroform-*d*) 16

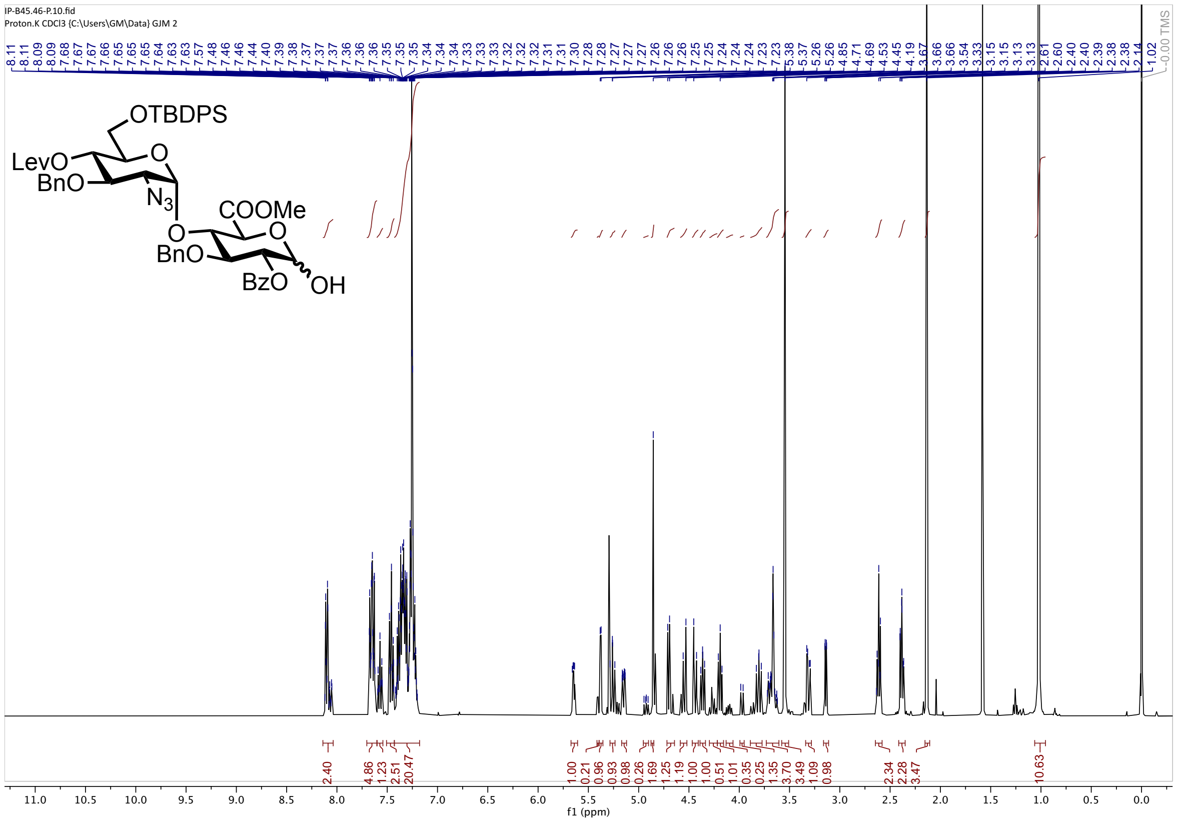

$^{13}C\{^{1}H\}$ NMR (101 MHz, Chloroform-*d*) 16

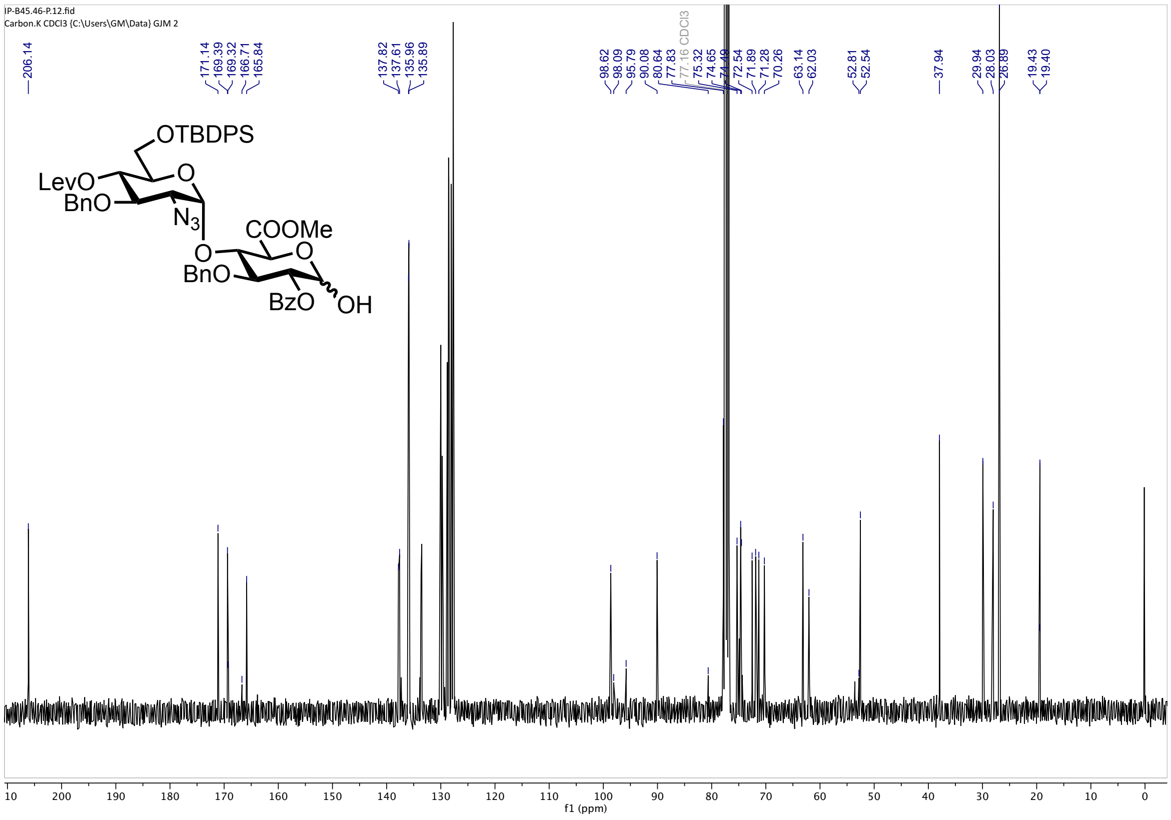

COSY NMR (400 MHz, Chloroform-*d*) 16

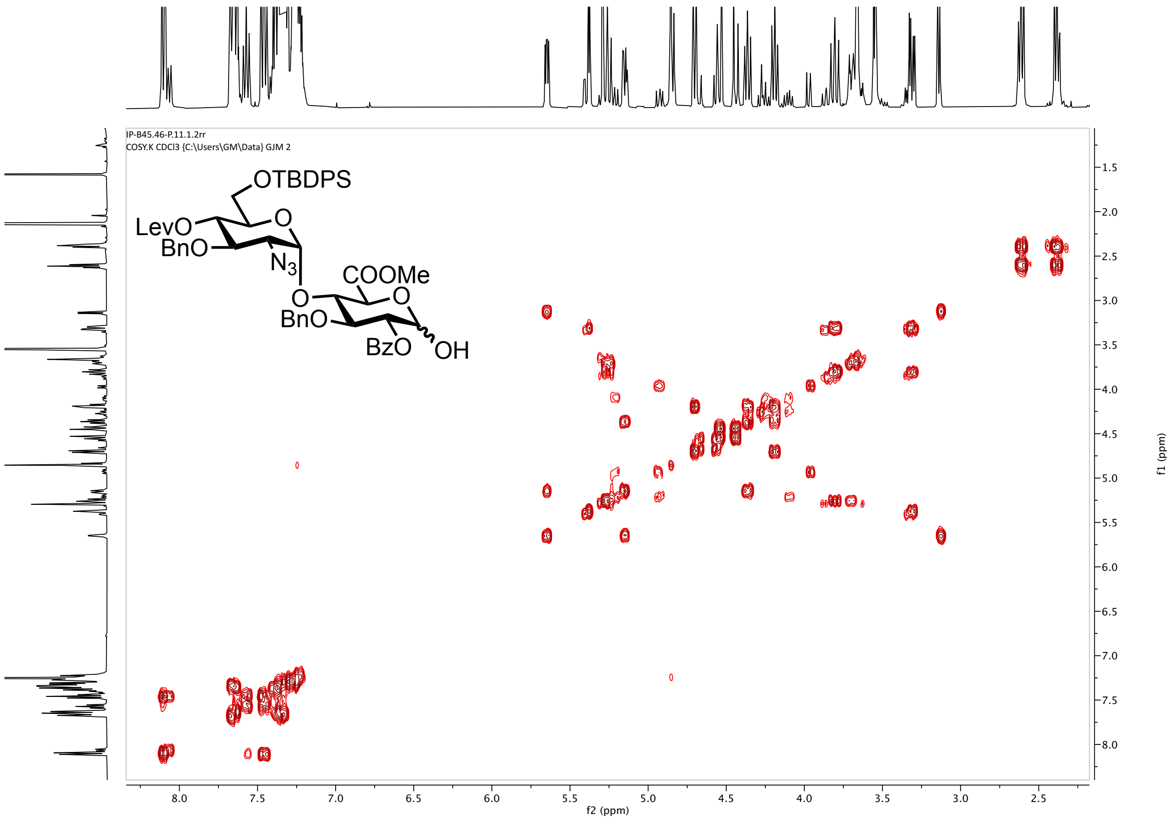

HSQC NMR (400 MHz x 101 MHz, Chloroform-*d*) 16

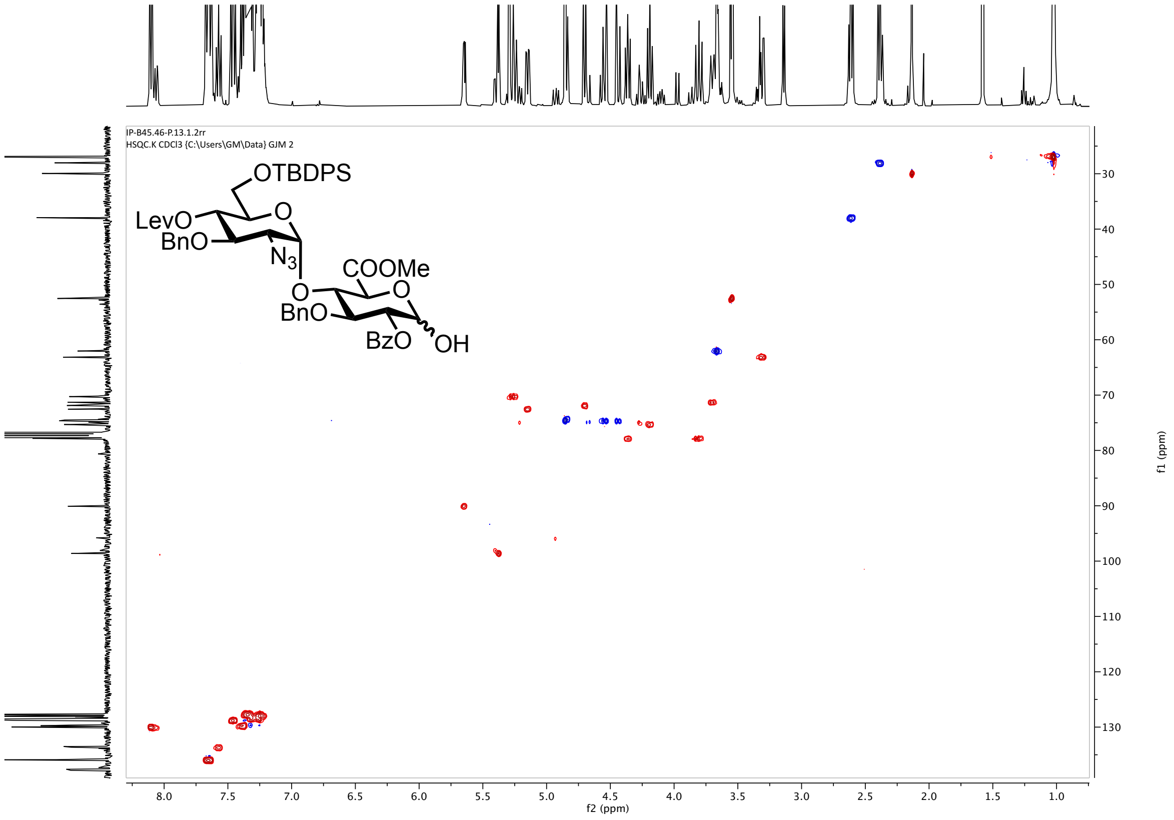

HMBC NMR (400 MHz x 101 MHz, Chloroform-*d*) 16

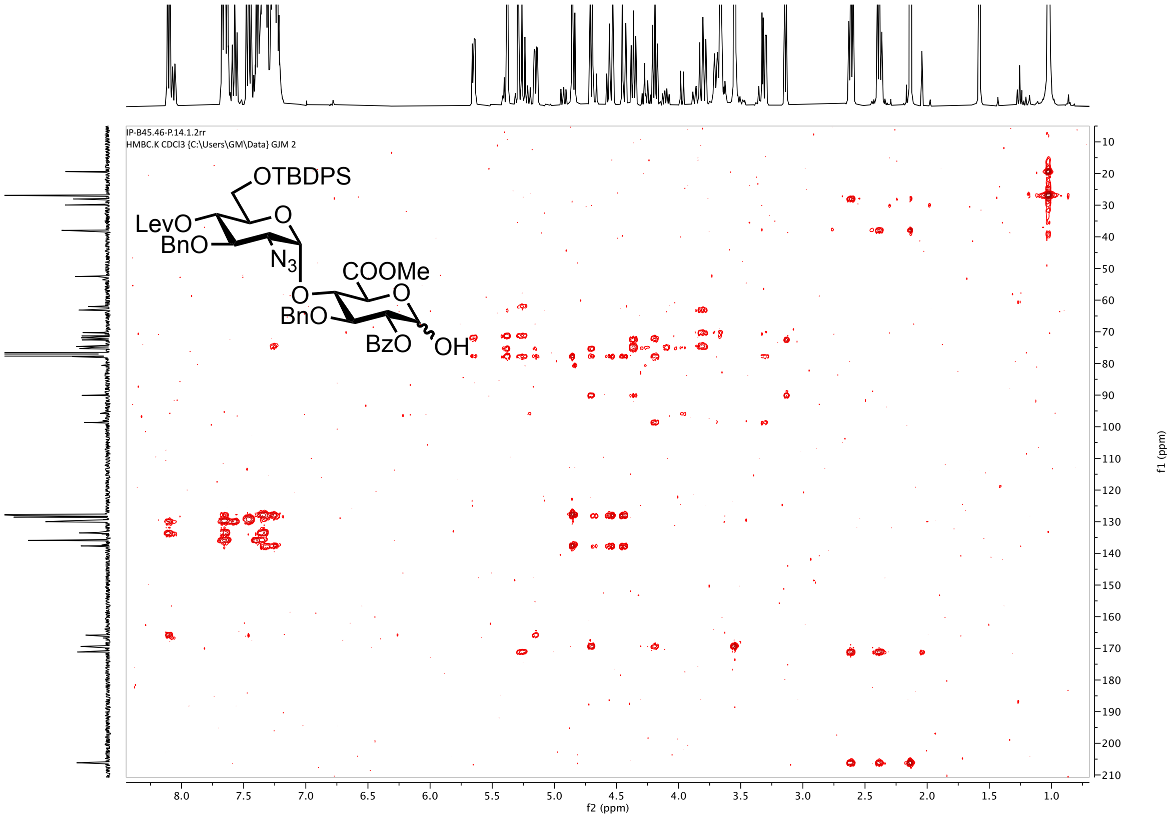

Compound 17

^{1}H NMR (400 MHz, Chloroform-*d*) 17

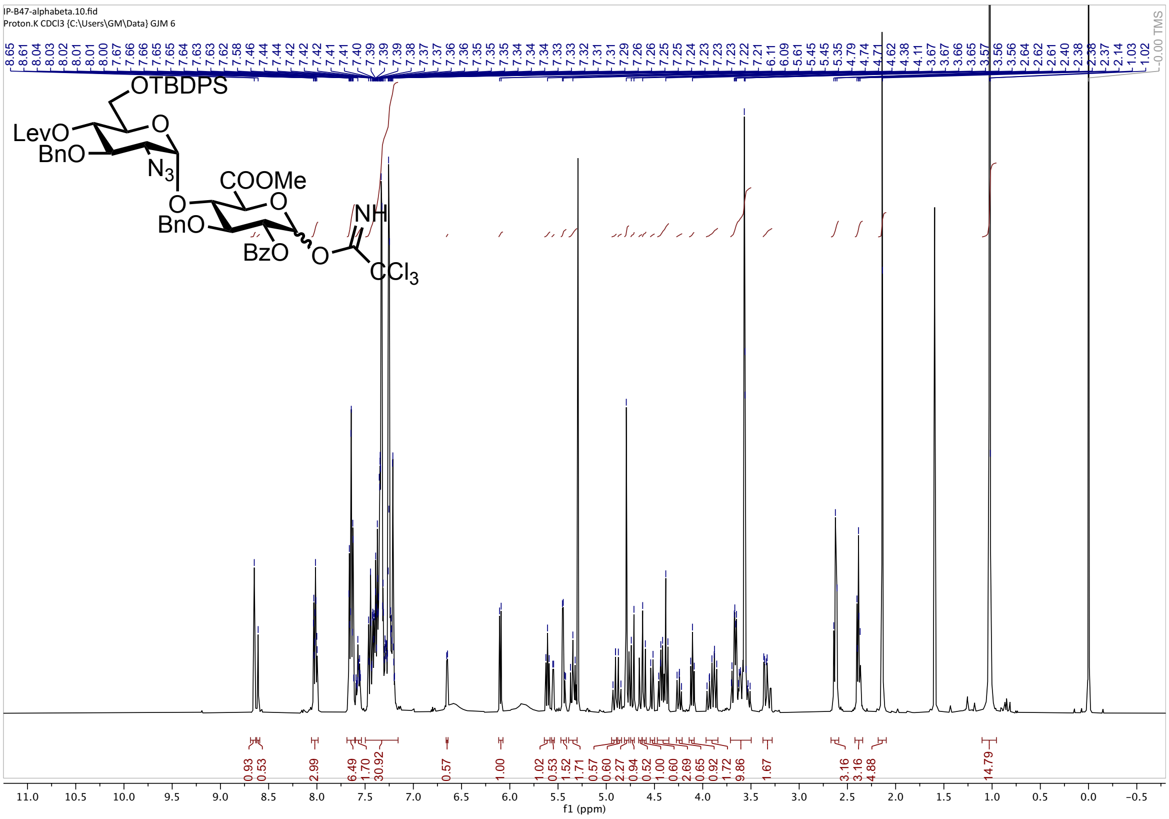

^{13}C{^{1}H} NMR (101 MHz, Chloroform-*d*) 17

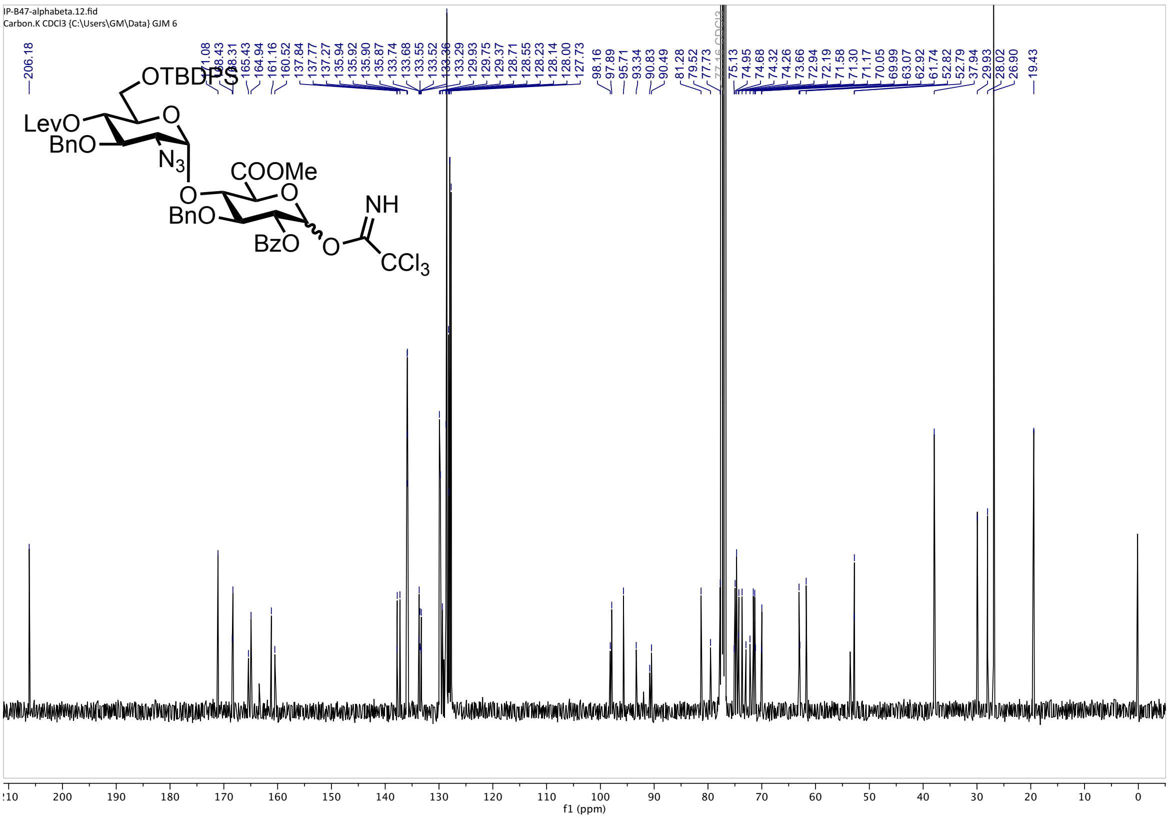

COSY NMR (400 MHz, Chloroform-*d*) 17

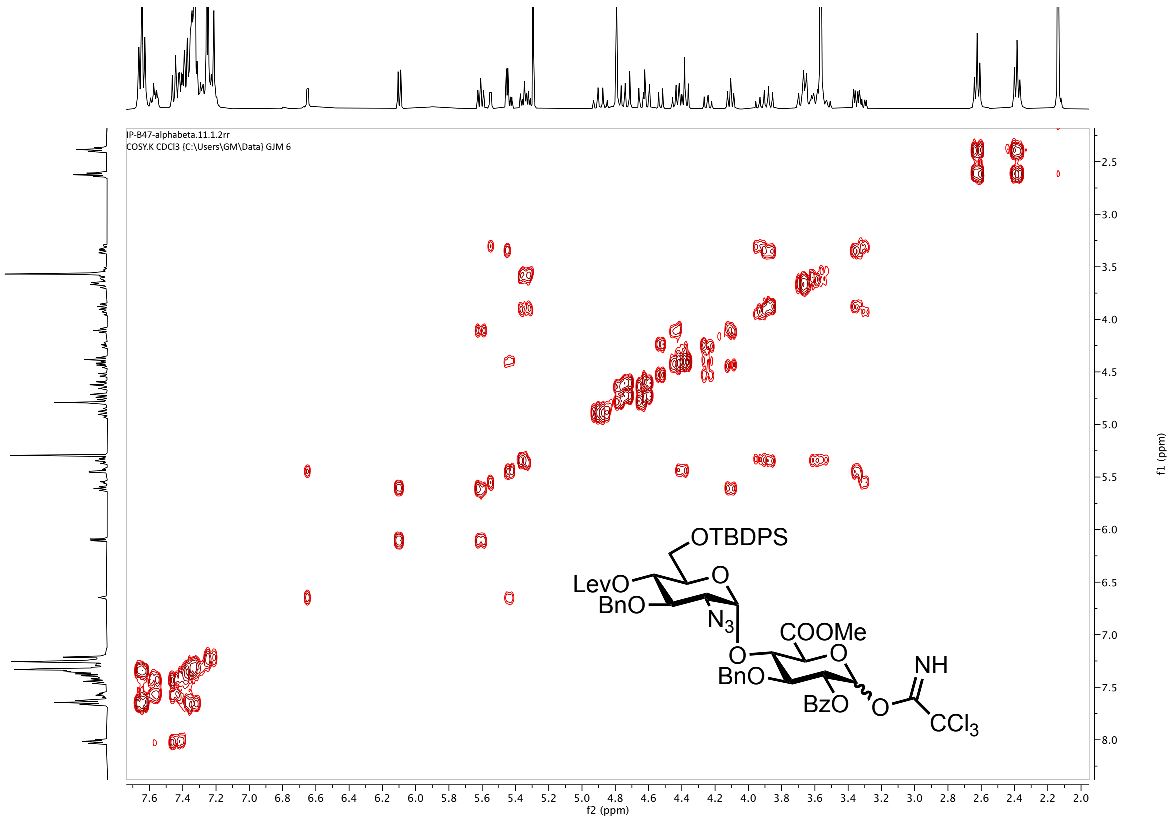

HSQC NMR (400 MHz x 101 MHz, Chloroform-*d*) 17

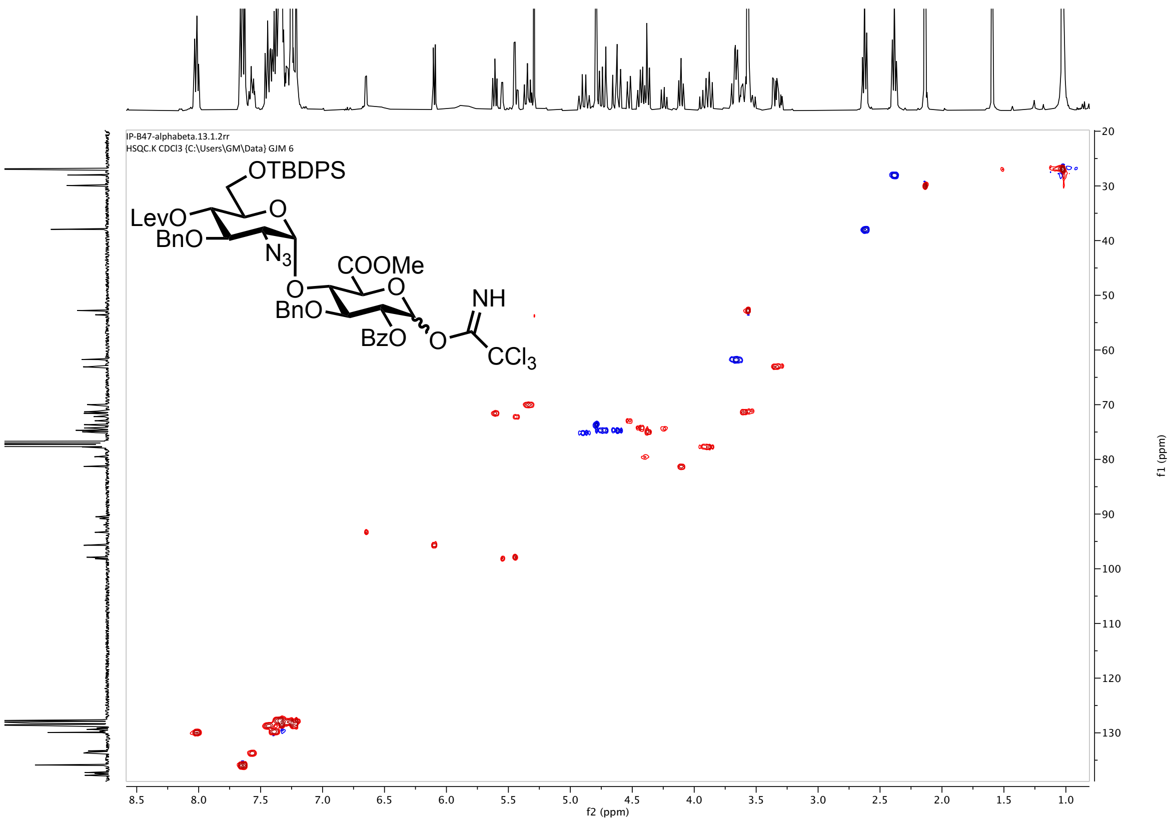

HMBC NMR (400 MHz x 101 MHz, Chloroform-*d*) 17

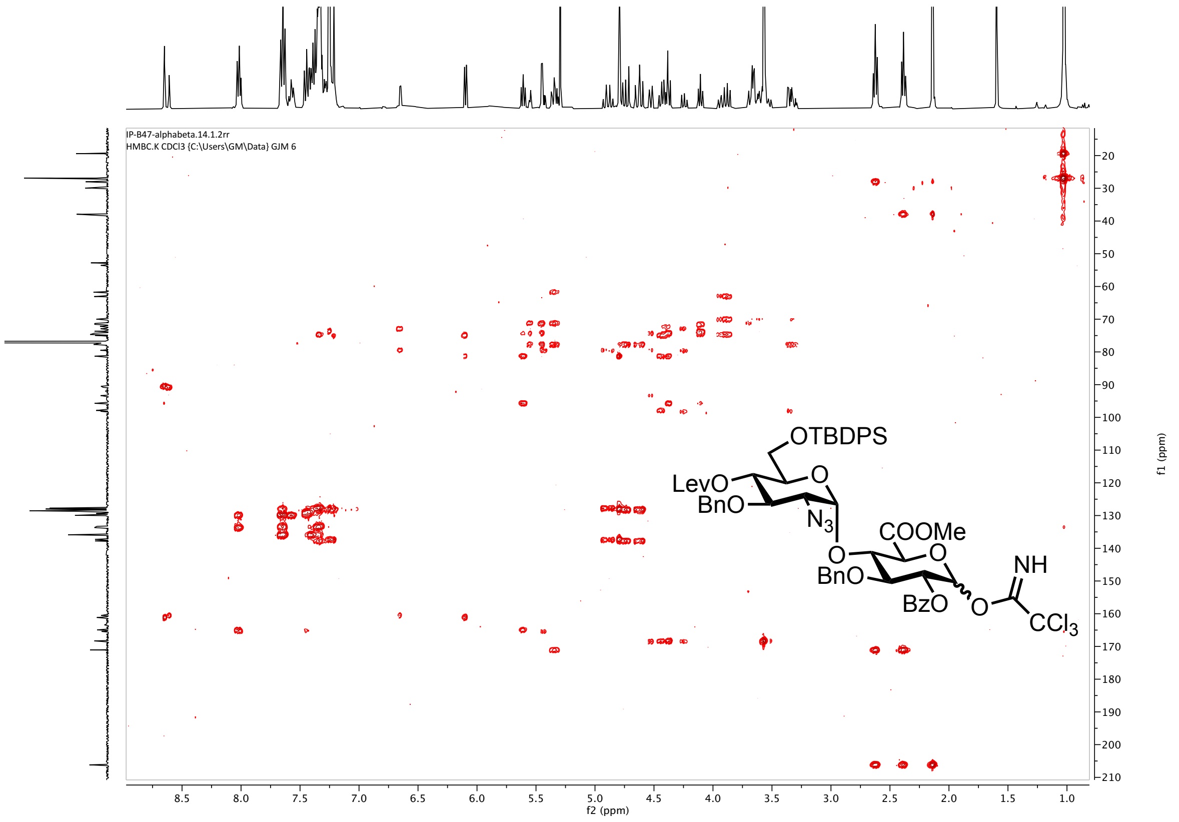

Compound 18

^{1}H NMR (400 MHz, Chloroform-*d*) 18

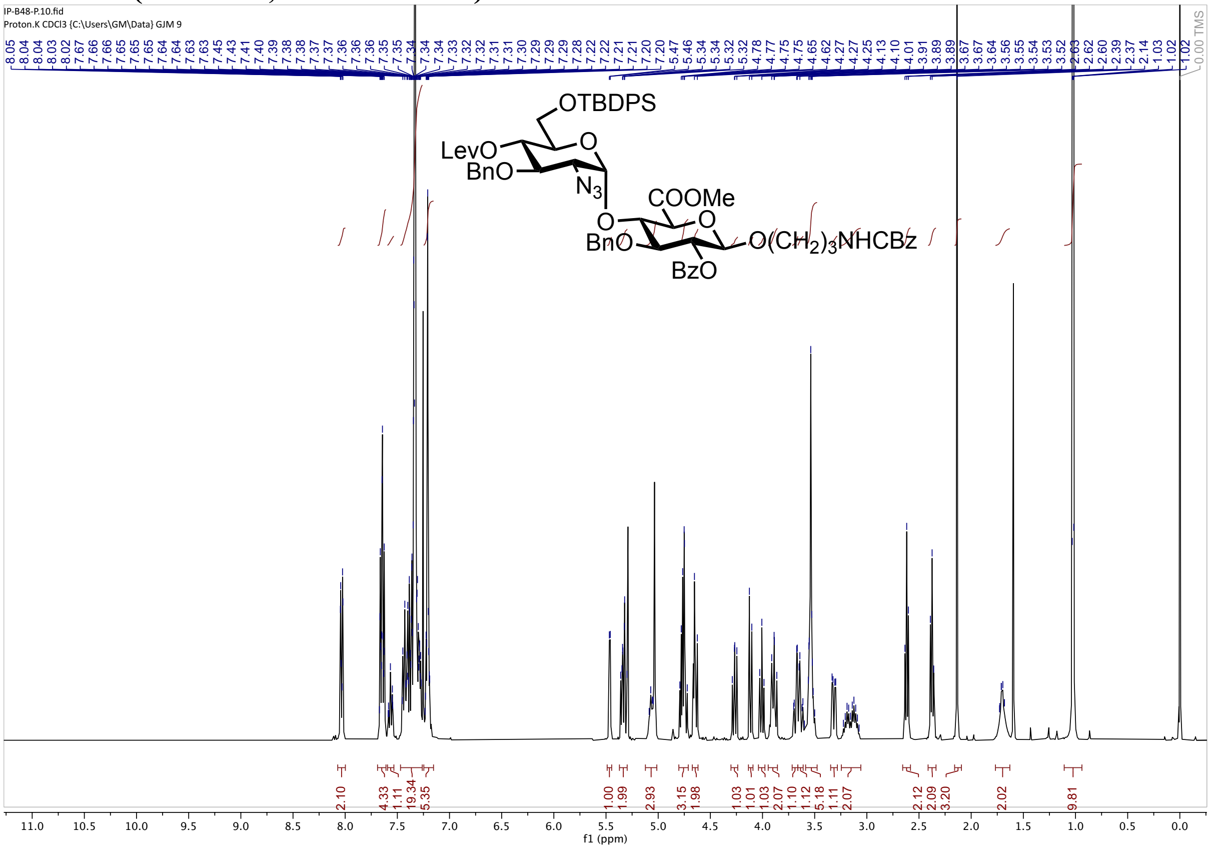

$^{13}C\{^{1}H\}$ NMR (101 MHz, Chloroform-*d*) 18

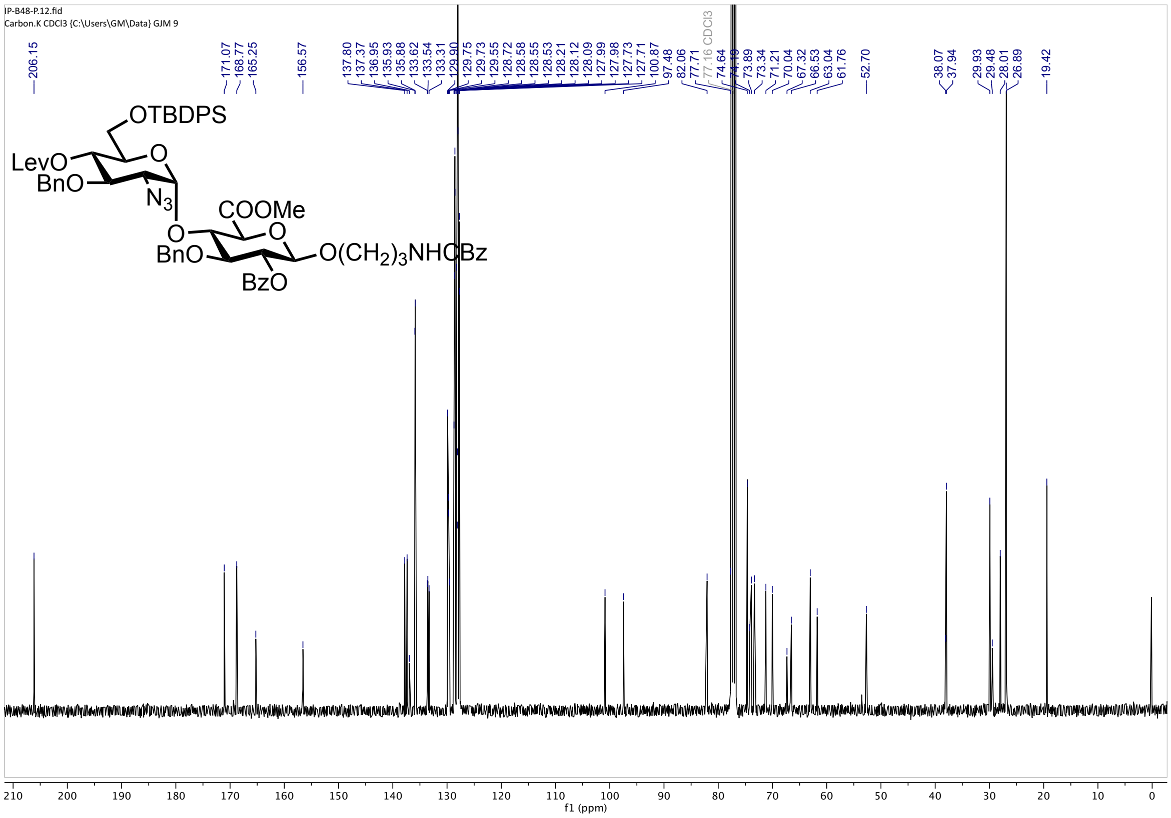

COSY NMR (400 MHz, Chloroform-*d*) 18

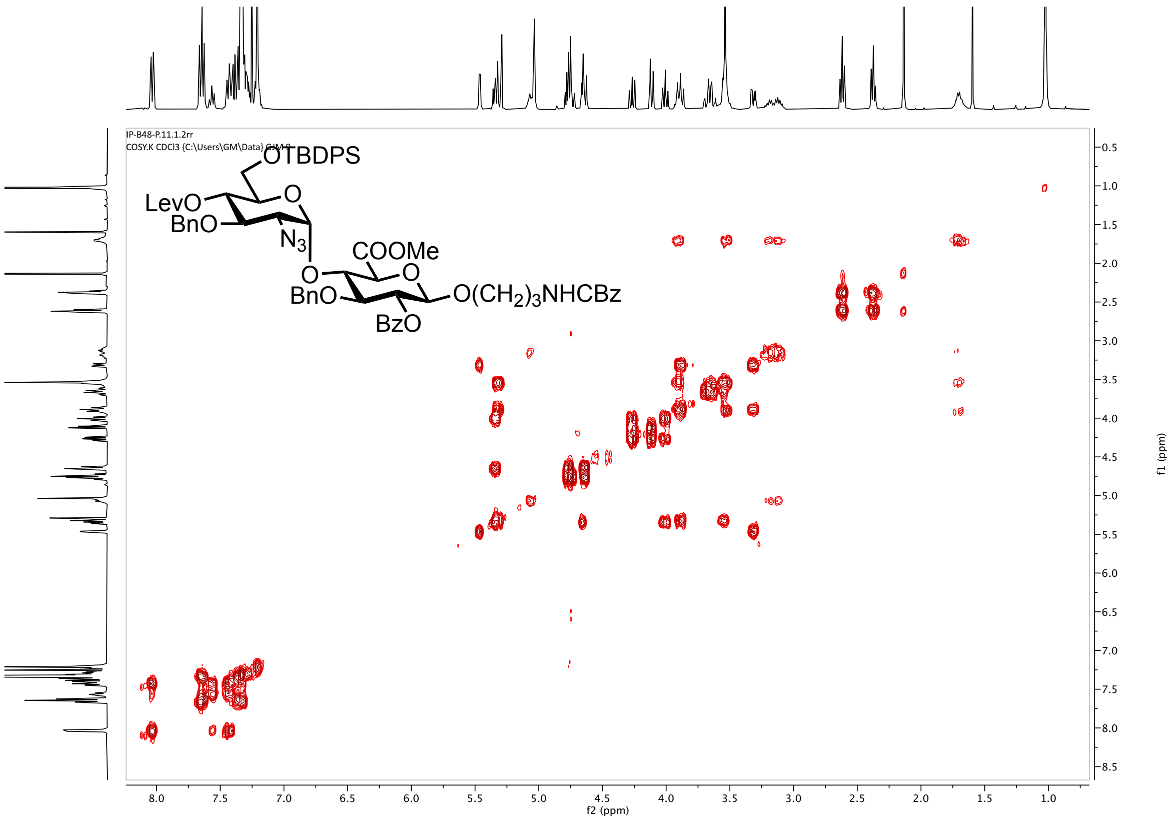

HSQC NMR (400 MHz x 101 MHz, Chloroform-*d*) 18

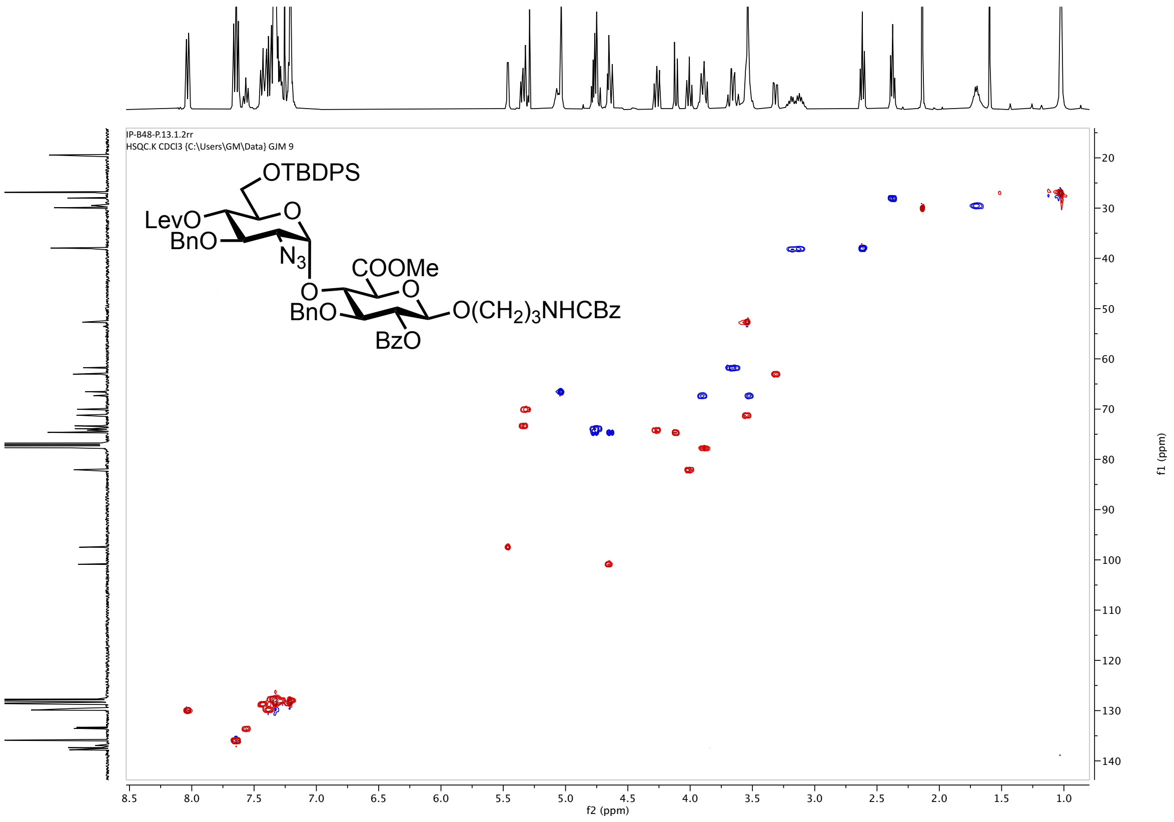

HMBC NMR (400 MHz x 101 MHz, Chloroform-*d*) 18

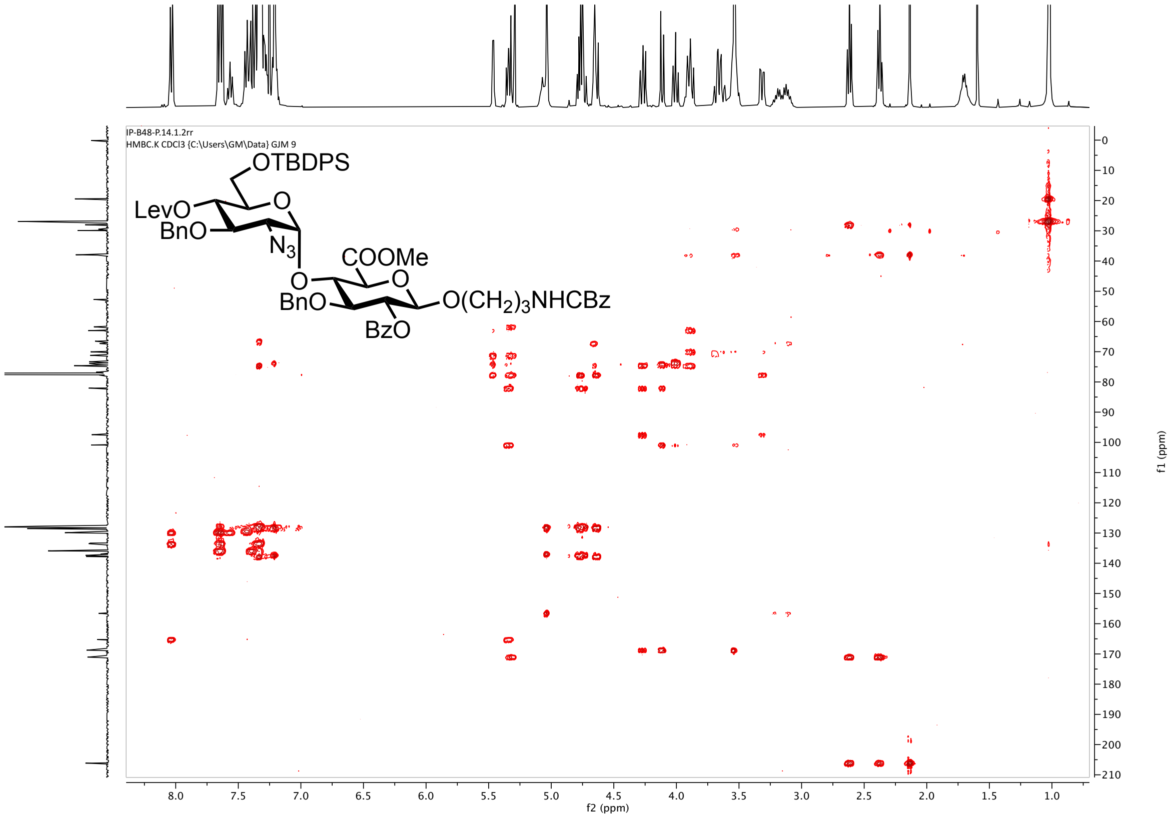

Compound 19

^{1}H NMR (400 MHz, Chloroform-*d*) 19

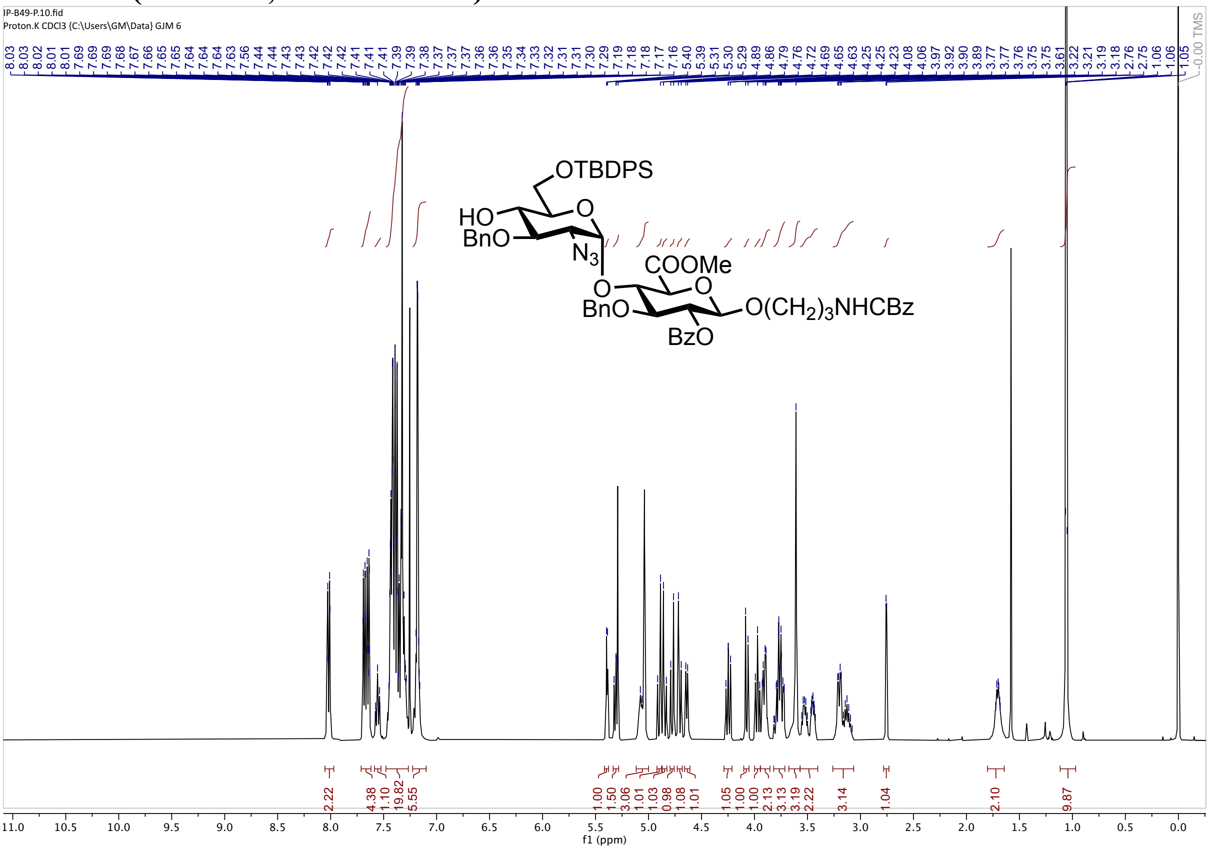

^{13}C{^{1}H} NMR (101 MHz, Chloroform-*d*) 19

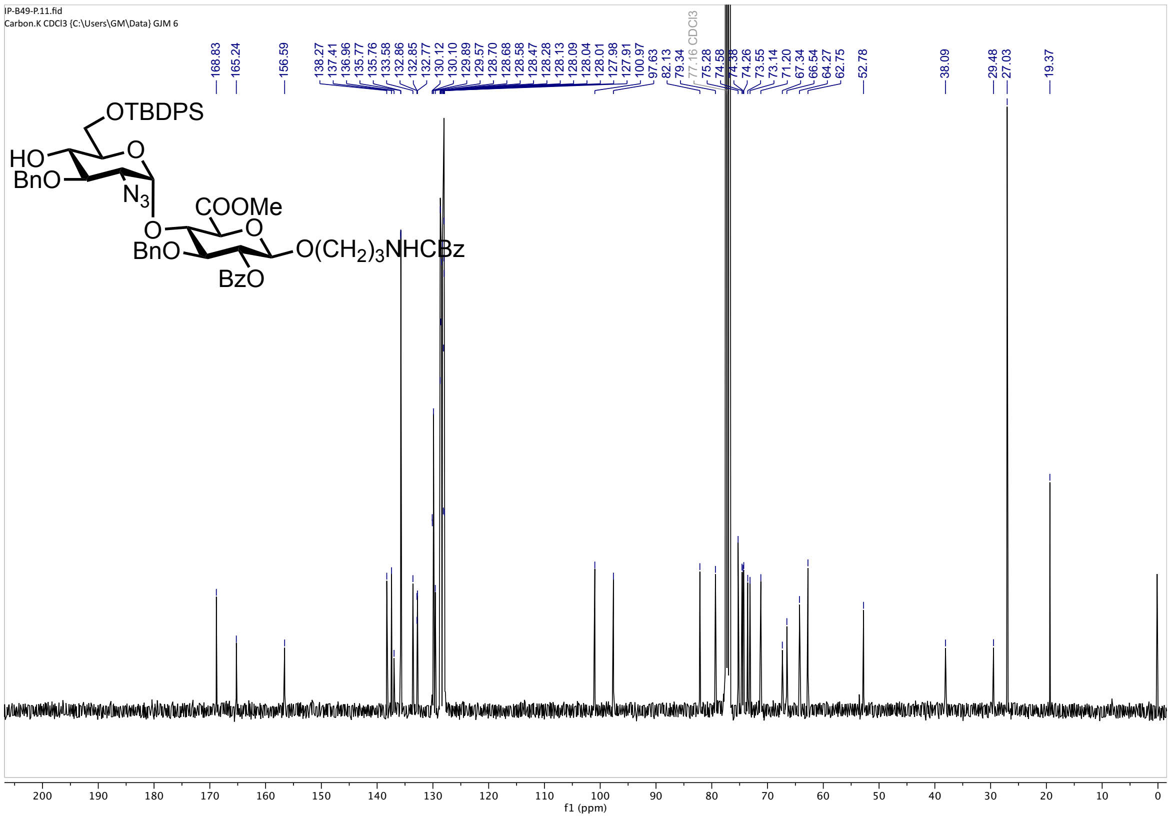

COSY NMR (400 MHz, Chloroform-*d*) 19

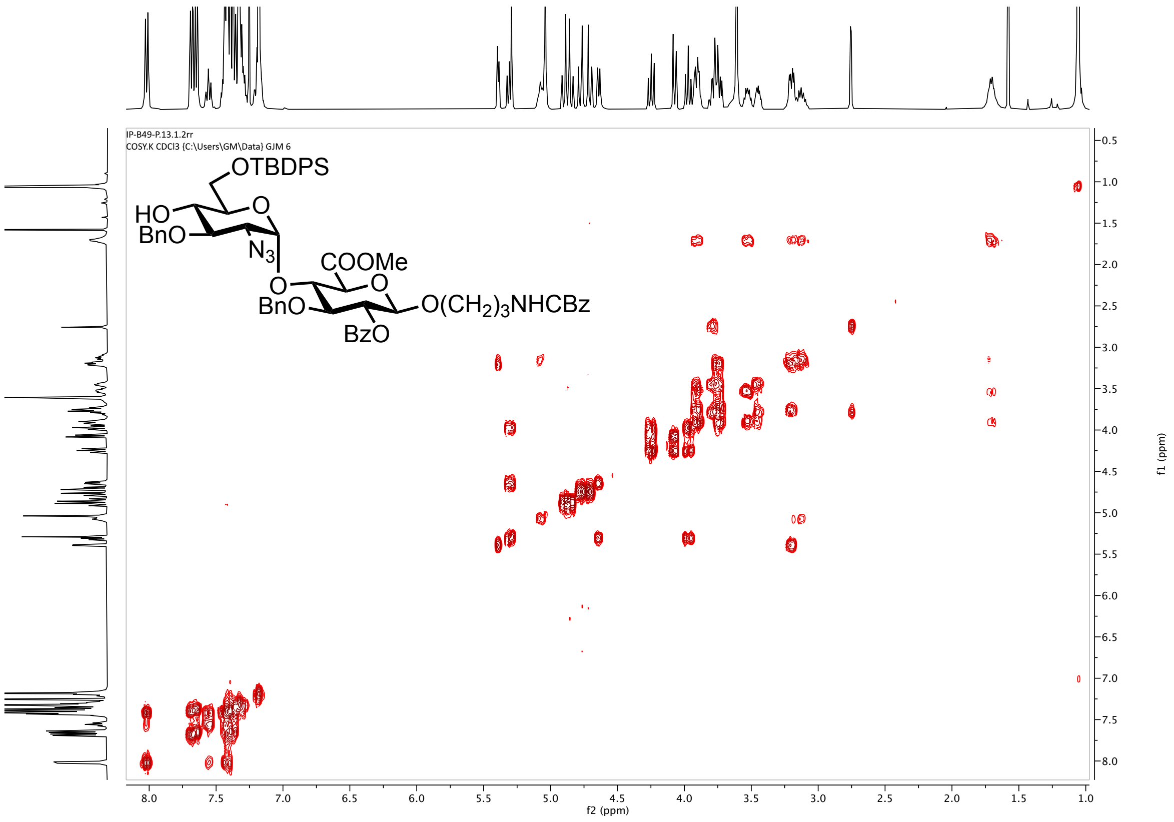

HSQC NMR (400 MHz x 101 MHz, Chloroform-*d*) 19

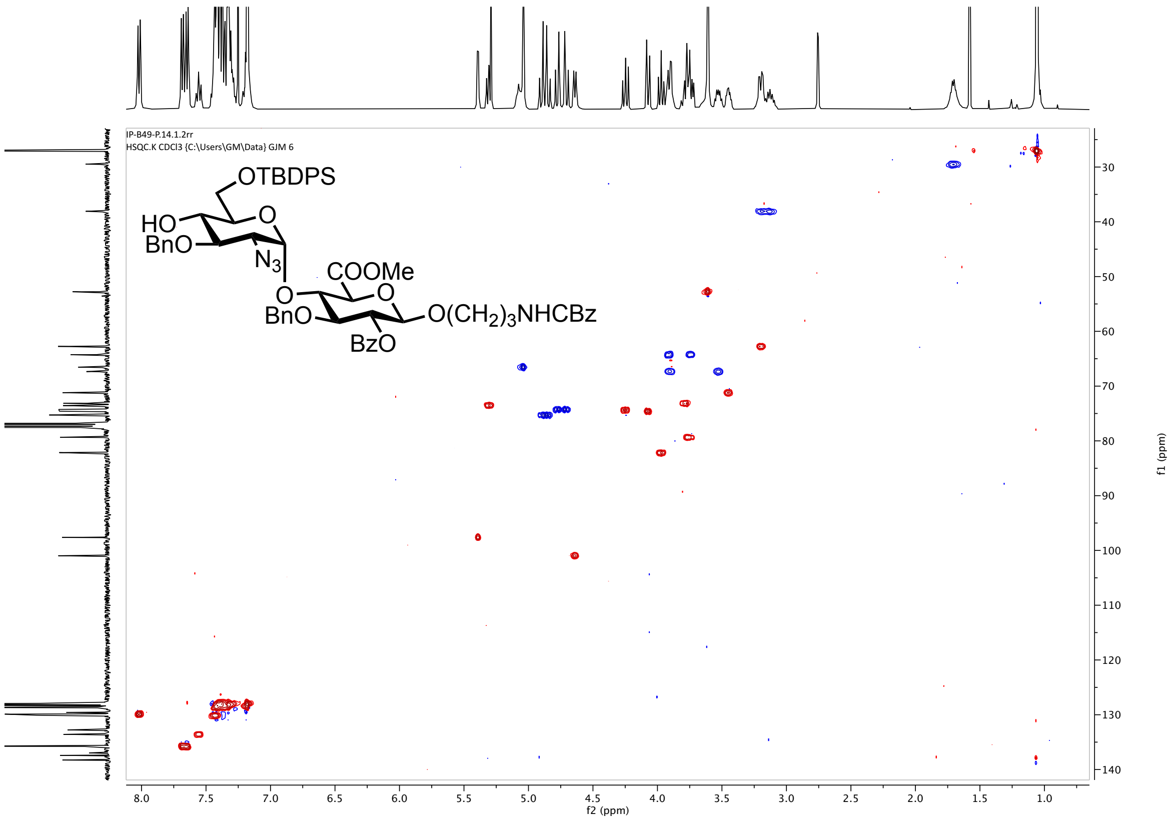

HMBC NMR (400 MHz x 101 MHz, Chloroform-*d*) 19

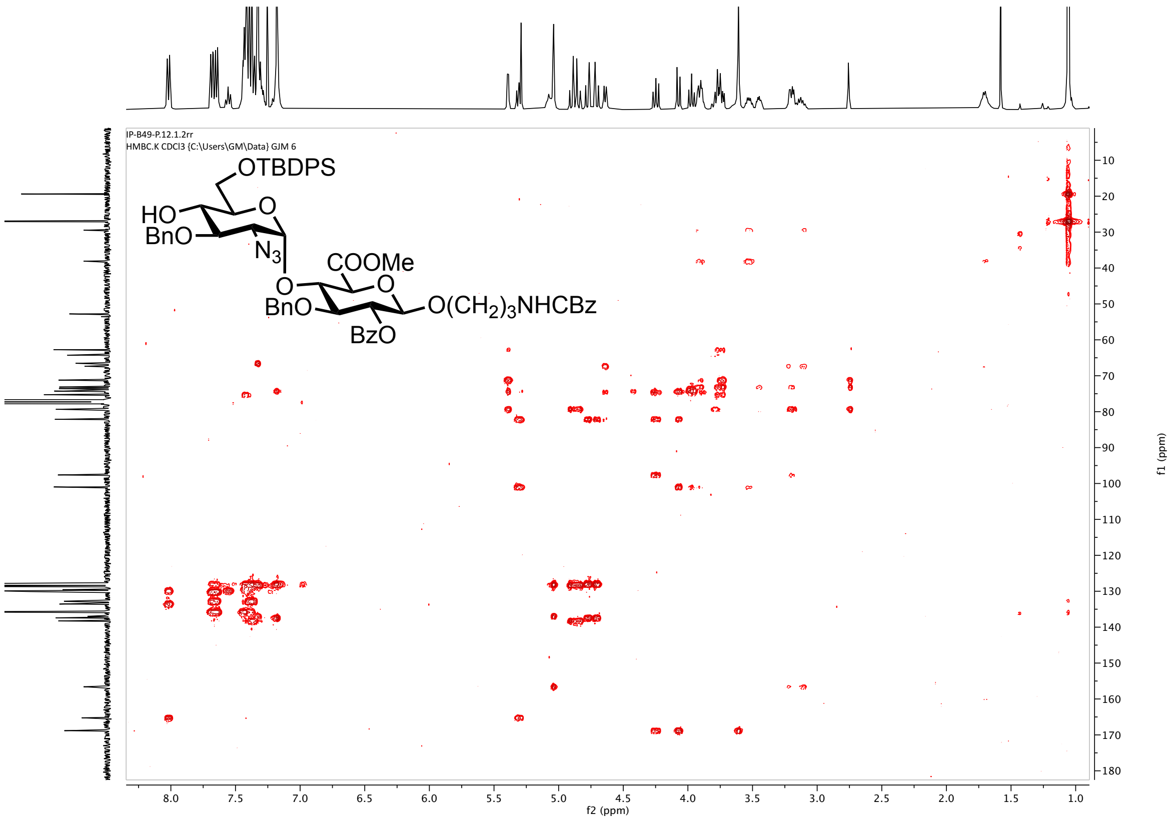

Compound 20

^{1}H NMR (400 MHz, Chloroform-*d*) 20

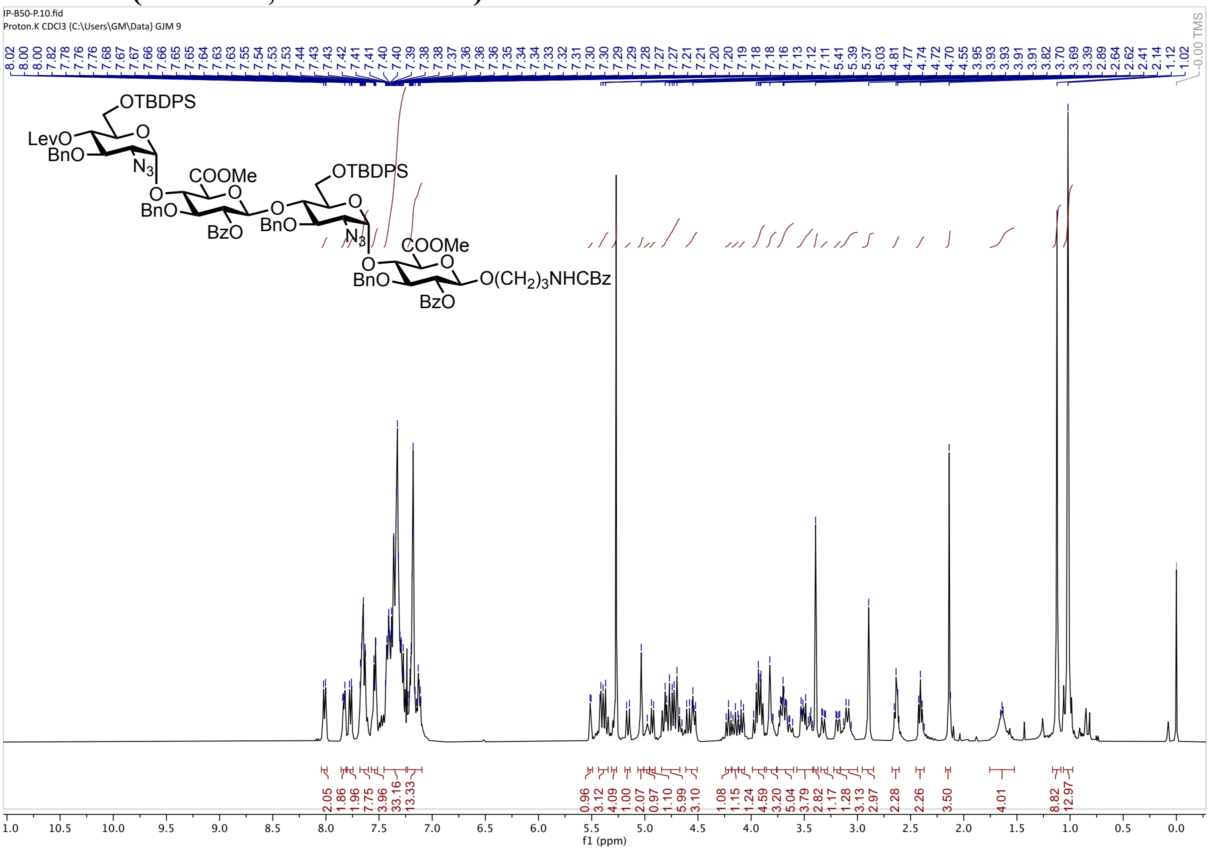

$^{13}C\{^{1}H\}$ NMR (101 MHz, Chloroform-*d*) 20

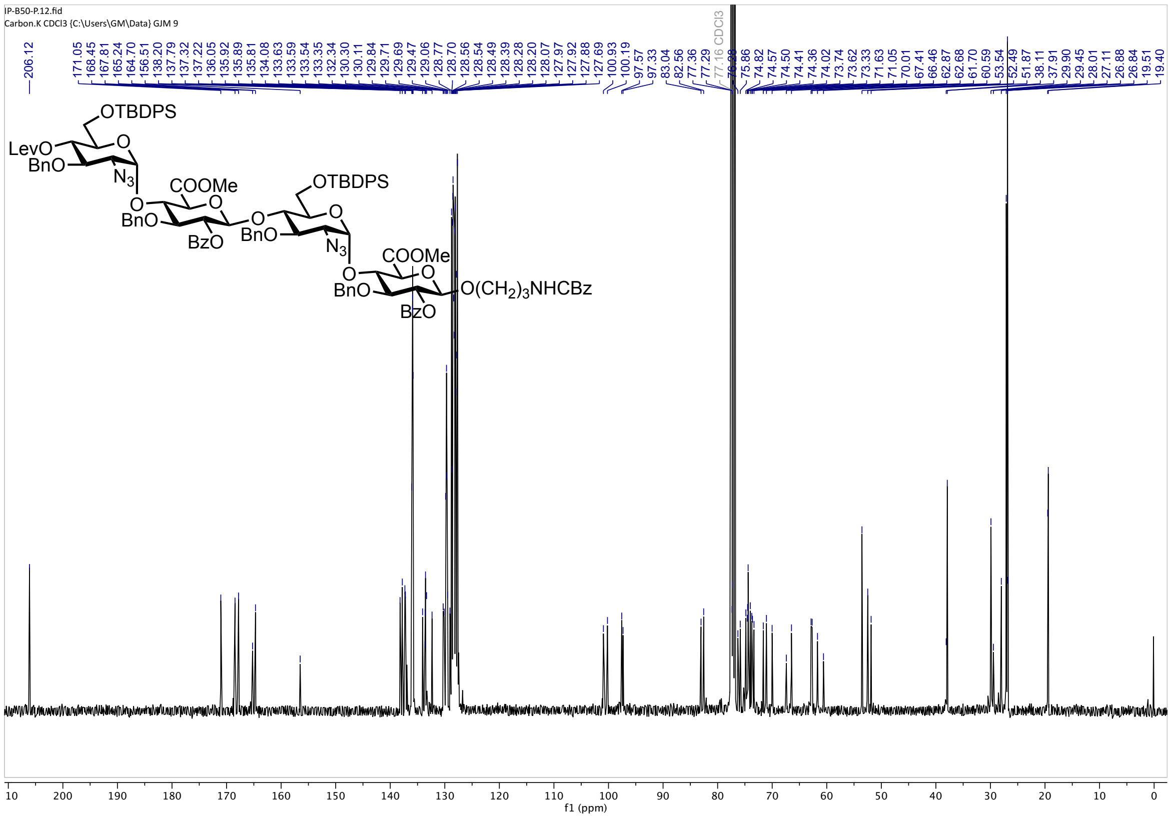

DEPT NMR (101 MHz, Chloroform-*d*) 20

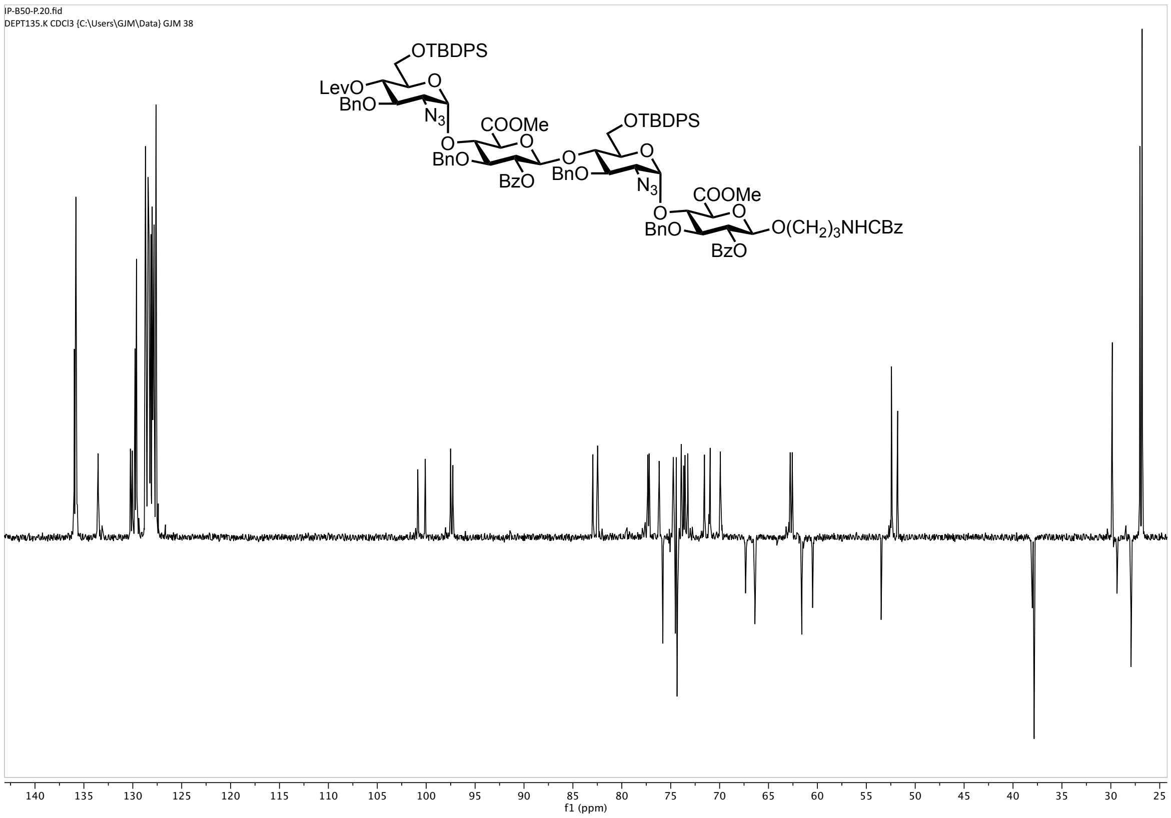

COSY NMR (400 MHz, Chloroform-*d*) 20

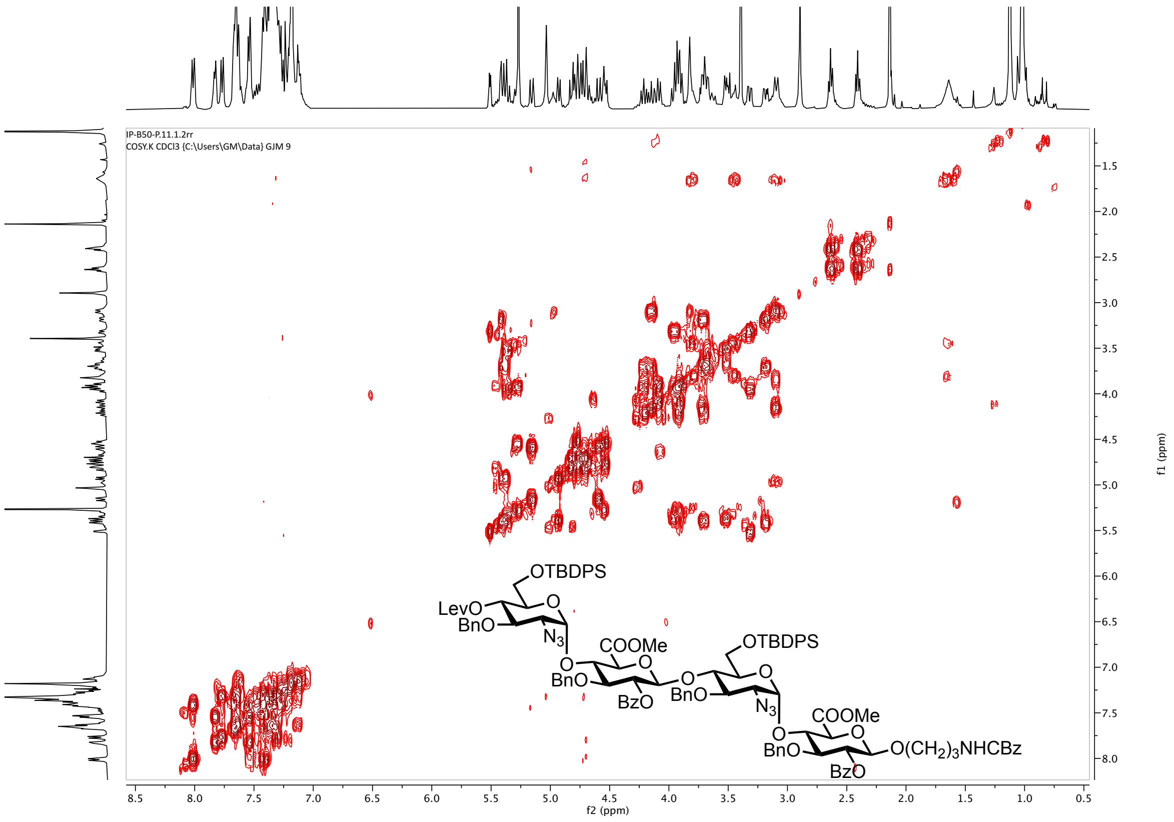

HSQC NMR (400 MHz x 101 MHz, Chloroform-*d*) 20

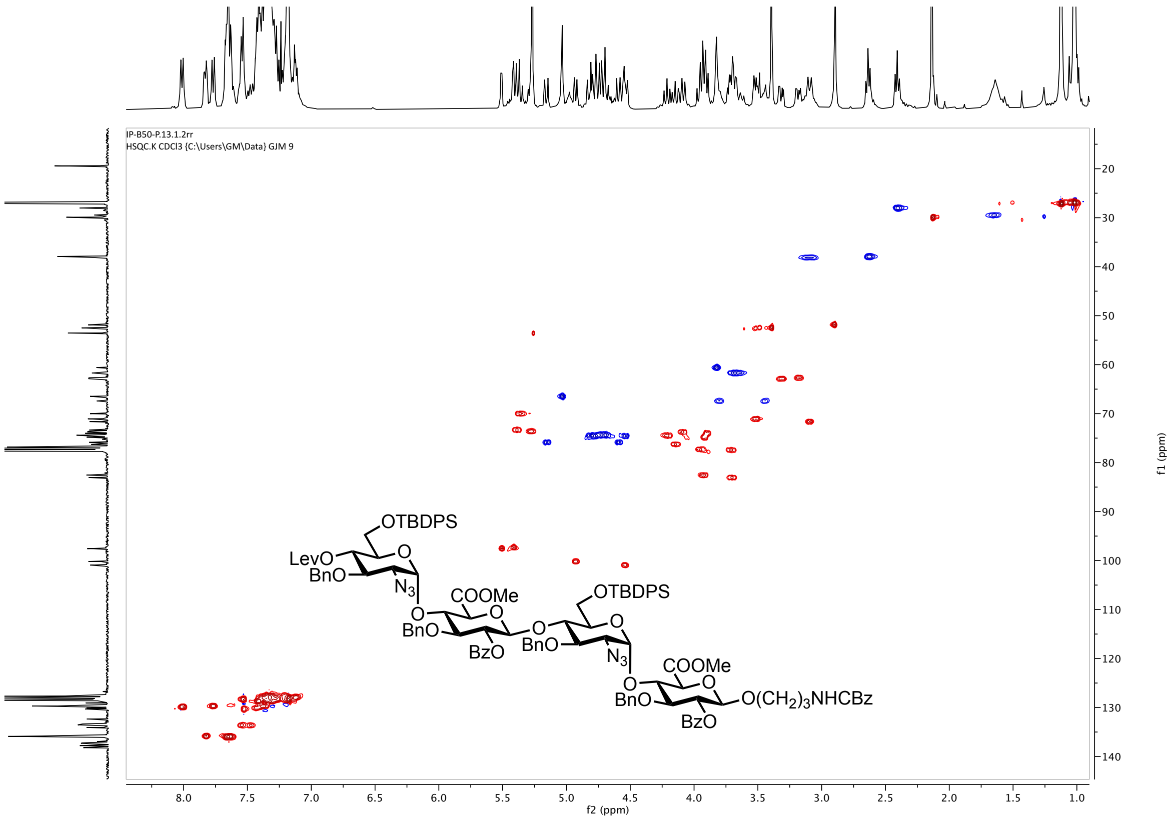

Coupled-HSQC NMR (400 MHz x 101 MHz, Chloroform-*d*) 20

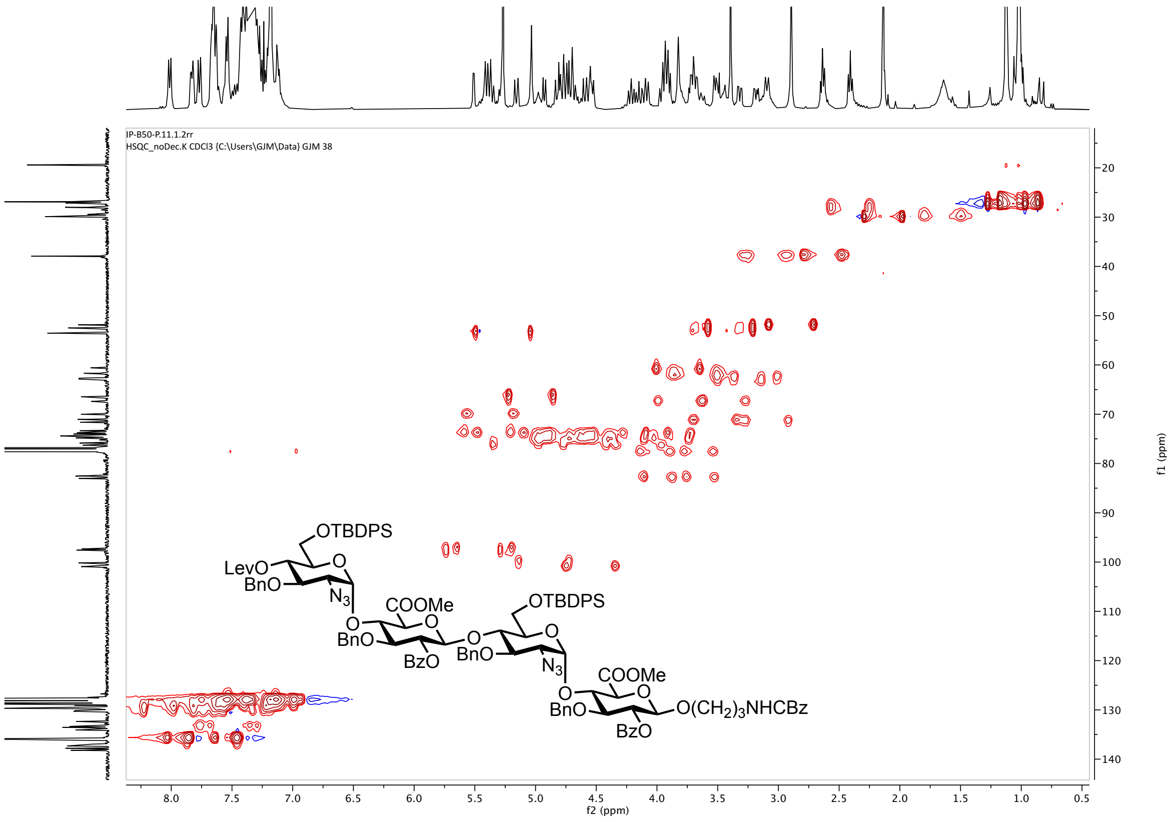

HMBC NMR (400 MHz x 101 MHz, Chloroform-*d*) 20

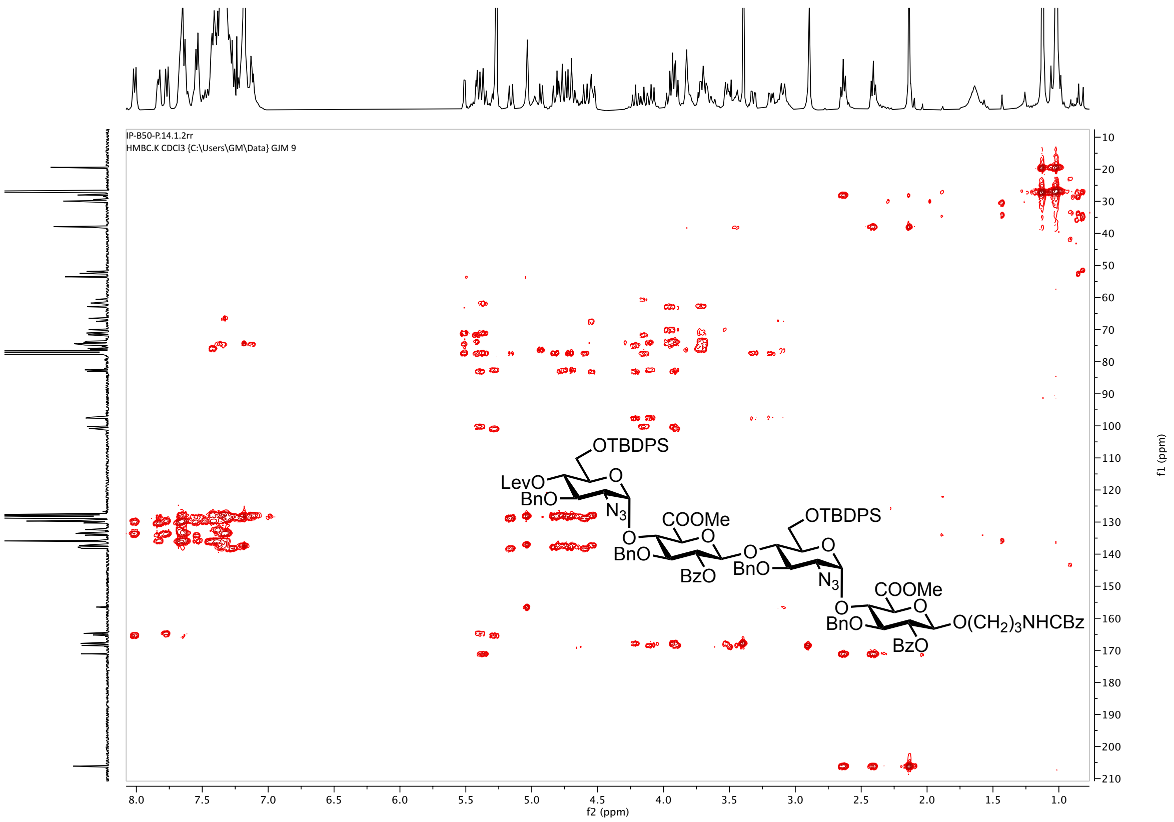

Compound 21

^{1}H NMR (400 MHz, Chloroform-*d*) 21

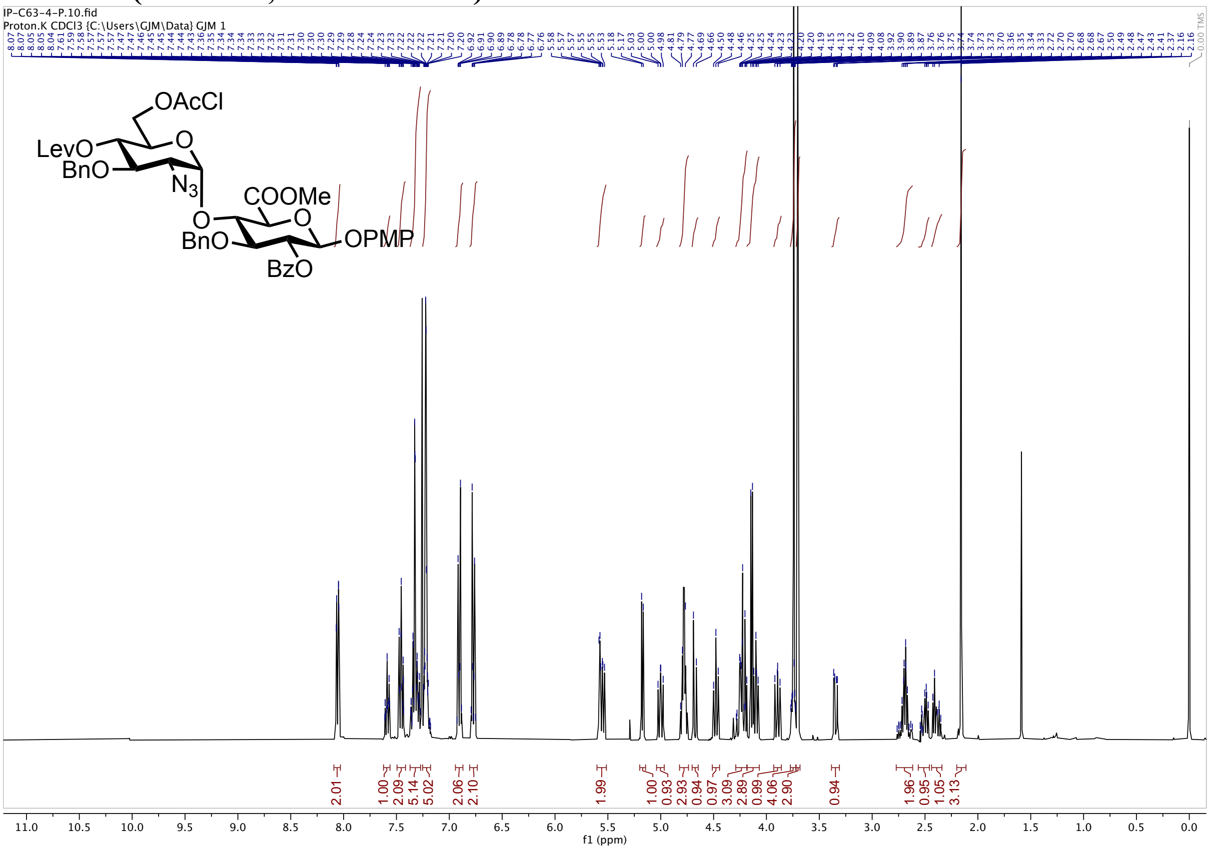

^{13}C{^{1}H} NMR (101 MHz, Chloroform-*d*) 21

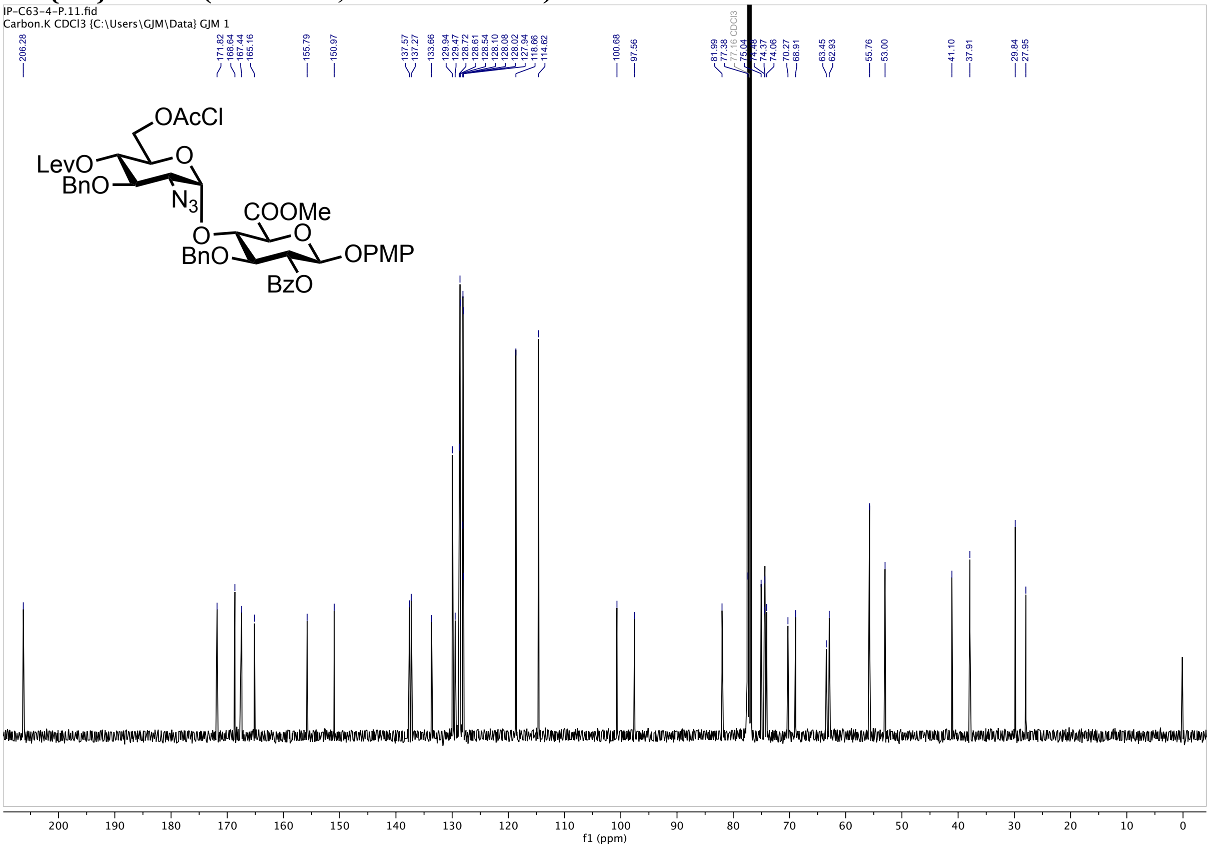

DEPT NMR (101 MHz, Chloroform-*d*) 21

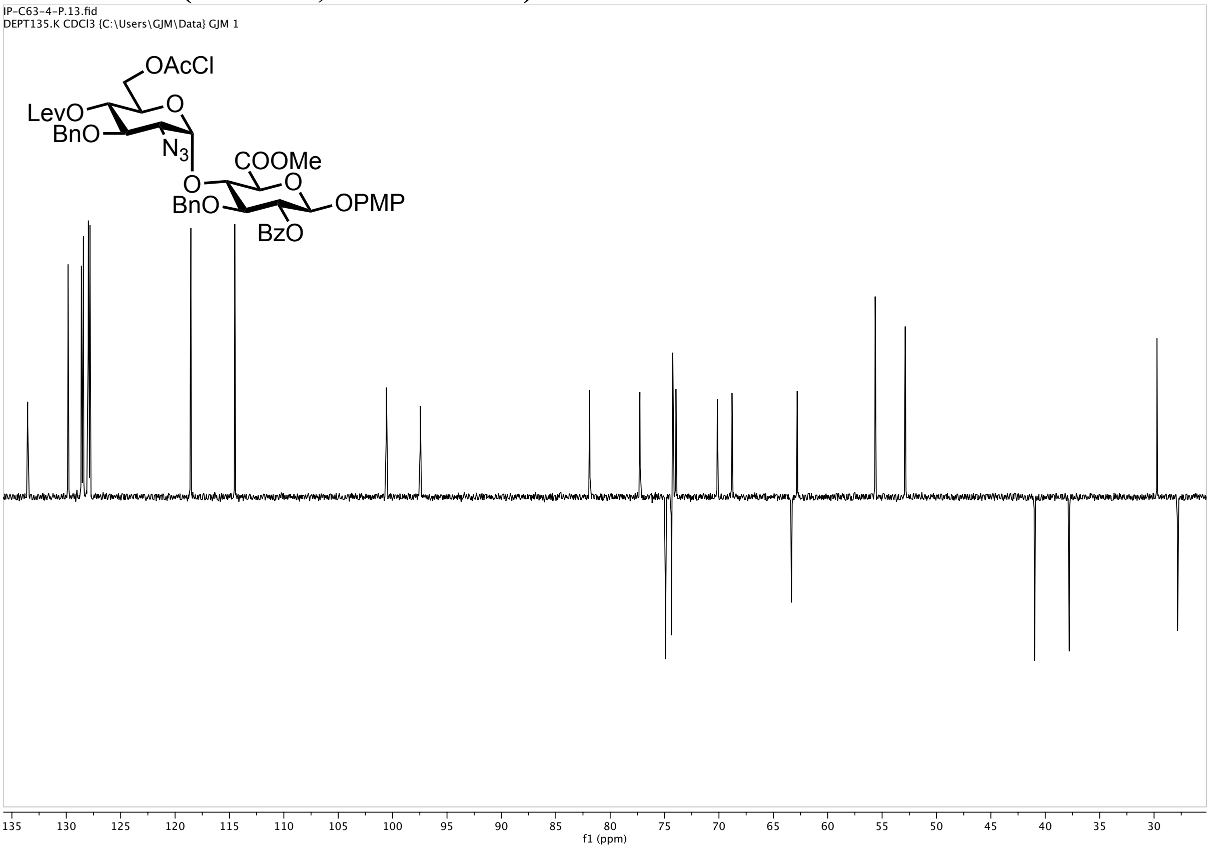

COSY NMR (400 MHz, Chloroform-*d*) 21

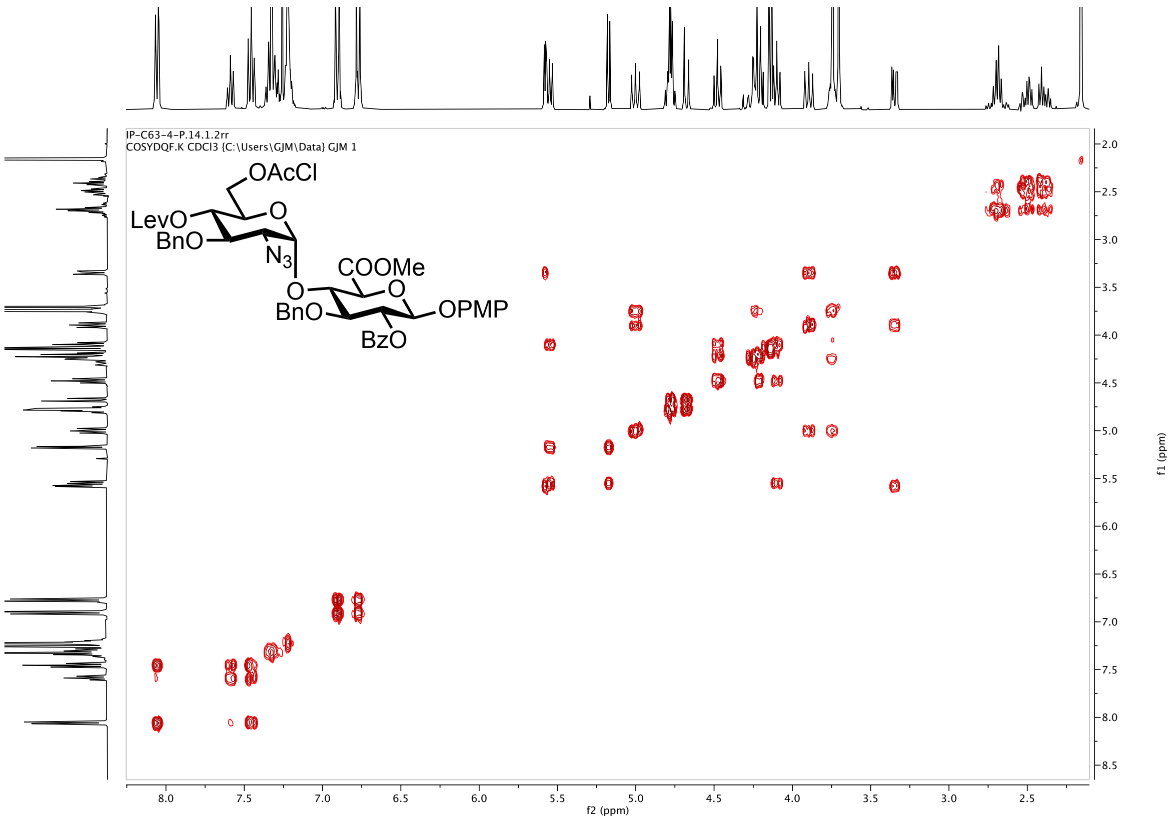

HSQC NMR (400 MHz x 101 MHz, Chloroform-*d*) 21

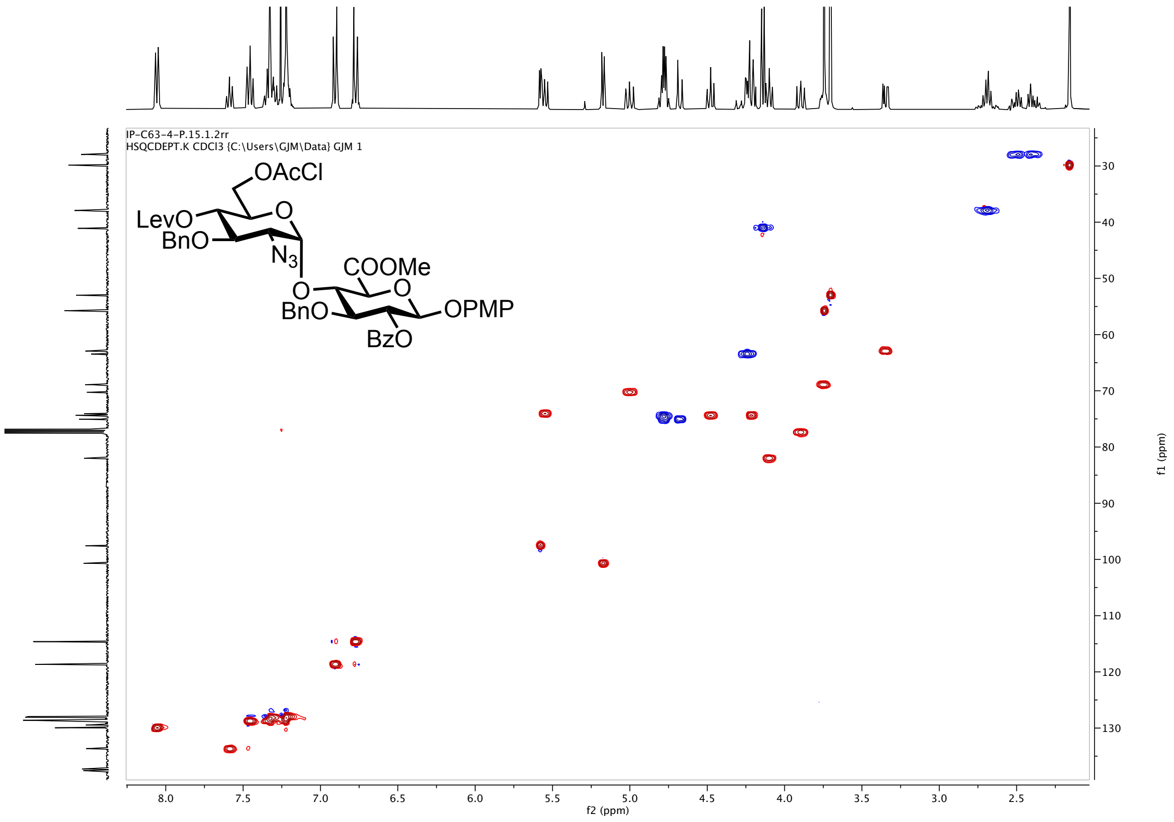

HMBC NMR (400 MHz x 101 MHz, Chloroform-*d*) 21

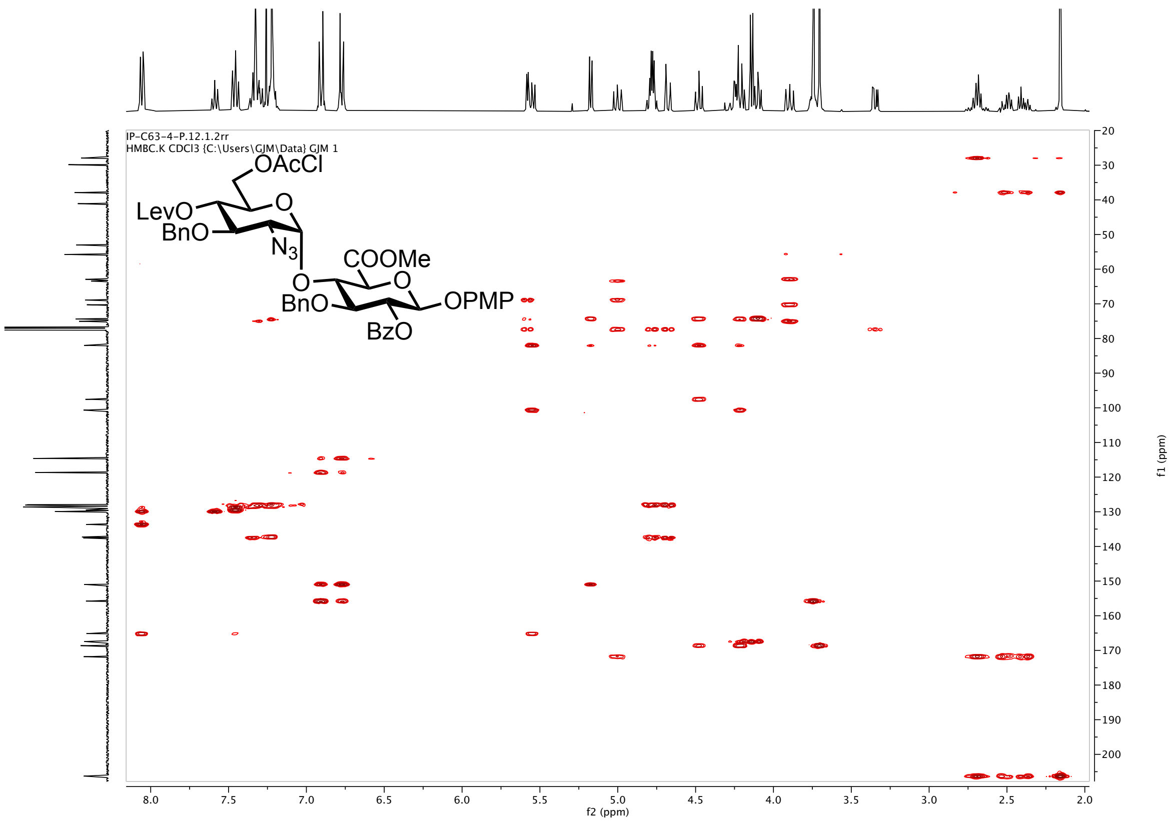

Compound 22

^{1}H NMR (400 MHz, Chloroform-*d*) 22

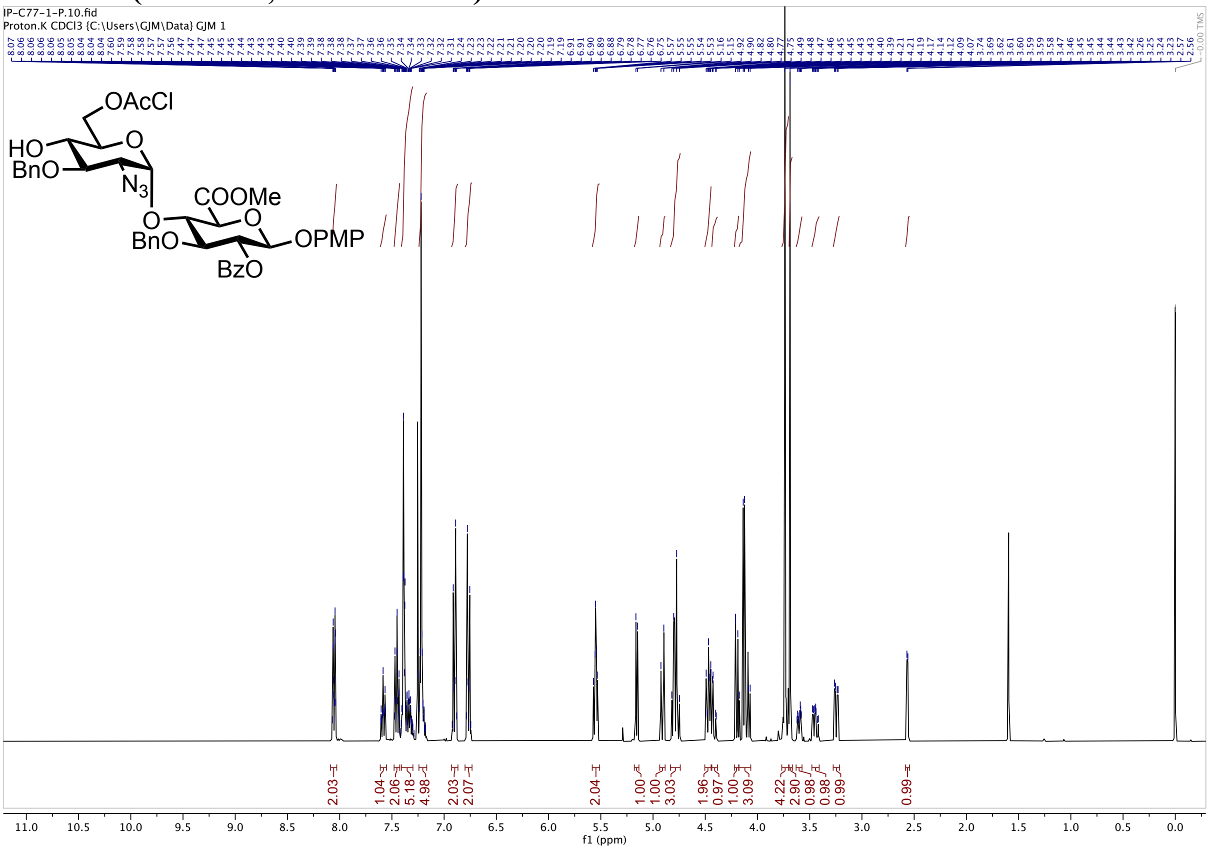

$^{13}C\{^{1}H\}$ NMR (101 MHz, Chloroform-*d*) 22

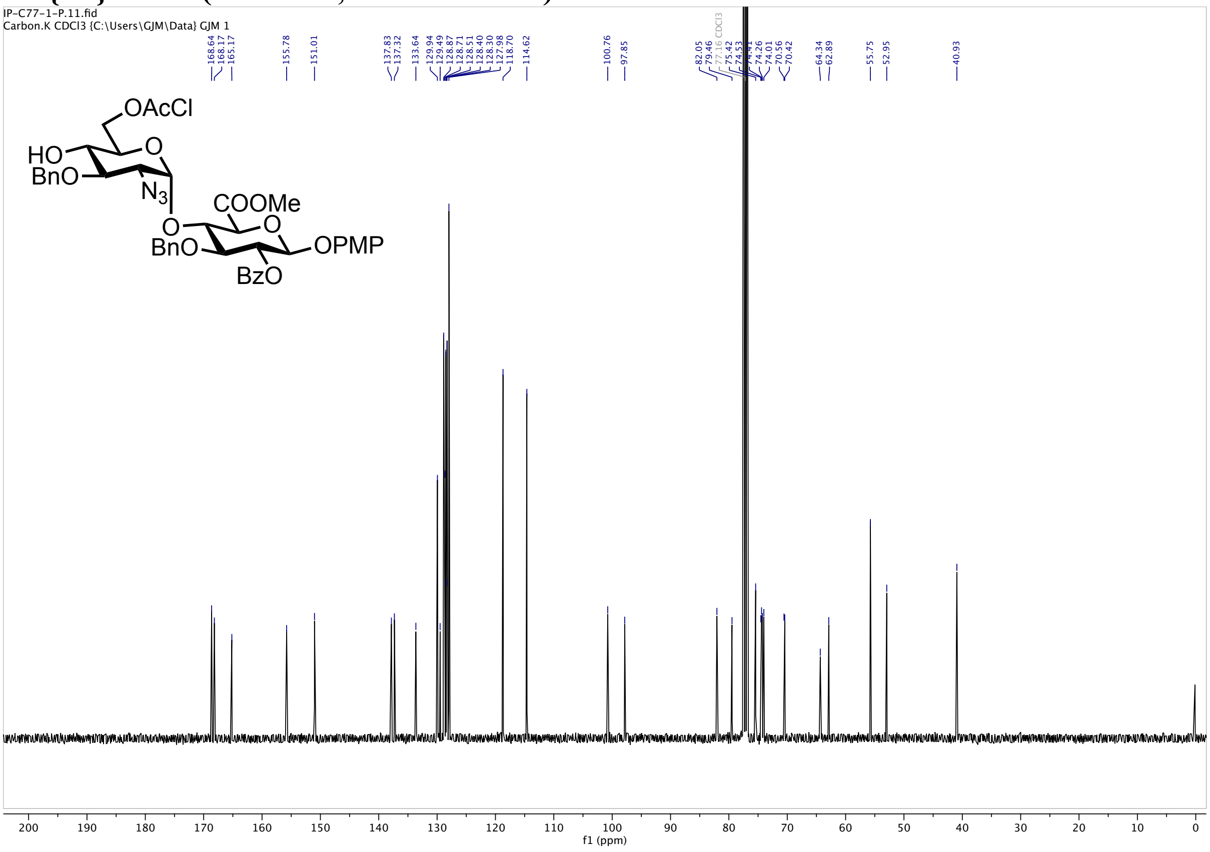

DEPT NMR (101 MHz, Chloroform-*d*) 22

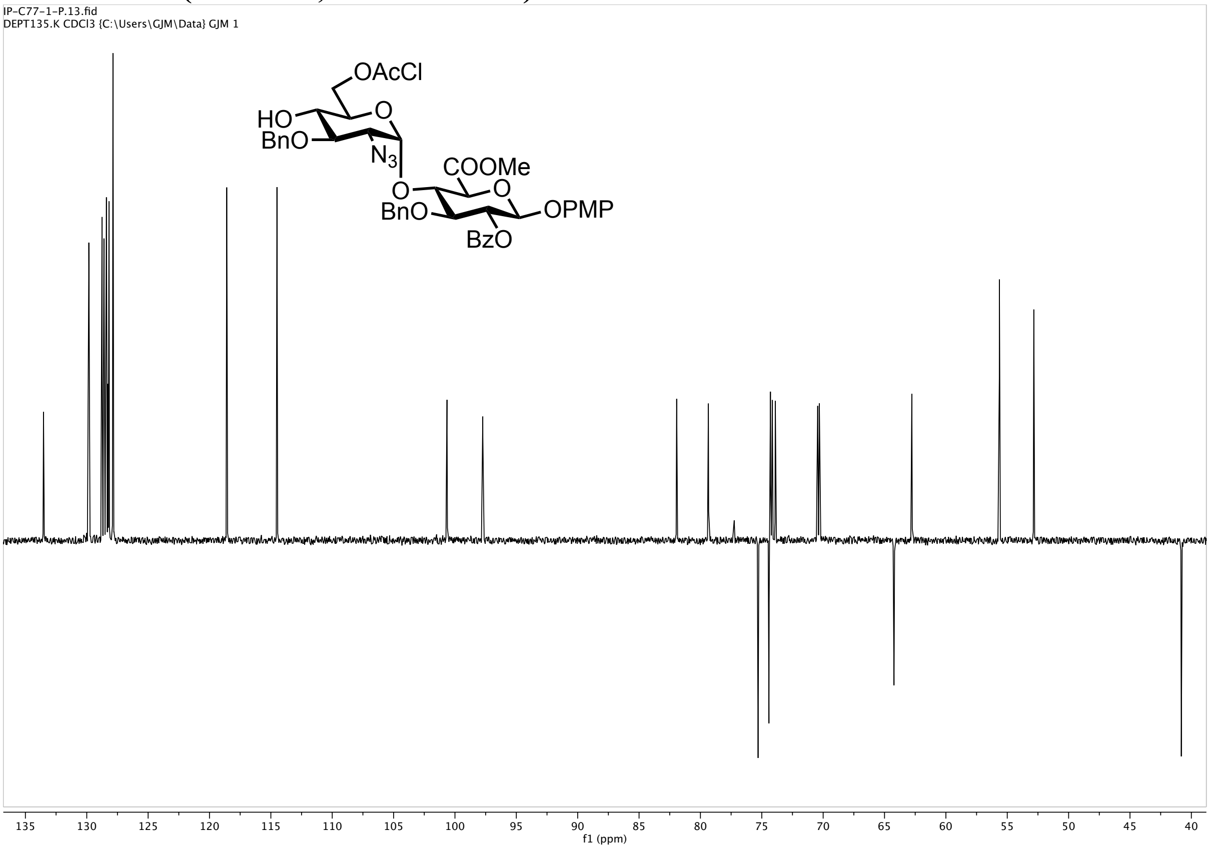

COSY NMR (400 MHz, Chloroform-*d*) 22

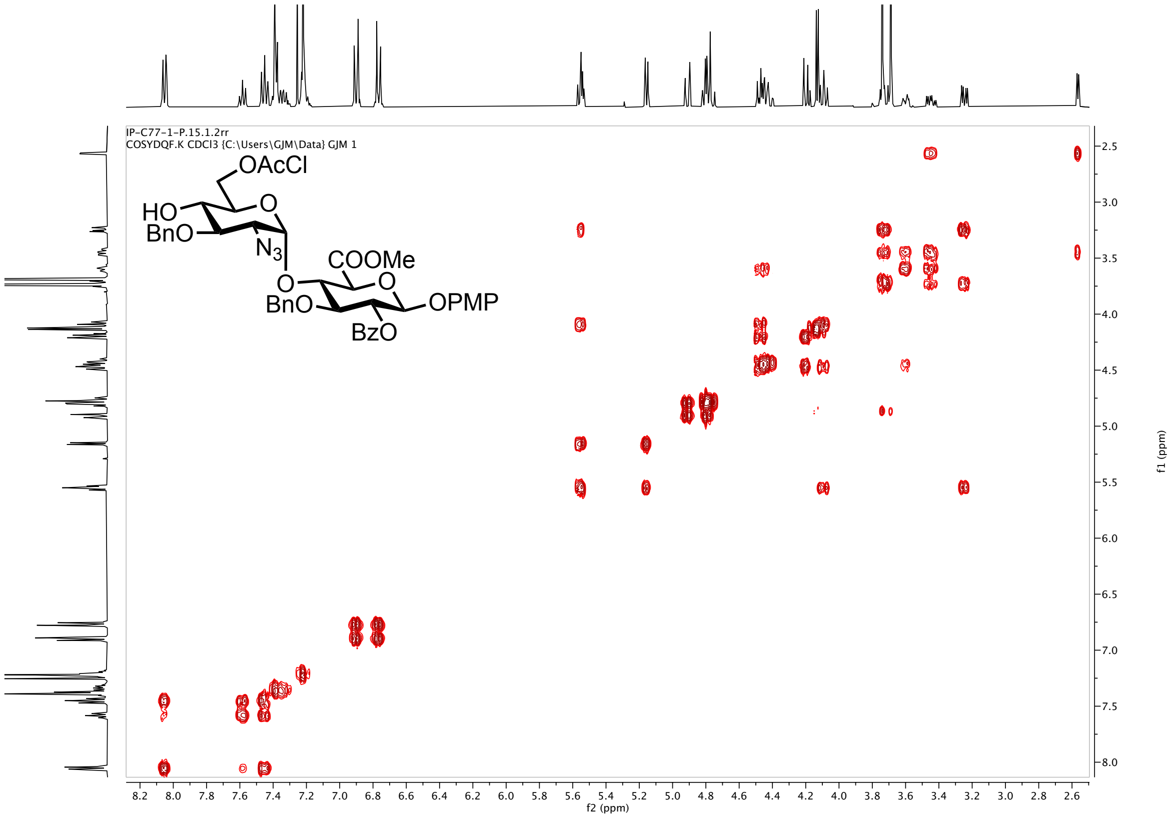

HSQC NMR (400 MHz x 101 MHz, Chloroform-*d*) 22

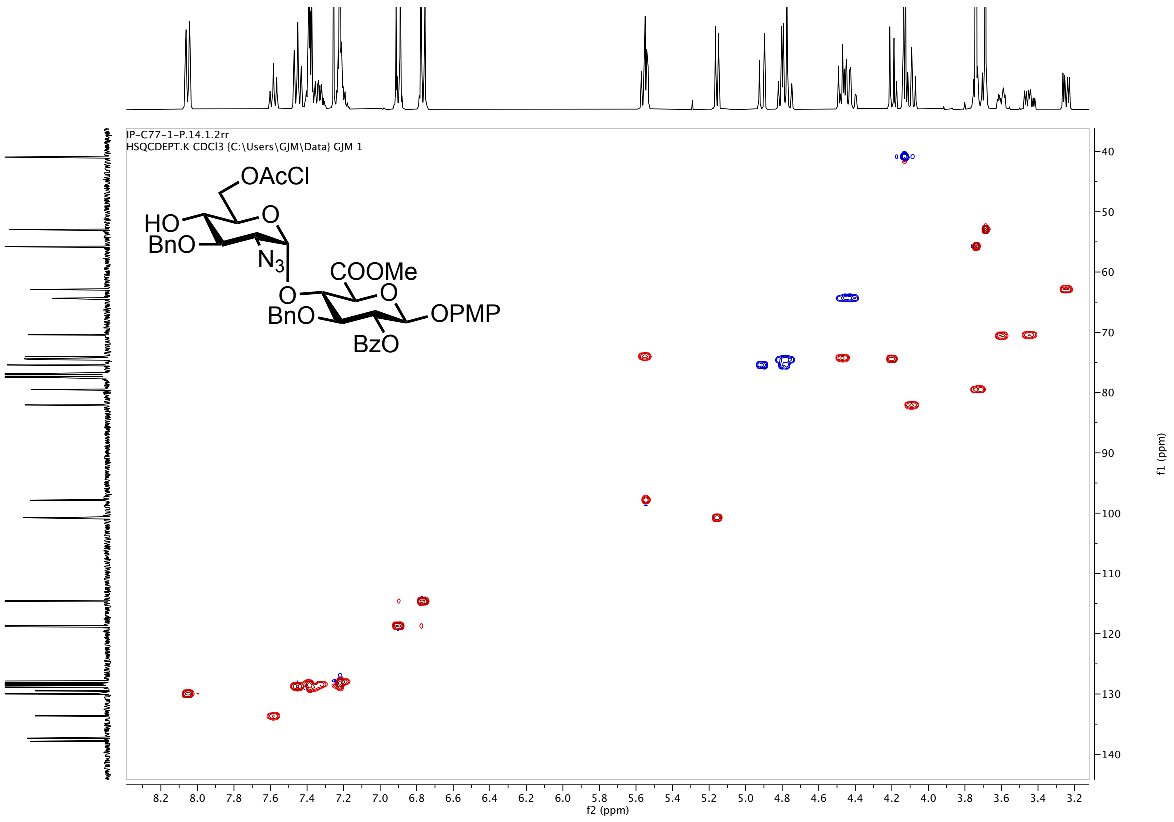

HMBC NMR (400 MHz x 101 MHz, Chloroform-*d*) 22

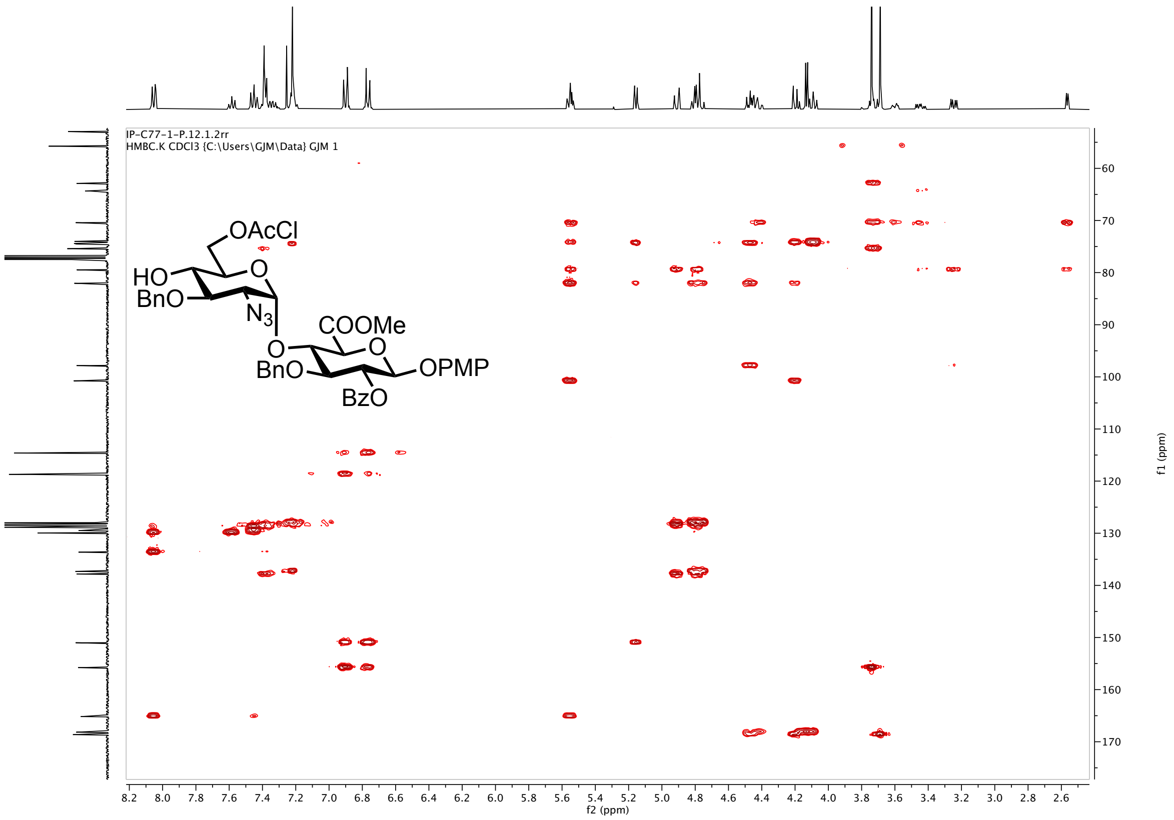

Compound 23

^{1}H NMR (400 MHz, Chloroform-*d*) 23

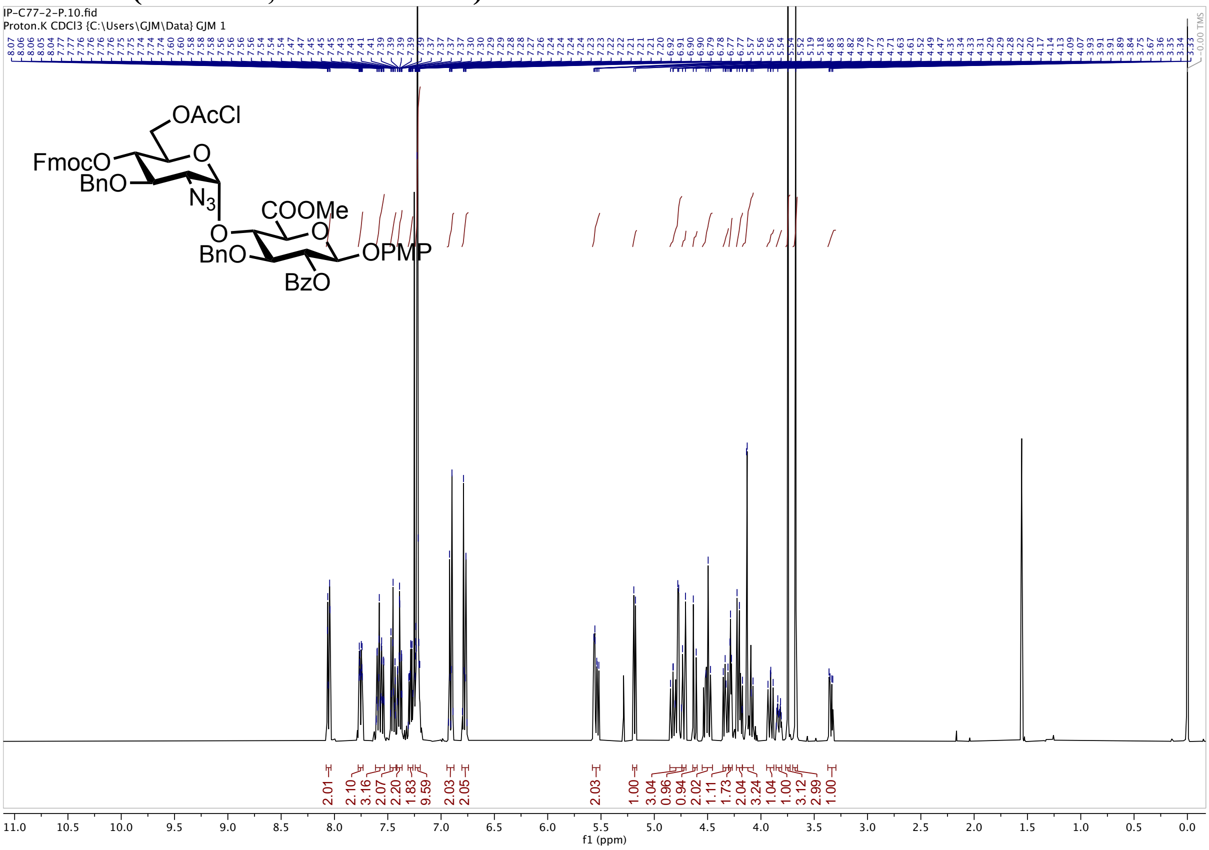

^{13}C{^{1}H} NMR (101 MHz, Chloroform-*d*) 23

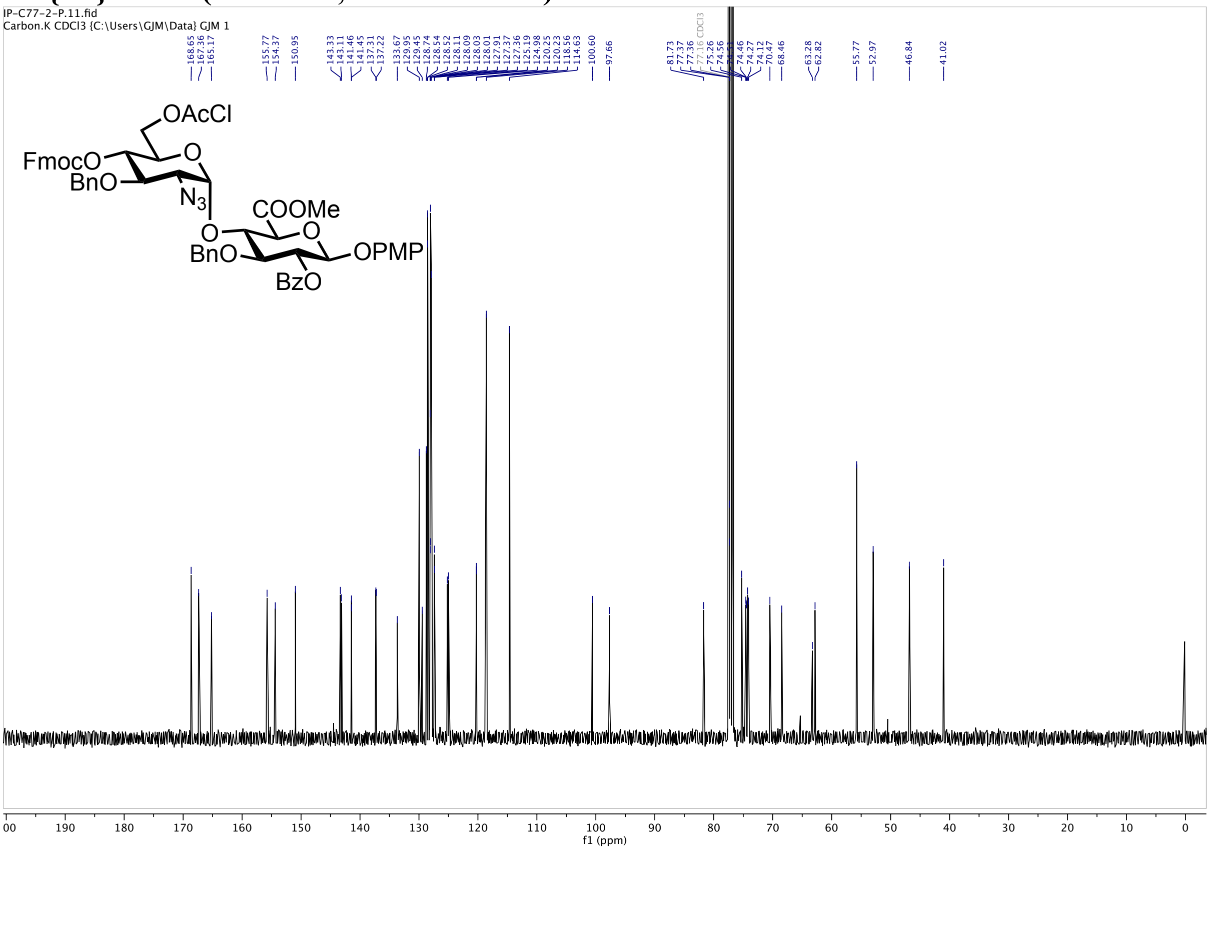

DEPT NMR (101 MHz, Chloroform-*d*) 23

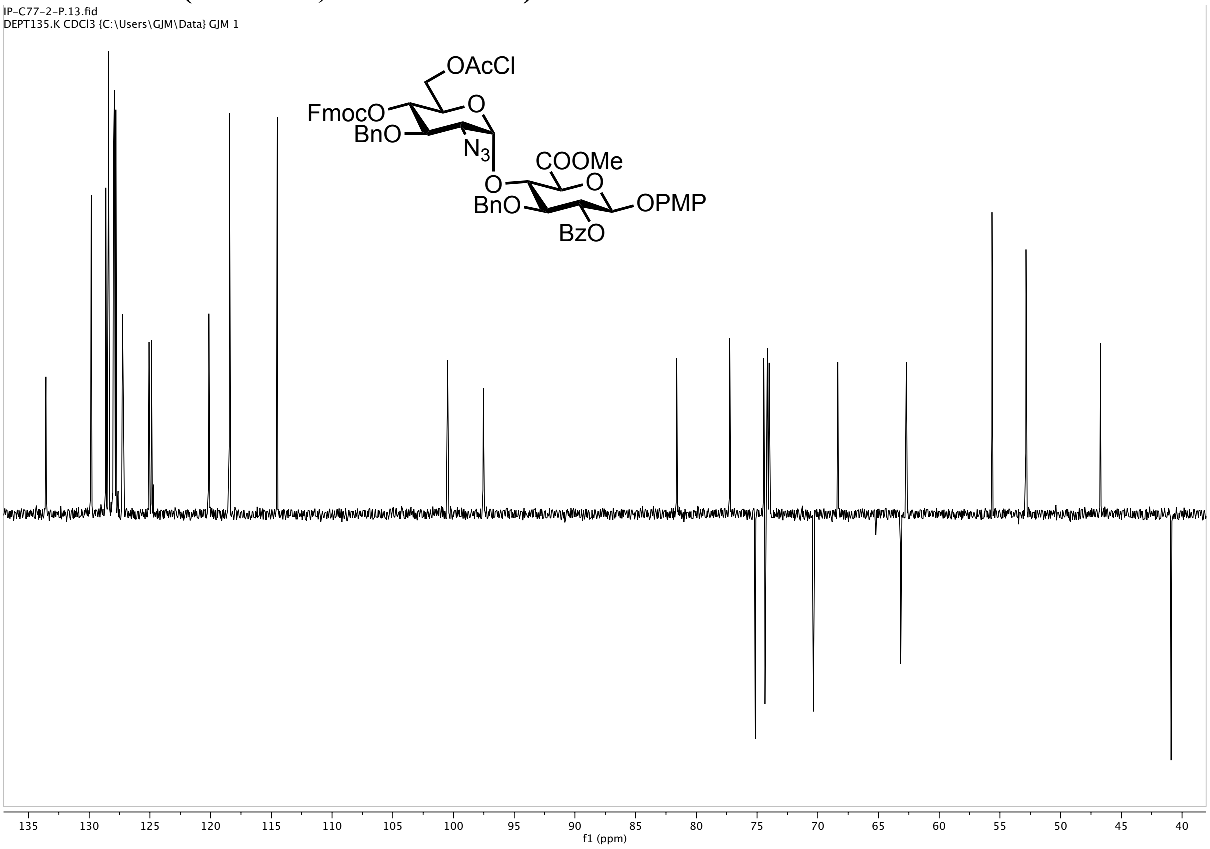

COSY NMR (400 MHz, Chloroform-*d*) 23

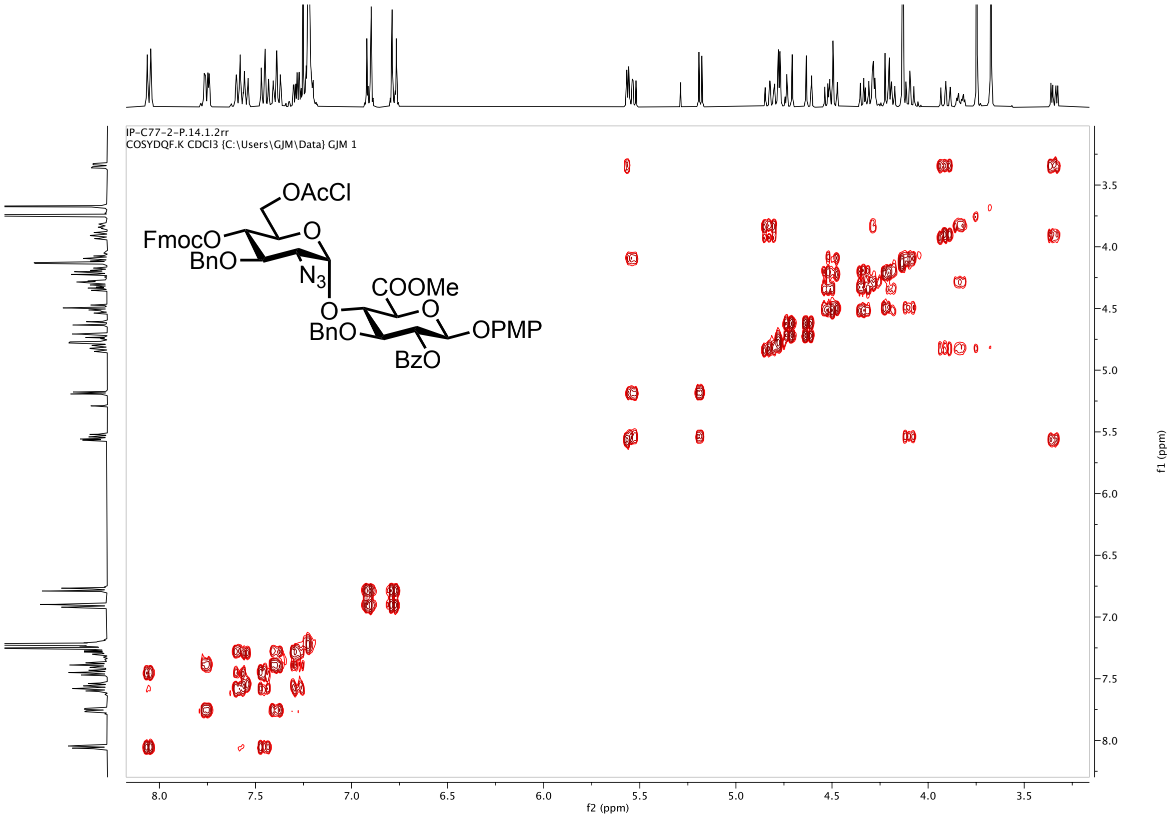

HSQC NMR (400 MHz x 101 MHz, Chloroform-*d*) 23

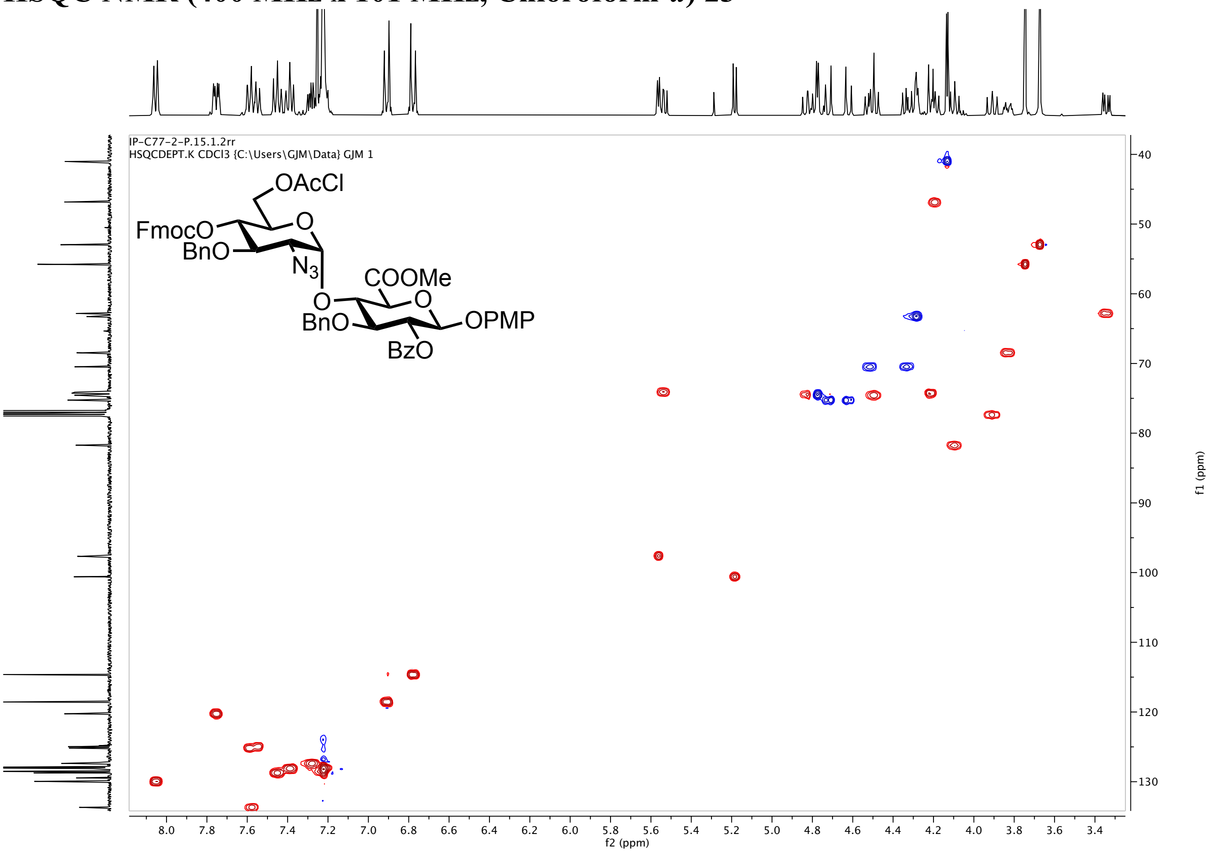

HMBC NMR (400 MHz x 101 MHz, Chloroform-*d*) 23

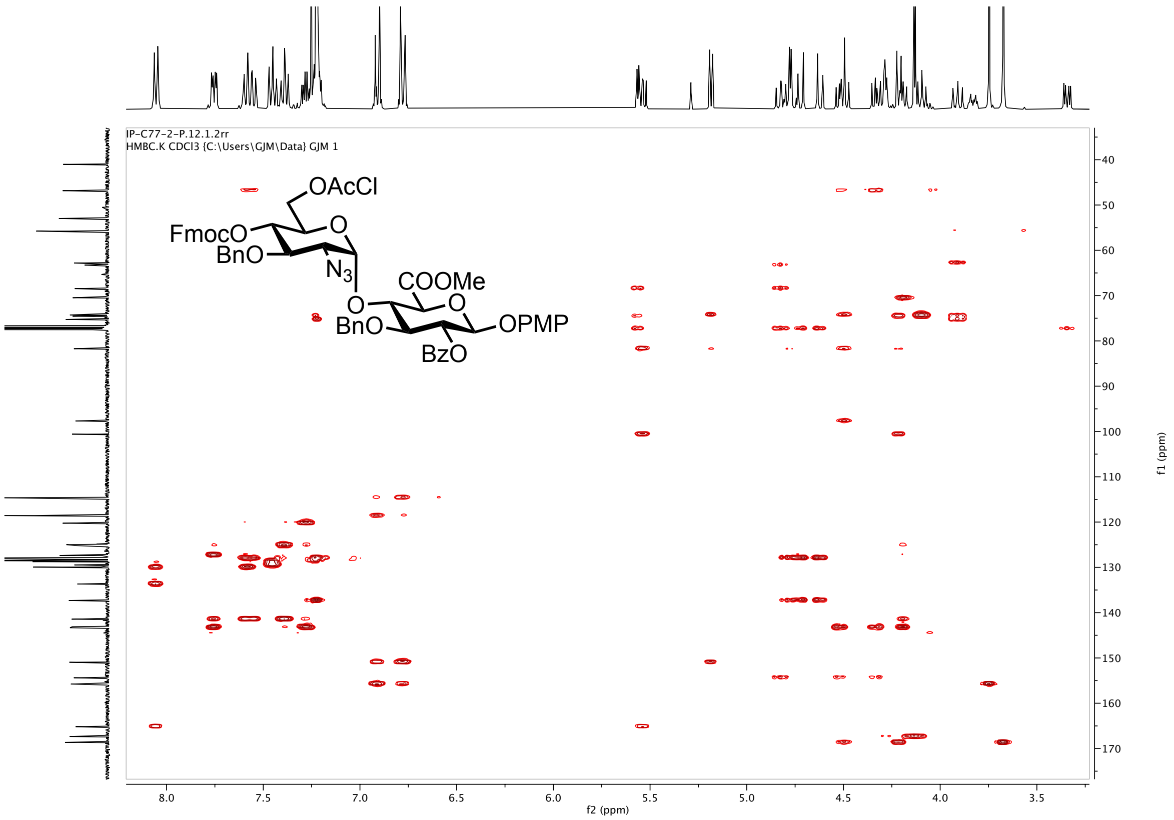

Compound S18

^{1}H NMR (400 MHz, Chloroform-*d*) S18

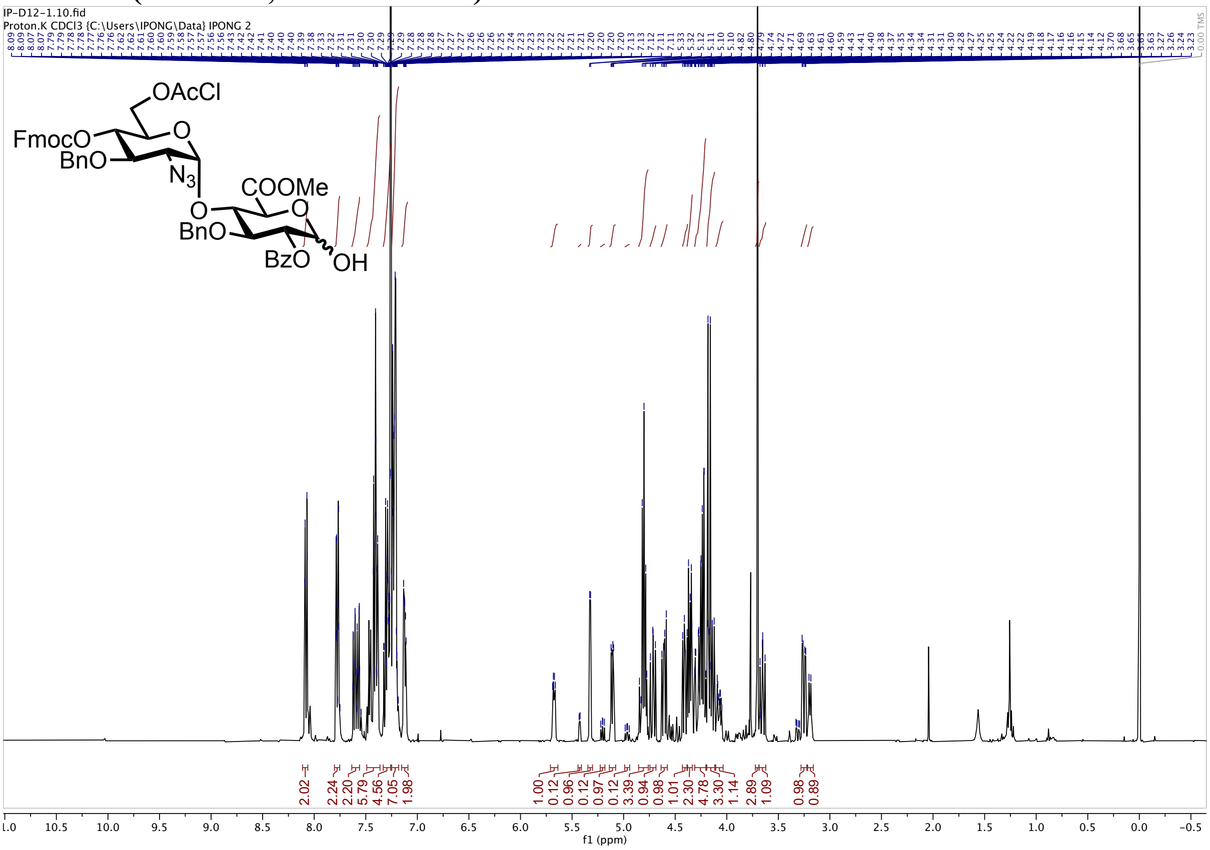

^{13}C{^{1}H} NMR (101 MHz, Chloroform-*d*) S18

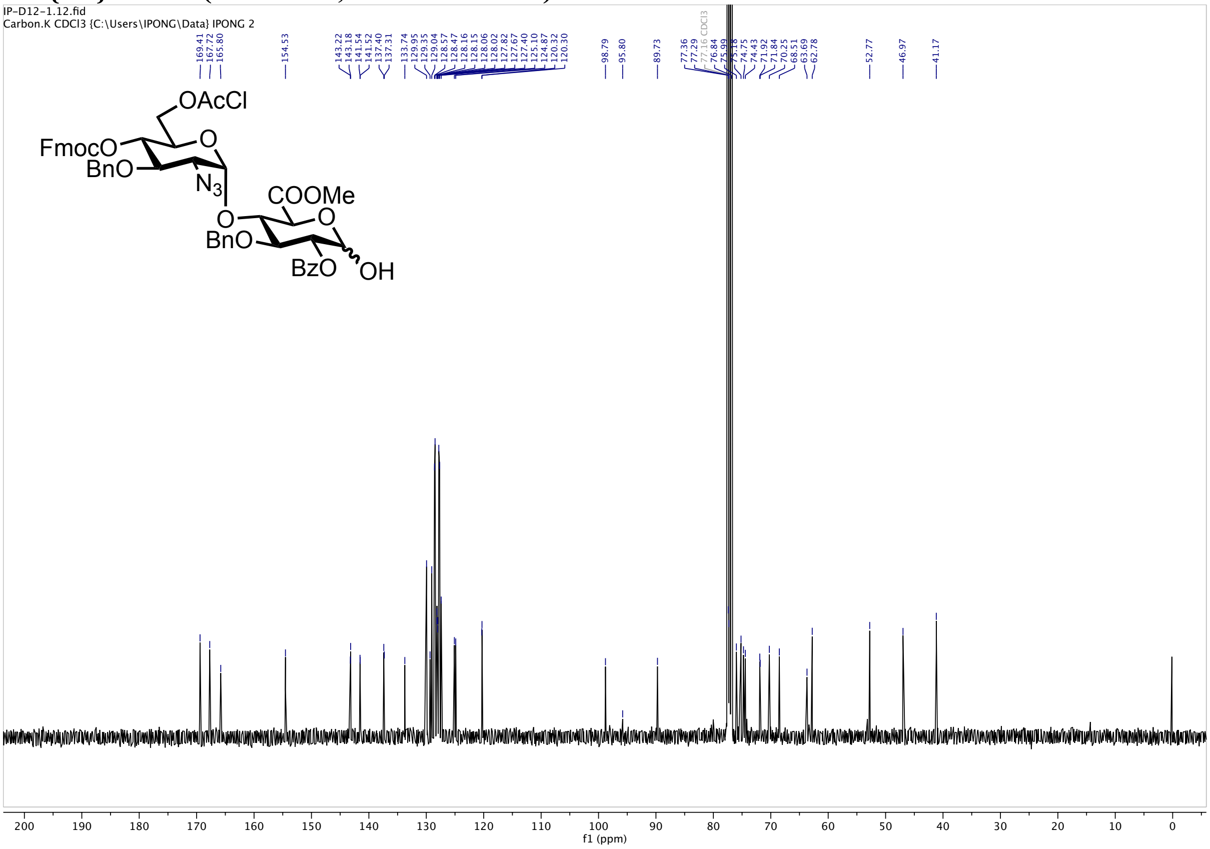

DEPT NMR (101 MHz, Chloroform-*d*) S18

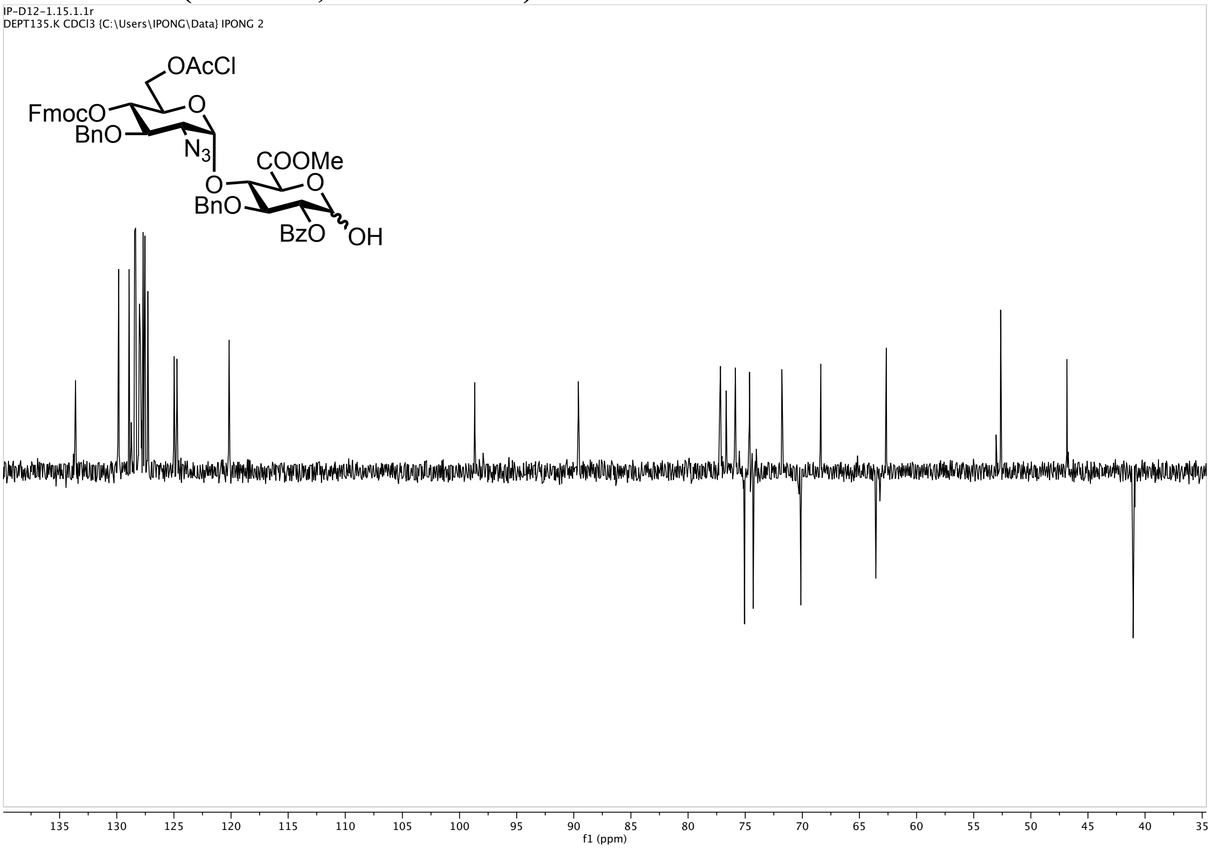

COSY NMR (400 MHz, Chloroform-*d*) S18

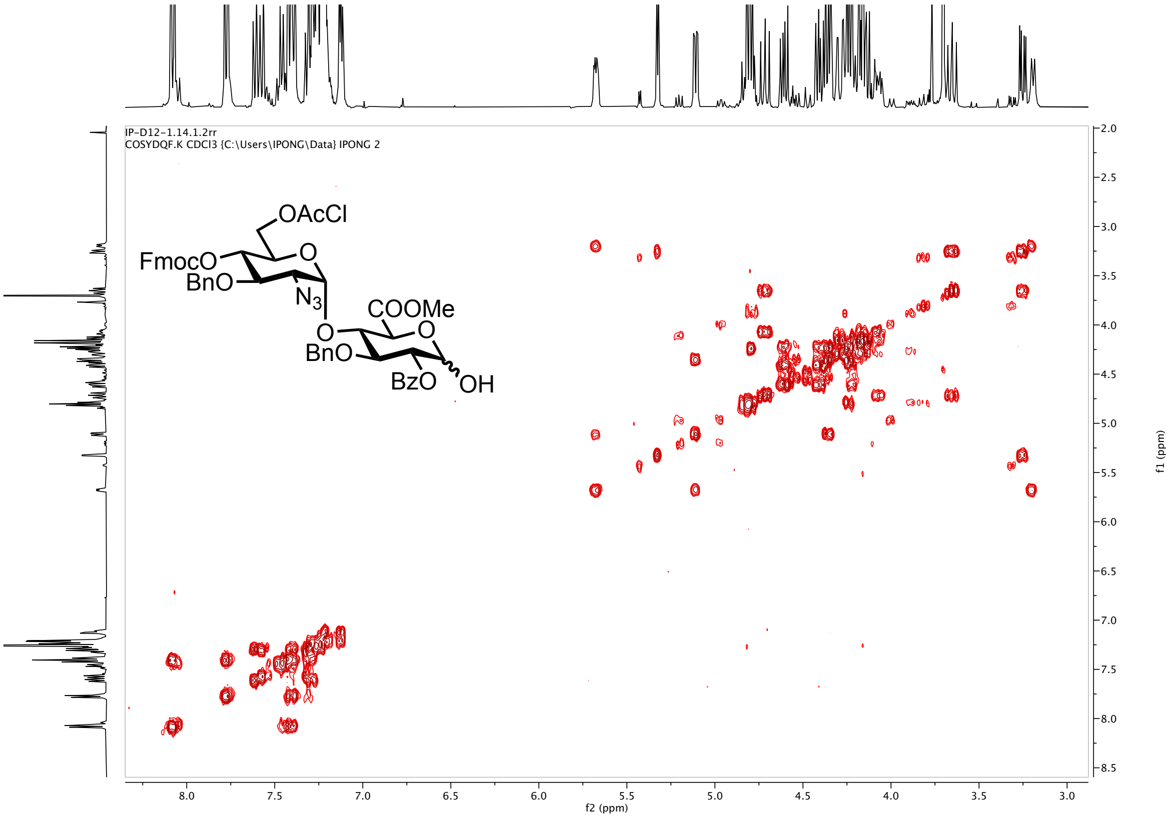

HSQC NMR (400 MHz x 101 MHz, Chloroform-*d*) S18

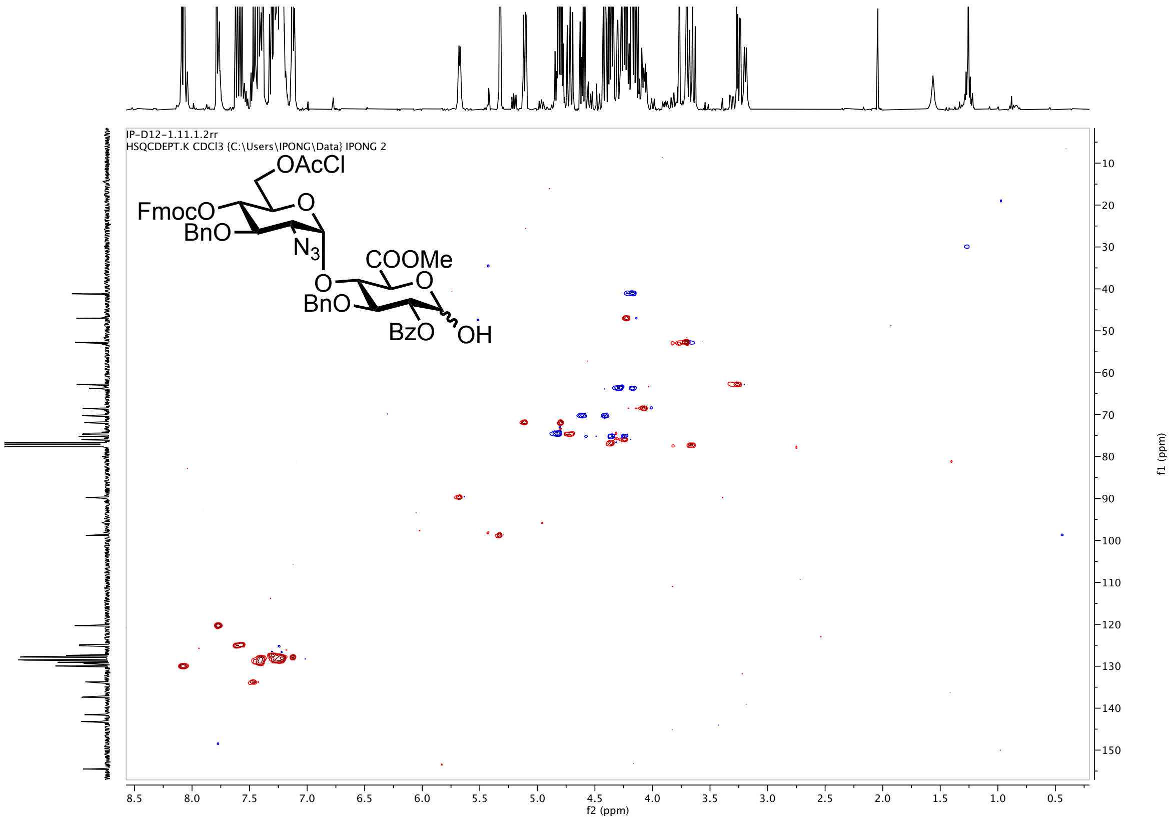

Coupled-HSQC NMR (400 MHz x 101 MHz, Chloroform-*d*) S18

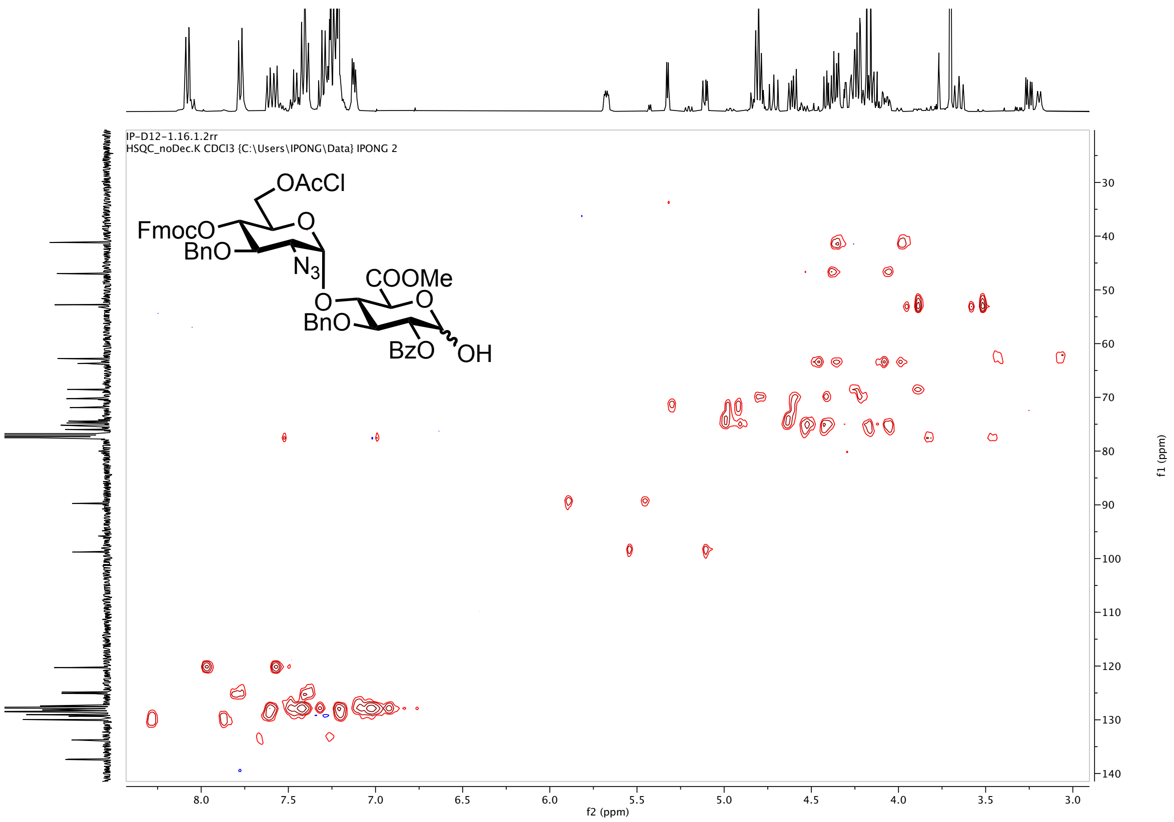

HMBC NMR (400 MHz x 101 MHz, Chloroform-*d*) S18

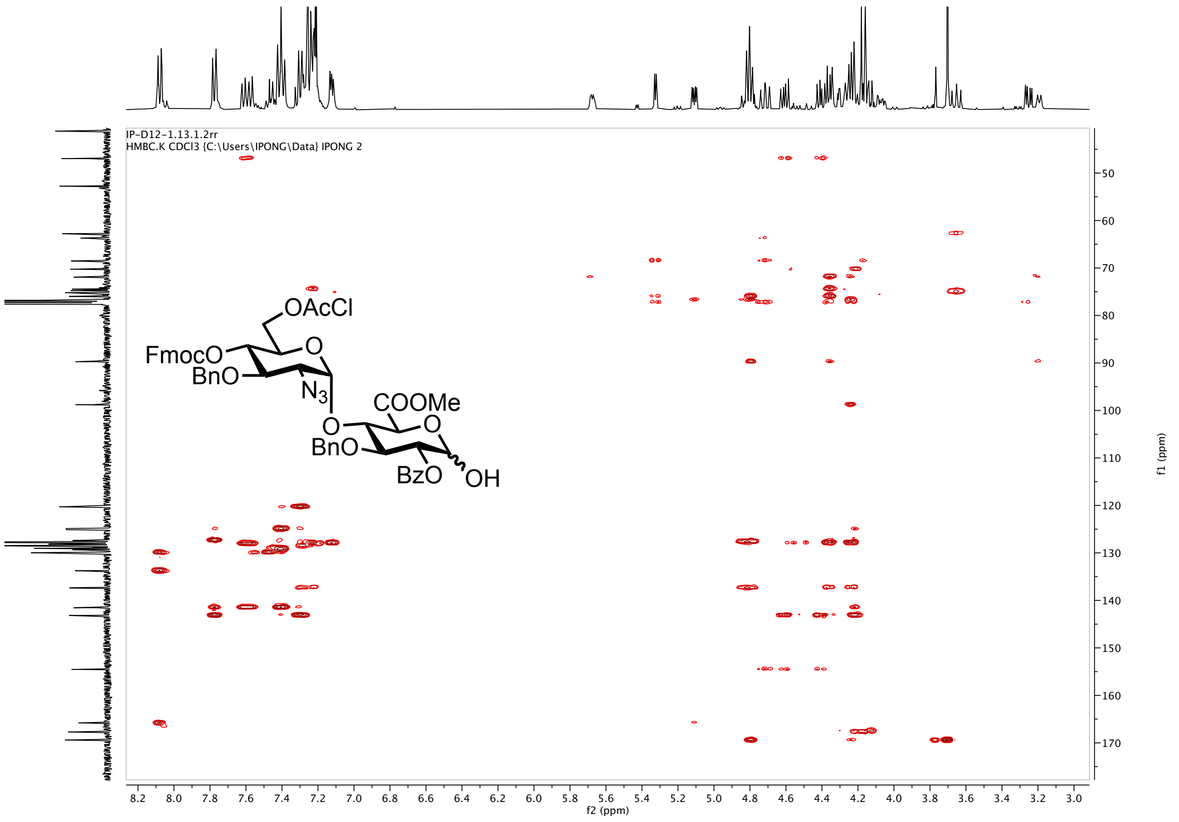

Compound 24

^{1}H NMR (400 MHz, Chloroform-*d*) 24

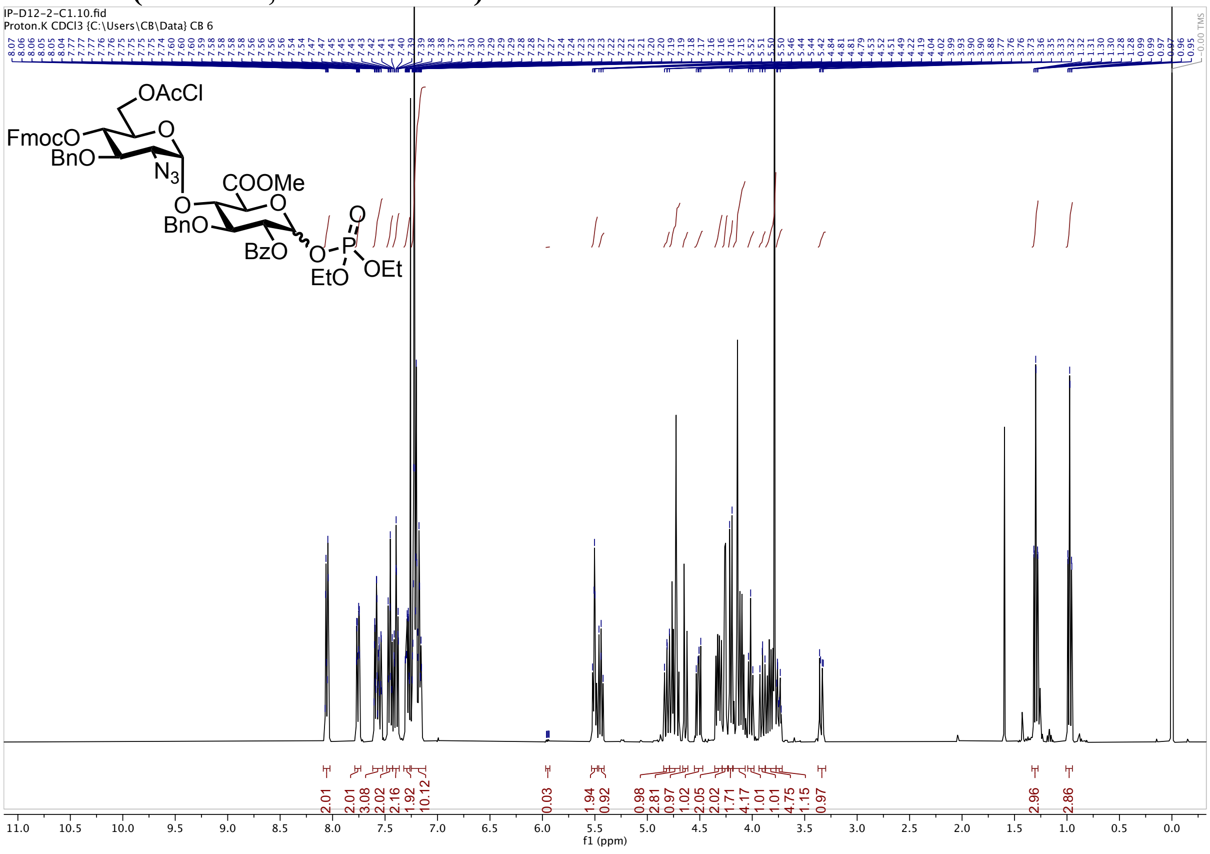

^{31}P NMR (162 MHz, Chloroform-*d*) 24

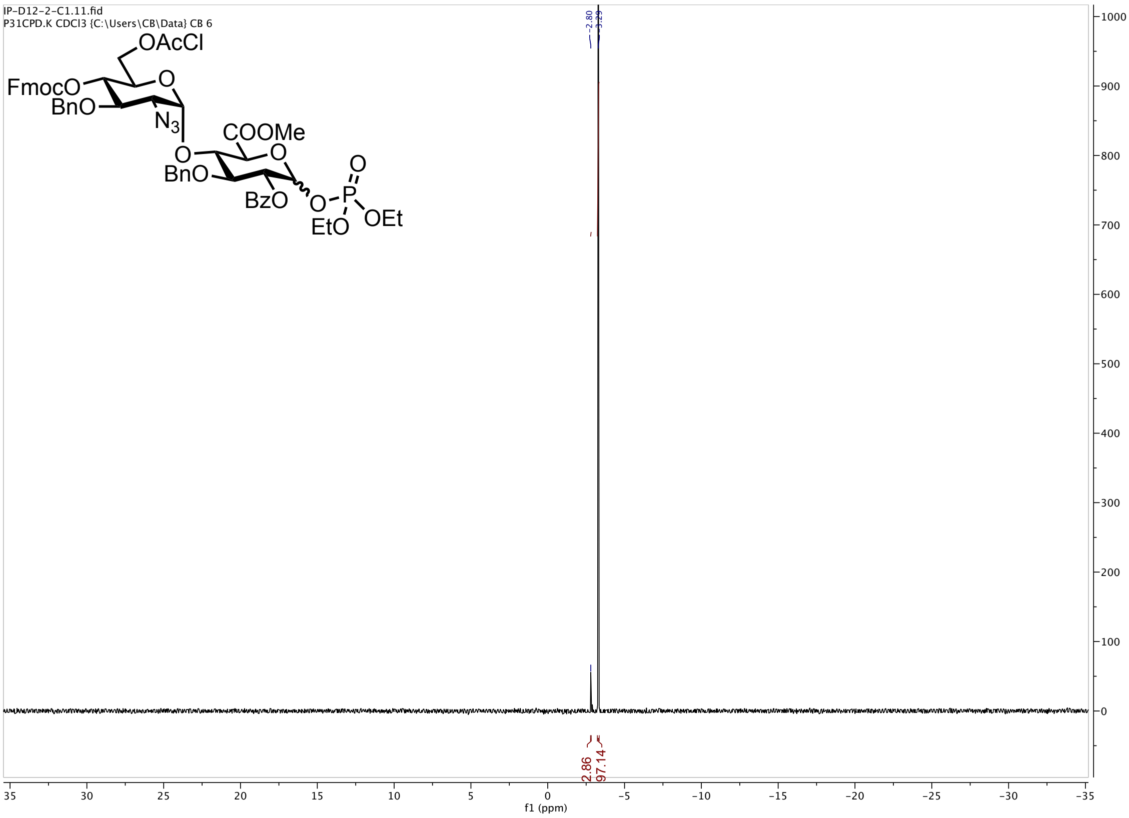

^{13}C{^{1}H} NMR (101 MHz, Chloroform-*d*) 24

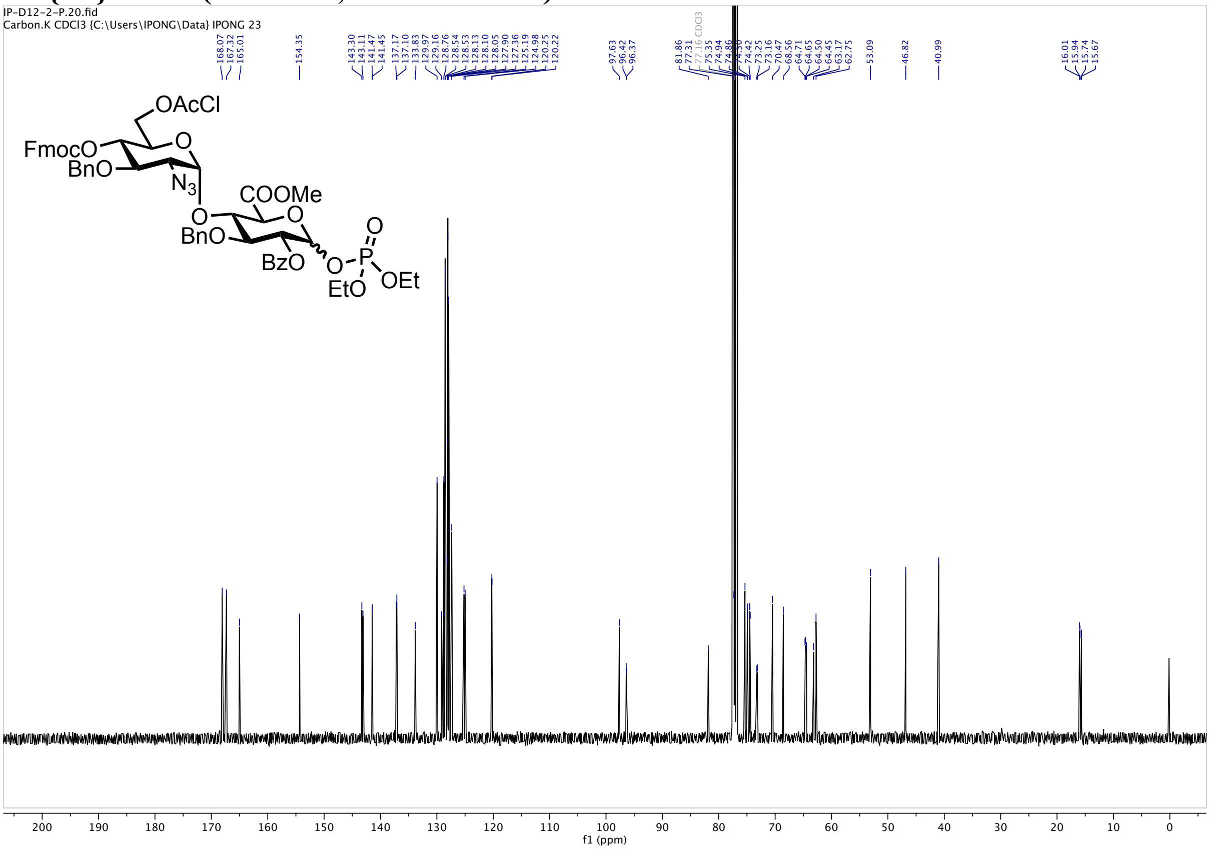

DEPT NMR (101 MHz, Chloroform-*d*) 24

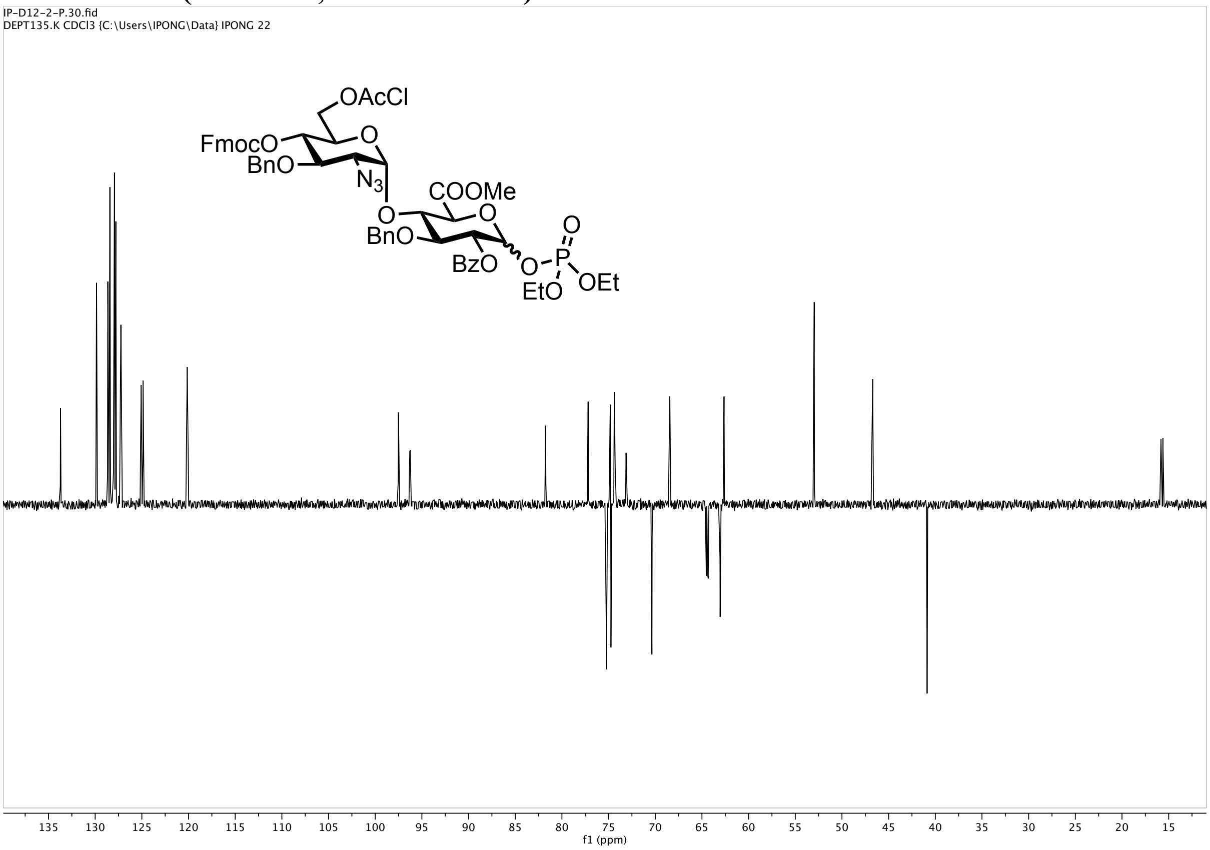

COSY NMR (400 MHz, Chloroform-*d*) 24

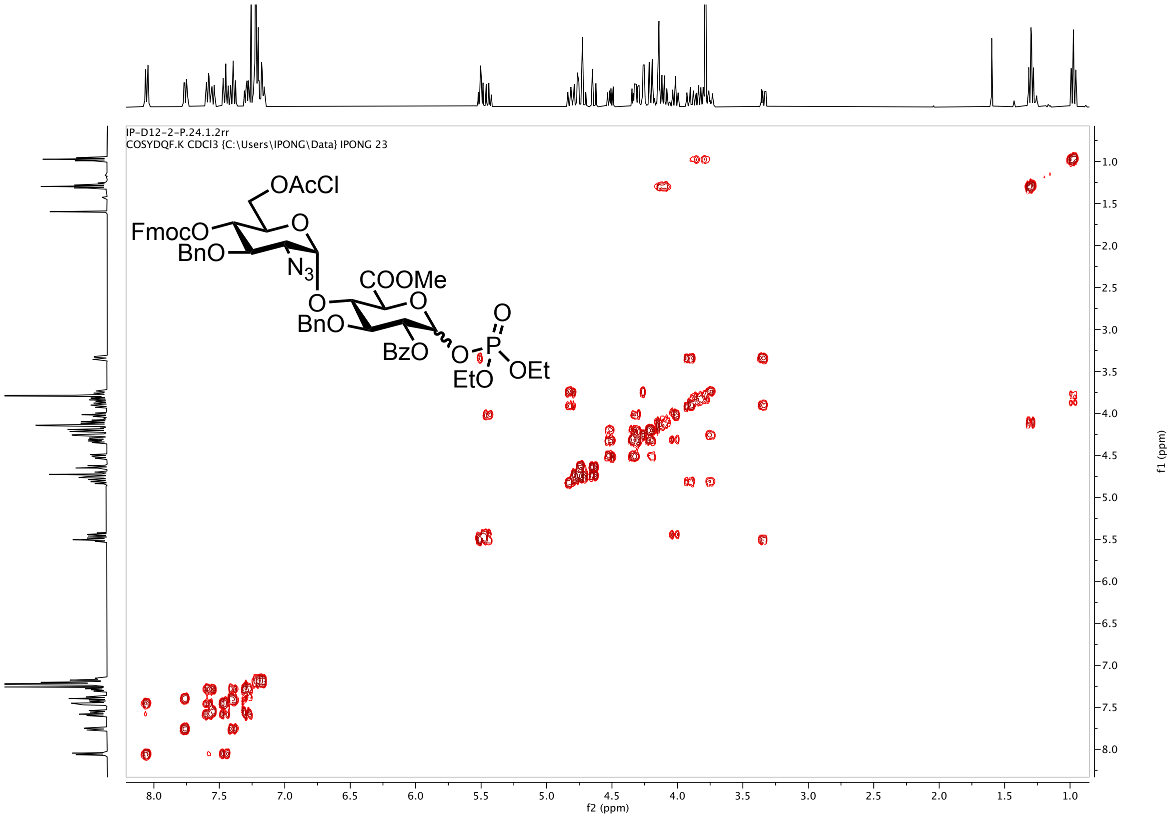

HSQC NMR (400 MHz x 101 MHz, Chloroform-*d*) 24

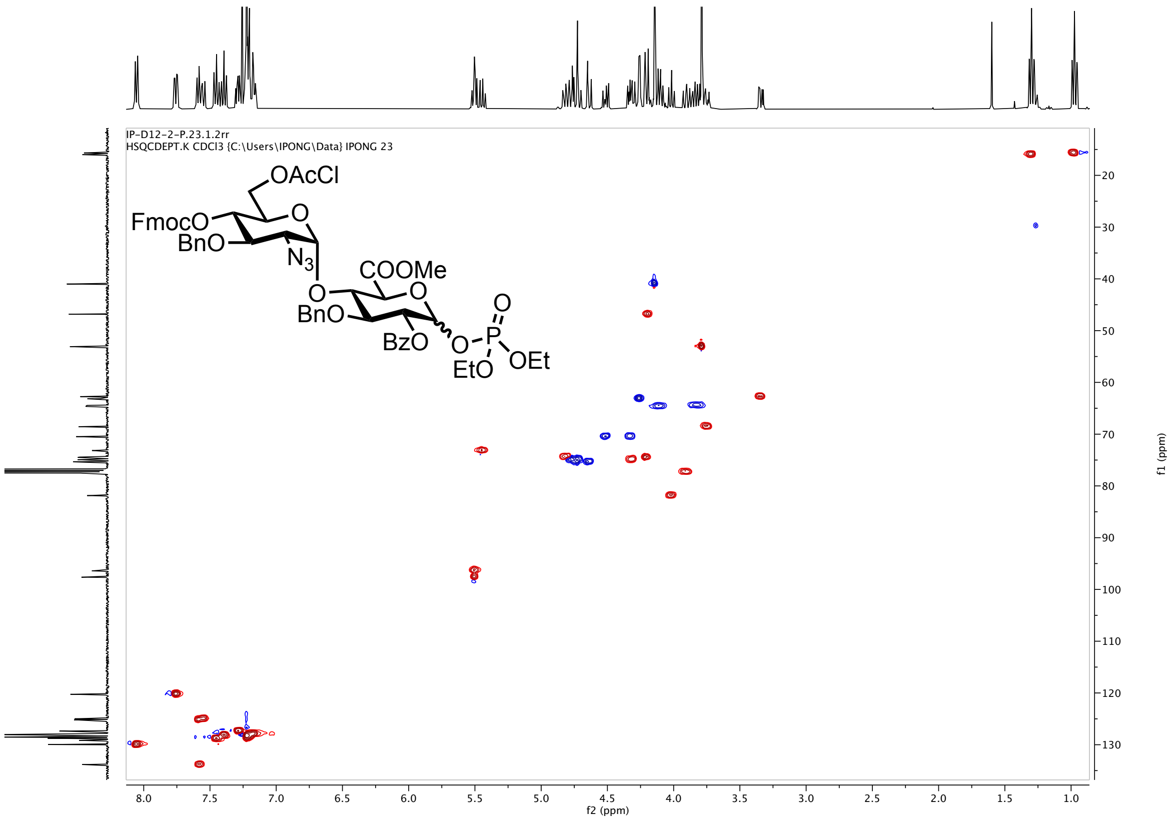

HMBC NMR (400 MHz x 101 MHz, Chloroform-*d*) 24

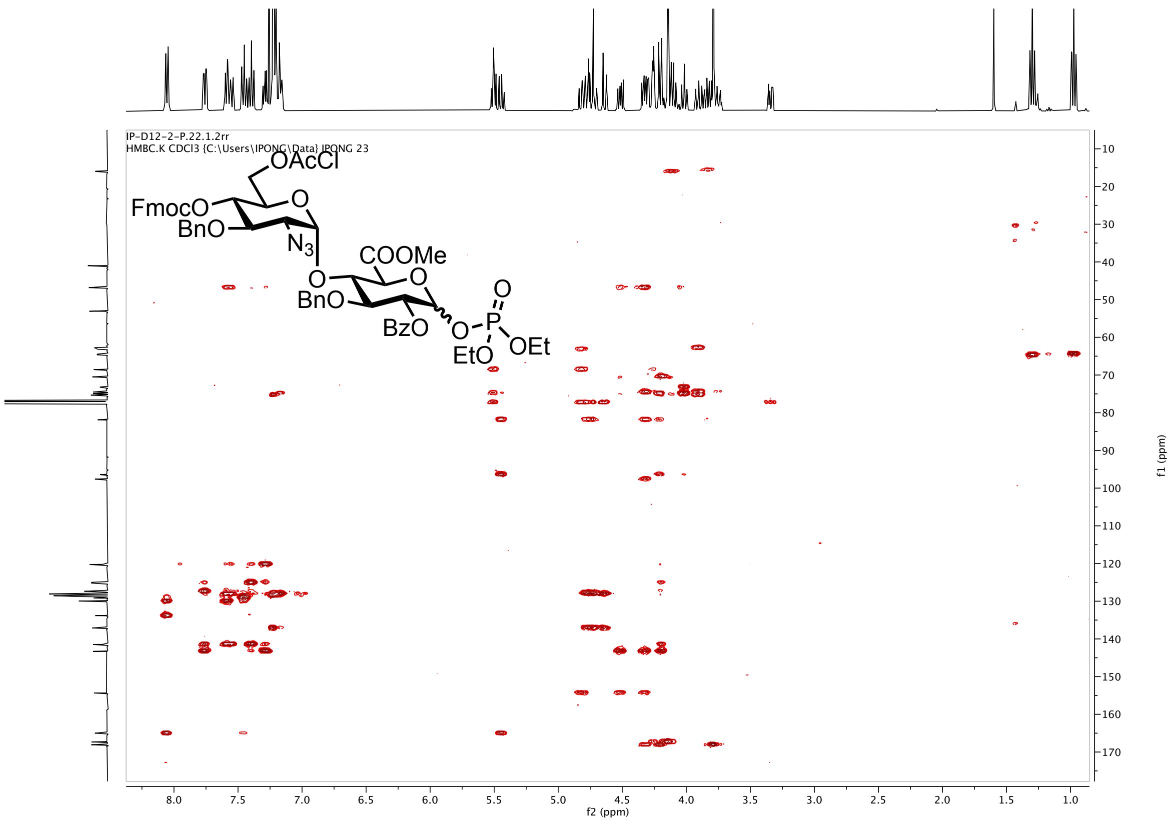

Compound 25

^{1}H NMR (400 MHz, Chloroform-*d*) 25

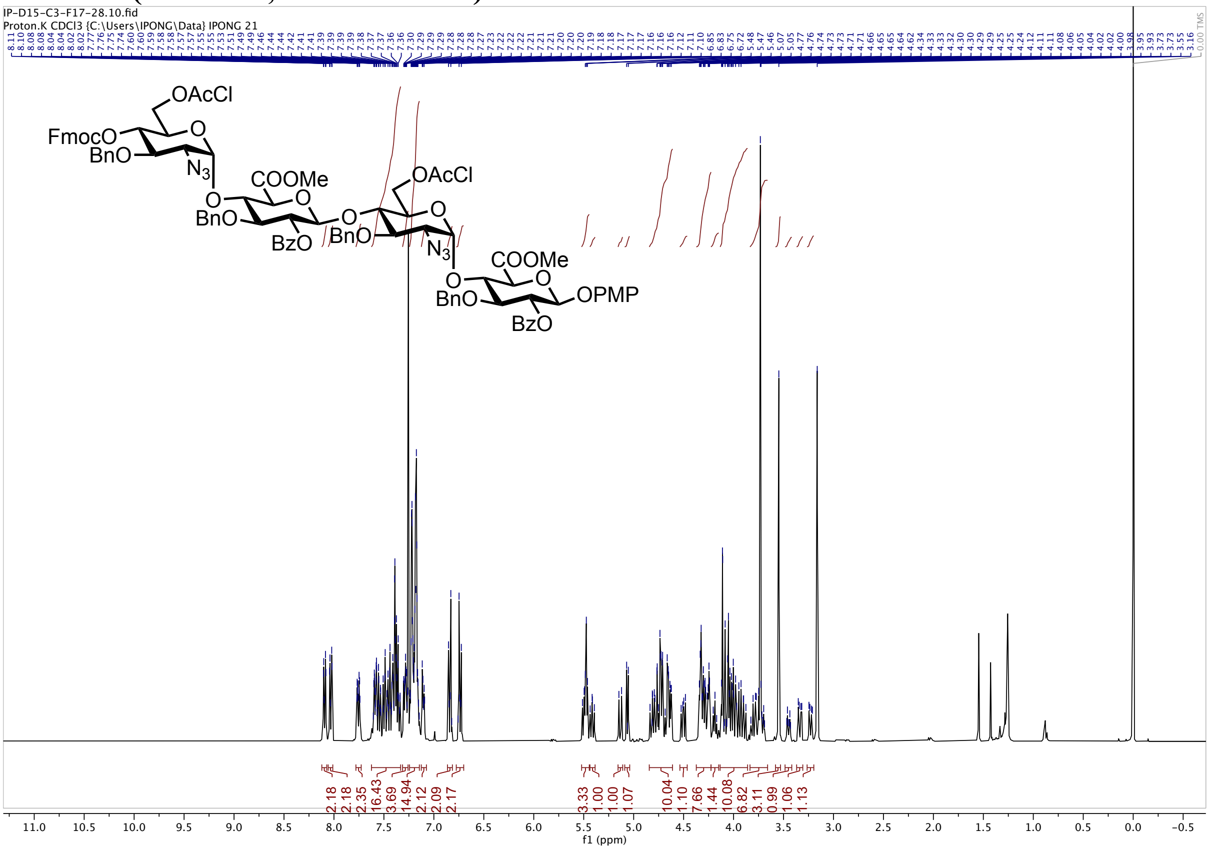

^{13}C{^{1}H} NMR (101 MHz, Chloroform-*d*) 25

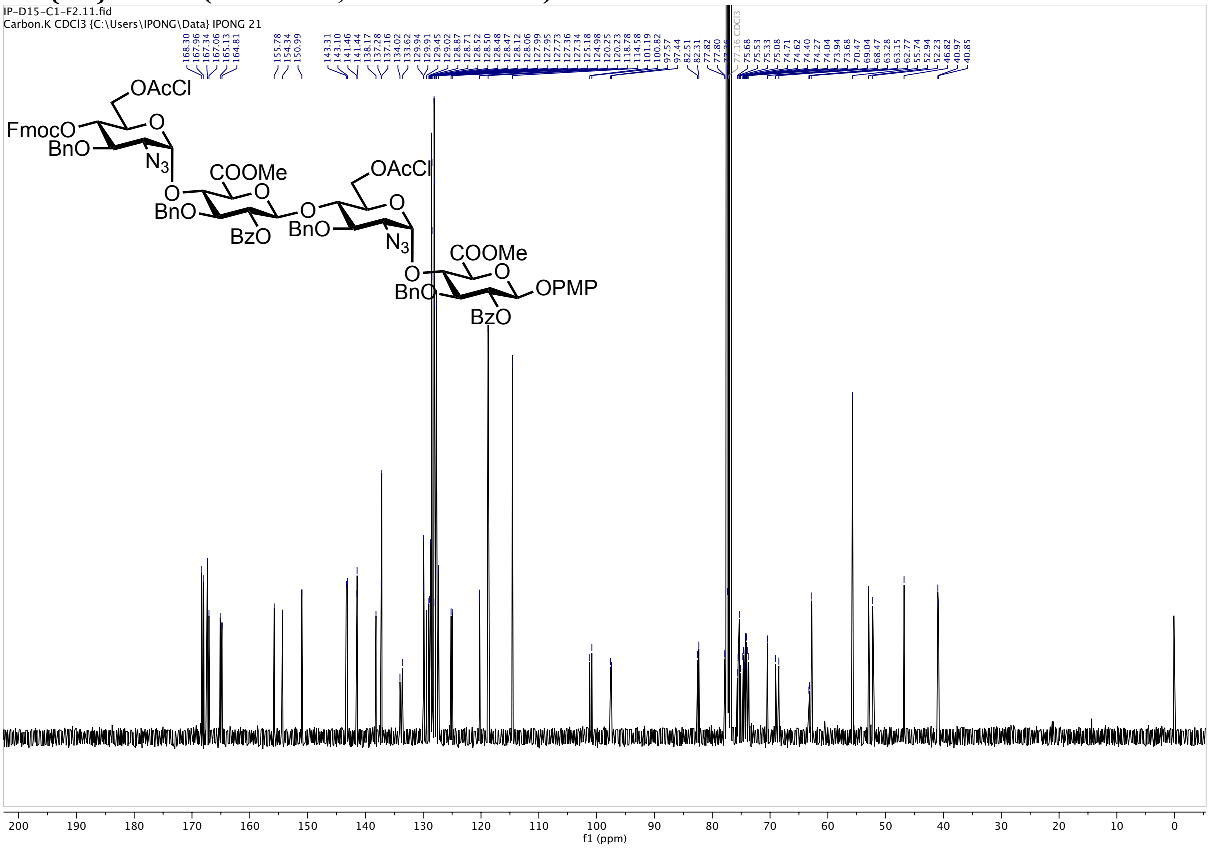

DEPT NMR (101 MHz, Chloroform-*d*) 25

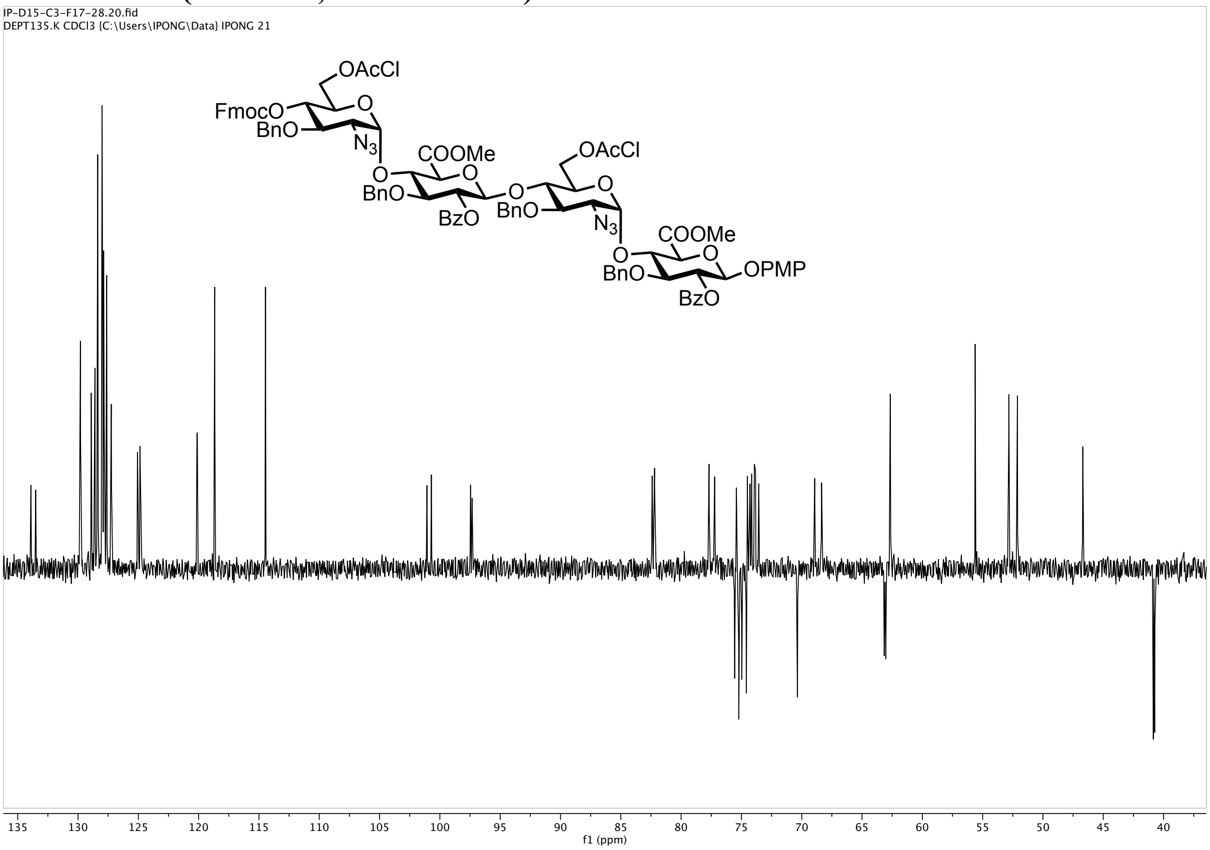

COSY NMR (400 MHz, Chloroform-*d*) 25

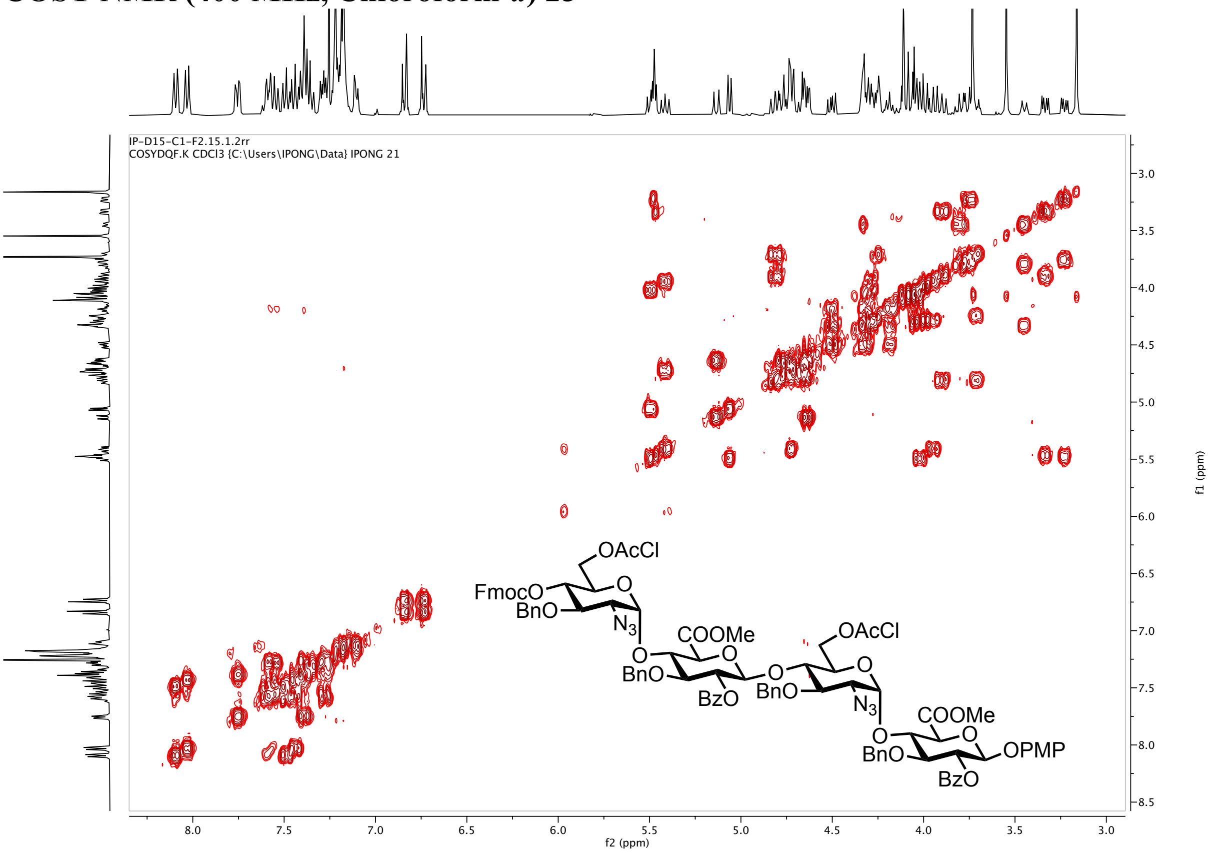

HSQC NMR (400 MHz x 101 MHz, Chloroform-*d*) 25

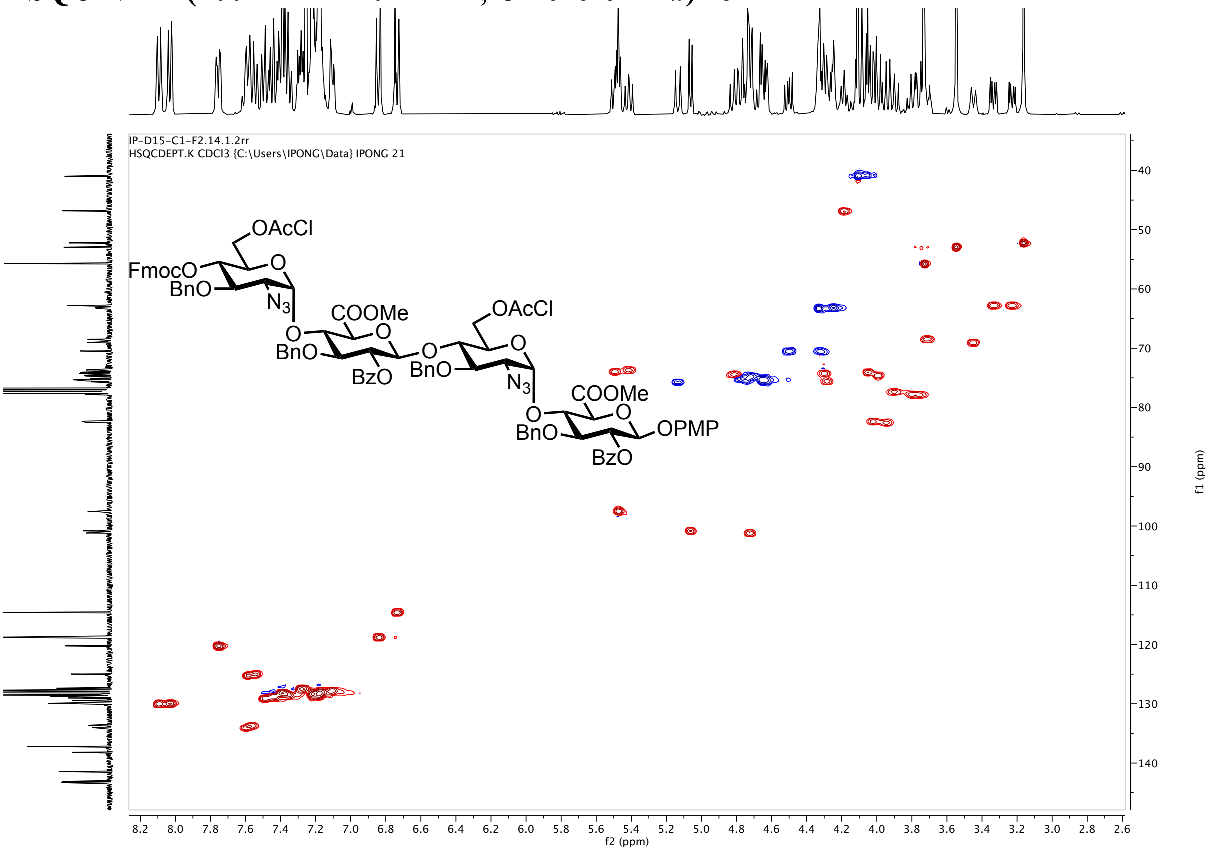

Coupled-HSQC NMR (400 MHz x 101 MHz, Chloroform-*d*) 25

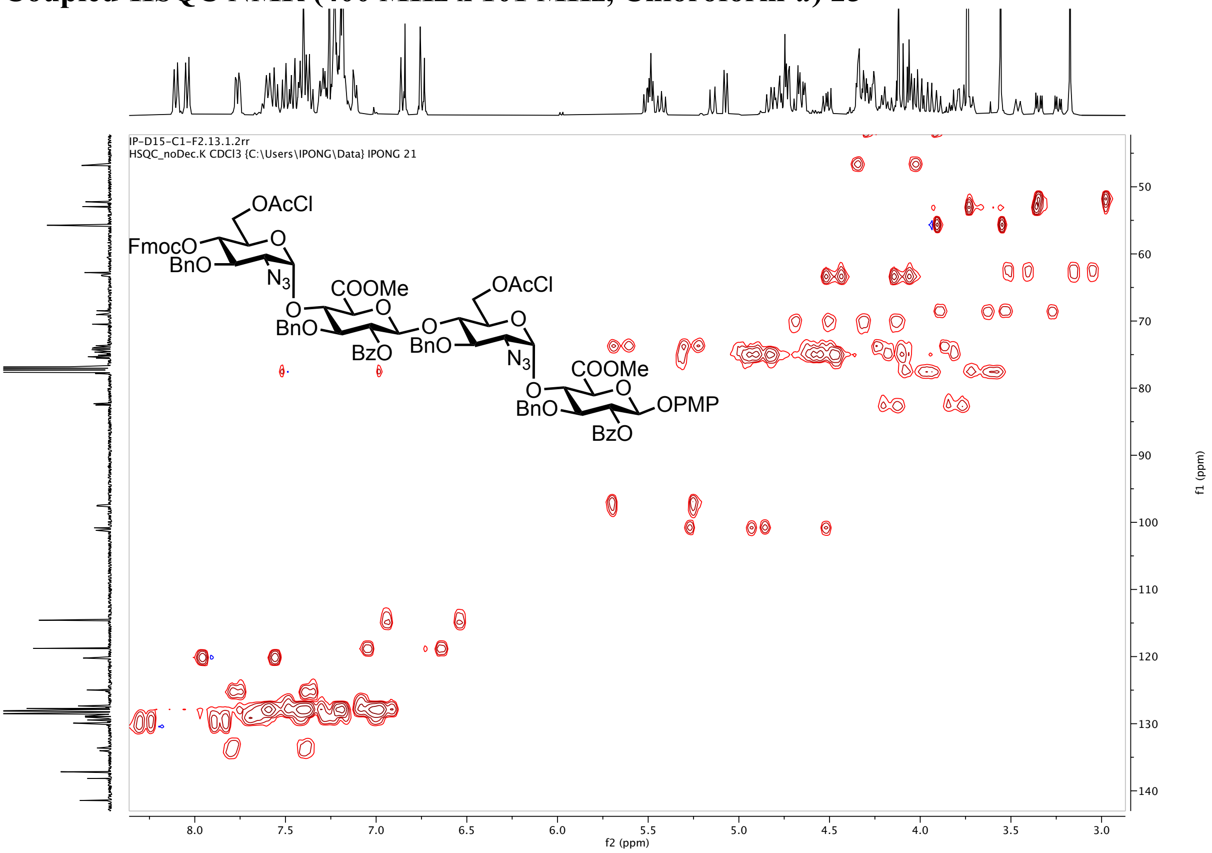

HMBC NMR (400 MHz x 101 MHz, Chloroform-*d*) 25

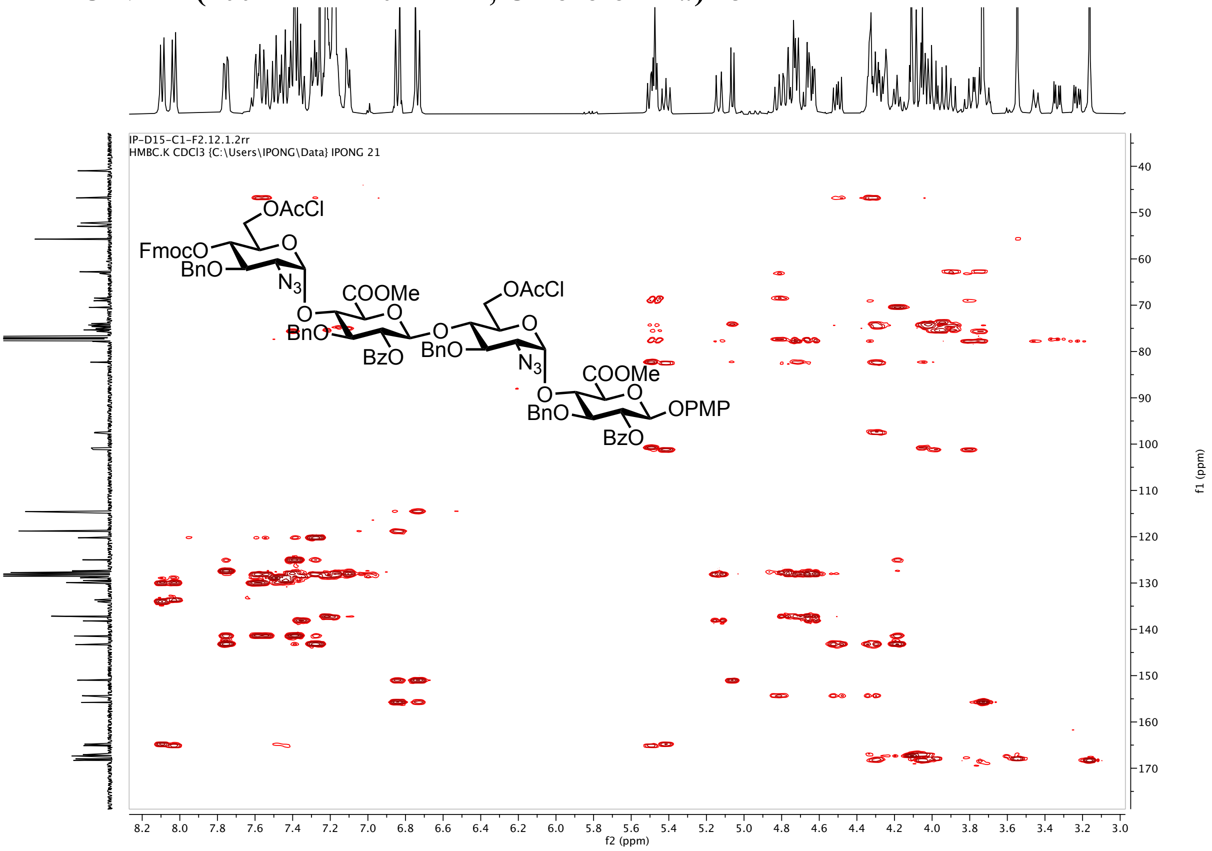

Supplement: Supplementary file 2 — jo3c01108_si_002.pdf [file jo3c01108_si_002.pdf]
